# Gene expression profiling of leukemic cells and primary thymocytes predicts a signature for apoptotic sensitivity to glucocorticoids

**DOI:** 10.1186/1475-2867-7-18

**Published:** 2007-11-28

**Authors:** Aaron L Miller, Spogmai Komak, M Scott Webb, Edward H Leiter, E Brad Thompson

**Affiliations:** 1 Department of Biochemistry and Molecular Biology, The University of Texas Medical Branch, Galveston, TX, USA; 2 Department of Surgery, The University of Texas Medical Branch, Galveston, TX, USA; 3 Diabetes and Obesity, Inflammatory Bowel Diseases, The Jackson, Laboratory, Bar Harbor, ME, USA

## Abstract

**Background:**

Glucocorticoids (GC's) play an integral role in treatment strategies designed to combat various forms of hematological malignancies. GCs also are powerful inhibitors of the immune system, through regulation of appropriate cytokines and by causing apoptosis of immature thymocytes. By activating the glucocorticoid receptor (GR), GCs evoke apoptosis through transcriptional regulation of a complex, interactive gene network over a period of time preceding activation of the apoptotic enzymes. In this study we used microarray technology to determine whether several disparate types of hematologic cells, all sensitive to GC-evoked apoptosis, would identify a common set of regulated genes. We compared gene expression signatures after treatment with two potent synthetic GCs, dexamethasone (Dex) and cortivazol (CVZ) using a panel of hematologic cells. Pediatric CD4+/CD8+ T-cell leukemia was represented by 3 CEM clones: two sensitive, CEM-C7–14 and CEM-C1–6, and one resistant, CEM-C1–15, to Dex. CEM-C1–15 was also tested when rendered GC-sensitive by several treatments. GC-sensitive pediatric B-cell leukemia was represented by the SUP-B15 line and adult B-cell leukemia by RS4;11 cells. Kasumi-1 cells gave an example of the rare Dex-sensitive acute myeloblastic leukemia (AML). To test the generality of the correlations in malignant cell gene sets, we compared with GC effects on mouse non-transformed thymocytes.

**Results:**

We identified a set of genes regulated by GCs in all GC-sensitive malignant cells. A portion of these were also regulated in the thymocytes. Because we knew that the highly Dex-resistant CEM-C1–15 cells could be killed by CVZ, we tested these cells with the latter steroid and again found that many of the same genes were now regulated as in the inherently GC-sensitive cells. The same result was obtained when we converted the Dex-resistant clone to Dex-sensitive by treatment with forskolin (FSK), to activate the adenyl cyclase/protein kinase A pathway (PKA).

**Conclusion:**

Our results have identified small sets of genes that correlate with GC-sensitivity in cells from several hematologic malignancies. Some of these are also regulated in normal mouse thymocytes.

## Background

This study was designed to find whether a common set of GC-regulated genes would be found in cell lines from several types of GC-sensitive malignancies. Several studies of malignant cells sensitive to GC-driven apoptosis identified various candidate genes regulated by GCs [1-5]. Such regulation precedes the final, irreversible activation of the apoptotic machinery. Though some genes had been detected in more than one study, detailed comparisons are difficult due to differing gene array platforms, bioinformatics methods, and cell conditions used. To avoid these problems we previously compared the genes regulated in a set of closely related clones from the CEM line of CD4, CD8 double-positive pediatric acute lymphoblastic leukemia (ALL) [6], discerning a set of genes regulated specifically in two clones sensitive to Dex-evoked apoptosis (CEM-C7–14 and CEM-C1–6) and not in a closely related Dex-resistant clone (CEM-C1–15). We now carry this work further. Treatment of the resistant clone with the phenylpyrazolo-steroid CVZ, a selective modulator of GR action [7], can provoke cell death [8-10]. Earlier work had also shown that CEM-C1–15, and its parental clone CEM-C1, can be converted to GC-sensitivity by activating the PKA pathway [11,12]. We therefore were able to compare the sets of genes regulated in C1–15 cells by Dex, which does not activate apoptosis, with those in the same cells treated with CVZ or converted to Dex-sensitive by activation of PKA. To ask whether overlapping gene sets would be found regulated in other types of malignant GC-sensitive cells, we selected three cell lines already known to be killed by GCs [13,14] and derived from other lineages. SUP-B15 is a B-cell, Philadelphia chromosome positive pediatric ALL line grown from bone marrow blasts. SUP-B15 cells express multiple B-cell, but not T-cell lineage markers [15]. The RS4;11 line, representing an adult form of ALL, was established from the bone marrow of a 32-year old Caucasian female ALL patient in relapse [16]. These cells are characterized by the t(4;11) chromosomal abnormality. Kasumi-1 is an acute myeloblastic leukemia (AML) originally obtained from the peripheral blood of a 7-year old Japanese male [17]. The cells contain a t(8;21) chromosomal abnormality which results in the expression of the AML1-ETO fusion protein, shown to bestow sensitivity to both extrinsic and intrinsic apoptotic mechanisms in leukemic cells [18]. These myeloid cells possess the highly unusual property for AML of apoptotic sensitivity to GCs [14]. As we found that indeed, a certain limited group of genes were GC-regulated in all the cells under conditions leading to GC-dependent apoptosis, we extended our process by comparing the above gene sets with genes regulated by GCs in mouse thymocytes, exquisitely GC-sensitive non-transformed cells.

A smaller set of genes were concordant with those found in the malignant human samples. Given that the experiments were done over a considerable period of time, and with evolving gene chip platforms, it is remarkable that a distinct set of genes were found to be GC-regulated during the pre-apoptotic phase, in cell lines from a variety of human GC-sensitive hematologic malignancies. While surely incomplete, the gene set found to be regulated in every instance could be useful in identifying GC-sensitive hematologic malignancies, if tested in clinical samples. We present it now to be of use to others engaged in examining such samples, noting that GC response is often also a predictor for response to many chemotherapies [19,20].

## Results

### Comparison of time course of GC-dependent cell death in several sub-types of human leukemias

**Figure 1:**
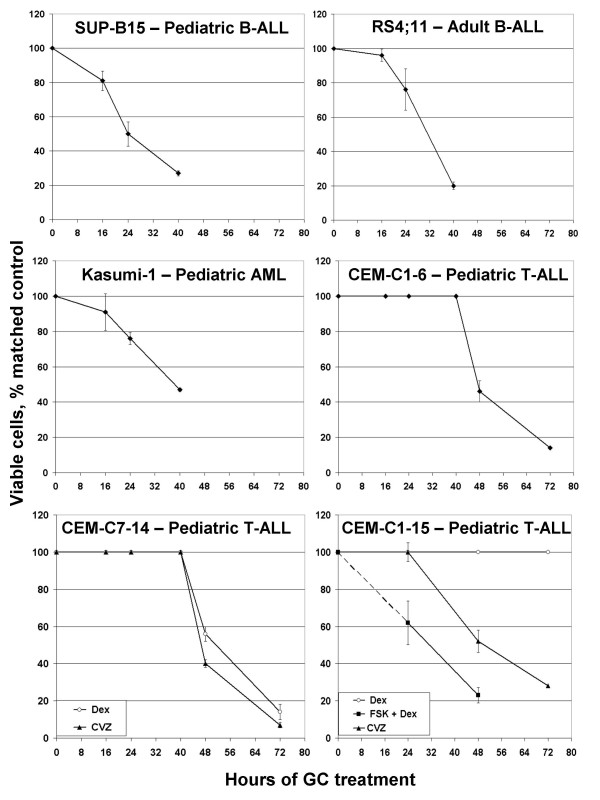
**Glucocorticoids kill multiple sub-types of leukemia**. Cells were seeded in triplicate at a starting density of 1 × 10^5 ^cells/ml and subsequently treated with vehicle or various concentrations of Dex (C7–14 and C1–15, symbol, open circle) or CVZ (C7–14 and C1–15, symbol, closed triangle). Dex-resistant CEM-C1–15 cells were restored to sensitivity by treatment with FSK and Dex (symbol, closed square, note: the "lag" time for this drug combination preceded the initial 24 hour count – dashed line 0–24 hours). Samples were analyzed by Trypan blue exclusion assay at various time-points thereafter. Presented is the percent of viable cells compared to matched control for the average of three independent replicates from vehicle vs. GC-treated at the time of evaluation. Error bars represent one standard deviation from the mean.

We have shown with CEM cells that although steroids must be continually present, their effects are reversible for many hours preceding the irreversible commitment to apoptosis. To validate their sensitivity of each cell type to GCs and define the limit of the pre-apoptotic, reversible "lag" phase, we treated with Dex or CVZ and followed viable cells over time. In preliminary experiments we determined the GC concentrations that maximized the apoptotic effect for each cell type (Methods). Counts of viable cells from GC-treated samples were compared to their matched vehicle controls (Fig. 1). Though its length varied, each human cell line showed a pre-apoptotic "lag" phase, during GC treatment of many hours, after which increased cell death was observed, except for resistant clone CEM-C1–15. When these were treated with CVZ or converted to Dex-sensitive through PKA stimulation [12], they also showed a lag. SUP-B15 cells were the most rapidly sensitive to GC; at a concentration of 10^-7 ^M, Dex reduced viable cell numbers to 50% of controls by 24 hours. Sensitive clones CEM-C7–14 and CEM-C1–6 (the latter a spontaneous revertant from a resistant clone) required the longest period (40 hours) of exposure to GC before initiation of the apoptotic response. As we had observed previously for CEM-C7–14 cells, treatment with CVZ, a more potent GC with the ability to selectively modulate GR action [7], increased cell death over that by Dex. Both RS4;11 and Kasumi-1 cells reacted similarly in regard to timing, but with differences in sensitivity, as the Dex concentration required for maximum apoptotic potency of Kasumi-1 cells was 10-fold less than for RS4;11. Only a brief meeting report documented the GC-sensitivity of SUP-15 and RS4;11 cells [13]. Our results confirm and extend that report.

As is well known, primary mouse thymocytes *in vitro *rapidly respond to GCs with overt apoptosis [21]. We found that under our experimental conditions, apoptosis of thymocytes began after about 1.5 hours of continued exposure *in vitro *to 10^-7 ^M Dex (data not shown). Together, these data confirm and/or extend prior reports and establish the dose and timing parameters used during the remainder of the studies.

### Sequential comparison of gene expression

For gene expression comparisons of all the malignant lines, cells were taken for RNA extraction during the late lag phase of pharmacological treatment, that is before overt apoptosis and at a time that removal of steroid would allow recovery of virtually all cells. This corresponded to the time at which empirical data showed a small fraction of the cells exhibiting phosphatidylserine eversion by annexin-v assay. Since thymocytes in primary culture are not growing, we extracted them after 1.5 hours of exposure to Dex, a time when cell viability did not differ appreciably from controls.

**Figure 2:**
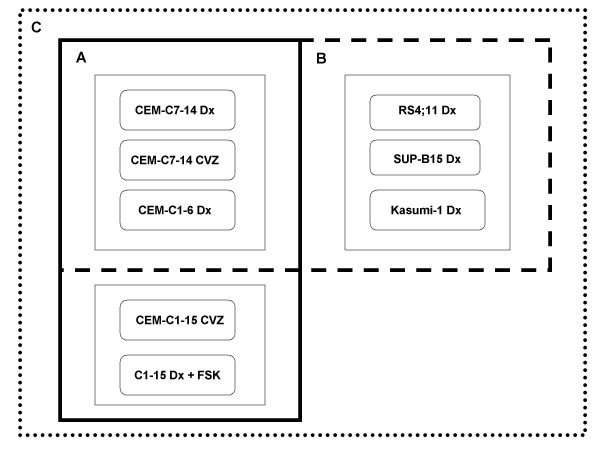
**Flow chart of various comparative schemes elucidates multiple GC-mediated gene signatures**. (A) CEM Signature: All genes regulated in common in the same sense in GC-treated CEM-C7–14, CEM-C1–6, CEM-C1–15 CVZ, and CEM-C1–15 Dex plus FSK cells were obtained (solid line box). This list was subsequently compared to Dex-treated resistant CEM-C1–15 cells and to mouse thymocytes. (B) Multiple Leukemia Signature: All genes regulated in common in the same sense in GC-treated CEM-C7–14, CEM-C1–6, RS4;11, SUP-B15, and Kasumi-1 cells were obtained (dashed line box). As before, this list was compared to Dex-treated resistant CEM-C1–15 cells and to mouse thymocytes. (C) Composite Signature: All genes regulated in common in the same sense in GC-treated CEM-C7–14, CEM-C1–6, RS4;11, SUP-B15, Kasumi-1, CEM-C1–15 CVZ, and CEM-C1–15 Dex plus FSK were compiled into a final list (spotted line box). This list was compared to Dex-treated resistant CEM-C1–15 and to mouse thymocytes.

The data from all experiments were analyzed in a concatenated series (Fig. 2). In overview, the sequence was: 1) identification of those genes regulated ≥ 1.2-fold in every CEM cell type under conditions that produced GC-dependent apoptosis; 2) notation (asterisk, Table 1 and Additional file 1) of those genes found similarly regulated in Dex-treated but apoptosis-resistant CEM-C1–15 cells; 3) retention of genes regulated in CEM-C1–15 cells *but in the opposite sense *from the GC-sensitive cells; 4) application of Spotfire^® ^statistical software to the regulated genes common to CEM cells under all GC-sensitive conditions. In Additional file 1, all the genes from step 1 are retained, with notation if not unique to GC-sensitive cells, (e.g. seen in CEM-C1–15). This resulted in a "signature" list of genes always regulated in CEM cells under conditions in which they were GC-sensitive (Fig. 2A). Usually, these differences in mRNA levels were found to be statistically significant.

**Table 1:** Gene expression signature for GC-sensitivity in CEM cells. (Signaling Network Genes)

		**Sensitive**	**Sensitive**	**Sensitive**	**Sensitive**	**Resensitized**	**Resistant**
**Name**	**Description**	**C7–14 Dx**	**C7–14 Z**	**C1–6 Dx**	**C1–15 Z**	**C1–15 Dx+F**	**C1–15 Dx**

AK2	adenylate kinase 2	-1.4	**-1.7**	**-1.6**	**-1.6**	-1.4	*
ARHGEF7	Rho guanine nucleotide exchange factor (GEF) 7	1.3	1.4	1.3	**1.3**	1.8	1.2
BCL2L11	BCL2-like 11 (apoptosis facilitator)	**3.1**	**14.5**	**5.9**	1.3	1.8	*
CALR	calreticulin	**-1.9**	**-1.7**	-1.6	-1.4	-1.6	-1.3
CDC6	CDC6 cell division cycle 6 homolog (S. cerevisiae)	**-1.3**	-1.2	**-1.5**	**-1.3**	-1.3	-1.3
DDIT4	DNA-damage-inducible transcript 4	**4.4**	**3.4**	2.5	1.2	4.4	1.5
FGFR1	fibroblast growth factor receptor 1	**1.6**	**3.1**	1.4	**1.7**	1.7	1.3
FKBP4	FK506 binding protein 4, 59 kDa	-1.9	**-2.1**	-1.3	**-1.5**	-1.6	*
FKBP5	FK506 binding protein 5	**6.6**	**11.2**	**4.4**	**3.8**	2.9	**2.8**
HDAC1	histone deacetylase 1	**-1.4**	-1.2	-1.2	**-1.3**	-1.2	-1.3
HRAS	v-Ha-ras Harvey rat sarcoma viral oncogene homolog	**-1.3**	-1.4	-1.3	**-1.4**	-1.4	*
ID1	inhibitor of DNA binding 1, dominant negative helix-loop-helix protein	-2.6	**-1.7**	-1.2	**-1.9**	-1.6	**-1.4**
IFRD1	interferon-related developmental regulator 1	**-1.8**	-1.2	-1.6	**-1.6**	-1.3	*
IL7R	interleukin 7 receptor	**8.3**	**18.0**	**6.3**	1.4	2.5	*
ITGA6	integrin, alpha 6	**5.0**	**10.2**	**2.4**	**6.6**	2.8	**1.6**
MAP4	microtubule-associated protein 4	**-1.3**	**-1.8**	-1.7	-1.3	-1.3	*
MT1A	metallothionein 1A (functional)	1.3	**1.3**	**2.2**	**1.4**	1.4	*
MTHFD1	methylenetetrahydrofolate dehydrogenase (NADP+ dependent) 1	**-1.4**	**-1.9**	**-1.5**	-1.5	-1.2	*
MYC	v-myc myelocytomatosis viral oncogene homolog (avian)	**-3.8**	**-4.6**	**-3.6**	-1.4	-2.2	*
NFKBIA	nuclear factor of kappa light polypeptide gene enhancer in B-cells inhibitor, alpha	**3.0**	**3.4**	**2.8**	**1.9**	1.5	**1.3**
NME1	non-metastatic cells 1, protein (NM23A) expressed in	**-1.5**	**-2.2**	**-1.6**	**-1.4**	-1.3	*
NR3C1	nuclear receptor subfamily 3, group C, member 1 (glucocorticoid receptor)	**4.2**	**6.6**	**2.1**	**1.7**	1.6	*
OGT	O-linked N-acetylglucosamine (GlcNAc) transferase	1.3	**1.7**	**2.0**	**1.6**	1.3	*
PIAS2	protein inhibitor of activated STAT, 2	1.2	1.4	1.5	**2.0**	1.3	*
PIK3R1	phosphoinositide-3-kinase, regulatory subunit 1 (p85 alpha)	**2.5**	**2.4**	1.7	**2.0**	1.9	*
RBMS1	RNA binding motif, single stranded interacting protein 1	1.4	2.0	1.7	**1.6**	1.5	**1.6**
SCARB1	scavenger receptor class B, member 1	**-1.7**	**-2.2**	-1.2	**-1.5**	-1.2	**-1.3**
SRM	spermidine synthase	**-1.8**	**-3.3**	**-1.7**	**-1.4**	-1.4	*
TAGLN2	transgelin 2	**-1.4**	**-2.0**	-1.3	-1.2	-1.2	*
TRAF3IP2	TRAF3 interacting protein 2	1.6	**2.1**	1.8	1.5	1.4	1.3
TSC22D3	TSC22 domain family, member 3	**33.1**	**74.0**	**20.4**	**7.4**	14.3	**5.0**
TXNIP	thioredoxin interacting protein	**2.8**	**3.4**	**3.7**	1.4	2.0	1.2
UBE2S	ubiquitin-conjugating enzyme E2S	-1.6	**-1.6**	-1.3	**-1.6**	-1.3	*
VCL	vinculin	1.8	**1.6**	**1.9**	**1.3**	1.5	*
YAF2	YY1 associated factor 2	**1.8**	**2.4**	**1.6**	**1.7**	1.3	1.6

Regulated transcripts from vehicle or GC-treated samples were evaluated and compared using Spotfire^® ^and Ingenuity^® ^bioinformatics software. Selection criteria for each gene were as follows: probe called present (Methods) on the microarray and regulated ≥ 1.2-fold (net 20% change). GC-mediated transcripts regulated in common in the same direction among sensitive CEM-C7–14 Dex (C7–14 Dx) and CVZ (C7–14 Z), CEM-C1–6 (C1–6 Dx), CEM-C1–15 CVZ (C1–15 Z), and CEM-C1–15 Dex plus FSK (C1–15 Dx+F) cells were obtained. This comparison resulted in 96 regulated genes (see Additional file 1). This list was subsequently compared to Dex-treated resistant CEM-C1–15 cells (C1–15 Dx). Shown is a subset of 35 genes from the original 96 that could be linked via a signaling network (symbol, closed diamond in Additional file 1). Bold type indicates statistically significant regulation p ≤ 0.05 between means of vehicle vs. GC-treated. Symbol, asterisk = gene "absent" by selection criteria.

Similarly, a "signature" gene set was derived for GC sensitivity for all the leukemic cell lines: 1) compile genes regulated ≥ 1.2-fold in every cell line, without regard to statistical significance; 2) annotate genes Dex-regulated in resistant C1–15 cells; 3) apply statistics. Genes regulated similarly in every GC-sensitive cell type (Fig. 2B, C) comprise a possible "signature" for multiple leukemias. Again, in most cases these were found statistically significant.

Separately, each gene set was compared to the genes regulated by Dex in mouse thymocytes, to see whether some were also present in the pre-apoptotic phase in non-transformed mammalian cells.

### Comparison of CEM cell clones reveals a signature for GC-evoked apoptosis

GC-sensitive clones CEM-C7–14 and CEM-C1–6 were treated in triplicate with vehicle alone or 10^-6 ^M Dex. CEM-C7–14 cells were also treated with the more potent GR modulator CVZ. The CEM-C1–15 sister clone is resistant to as much as 4.0^-6 ^Dex but can be killed by CVZ. Therefore these cells were treated with 10^-6 ^M Dex or 10^-6 ^M CVZ. Alternatively, we made use of the fact that CEM-C1–15 cells can be converted to Dex-sensitive by activating the adenyl cyclase/PKA signaling pathway [12] and (Fig. 1). We refined the gene set obtained from the steroids-only treatments by adding the requirement that they were also regulated in C1–15 cells after FSK plus Dex exposure. (See Fig. 2A, solid box.)

**Figure 3:**
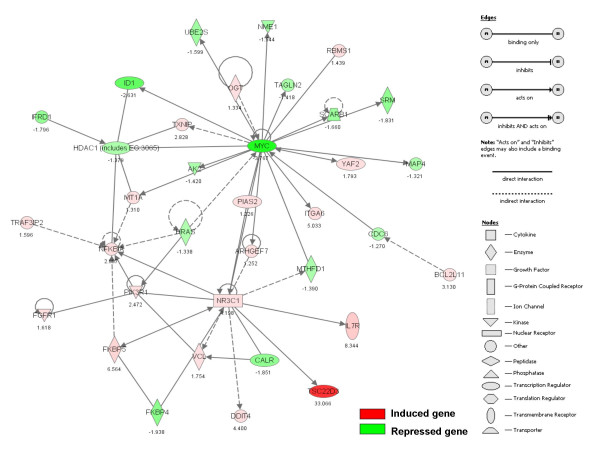
**A signaling network links genes regulated by GCs in CEM cells**. Ingenuity^® ^bioinformatics pathway analysis tool was used to connect a subset of 35 genes from the CEM signatory list (Table 1, and symbol, closed diamond in Additional file 1) based upon a database of published observations. Symbols for genes representing specific categories of cellular molecules as well as interactive relationships are depicted in the legend. Color gradations are based upon gene regulation at the fold-change level. Red color: induced gene; green color: repressed gene. Fold-change data from CEM-C7–14 cells treated with Dex are presented as representative of the CEM signature.

Initially, all genes were listed whose mRNAs if "present" as judged by Affymetrix software, were changed 20% or more in the two Dex-treated, Dex-sensitive clones, in one of these treated with 10-fold less CVZ, in the Dex-resistant clone treated with high-dose CVZ, and in the Dex-resistant clone treated with FSK plus Dex. After this list was prepared, the data was evaluated statistically with the exception of the FSK-sensitized C1–15 cell samples, for which only duplicate experiments were performed. This analysis resulted in 96 regulated genes, of which 48 were induced and 48 were repressed (see Additional file 1). Statistical analysis of the means from both control and GC-treated samples were used to generate p-values (bold type in the table). Of the 96 genes, 43 showed a suggestion of weak regulation by Dex in the resistant clone CEM-C1–15, though usually without reaching statistical significance. Of the 43, GSK3B and IL10RB were found to be regulated in opposite directions between Dex-sensitive and -resistant CEM clones. Through the use of Ingenuity^® ^pathway analysis, a signaling network was revealed, linking 35 of the 96 genes. (Fig. 3, Table 1, the 35 also are designated by, closed diamond in Additional file 1). It is obvious from the network that two major signal transduction nodes exist: 1) affiliation with induction of NR3C1 – the GR; and 2) affiliation with repression of MYC. Minor signaling nodes appear routed through HDAC1, NFKBIA, and PIK3R1. These data suggest that the gene set identified includes genes involved in a relevant signaling pathway, and are not just a random assortment that happen to be GC-regulated. In an independent study of the time course of response in CEM-C7–14 exposed to Dex, we found that the change in these genes was progressive over the interval of steroid exposure (M.S. Webb and E.B. Thompson, manuscript submitted).

To determine which of the conserved GC-regulated genes, if any, were also regulated in a naturally non-transformed sensitive lymphoid cell system, we compared regulation between human CEM cells and mouse thymocytes. The latter are exquisitely sensitive to GCs, undergoing apoptosis within hours of exposure instead of days as for the malignant cells [21]. Examination of the conserved gene set yielded 34 transcripts that were regulated in thymocytes also. However, of these, only 20 were regulated in the same sense in the thymocytes; the other 14 were regulated in the opposite sense. (see Additional file 1, symbol, closed circle). These data suggest that there is a shared pathway of GC-regulated genes, relevant to apoptosis, in both malignant and normal mammalian lymphoid cells.

### Genes regulated by GCs in multiple sub-types of GC-sensitive leukemias show a common genetic signature

To test for a common signature for GC-sensitivity among various leukemic cells, we compared cell lines representing three types of GC-sensitive leukemias with the CEM cells (Fig. 2B, dashed-line box). From literature reports of GC-sensitivity [13,14], we chose SUP-B15 (pediatric B-cell ALL), RS4;11 (an adult B-cell ALL), and Kasumi-1 (a special type of GC-sensitive pediatric AML). As in CEM cells, preliminary experiments established both optimal concentrations of GCs and timing of phosphatidylserine membrane eversion for each cell line to capture gene expression data before irreversible onset of apoptosis. Comparison of GC-sensitive CEMs with RS4;11, SUP-B15, and Kasumi-1 identified 122 genes, 52 induced and 70 repressed, all regulated ≥ 1.2-fold in the same sense in all cell lines. These were then tested for statistical significance (see Additional file 2). Of these genes, 26 were also found to be regulated by Dex in the Dex-resistant CEM-C1–15 cells in the same sense and thus are not of themselves both necessary and sufficient for evoking apoptosis. Four genes, FOXO3A, SFRS2, SLC7A1, and PAI-RBP1 were regulated in a different sense (e.g. induced vs. repressed) between sensitive cells and the resistant CEM-C1–15 cells. As had been done with CEM cells alone, the 122 gene set was compared to the mouse thymocyte data. Thirty-three genes were also found regulated in the thymus; 19 in the same sense as in the GC-sensitive leukemias and 14 in the opposite sense (see Additional file 2 symbol, closed circle).

### Quantitative real-time PCR confirms GC-regulation of an induced and repressed gene in multiple hematologic malignancies

**Figure 4:**
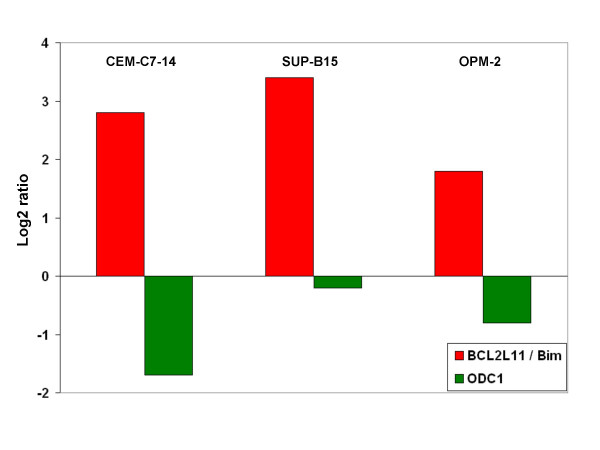
**Quantitative real-time PCR confirms regulation of BCL2L11/Bim and ODC1 by Dex in multiple hematologic malignancies**. CEM-C7–14, SUP-B15, and OPM-2 cells were diluted to 4 × 10^5 ^cells/ml and treated with ethanol vehicle, 10^-6 ^M Dex (C7–14 and OPM-2), or 10^-7 ^M Dex (SUP-B15) for 24 hours. RNA was extracted and qRT-PCR was performed for BCL2L11/Bim and ODC1 transcripts. The log2 ratio for BCL2L11/Bim and ODC1 are presented. Regulation of both transcripts was determined to be statistically significant p ≤ 0.05 when compared to the internal standard after three independent PCR reactions.

As proof-of-principle that our approach was valid, we confirmed the regulation of two genes established as important for GC-sensitivity: 1) the pro-apoptotic Bcl-2 family member (BCL2-like 11, BCL2L11/Bim), a GC-induced gene; and 2) ornithine decarboxylase (ODC1), a MYC-dependent, GC-repressed gene [22-24] in two cell lines evaluated by microarray (CEM-C7–14 and SUP-B15, see Additional file 2) and the GC-sensitive multiple myeloma cell line OPM-2, which was not evaluated for global gene changes in this array study. Fig. 4 shows the results from real-time PCR assays for BCL2L11 and ODC1 regulation in CEM-C7–14, SUP-B15, and OPM-2 cells treated with Dex for 24 hours. Both genes were confirmed as regulated, one induced and the other repressed in all three cell lines.

### Refinement of the GC-sensitivity signature by comparison of genes regulated in naturally sensitive and resistant-restored-to-sensitive cells

The gene set derived by comparing responses in all the cell clones and lines inherently sensitive to GC-evoked apoptosis (Fig. 2B) was compared to the set regulated by Dex-resistant CEM-C1–15 cells treated with high concentration CVZ (10^-6 ^M) or to Dex in combination with FSK, the activator of the adenyl cyclase/PKA pathway (Fig. 2C, large dotted-line box). Genes regulated in common under all of these conditions were identified as possible significant players in the role of the GC-mediated apoptotic response (Table 2). A total of 27 genes met these criteria, and of these 17 were shown to be induced while 10 were observed as repressed. Nearly all genes were found to be statistically significantly regulated by GC (bold type) where the calculation could be applied. In Dex-resistant CEM-C1–15 cells, 14 of these 27 genes were also seen to be regulated by Dex.

**Table 2:** Gene expression signature for GC-sensitivity among sensitive and Dex-resistant-restored-to-sensitive leukemias

**GC-response**		**Sen**.	**Sen**.	**Sen**.	**Sen**.	**Sen**.	**Sen**.	**Sen**.	**Resen**.	**Res**.
**Patient-derived line**		**Ped**.	**Ped**.	**Ped**.	**Adult**	**Ped**.	**Ped**.	**Ped**.	**Ped**.	**Ped**.

**Cell lineage**		**T-cell**	**T-cell**	**T-cell**	**B-cell**	**B-cell**	**Myeloid**	**T-cell**	**T-cell**	**T-cell**

**Sub-type of leukemia**		**ALL**	**ALL**	**ALL**	**ALL**	**ALL**	**AML**	**ALL**	**ALL**	**ALL**

**Name**	**Description**	**C7–14 Dx**	**C7–14 Z**	**C1–6 Dx**	**RS4 Dx**	**SUP Dx**	**Kas Dx**	**C1–15 Z**	**C1–15 Dx+F**	**C1–15 Dx**

AK2	adenylate kinase 2	-1.4	**-1.7**	**-1.6**	**-1.4**	**-2.2**	**-3.3**	**-1.6**	-1.4	*
BCL2L11	BCL2-like 11 (apoptosis facilitator)	**3.1**	**14.5**	**5.9**	**1.2**	**3.1**	**5.1**	1.3	1.8	*
BTG1	B-cell translocation gene 1, anti-proliferative	**9.3**	**9.1**	**4.4**	1.2	**3.9**	**8.8**	**3.3**	1.7	1.9
CD53	CD53 antigen	**3.1**	**3.4**	**3.2**	**1.4**	**3.1**	**7.2**	**1.5**	1.8	**1.4**
CDC6	CDC6 cell division cycle 6 homolog (S. cerevisiae)	**-1.3**	-1.2	-1.5	**-1.7**	**-2.9**	**-4.7**	**-1.3**	-1.3	-1.3
DDIT4	DNA-damage-inducible transcript 4	**4.4**	**3.4**	2.5	**5.5**	**4.6**	**22.4**	1.2	4.4	1.5
DSCR1	Down syndrome critical region gene 1	**5.0**	**8.2**	**4.8**	**1.5**	**7.7**	**7.9**	**1.4**	1.6	*
EIF3S9	eukaryotic translation initiation factor 3, subunit 9 eta	**-1.5**	**-2.2**	-1.3	**-1.2**	**-2.0**	**-2.2**	-1.2	-1.2	*
FADS1	fatty acid desaturase 1	-1.5	**-4.1**	**-1.9**	**-1.5**	**-1.5**	**-1.8**	-1.9	-1.3	-1.3
FKBP5	FK506 binding protein 5	**6.6**	**11.2**	**4.4**	**7.0**	**21.4**	**13.7**	**3.8**	2.9	**2.8**
IDH3A	isocitrate dehydrogenase 3 (NAD+) alpha	**-1.5**	**-1.3**	**-1.5**	**-1.6**	**-1.7**	**-2.4**	**-1.2**	-1.3	*
MARS	methionine-tRNA synthetase	-1.5	**-1.6**	-1.5	**-1.5**	**-2.2**	**-2.2**	**-1.2**	-1.3	*
MEP50	WD repeat domain 77	**-1.7**	**-1.4**	-1.8	**-1.8**	**-1.5**	**-1.4**	**-1.5**	-1.3	*
MT1X	metallothionein 1×	1.4	**2.1**	2.2	**1.6**	**1.8**	**1.7**	**1.3**	1.4	*
NFKBIA	NFκB inhibitor, alpha	**3.0**	**3.4**	**2.8**	**1.9**	**2.5**	**5.5**	**1.9**	1.5	**1.3**
OGT	O-linked N-acetylglucosamine (GlcNAc) transferase	1.3	**1.7**	**2.0**	**2.0**	**1.3**	**2.1**	**1.6**	1.3	*
PA2G4	proliferation-associated 2G4, 38 kDa	**-1.7**	**-1.9**	-1.7	-1.3	**-2.5**	**-3.5**	-1.3	-1.2	*
PARD3	par-3 partitioning defective 3 homolog (C. elegans)	1.6	**1.7**	**1.4**	**2.5**	1.5	**3.0**	1.3	1.4	*
PRG1	proteoglycan 1, secretory granule	2.6	**4.5**	**3.2**	**2.7**	**1.5**	**2.7**	1.2	1.9	**1.6**
RAPGEF2	Rap guanine nucleotide exchange factor (GEF) 2	1.7	**1.7**	1.4	**1.8**	**2.0**	**4.2**	**1.3**	1.3	*
RBMS1	RNA binding motif, single stranded interacting protein 1	1.4	2.0	1.7	**3.0**	**4.3**	**2.3**	**1.6**	1.5	**1.6**
SCARB1	scavenger receptor class B, member 1	**-1.7**	**-2.2**	-1.2	**-1.4**	**-4.9**	**-33.3**	**-1.5**	-1.2	**-1.3**
TFPI	tissue factor pathway inhibitor	**3.1**	**5.4**	**1.9**	**4.9**	**9.6**	**24.6**	**2.0**	1.8	**1.7**
TSC22D3	TSC22 domain family, member 3	**33.1**	**74.0**	**20.4**	**5.2**	**17.5**	**20.0**	**7.4**	14.0	**5.0**
TXNIP	thioredoxin interacting protein	**2.8**	**3.4**	**3.7**	**10.0**	**7.7**	**2.1**	1.4	2.0	1.2
YAF2	YY1 associated factor 2	**1.8**	**2.4**	**1.6**	**1.7**	**1.9**	**5.8**	**1.7**	1.3	1.6
ZNF259	zinc finger protein 259	**-1.4**	-1.5	**-1.8**	**-1.2**	-1.3	**-1.6**	-1.3	-1.2	*

The 122 genes obtained from comparison of both pediatric (Ped.) and adult GC-sensitive (Sen.) leukemias (see Additional file 2) were compared to Dex-resistant, CVZ-sensitive CEM-C1–15 CVZ (C1–15 Z), and resistant-restored-to-sensitive (Resen.) CEM-C1–15 Dex plus FSK (C1–15 Dx+F) regulated transcripts. These comparisons resulted in 27 genes regulated in the same sense among all naturally GC-sensitive and resistant-restored-to-sensitive human leukemias. This list was subsequently compared to Dex-treated resistant (Res.) CEM-C1–15 cells (C1–15 Dx). Bold type indicates statistically significant regulation p ≤ 0.05 between means of vehicle vs. GC-treated. Symbol, asterisk = gene "absent" by selection criteria.

When we compared these 27 genes to Dex-treated thymocytes to obtain those conserved between human and mouse (Table 3) we found 10 genes (8 induced, 2 repressed). Two of the 10 were regulated in the opposite sense in the mouse cells (Table 3, OGT and PA2G4, symbol, closed circle). Six of the 10 genes were also regulated by Dex even in resistant CEM-C1–15 cells. All 10 however, were regulated in CEM-C1–15 cells treated with CVZ or with FSK plus Dex. We conclude that certain genes are regulated by GC in normal mouse thymocytes as they are in several types of GC-sensitive human leukemias. Among the leukemia types tested, we have identified a set of genes that appear to give a partial profile for sensitivity for GC-driven apoptosis. These genes may give clues to the pre-apoptotic pathway. We hope that this GC-sensitive profile encourages application to clinical samples.

**Table 3:** Conserved gene expression signature for GC-sensitivity in thymocytes and multiple leukemias

**GC-response**			**Sen**.	**Sen**.	**Sen**.	**Sen**.	**Sen**.	**Sen**.	**Sen**.	**Sen**.	**Resen**.	**Res**.
**Mouse vs. Human**			**Mouse**	**Ped**.	**Ped**.	**Ped**.	**Adult**	**Ped**.	**Ped**.	**Ped**.	**Ped**.	**Ped**.

**Cell lineage**			**T-cell**	**T-cell**	**T-cell**	**T-cell**	**B-cell**	**B-cell**	**Myeloid**	**T-cell**	**T-cell**	**T-cell**

**Type of cell**			**Thymocyte**	**ALL**	**ALL**	**ALL**	**ALL**	**ALL**	**AML**	**ALL**	**ALL**	**ALL**

**Name**	**Description**	**Opp. R**	**C57/BL6 Dx**	**C7–14 Dx**	**C7–14 Z**	**C1–6 Dx**	**RS4 Dx**	**SUP Dx**	**Kas Dx**	**C1–15 Z**	**C1–15 Dx+F**	**C1–15 Dx**

BCL2L11	BCL2-like 11 (apoptosis facilitator)		**1.8**	**3.1**	**14.5**	**5.9**	**1.2**	**3.1**	**5.1**	1.3	1.8	*
CDC6	CDC6 cell division cycle 6 homolog (S. cerevisiae)		**-1.2**	**-1.3**	-1.2	-1.5	**-1.7**	**-2.9**	**-4.7**	**-1.3**	-1.3	-1.3
DDIT4	DNA-damage-inducible transcript 4		**2.9**	**4.4**	**3.4**	2.5	**5.5**	**4.6**	**22.4**	1.2	4.4	1.5
DSCR1	Down syndrome critical region gene 1		1.3	**5.0**	**8.2**	**4.8**	**1.5**	**7.7**	**7.9**	**1.4**	1.6	*
FKBP5	FK506 binding protein 5		**1.7**	**6.6**	**11.2**	**4.4**	**7.0**	**21.4**	**13.7**	**3.8**	2.9	**2.8**
NFKBIA	NFκB inhibitor, alpha		**1.5**	**3.0**	**3.4**	**2.8**	**1.9**	**2.5**	**5.5**	**1.9**	1.5	**1.3**
OGT	O-linked N-acetylglucosamine (GlcNAc) transferase	◯	-1.5	1.3	1.7	2.0	2.0	1.3	2.1	1.6	1.3	*
PA2G4	proliferation-associated 2G4, 38 kDa	◯	**1.2**	**-1.7**	**-1.9**	-1.7	-1.3	**-2.5**	**-3.5**	-1.3	-1.2	*
PRG1	proteoglycan 1, secretory granule		**1.6**	2.6	**4.5**	**3.2**	**2.7**	**1.5**	**2.7**	1.2	1.9	**1.6**
TXNIP	thioredoxin interacting protein		**2.6**	**2.8**	**3.4**	**3.7**	**10.0**	**7.7**	**2.1**	1.4	2.0	1.2

To obtain genes regulated by GCs in a conserved manner in both pediatric (Ped.) and adult leukemias, the 27 genes regulated in the same sense among all naturally GC-sensitive (Sen.) and resistant-restored-to-sensitive (Resen.) human leukemias (Table 2) were compared to mouse thymocytes (C57/BL6 Dx). This analysis identified 10 genes, 2 of which were regulated in an opposite sense regulation between human and mouse indicated by (Opp. R, symbol, closed circle). This list was also compared to Dex-treated resistant (Res.) CEM-C1–15 cells (C1–15 Dx). Bold type indicates statistically significant regulation p ≤ 0.05 between means of vehicle vs. GC-treated. Symbol, asterisk = gene "absent" by selection criteria.

## Discussion

Seeking the genes responsible for the build up over time to GC-mediated apoptosis, we have used microarrays to uncover genetic "signatures". By sequentially screening transcripts regulated in common between various sub-types of sensitive, resistant, and resistant-restored-to-sensitive leukemic human cell lines and even non-malignant mouse thymocytes, we have discovered a small list of genes regulated in common among all GC-sensitive conditions. We believe these genes to be at the core of the apoptotic signature to GCs. As might be expected, cell type-specific subsets were found, and thymocytes showed fewer genes regulated in common with the GC-sensitive malignant cells. Among the signatory genes we find BCL2-like 11 apoptosis facilitator (BCL2L11, Bim), Down syndrome critical region gene 1 (DSCR1), Thioredoxin-interacting protein (TXNIP, VDUP1), DNA-damage-inducible transcript 4 (DDIT4, RTP801, REDD1, or dig2), Nuclear factor of kappa light polypeptide gene enhancer in B-cells inhibitor, alpha (NFKBIA), FK506 binding protein 5 (FKBP5), and exclusive to the examined human leukemias GC-induced leucine zipper (TSC22D3, DSIPI, or GILZ), all of which have been investigated for possible roles in GC-stimulated apoptosis [22,25-31]. These prior studies show both anti- and pro-apoptotic actions for many of these genes studied in isolation, but no study thus far has been able to link particular genes together to fill in all the critical gaps in the apoptotic mechanism. Our results suggest that this is because these genes work as part of a network and only in the context of the particular cellular network in which they are found can their action be fully understood. Further experiments manipulating more than one gene simultaneously will be necessary to close these gaps in such networks. Nonetheless, the fact that a relatively small set of genes are implicated in several leukemic lineages speaks strongly to the universality of the key genes systems at work in GC-dependent cell death.

### Core profile genes have been shown relevant to GC-dependent apoptosis

Several genes in the set shared by all the GC-sensitive leukemic cells tested (see Table 2 and Additional file 2) have been demonstrated to be important for GC-dependent apoptosis. These genes include BCL2L11/Bim, DDIT4, DSCR1, TXNIP, NFKBIA, FKBP5, and TSC22D3/DSIPI/GILZ. In 1999, Bouillet et al. [25] observed that lymphocytes derived from Bim knockout mice were partially resistant to Dex-mediated apoptosis. Subsequently, we noted that Bim was upregulated by GC exclusively in two Dex-sensitive CEM clones [6]. Independently, this finding was confirmed in CEM cells and extended to mouse lymphoma lines [32]. Since then, Bim induction in response to GCs has been shown in chronic lymphocytic leukemia cells destined for apoptosis [33] and in thymocytes [34]. Recently, our finding that GCs activate p38 MAPK in both human and mouse lymphoid cells [35] has been extended to show an interaction between GC-mediated p38 MAPK stimulation and the expression of Bim, linking these two pro-apoptotic pathways in context to the GC signal transduction system as well as enzymatic protein activity and transcription [36]. We have demonstrated by qRT-PCR and confirm here by microarray that CVZ, which causes apoptosis in CEM-C1–15 cells, induces Bim in them, whereas Dex, to which they are resistant, does not [7]. It has also been shown that Bim regulation converges with the PKA pathway [37] consistent with our findings of Bim induction in the CEM-C1–15 cells restored to Dex-sensitive by FSK activation of that pathway. The rise in Bim, which occurs late in the lag phase, may be one of the final precipitating factors initiating apoptosis.

Our data indicated that DSCR1, like Bim, was induced in all GC-sensitive conditions in both human and mouse indicating its importance in the apoptotic program regulated by GCs. DSCR1 antagonizes the biological effects of the phosphatase calcineurin by interacting with catalytic subunit A and disrupting its downstream signaling pathways [38]. Data from glioblastoma cells suggests that in some cell types at least, DSCR1 may also stabilize IkappaB alpha, by an action independent of calcineurin [39].

TXNIP and DDIT4 regulate the redox state within the cell [40,41]. This effect was tested and the induction of TXNIP with the subsequent reduction in thioredoxin activity in response to Dex was confirmed, in WEHI7.2 cells [28]. Our comparative study indicates TXNIP to be regulated in all cells and conditions tested, but induction by Dex in the Dex-resistant cell line was very weak (Table 3).

NFKBIA antagonizes the action of transcription factor NFκB. We found NFKBIA to be induced by GCs in all systems tested, but like TXNIP, only weakly by Dex in resistant C1–15 cells. Inhibition of NFκB has been correlated with restoration of GC-sensitivity to resistant human monocytic/macrophage THP1 and U937 cell lines [30]. Several reports have suggested the use of the inductive response of the immunophilin FKBP5 (also known as FKBP51) as a diagnostic tool for GC response in peripheral blood mononuclear, T- and B-lymphocytes, and the highly Dex-resistant lymphoblastoid cell line IM-9 [42-44]. We call attention to the fact that Dex strongly induced FKBP5 in the resistant C1–15 cells. Thus FKBP5 induction alone is not always an indicator of apoptotic sensitivity to GC.

TSC22D3/DSIPI/GILZ was highly induced in all the leukemic cells tested, regardless of apoptotic sensitivity or resistance. Thus, as with FKBP5, GILZ induction alone cannot be relied upon as an indicator of apoptotic sensitivity to GCs.

The final gene sets that we have discovered are almost certainly incomplete, limited as they are by the numbers of genes represented on the smallest microarray chips employed. Thus our gene profile sets, while valid, may be expanded by additional studies with more complete arrays on larger chips. Nevertheless, the gene sets we have identified provide an initial tool that can be tested as prognostic indicators and almost certainly offer greater reliability than a single gene, e.g. FKBP5 or GILZ.

### Coincidence of regulated genes in varied leukemic cell types may provide the basis for prognostic screening

Therefore, we propose that our data offers the basis for developing a prognostic panel to screen relevant patients for the likelihood of an apoptotic response to GC. In at least some leukemias, response to GC is also predictive of response to overall chemotherapy [19,20]. Our work identifies the commonality of certain genes regulated by GCs in the apoptotically sensitive state of several leukemias, regardless of lineage. We present in Additional file 3 the list of 191, (83 induced, symbol plus, 108 repressed, symbol minus) genes regulated in common and specific to each list from the CEM signature (see Additional file 1) and the multiple cell line signature (see Additional file 2).

Though low cellular GR levels or GR mutations may account for a fraction of GR-resistant leukemias, the majority of GC-resistant cases are GR-positive [45-47]. In one study, involving xenografts derived from pediatric ALL patient biopsies, the failure of Bim to induce upon exposure to GC was associated with the resistant phenotype [48]. Combining this marker with the additional genes we have identified should strengthen the correlations.

The differences in GC-regulated genes found between the various types of leukemic cells examined may also be of importance. Examination of Additional files 4, 5, 6, 7, 8 and 9 shows that groups of genes are GC-controlled specifically in the adult B-cell ALL RS4;11, pediatric AML Kasumi-1, or pediatric B-cell ALL SUP-B15 lines. Since we were only able to examine one example of each of these types, it would be premature to conclude that the regulated genes found here as cell line-specific are representative of GC-apoptotic sensitivity in all leukemias of that class. We provide here in the additional files the comparisons that reveal the seemingly cell type-specific regulated genes, for the use of others and for the basis of wider-ranging experiments.

Shared GC-dependent gene regulation in mouse thymocytes and GC-sensitive leukemias speaks to a conserved pathway for GC-evoked apoptosis in lymphoid and other cells of hematologic origin. Since the "mouse chip" we employed only contains 6,000 probes, its breadth of sampling is limited compared to the human chips used. Even so, a sub-set of regulated genes common to the GC-sensitive human leukemic cell lines was also found to be regulated in the thymocytes. This indicates a remarkable underlying fundamental basis for the GC-dependent pre-apoptotic pathway considering the many differences between the systems: species, cells proliferating or not, oncogenetically transformed or not, lymphoid or myeloid. Finding several genes in the leukemic cells that are also regulated in the thymocyte system argues strongly for an essential basic network involved in GC-dependent apoptosis.

## Conclusion

In this study we have identified genes regulated by GCs using several apoptotic-sensitive cell lines and compared them to resistant, and resistant-restored-to-sensitive leukemic cells. We confirm and extend the brief meeting report [13] of the GC-sensitivity of SUP-B15 and RS4;11 cells. We have also found that there is a conserved group of genes regulated by GCs in common between cancer cell lines and primary mouse thymocytes. We present a preliminary genomic "signature" for apoptotic sensitivity to GCs in leukemias, in hope that it can be tested for screening clinical samples.

## Methods

### Reagents

Dex and all other chemicals except CVZ, FSK, and DEPC-treated water were purchased from Sigma-Aldrich (St. Louis, MO). CVZ was obtained through the kind offices of Dr. J. P. Raynaud, Roussel-UCLAF, Paris, France. FSK was from Calbiochem (La Jolla, CA) and DEPC-treated water was from Ambion (Austin, TX).

### Cell culture

The CCRF-CEM cell line was originally derived from a pediatric patient with ALL [49]. Highly Dex-sensitive CEM-C7–14 and Dex-resistant CEM-C1–15 cell clones were subcloned from the original C7 and C1 clones without selective pressure. CEM-C1–6, a Dex-sensitive spontaneous revertant was derived from the Dex-resistant C1 parent [50]. CEM cells were grown in RPMI 1640 (Cellgro Media Tech, Herndon, VA) pH 7.4 supplemented with 5% heat-inactivated fetal bovine serum (FBS) (Atlanta Biologicals, Norcross, GA). ALL cell lines RS4;11, SUP-B15, and the AML cell line Kasumi-1 were obtained from the American Type Culture Collection (ATCC, Manassas, VA). OPM-2 cells were originally derived from a patient with multiple myeloma [51]. RS4;11 and Kasumi-1 cells were grown in RPMI 1640 supplemented with 10 mM Hepes, 1 mM sodium pyruvate, and 10% FBS for RS4;11 or 20% FBS for Kasumi-1. SUP-B15 were cultured in Iscove's modified Dulbecco's medium containing 4 mM L-glutamine, 0.05 mM 2-mercaptoethanol, and 20% FBS. OPM-2 cells were grown in RPMI 1640 plus 10% defined FBS (Hyclone, Logan, UT). Mouse experiments were performed using sterile techniques under the approval and guidance of UTMB's Institutional Animal Care and Use Committee (IACUC) committee. Six 4-week old female C57 Black 6 mice (Harlan Sprague Dawley, Indianapolis, IN) were humanly euthanized using CO_2 _asphyxiation with subsequent surgical extraction of the whole thymus. The whole thymuses were deposited in a nylon mesh bag constructed by sealing the sides of LAB PAK mesh sheets (SEFAR America Inc., Kansas City, MO) and submerged in a small volume of 37°C RPMI 1640 + 5% FBS in a sterile 35 × 10 mm plastic petri dish. Thymocytes were separated by mechanical disruption of the thymus using sterile bent forceps and abrading the tissue from all six mice. All cells were cultured at 37°C in a humidified atmosphere of 95% air, 5% CO_2_. All cells except the thymocytes were grown in conditions ensuring logarithmic growth at the time of addition of the steroids or other compounds. Cell viability was determined by Trypan blue dye (Sigma-Aldrich) exclusion through use of a manual hemacytometer or by Vi-cell automated cell (Beckman Coulter, Miami, FL) counting. To determine GC-sensitivity, cells were diluted to 1 × 10^5 ^viable cells/ml in triplicate wells and exposed to vehicle control ethanol or DMSO (< 0.1% at final concentration) or various concentrations of Dex from 10^-9 ^M to 10^-6 ^M; thymocytes were exposed to 10^-7 ^M Dex only. Previous experiments had established the optimal concentrations of CVZ in CEM clones [7]. These experiments resulted in the following GC concentrations: CEM-C7–14 = 10^-6 ^M Dex, 10^-7 ^M CVZ; CEM-C1–6 = 10^-6 ^M Dex; CEM-C1–15 = 10^-6 ^M Dex, 10^-6 ^M CVZ; RS4;11 = 10^-6 ^M Dex; SUP-B15 = 10^-7 ^M Dex; Kasumi-1 = 10^-7 ^M Dex; OPM-2 = 10^-6 ^M Dex; and thymocytes = 10^-7 ^M Dex. We have previously noted the optimal conditions for resensitization of CEM-C1–15 cells to Dex through the use of FSK [12].

Preliminary experiments established the time of initial Dex-mediated phosphatidylserine membrane eversion, an early apoptotic feature for each cell type ([6] and data not shown). Treatment of CEM cells with CVZ or Dex plus FSK was taken at this same time-point. Experiments were performed in triplicate for both control and GC-treated samples, except for CEM-C1–15 cells treated with CVZ, for which three ethanolic and one DMSO 20 hour controls were averaged and compared to three CVZ-treated cultures and CEM-C1–15 samples treated with ethanolic/DMSO vehicle or Dex plus FSK which were done in duplicate. The gene expression profiles of ethanolic and DMSO-treated cells did not differ significantly/greatly between any of the samples. For microarray analysis of each cell type, RNA was extracted at the time of phosphatidylserine membrane eversion: CEM cells = 20 hours, RS4;11 = 24 hours, SUP-B15 = 24 hours, Kasumi-1 = 12 hours, and thymocytes = 1.5 hours.

### RNA extraction

At the appropriate time-point (Cell culture) cells were harvested by centrifugation 200 × g for 10 minutes and processed according to the instructions with the RNeasy extraction kit (Qiagen, Santa Clara, CA). The quality of each RNA preparation was validated independently by the UTMB Bioinformatics Core before its use.

### Target labeling and hybridization

Affymetrix microarray probe labeling and target hybridization was carried out as previously described [6]. All CEM cells were evaluated on HG_U95 Av2; RS4;11, SUP-B15, and Kasumi-1 on HGU133 Plus 2.0; and mouse thymocytes on U74Av2 Affymetrix GeneChip arrays. GeneChip arrays were analyzed in the UTMB Bioinformatics Core facility by Affymetrix GeneChip Suite software.

### Bioinformatic analysis

Affymetrix pivot data files for all chips were imported into Spotfire^® ^functional genomics software package version 8.1 (Somerville, MA). Genes whose transcript levels permitted a "present" call in ≥ 2 of 3 experiments were kept for further evaluation in all except CEM-C1–15 treated with Dex and FSK for which "present" was equal to 2 out of 2 experiments. A fold change cut-off was set at 1.2, a net 20% change, for each regulated gene. The mean transcript signal intensities corrected for baseline from triplicate (or in some cases 4) independent experimental samples were calculated, and control vs. treated p-values were determined except for CEM-C1–15 cells treated with Dex and FSK. A p-value of ≤ 0.05 was taken as statistically significant. Comparisons between arrays were accomplished through the use of Ingenuity^® ^(Redwood City, CA) pathway analysis tool bioinformatics software. Discrepancies between fold-changes of non-statistically significant and significantly regulated genes within the additional files are the result of multiple probes for certain genes.

### CEM signature

A list of genes regulated by GCs was generated by comparing transcripts regulated in common without regard to statistical significance among all tested GC-sensitive conditions in CEM cells: Dex-treated CEM-C7–14, CVZ-treated CEM-C7–14, Dex-treated CEM-C1–6, CVZ-treated CEM-C1–15, and Dex plus FSK treated CEM-C1–15 cells (Fig. 2A). This list was then compared to Dex-treated resistant CEM-C1–15 and genes failing our "present" call were designated with an asterisk. The list was further compared to Dex-treated mouse thymocytes and genes failing our "present" call were assigned an asterisk; genes oppositely regulated between species (e.g. up-regulated in one cell type and down-regulated in another) were assigned a closed circle. Each regulated gene was then analyzed for statistically significant regulation and marked with bold type in the table if the condition was met. An interconnected cell signaling network was generated using Ingenuity^® ^for the GC-regulated CEM signatory genes.

### Multiple leukemias signature

A list of regulated genes was generated by comparing transcripts regulated in common in the same sense (up or down) without regard to statistical significance among all naturally GC-sensitive leukemic cell lines: Dex-treated CEM-C7–14, CVZ-treated CEM-C7–14, Dex-treated CEM-C1–6, Dex-treated RS4;11, Dex-treated SUP-B15, and Dex-treated Kasumi-1 cells (Fig. 2B). Dex-treated resistant CEM-C1–15 and Dex-treated sensitive mouse thymocytes were then compared to the multiple leukemia signatory list and genes called "absent" designated with an asterisk or oppositely regulated between species designated with a closed circle. Genes that were regulated statistically significantly were designated with bold type in the table.

### Composite signature

A list of gene transcripts regulated in common by GCs in all sensitive and resistant- restored-to-sensitive conditions without regard to statistical significance was generated: Dex-treated CEM-C7–14, CVZ-treated CEM-C7–14, Dex-treated CEM-C1–6, Dex-treated RS4;11, Dex-treated SUP-B15, Dex-treated Kasumi-1, CVZ-treated CEM-C1–15, and Dex plus FSK treated CEM-C1–15 (Fig. 2C). This list was then compared to the Dex-resistant CEM-C1–15 and Dex-sensitive mouse thymocyte conditions. As before, "absent" calls were assigned an asterisk or oppositely regulated between species designated with a closed circle. Present genes regulated to a statistically significant extent were designated with bold type.

### Quantitative real-time PCR

CEM-C7–14 and OPM-2 cells were treated with 10^-6 ^M Dex and SUP-B15 cells with 10^-7 ^M Dex for 24 hours. RNA extraction and qRT-PCR was carried out as previously described [7]. Primers sequences for BCL2L11 (Bim) were forward: 5' – GTTCTGAGTGTGACCGAGAAGGTA and reverse: 3' – GTGGCTCTGTCTGTAGGGAGGTA. Primer sequences for ODC1 were forward: 5' – GACTTTTGATAGTGAAGTTGAGTTGATGA and reverse: 3' – GGCACCGAATTTCACACTGA.

## Competing interests

The author(s) declare that they have no competing interests.

## Authors' contributions

EBT and SK developed the concept. ALM, SK, MSW, EHL, and EBT were involved in experimental design. ALM, SK, and MSW processed the microarray data. ALM conducted the cell-based experiments and made the figures/tables. SK and ALM wrote the initial drafts of the manuscript. EBT supervised the research, wrote sections, edited the manuscript, and served as the principal investigator. All authors read and approved the final manuscript.

## Supplementary Material

Additional file 1 Gene expression signature for GC-sensitivity in CEM cells. Regulated transcripts from vehicle or GC-treated samples were evaluated and compared using Spotfire^® ^and Ingenuity^® ^bioinformatics software. Selection criteria for each gene were as follows: probe called present (Methods) on the microarray and regulated ≥ 1.2-fold (net 20% change). GC-mediated transcripts regulated in common in the same direction among sensitive CEM-C7–14 Dex (C7–14 Dx) and CVZ (C7–14 Z), CEM-C1–6 (C1–6 Dx), CEM-C1–15 CVZ (C1–15 Z), and CEM-C1–15 Dex plus FSK (C1–15 Dx+F) cells were obtained. The resulting 96 regulated genes are depicted. This list was subsequently compared to Dex-treated resistant CEM-C1–15 cells (C1–15 Dx) and to mouse thymocytes (C57/BL6 Dx). Genes linked via an Ingenuity^® ^generated signaling pathway (Network) are designated with (symbol, closed diamond). Opposite sense regulation (Opp. R) between human and mouse is indicated by (symbol, closed circle). Bold type indicates statistically significant regulation p ≤ 0.05 between means of vehicle vs. GC-treated. Symbol, asterisk = gene "absent" by selection criteria. Click here for file

Additional file 2 Gene expression signature for GC-sensitivity in multiple sub-types of sensitive human leukemias. Messenger RNA was extracted and regulated genes were analyzed as previously stated. A list of GC-mediated transcripts regulated in common in the same sense among sensitive CEM-C7–14 Dex (C7–14 Dx) and CVZ (C7–14 Z), CEM-C1–6 (C1–6 Dx), RS4;11 (RS4 Dx), SUP-B15 (SUP Dx), and Kasumi-1 (Kas Dx) leukemic cells was generated. These comparisons resulted in 122 regulated genes. This list was subsequently compared to Dex-treated resistant CEM-C1–15 cells (C1–15 Dx) and to mouse thymocytes (C57/BL6 Dx). Opposite sense regulation (Opp. R) between human and mouse is indicated by (symbol, closed circle). Bold type indicates statistically significant regulation p ≤ 0.05 between means of vehicle vs. GC-treated. Symbol, asterisk = gene "absent" by selection criteria. Click here for file

Additional file 3 Genes regulated by GCs in sensitive and Dex-resistant-restored-to-sensitive human leukemias. To obtain the final list of genes regulated by GCs in all sensitive model systems, the results from Additional file 1 and Additional file 2 were combined. Depicted are 191 genes, (83 induced, symbol plus) and (108 repressed, symbol minus) genes specific to and regulated in common between the CEM and other leukemia signatures. Click here for file

Additional file 4 Genes present on both HG_U95 Av2 and HGU133 Plus 2.0 chips. Genes regulated by GCs using selection criteria (Methods) in CEM-C7–14, CEM-C1–6, RS4;11, SUP-B15, and Kasumi-1 cells. Bold type indicates statistically significant regulation p ≤ 0.05 between means of vehicle vs. GC-treated. Blank = gene "absent" by selection criteria. Click here for file

Additional file 5 Adult ALL vs. Pediatric ALL and AML. Genes statistically significantly regulated by Dex in adult B-cell ALL RS4;11 cells compared to other GC-sensitive pediatric leukemias. Blank = gene "absent" by selection criteria. Click here for file

Additional file 6 Myeloid AML vs. Lymphoid ALL. Genes statistically significantly regulated by Dex in Kasumi-1 AML cells compared to GC-sensitive lymphoid leukemic cells. Blank = gene "absent" by selection criteria. Click here for file

Additional file 7 T-cell ALL vs. B-cell ALL. Genes regulated in common in the same sense by Dex in GC-sensitive T-cell ALL CEM-C7–14, CEM-C1–6 and GC-sensitive B-cell ALL RS4;11, SUP-B15 cells. Bold type indicates statistically significant regulation p ≤ 0.05 between means of vehicle vs. GC-treated. Click here for file

Additional file 8 Pediatric T-cell ALL vs. Pediatric B-cell ALL. Genes regulated in common in the same sense by Dex in T-cell ALL CEM-C7–14, CEM-C1–6, and B-cell SUP-B15 cells. Bold type indicates statistically significant regulation p ≤ 0.05 between means of vehicle vs. GC-treated. Click here for file

Additional file 9 Pediatric B-cell ALL vs. Adult B-cell ALL. Genes statistically significantly regulated by Dex in pediatric SUP-B15 cells compared to adult RS4;11 cells. Blank = gene "absent" by selection criteria. Click here for file
