# Supplementary material for: Gene expression profiling of leukemic cells and primary thymocytes predicts a signature for apoptotic sensitivity to glucocorticoids
Source: Cancer Cell Int. 2007 Nov 28;7:18. doi: 10.1186/1475-2867-7-18 (PMC2228275; doi:10.1186/1475-2867-7-18)
Supplement: Additional file 1 — Gene expression signature for GC-sensitivity in CEM cells. Regulated transcripts from vehicle or GC-treated samples were evaluated and compared using Spotfire® and Ingenuity® bioinformatics software. Selection criteria for each gene were as follows: probe called present (Methods) on the microarray and regulated ≥ 1.2-fold (net 20% change). GC-mediated transcripts regulated in common in the same direction among sensitive CEM-C7–14 Dex (C7–14 Dx) and CVZ (C7–14 Z), CEM-C1–6 (C1–6 Dx), CEM-C1–15 CVZ (C1–15 Z), and CEM-C1–15 Dex plus FSK (C1–15 Dx+F) cells were obtained. The resulting 96 regulated genes are depicted. This list was subsequently compared to Dex-treated resistant CEM-C1–15 cells (C1–15 Dx) and to mouse thymocytes (C57/BL6 Dx). Genes linked via an Ingenuity® generated signaling pathway (Network) are designated with (symbol, closed diamond). Opposite sense regulation (Opp. R) between human and mouse is indicated by (symbol, closed circle). Bold type indicates statistically significant regulation p ≤ 0.05 between means of vehicle vs. GC-treated. Symbol, asterisk = gene "absent" by selection criteria. [file 1475-2867-7-18-S1.pdf]

Additional file 1: Gene expression signature for GC-sensitivity in CEM cells.										
Page 1										
GC-response				Sensitive	Sensitive	Sensitive	Sensitive	Sensitive	Resensitized	Resistant
Type of lymphoid cell				Thymocyte	ALL	ALL	ALL	ALL	ALL	ALL
Name	Description	Network	Opp. R	C57/BL6 Dx	C7-14 Dx	C7-14 Z	C1-6 Dx	C1-15 Z	C1-15 Dx+F	C1-15 Dx
ABCE1	ATP-binding cassette, sub-family E (OABP), member 1		•	**1.3**	**-1.5**	**-2.0**	-1.7	-1.3	-1.2	*
AK2	adenylate kinase 2	♦		*	-1.4	**-1.7**	**-1.6**	**-1.6**	-1.4	*
ARHGEF7	Rho guanine nucleotide exchange factor (GEF) 7	♦	•	**-1.3**	**1.3**	1.4	1.3	**1.3**	1.8	1.2
BCL2L11	BCL2-like 11 (apoptosis facilitator)	♦		**1.8**	**3.1**	**14.5**	**5.9**	1.3	1.8	*
BTG1	B-cell translocation gene 1, anti-proliferative			*	**9.3**	**9.1**	**4.4**	**3.3**	1.7	1.9
BTG2	BTG family, member 2		•	-1.2	**3.1**	**2.9**	**2.3**	1.3	1.3	**1.4**
CALR	calreticulin	♦		*	**-1.9**	**-1.7**	-1.6	-1.4	-1.6	-1.3
CCNG2	cyclin G2		•	**-1.3**	**2.3**	**1.6**	1.3	1.3	1.4	1.3
CD53	CD53 antigen			*	**3.1**	**3.4**	**3.2**	**1.5**	1.8	**1.4**
CDC6	CDC6 cell division cycle 6 homolog (S. cerevisiae)	♦		**-1.2**	**-1.3**	-1.2	**-1.5**	**-1.3**	-1.3	-1.3
CHP	calcium binding protein P22		•	-1.7	1.2	1.2	1.3	1.5	1.2	*
CXCR4	chemokine (C-X-C motif) receptor 4			**1.3**	**1.3**	**1.3**	**1.5**	**1.5**	3.1	1.3
DDIT4	DNA-damage-inducible transcript 4	♦		**2.9**	**4.4**	**3.4**	2.5	1.2	4.4	1.5
DHODH	dihydroorotate dehydrogenase		•	1.3	-1.9	**-2.4**	-2.0	**-1.5**	-1.2	-1.3
DKC1	dyskeratosis congenita 1, dyskerin			*	**-1.7**	**-2.3**	**-1.3**	-1.4	-1.2	*
DSCR1	Down syndrome critical region gene 1			1.3	**5.0**	**8.2**	**4.8**	**1.4**	1.6	*
EIF3S9	eukaryotic translation initiation factor 3, subunit 9 eta, 116kDa			*	**-1.5**	**-2.2**	-1.3	-1.2	-1.2	*
ENO2	enolase 2 (gamma, neuronal)			*	-1.2	-1.4	-1.5	**-2.0**	-1.5	-1.2
FADS1	fatty acid desaturase 1			*	**-1.5**	**-4.1**	**-1.9**	-1.9	-1.3	-1.3
FBN1	fibrillin 1			*	1.6	1.2	**1.2**	1.2	1.4	*
FGFR1	fibroblast growth factor receptor 1	♦		*	**1.6**	**3.1**	1.4	**1.7**	1.7	1.3
FKBP4	FK506 binding protein 4, 59kDa	♦	•	**1.3**	-1.9	**-2.1**	-1.3	**-1.5**	-1.6	*
FKBP5	FK506 binding protein 5	♦		**1.7**	**6.6**	**11.2**	**4.4**	**3.8**	2.9	**2.8**
FOXK2	forkhead box K2			*	-1.3	-1.4	**-1.4**	-1.2	-1.3	*
GLB1	galactosidase, beta 1			*	**1.4**	**2.3**	**1.9**	1.5	1.4	1.3
GLRX	glutaredoxin (thioltransferase)			*	**3.4**	**5.2**	2.1	1.4	1.2	1.4
GPR125	G protein-coupled receptor 125			*	**-1.8**	-1.5	-1.6	**-1.4**	-1.3	*
GRAP2	GRB2-related adaptor protein 2			*	**2.4**	**2.9**	**3.6**	**2.8**	2.1	**2.7**
GSK3B	glycogen synthase kinase 3 beta			*	1.6	**1.9**	1.2	**1.8**	1.2	-1.2
GTPBP6	GTP binding protein 6 (putative)			*	-1.4	**-1.8**	-1.3	-1.2	-1.4	*
HDAC1	histone deacetylase 1	♦		**-1.6**	**-1.4**	-1.2	-1.2	**-1.3**	-1.2	-1.3
HRAS	v-Ha-ras Harvey rat sarcoma viral oncogene homolog	♦		*	**-1.3**	-1.4	-1.3	**-1.4**	-1.4	*
HSPE1	heat shock 10kDa protein 1 (chaperonin 10)		•	**1.3**	**-1.8**	**-2.1**	**-2.1**	**-1.5**	-1.3	*

**Additional file 1: Gene expression signature for GC-sensitivity in CEM cells.**										
**Page 2**										
**GC-response**				**Sensitive**	**Sensitive**	**Sensitive**	**Sensitive**	**Sensitive**	**Resensitized**	**Resistant**
**Type of lymphoid cell**				**Thymocyte**	**ALL**	**ALL**	**ALL**	**ALL**	**ALL**	**ALL**
**Name**	**Description**	**Network**	**Opp. R**	**C57/BL6 Dx**	**C7-14 Dx**	**C7-14 Z**	**C1-6 Dx**	**C1-15 Z**	**C1-15 Dx+F**	**C1-15 Dx**
ID1	inhibitor of DNA binding 1, dominant negative helix-loop-helix protein	♦		*	-2.6	**-1.7**	-1.2	**-1.9**	-1.6	**-1.4**
IDH3A	isocitrate dehydrogenase 3 (NAD+) alpha			*	**-1.5**	**-1.3**	**-1.5**	**-1.2**	-1.3	*
IDS	iduronate 2-sulfatase (Hunter syndrome)			*	1.4	**1.9**	1.2	**1.5**	1.4	1.3
IFRD1	interferon-related developmental regulator 1	♦		-1.3	**-1.8**	-1.2	-1.6	**-1.6**	-1.3	*
IL32	interleukin 32			*	-1.5	**-1.9**	**-1.9**	-1.4	-1.6	*
IL10RB	interleukin 10 receptor, beta			*	**1.6**	**1.9**	1.4	**1.6**	1.8	-1.2
IL7R	interleukin 7 receptor	♦		**1.4**	**8.3**	**18.0**	**6.3**	1.4	2.5	*
ILF3	interleukin enhancer binding factor 3, 90kDa			*	**-1.3**	-1.2	-1.3	**-1.2**	-1.4	*
IMPDH1	IMP (inosine monophosphate) dehydrogenase 1			*	**-1.6**	**-1.9**	**-1.4**	-1.3	-1.4	*
IQGAP2	IQ motif containing GTPase activating protein 2			*	**1.4**	**1.9**	**1.9**	**1.8**	1.9	1.3
ITGA6	integrin, alpha 6	♦		*	**5.0**	**10.2**	**2.4**	**6.6**	2.8	**1.6**
KIAA0020	KIAA0020			*	**-1.7**	**-3.6**	**-2.2**	-1.2	-1.3	*
LRPPRC	leucine-rich PPR-motif containing			*	**-1.8**	**-1.4**	**-1.7**	-1.3	-1.3	-1.3
MAP4	microtubule-associated protein 4	♦		**-1.6**	**-1.3**	**-1.8**	-1.7	-1.3	-1.3	*
MARS	methionine-tRNA synthetase			*	-1.5	**-1.6**	**-1.5**	**-1.2**	-1.3	*
MEP50	WD repeat domain 77			*	**-1.7**	**-1.4**	-1.8	**-1.5**	-1.3	*
MLX	MAX-like protein X			*	**-1.3**	-1.2	-1.5	-1.2	-1.3	*
MPI	mannose phosphate isomerase			*	**-1.3**	**-1.7**	-1.4	-1.4	-1.2	*
MT1A	metallothionein 1A (functional)	♦		*	1.3	**1.3**	**2.2**	**1.4**	1.4	*
MT1B	metallothionein 1B (functional)			*	1.3	**1.5**	1.6	**1.2**	1.5	*
MT1X	metallothionein 1X			*	1.4	**2.1**	2.2	**1.3**	1.4	*
MTHFD1	methylenetetrahydrofolate dehydrogenase (NADP+ dependent) 1	♦		*	**-1.4**	**-1.9**	**-1.5**	-1.5	-1.2	*
MYC	v-myc myelocytomatosis viral oncogene homolog (avian)	♦		**-2.2**	**-3.8**	**-4.6**	**-3.6**	-1.4	-2.2	*
NFATC3	nuclear factor of activated T-cells, cytoplasmic, calcineurin-dependent 3			*	**-1.4**	**-1.3**	**-1.9**	-1.8	-1.6	*
NFIL3	nuclear factor, interleukin 3 regulated			**6.4**	**6.0**	**7.4**	**3.6**	**1.6**	2.1	*
NFKBIA	nuclear factor of kappa light polypeptide gene enhancer in B-cells inhibitor, alpha	♦		**1.5**	**3.0**	**3.4**	**2.8**	**1.9**	1.5	**1.3**
NME1	non-metastatic cells 1, protein (NM23A) expressed in	♦		*	**-1.5**	**-2.2**	**-1.6**	**-1.4**	-1.3	*
NR3C1	nuclear receptor subfamily 3, group C, member 1 (glucocorticoid receptor)	♦	•	-1.7	**4.2**	**6.6**	**2.1**	**1.7**	1.6	*
OGT	O-linked N-acetylglucosamine (GlcNAc) transferase	♦	•	-1.5	1.3	**1.7**	**2.0**	**1.6**	1.3	*
PA2G4	proliferation-associated 2G4, 38kDa		•	**1.2**	**-1.7**	**-1.9**	-1.7	-1.3	-1.2	*
PARD3	par-3 partitioning defective 3 homolog (C. elegans)			*	1.6	**1.7**	**1.4**	1.3	1.4	*
PIAS2	protein inhibitor of activated STAT, 2	♦	•	**-2.3**	1.2	1.4	1.5	**2.0**	1.3	*
PIK3R1	phosphoinositide-3-kinase, regulatory subunit 1 (p85 alpha)	♦		*	**2.5**	**2.4**	1.7	**2.0**	1.9	*

Additional file 1: Gene expression signature for GC-sensitivity in CEM cells.										
**Page 3**										
**GC-response**				Sensitive	Sensitive	Sensitive	Sensitive	Sensitive	Resensitized	Resistant
**Type of lymphoid cell**				Thymocyte	ALL	ALL	ALL	ALL	ALL	ALL
**Name**	**Description**	**Network**	**Opp. R**	**C57/BL6 Dx**	**C7-14 Dx**	**C7-14 Z**	**C1-6 Dx**	**C1-15 Z**	**C1-15 Dx+F**	**C1-15 Dx**
PPP2R5C	protein phosphatase 2, regulatory subunit B (B56), gamma isoform			*	1.5	**1.7**	**1.5**	**1.4**	1.2	1.2
PRG1	proteoglycan 1, secretory granule			**1.6**	**2.6**	**4.5**	**3.2**	1.2	1.9	**1.6**
PTPN2	protein tyrosine phosphatase, non-receptor type 2			*	**-1.3**	-1.3	**-2.1**	-1.2	-1.3	*
RABGGTB	Rab geranylgeranyltransferase, beta subunit			-1.2	-1.3	**-1.3**	**-1.7**	**-1.8**	-1.3	-1.2
RAG1	recombination activating gene 1			**-1.3**	**-3.6**	**-3.2**	**-4.2**	-1.3	-3.0	*
RAPGEF2	Rap guanine nucleotide exchange factor (GEF) 2			*	1.7	**1.7**	1.4	**1.3**	1.3	*
RBMS1	RNA binding motif, single stranded interacting protein 1	♦		*	1.4	2.0	1.7	**1.6**	1.5	**1.6**
RRS1	RRS1 ribosome biogenesis regulator homolog (S. cerevisiae)			*	-1.9	**-2.7**	-1.9	**-1.6**	-1.2	-1.3
SCARB1	scavenger receptor class B, member 1	♦		*	**-1.7**	**-2.2**	-1.2	**-1.5**	-1.2	**-1.3**
SMN1	survival of motor neuron 1, telomeric			*	**-1.6**	-1.4	-1.8	-1.4	-1.3	*
SMPD1	sphingomyelin phosphodiesterase 1, acid lysosomal (acid sphingomyelinase)			*	**2.3**	**2.8**	1.3	1.3	1.2	1.2
SRM	spermidine synthase	♦		*	**-1.8**	**-3.3**	**-1.7**	**-1.4**	-1.4	*
TAGLN2	transgelin 2	♦		*	**-1.4**	**-2.0**	-1.3	-1.2	-1.2	*
TARBP1	Tar (HIV-1) RNA binding protein 1			*	**-1.6**	**-1.6**	**-1.8**	**-1.6**	-1.3	*
TFPI	tissue factor pathway inhibitor (lipoprotein-associated coagulation inhibitor)			*	**3.1**	**5.4**	**1.9**	**2.0**	1.8	**1.7**
TMEM123	transmembrane protein 123			*	**1.3**	**1.3**	1.3	1.4	1.2	1.2
TRAF3IP2	TRAF3 interacting protein 2	♦		*	1.6	**2.1**	1.8	1.5	1.4	1.3
TRAM2	translocation associated membrane protein 2			*	**1.5**	1.5	**1.7**	**1.3**	1.4	*
TRIP13	thyroid hormone receptor interactor 13			*	**-1.5**	**-1.4**	**-1.6**	**-1.2**	-1.3	*
TSC22D3	TSC22 domain family, member 3	♦		*	**33.1**	**74.0**	**20.4**	**7.4**	14.3	**5.0**
TUBA1	tubulin, alpha 1			1.3	**10.4**	**8.9**	**3.0**	**1.5**	2.0	1.3
TXNIP	thioredoxin interacting protein	♦		**2.6**	**2.8**	**3.4**	**3.7**	1.4	2.0	1.2
UBE2M	ubiquitin-conjugating enzyme E2M (UBC12 homolog, yeast)			*	**-1.4**	**-1.3**	-1.3	-1.4	-1.3	-1.3
UBE2S	ubiquitin-conjugating enzyme E2S	♦	•	**1.5**	-1.6	**-1.6**	-1.3	**-1.6**	-1.3	*
VCL	vinculin	♦	•	-1.3	1.8	**1.6**	**1.9**	**1.3**	1.5	*
WFS1	Wolfram syndrome 1 (wolframin)			*	**2.6**	**2.2**	**2.8**	**1.5**	1.8	1.3
XPOT	exportin, tRNA (nuclear export receptor for tRNAs)			-1.3	-1.4	**-1.8**	-1.5	**-1.4**	-1.2	*
YAF2	YY1 associated factor 2	♦		*	**1.8**	**2.4**	**1.6**	**1.7**	1.3	1.6
ZFP36L2	zinc finger protein 36, C3H type-like 2			**3.3**	**3.0**	**2.5**	**2.5**	**3.3**	10.1	**1.9**
ZNF259	zinc finger protein 259			*	**-1.4**	**-1.4**	**-1.8**	-1.3	-1.2	*
