# Supplementary material for: Gene expression profiling of leukemic cells and primary thymocytes predicts a signature for apoptotic sensitivity to glucocorticoids
Source: Cancer Cell Int. 2007 Nov 28;7:18. doi: 10.1186/1475-2867-7-18 (PMC2228275; doi:10.1186/1475-2867-7-18)
Supplement: Additional file 2 — Gene expression signature for GC-sensitivity in multiple sub-types of sensitive human leukemias. Messenger RNA was extracted and regulated genes were analyzed as previously stated. A list of GC-mediated transcripts regulated in common in the same sense among sensitive CEM-C7–14 Dex (C7–14 Dx) and CVZ (C7–14 Z), CEM-C1–6 (C1–6 Dx), RS4;11 (RS4 Dx), SUP-B15 (SUP Dx), and Kasumi-1 (Kas Dx) leukemic cells was generated. These comparisons resulted in 122 regulated genes. This list was subsequently compared to Dex-treated resistant CEM-C1–15 cells (C1–15 Dx) and to mouse thymocytes (C57/BL6 Dx). Opposite sense regulation (Opp. R) between human and mouse is indicated by (symbol, closed circle). Bold type indicates statistically significant regulation p ≤ 0.05 between means of vehicle vs. GC-treated. Symbol, asterisk = gene "absent" by selection criteria. [file 1475-2867-7-18-S2.pdf]

**Additional file 2: Gene expression signature for GC-sensitivity among multiple leukemias.**										
**Page 1**										
**GC-response**			**Sensitive**	**Sensitive**	**Sensitive**	**Sensitive**	**Sensitive**	**Sensitive**	**Sensitive**	**Resistant**
**Mouse vs. Patient-derived cell line**			**Mouse**	**Pediatric**	**Pediatric**	**Pediatric**	**Adult**	**Pediatric**	**Pediatric**	**Pediatric**
**Cell lineage**			**T-cell**	**T-cell**	**T-cell**	**T-cell**	**B-cell**	**B-cell**	**Myeloid**	**T-cell**
**Sub-type of leukemia or primary thymocyte**			**Thymocyte**	**ALL**	**ALL**	**ALL**	**ALL**	**ALL**	**AML**	**ALL**
**Name**	**Description**	**Opp. R**	**C57/BL6 Dx**	**C7-14 Dx**	**C7-14 Z**	**C1-6 Dx**	**RS4 Dx**	**SUP Dx**	**Kas Dx**	**C1-15 Dx**
AARS	alanyl-tRNA synthetase		**-1.5**	**-1.3**	**-1.3**	-1.2	**-2.0**	**-1.9**	**-1.5**	*
ABI1	abl-interactor 1		*	1.3	**1.8**	1.3	**1.4**	**1.7**	**2.8**	**1.4**
ACLY	ATP citrate lyase		*	-1.3	**-1.8**	-1.2	**-1.4**	**-1.7**	**-1.3**	*
ADAM9	ADAM metallopeptidase domain 9 (meltrin gamma)		*	1.3	**1.8**	1.3	**2.4**	**1.6**	**2.1**	1.3
AK2	adenylate kinase 2		*	-1.4	**-1.7**	**-1.6**	**-1.4**	**-2.2**	**-3.3**	*
AKAP1	A kinase anchor protein 1		-1.2	**-1.6**	**-2.5**	-1.9	**-1.8**	**-2.4**	**-2.2**	*
AP3S1	adaptor-related protein complex 3, sigma 1 subunit		*	**1.6**	**2.0**	**1.9**	**1.4**	**2.4**	**2.1**	*
APG12L	ATG12 autophagy related 12 homolog (S. cerevisiae)	•	-1.4	**1.6**	**2.0**	**1.9**	**1.3**	**1.9**	**2.5**	*
APPBP1	amyloid beta precursor protein binding protein 1		*	-1.2	**-1.6**	-1.5	**-1.2**	**-1.3**	**-1.2**	*
ATF5	activating transcription factor 5		*	-1.5	**-1.9**	-1.3	**-1.6**	**-2.0**	**-1.4**	*
ATIC	5-aminoimidazole-4-carboxamide ribonucleotide formyltransferase/IMP cyclohydrolase		*	**-1.4**	**-1.9**	**-2.0**	**-2.1**	**-3.2**	**-1.3**	*
ATP6V0D1	ATPase, H+ transporting, lysosomal 38kDa, V0 subunit D1		*	**1.3**	1.3	1.3	**1.4**	1.2	1.7	*
BCL2L11	BCL2-like 11 (apoptosis facilitator)		**1.8**	**3.1**	**14.5**	**5.9**	**1.2**	**3.1**	**5.1**	*
BDH	3-hydroxybutyrate dehydrogenase, type 1		*	**-1.3**	-1.5	**-1.5**	**-1.3**	-1.7	**-1.6**	*
BIRC2	baculoviral IAP repeat-containing 2		*	**1.9**	**1.7**	1.5	**1.4**	**1.9**	**1.6**	*
BTG1	B-cell translocation gene 1, anti-proliferative		*	**9.3**	**9.1**	**4.4**	1.2	**3.9**	**8.8**	1.9
BYSL	bystin-like		*	**-2.2**	**-1.8**	**-2.3**	**-6.2**	**-8.4**	**-4.0**	*
CAPN7	calpain 7	•	-1.7	1.4	1.4	1.3	**1.4**	1.5	1.2	*
CCT5	chaperonin containing TCP1, subunit 5 (epsilon)	•	1.8	**-1.6**	**-1.8**	**-1.7**	-1.3	**-1.5**	**-1.5**	*
CD53	CD53 antigen		*	**3.1**	**3.4**	**3.2**	**1.4**	**3.1**	**7.2**	**1.4**
CD99	CD99 antigen		*	1.2	**1.5**	**1.8**	**1.7**	**3.4**	**2.6**	*
CDC6	CDC6 cell division cycle 6 homolog (S. cerevisiae)		**-1.2**	**-1.3**	-1.2	-1.5	**-1.7**	**-2.9**	**-4.7**	-1.3
CDC25A	cell division cycle 25A		*	**-1.4**	-1.2	-1.4	**-1.6**	**-1.4**	**-19.4**	*
CEBPZ	CCAAT/enhancer binding protein zeta		*	**-1.5**	**-1.4**	**-1.8**	**-1.7**	**-1.5**	**-2.5**	*
CENTB2	centaurin, beta 2		*	1.3	**1.8**	**1.3**	**1.5**	**1.5**	1.3	*
CHC1	regulator of chromosome condensation 1		*	**-1.7**	**-2.1**	**-1.6**	-1.2	**-1.8**	**-1.7**	*
CLK1	CDC-like kinase 1	•	-1.6	1.2	**1.7**	1.3	**1.5**	**1.7**	**1.6**	*
CSE1L	CSE1 chromosome segregation 1-like (yeast)		*	-1.3	**-1.5**	-1.4	**-1.6**	**-1.4**	**-2.5**	*
CTPS	CTP synthase	•	1.3	**-1.7**	-2.5	-1.8	**-1.5**	**-1.4**	**-2.4**	*
CUGBP2	CUG triplet repeat, RNA binding protein 2		1.2	**2.0**	**2.2**	**1.8**	**5.7**	**2.4**	**1.7**	*
DDIT4	DNA-damage-inducible transcript 4		**2.9**	**4.4**	**3.4**	2.5	**5.5**	**4.6**	**22.4**	1.5
DSCR1	Down syndrome critical region gene 1		1.3	**5.0**	**8.2**	**4.8**	**1.5**	**7.7**	**7.9**	*
EEF1E1	eukaryotic translation elongation factor 1 epsilon 1		*	**-1.7**	**-2.1**	-2.3	**-1.6**	**-3.3**	**-2.4**	-1.7
EIF2S1	eukaryotic translation initiation factor 2, subunit 1 alpha, 35kDa		*	**-1.4**	-1.2	**-1.4**	**-1.2**	**-1.6**	**-1.8**	*
EIF3S9	eukaryotic translation initiation factor 3, subunit 9 eta, 116kDa		*	**-1.5**	**-2.2**	-1.3	**-1.2**	**-2.0**	**-2.2**	*

Additional file 2: Gene expression signature for GC-sensitivity among multiple leukemias.										
Page 2										
GC-response			Sensitive	Sensitive	Sensitive	Sensitive	Sensitive	Sensitive	Sensitive	Resistant
Mouse vs. Patient-derived cell line			Mouse	Pediatric	Pediatric	Pediatric	Adult	Pediatric	Pediatric	Pediatric
Cell lineage			T-cell	T-cell	T-cell	T-cell	B-cell	B-cell	Myeloid	T-cell
Sub-type of leukemia or primary thymocyte			Thymocyte	ALL	ALL	ALL	ALL	ALL	AML	ALL
Name	Description	Opp. R	C57/BL6 Dx	C7-14 Dx	C7-14 Z	C1-6 Dx	RS4 Dx	SUP Dx	Kas Dx	C1-15 Dx
EXOSC2	exosome component 2		*	**-1.7**	**-1.7**	-1.2	-1.2	**-2.4**	**-2.6**	*
FADS1	fatty acid desaturase 1		*	-1.5	**-4.1**	**-1.9**	**-1.5**	**-1.5**	**-1.8**	-1.3
FH	fumarate hydratase		*	-1.2	**-1.4**	-1.2	**-1.4**	**-2.1**	**-2.6**	-1.2
FKBP5	FK506 binding protein 5		**1.7**	**6.6**	**11.2**	**4.4**	**7.0**	**21.4**	**13.7**	**2.8**
FNBP1L	formin binding protein 1-like		*	**1.9**	1.5	1.8	**1.4**	**3.0**	**3.5**	1.3
FOXO3A	forkhead box O3A		*	1.3	1.4	1.2	**1.7**	**2.7**	**2.3**	-1.5
GLUL	glutamate-ammonia ligase (glutamine synthetase)		1.2	**1.9**	**2.2**	3.4	**2.3**	**2.5**	**1.8**	*
GM2A	GM2 ganglioside activator		*	**1.3**	**2.1**	1.5	1.4	1.5	**1.6**	*
GMPS	guanine monphosphate synthetase		*	-1.3	**-1.3**	-1.4	**-1.4**	**-1.4**	**-2.1**	*
GSPT1	G1 to S phase transition 1		-1.2	**-1.5**	**-1.4**	**-1.8**	**-1.6**	**-1.2**	**-1.4**	*
GTF3A	general transcription factor IIIA	•	**1.5**	**-1.3**	**-1.6**	-1.5	**-1.7**	**-1.4**	**-2.0**	*
HBP1	HMG-box transcription factor 1		*	1.5	**1.8**	**1.6**	**1.6**	**1.4**	**1.5**	*
HNRPAB	heterogeneous nuclear ribonucleoprotein A/B		*	**-1.5**	**-1.8**	**-1.4**	**-1.4**	**-1.6**	**-1.8**	*
HRMT1L2	HMT1 hnRNP methyltransferase-like 2 (S. cerevisiae)	•	**1.6**	**-1.5**	**-2.2**	**-1.7**	**-1.2**	**-2.6**	**-2.6**	*
HS6ST1	heparan sulfate 6-O-sulfotransferase 1		*	1.3	**2.1**	**2.2**	**1.3**	**1.8**	**1.5**	*
IARS	isoleucine-tRNA synthetase		**-1.3**	**-1.6**	**-1.7**	**-1.7**	**-1.4**	**-2.2**	**-3.8**	*
IDH3A	isocitrate dehydrogenase 3 (NAD+) alpha		*	**-1.5**	**-1.3**	**-1.5**	**-1.6**	**-1.7**	**-2.4**	*
IFNGR1	interferon gamma receptor 1		*	**1.7**	**2.0**	1.2	**5.7**	**3.3**	**2.6**	*
INPP1	inositol polyphosphate-1-phosphatase		*	**3.5**	**4.5**	**17.6**	**1.4**	**2.7**	**13.6**	*
JAK1	Janus kinase 1 (a protein tyrosine kinase)		**1.4**	**2.2**	**4.8**	**2.6**	**1.9**	**1.8**	**3.4**	1.9
KIAA0133	KIAA0133		*	-1.5	**-1.4**	-1.3	**-1.5**	**-1.4**	**-1.6**	*
LRP8	low density lipoprotein receptor-related protein 8, apolipoprotein e receptor		*	**-2.2**	-3.6	**-2.8**	**-1.6**	**-2.1**	**-2.6**	-1.2
LSM7	LSM7 homolog, U6 small nuclear RNA associated (S. cerevisiae)		*	-1.2	**-1.4**	-1.4	**-1.5**	**-1.5**	**-1.4**	*
M11S1	GPI-anchored membrane protein 1		*	-1.2	-1.3	-1.3	**-2.0**	**-1.3**	**-1.5**	*
MAP2K1	mitogen-activated protein kinase kinase 1		*	**1.5**	**1.7**	**1.7**	**1.5**	**1.9**	**2.1**	1.4
MARS	methionine-tRNA synthetase		*	-1.5	**-1.6**	-1.5	**-1.5**	**-2.2**	**-2.2**	*
MEP50	WD repeat domain 77		*	**-1.7**	**-1.4**	-1.8	**-1.8**	**-1.5**	**-1.4**	*
MGC5508	transmembrane protein 109		*	**-1.4**	**-1.5**	**-1.6**	**-1.4**	**-1.7**	**-1.5**	*
MGC17330	HGFL gene		*	**7.3**	**6.8**	**5.5**	**34.8**	**7.3**	**12.3**	*
MSN	moesin		*	1.2	1.3	1.2	**1.4**	**1.2**	**1.7**	1.2
MT1H	metallothionein 1H		*	**1.3**	**1.5**	**2.1**	**1.4**	**3.0**	**1.3**	*
MT1X	metallothionein 1X		*	1.4	**2.1**	2.2	**1.6**	**1.8**	**1.7**	*
NCK1	NCK adaptor protein 1	•	**-1.6**	**1.4**	**2.3**	**2.3**	**1.7**	**1.5**	**1.5**	1.4
NCL	nucleolin		*	**-1.3**	**-1.5**	-1.2	**-1.6**	**-1.9**	**-1.2**	*
NDRG1	N-myc downstream regulated gene 1		*	**1.6**	**1.7**	1.4	**9.2**	**3.1**	**2.3**	*

**Additional file 2: Gene expression signature for GC-sensitivity among multiple leukemias.**										
**Page 3**										
**GC-response**			**Sensitive**	**Sensitive**	**Sensitive**	**Sensitive**	**Sensitive**	**Sensitive**	**Sensitive**	**Resistant**
**Mouse vs. Patient-derived cell line**			**Mouse**	**Pediatric**	**Pediatric**	**Pediatric**	**Adult**	**Pediatric**	**Pediatric**	**Pediatric**
**Cell lineage**			**T-cell**	**T-cell**	**T-cell**	**T-cell**	**B-cell**	**B-cell**	**Myeloid**	**T-cell**
**Sub-type of leukemia or primary thymocyte**			**Thymocyte**	**ALL**	**ALL**	**ALL**	**ALL**	**ALL**	**AML**	**ALL**
**Name**	**Description**	**Opp. R**	**C57/BL6 Dx**	**C7-14 Dx**	**C7-14 Z**	**C1-6 Dx**	**RS4 Dx**	**SUP Dx**	**Kas Dx**	**C1-15 Dx**
NFKBIA	nuclear factor of kappa light polypeptide gene enhancer in B-cells inhibitor, alpha		**1.5**	**3.0**	**3.4**	**2.8**	**1.9**	**2.5**	**5.5**	**1.3**
NOLA2	nucleolar protein family A, member 2 (H/ACA small nucleolar RNPs)		*	-1.3	**-1.9**	-1.6	**-1.5**	**-1.8**	**-2.0**	*
NUP62	nucleoporin 62kDa		*	**-1.4**	-1.4	-1.3	**-1.4**	-1.4	**-2.6**	*
NUP98	nucleoporin 98kDa		*	-1.2	-1.2	-1.3	**-1.7**	**-2.0**	**-1.4**	*
ODC1	ornithine decarboxylase 1		*	**-2.1**	**-2.7**	**-2.1**	**-1.2**	**-1.5**	**-6.6**	*
OGT	O-linked N-acetylglucosamine (GlcNAc) transferase	•	-1.5	1.3	**1.7**	**2.0**	**2.0**	**1.3**	**2.1**	*
PA2G4	proliferation-associated 2G4, 38kDa	•	**1.2**	**-1.7**	**-1.9**	-1.7	-1.3	**-2.5**	**-3.5**	*
PAI-RBP1	SERPINE1 mRNA binding protein 1		*	**-1.6**	-1.5	-1.6	**-1.5**	**-1.9**	**-2.2**	1.3
PARD3	par-3 partitioning defective 3 homolog (C. elegans)		*	1.6	**1.7**	**1.4**	**2.5**	1.5	**3.0**	*
PFAS	phosphoribosylformylglycinamidine synthase (FGAR amidotransferase)		*	-1.4	**-2.1**	-1.2	**-2.4**	**-2.7**	**-2.3**	*
PICALM	phosphatidylinositol binding clathrin assembly protein		*	**1.7**	**1.7**	**1.5**	**2.1**	**2.0**	**2.1**	*
POLE2	polymerase (DNA directed), epsilon 2 (p59 subunit)		*	-1.2	-1.3	-1.7	**-1.8**	**-2.1**	**-2.0**	*
POLR2D	polymerase (RNA) II (DNA directed) polypeptide D	•	1.3	-1.3	**-1.3**	**-1.8**	-1.2	-1.3	-2.0	*
POLR2I	polymerase (RNA) II (DNA directed) polypeptide I, 14.5kDa		*	-1.4	**-1.9**	-1.5	**-1.6**	**-1.8**	**-1.7**	*
PRG1	proteoglycan 1, secretory granule		**1.6**	2.6	**4.5**	**3.2**	**2.7**	**1.5**	**2.7**	**1.6**
PSEN1	presenilin 1 (Alzheimer disease 3)		*	**1.7**	**1.8**	**1.5**	**1.3**	**1.5**	**2.0**	*
RAD23A	RAD23 homolog A (S. cerevisiae)		*	**-1.4**	**-1.6**	-1.3	-1.2	**-1.4**	-1.4	*
RANBP1	RAN binding protein 1	•	1.4	-1.3	**-1.8**	-1.5	**-1.9**	**-1.6**	**-2.8**	*
RAPGEF2	Rap guanine nucleotide exchange factor (GEF) 2		*	1.7	**1.7**	1.4	**1.8**	**2.0**	**4.2**	*
RASA1	RAS p21 protein activator (GTPase activating protein) 1		*	**1.9**	**2.2**	**2.1**	**1.9**	**2.3**	**3.5**	*
RBMS1	RNA binding motif, single stranded interacting protein 1		*	1.4	2.0	1.7	**3.0**	**4.3**	**2.3**	**1.6**
RDH11	retinol dehydrogenase 11 (all-trans and 9-cis)		*	-1.3	**-1.6**	-1.4	**-1.3**	**-1.8**	**-1.6**	*
SAP30	sin3-associated polypeptide, 30kDa		**1.6**	**2.2**	**1.8**	1.7	**1.7**	**2.3**	**3.8**	*
SCARB1	scavenger receptor class B, member 1		*	**-1.7**	**-2.2**	-1.2	**-1.4**	**-4.9**	**-33.3**	**-1.3**
SFRS2	splicing factor, arginine/serine-rich 2		*	-1.2	**-1.2**	-1.2	**-1.3**	**-1.4**	**-1.5**	1.2
SIVA	CD27-binding (Siva) protein		*	-1.4	-1.3	-1.3	**-1.6**	**-1.7**	**-2.9**	*
SLA	Src-like-adaptor	•	-1.5	**2.8**	**3.1**	**3.1**	**2.1**	**4.4**	**10.8**	*
SLC7A1	solute carrier family 7 (cationic amino acid transporter, y+ system), member 1		*	-1.7	**-2.0**	**-2.1**	**-2.4**	**-2.7**	**-2.3**	1.3
SLC16A1	solute carrier family 16 (monocarboxylic acid transporters), member 1		**-1.5**	-1.3	-1.3	-1.5	**-1.2**	**-2.8**	**-3.9**	*
SLC29A1	solute carrier family 29 (nucleoside transporters), member 1		*	**-1.7**	**-2.1**	-1.5	-1.3	**-2.6**	**-2.0**	*
SMARCA4	SWI/SNF related, matrix associated, actin dependent regulator of chromatin, subfamily a, member 4		-1.3	-1.3	**-1.2**	-1.6	**-1.9**	**-1.6**	-1.4	*
SORD	sorbitol dehydrogenase		*	-1.4	**-1.8**	-1.5	**-1.3**	**-1.6**	**-2.9**	*
SRD5A1	steroid-5-alpha-reductase, alpha polypeptide 1		*	2.7	**4.6**	**3.0**	**2.0**	**1.2**	**4.9**	1.2
SSBP1	single-stranded DNA binding protein 1		*	-1.3	-1.2	-1.4	**-1.3**	**-1.8**	**-1.4**	*
STAT2	signal transducer and activator of transcription 2, 113kDa		*	1.4	**1.3**	1.4	1.3	**1.2**	**1.5**	*

Additional file 2: Gene expression signature for GC-sensitivity among multiple leukemias.											
Page 4											
GC-response			Sensitive	Sensitive	Sensitive	Sensitive	Sensitive	Sensitive	Sensitive	Resistant
Mouse vs. Patient-derived cell line			Mouse	Pediatric	Pediatric	Pediatric	Adult	Pediatric	Pediatric	Pediatric
Cell lineage			T-cell	T-cell	T-cell	T-cell	B-cell	B-cell	Myeloid	T-cell
Sub-type of leukemia or primary thymocyte			Thymocyte	ALL	ALL	ALL	ALL	ALL	AML	ALL
Name	Description	Opp. R	C57/BL6 Dx	C7-14 Dx	C7-14 Z	C1-6 Dx	RS4 Dx	SUP Dx	Kas Dx	C1-15 Dx
STIP1	stress-induced-phosphoprotein 1 (Hsp70/Hsp90-organizing protein)	•	**1.5**	**-1.8**	**-1.4**	**-1.6**	**-1.6**	**-1.4**	**-1.3**	*
SYNCRIP	synaptotagmin binding, cytoplasmic RNA interacting protein		-1.3	-1.3	**-1.5**	**-1.4**	**-1.7**	**-1.6**	**-1.9**	*
TCP1	t-complex 1		*	**-1.4**	**-1.5**	**-1.7**	**-1.3**	**-1.5**	**-1.6**	*
TFAM	transcription factor A, mitochondrial		*	-1.3	-1.5	**-1.8**	**-1.4**	**-1.8**	**-2.0**	*
TFPI	tissue factor pathway inhibitor (lipoprotein-associated coagulation inhibitor)		*	**3.1**	**5.4**	**1.9**	**4.9**	**9.6**	**24.6**	**1.7**
TOMM40	translocase of outer mitochondrial membrane 40 homolog (yeast)		*	-1.5	**-2.0**	-1.3	-1.2	**-1.4**	**-1.6**	*
TRAP1	TNF receptor-associated protein 1		*	**-1.6**	**-1.8**	-1.6	**-1.3**	**-1.9**	**-1.8**	*
TSC22D3	TSC22 domain family, member 3		*	**33.1**	**74.0**	**20.4**	**5.2**	**17.5**	**20.0**	**5.0**
TSFM	Ts translation elongation factor, mitochondrial		*	**-1.5**	**-1.4**	**-1.6**	**-1.2**	**-2.5**	**-1.6**	*
TSNAX	translin-associated factor X		*	**2.1**	**2.6**	**2.2**	**1.2**	**1.9**	**2.0**	*
TXNIP	thioredoxin interacting protein		**2.6**	**2.8**	**3.4**	**3.7**	**10.0**	**7.7**	**2.1**	1.2
UBTF	upstream binding transcription factor, RNA polymerase I		*	**-1.3**	**-1.3**	-1.3	**-1.2**	**-1.3**	**-1.4**	*
VARS2	valyl-tRNA synthetase		*	-1.5	-1.3	-1.3	**-1.3**	**-2.1**	**-2.1**	*
YAF2	YY1 associated factor 2		*	**1.8**	**2.4**	**1.6**	**1.7**	**1.9**	**5.8**	1.6
YARS	tyrosyl-tRNA synthetase		*	-1.6	**-1.5**	-1.3	**-1.9**	**-2.6**	**-2.4**	*
ZHX3	zinc fingers and homeoboxes 3		*	1.3	**1.4**	1.3	**2.0**	**2.0**	1.4	1.3
ZNF259	zinc finger protein 259		*	**-1.4**	-1.5	**-1.8**	**-1.2**	-1.3	**-1.6**	*
