# Supplementary material for: Gene expression profiling of leukemic cells and primary thymocytes predicts a signature for apoptotic sensitivity to glucocorticoids
Source: Cancer Cell Int. 2007 Nov 28;7:18. doi: 10.1186/1475-2867-7-18 (PMC2228275; doi:10.1186/1475-2867-7-18)
Supplement: Additional file 3 — Genes regulated by GCs in sensitive and Dex-resistant-restored-to-sensitive human leukemias. To obtain the final list of genes regulated by GCs in all sensitive model systems, the results from Additional file 1 and Additional file 2 were combined. Depicted are 191 genes, (83 induced, symbol plus) and (108 repressed, symbol minus) genes specific to and regulated in common between the CEM and other leukemia signatures. [file 1475-2867-7-18-S3.pdf]

**Additional file 3: GC-regulated genes in sensitive and resensitized human leukemias.**			
**(83 Induced, 108 Repressed = 191 Total Genes)**			
**Page 1**			
**Induced Genes**		**Add. file 1**	**Add. file 2**
ABI1	abl-interactor 1		+
ADAM9	ADAM metallopeptidase domain 9 (meltrin gamma)		+
AP3S1	adaptor-related protein complex 3, sigma 1 subunit		+
APG12L	ATG12 autophagy related 12 homolog (S. cerevisiae)		+
ARHGEF7	Rho guanine nucleotide exchange factor (GEF) 7	+	
ATP6V0D1	ATPase, H+ transporting, lysosomal 38kDa, V0 subunit D1		+
BCL2L11	BCL2-like 11 (apoptosis facilitator)	+	+
BIRC2	baculoviral IAP repeat-containing 2		+
BTG1	B-cell translocation gene 1, anti-proliferative	+	+
BTG2	BTG family, member 2	+	+
CAPN7	calpain 7		+
CCNG2	cyclin G2	+	
CD53	CD53 antigen	+	+
CD99	CD99 antigen		+
CENTB2	centaurin, beta 2		+
CHP	calcium binding protein P22	+	
CLK1	CDC-like kinase 1		+
CUGBP2	CUG triplet repeat, RNA binding protein 2		+
CXCR4	chemokine (C-X-C motif) receptor 4	+	
DDIT4	DNA-damage-inducible transcript 4	+	+
DSCR1	Down syndrome critical region gene 1	+	+
FBN1	fibrillin 1	+	
FGFR1	fibroblast growth factor receptor 1	+	
FKBP5	FK506 binding protein 5	+	+
FNBP1L	formin binding protein 1-like		+
FOXO3A	forkhead box O3A		+
GLB1	galactosidase, beta 1	+	
GLRX	glutaredoxin (thioltransferase)	+	
GLUL	glutamate-ammonia ligase (glutamine synthetase)		+
GM2A	GM2 ganglioside activator		+
GRAP2	GRB2-related adaptor protein 2	+	
GSK3B	glycogen synthase kinase 3 beta	+	
HBP1	HMG-box transcription factor 1		+
HS6ST1	heparan sulfate 6-O-sulfotransferase 1		+
IDS	iduronate 2-sulfatase (Hunter syndrome)	+	
IFNGR1	interferon gamma receptor 1		+
IL7R	interleukin 7 receptor	+	
IL10RB	interleukin 10 receptor, beta	+	
INPP1	inositol polyphosphate-1-phosphatase		+
IQGAP2	IQ motif containing GTPase activating protein 2	+	
ITGA6	integrin, alpha 6	+	
JAK1	Janus kinase 1 (a protein tyrosine kinase)		+
MAP2K1	mitogen-activated protein kinase kinase 1		+
MGC17330	HGFL gene		+
MSN	moesin		+
MT1A	metallothionein 1A (functional)	+	
MT1B	metallothionein 1B (functional)	+	
MT1H	metallothionein 1H		+

Additional file 3: GC-regulated genes in sensitive and resensitized human leukemias.			
(83 Induced, 108 Repressed = 191 Total Genes)			
Page 2			
**Induced Genes**		**Add. file 1**	**Add. file 2**
MT1X	metallothionein 1X	+	+
NCK1	NCK adaptor protein 1		+
NDRG1	N-myc downstream regulated gene 1		+
NFIL3	nuclear factor, interleukin 3 regulated	+	
NFKBIA	nuclear factor of kappa light polypeptide gene enhancer in B-cells inhibitor, alpha	+	+
NR3C1	nuclear receptor subfamily 3, group C, member 1 (glucocorticoid receptor)	+	
OGT	O-linked N-acetylglucosamine (GlcNAc) transferase	+	+
PARD3	par-3 partitioning defective 3 homolog (C. elegans)	+	+
PIAS2	protein inhibitor of activated STAT, 2	+	
PICALM	phosphatidylinositol binding clathrin assembly protein		+
PIK3R1	phosphoinositide-3-kinase, regulatory subunit 1 (p85 alpha)	+	
PPP2R5C	protein phosphatase 2, regulatory subunit B (B56), gamma isoform	+	
PRG1	proteoglycan 1, secretory granule	+	+
PSEN1	presenilin 1 (Alzheimer disease 3)		+
RAPGEF2	Rap guanine nucleotide exchange factor (GEF) 2	+	+
RASA1	RAS p21 protein activator (GTPase activating protein) 1		+
RBMS1	RNA binding motif, single stranded interacting protein 1	+	+
SAP30	sin3-associated polypeptide, 30kDa		+
SLA	Src-like-adaptor		+
SMPD1	sphingomyelin phosphodiesterase 1, acid lysosomal (acid sphingomyelinase)	+	
SRD5A1	steroid-5-alpha-reductase, alpha polypeptide 1		+
STAT2	signal transducer and activator of transcription 2, 113kDa		+
TFPI	tissue factor pathway inhibitor (lipoprotein-associated coagulation inhibitor)	+	+
TMEM123	transmembrane protein 123	+	
TRAF3IP2	TRAF3 interacting protein 2	+	
TRAM2	translocation associated membrane protein 2	+	
TSC22D3	TSC22 domain family, member 3	+	+
TSNAX	translin-associated factor X		+
TUBA1	tubulin, alpha 1	+	
TXNIP	thioredoxin interacting protein	+	+
VCL	vinculin	+	
WFS1	Wolfram syndrome 1 (wolframin)	+	
YAF2	YY1 associated factor 2	+	+
ZFP36L2	zinc finger protein 36, C3H type-like 2	+	
ZHX3	zinc fingers and homeoboxes 3		+
**Repressed Genes**		**Add. file 1**	**Add. file 2**
AARS	alanyl-tRNA synthetase		-
ABCE1	ATP-binding cassette, sub-family E (OABP), member 1	-	
ACLY	ATP citrate lyase		-
AK2	adenylate kinase 2	-	-
AKAP1	A kinase anchor protein 1		-
APPBP1	amyloid beta precursor protein binding protein 1		-
ATF5	activating transcription factor 5		-
ATIC	5-aminoimidazole-4-carboxamide ribonucleotide formyltransferase/IMP cyclohydrolase		-
BDH	3-hydroxybutyrate dehydrogenase, type 1		-
BYSL	bystin-like		-
CALR	calreticulin	-	
CCT5	chaperonin containing TCP1, subunit 5 (epsilon)		-

**Additional file 3: GC-regulated genes in sensitive and resensitized human leukemias.**			
**(83 Induced, 108 Repressed = 191 Total Genes)**			
**Page 3**			
**Repressed Genes**		**Add. file 1**	**Add. file 2**
CDC25A	cell division cycle 25A		-
CDC6	CDC6 cell division cycle 6 homolog (S. cerevisiae)	-	-
CEBPZ	CCAAT/enhancer binding protein zeta		-
CHC1	regulator of chromosome condensation 1		-
CSE1L	CSE1 chromosome segregation 1-like (yeast)		-
CTPS	CTP synthase		-
DHODH	dihydroorotate dehydrogenase	-	
DKC1	dyskeratosis congenita 1, dyskerin	-	
EEF1E1	eukaryotic translation elongation factor 1 epsilon 1		-
EIF2S1	eukaryotic translation initiation factor 2, subunit 1 alpha, 35kDa		-
EIF3S9	eukaryotic translation initiation factor 3, subunit 9 eta, 116kDa	-	-
ENO2	enolase 2 (gamma, neuronal)	-	
EXOSC2	exosome component 2		-
FADS1	fatty acid desaturase 1	-	-
FH	fumarate hydratase		-
FKBP4	FK506 binding protein 4, 59kDa	-	
FOXK2	forkhead box K2	-	
GMPS	guanine monphosphate synthetase		-
GPR125	G protein-coupled receptor 125	-	
GSPT1	G1 to S phase transition 1		-
GTF3A	general transcription factor IIIA		-
GTPBP6	GTP binding protein 6 (putative)	-	
HDAC1	histone deacetylase 1	-	
HNRPAB	heterogeneous nuclear ribonucleoprotein A/B		-
HRAS	v-Ha-ras Harvey rat sarcoma viral oncogene homolog	-	
HRMT1L2	HMT1 hnRNP methyltransferase-like 2 (S. cerevisiae)		-
HSPE1	heat shock 10kDa protein 1 (chaperonin 10)	-	
IARS	isoleucine-tRNA synthetase		-
ID1	inhibitor of DNA binding 1, dominant negative helix-loop-helix protein	-	
IDH3A	isocitrate dehydrogenase 3 (NAD+) alpha	-	-
IFRD1	interferon-related developmental regulator 1	-	
IL32	interleukin 32	-	
ILF3	interleukin enhancer binding factor 3, 90kDa	-	
IMPDH1	IMP (inosine monophosphate) dehydrogenase 1	-	
KIAA0020	KIAA0020	-	
KIAA0133	KIAA0133		-
LRP8	low density lipoprotein receptor-related protein 8, apolipoprotein e receptor		-
LRPPRC	leucine-rich PPR-motif containing	-	
LSM7	LSM7 homolog, U6 small nuclear RNA associated (S. cerevisiae)		-
M11S1	GPI-anchored membrane protein 1		-
MAP4	microtubule-associated protein 4	-	
MARS	methionine-tRNA synthetase	-	-
MEP50	WD repeat domain 77	-	-
MGC5508	transmembrane protein 109		-
MLX	MAX-like protein X	-	
MPI	mannose phosphate isomerase	-	
MTHFD1	methylenetetrahydrofolate dehydrogenase (NADP+ dependent) 1	-	
MYC	v-myc myelocytomatosis viral oncogene homolog (avian)	-	

Additional file 3: GC-regulated genes in sensitive and resensitized human leukemias.			
(83 Induced, 108 Repressed = 191 Total Genes)			
Page 4			
Repressed Genes		Add. file 1	Add. file 2
NCL	nucleolin		-
NFATC3	nuclear factor of activated T-cells, cytoplasmic, calcineurin-dependent 3	-	
NME1	non-metastatic cells 1, protein (NM23A) expressed in	-	
NOLA2	nucleolar protein family A, member 2 (H/ACA small nucleolar RNPs)		-
NUP62	nucleoporin 62kDa		-
NUP98	nucleoporin 98kDa		-
ODC1	ornithine decarboxylase 1		-
PA2G4	proliferation-associated 2G4, 38kDa	-	-
PAI-RBP1	SERPINE1 mRNA binding protein 1		-
PFAS	phosphoribosylformylglycinamidine synthase (FGAR amidotransferase)		-
POLE2	polymerase (DNA directed), epsilon 2 (p59 subunit)		-
POLR2D	polymerase (RNA) II (DNA directed) polypeptide D		-
POLR2I	polymerase (RNA) II (DNA directed) polypeptide I, 14.5kDa		-
PTPN2	protein tyrosine phosphatase, non-receptor type 2	-	
RABGGTB	Rab geranylgeranyltransferase, beta subunit	-	
RAD23A	RAD23 homolog A (S. cerevisiae)		-
RAG1	recombination activating gene 1	-	
RANBP1	RAN binding protein 1		-
RDH11	retinol dehydrogenase 11 (all-trans and 9-cis)		-
RRS1	RRS1 ribosome biogenesis regulator homolog (S. cerevisiae)	-	
SCARB1	scavenger receptor class B, member 1	-	-
SFRS2	splicing factor, arginine/serine-rich 2		-
SIVA	CD27-binding (Siva) protein		-
SLC7A1	solute carrier family 7 (cationic amino acid transporter, y+ system), member 1		-
SLC16A1	solute carrier family 16 (monocarboxylic acid transporters), member 1		-
SLC29A1	solute carrier family 29 (nucleoside transporters), member 1		-
SMARCA4	SWI/SNF related, matrix associated, actin dependent regulator of chromatin, subfamily a, member 4		-
SMN1	survival of motor neuron 1, telomeric	-	
SORD	sorbitol dehydrogenase		-
SRM	spermidine synthase	-	
SSBP1	single-stranded DNA binding protein 1		-
STIP1	stress-induced-phosphoprotein 1 (Hsp70/Hsp90-organizing protein)		-
SYNCRIP	synaptotagmin binding, cytoplasmic RNA interacting protein		-
TAGLN2	transgelin 2	-	
TARBP1	Tar (HIV-1) RNA binding protein 1	-	
TCP1	t-complex 1		-
TFAM	transcription factor A, mitochondrial		-
TOMM40	translocase of outer mitochondrial membrane 40 homolog (yeast)		-
TRAP1	TNF receptor-associated protein 1		-
TRIP13	thyroid hormone receptor interactor 13	-	
TSFM	Ts translation elongation factor, mitochondrial		-
UBE2M	ubiquitin-conjugating enzyme E2M (UBC12 homolog, yeast)	-	
UBE2S	ubiquitin-conjugating enzyme E2S	-	
UBTF	upstream binding transcription factor, RNA polymerase I		-
VARS2	valyl-tRNA synthetase		-
XPOT	exportin, tRNA (nuclear export receptor for tRNAs)	-	
YARS	tyrosyl-tRNA synthetase		-
ZNF259	zinc finger protein 259	-	-
