# Supplementary material for: Gene expression profiling of leukemic cells and primary thymocytes predicts a signature for apoptotic sensitivity to glucocorticoids
Source: Cancer Cell Int. 2007 Nov 28;7:18. doi: 10.1186/1475-2867-7-18 (PMC2228275; doi:10.1186/1475-2867-7-18)
Supplement: Additional file 4 — Genes present on both HG_U95 Av2 and HGU133 Plus 2.0 chips. Genes regulated by GCs using selection criteria (Methods) in CEM-C7–14, CEM-C1–6, RS4;11, SUP-B15, and Kasumi-1 cells. Bold type indicates statistically significant regulation p ≤ 0.05 between means of vehicle vs. GC-treated. Blank = gene "absent" by selection criteria. [file 1475-2867-7-18-S4.pdf]

**Additional file 4: Genes present on both HG_U95 Av2 and HGU133 Plus 2.0 chips.**													
**Blank = "absent"**													
**GC-response**		**Sensitive**	**Sensitive**	**Sensitive**	**Sensitive**	**Sensitive**	**Sensitive**	**Sensitive**	**Sensitive**	**Sensitive**	**Sensitive**	**Sensitive**	**Sensitive**
**Patient-derived cell line**		**Pediatric**	**Pediatric**	**Pediatric**	**Pediatric**	**Pediatric**	**Pediatric**	**Adult**	**Adult**	**Pediatric**	**Pediatric**	**Pediatric**	**Pediatric**
**Cell lineage**		**T-cell**	**T-cell**	**T-cell**	**T-cell**	**T-cell**	**T-cell**	**B-cell**	**B-cell**	**B-cell**	**B-cell**	**Myeloid**	**Myeloid**
**Sub-type of leukemia**		**ALL**	**ALL**	**ALL**	**ALL**	**ALL**	**ALL**	**ALL**	**ALL**	**ALL**	**ALL**	**AML**	**AML**
**Name**	**Description**	**C7-14 Dx**	**C7-14 Dx**	**C7-14 Z**	**C7-14 Z**	**C1-6 Dx**	**C1-6 Dx**	**RS4 Dx**	**RS4 Dx**	**SUP Dx**	**SUP Dx**	**Kas Dx**	**Kas Dx**
			**Stat. sign**		**Stat. sign**		**Stat. sign**		**Stat. sign**		**Stat. sign**		**Stat. sign**
76P	gamma tubulin ring complex protein (76p gene)					1.3		-1.9	**-1.9**	-1.6	**-1.6**	1.6	**1.6**
AANAT	arylalkylamine N-acetyltransferase	1.4				1.5							
AARS	alanyl-tRNA synthetase	-1.3	**-1.3**	-1.3	**-1.3**	-1.2		-2.0	**-2.0**	-1.9	**-1.9**	-1.5	**-1.5**
AASDHPPT	aminoadipate-semialdehyde dehydrogenase-phosphopantetheinyl transferase	-1.4		-1.4	**-1.4**	-1.7		-1.4	**-1.4**	-1.3	**-1.3**		
AATF	apoptosis antagonizing transcription factor	-1.5		-1.5	**-1.5**	-1.4	**-1.4**	-1.3	**-1.3**				
ABAT	4-aminobutyrate aminotransferase							-21.9	**-21.9**	-1.6		14.5	**14.5**
ABCB10	ATP-binding cassette, sub-family B (MDR/TAP), member 10									1.3	**1.3**	-1.7	**-1.7**
ABCB6	ATP-binding cassette, sub-family B (MDR/TAP), member 6	1.3	**1.3**					-1.6	**-1.6**	1.2		3.4	**3.4**
ABCB7	ATP-binding cassette, sub-family B (MDR/TAP), member 7	1.2						1.3	**1.3**				
ABCB9	ATP-binding cassette, sub-family B (MDR/TAP), member 9	1.5		1.3									
ABCC1	ATP-binding cassette, sub-family C (CFTR/MRP), member 1			1.7	**1.7**	1.5	**1.5**	1.9	**1.9**	-2.0	**-2.0**	-3.3	**-3.3**
ABCC5	ATP-binding cassette, sub-family C (CFTR/MRP), member 5	2.1	**2.1**	2.0	**2.0**	1.8		-1.4	**-1.4**	-1.8	**-1.8**	1.3	
ABCD3	ATP-binding cassette, sub-family D (ALD), member 3			1.4		1.2		1.5				-1.8	**-1.8**
ABCE1	ATP-binding cassette, sub-family E (OABP), member 1	-1.5	**-1.5**	-2.0	**-2.0**	-1.7		1.4	**1.4**	-2.0	**-2.0**	-4.2	**-4.2**
ABCF1	ATP-binding cassette, sub-family F (GCN20), member 1									-1.5	**-1.5**	-2.0	**-2.0**
ABCF2	ATP-binding cassette, sub-family F (GCN20), member 2									-1.9	**-1.9**	-1.9	**-1.9**
ABCG1	ATP-binding cassette, sub-family G (WHITE), member 1					-2.0	**-2.0**						
ABI1	abl-interactor 1	1.3		1.8	**1.8**	1.3		1.4	**1.4**	1.7	**1.7**	2.8	**2.8**
ABI2	abl interactor 2			1.8	**1.8**	-1.5		1.3	**1.3**	-1.9	**-1.9**	-2.7	**-2.7**
ABL1	v-abl Abelson murine leukemia viral oncogene homolog 1			-1.2		1.5						-1.4	**-1.4**
ABLIM1	actin binding LIM protein 1							-5.8	**-5.8**	-3.1	**-3.1**		
ABR	active BCR-related gene											1.4	**1.4**
ACAA1	acetyl-Coenzyme A acyltransferase 1 (peroxisomal 3-oxoacyl-Coenzyme A thiolase)									-1.3	**-1.3**	1.2	**1.2**
ACACA	acetyl-Coenzyme A carboxylase alpha	-1.2										-6.5	**-6.5**
ACACB	acetyl-Coenzyme A carboxylase beta					1.8		9.5	**9.5**			1.7	**1.7**
ACADM	acyl-Coenzyme A dehydrogenase, C-4 to C-12 straight chain							-1.8	**-1.8**			1.7	**1.7**
ACADSB	acyl-Coenzyme A dehydrogenase, short/branched chain	1.2								-2.0	**-2.0**	1.4	
ACADVL	acyl-Coenzyme A dehydrogenase, very long chain	1.4								1.2			
ACAT2	acetyl-Coenzyme A acetyltransferase 2 (acetoacetyl Coenzyme A thiolase)			-2.0	**-2.0**			-2.4	**-2.4**	-1.5	**-1.5**	1.3	
ACBD3	acyl-Coenzyme A binding domain containing 3							-2.0	**-2.0**	1.6	**1.4**	1.5	**1.5**
ACD	adrenocortical dysplasia homolog (mouse)							-1.3	**-1.3**				
ACIN1	apoptotic chromatin condensation inducer 1											1.6	**1.6**
ACLY	ATP citrate lyase	-1.3		-1.8	**-1.8**	-1.2		-1.4	**-1.4**	-1.7	**-1.7**	-1.3	**-1.3**
ACO1	aconitase 1, soluble	1.3	**1.3**	2.0	**2.0**	1.4	**1.4**	1.4	**1.4**	-1.4		-1.2	**-1.2**
ACO2	aconitase 2, mitochondrial			-1.3		-1.5						-1.6	**-1.6**
ACOT2	acyl-CoA thioesterase 2							5.0	**5.0**			-7.8	**-7.8**
ACOT7	acyl-CoA thioesterase 7							-1.6	**-1.6**	-1.4	**-1.4**	-1.3	**-1.3**
ACOX1	acyl-Coenzyme A oxidase 1, palmitoyl	1.4						5.2	**5.2**	1.2		-3.5	**-3.5**
ACP1	acid phosphatase 1, soluble							-2.1	**-2.1**			-1.8	**-1.8**
ACP2	acid phosphatase 2, lysosomal	1.6	**1.6**	1.6	**1.6**								
ACR	acrosin			-1.4									
ACSL3	acyl-CoA synthetase long-chain family member 3	-1.3		-1.5	**-1.5**	-1.3		-1.8	**-1.8**	-1.3	**-1.3**	2.2	**2.2**
ACSL4	acyl-CoA synthetase long-chain family member 4	-1.4		1.4				-1.7	**-1.7**			1.4	
ACTA1	actin, alpha 1, skeletal muscle	1.3		1.7									
ACTB	actin, beta							-1.5	**-1.5**			1.6	**1.6**
ACTG1	actin, gamma 1	-1.2	**-1.2**										
ACTL6A	actin-like 6A					-1.4		-1.5	**-1.5**	-1.3	**-1.3**	-1.6	**-1.6**
ACTN1	actinin, alpha 1	1.4						5.4	**5.4**	-1.2	**-1.2**	-3.5	**-3.5**
ACTN4	actinin, alpha 4							-1.6		1.4		1.7	**1.7**

ACTR1A	ARP1 actin-related protein 1 homolog A, centractin alpha (yeast)	1.2						1.2					
ACTR2	ARP2 actin-related protein 2 homolog (yeast)	1.2	**1.2**	1.4	**1.4**	-1.2		-1.4	**-1.4**	1.3	**1.3**	1.6	**1.6**
ACTR3	ARP3 actin-related protein 3 homolog (yeast)							2.0	**2.0**	1.2		-1.6	**-1.6**
ACVR1B	activin A receptor, type IB			1.7		1.2		1.7	**1.7**	-1.6		-1.5	**-1.5**
ACY1	aminoacylase 1							1.7	**1.7**			-4.3	**-4.3**
ACYP1	acylphosphatase 1, erythrocyte (common) type			-1.3	**-1.3**	-1.3							
ACYP2	acylphosphatase 2, muscle type											1.4	**1.4**
ADA	adenosine deaminase	-1.3	**-1.3**	-1.3	**-1.3**	-1.5	**-1.5**	-9.1	**-9.1**	1.3	**1.3**	3.4	**3.4**
ADAM10	ADAM metallopeptidase domain 10			2.4	**2.4**			-3.0	**-3.0**	2.1	**2.1**	2.8	**2.8**
ADAM15	ADAM metallopeptidase domain 15 (metargidin)												
ADAM17	ADAM metallopeptidase domain 17 (tumor necrosis factor, alpha, converting enzyme)	1.2		6.9	**2.0**			-1.7	**-1.7**	1.5	**1.5**	1.8	**1.8**
ADAM9	ADAM metallopeptidase domain 9 (meltrin gamma)	1.3		1.8	**1.8**	1.3		2.4	**2.4**	1.6	**1.6**	2.1	**2.1**
ADAR	adenosine deaminase, RNA-specific					-1.3				-1.2	**-1.2**		
ADARB1	adenosine deaminase, RNA-specific, B1 (RED1 homolog rat)	1.3		1.4				-34.5	**-34.5**			1.7	**1.7**
ADCY3	adenylate cyclase 3	1.2		1.4	**1.4**			-1.2		1.3		-1.8	**-1.8**
ADCY7	adenylate cyclase 7	1.3		1.3		-1.4		-3.1	**-3.1**	-1.5	**-1.5**	3.7	**3.7**
ADD1	adducin 1 (alpha)			1.6	**1.6**	1.2		1.2	**1.2**	1.8	**1.8**	2.0	**2.0**
ADD3	adducin 3 (gamma)	1.4		1.5	**1.5**					2.7	**2.7**	2.8	**2.8**
ADFP	adipose differentiation-related protein							3.3	**3.3**	1.5	**1.5**	-2.0	**-2.0**
ADH5	alcohol dehydrogenase 5 (class III), chi polypeptide			1.4		-1.3		2.4	**2.4**	-1.3		-2.1	**-2.1**
ADIPOR2	adiponectin receptor 2			-1.3				-1.5	**-1.5**	1.9	**1.9**	1.2	
ADK	adenosine kinase	1.2		-1.2		-1.6		1.6	**1.6**	-2.0	**-2.0**	-4.1	**-4.1**
ADM	adrenomedullin	1.5		2.4		1.6							
ADNP	activity-dependent neuroprotector					-1.5		-1.4	**-1.4**	-1.2	**-1.2**	1.5	**1.5**
ADRBK1	adrenergic, beta, receptor kinase 1							-2.0	**-2.0**	-1.6		-1.3	**-1.3**
ADRBK2	adrenergic, beta, receptor kinase 2	-1.4				-1.3		1.6	**1.6**	-1.5	**-1.5**	-2.2	**-2.2**
ADRM1	adhesion regulating molecule 1			-1.2				2.2	**2.2**			-3.5	**-3.5**
ADSL	adenylosuccinate lyase					-1.3		1.5	**1.5**	-1.5	**-1.5**	-2.4	**-2.4**
AEBP1	AE binding protein 1					1.2		-1.6	**-1.6**	1.5	**1.5**	2.1	**2.1**
AES	amino-terminal enhancer of split	1.3	**1.2**	1.4	**1.4**			-2.1	**-2.1**	-1.3		3.0	**3.0**
AFF1	AF4/FMR2 family, member 1							-3.5	**-3.5**	2.8	**2.8**	8.6	**8.6**
AFG3L2	AFG3 ATPase family gene 3-like 2 (yeast)	-1.3	**-1.3**	-1.5	**-1.5**			1.3	**1.3**	-1.4		-2.0	**-2.0**
AGA	aspartylglucosaminidase					-1.8		-1.3	**-1.3**	-1.5		-1.7	**-1.7**
AGER	advanced glycosylation end product-specific receptor	1.4				1.6							
AGGF1	angiogenic factor with G patch and FHA domains 1	1.3		1.5		-1.2		1.8	**1.8**	-1.6		-1.8	**-1.8**
AGL	amylo-1, 6-glucosidase, 4-alpha-glucanotransferase (glycogen debranching enzyme, glycogen storage disease type III)	1.3		1.3		-1.4		-2.0	**-2.0**	-1.4		3.3	**3.3**
AGPAT1	1-acylglycerol-3-phosphate O-acyltransferase 1 (lysophosphatidic acid acyltransferase, alpha)	1.4	**1.4**	1.5	**1.5**	1.4		1.8	**1.8**	-1.4	**-1.4**	1.3	**1.3**
AGPAT2	1-acylglycerol-3-phosphate O-acyltransferase 2 (lysophosphatidic acid acyltransferase, beta)	-1.2						1.5	**1.5**			-1.4	**-1.4**
AGPS	alkylglycerone phosphate synthase			1.5		1.3		5.5	**5.5**	5.0	**5.0**	-2.6	**-2.6**
AGRN	agrin	-1.3		-1.6				-1.6	**-1.6**	-2.8	**-2.8**	1.6	
AHCY	S-adenosylhomocysteine hydrolase	-1.3		-1.6	**-1.6**	-1.4		1.4	**1.4**	-1.4	**-1.4**	-2.7	**-2.7**
AHCYL1	S-adenosylhomocysteine hydrolase-like 1	-1.3		-1.4		-1.2		1.6	**1.6**	-1.4	**-1.4**	-1.6	**-1.6**
AHNAK	AHNAK nucleoprotein (desmoyokin)					2.3	**2.3**						
AHSA1	AHA1, activator of heat shock 90kDa protein ATPase homolog 1 (yeast)	-1.3		-1.3	**-1.3**	-1.3				-1.7	**-1.7**	-1.4	**-1.4**
AIF1	allograft inflammatory factor 1	1.6				-1.4		2.5	**2.5**			-39.1	**-39.1**
AIM1	absent in melanoma 1	3.5	**3.5**	6.1	**6.1**	13.8	**13.8**			-1.3		8.3	**8.3**
AIP	aryl hydrocarbon receptor interacting protein	-1.3						-1.4	**-1.4**			1.2	
AK2	adenylate kinase 2	-1.4		-1.7	**-1.7**	-1.6	**-1.6**	-1.4	**-1.4**	-2.2	**-2.2**	-3.3	**-3.3**
AKAP1	A kinase (PRKA) anchor protein 1	-1.6	**-1.6**	-2.5	**-2.5**	-1.9		-1.8	**-1.8**	-2.4	**-2.4**	-2.2	**-2.2**
AKAP10	A kinase (PRKA) anchor protein 10					-1.2		-2.7	**-2.7**			2.9	**2.9**
AKAP11	A kinase (PRKA) anchor protein 11			1.2				-1.2	**-1.2**	-1.3	**-1.3**	-1.5	**-1.5**
AKAP13	A kinase (PRKA) anchor protein 13	1.9						-2.9	**-2.9**	4.1	**4.1**	51.0	**51.0**
AKAP2	A kinase (PRKA) anchor protein 2									4.0	**4.0**	2.2	**2.2**
AKAP8	A kinase (PRKA) anchor protein 8	-1.3		-1.4				1.4				-1.9	**-1.9**
AKAP9	A kinase (PRKA) anchor protein (yotiao) 9					-1.5		-1.2		-1.4		1.6	**1.6**
AKR1A1	aldo-keto reductase family 1, member A1 (aldehyde reductase)							-2.6	**-2.6**	-2.3	**-2.3**	-1.4	
AKR1B1	aldo-keto reductase family 1, member B1 (aldose reductase)							-4.2	**-4.2**	-1.2	**-1.2**	8.1	**8.1**
AKR1C2	aldo-keto reductase family 1, member C2 (dihydrodiol dehydrogenase 2; bile acid binding protein; 3-alpha hydroxysteroid dehydrogenase, type III)					2.1	**2.1**						

AKR1C3	aldo-keto reductase family 1, member C3 (3-alpha hydroxysteroid dehydrogenase, type II)	1.4		1.9	**1.9**					3.7	**3.7**	90.6	**90.6**
AKR7A2	aldo-keto reductase family 7, member A2 (aflatoxin aldehyde reductase)	1.2				-1.2		-1.6	**-1.6**			-1.3	**-1.3**
AKT1	v-akt murine thymoma viral oncogene homolog 1			-1.3				-1.5	**-1.5**			1.3	**1.3**
ALAS1	aminolevulinate, delta-, synthase 1					-1.3		2.4	**2.4**			-1.9	**-1.9**
ALCAM	activated leukocyte cell adhesion molecule					1.8		1.4	**1.4**	4.0	**4.0**	-4.0	**-4.0**
ALDH1A2	aldehyde dehydrogenase 1 family, member A2			2.1	**2.1**	1.6							
ALDH4A1	aldehyde dehydrogenase 4 family, member A1	1.6	**1.6**	1.7	**1.7**	1.4		1.9	**1.9**			-3.5	**-3.5**
ALDH5A1	aldehyde dehydrogenase 5 family, member A1 (succinate-semialdehyde dehydrogenase)							-1.5	**-1.5**	-1.4	**-1.4**	-2.2	**-2.2**
ALDH6A1	aldehyde dehydrogenase 6 family, member A1	1.5		2.3	**2.3**	1.6	**1.6**	1.6	**1.6**	-1.7	**-1.7**		
ALDH9A1	aldehyde dehydrogenase 9 family, member A1					-1.3				-1.6	**-1.6**	-1.6	**-1.6**
ALDOA	aldolase A, fructose-bisphosphate	-1.2		-1.5				-1.5	**-1.5**	-1.3	**-1.3**	-1.3	**-1.3**
ALDOC	aldolase C, fructose-bisphosphate			-1.4	**-1.4**								
ALG3	asparagine-linked glycosylation 3 homolog (S. cerevisiae, alpha-1,3-mannosyltransferase)	-1.2		-1.8	**-1.8**	-1.2							
ALOX5AP	arachidonate 5-lipoxygenase-activating protein	2.4	**2.4**	2.2	**2.2**	1.8	**1.8**					2.3	**2.3**
ALS2CR3	trafficking protein, kinesin binding 2	1.4				-1.2		1.7	**1.7**	2.7	**2.4**	2.8	**2.8**
AMD1	adenosylmethionine decarboxylase 1	-1.4	**-1.4**			-1.3		1.4	**1.4**			-2.4	**-2.4**
AMFR	autocrine motility factor receptor									1.2		1.3	**1.3**
AMPD2	adenosine monophosphate deaminase 2 (isoform L)			-1.4	**-1.4**			1.9	**1.9**	-2.5	**-2.5**	-2.9	**-2.9**
ANAPC10	anaphase promoting complex subunit 10			1.3		-1.4		1.8	**1.8**	1.3		-1.3	**-1.3**
ANAPC13	anaphase promoting complex subunit 13					-1.2		1.2	**1.2**	-1.3	**-1.3**	-1.7	**-1.7**
ANAPC5	anaphase promoting complex subunit 5					-1.4		1.9	**1.5**	-1.8	**-1.8**	-2.3	**-2.3**
ANGPT1	angiopoietin 1			1.6		-1.3		1.8	**1.8**			-8.8	**-8.8**
ANKRD28	ankyrin repeat domain 28					-2.0	**-2.0**	2.2	**2.2**	2.2	**2.2**	-4.1	**-4.1**
ANKRD40	ankyrin repeat domain 40	-1.4				-1.2		-1.5	**-1.5**	-1.5		1.4	**1.4**
ANKS1	ankyrin repeat and sterile alpha motif domain containing 1A							1.8		3.3	**3.3**	-1.4	**-1.4**
ANP32A	acidic (leucine-rich) nuclear phosphoprotein 32 family, member A	1.3		1.2	**1.2**	1.2		2.1	**2.1**	-1.3	**-1.3**	-1.8	**-1.8**
ANP32B	acidic (leucine-rich) nuclear phosphoprotein 32 family, member B			1.2						-1.3	**-1.3**	-1.6	**-1.6**
ANXA1	annexin A1	-1.4		-1.6									
ANXA11	annexin A11							1.8	**1.8**	1.8	**1.8**	-1.2	**-1.2**
ANXA2	annexin A2					-1.3				1.4	**1.4**	2.7	**2.7**
ANXA3	annexin A3												
ANXA4	annexin A4							1.2		1.3	**1.3**	1.8	**1.8**
ANXA5	annexin A5	1.2		1.5				-2.0	**-2.0**	2.0	**2.0**	3.6	**3.6**
ANXA6	annexin A6	1.2		1.5	**1.5**	1.3	**1.3**	2.4	**2.4**			-1.3	**-1.3**
ANXA7	annexin A7							1.6	**1.6**			-1.3	**-1.3**
AOF2	amine oxidase (flavin containing) domain 2			-1.3		-1.3				1.5	**1.5**	-1.9	**-1.9**
AP1B1	adaptor-related protein complex 1, beta 1 subunit					1.5		-1.4	**-1.4**	1.5		2.2	**2.2**
AP1G1	adaptor-related protein complex 1, gamma 1 subunit			1.2				-1.3		-1.4	**-1.2**	1.6	**1.6**
AP1G2	adaptor-related protein complex 1, gamma 2 subunit	1.4		1.3								2.3	**2.3**
AP1S2	adaptor-related protein complex 1, sigma 2 subunit	1.8	**1.8**	2.3	**2.3**	1.2		-27.2	**-27.2**	1.6	**1.6**	21.8	**21.8**
AP2B1	adaptor-related protein complex 2, beta 1 subunit							1.6		1.6	**1.6**	1.6	**1.6**
AP2M1	adaptor-related protein complex 2, mu 1 subunit							1.2					
AP2S1	adaptor-related protein complex 2, sigma 1 subunit	1.3	**1.3**									-1.9	**-1.9**
AP3B1	adaptor-related protein complex 3, beta 1 subunit									-1.3		1.6	**1.6**
AP3D1	adaptor-related protein complex 3, delta 1 subunit					-1.3		1.5	**1.5**	1.3	**1.3**		
AP3S1	adaptor-related protein complex 3, sigma 1 subunit	1.6	**1.6**	2.0	**2.0**	1.9	**1.9**	1.4	**1.4**	2.4	**2.4**	2.1	**2.1**
AP3S2	adaptor-related protein complex 3, sigma 2 subunit					-1.3		2.2	**2.2**	-1.4		-1.8	**-1.8**
APBB1	amyloid beta (A4) precursor protein-binding, family B, member 1 (Fe65)			1.2									
APEX1	APEX nuclease (multifunctional DNA repair enzyme) 1			-1.3	**-1.3**	-1.3				-2.2	**-2.2**	-3.4	**-3.4**
APG12L	ATG12 autophagy related 12 homolog (S. cerevisiae)	1.6	**1.6**	2.0	**2.0**	1.9	**1.9**	1.3	**1.3**	1.9	**1.9**	2.5	**2.5**
API5	apoptosis inhibitor 5	1.3		1.7				1.6	**1.6**	-2.1	**-2.1**	-2.0	**-2.0**
APLP2	amyloid beta (A4) precursor-like protein 2							4.2	**4.2**	-1.7	**-1.7**	-12.0	**-12.0**
APOBEC3B	apolipoprotein B mRNA editing enzyme, catalytic polypeptide-like 3B	1.7				1.8		-1.9	**-1.9**			2.0	**2.0**
APOM	apolipoprotein M					1.4				-1.4			
APPBP1	amyloid beta precursor protein binding protein 1	-1.2		-1.6	**-1.6**	-1.5		-1.2	**-1.2**	-1.3	**-1.3**	-1.2	**-1.2**
APPBP2	amyloid beta precursor protein (cytoplasmic tail) binding protein 2	1.3		1.2		1.7		2.0	**2.0**	1.4	**1.4**	-1.3	**-1.3**
APRT	adenine phosphoribosyltransferase			-1.5						-1.7	**-1.7**	-2.6	**-2.6**
AQP3	aquaporin 3 (Gill blood group)					-1.3							

AQP5	aquaporin 5	1.4				1.3							
AQP7	aquaporin 7					1.6							
ARAF	v-raf murine sarcoma 3611 viral oncogene homolog							-1.9		1.3		2.0	
ARCN1	archain 1												
ARD1	ARD1 homolog A, N-acetyltransferase (S. cerevisiae)	-1.3				-1.2							
ARF1	ADP-ribosylation factor 1							1.3	**1.3**	-1.2	**-1.2**	-1.2	**-1.2**
ARF3	ADP-ribosylation factor 3	1.3	**1.3**			1.2		1.4	**1.4**	-1.3	**-1.3**	-1.3	**-1.3**
ARF4	ADP-ribosylation factor 4							1.3	**1.3**	-1.3		-1.6	**-1.6**
ARF5	ADP-ribosylation factor 5					1.4		-2.4	**-2.4**			2.0	**2.0**
ARF6	ADP-ribosylation factor 6	1.2		1.2				1.4	**1.4**	1.7	**1.7**	1.7	**1.7**
ARFGAP3	ADP-ribosylation factor GTPase activating protein 3					1.3		1.4	**1.4**				
ARFGEF1	ADP-ribosylation factor guanine nucleotide-exchange factor 1(brefeldin A-inhibited)							-1.6	**-1.6**	1.5	**1.5**	1.8	**1.8**
ARFGEF2	ADP-ribosylation factor guanine nucleotide-exchange factor 2 (brefeldin A-inhibited)			1.3				1.3	**1.3**			1.4	**-1.2**
ARFIP1	ADP-ribosylation factor interacting protein 1 (arfaptin 1)	1.2						1.3					
ARFIP2	ADP-ribosylation factor interacting protein 2 (arfaptin 2)							1.4				-1.9	**-1.9**
ARFRP1	ADP-ribosylation factor related protein 1			-1.4									
ARHGAP1	Rho GTPase activating protein 1					1.2		-1.2				1.4	**1.2**
ARHGAP11A	Rho GTPase activating protein 11A			1.2				-1.2		1.6	**1.6**		
ARHGAP12	Rho GTPase activating protein 12	-1.2				-1.3	**-1.3**	1.7	**1.7**			-1.3	
ARHGAP4	Rho GTPase activating protein 4									1.5			
ARHGAP5	Rho GTPase activating protein 5			1.7				-1.4	**-1.4**	-1.2		2.3	**2.3**
ARHGDIA	Rho GDP dissociation inhibitor (GDI) alpha	-1.3		-2.0				-2.1	**-1.3**	-2.4		-1.3	
ARHGDIB	Rho GDP dissociation inhibitor (GDI) beta			1.4	**1.4**			-1.4	**-1.4**			1.5	**1.5**
ARHGEF1	Rho guanine nucleotide exchange factor (GEF) 1	1.4	**1.4**			1.5	**1.5**					1.3	
ARHGEF12	Rho guanine nucleotide exchange factor (GEF) 12					3.4		-1.6	**-1.5**			1.4	
ARHGEF18	rho/rac guanine nucleotide exchange factor (GEF) 18	1.3	**1.3**	1.7	**1.7**	1.4		1.5	**1.5**	2.5	**2.5**	3.3	**3.3**
ARHGEF2	rho/rac guanine nucleotide exchange factor (GEF) 2					-1.3		1.3	**1.3**	1.3	**1.3**	-1.7	**-1.7**
ARHGEF6	Rac/Cdc42 guanine nucleotide exchange factor (GEF) 6	1.3	**1.3**	1.8	**1.8**			-1.4	**-1.4**	-1.3	**-1.3**		
ARHGEF7	Rho guanine nucleotide exchange factor (GEF) 7	1.3		1.4	**1.4**	1.3		-1.8	**-1.8**	3.6	**3.6**	5.1	**5.1**
ARID1A	AT rich interactive domain 1A (SWI-like)			1.2				-1.3	**-1.3**	1.5	**1.5**	2.1	**2.1**
ARID3A	AT rich interactive domain 3A (BRIGHT-like)			1.2		1.3							
ARID4A	AT rich interactive domain 4A (RBP1-like)			1.6				2.9	**2.9**	1.4			
ARID5B	AT rich interactive domain 5B (MRF1-like)	-1.3						-34.1	**-34.1**	-2.2	**-2.2**	8.6	**8.6**
ARIH1	ariadne homolog, ubiquitin-conjugating enzyme E2 binding protein, 1 (Drosophila)					-1.4		1.7	**1.7**	1.6	**1.6**	1.8	**1.8**
ARIH2	ariadne homolog 2 (Drosophila)							-1.5	**-1.5**	1.6	**1.6**	-2.4	**-2.4**
ARL1	ADP-ribosylation factor-like 1	-1.2	**-1.2**	1.3		-1.6		1.3	**1.3**	-1.4		-1.5	**-1.5**
ARL2	ADP-ribosylation factor-like 2	-1.3						-1.3				-1.7	**-1.7**
ARL2BP	ADP-ribosylation factor-like 2 binding protein	1.3								-1.3		1.7	**1.7**
ARL4A	ADP-ribosylation factor-like 4A					1.4		26.8	**26.8**			-33.8	**-33.8**
ARL6IP	ADP-ribosylation factor-like 6 interacting protein 1			1.2	**1.2**			-2.2	**-2.2**	1.7	**1.7**	2.7	**2.7**
ARL6IP5	ADP-ribosylation-like factor 6 interacting protein 5	1.7		1.8	**1.8**					1.4	**1.4**	2.2	**2.2**
ARL7	ADP-ribosylation factor-like 4C			1.4		-1.4				2.8	**2.8**		
ARMET	arginine-rich, mutated in early stage tumors			-1.2						1.4	**1.4**	-1.6	**-1.6**
ARNT	aryl hydrocarbon receptor nuclear translocator					1.7		1.7		1.4		-1.3	
ARPC1A	actin related protein 2/3 complex, subunit 1A, 41kDa							-2.8	**-2.8**	1.5		2.2	**2.2**
ARPC1B	actin related protein 2/3 complex, subunit 1B, 41kDa					-1.3	**-1.3**	1.3	**1.3**			-1.3	
ARPC2	actin related protein 2/3 complex, subunit 2, 34kDa							-1.3	**-1.3**			1.4	**1.4**
ARPC3	actin related protein 2/3 complex, subunit 3, 21kDa	1.2								1.4	**1.4**		
ARPC4	actin related protein 2/3 complex, subunit 4, 20kDa							-4.7	**-4.7**	1.3		4.1	**4.1**
ARPC5	actin related protein 2/3 complex, subunit 5, 16kDa	1.3		1.4	**1.4**			-1.4	**-1.4**	1.4	**1.4**	2.3	**2.3**
ARPP-19	cyclic AMP phosphoprotein, 19 kD			1.3		-1.2		-2.5	**-2.5**	-1.6		2.4	**2.4**
ARRB2	arrestin, beta 2			-1.4	**-1.4**	-1.3		-2.1	**-2.1**			1.3	**1.3**
ASAH1	N-acylsphingosine amidohydrolase (acid ceramidase) 1	1.4						4.4	**4.4**	-1.4	**-1.3**	-3.8	**-3.8**
ASB1	ankyrin repeat and SOCS box-containing 1							-1.8	**-1.8**	1.6		1.4	**1.4**
ASB9	ankyrin repeat and SOCS box-containing 9			1.7		-1.3				1.3			
ASCC2	activating signal cointegrator 1 complex subunit 2												
ASCC3	activating signal cointegrator 1 complex subunit 3					-2.2	**-2.2**			-1.3		-1.5	**-1.5**
ASCC3L1	activating signal cointegrator 1 complex subunit 3-like 1							-1.5	**-1.5**	-1.4	**-1.4**		

ASF1A	ASF1 anti-silencing function 1 homolog A (S. cerevisiae)			1.9	**1.9**							-1.2	**-1.2**
ASH2L	ash2 (absent, small, or homeotic)-like (Drosophila)											-1.4	
ASK	DBF4 homolog (S. cerevisiae)					-1.3		-1.7	**-1.7**	1.5	**1.5**		
ASMTL	acetylserotonin O-methyltransferase-like					1.3		-2.7	**-2.7**	-1.2		3.2	**3.2**
ASNA1	arsA arsenite transporter, ATP-binding, homolog 1 (bacterial)									-1.4	**-1.4**	-1.3	**-1.3**
ASNS	asparagine synthetase			-1.2				2.9	**2.9**			-9.0	**-9.0**
ASS	argininosuccinate synthetase 1	-1.3		-1.4				-1.4	**-1.4**	-3.8	**-3.8**	-2.0	**-2.0**
ASXL1	additional sex combs like 1 (Drosophila)							-1.6	**-1.6**	-1.4	**-1.4**	-2.2	**-2.2**
ATF2	activating transcription factor 2			1.6		-1.4		1.6	**1.6**	1.5	**1.5**	-1.5	**-1.5**
ATF3	activating transcription factor 3					-1.8		-1.7		-1.5	**-1.5**	-2.0	**-2.0**
ATF4	activating transcription factor 4 (tax-responsive enhancer element B67)	-1.5	**-1.5**	-1.3		-1.3		-1.3	**-1.3**	-1.4	**-1.4**		
ATF5	activating transcription factor 5	-1.5		-1.9	**-1.9**	-1.3		-1.6	**-1.6**	-2.0	**-2.0**	-1.4	
ATF6	activating transcription factor 6	1.3						5.8	**5.8**	-1.4		-5.3	**-5.3**
ATIC	5-aminoimidazole-4-carboxamide ribonucleotide formyltransferase/IMP cyclohydrolase	-1.4	**-1.4**	-1.9	**-1.9**	-2.0	**-2.0**	-2.1	**-2.1**	-3.2	**-3.2**	-1.3	**-1.3**
ATM	ataxia telangiectasia mutated (includes complementation groups A, C and D)	1.8						1.5		1.2	**1.2**	1.5	**1.3**
ATOX1	ATX1 antioxidant protein 1 homolog (yeast)							5.6	**5.6**			-13.3	**-13.3**
ATP11B	ATPase, Class VI, type 11B	1.3		1.5	**1.5**	1.5	**1.5**	-1.6	**-1.6**	5.1	**5.1**	4.7	**4.7**
ATP1A1	ATPase, Na+/K+ transporting, alpha 1 polypeptide							1.3	**1.3**	-1.6	**-1.6**	-2.4	**-2.4**
ATP1A3	ATPase, Na+/K+ transporting, alpha 3 polypeptide	-1.5		-3.1				-13.1	**-13.1**				
ATP1B3	ATPase, Na+/K+ transporting, beta 3 polypeptide			-1.3	**-1.3**	-1.4		-1.6				1.9	**1.9**
ATP2A1	ATPase, Ca++ transporting, cardiac muscle, fast twitch 1					-1.3							
ATP2A2	ATPase, Ca++ transporting, cardiac muscle, slow twitch 2	1.2		-1.4		1.3		1.3	**1.3**	-1.5	**-1.5**	-2.5	**-2.5**
ATP2A3	ATPase, Ca++ transporting, ubiquitous	1.2						2.5	**2.5**	1.2	**1.2**	-2.0	**-2.0**
ATP2B1	ATPase, Ca++ transporting, plasma membrane 1	-1.4				-2.0		-2.1	**-2.1**	-2.1	**-2.1**		
ATP2B2	ATPase, Ca++ transporting, plasma membrane 2					1.8							
ATP2B4	ATPase, Ca++ transporting, plasma membrane 4			1.3		-1.2		-3.8	**-3.8**	2.8	**2.8**	3.9	**3.9**
ATP2C1	ATPase, Ca++ transporting, type 2C, member 1			1.5	**1.5**	1.3		1.9	**1.9**	1.2		1.5	**1.5**
ATP5A1	ATP synthase, H+ transporting, mitochondrial F1 complex, alpha subunit 1, cardiac muscle			1.3				1.3	**1.3**			-1.5	**-1.5**
ATP5C1	ATP synthase, H+ transporting, mitochondrial F1 complex, gamma polypeptide 1					-1.4		1.4	**1.4**			-1.5	**-1.5**
ATP5D	ATP synthase, H+ transporting, mitochondrial F1 complex, delta subunit							-1.2				-1.7	**-1.7**
ATP5F1	ATP synthase, H+ transporting, mitochondrial F0 complex, subunit B1			2.1		1.3						1.3	**1.3**
ATP5G1	ATP synthase, H+ transporting, mitochondrial F0 complex, subunit C1 (subunit 9)	-1.2		-1.5	**-1.5**	-1.3	**-1.3**			-2.4	**-2.4**	-3.9	**-3.9**
ATP5G3	ATP synthase, H+ transporting, mitochondrial F0 complex, subunit C3 (subunit 9)					-1.2		2.3	**2.3**			-3.8	**-3.8**
ATP5H	ATP synthase, H+ transporting, mitochondrial F0 complex, subunit d					-1.2		1.5	**1.5**	-1.2	**-1.2**	-2.4	**-2.4**
ATP5I	ATP synthase, H+ transporting, mitochondrial F0 complex, subunit E			-1.2		-1.3	**-1.3**			1.3	**1.3**	-1.4	
ATP5J	ATP synthase, H+ transporting, mitochondrial F0 complex, subunit F6					-1.3		-1.8	**-1.8**			3.0	**3.0**
ATP5J2	ATP synthase, H+ transporting, mitochondrial F0 complex, subunit F2							-1.3	**-1.3**				
ATP5L	ATP synthase, H+ transporting, mitochondrial F0 complex, subunit G			1.2				1.7	**1.7**	1.6	**1.6**	-1.6	**-1.6**
ATP5O	ATP synthase, H+ transporting, mitochondrial F1 complex, O subunit (oligomycin sensitivity conferring protein)									-1.2	**-1.2**	-1.8	**-1.8**
ATP5S	ATP synthase, H+ transporting, mitochondrial F0 complex, subunit s (factor B)					1.4		-1.4		2.9	**2.9**	2.0	**2.0**
ATP6AP1	ATPase, H+ transporting, lysosomal accessory protein 1							1.3					
ATP6AP2	ATPase, H+ transporting, lysosomal accessory protein 2			1.5	**1.5**			2.2	**2.2**	-1.2	**1.2**	-1.8	**-1.8**
ATP6V0A2	ATPase, H+ transporting, lysosomal V0 subunit a2											-1.6	**-1.6**
ATP6V0B	ATPase, H+ transporting, lysosomal 21kDa, V0 subunit b	-1.2										-1.6	**-1.6**
ATP6V0C	ATPase, H+ transporting, lysosomal 16kDa, V0 subunit c							-1.4	**-1.4**	1.3	**1.3**	1.9	**1.9**
ATP6V0D1	ATPase, H+ transporting, lysosomal 38kDa, V0 subunit d1	1.3	**1.3**	1.3		1.3		1.4	**1.4**	1.2		1.7	**-1.3**
ATP6V0E	ATPase, H+ transporting, lysosomal 9kDa, V0 subunit e1					-1.4		1.3	**1.3**	-1.5		-1.4	**-1.4**
ATP6V0E2	ATPase, H+ transporting V0 subunit e2	1.7		1.7	**1.7**	2.2							
ATP6V1A	ATPase, H+ transporting, lysosomal 70kDa, V1 subunit A	1.5	**1.5**	2.5	**2.5**	1.3		1.9	**1.9**			-1.9	**-1.9**
ATP6V1B1	ATPase, H+ transporting, lysosomal 56/58kDa, V1 subunit B1 (Renal tubular acidosis with deafness)	1.3		-1.4		1.3							
ATP6V1B2	ATPase, H+ transporting, lysosomal 56/58kDa, V1 subunit B2			1.2				1.5	**1.5**				
ATP6V1C1	ATPase, H+ transporting, lysosomal 42kDa, V1 subunit C1	-1.4	**-1.4**	1.3				-2.1	**-2.1**			2.1	**2.1**
ATP6V1D	ATPase, H+ transporting, lysosomal 34kDa, V1 subunit D			1.6	**1.6**			-1.3	**-1.3**	1.3	**1.3**	1.9	**1.9**
ATP6V1E1	ATPase, H+ transporting, lysosomal 31kDa, V1 subunit E1									1.2		1.4	**1.4**
ATP6V1F	ATPase, H+ transporting, lysosomal 14kDa, V1 subunit F									-1.3	**-1.3**		
ATP6V1G1	ATPase, H+ transporting, lysosomal 13kDa, V1 subunit G1	-1.2		1.3								1.3	**1.3**
ATP6V1H	ATPase, H+ transporting, lysosomal 50/57kDa, V1 subunit H	1.3								1.2		1.4	**1.4**
ATP7A	ATPase, Cu++ transporting, alpha polypeptide (Menkes syndrome)	2.0										1.5	**1.5**

ATP9B	ATPase, Class II, type 9B							-1.7	**-1.7**			-1.3	
ATR	ataxia telangiectasia and Rad3 related					-1.2						-1.3	**-1.3**
ATRN	attractin	-1.2		1.3				1.6				-1.4	**-1.4**
ATRX	alpha thalassemia/mental retardation syndrome X-linked (RAD54 homolog, S. cerevisiae)					-1.3		1.4	**1.4**			1.7	**1.7**
ATXN10	ataxin 10	-1.3	**-1.3**	-1.3	**-1.3**	-1.5	**-1.5**	1.3				-1.7	**-1.7**
ATXN2	ataxin 2	-1.4				-1.3				-1.3		-1.3	
ATXN2L	ataxin 2-like							-2.2	**-2.2**			1.8	**1.8**
ATXN3	ataxin 3			1.4		1.2		1.2		1.4	**1.3**	2.0	**2.0**
AUH	AU RNA binding protein/enoyl-Coenzyme A hydratase	2.5	**2.5**	2.2	**2.2**	1.7	**1.7**			1.4		2.4	**2.4**
AURKB	aurora kinase B							-3.3	**-3.3**	1.4	**1.4**	3.0	**3.0**
B2M	beta-2-microglobulin	-1.5						-1.6	**-1.6**			3.1	**3.1**
B3GNT1	UDP-GlcNAc:betaGal beta-1,3-N-acetylglucosaminyltransferase 1			-1.6				-2.7	**-2.7**	-1.4		1.4	
B4GALT1	UDP-Gal:betaGlcNAc beta 1,4- galactosyltransferase, polypeptide 1			-1.3				1.3		1.3	**1.3**	1.7	**1.7**
B4GALT2	UDP-Gal:betaGlcNAc beta 1,4- galactosyltransferase, polypeptide 2	-1.8	**-1.8**	-3.3	**-3.3**			-1.3		-2.1	**-2.1**	-2.4	**-2.4**
B4GALT3	UDP-Gal:betaGlcNAc beta 1,4- galactosyltransferase, polypeptide 3	-1.3		-1.4				1.3		-1.3		-1.4	**-1.4**
B4GALT4	UDP-Gal:betaGlcNAc beta 1,4- galactosyltransferase, polypeptide 4			1.6	**1.6**			1.5				8.1	**8.1**
B4GALT6	UDP-Gal:betaGlcNAc beta 1,4- galactosyltransferase, polypeptide 6					-1.6		2.5	**2.5**	1.6		-6.7	**-6.7**
B930013M22RIK	ras responsive element binding protein 1			1.3				3.1	**3.1**	2.1	**2.1**	1.5	**1.5**
BACH1	BTB and CNC homology 1, basic leucine zipper transcription factor 1			1.3		1.3		1.2	**1.2**	1.3	**1.3**	1.2	**1.2**
BAD	BCL2-antagonist of cell death	1.2											
BAG1	BCL2-associated athanogene							-1.2		-1.4	**-1.4**	-2.0	**-2.0**
BAG2	BCL2-associated athanogene 2	-1.2				-1.6		-1.2	**-1.2**	-1.8	**-1.8**	-1.5	**-1.5**
BAG5	BCL2-associated athanogene 5					-1.3						-1.4	**-1.4**
BAIAP1	membrane associated guanylate kinase, WW and PDZ domain containing 1					1.7		-3.3	**-3.3**			2.1	
BAMBI	BMP and activin membrane-bound inhibitor homolog (Xenopus laevis)					-1.7	**-1.7**	-2.7	**-2.7**			1.5	**1.5**
BANF1	barrier to autointegration factor 1			-1.3				-2.0	**-2.0**	-1.5	**-1.5**		
BAP1	BRCA1 associated protein-1 (ubiquitin carboxy-terminal hydrolase)									-1.7	**-1.7**	-1.2	
BAPX1	bagpipe homeobox homolog 1 (Drosophila)			1.3									
BARD1	BRCA1 associated RING domain 1	1.3	**1.3**					-3.6	**-3.6**	1.4	**1.3**	3.4	**3.4**
BAT1	HLA-B associated transcript 1							1.5	**1.5**	-1.6	**-1.6**	-1.4	
BAT2D1	BAT2 domain containing 1	1.4				-1.2		1.2		-1.4		-1.4	**-1.4**
BAT3	HLA-B associated transcript 3							-1.4	**-1.4**	-1.4	**-1.4**		
BAT8	euchromatic histone-lysine N-methyltransferase 2			-1.4	**-1.4**					-4.0	**-4.0**	-2.8	**-2.8**
BAX	BCL2-associated X protein	-1.2		-1.4	**-1.4**	1.3		-1.8	**-1.8**				
BAZ1A	bromodomain adjacent to zinc finger domain, 1A	-1.2		-1.3	**-1.3**	-1.6	**-1.6**	1.3				-1.6	**-1.6**
BAZ1B	bromodomain adjacent to zinc finger domain, 1B					-1.2		-1.9	**-1.9**	-1.2	**-1.2**		
BAZ2A	bromodomain adjacent to zinc finger domain, 2A	1.3										-2.9	**-2.9**
BBS4	Bardet-Biedl syndrome 4					-2.3	**-2.3**			-1.2			
BBX	bobby sox homolog (Drosophila)			1.5	**1.5**			1.6	**1.6**	-1.3	**-1.3**	1.8	**1.8**
BC-2	chromatin modifying protein 2A	1.2		1.4				-1.4	**-1.4**	1.5	**1.5**	1.5	**1.5**
BCAP29	B-cell receptor-associated protein 29			1.6				2.0	**2.0**	1.5	**1.5**	1.4	
BCAP31	B-cell receptor-associated protein 31												
BCAS2	breast carcinoma amplified sequence 2			-1.4		-1.9							
BCAT1	branched chain aminotransferase 1, cytosolic	1.4		1.3				-4.9	**-4.9**	-3.3	**-3.3**	2.2	**2.2**
BCAT2	branched chain aminotransferase 2, mitochondrial	-1.3	**-1.3**	-1.3		-1.6		1.7	**1.7**			-2.7	**-2.7**
BCKDHA	branched chain keto acid dehydrogenase E1, alpha polypeptide			1.3	**1.3**			1.5				-2.2	**-2.2**
BCKDHB	branched chain keto acid dehydrogenase E1, beta polypeptide (maple syrup urine disease)					-1.4		2.2	**2.2**			-2.5	**-2.5**
BCL10	B-cell CLL/lymphoma 10							1.2	**1.2**	-1.5			
BCL11A	B-cell CLL/lymphoma 11A (zinc finger protein)	-1.2						-1.8	**-1.8**			1.2	**1.2**
BCL2	B-cell CLL/lymphoma 2	1.4						-11.2	**-11.2**	-2.8	**-2.8**	1.5	**1.5**
BCL2L1	BCL2-like 1	-1.3		-1.4	**-1.4**	-2.0				1.7	**1.7**		
BCL2L11	BCL2-like 11 (apoptosis facilitator)	3.1	**3.1**	14.5	**14.5**	5.9	**5.9**	1.5	**1.2**	3.1	**3.1**	5.1	**5.1**
BCL6	B-cell CLL/lymphoma 6 (zinc finger protein 51)	1.7		3.0	**3.0**			-11.8	**-11.8**	1.3		6.6	**6.6**
BCL7A	B-cell CLL/lymphoma 7A			-1.4	**-1.4**	-1.4		-3.3	**-3.3**	-1.3	**-1.3**	1.6	**1.5**
BCL7B	B-cell CLL/lymphoma 7B					-1.2		-1.4	**-1.4**	-1.4			
BCLAF1	BCL2-associated transcription factor 1	-1.3	**-1.3**			-1.5		2.0	**2.0**	-1.7	**-1.7**	-1.8	**-1.8**
BCR	breakpoint cluster region	1.2		1.8	**1.8**	1.3		-1.4	**-1.4**			4.7	**4.7**
BDH1	3-hydroxybutyrate dehydrogenase, type 1	-1.3	**-1.3**	-1.5		-1.5	**-1.5**	-1.3	**-1.3**	-1.7		-1.6	**-1.6**

BECN1	beclin 1 (coiled-coil, myosin-like BCL2 interacting protein)							1.2				1.7	**1.7**
BET1	BET1 homolog (S. cerevisiae)					-1.4		-2.2	**-2.2**	-1.4		1.6	**1.6**
BHLHB2	basic helix-loop-helix domain containing, class B, 2	-1.3				-2.0							
BICD1	bicaudal D homolog 1 (Drosophila)	1.3						-2.6	**-2.6**	-1.4	**-1.4**	3.6	**3.6**
BICD2	bicaudal D homolog 2 (Drosophila)			-1.5	**-1.5**			-1.2	**-1.2**	-1.9		1.3	**-1.2**
BID	BH3 interacting domain death agonist	1.4	**1.4**	1.6	**1.6**	1.5		-3.4	**-3.4**	-1.9	**-1.9**	-1.3	**-1.3**
BIK	BCL2-interacting killer (apoptosis-inducing)			-1.7	**-1.7**	2.0	**2.0**			6.3	**6.3**		
BIN1	bridging integrator 1					1.6	**1.6**	-1.3	**-1.3**			9.6	**9.6**
BIRC2	baculoviral IAP repeat-containing 2	1.9	**1.9**	1.7	**1.7**	1.5		1.4	**1.4**	1.9	**1.9**	1.6	**1.6**
BIRC3	--	2.8	**2.8**	6.8	**6.8**	5.8	**5.8**						
BIRC5	baculoviral IAP repeat-containing 5 (survivin)							-1.8	**-1.8**			2.4	**2.4**
BLCAP	bladder cancer associated protein							1.4	**1.4**	-1.5	**-1.5**	-1.4	**-1.4**
BLM	Bloom syndrome					-1.4				-1.8	**-1.8**	-2.6	**-2.6**
BLMH	bleomycin hydrolase			1.4	**1.4**	-1.6		1.3	**1.3**			-1.3	
BLOC1S1	biogenesis of lysosome-related organelles complex-1, subunit 1							-1.9	**-1.9**			1.6	**1.6**
BMP2K	BMP2 inducible kinase							1.5	**1.5**	1.4		-1.6	**-1.6**
BNIP1	BCL2/adenovirus E1B 19kDa interacting protein 1					1.4						-1.2	
BNIP2	BCL2/adenovirus E1B 19kDa interacting protein 2	1.5	**1.5**					-1.4	**-1.4**	1.7	**1.7**	1.4	**1.4**
BNIP3	BCL2/adenovirus E1B 19kDa interacting protein 3	1.4	**1.4**	1.2				312.5	**312.5**			-331.2	**-331.2**
BNIP3L	BCL2/adenovirus E1B 19kDa interacting protein 3-like	1.7		1.4	**1.4**			2.0	**2.0**	-1.3	**-1.3**	1.9	**1.9**
BOP1	block of proliferation 1	-1.4	**-1.4**	-1.8	**-1.8**	-1.6		1.3		-2.6	**-2.6**	-3.6	**-3.6**
BPGM	2,3-bisphosphoglycerate mutase							-1.5	**-1.5**	1.2		1.4	
BPHL	biphenyl hydrolase-like (serine hydrolase; breast epithelial mucin-associated antigen)												
BRAP	BRCA1 associated protein	1.3								1.3	**1.3**		
BRCA1	breast cancer 1, early onset			1.4	**1.4**			-1.5	**-1.5**	-1.4	**-1.4**	-2.6	**-2.6**
BRCA2	breast cancer 2, early onset					-1.3		-2.7	**-2.7**	1.6			
BRD2	bromodomain containing 2			-1.2	**-1.2**			-1.4				1.5	**1.5**
BRD3	bromodomain containing 3									-1.5	**-1.5**	-1.2	
BRD4	bromodomain containing 4	1.2		1.2		2.1		-1.3	**-1.3**			-1.7	**1.4**
BRD8	bromodomain containing 8	1.6	**1.6**	1.4	**1.4**					1.4	**1.4**	1.4	
BRE	brain and reproductive organ-expressed (TNFRSF1A modulator)							-1.8		-2.1	**-2.1**	3.0	**3.0**
BRMS1	breast cancer metastasis suppressor 1			-1.6	**-1.6**			-1.6	**-1.6**				
BRP44	brain protein 44	1.2		1.3						-1.3	**-1.3**	-1.4	**-1.4**
BRRN1	non-SMC condensin I complex, subunit H							-1.9	**-1.9**			-1.8	**-1.8**
BSCL2	Bernardinelli-Seip congenital lipodystrophy 2 (seipin)	1.2											
BSG	basigin (Ok blood group)			-1.4		1.2		1.7		-1.4	**-1.4**	-2.4	**-2.4**
BST1	bone marrow stromal cell antigen 1					-1.3							
BST2	bone marrow stromal cell antigen 2			1.4	**1.4**			-1.5	**-1.5**			1.4	**1.4**
BTAF1	BTAF1 RNA polymerase II, B-TFIID transcription factor-associated, 170kDa (Mot1 homolog, S. cerevisiae)							1.6	**1.6**			-2.0	**-2.0**
BTBD2	BTB (POZ) domain containing 2			-1.4				-1.4		-1.8	**-1.8**	1.6	**1.6**
BTF3	basic transcription factor 3					-1.3				1.3			
BTG1	B-cell translocation gene 1, anti-proliferative	9.3	**9.3**	9.1	**9.1**	4.4	**4.4**	1.2		3.9	**3.9**	8.8	**8.8**
BTG2	BTG family, member 2	3.1	**3.1**	2.9	**2.9**	2.3	**2.3**			3.9	**3.9**	58.4	**58.4**
BTG3	BTG family, member 3	-1.2		-1.2				-1.6	**-1.6**	1.2		-2.2	**-2.2**
BTN3A2	butyrophilin, subfamily 3, member A2							-5.8	**-5.8**	-1.2		3.9	**3.9**
BTN3A3	butyrophilin, subfamily 3, member A3					-2.0		-5.9	**-5.9**	-1.4	**-1.4**	4.2	**4.2**
BUB1	BUB1 budding uninhibited by benzimidazoles 1 homolog (yeast)			1.4				-1.3	**-1.3**	1.4	**1.4**	-1.3	**-1.3**
BUB1B	BUB1 budding uninhibited by benzimidazoles 1 homolog beta (yeast)	1.3						-1.9	**-1.9**			1.3	
BUB3	BUB3 budding uninhibited by benzimidazoles 3 homolog (yeast)							-1.4	**-1.4**	1.3	**1.3**	-1.4	**-1.4**
BUD31	BUD31 homolog (yeast)												
BYSL	bystin-like	-2.2	**-2.2**	-1.8	**-1.8**	-2.3	**-2.3**	-6.2	**-6.2**	-8.4	**-8.4**	-4.0	**-4.0**
BZRP	translocator protein (18kDa)	1.3		1.4		1.4				-1.8	**-1.8**	-1.4	**-1.4**
C13ORF24	chromosome 13 open reading frame 24					-1.2		1.2	**1.2**			-1.5	**-1.5**
C14ORF11	chromosome 14 open reading frame 11	-1.5		1.4		1.2							
C19ORF10	chromosome 19 open reading frame 10	-1.3		-1.5	**-1.5**			1.3	**1.3**			-2.2	**-2.2**
C19ORF2	chromosome 19 open reading frame 2	-1.3		-1.4	**-1.4**	-1.2		1.8	**1.8**	-1.3	**-1.3**	-2.7	**-2.7**
C19ORF29	chromosome 19 open reading frame 29			-1.2		1.7				-1.6	**-1.6**		
C1D	nuclear DNA-binding protein			-1.3	**-1.3**	-1.4		-1.6	**-1.6**				

C1ORF16	Smg-7 homolog, nonsense mediated mRNA decay factor (C. elegans)			-1.4		-1.2						-2.0	**-2.0**
C1ORF48	NSL1, MIND kinetochore complex component, homolog (S. cerevisiae)					-1.3		-1.5	**-1.5**	-1.3		1.4	**1.4**
C1QBP	complement component 1, q subcomponent binding protein	-1.8	**-1.8**	-2.0	**-2.0**	-2.0		-2.0	**-2.0**	-1.9	**-1.9**		
C1QL1	complement component 1, q subcomponent-like 1					1.5							
C2	complement component 2	1.4		1.2		1.7							
C20ORF14	PRP6 pre-mRNA processing factor 6 homolog (S. cerevisiae)									-1.3			
C20ORF18	RanBP-type and C3HC4-type zinc finger containing 1			-1.3		-1.3						1.3	**1.3**
C21ORF33	chromosome 21 open reading frame 33	1.3	**1.2**					1.8	**1.8**			-2.5	**-2.5**
C3F	membrane bound O-acyltransferase domain containing 5	1.8	**1.8**	-1.3				-1.2		-1.5	**-1.5**		
C5ORF13	chromosome 5 open reading frame 13	-1.3	**-1.3**	-1.4	**-1.4**	-1.5		-3.7	**-3.7**	-3.3	**-3.3**	4.7	**4.7**
C5ORF18	receptor accessory protein 5					1.7		3.6	**3.6**	1.4	**1.4**	-4.6	**-4.6**
C5ORF22	chromosome 5 open reading frame 22					-1.3		1.2	**1.2**			-1.4	**-1.3**
C6ORF108	chromosome 6 open reading frame 108					-1.2		2.7	**2.7**	-3.3	**-3.3**	-51.4	**-51.4**
C6ORF69	potassium channel tetramerisation domain containing 20			1.5				-1.5	**-1.3**			1.2	**1.2**
C7ORF44	chromosome 7 open reading frame 44					-1.4		2.8	**2.8**	-1.4		-4.8	**-4.8**
CABIN1	calcineurin binding protein 1	1.7	**1.7**	1.5		2.0	**2.0**	1.4	**-1.3**	-1.4	**-1.4**		
CACNB3	calcium channel, voltage-dependent, beta 3 subunit	1.9	**1.9**	1.5	**1.5**					1.4	**1.4**	1.2	**1.2**
CACYBP	calcyclin binding protein					2.0		1.7	**-1.5**	-1.5	**-1.5**	-1.9	**-1.9**
CAD	carbamoyl-phosphate synthetase 2, aspartate transcarbamylase, and dihydroorotase	-1.3		-1.8	**-1.8**	-1.6				-2.8	**-2.8**	-3.7	**-3.7**
CALB2	calbindin 2, 29kDa (calretinin)												
CALCOCO1	calcium binding and coiled-coil domain 1	1.6				1.8				1.3		1.6	**1.6**
CALM1	calmodulin 1 (phosphorylase kinase, delta)	-1.4		1.5	**-1.5**	2.1		-1.4	**-1.4**	1.6	**1.6**	4.4	**4.4**
CALM2	calmodulin 2 (phosphorylase kinase, delta)			-1.3				-1.2	**-1.2**	1.2	**1.2**		
CALM3	calmodulin 3 (phosphorylase kinase, delta)	1.2						-1.5	**-1.5**	-1.2	**1.2**	1.3	**1.3**
CALR	calreticulin	-1.9	**-1.9**	-1.7	**-1.7**	-1.6		4.7	**4.7**	-1.5	**-1.4**	-5.3	**-5.3**
CALU	calumenin			-1.4				-1.4		-2.6	**-2.6**	-1.3	**-1.3**
CAMK2B	calcium/calmodulin-dependent protein kinase (CaM kinase) II beta					1.7						1.2	
CAMK2G	calcium/calmodulin-dependent protein kinase (CaM kinase) II gamma					1.5		2.1	**2.1**			-2.6	**-2.6**
CAMK4	calcium/calmodulin-dependent protein kinase IV					1.3		-5.8	**-5.8**	-1.7		8.0	**8.0**
CAMKK2	calcium/calmodulin-dependent protein kinase kinase 2, beta	-1.3		-1.6				1.7	**1.7**	-2.1	**-2.1**	-3.7	**-3.7**
CAMLG	calcium modulating ligand					-1.2		2.9	**2.9**	-1.5	**-1.5**	-2.0	**-2.0**
CAMTA2	calmodulin binding transcription activator 2	1.4	**1.4**	1.3				-1.5	**-1.5**			2.0	**2.0**
CANX	calnexin	-1.3		-1.4	**-1.4**	-1.3		1.6	**1.6**	-1.3	**-1.3**	-2.5	**-2.5**
CAP1	CAP, adenylate cyclase-associated protein 1 (yeast)			1.4	**1.4**			1.3	**1.3**	1.3	**1.3**	1.7	**1.7**
CAP350	centrosomal protein 350kDa							1.6	**1.6**	2.3	**2.3**	1.5	**1.5**
CAPG	capping protein (actin filament), gelsolin-like					-1.6		-49.5	**-49.5**			61.6	**61.6**
CAPN1	calpain 1, (mu/I) large subunit	1.3						1.2				2.8	**2.8**
CAPN2	calpain 2, (m/II) large subunit							2.4	**2.4**	1.2		-2.2	**-2.2**
CAPN7	calpain 7	1.4		1.4		1.3		1.4	**1.4**	1.5		1.2	
CAPNS1	calpain, small subunit 1							-1.3	**-1.3**			1.6	**1.6**
CAPZA1	--	1.2						1.3	**1.3**				
CAPZA1	capping protein (actin filament) muscle Z-line, alpha 1												
CAPZA2	capping protein (actin filament) muscle Z-line, alpha 2					1.2		-2.1	**-2.1**	1.8	**1.8**	1.9	**1.9**
CAPZB	capping protein (actin filament) muscle Z-line, beta											1.2	**1.2**
CARD8	caspase recruitment domain family, member 8			1.8				1.3	**1.3**			-1.3	**-1.3**
CARM1	coactivator-associated arginine methyltransferase 1	-1.6	**-1.6**					-1.8	**-1.8**	-1.7	**-1.7**	-1.4	**-1.4**
CARS	cysteinyl-tRNA synthetase			-1.2	**-1.2**	-1.4	**-1.4**	-1.7	**-1.7**	-1.6	**-1.2**		
CASC3	cancer susceptibility candidate 3												
CASK	calcium/calmodulin-dependent serine protein kinase (MAGUK family)							-1.3	**-1.3**			1.2	**1.2**
CASP10	caspase 10, apoptosis-related cysteine peptidase	1.3		1.4		1.4	**1.4**						
CASP2	caspase 2, apoptosis-related cysteine peptidase (neural precursor cell expressed, developmentally down-regulated 2)			1.9				-3.1	**-3.1**	-1.8	**-1.8**	2.2	**2.2**
CASP3	caspase 3, apoptosis-related cysteine peptidase					-1.4							
CASP4	caspase 4, apoptosis-related cysteine peptidase	1.5	**1.5**	1.9	**1.9**	1.3				2.0	**2.0**	1.9	**1.9**
CASP6	caspase 6, apoptosis-related cysteine peptidase					-1.3		2.4	**2.4**	1.4	**1.4**	-1.8	**-1.8**
CASP7	caspase 7, apoptosis-related cysteine peptidase									1.3	**1.3**		
CASP8	caspase 8, apoptosis-related cysteine peptidase	1.3		2.7	**2.7**	1.3		3.2	**3.2**	1.5	**1.5**	-1.5	**-1.5**
CASP9	caspase 9, apoptosis-related cysteine peptidase	1.3		1.4		1.3		-1.3	**-1.3**			1.3	**1.3**
CAST	calpastatin	1.4		2.1	**2.1**			2.5	**2.5**	1.5	**1.5**	2.4	**2.4**

CAT	catalase	1.3	**1.3**	1.8	**1.8**			4.5	**4.5**	-1.3	**-1.3**	1.4	**1.4**
CBFA2T2	core-binding factor, runt domain, alpha subunit 2; translocated to, 2			1.4		1.2		1.5	**1.5**	1.3		-1.3	**-1.3**
CBFA2T3	core-binding factor, runt domain, alpha subunit 2; translocated to, 3	-2.1	**-2.1**	-2.3	**-2.3**					1.5	**1.5**	1.3	**1.3**
CBFB	core-binding factor, beta subunit							-2.3	**-2.3**			1.5	**1.5**
CBL	Cas-Br-M (murine) ecotropic retroviral transforming sequence			-1.3				1.6	**1.6**			-1.2	
CBLB	Cas-Br-M (murine) ecotropic retroviral transforming sequence b			2.1	**2.1**	1.2		-1.6	**-1.6**	1.8	**1.8**	4.1	**4.1**
CBX1	chromobox homolog 1 (HP1 beta homolog Drosophila)					-1.4						-1.6	**-1.6**
CBX3	chromobox homolog 3 (HP1 gamma homolog, Drosophila)	-1.2		-1.3		-1.2		2.2	**2.2**	-1.8	**1.2**	-2.1	**-2.1**
CBX5	chromobox homolog 5 (HP1 alpha homolog, Drosophila)	1.2				-1.2		-10.9	**-10.9**	-2.1		3.1	**3.1**
CBX6	chromobox homolog 6	-1.3		-1.3	**-1.3**			-1.4	**-1.4**	-2.1	**-2.1**	-2.0	**-2.0**
CBX7	chromobox homolog 7												
CCBL1	cysteine conjugate-beta lyase; cytoplasmic (glutamine transaminase K, kyneurenine aminotransferase)					1.4							
CCBP2	chemokine binding protein 2			-1.2		1.7							
CCDC28A	coiled-coil domain containing 28A	1.6	**1.6**	1.7	**1.7**			-1.3	**-1.3**			1.8	**1.8**
CCDC6	coiled-coil domain containing 6	1.3		2.4	**2.4**	1.6		2.6	**2.6**	1.9	**1.9**	-4.5	**-4.5**
CCDC85B	coiled-coil domain containing 85B	-1.4		-1.9	**-1.9**	-1.3		1.2		-1.8	**-1.8**	-4.1	**-4.1**
CCHCR1	coiled-coil alpha-helical rod protein 1					1.4	**1.4**	-1.3		1.2		1.5	
CCKAR	cholecystokinin A receptor	1.2											
CCNA2	cyclin A2			1.3				-2.2	**-2.2**	1.3	**1.3**	1.5	**1.5**
CCNB1	cyclin B1							-1.5	**-1.5**	1.9	**1.9**	2.6	**2.6**
CCNB2	cyclin B2	1.4	**1.4**	1.5	**1.5**					1.4	**1.4**	1.4	**1.4**
CCNC	cyclin C			1.5		-1.2		1.4	**1.3**	-1.8		-1.4	**-1.4**
CCND3	cyclin D3			-1.4	**-1.4**	-1.8	**-1.8**	-10.6	**-10.6**			7.2	**7.2**
CCNE1	cyclin E1			1.7				2.3	**2.3**			-14.9	**-14.9**
CCNE2	cyclin E2	1.2	**1.2**	1.3	**1.3**			-2.9	**-2.9**	-1.3	**-1.3**	2.2	**2.2**
CCNF	cyclin F	1.3		1.2		1.6		-2.6	**-2.6**	2.0	**2.0**	2.7	**2.7**
CCNG1	cyclin G1			1.7		1.3		1.8	**1.8**	1.3	**1.3**		
CCNG2	cyclin G2	2.3	**2.3**	1.6	**1.6**	1.3				2.2	**2.2**	3.1	**3.1**
CCNH	cyclin H							1.6	**1.6**	1.3	**1.3**	-1.5	**-1.5**
CCNI	cyclin I			1.3				-1.7	**-1.7**	1.5	**1.5**	1.6	**1.6**
CCNT1	cyclin T1							1.3				-1.2	
CCNT2	cyclin T2	-1.4		-1.2				2.6	**2.6**	2.0	**2.0**	-2.0	**-2.0**
CCR4	chemokine (C-C motif) receptor 4					4.7							
CCR7	chemokine (C-C motif) receptor 7	-1.6	**-1.6**	-1.4	**-1.4**	-1.4							
CCRL2	chemokine (C-C motif) receptor-like 2	1.2		1.5									
CCT2	chaperonin containing TCP1, subunit 2 (beta)	-1.3	**-1.3**	-1.4	**-1.4**	-1.3				-2.2	**-2.2**	-2.1	**-2.1**
CCT3	chaperonin containing TCP1, subunit 3 (gamma)	-1.3		-1.5	**-1.5**	-1.4		1.2	**1.2**	-1.6	**-1.6**	-1.7	**-1.7**
CCT4	chaperonin containing TCP1, subunit 4 (delta)					-1.4				-1.4	**-1.4**	-1.4	**-1.4**
CCT5	chaperonin containing TCP1, subunit 5 (epsilon)	-1.6	**-1.6**	-1.8	**-1.8**	-1.7		-1.3		-1.5	**-1.5**	-1.5	**-1.5**
CCT6A	chaperonin containing TCP1, subunit 6A (zeta 1)	-1.6	**-1.6**	-1.4	**-1.4**	-1.7	**-1.7**	4.9	**4.9**	-1.4		-5.1	**-5.1**
CCT7	chaperonin containing TCP1, subunit 7 (eta)	-1.4		-1.5	**-1.5**	-1.6	**-1.6**			-1.5	**-1.5**	-2.1	**-2.1**
CCT8	chaperonin containing TCP1, subunit 8 (theta)	-1.7		-1.5	**-1.5**	-1.6		1.2	**1.2**	-1.6	**-1.6**	-1.3	**-1.3**
CD164	CD164 molecule, sialomucin	1.3		1.4	**1.4**	1.5		-1.7	**-1.7**	1.8	**1.8**	2.6	**2.6**
CD1A	CD1a molecule	-1.5		-1.8	**-1.8**								
CD1C	CD1c molecule	-1.4											
CD1D	CD1d molecule	-2.0	**-2.0**	-1.2									
CD1E	CD1e molecule	-2.6	**-2.6**	-2.0	**-2.0**								
CD2AP	CD2-associated protein											1.4	**1.4**
CD34	CD34 molecule							12.3	**12.3**	-3.0	**-3.0**	-18.1	**-18.1**
CD37	CD37 molecule	1.2						2.5	**2.5**			-4.9	**-4.9**
CD38	CD38 molecule	-1.3						-2.9	**-2.9**	-2.4	**-2.4**	8.4	**8.4**
CD3D	CD3d molecule, delta (CD3-TCR complex)	1.2	**1.2**	1.3	**1.3**								
CD3E	CD3e molecule, epsilon (CD3-TCR complex)			1.2		1.3							
CD3G	CD3g molecule, gamma (CD3-TCR complex)			1.2									
CD3Z	CD247 molecule												
CD4	CD4 molecule	1.2		1.4	**1.4**			5.4	**5.4**			-5.8	**-5.8**
CD40	CD40 molecule, TNF receptor superfamily member 5					1.5		-1.4	**-1.4**			1.3	**1.3**
CD44	CD44 molecule (Indian blood group)	-1.7	**-1.7**			-1.9		3.2	**3.2**	-3.0	**-3.0**	-13.1	**-13.1**

CD47	CD47 molecule	1.7	**1.7**	1.9	**1.9**	1.5		-2.4	**-2.4**	-1.7	**-1.7**	2.0	**2.0**
CD48	CD48 molecule	1.9	**1.9**	2.6	**2.6**			-14.5	**-14.5**	-2.5	**-2.5**	4.3	**4.3**
CD52	CD52 molecule	1.6										98.6	**98.6**
CD53	CD53 molecule	3.1	**3.1**	3.4	**3.4**	3.2	**3.2**	1.4	**1.4**	3.1	**3.1**	7.2	**7.2**
CD58	CD58 molecule			1.3				-1.3		3.0	**3.0**	2.5	**2.5**
CD59	CD59 molecule, complement regulatory protein	1.3		2.2	**2.2**	1.8	**1.8**	-3.7	**-3.7**	1.3		10.6	**10.6**
CD6	CD6 molecule			-1.3									
CD63	CD63 molecule			1.5	**1.5**			1.4	**1.4**	1.2	**1.2**		
CD69	CD69 molecule	4.9	**4.9**	4.7	**4.7**	2.0	**2.0**	6.3	**6.3**	3.8	**3.8**	-1.5	**-1.5**
CD7	CD7 molecule	1.3	**1.3**			1.3							
CD72	CD72 molecule					1.4		-23.3	**-23.3**	1.6	**1.6**	31.2	**31.2**
CD79A	CD79a molecule, immunoglobulin-associated alpha	2.0	**2.0**	2.5	**2.5**	3.6	**3.6**	-24.9	**-24.9**	1.3	**1.3**	24.6	**24.6**
CD81	CD81 molecule	-1.2		-1.3		-1.2		-1.9	**-1.9**			1.3	**1.3**
CD83	CD83 molecule							2.1	**2.1**	-1.4		-1.6	
CD8A	CD8a molecule	1.9		2.8	**2.8**	2.0							
CD99	CD99 molecule	1.2		1.5	**1.5**	1.8	**1.8**	1.7	**1.7**	3.4	**3.4**	2.6	**2.6**
CDC123	cell division cycle 123 homolog (S. cerevisiae)	-1.3	**-1.3**	-1.4	**-1.4**	-1.6				-1.7	**-1.7**	-2.0	**-2.0**
CDC16	cell division cycle 16 homolog (S. cerevisiae)							1.6	**1.6**	1.2	**1.2**	1.2	
CDC2	cell division cycle 2, G1 to S and G2 to M			-1.2				-1.6	**-1.6**	2.0	**2.0**		
CDC20	cell division cycle 20 homolog (S. cerevisiae)									1.5	**1.5**		
CDC23	cell division cycle 23 homolog (S. cerevisiae)							-1.9	**-1.9**	-1.3	**-1.3**	-2.1	**-2.1**
CDC25A	cell division cycle 25 homolog A (S. cerevisiae)	-1.4	**-1.4**	-1.2		-1.4		-1.6	**-1.6**	-1.4	**-1.4**	-19.4	**-19.4**
CDC25B	cell division cycle 25 homolog B (S. cerevisiae)							-1.2	**-1.2**	1.6	**1.6**	1.6	**1.6**
CDC25C	cell division cycle 25 homolog C (S. cerevisiae)	1.4		1.5	**1.5**					1.5			
CDC27	cell division cycle 27 homolog (S. cerevisiae)							1.2		1.4	**1.4**	-1.5	**-1.5**
CDC2L1	cell division cycle 2-like 1 (PITSLRE proteins)	1.3				1.3							
CDC2L2	cell division cycle 2-like 2 (PITSLRE proteins)									1.5	**1.5**	1.3	
CDC2L6	cell division cycle 2-like 6 (CDK8-like)	1.6		1.3		1.8	**1.8**	-1.4	**-1.4**	3.3	**3.3**	3.8	**3.8**
CDC34	cell division cycle 34 homolog (S. cerevisiae)											-1.2	
CDC37	cell division cycle 37 homolog (S. cerevisiae)			-1.3		-1.2							
CDC40	cell division cycle 40 homolog (S. cerevisiae)							1.3		-1.2			
CDC42EP3	CDC42 effector protein (Rho GTPase binding) 3					2.0		-1.6	**-1.6**	4.0	**4.0**	6.2	**6.2**
CDC45L	CDC45 cell division cycle 45-like (S. cerevisiae)												
CDC5L	CDC5 cell division cycle 5-like (S. pombe)									1.3	**1.3**	1.6	**1.6**
CDC6	cell division cycle 6 homolog (S. cerevisiae)	-1.3	**-1.3**	-1.2		-1.5	**-1.5**	-1.7	**-1.7**	-2.9	**-2.9**	-4.7	**-4.7**
CDC7	cell division cycle 7 homolog (S. cerevisiae)	1.3	**1.3**					-1.4	**-1.4**	-1.4	**-1.4**	-2.4	**-2.4**
CDH2	cadherin 2, type 1, N-cadherin (neuronal)					-1.4		30.0	**30.0**			-72.0	**-72.0**
CDH4	cadherin 4, type 1, R-cadherin (retinal)	1.3		1.5	**1.5**	1.5				2.0	**2.0**	1.2	
CDIPT	CDP-diacylglycerol--inositol 3-phosphatidyltransferase (phosphatidylinositol synthase)							-1.6	**-1.6**	1.4	**1.4**	2.8	**2.8**
CDK2	cyclin-dependent kinase 2			1.3	**1.3**			-2.3	**-2.3**	-1.5	**-1.5**	-1.6	**-1.6**
CDK2AP1	CDK2-associated protein 1					-1.4							
CDK4	cyclin-dependent kinase 4	-1.4	**-1.4**	-1.8	**-1.8**	-1.6	**-1.6**			-2.4	**-2.4**	-4.4	**-4.4**
CDK5	cyclin-dependent kinase 5	1.2				1.3		-1.9				1.6	**1.6**
CDK6	cyclin-dependent kinase 6			1.6	**1.6**	1.2		-3.0	**-3.0**	-1.5	**-1.5**	2.1	**2.1**
CDK7	cyclin-dependent kinase 7 (MO15 homolog, Xenopus laevis, cdk-activating kinase)					-1.6		2.1	**2.1**			-1.6	**-1.6**
CDK8	cyclin-dependent kinase 8			1.2				-1.5	**-1.5**			1.3	
CDK9	cyclin-dependent kinase 9 (CDC2-related kinase)	1.3		1.6	**1.6**			-7.3	**-7.3**	1.7	**1.7**	10.9	**10.9**
CDKN1B	cyclin-dependent kinase inhibitor 1B (p27, Kip1)	1.7	**1.7**	1.4				-1.6	**-1.6**	1.6	**1.6**	3.6	**3.6**
CDKN2C	cyclin-dependent kinase inhibitor 2C (p18, inhibits CDK4)	1.2		1.6	**1.6**	1.3				1.5	**1.3**	1.6	**1.6**
CDKN2D	cyclin-dependent kinase inhibitor 2D (p19, inhibits CDK4)											11.1	**11.1**
CDKN3	cyclin-dependent kinase inhibitor 3 (CDK2-associated dual specificity phosphatase)					-1.2		-1.4	**-1.4**	1.5	**1.5**	1.2	**1.2**
CDR2	cerebellar degeneration-related protein 2, 62kDa	-1.7				-1.7							
CDS2	CDP-diacylglycerol synthase (phosphatidate cytidylyltransferase) 2					1.3				1.6	**1.6**	1.6	**1.5**
CDT1	chromatin licensing and DNA replication factor 1	-1.3		-1.4	**-1.4**			-1.3		-1.4		-4.6	**-4.6**
CDYL	chromodomain protein, Y-like									1.5		-1.2	
CEBPB	CCAAT/enhancer binding protein (C/EBP), beta	-1.4		-1.9	**-1.9**	-1.4		1.5	**1.5**	-1.5	**-1.5**	-3.1	**-3.1**
CEBPG	CCAAT/enhancer binding protein (C/EBP), gamma	-1.2						-1.3	**-1.3**			-1.6	**-1.6**
CEBPZ	CCAAT/enhancer binding protein zeta	-1.5	**-1.5**	-1.4	**-1.4**	-1.8	**-1.8**	-2.2	**-1.7**	-2.6	**-1.5**	-2.5	**-2.5**

CENPA	centromere protein A	1.3		1.3	**1.3**			-1.4	**-1.3**	1.7	**1.7**	1.4	**1.4**
CENPB	centromere protein B, 80kDa											-1.5	**-1.5**
CENPC1	centromere protein C 1	1.5	**1.5**	1.6	**1.6**					1.3	**1.3**		
CENPE	centromere protein E, 312kDa					1.4	**1.4**			1.6	**1.6**	2.0	**2.0**
CENPF	centromere protein F, 350/400ka (mitosin)							-2.3	**-2.3**	1.5	**1.5**	2.3	**2.3**
CENTB1	centaurin, beta 1							-21.1	**-21.1**			42.8	**42.8**
CENTB2	centaurin, beta 2	1.3		1.8	**1.8**	1.3	**1.3**	1.5	**1.5**	1.5	**1.5**	1.3	
CENTD1	centaurin, delta 1			-1.2				-1.2		1.8	**1.8**	3.3	**3.3**
CENTD2	centaurin, delta 2												
CENTG2	centaurin, gamma 2			1.8						-1.3	**-1.3**		
CEP110	centrosomal protein 110kDa												
CEP2	centrosomal protein 250kDa	1.2											
CETN2	centrin, EF-hand protein, 2							-1.2					
CETN3	centrin, EF-hand protein, 3 (CDC31 homolog, yeast)	-1.4		-1.3		-1.4		-1.5	**-1.5**				
CFL1	cofilin 1 (non-muscle)							-1.6	**-1.6**			1.3	**1.3**
CFLAR	CASP8 and FADD-like apoptosis regulator			1.7		1.5	**1.5**	3.8	**3.8**	2.1	**2.1**	-1.6	
CGB	chorionic gonadotropin, beta polypeptide					1.3							
CGGBP1	CGG triplet repeat binding protein 1			1.4								1.4	**1.4**
CGRRF1	cell growth regulator with ring finger domain 1	1.8		1.3		-1.2						1.2	
CH25H	cholesterol 25-hydroxylase	2.3	**2.3**	1.9									
CHAF1A	chromatin assembly factor 1, subunit A (p150)			-1.3				-1.2		-2.3	**-2.3**	-2.7	**-2.7**
CHAF1B	chromatin assembly factor 1, subunit B (p60)							-1.7	**-1.7**			-2.4	**-2.4**
CHC1	regulator of chromosome condensation 1	-1.7	**-1.7**	-2.1	**-2.1**	-1.6	**-1.6**	-1.2		-1.8	**-1.8**	-1.7	**-1.7**
CHC1L	regulator of chromosome condensation (RCC1) and BTB (POZ) domain containing protein 2	1.4								-1.2		1.4	
CHD1	chromodomain helicase DNA binding protein 1					-1.2		1.2		-1.3	**-1.3**	-1.3	**-1.3**
CHD3	chromodomain helicase DNA binding protein 3			1.2				-9.4	**-9.4**	-1.5	**-1.3**	5.0	**5.0**
CHD4	chromodomain helicase DNA binding protein 4					-1.2				-1.4	**-1.4**		
CHD8	chromodomain helicase DNA binding protein 8									-1.3	**-1.3**		
CHEK1	CHK1 checkpoint homolog (S. pombe)							-1.8	**-1.8**	-1.3	**-1.3**	-2.1	**-2.1**
CHERP	calcium homeostasis endoplasmic reticulum protein			-1.3						-1.4	**-1.4**	-1.6	**-1.6**
CHES1	checkpoint suppressor 1	1.5	**1.5**	1.6	**1.6**			1.8	**1.8**	1.6	**1.6**	1.5	**1.5**
CHI3L2	chitinase 3-like 2	-1.3		-1.9	**-1.9**	-1.8	**-1.8**	-3.1	**-3.1**				
CHIT1	chitinase 1 (chitotriosidase)					1.4							
CHP	calcium binding protein P22	1.2		1.2		1.3		2.4	**2.4**			1.6	**1.6**
CHRM3	cholinergic receptor, muscarinic 3					1.3							
CHRNA5	cholinergic receptor, nicotinic, alpha 5							-2.2	**-2.2**	-1.5		1.7	**1.7**
CHRNB1	cholinergic receptor, nicotinic, beta 1 (muscle)	1.4		1.3	**1.3**	1.4							
CHST1	carbohydrate (keratan sulfate Gal-6) sulfotransferase 1	1.3				1.2							
CHST10	carbohydrate sulfotransferase 10							2.3	**2.3**			-2.6	**-2.6**
CHST2	carbohydrate (N-acetylglucosamine-6-O) sulfotransferase 2	-1.4	**-1.4**	1.6	**1.6**					1.2			
CHSY1	carbohydrate (chondroitin) synthase 1	-1.2						1.8	**1.8**	2.5	**2.5**	-2.1	**-2.1**
CHUK	conserved helix-loop-helix ubiquitous kinase	-1.2		-1.2		-1.3		1.5	**1.5**			-1.5	**-1.5**
CIAPIN1	cytokine induced apoptosis inhibitor 1	1.2		1.7	**1.7**			1.3	**1.3**	-1.6	**-1.6**	-1.8	**-1.8**
CIB1	calcium and integrin binding 1 (calmyrin)	-1.3										-1.3	
CIC	capicua homolog (Drosophila)											2.4	**2.4**
CIR	CBF1 interacting corepressor			1.6	**1.6**	-1.3				1.2			
CIT	citron (rho-interacting, serine/threonine kinase 21)	1.5		1.3									
CITED2	Cbp/p300-interacting transactivator, with Glu/Asp-rich carboxy-terminal domain, 2	1.3		1.4				10.5	**10.5**	2.8	**2.8**	-7.0	**-7.0**
CKAP5	cytoskeleton associated protein 5												
CKB	creatine kinase, brain					1.3		3.2	**3.2**			-5.0	**-5.0**
CKM	creatine kinase, muscle					1.3							
CKS1B	CDC28 protein kinase regulatory subunit 1B	-1.4		-1.6	**-1.6**			-1.6	**-1.6**			-1.4	**-1.4**
CKS2	CDC28 protein kinase regulatory subunit 2							-1.3	**-1.3**	1.2	**1.2**		
CLASP1	cytoplasmic linker associated protein 1					1.7	**1.7**	1.5	**1.5**	1.7		1.8	**1.8**
CLASP2	cytoplasmic linker associated protein 2					-1.3		-1.7	**-1.7**	1.7	**1.7**	2.6	**2.6**
CLCN3	chloride channel 3					1.4		-3.0	**-3.0**	-1.3		3.5	**3.5**
CLCN5	chloride channel 5 (nephrolithiasis 2, X-linked, Dent disease)					1.5		-1.4				-1.6	**-1.6**
CLCN7	chloride channel 7	1.3				1.5		1.2				-1.3	**-1.3**

CLEC11A	C-type lectin domain family 11, member A			-1.3				1.5	**1.5**	1.4	**1.4**	-10.9	**-10.9**
CLIC1	chloride intracellular channel 1							-1.3	**-1.3**	1.3	**1.3**		
CLIC4	chloride intracellular channel 4	-1.4		-1.2		-1.4		-2.2	**-2.2**	1.4		2.0	**2.0**
CLK1	CDC-like kinase 1	1.2		1.7	**1.7**	1.3		1.5	**1.5**	1.7	**1.7**	1.6	**1.6**
CLK2	CDC-like kinase 2												
CLK3	CDC-like kinase 3												
CLN3	ceroid-lipofuscinosis, neuronal 3, juvenile (Batten, Spielmeyer-Vogt disease)											1.6	
CLNS1A	chloride channel, nucleotide-sensitive, 1A	-1.2		-1.3	**-1.3**	-1.4							
CLOCK	clock homolog (mouse)					-1.3		1.2		2.0		-1.4	**-1.4**
CLPP	ClpP caseinolytic peptidase, ATP-dependent, proteolytic subunit homolog (E. coli)	1.5	**1.5**			1.4				-1.3	**-1.3**	-1.3	**-1.3**
CLPX	ClpX caseinolytic peptidase X homolog (E. coli)	-1.2				-1.3							
CLSTN1	calsyntenin 1	1.3	**1.3**					1.3	**1.3**			-1.4	**-1.4**
CLTA	clathrin, light chain (Lca)							1.4	**1.4**	1.4		-1.3	**-1.3**
CLTB	clathrin, light chain (Lcb)	1.3				-1.4		-2.0	**-2.0**	-1.4	**-1.4**	-1.3	**-1.3**
CLTC	clathrin, heavy chain (Hc)					-1.5	**-1.5**	1.4	**1.4**			-1.3	**-1.3**
CLU	clusterin					1.4							
CMA1	chymase 1, mast cell												
CMAH	cytidine monophosphate-N-acetylneuraminic acid hydroxylase (CMP-N-acetylneuraminate monooxygenase)					1.7				-1.4	**-1.4**	1.6	
CNAP1	non-SMC condensin I complex, subunit D2			1.4	**1.4**			-2.7	**-2.7**			2.2	**2.2**
CNIH	cornichon homolog (Drosophila)					-1.4							
CNKSR1	connector enhancer of kinase suppressor of Ras 1												
CNNM2	cyclin M2			1.4				1.7	**1.7**			-1.5	
CNOT1	CCR4-NOT transcription complex, subunit 1	1.2	**1.2**							-1.2	**-1.2**	-1.4	**-1.4**
CNOT4	CCR4-NOT transcription complex, subunit 4							1.5	**-1.3**	1.4	**1.4**	1.4	
CNOT8	CCR4-NOT transcription complex, subunit 8							-1.4	**-1.4**	1.3			
CNP	2',3'-cyclic nucleotide 3' phosphodiesterase	-1.3	**-1.3**			1.3		-1.3	**-1.3**	-1.3		-1.2	
CNTN1	contactin 1					1.3		-1.5		-1.2			
COASY	Coenzyme A synthase			1.3									
COBRA1	cofactor of BRCA1					1.2		-1.3	**-1.3**				
COG2	component of oligomeric golgi complex 2			-1.3								-1.7	**-1.7**
COG4	component of oligomeric golgi complex 4			1.2	**1.2**							1.4	**1.4**
COG5	component of oligomeric golgi complex 5							2.0	**1.3**	-1.3	**-1.3**	-1.4	
COIL	coilin			-1.2		1.3		1.2				-2.0	**-2.0**
COL6A1	collagen, type VI, alpha 1			1.5						2.1	**2.1**		
COL8A1	collagen, type VIII, alpha 1	-1.4								1.3			
COMMD4	COMM domain containing 4	1.2										-1.5	**-1.5**
COMT	catechol-O-methyltransferase	-1.4		-1.7	**-1.7**	-1.4		-2.1	**-2.1**	-1.4		1.9	
COPA	coatomer protein complex, subunit alpha	1.2		1.4	**1.4**			1.2	**1.2**			1.9	**1.9**
COPB	coatomer protein complex, subunit beta 1							1.4	**1.4**				
COPB2	coatomer protein complex, subunit beta 2 (beta prime)			1.2				1.2	**1.2**			1.2	
COPE	coatomer protein complex, subunit epsilon												
COPS2	COP9 constitutive photomorphogenic homolog subunit 2 (Arabidopsis)	-1.2		1.6		-1.3		1.6	**1.6**	-1.2	**-1.2**	-2.4	**-2.4**
COPS3	COP9 constitutive photomorphogenic homolog subunit 3 (Arabidopsis)					-1.2		-2.0	**-2.0**	-1.2	**-1.2**		
COPS5	COP9 constitutive photomorphogenic homolog subunit 5 (Arabidopsis)					-1.3							
COPS6	COP9 constitutive photomorphogenic homolog subunit 6 (Arabidopsis)	-1.2						-1.7	**-1.7**	-1.5	**-1.5**	-1.8	**-1.8**
COPS7A	COP9 constitutive photomorphogenic homolog subunit 7A (Arabidopsis)							-1.4	**-1.4**			1.3	**1.3**
COPS8	COP9 constitutive photomorphogenic homolog subunit 8 (Arabidopsis)					-1.4		-3.2	**-3.2**	1.3		2.6	**2.6**
COQ7	coenzyme Q7 homolog, ubiquinone (yeast)	-1.3		1.2		-1.3						1.8	
CORO1A	coronin, actin binding protein, 1A	1.3				1.3		-1.4		-1.7	**-1.7**		
CORO2A	coronin, actin binding protein, 2A	1.3											
CORT	cortistatin												
COVA1	cytosolic ovarian carcinoma antigen 1			1.3		1.2				1.8	**1.8**	1.8	
COX11	COX11 homolog, cytochrome c oxidase assembly protein (yeast)					-1.5		4.1	**4.1**	-1.4	**-1.3**	-2.9	**-2.9**
COX17	COX17 cytochrome c oxidase assembly homolog (S. cerevisiae)					-1.5				1.6	**1.6**		
COX4I1	cytochrome c oxidase subunit IV isoform 1												
COX5A	cytochrome c oxidase subunit Va			-1.2		-1.2						-1.6	**-1.6**
COX5B	cytochrome c oxidase subunit Vb									-1.2	**-1.2**	-1.6	**-1.6**
COX6A1	cytochrome c oxidase subunit VIa polypeptide 1							-1.2	**-1.2**				

COX6A2	cytochrome c oxidase subunit VIa polypeptide 2					1.3							
COX6B1	cytochrome c oxidase subunit Vib polypeptide 1 (ubiquitous)			1.3								-1.5	**-1.5**
COX6C	cytochrome c oxidase subunit VIc			1.2									
COX7A2	cytochrome c oxidase subunit VIIa polypeptide 2 (liver)												
COX7A2L	cytochrome c oxidase subunit VIIa polypeptide 2 like					-1.3	**-1.3**						
COX7B	cytochrome c oxidase subunit VIIb									-1.3	**-1.3**	-1.7	**-1.7**
COX7C	cytochrome c oxidase subunit VIIc											-1.3	
COX8A	cytochrome c oxidase subunit 8A (ubiquitous)							-1.5	**-1.5**				
CP	ceruloplasmin (ferroxidase)												
CPB2	carboxypeptidase B2 (plasma, carboxypeptidase U)	1.5				1.6							
CPD	carboxypeptidase D	1.4	**1.4**	1.7		1.4		25.6	**25.6**			-4.0	**-4.0**
CPEB3	cytoplasmic polyadenylation element binding protein 3	1.4		1.6	**1.6**	1.3		-1.4		3.0	**3.0**	2.0	
CPNE1	copine I							-1.5	**-1.5**			2.1	**2.1**
CPNE3	copine III	1.5		1.4	**1.4**	1.3		-1.5	**-1.5**			2.7	**2.7**
CPOX	coproporphyrinogen oxidase	1.5	**1.5**	1.2				1.8	**1.8**			-2.6	**-2.6**
CPS1	carbamoyl-phosphate synthetase 1, mitochondrial												
CPSF1	cleavage and polyadenylation specific factor 1, 160kDa							2.3	**2.3**	-1.3		-2.3	**-2.3**
CPSF5	nudix (nucleoside diphosphate linked moiety X)-type motif 21	-1.3		1.7		-1.2		-1.8	**-1.8**	-1.3	**-1.3**	-2.2	**-2.2**
CPSF6	cleavage and polyadenylation specific factor 6, 68kDa							-1.6	**-1.6**	-1.2		-2.0	**-2.0**
CPT1A	carnitine palmitoyltransferase 1A (liver)							1.6	**1.6**	-2.0		-2.0	**-2.0**
CPT1B	carnitine palmitoyltransferase 1B (muscle)	1.2										3.5	**3.5**
CRADD	CASP2 and RIPK1 domain containing adaptor with death domain	1.3				1.4				1.5	**1.5**	1.8	**1.8**
CREB1	cAMP responsive element binding protein 1	1.3		1.3				-1.5	**-1.5**	1.4	**1.4**	2.6	**2.6**
CREB3	cAMP responsive element binding protein 3					1.3				1.6	**1.6**		
CREBBP	CREB binding protein (Rubinstein-Taybi syndrome)							-10.0	**-10.0**	1.3	**1.3**	14.0	**14.0**
CREBL2	cAMP responsive element binding protein-like 2	1.2		1.4				-2.6	**-2.6**	1.3		3.0	**3.0**
CREBZF	CREB/ATF bZIP transcription factor	-1.4		1.3		-1.3		-1.5	**-1.5**	1.6		-1.9	**-1.9**
CREG1	cellular repressor of E1A-stimulated genes 1	1.6	**1.6**	1.4		2.0		1.8	**1.8**	-1.5	**-1.5**	-2.3	**-2.3**
CREM	cAMP responsive element modulator	1.2		1.3				7.7	**7.7**	1.3		-1.9	**-1.9**
CRI1	EP300 interacting inhibitor of differentiation 1							1.9	**1.9**	1.4		-2.5	**-2.5**
CRIP1	cysteine-rich protein 1 (intestinal)			-1.7		-1.3		-16.0	**-16.0**			9.6	**9.6**
CRK	v-crk sarcoma virus CT10 oncogene homolog (avian)					-1.5		-1.2		1.8	**1.8**	2.0	**2.0**
CRKL	v-crk sarcoma virus CT10 oncogene homolog (avian)-like									-1.3		-1.4	**-1.4**
CROP	cisplatin resistance-associated overexpressed protein							1.9	**1.9**	-1.5	**-1.5**	-1.7	**-1.7**
CRSP9	cofactor required for Sp1 transcriptional activation, subunit 9, 33kDa							1.2		2.2	**2.2**	-1.2	**-1.2**
CRTAP (incluc	cartilage associated protein							3.6	**3.6**	-2.2	**-2.2**	-3.4	**-3.4**
CRY1	cryptochrome 1 (photolyase-like)												
CRYAB	crystallin, alpha B	-1.4											
CRYBA4	crystallin, beta A4					1.4							
CRYBB2	crystallin, beta B2			-2.0	**-2.0**								
CRYZ	crystallin, zeta (quinone reductase)					-1.6	**-1.6**	-1.7	**-1.7**	-1.4	**-1.4**	1.3	**1.3**
CS	citrate synthase											-1.2	
CSDA	cold shock domain protein A			1.3	**1.3**	1.3				4.4	**4.4**	6.4	**6.4**
CSE1L	CSE1 chromosome segregation 1-like (yeast)	-1.3		-1.5	**-1.5**	-1.4		-1.6	**-1.6**	-1.4	**-1.4**	-2.5	**-2.5**
CSF1	colony stimulating factor 1 (macrophage)					1.4							
CSK	c-src tyrosine kinase	-1.8	**-1.8**	-1.9	**-1.9**	-1.3		-1.6	**-1.6**			-1.7	**-1.7**
CSNK1A1	casein kinase 1, alpha 1							2.0	**2.0**	1.7	**1.5**	1.3	**1.3**
CSNK1D	casein kinase 1, delta							1.4	**1.4**	1.3	**1.3**		
CSNK1E	casein kinase 1, epsilon					-1.4		-1.3		1.7	**1.7**	1.3	
CSNK1G2	casein kinase 1, gamma 2	1.3				1.4		-1.2	**-1.2**				
CSNK2A1	casein kinase 2, alpha 1 polypeptide	-1.4		-1.2		-1.3		1.5	**1.5**	-1.6	**-1.4**	-2.0	**-2.0**
CSNK2A2	casein kinase 2, alpha prime polypeptide							1.9	**1.9**	1.9	**1.9**		
CSNK2B	casein kinase 2, beta polypeptide					-1.2		-1.3	**-1.3**				
CSPG5	chondroitin sulfate proteoglycan 5 (neuroglycan C)							1.2				-1.7	**-1.7**
CSPG6	structural maintenance of chromosomes 3					-1.6	**-1.6**	2.0	**2.0**	-1.7	**-1.7**	-2.9	**-2.9**
CSRP1	cysteine and glycine-rich protein 1					-1.4		-1.2				-1.3	**-1.3**
CST7	cystatin F (leukocystatin)	1.4						-26.4	**-26.4**	2.4	**2.4**	190.4	**190.4**
CSTB	cystatin B (stefin B)							1.5	**1.5**			1.7	

CSTF1	cleavage stimulation factor, 3' pre-RNA, subunit 1, 50kDa			1.2		-1.3		1.6	**1.6**	1.3	**1.3**	-1.7	**-1.7**
CSTF2	cleavage stimulation factor, 3' pre-RNA, subunit 2, 64kDa												
CSTF3	cleavage stimulation factor, 3' pre-RNA, subunit 3, 77kDa							2.2	**2.2**	-1.3		-4.0	**-4.0**
CTAGE5	CTAGE family, member 5			1.2						1.5		1.3	
CTBP1	C-terminal binding protein 1	1.3	**1.3**	1.4	**1.4**	1.3	**1.3**	-1.5	**-1.5**	1.3	**1.3**	2.2	**2.2**
CTCF	CCCTC-binding factor (zinc finger protein)	-1.2						-1.2	**-1.2**				
CTDP1	CTD (carboxy-terminal domain, RNA polymerase II, polypeptide A) phosphatase, subunit 1					-1.4		-1.3					
CTDSP2	CTD (carboxy-terminal domain, RNA polymerase II, polypeptide A) small phosphatase 2	1.4	**1.4**	1.5	**1.5**	1.6		1.9	**1.9**	-1.3	**-1.3**	-1.3	**-1.2**
CTNNA1	catenin (cadherin-associated protein), alpha 1, 102kDa	1.3	**1.3**	1.6	**1.6**	-1.3				1.7	**1.7**	1.7	**1.7**
CTNNAL1	catenin (cadherin-associated protein), alpha-like 1			1.2		-1.3	**-1.3**	-2.0	**-2.0**				
CTNNB1	catenin (cadherin-associated protein), beta 1, 88kDa							1.4	**1.4**	1.4	**1.4**	2.5	**2.5**
CTPS	CTP synthase	-1.7		-2.5		-1.8		-1.5	**-1.5**	-1.4	**-1.4**	-2.4	**-2.4**
CTR9	Ctr9, Paf1/RNA polymerase II complex component, homolog (S. cerevisiae)			1.2				1.4	**1.4**	-1.3		-1.9	**-1.9**
CTSB	cathepsin B	1.2						1.9	**1.6**	2.1	**2.1**	2.4	**2.4**
CTSC	cathepsin C												
CTSC	--	-1.5	**-1.5**	-1.3	**-1.3**	-1.4		2.7	**2.7**	-5.2	**-5.2**	-7.0	**-7.0**
CTSD	cathepsin D	1.2				1.2		2.0					
CTSH	cathepsin H							-1.4	**-1.4**				
CTSW	cathepsin W												
CUGBP1	CUG triplet repeat, RNA binding protein 1							-1.8	**-1.8**	-1.3	**-1.3**	-1.4	**-1.4**
CUGBP2	CUG triplet repeat, RNA binding protein 2	2.1	**2.0**	2.2	**2.2**	1.8	**1.8**	5.7	**5.7**	2.4	**2.4**	2.0	**1.7**
CUL1	cullin 1	1.7	**1.7**	1.6	**1.6**	1.2		-1.3	**-1.3**			2.0	
CUL2	cullin 2							3.6	**3.6**	1.3		-3.9	**-3.9**
CUL4A	cullin 4A	1.2				-1.4		1.5	**1.5**	1.4	**1.4**	-1.5	**-1.5**
CUL4B	cullin 4B											2.8	**2.8**
CUL5	cullin 5					1.3		1.4	**1.4**			1.3	
CUL7	cullin 7			-1.3								1.6	**1.6**
CUTL1	cut-like 1, CCAAT displacement protein (Drosophila)			1.4	**1.4**	1.5		-1.7	**-1.5**	1.7	**1.7**	1.9	**1.9**
CXCR4	chemokine (C-X-C motif) receptor 4	1.3	**1.3**	1.3	**1.3**	1.5	**1.5**	-2.5	**-2.5**	1.8	**1.8**	63.4	**63.4**
CXORF40A	chromosome X open reading frame 40A					-1.4		-1.2	**-1.2**				
CYB5	cytochrome b5 type A (microsomal)			1.3		1.7		-1.3	**-1.3**	2.3	**2.3**	-1.5	**-1.5**
CYB5-M	cytochrome b5 type B (outer mitochondrial membrane)					-1.4		-1.4	**-1.4**				
CYBA	cytochrome b-245, alpha polypeptide									1.6	**1.6**		
CYC1	cytochrome c-1	-1.3	**-1.3**	-1.3	**-1.3**	-1.5		1.5	**1.5**	-1.6	**-1.6**	-4.4	**-4.4**
CYCS	cytochrome c, somatic	-1.6	**-1.6**	-1.6	**-1.6**	-1.9	**-1.9**	4.9	**4.9**	-1.6	**-1.6**	-13.5	**-13.5**
CYFIP1	cytoplasmic FMR1 interacting protein 1							-2.0	**-2.0**	2.3	**2.3**	2.9	**2.9**
CYFIP2	cytoplasmic FMR1 interacting protein 2			-1.5	**-1.5**	-1.3	**-1.3**	-44.2	**-44.2**	-2.8	**-2.8**	50.3	**50.3**
CYLD	cylindromatosis (turban tumor syndrome)			1.4				2.9		-1.4		-1.9	**-1.9**
CYP11A1	cytochrome P450, family 11, subfamily A, polypeptide 1	1.2				1.6							
CYP2E1	cytochrome P450, family 2, subfamily E, polypeptide 1							-1.4		-2.0			
CYP51A1	cytochrome P450, family 51, subfamily A, polypeptide 1	-1.4				-1.4	**-1.4**	1.3	**1.3**	1.3		-2.1	**-2.1**
D4S234E	DNA segment on chromosome 4 (unique) 234 expressed sequence	1.3								-1.5			
DAAM1	dishevelled associated activator of morphogenesis 1			1.2		-1.5		-3.4	**-3.4**	1.7	**1.7**	8.5	**8.5**
DAB2	disabled homolog 2, mitogen-responsive phosphoprotein (Drosophila)					-1.7		50.8	**50.8**	-2.4	**-2.4**	-32.6	**-32.6**
DACH1	dachshund homolog 1 (Drosophila)					1.2		8.1	**8.1**			-7.6	**-7.6**
DAD1	defender against cell death 1							-4.1	**-4.1**			2.7	**2.7**
DAG1	dystroglycan 1 (dystrophin-associated glycoprotein 1)							1.5	**1.5**				
DAP	death-associated protein			1.2									
DAP3	death associated protein 3	-1.4	**-1.4**	-1.2		-1.6	**-1.6**	1.9	**1.9**			-1.7	**-1.7**
DAPK1	death-associated protein kinase 1					-1.3		3.4	**3.4**			-4.0	**-4.0**
DAPK3	death-associated protein kinase 3	1.2	**1.2**					-1.6	**1.5**	-1.3	**-1.3**	-1.8	**-1.8**
DARS	aspartyl-tRNA synthetase			-1.2		-1.4		1.3	**1.3**	1.2		-1.5	**-1.5**
DATF1	death inducer-obliterator 1			1.4				1.3	**1.3**	1.5	**1.5**	1.8	**1.8**
DAXX	death-associated protein 6			-1.2		-1.2							
DAZAP2	DAZ associated protein 2	1.2						-1.7	**-1.7**	-1.3		1.7	**1.7**
DBI	diazepam binding inhibitor (GABA receptor modulator, acyl-Coenzyme A binding protein)			-1.3	**-1.3**	-1.3		-1.9	**-1.9**	-1.4		1.5	**1.5**
DBN1	drebrin 1	-1.5	**-1.5**	-1.8	**-1.8**	-1.2		-1.5	**-1.5**			-1.3	
DCK	deoxycytidine kinase			1.4	**1.4**			-1.9	**-1.9**	1.3	**1.3**	2.4	**2.4**

DCLRE1A	DNA cross-link repair 1A (PSO2 homolog, S. cerevisiae)			-1.5									
DCP2	DCP2 decapping enzyme homolog (S. cerevisiae)	1.3						-1.5	**-1.5**	1.3	**1.3**		
DCTD	dCMP deaminase	-1.3	**-1.3**	-1.6	**-1.6**	-1.3				-1.2		-1.5	**-1.5**
DCTN1	dynactin 1 (p150, glued homolog, Drosophila)							1.3	**1.3**			1.2	**1.2**
DCTN2	dynactin 2 (p50)			1.4	**1.4**			1.4	**1.4**	1.5	**1.5**	1.6	**1.6**
DCTN3	dynactin 3 (p22)	1.3	**1.3**	1.4	**1.4**	1.3				1.3	**1.3**	2.1	**2.1**
DCTN6	dynactin 6									-1.4	**-1.4**	-1.4	**-1.4**
DDB1	damage-specific DNA binding protein 1, 127kDa					1.2		1.3		-1.3	**-1.3**	-1.6	**-1.6**
DDB2	damage-specific DNA binding protein 2, 48kDa	1.3		1.2	**1.2**			-3.2	**-3.2**	-1.3		2.1	**2.1**
DDC	dopa decarboxylase (aromatic L-amino acid decarboxylase)			-1.3	**-1.3**	-1.3							
DDIT4	DNA-damage-inducible transcript 4	4.4	**4.4**	3.4	**3.4**	2.5		5.5	**5.5**	4.6	**4.6**	22.4	**22.4**
DDOST	dolichyl-diphosphooligosaccharide-protein glycosyltransferase							1.8	**1.8**	-1.4		-1.9	**-1.9**
DDT	D-dopachrome tautomerase					1.3		-1.9	**-1.9**	1.3	**1.3**	1.6	**1.6**
DDX1	DEAD (Asp-Glu-Ala-Asp) box polypeptide 1							1.2	**1.2**	-1.3	**-1.3**	-1.8	**-1.8**
DDX10	DEAD (Asp-Glu-Ala-Asp) box polypeptide 10	-1.2		-1.7	**-1.7**	-1.5		1.2	**1.2**	-2.1	**-2.1**	-2.8	**-2.8**
DDX11	DEAD/H (Asp-Glu-Ala-Asp/His) box polypeptide 11 (CHL1-like helicase homolog, S. cerevisiae)			-1.3		1.4		-2.3	**-2.3**	-1.5	**-1.5**	-2.2	**-2.2**
DDX17	DEAD (Asp-Glu-Ala-Asp) box polypeptide 17	1.4		1.4				7.2	**7.2**			-4.4	**-4.4**
DDX18	DEAD (Asp-Glu-Ala-Asp) box polypeptide 18	-1.5				-1.6		-1.3	**-1.3**	-1.7	**-1.7**	-2.6	**-2.6**
DDX21	DEAD (Asp-Glu-Ala-Asp) box polypeptide 21	-1.7	**-1.7**	-2.8	**-2.8**	-2.0		1.7	**1.7**	-2.1	**-2.1**	-3.9	**-3.9**
DDX23	DEAD (Asp-Glu-Ala-Asp) box polypeptide 23					-1.2						-1.9	
DDX39	DEAD (Asp-Glu-Ala-Asp) box polypeptide 39			-1.3	**-1.3**	-1.2		-1.3	**-1.3**			-1.2	
DDX3X	DEAD (Asp-Glu-Ala-Asp) box polypeptide 3, X-linked	-1.3		2.0	**2.0**	-1.4	**-1.4**	-1.8	**-1.8**			2.0	**2.0**
DDX42	DEAD (Asp-Glu-Ala-Asp) box polypeptide 42					-1.2		1.6	**1.6**	-1.3		-1.6	**-1.6**
DDX5	DEAD (Asp-Glu-Ala-Asp) box polypeptide 5											-1.2	
DDX52	DEAD (Asp-Glu-Ala-Asp) box polypeptide 52							1.7	**1.7**	-1.5	**-1.5**	-2.0	**-2.0**
DEAF1	deformed epidermal autoregulatory factor 1 (Drosophila)			-1.5	**-1.5**			1.2		-2.0	**-2.0**	-2.1	**-2.1**
DECR1	2,4-dienoyl CoA reductase 1, mitochondrial	1.5		1.5	**1.5**			1.6	**1.6**			5.2	**5.2**
DEDD	death effector domain containing									1.5	**1.5**	1.4	**1.4**
DEGS1	degenerative spermatocyte homolog 1, lipid desaturase (Drosophila)	1.6	**1.6**	1.7	**1.7**					1.7	**1.7**		
DEK	DEK oncogene (DNA binding)							-1.9	**-1.9**			1.4	**1.4**
DEXI	dexamethasone-induced transcript			1.2				-1.5	**-1.5**			1.6	**1.6**
DFFA	DNA fragmentation factor, 45kDa, alpha polypeptide			-1.2	**-1.2**	-1.2		1.6	**1.6**	-2.0	**-2.0**	-1.8	**-1.8**
DGAT1	diacylglycerol O-acyltransferase homolog 1 (mouse)	1.3		1.5	**1.5**			3.6	**3.6**			-1.8	**-1.8**
DGCR14	DiGeorge syndrome critical region gene 14									1.4		1.3	**1.3**
DGCR6	DiGeorge syndrome critical region gene 6									-1.7	**-1.7**	1.3	
DGKA	diacylglycerol kinase, alpha 80kDa	1.6	**1.6**	1.5		1.5	**1.5**					2.3	**2.3**
DGKD	diacylglycerol kinase, delta 130kDa			-1.2				-2.5	**-2.5**	1.3		3.2	**3.2**
DGKG	diacylglycerol kinase, gamma 90kDa					-4.6	**-4.6**	4.1	**4.1**			-2.6	**-2.6**
DGKZ	diacylglycerol kinase, zeta 104kDa							1.3		-1.5		5.4	**5.4**
DGUOK	deoxyguanosine kinase	-1.3	**-1.3**			-1.3	**-1.3**	-1.7		-1.3		1.4	**1.4**
DHCR24	24-dehydrocholesterol reductase	-1.5		-1.6	**-1.6**			-1.4				-1.3	
DHCR7	7-dehydrocholesterol reductase	-1.8		-2.8	**-2.8**	-1.5				-1.7	**-1.7**	-2.6	**-2.6**
DHFR	dihydrofolate reductase			-1.3		-1.3		-1.7	**-1.7**	-1.9	**-1.9**	-2.5	**-2.5**
DHODH	dihydroorotate dehydrogenase	-1.9		-2.4	**-2.4**	-2.0				-1.5	**-1.5**	-1.4	**-1.4**
DHPS	deoxyhypusine synthase	-1.2		-1.5	**-1.5**			-1.5	**-1.5**				
DHRS1	dehydrogenase/reductase (SDR family) member 1	1.7	**1.7**	1.6	**1.6**	1.4		2.3	**2.3**	1.5		-2.0	**-2.0**
DHRS7	dehydrogenase/reductase (SDR family) member 7	1.6		1.4	**1.4**			3.4	**3.4**	-1.2		-1.8	**-1.8**
DHX15	DEAH (Asp-Glu-Ala-His) box polypeptide 15			-1.3		-1.5		-1.3	**-1.3**	-1.6	**-1.6**	-1.4	**-1.4**
DHX16	DEAH (Asp-Glu-Ala-His) box polypeptide 16									-1.2	**-1.2**		
DHX30	DEAH (Asp-Glu-Ala-His) box polypeptide 30	-1.3				-1.3		-2.1	**-2.1**	-1.7	**-1.7**	1.8	**1.8**
DHX8	DEAH (Asp-Glu-Ala-His) box polypeptide 8			1.6	**1.6**					1.2			
DHX9	DEAH (Asp-Glu-Ala-His) box polypeptide 9			-1.3		-1.4		-1.4	**-1.4**	-1.7		2.3	**2.3**
DIA1	cytochrome b5 reductase 3							-1.2		1.3	**1.3**	1.6	**1.6**
DIAPH1	diaphanous homolog 1 (Drosophila)					-1.4	**-1.4**	-1.7	**-1.7**	-1.4	**-1.4**	-1.5	**-1.5**
DICER1	Dicer1, Dcr-1 homolog (Drosophila)			-1.4	**-1.4**			-1.6	**-1.6**	1.2		1.8	**1.8**
DIP	death-inducing-protein	1.5		2.4	**2.4**	1.8	**1.8**	-2.3	**-2.3**	-1.4	**-1.4**	3.5	**3.5**
DKC1	dyskeratosis congenita 1, dyskerin	-1.7	**-1.7**	-2.3	**-2.3**	-1.3	**-1.3**			-2.6	**-2.6**	-3.3	**-3.3**
DKFZP434C212	GTPase activating protein and VPS9 domains 1			-1.2		-1.3		1.2					

DLAT	dihydrolipoamide S-acetyltransferase (E2 component of pyruvate dehydrogenase complex)	-1.3				-1.5		-1.6	**-1.6**	-2.1	**-2.1**	-2.2	**-2.2**
DLD	dihydrolipoamide dehydrogenase					-1.2		-1.3		-1.3	**-1.3**		
DLEU1	deleted in lymphocytic leukemia, 1	-1.4		-1.6	**-1.6**			1.5	**1.5**	-1.8	**-1.8**	-2.0	**-2.0**
DLEU2	deleted in lymphocytic leukemia, 2												
DLG1	discs, large homolog 1 (Drosophila)	-1.3				1.3		-1.5	**-1.5**	-1.9	**-1.9**	1.8	**1.8**
DLG5	discs, large homolog 5 (Drosophila)					1.3	**1.3**	2.2	**2.2**	2.0	**2.0**	3.1	**3.1**
DLG7	discs, large homolog 7 (Drosophila)			1.4				-1.3	**-1.3**			1.8	**1.8**
DLGAP1	discs, large (Drosophila) homolog-associated protein 1							1.2		-1.3		-1.2	
DLST	dihydrolipoamide S-succinyltransferase (E2 component of 2-oxo-glutarate complex)							-1.2				1.5	**1.5**
DMPK	dystrophia myotonica-protein kinase	1.3		1.4								-1.2	
DMTF1	cyclin D binding myb-like transcription factor 1							-1.3		1.3		1.8	**1.8**
DMWD	dystrophia myotonica-containing WD repeat motif			1.3		1.4						-1.5	**-1.5**
DMXL1	Dmx-like 1	1.4						2.2	**2.2**	-2.7	**1.8**	1.6	**1.6**
DMXL2	Dmx-like 2	1.6	**1.6**			-1.3		1.7	**1.7**	-1.4		-1.2	**-1.2**
DNAJA1	DnaJ (Hsp40) homolog, subfamily A, member 1	-1.3	**-1.3**			-1.7	**-1.7**	-1.7	**-1.7**	-1.4	**-1.4**		
DNAJA2	DnaJ (Hsp40) homolog, subfamily A, member 2			-1.2		-1.4		15.8	**15.8**	1.2		-25.2	**-25.2**
DNAJB1	DnaJ (Hsp40) homolog, subfamily B, member 1					-1.4	**-1.4**	-1.9	**-1.9**	1.4		1.5	**1.5**
DNAJB6	DnaJ (Hsp40) homolog, subfamily B, member 6					-1.3		-1.7	**-1.7**	1.3		2.0	**2.0**
DNAJC11	DnaJ (Hsp40) homolog, subfamily C, member 11	-1.2	**-1.2**	-1.4	**-1.4**			1.4	**1.4**	1.9	**1.9**	-1.8	**-1.8**
DNAJC13	DnaJ (Hsp40) homolog, subfamily C, member 13							2.0	**2.0**			-1.7	
DNAJC3	DnaJ (Hsp40) homolog, subfamily C, member 3			1.6				1.7	**1.7**				
DNAJC7	DnaJ (Hsp40) homolog, subfamily C, member 7							1.3	**1.3**	-1.4	**-1.4**	-1.6	**-1.6**
DNCH1	dynein, cytoplasmic 1, heavy chain 1	1.3								4.0	**4.0**	1.8	**1.8**
DNCI2	dynein, cytoplasmic 1, intermediate chain 2							2.9	**2.9**	1.5	**1.4**	-1.5	**-1.5**
DNCL1	dynein, light chain, LC8-type 1												
DNM1	dynamin 1					1.3							
DNM1L	dynamin 1-like							1.5				-1.7	**1.2**
DNM2	dynamin 2					1.3	**1.3**					1.3	
DNMT1	DNA (cytosine-5-)-methyltransferase 1									-1.4	**-1.4**	-2.8	**-2.8**
DNPEP	aspartyl aminopeptidase	-1.2	**-1.2**	-1.3	**-1.3**	-1.4		-4.5	**-4.5**	-1.6	**-1.6**	1.6	**1.6**
DOC-1R	CDK2-associated protein 2							-2.0	**-2.0**	2.0	**2.0**	2.5	**2.5**
DOCK2	dedicator of cytokinesis 2							1.8	**1.8**			-2.8	**-2.8**
DOCK9	dedicator of cytokinesis 9			-1.3		-1.5				5.7	**5.7**		
DOK1	docking protein 1, 62kDa (downstream of tyrosine kinase 1)			1.7	**1.7**	1.3		2.3	**2.3**			-2.3	**-2.3**
DPAGT1	dolichyl-phosphate (UDP-N-acetylglucosamine) N-acetylglucosaminephosphotransferase 1 (GlcNAc-1-P transferase)									-1.8	**-1.8**	-1.6	**-1.6**
DPEP1	dipeptidase 1 (renal)	1.9	**1.9**			4.0	**4.0**			13.0	**13.0**	15.1	**15.1**
DPF2	D4, zinc and double PHD fingers family 2					1.5				1.3			
DPH2L1	DPH1 homolog (S. cerevisiae)					-1.3		-1.7	**-1.7**			1.6	**1.6**
DPM1	dolichyl-phosphate mannosyltransferase polypeptide 1, catalytic subunit					-1.2						-1.4	**-1.4**
DPM2	dolichyl-phosphate mannosyltransferase polypeptide 2, regulatory subunit							-1.2				-1.5	**-1.5**
DPT	dermatopontin			1.2		1.3		-22.5	**-22.5**			2.9	
DPYD	dihydropyrimidine dehydrogenase	1.2				-1.3		-8.8	**-8.8**			7.7	**7.7**
DPYSL2	dihydropyrimidinase-like 2			1.3		-1.3		-4.2	**-4.2**	3.1	**3.1**	3.7	**3.7**
DR1	down-regulator of transcription 1, TBP-binding (negative cofactor 2)			1.8	**1.6**			-1.6	**-1.6**	1.2			
DRAP1	DR1-associated protein 1 (negative cofactor 2 alpha)					-1.2		-1.5	**-1.5**	-2.2	**-2.2**		
DRG1	developmentally regulated GTP binding protein 1					-1.5	**-1.5**			-1.4	**-1.4**	-1.6	**-1.6**
DSCR1	Down syndrome critical region gene 1	5.0	**5.0**	8.2	**8.2**	4.8	**4.8**	1.6	**1.5**	7.7	**7.7**	7.9	**7.9**
DSG2	desmoglein 2					-1.3		86.2	**86.2**	-1.9		-43.8	**-43.8**
DST	dystonin			1.5		-1.2		-8.3	**-8.3**			15.8	**15.8**
DSTN	destrin (actin depolymerizing factor)			1.7	**1.7**			3.9	**3.9**			1.2	
DTX4	deltex 4 homolog (Drosophila)							-2.2	**-2.2**			3.3	**3.3**
DTYMK	deoxythymidylate kinase (thymidylate kinase)			-1.7		-1.3		-1.9	**-1.9**	-1.3		1.4	**1.2**
DULLARD	dullard homolog (Xenopus laevis)					-1.2		-1.4	**-1.4**	1.8	**1.8**	1.4	**1.4**
DUSP10	dual specificity phosphatase 10	2.7	**2.7**	7.3	**7.3**	1.4		-1.4	**-1.4**			1.9	**1.9**
DUSP11	dual specificity phosphatase 11 (RNA/RNP complex 1-interacting)									-1.3	**-1.3**	1.4	**1.4**
DUSP14	dual specificity phosphatase 14					-1.3				-1.3		-1.6	**-1.6**
DUSP3	dual specificity phosphatase 3 (vaccinia virus phosphatase VH1-related)			-1.6		-1.6		6.4	**6.4**			-17.5	**-17.5**
DUSP6	dual specificity phosphatase 6	1.4		1.8	**1.8**			15.8	**15.8**	1.7	**1.7**	-28.2	**-28.2**

DUSP7	dual specificity phosphatase 7	-2.0		-1.6		-2.0	**-2.0**			-1.2	**-1.2**	-1.4	**-1.4**
DUT	dUTP pyrophosphatase									-2.0	**-2.0**	-3.9	**-3.9**
DVL3	dishevelled, dsh homolog 3 (Drosophila)	1.6		1.3				-1.6	**-1.6**			1.9	**1.9**
DXYS155E	chromosome X and Y open reading frame 3	1.2				1.3		-1.4				1.8	**1.8**
DYRK1A	dual-specificity tyrosine-(Y)-phosphorylation regulated kinase 1A			1.3						-1.6	**-1.6**	-1.2	**-1.2**
DYRK2	dual-specificity tyrosine-(Y)-phosphorylation regulated kinase 2							-1.5		1.2		-1.4	**-1.4**
DYRK3	dual-specificity tyrosine-(Y)-phosphorylation regulated kinase 3			1.2		-1.5	**-1.5**						
DZIP3	zinc finger DAZ interacting protein 3					1.4		-1.3	**-1.3**	1.2		2.5	**2.5**
E2F1	E2F transcription factor 1			-1.3		-1.2	**-1.2**	-1.7	**-1.7**	-1.5		-2.7	**-2.7**
E2F3	E2F transcription factor 3	1.5		1.6				1.4		1.6	**1.6**		
E2F4	E2F transcription factor 4, p107/p130-binding												
E2F5	E2F transcription factor 5, p130-binding	-1.6	**-1.6**	-1.9	**-1.9**	-1.7				-1.8		-3.5	**-3.5**
E2F6	E2F transcription factor 6			-1.2		-1.3				-1.3	**-1.3**	-2.4	**-2.4**
EBAG9	estrogen receptor binding site associated, antigen, 9							-1.8	**-1.8**			2.5	**2.5**
EBNA1BP2	EBNA1 binding protein 2	-1.5	**-1.5**	-1.9	**-1.9**	-2.0				-1.8	**-1.8**	-2.6	**-2.6**
EBP	emopamil binding protein (sterol isomerase)	-1.2	**-1.2**	-1.5	**-1.5**			-2.1	**-2.1**	-1.5		1.4	
ECE1	endothelin converting enzyme 1	1.3						1.6				-1.7	
ECH1	enoyl Coenzyme A hydratase 1, peroxisomal											-1.6	**-1.6**
ECHS1	enoyl Coenzyme A hydratase, short chain, 1, mitochondrial			-1.4	**-1.4**	-1.3				-1.4	**-1.4**	-1.6	**-1.6**
EDD1	E3 ubiquitin protein ligase, HECT domain containing, 1	1.3	**1.3**	1.2		-1.4	**-1.4**	-1.4	**-1.4**	1.4	**1.4**	1.9	**1.9**
EDEM1	ER degradation enhancer, mannosidase alpha-like 1	-1.2						-1.6	**-1.5**	-1.2	**-1.2**	1.4	**1.4**
EDG6	endothelial differentiation, lysophosphatidic acid G-protein-coupled receptor, 6					1.4				1.8	**1.8**	-1.2	
EED	embryonic ectoderm development					-1.4		-1.9	**-1.9**			1.2	**1.2**
EEF1A1	eukaryotic translation elongation factor 1 alpha 1			-1.6		1.3		4.9	**4.9**			-5.1	**-5.1**
EEF1A2	eukaryotic translation elongation factor 1 alpha 2												
EEF1B2	eukaryotic translation elongation factor 1 beta 2					-1.2							
EEF1D	eukaryotic translation elongation factor 1 delta (guanine nucleotide exchange protein)					-1.2		1.6	**1.6**	1.2		-1.3	**-1.3**
EEF1E1	eukaryotic translation elongation factor 1 epsilon 1	-1.7	**-1.7**	-2.1	**-2.1**	-2.3		-1.6	**-1.6**	-3.3	**-3.3**	-2.4	**-2.4**
EEF1G	eukaryotic translation elongation factor 1 gamma							-1.4		-1.5		1.3	
EEF2	eukaryotic translation elongation factor 2							1.3	**1.3**				
EFNA3	ephrin-A3					2.2							
EFNB2	ephrin-B2							-5.9	**-5.9**	-2.8	**-2.8**	5.8	**5.8**
EHBP1	EH domain binding protein 1							1.8	**1.4**	1.2		-2.6	**-2.6**
EHD1	EH-domain containing 1			-1.2	**-1.2**			1.6	**1.6**			-1.4	**-1.4**
EI24	etoposide induced 2.4 mRNA			-1.3				1.3	**1.3**	-1.5	**-1.5**	-2.1	**-2.1**
EIF1	eukaryotic translation initiation factor 1	-1.4	**-1.4**	-1.4	**-1.4**	-1.7	**-1.7**					1.3	**1.3**
EIF1AX	eukaryotic translation initiation factor 1A, X-linked			-1.6		-1.4		-2.2	**-2.2**	-1.5	**-1.5**	-2.8	**-2.8**
EIF2A	eukaryotic translation initiation factor 2A, 65kDa	1.2						1.3	**1.3**			1.2	**1.2**
EIF2AK2	eukaryotic translation initiation factor 2-alpha kinase 2	-1.3						-1.2		-1.5	**-1.5**		
EIF2B2	eukaryotic translation initiation factor 2B, subunit 2 beta, 39kDa					-1.2						-1.2	**-1.2**
EIF2B4	eukaryotic translation initiation factor 2B, subunit 4 delta, 67kDa											-1.4	**-1.4**
EIF2B5	eukaryotic translation initiation factor 2B, subunit 5 epsilon, 82kDa									-1.2		-1.5	**-1.5**
EIF2C2	eukaryotic translation initiation factor 2C, 2	-2.1				-1.9		2.6	**2.6**	-1.6	**-1.6**	-4.3	**-4.3**
EIF2S1	eukaryotic translation initiation factor 2, subunit 1 alpha, 35kDa	-1.4	**-1.4**	-1.2		-1.4	**-1.4**	-1.2	**-1.2**	-1.6	**-1.6**	-1.8	**-1.8**
EIF2S2	eukaryotic translation initiation factor 2, subunit 2 beta, 38kDa	-1.3										-1.6	**-1.6**
EIF2S3	eukaryotic translation initiation factor 2, subunit 3 gamma, 52kDa			1.7				1.3	**1.3**				
EIF3S10	eukaryotic translation initiation factor 3, subunit 10 theta, 150/170kDa	-1.4				-1.7	**-1.7**	3.9	**3.9**	-1.3	**-1.3**	-5.4	**-5.4**
EIF3S12	eukaryotic translation initiation factor 3, subunit 12			-1.3		-1.2		-1.3		-1.5	**-1.3**	-1.2	
EIF3S2	eukaryotic translation initiation factor 3, subunit 2 beta, 36kDa					-1.5	**-1.5**			-1.6	**-1.6**	-1.6	**-1.6**
EIF3S3	eukaryotic translation initiation factor 3, subunit 3 gamma, 40kDa					-1.2						1.4	**1.4**
EIF3S4	eukaryotic translation initiation factor 3, subunit 4 delta, 44kDa												
EIF3S5	eukaryotic translation initiation factor 3, subunit 5 epsilon, 47kDa			1.3	**1.3**					1.2	**1.2**	1.3	**1.3**
EIF3S6	eukaryotic translation initiation factor 3, subunit 6 48kDa					-1.2		-1.5	**-1.5**	-1.6	**-1.6**	1.2	
EIF3S7	eukaryotic translation initiation factor 3, subunit 7 zeta, 66/67kDa	-1.2				-1.6		1.3	**1.3**			-1.3	**-1.3**
EIF3S8	eukaryotic translation initiation factor 3, subunit 8, 110kDa	-1.2	**-1.2**			-1.2		-3.1	**-3.1**	-1.6	**-1.4**	3.4	**3.4**
EIF3S9	eukaryotic translation initiation factor 3, subunit 9 eta, 116kDa	-1.5	**-1.5**	-2.2	**-2.2**	-1.3		-1.2	**-1.2**	-2.0	**-2.0**	-2.2	**-2.2**
EIF4A1	eukaryotic translation initiation factor 4A, isoform 1	-1.7	**-1.4**	-2.0	**-2.0**	-1.3				-1.8	**-1.8**		
EIF4A2	eukaryotic translation initiation factor 4A, isoform 2							1.4	**1.4**			-1.3	

EIF4B	eukaryotic translation initiation factor 4B			1.3				1.8	**1.8**	-2.4	**-2.4**	-1.8	**-1.8**
EIF4E	eukaryotic translation initiation factor 4E			-1.3		-1.4		-1.3	**-1.3**	1.3		-1.4	**-1.4**
EIF4E2	eukaryotic translation initiation factor 4E family member 2							-1.6	**-1.6**	1.2	**1.2**	1.5	**1.5**
EIF4EBP1	eukaryotic translation initiation factor 4E binding protein 1			-1.9	**-1.9**	-1.6				-3.1	**-3.1**	-5.7	**-5.7**
EIF4EBP2	eukaryotic translation initiation factor 4E binding protein 2			1.2						-1.3	**-1.3**	-1.3	**-1.3**
EIF4G1	eukaryotic translation initiation factor 4 gamma, 1	-1.5		-1.5	**-1.5**	-1.4	**-1.4**					-1.4	**-1.4**
EIF4G2	eukaryotic translation initiation factor 4 gamma, 2												
EIF4G3	eukaryotic translation initiation factor 4 gamma, 3					-1.5		1.8		-1.2	**-1.2**	-1.6	**-1.6**
EIF5	eukaryotic translation initiation factor 5	-1.3		-1.5	**-1.5**	-1.3		1.2	**1.2**	-1.2		-1.8	**-1.8**
EIF5A	eukaryotic translation initiation factor 5A					-1.2		-10.2	**-10.2**	-2.0	**-2.0**	3.5	**3.5**
EIF5B	eukaryotic translation initiation factor 5B	1.6		-1.7	**-1.7**	-1.2		2.5	**2.5**	-1.6	**-1.6**	-4.3	**-4.3**
ELAC2	elaC homolog 2 (E. coli)	-1.4		-1.3						1.4		1.2	
ELAVL3	ELAV (embryonic lethal, abnormal vision, Drosophila)-like 3 (Hu antigen C)					1.6							
ELF1	E74-like factor 1 (ets domain transcription factor)			1.6	**1.6**			-1.8	**-1.8**	1.4	**1.4**	2.7	**2.7**
ELF2	E74-like factor 2 (ets domain transcription factor)			1.3				-1.8	**-1.8**			1.4	**1.4**
ELK1	ELK1, member of ETS oncogene family					1.3		-1.2				1.5	**1.4**
ELK3	ELK3, ETS-domain protein (SRF accessory protein 2)							5.2	**5.2**			-2.4	**-2.4**
ELMO1	engulfment and cell motility 1	-1.3						3.6	**3.6**	2.1	**2.1**		
ELOVL5	ELOVL family member 5, elongation of long chain fatty acids (FEN1/Elo2, SUR4/Elo3-like, yeast)			1.4	**1.4**			-1.6	**-1.6**	-2.0	**1.3**	1.8	**1.8**
ELOVL6	ELOVL family member 6, elongation of long chain fatty acids (FEN1/Elo2, SUR4/Elo3-like, yeast)	-1.3				1.4		-3.4	**-3.4**	-3.8	**-3.8**	-1.8	**-1.8**
EMD	emerin (Emery-Dreifuss muscular dystrophy)											-1.4	
EMP3	epithelial membrane protein 3			-1.3									
ENO1	enolase 1, (alpha)	-1.3	**-1.3**	-1.6	**-1.6**	-1.5				-1.5	**-1.5**	-2.6	**-2.6**
ENO2	enolase 2 (gamma, neuronal)	-1.2		-1.4		-1.5							
ENTH	clathrin interactor 1			1.3				2.5	**2.5**	1.9	**1.9**	-1.5	**-1.5**
ENTPD4	ectonucleoside triphosphate diphosphohydrolase 4	1.3		1.4		-1.4		1.5	**1.5**	1.3	**1.3**		
ENTPD6	ectonucleoside triphosphate diphosphohydrolase 6 (putative function)	-1.2		-1.4	**-1.4**							-2.0	**-2.0**
EP300	E1A binding protein p300					-1.3						1.4	**1.4**
EP400	E1A binding protein p400							1.3	**1.3**	1.4	**1.4**	-1.6	
EPAS1	endothelial PAS domain protein 1	-1.3								15.9	**15.9**	70.9	**70.9**
EPB41	erythrocyte membrane protein band 4.1 (elliptocytosis 1, RH-linked)			1.5		1.3		-2.0		-2.3		-1.7	**-1.7**
EPB41L2	erythrocyte membrane protein band 4.1-like 2			1.2				1.8	**1.8**			-1.9	**-1.9**
EPHA1	EPH receptor A1	1.7	**1.7**										
EPHB6	EPH receptor B6	-1.4		-1.9	**-1.9**	-1.8	**-1.8**						
EPIM	syntaxin 2			-1.2		1.3		1.3	**1.3**	1.5			
EPM2A	epilepsy, progressive myoclonus type 2A, Lafora disease (laforin)							-2.2	**-2.2**			1.6	
EPOR	erythropoietin receptor			1.2		1.7		16.6	**16.6**			-6.1	**-6.1**
EPRS	glutamyl-prolyl-tRNA synthetase							1.3	**1.3**	-1.7	**-1.7**	-2.4	**-2.4**
EPS15	epidermal growth factor receptor pathway substrate 15	1.3	**1.3**	1.4	**1.4**			1.6	**1.6**	1.8	**1.8**	1.5	**1.5**
ERAL1	Era G-protein-like 1 (E. coli)												
ERCC1	excision repair cross-complementing rodent repair deficiency, complementation group 1 (includes overlapping antisense sequence)	-1.3	**-1.3**	-1.4	**-1.3**	-1.3		-1.8	**-1.8**	1.3	**1.3**		
ERCC2	excision repair cross-complementing rodent repair deficiency, complementation group 2 (xeroderma pigmentosum D)	1.2											
ERCC3	excision repair cross-complementing rodent repair deficiency, complementation group 3 (xeroderma pigmentosum group B complementing)			1.2						-1.3	**-1.3**		
ERCC4	excision repair cross-complementing rodent repair deficiency, complementation group 4	1.5		1.2				-1.3				1.2	
ERCC5	excision repair cross-complementing rodent repair deficiency, complementation group 5 (xeroderma pigmentosum, complementation group G (Cockayne syndrome))	1.6		1.6	**1.3**			-1.4	**-1.4**	-1.3		1.8	**1.8**
ERCC8	excision repair cross-complementing rodent repair deficiency, complementation group 8			1.3	**1.3**					-1.8	**-1.8**	-1.8	**-1.4**
ERF	Ets2 repressor factor			1.3		1.4		2.1	**2.1**	-1.5	**-1.5**	-2.5	**-2.5**
ERG	v-ets erythroblastosis virus E26 oncogene homolog (avian)					-1.4		-2.7	**-2.7**	1.8	**1.8**	2.1	**2.1**
ERH	enhancer of rudimentary homolog (Drosophila)					-1.2		-1.2	**-1.2**				
ERP29	endoplasmic reticulum protein 29			-1.3				1.4	**1.4**	-1.3	**-1.3**	-1.8	**-1.8**
ESD	esterase D/formylglutathione hydrolase					-1.5		2.3	**2.3**	1.7	**1.7**	-1.9	**-1.9**
ESPL1	extra spindle pole bodies homolog 1 (S. cerevisiae)	1.3	**1.3**			1.2		-1.2		1.3	**1.3**	1.2	
ESRRA	estrogen-related receptor alpha	-1.3		-1.3				1.4	**1.4**			-1.3	**-1.3**
EST1B	Smg-5 homolog, nonsense mediated mRNA decay factor (C. elegans)			-1.7	**-1.7**							-1.4	
ETF1	eukaryotic translation termination factor 1					-1.3		1.3	**1.3**	-1.6	**-1.6**	-2.4	**-2.4**
ETFA	electron-transfer-flavoprotein, alpha polypeptide (glutaric aciduria II)							-1.3	**-1.3**				
ETFB	electron-transfer-flavoprotein, beta polypeptide			1.3	**1.3**					2.1	**2.1**	-1.7	**-1.7**
ETFDH	electron-transferring-flavoprotein dehydrogenase			1.3		-1.2							

ETHE1	ethylmalonic encephalopathy 1	1.3		1.4	**1.4**			1.6	**1.6**	-1.3	**-1.3**	-1.6	**-1.6**
ETS1	v-ets erythroblastosis virus E26 oncogene homolog 1 (avian)			2.4	**2.4**			-29.1	**-29.1**			26.5	**26.5**
ETS2	v-ets erythroblastosis virus E26 oncogene homolog 2 (avian)	-1.6	**-1.3**			-1.7	**-1.7**	-1.5	**-1.5**	5.1	**5.1**	4.1	**4.1**
ETV5	ets variant gene 5 (ets-related molecule)	-1.3				-10.4	**-10.4**			1.9	**1.9**		
ETV6	ets variant gene 6 (TEL oncogene)	-1.4		-1.3		-1.6		2.5	**2.5**	-1.6	**-1.6**	-3.1	**-3.1**
EVER1	transmembrane channel-like 6							1.3		1.6	**1.6**	-2.4	**-2.4**
EWSR1	Ewing sarcoma breakpoint region 1							-1.3		-1.5		-1.3	**-1.3**
EXO1	exonuclease 1							-1.6	**-1.6**	-2.1	**-2.1**	-4.6	**-4.6**
EXOSC10	exosome component 10												
EXOSC2	exosome component 2	-1.7	**-1.7**	-1.7	**-1.7**	-1.2		-1.2		-2.4	**-2.4**	-2.6	**-2.6**
EXOSC7	exosome component 7	-1.8	**-1.8**	-2.3	**-2.3**	-1.7						-2.1	**-2.1**
EXOSC8	exosome component 8	-1.3		-1.6	**-1.6**	-1.4		-2.4	**-2.4**				
EXT1	exostoses (multiple) 1	1.3				-1.3				-1.8	**-1.8**	-2.5	**-2.5**
EXTL2	exostoses (multiple)-like 2	1.2				1.2		1.5	**1.5**	-2.0	**-2.0**	-1.9	**-1.9**
EXTL3	exostoses (multiple)-like 3												
EZH1	enhancer of zeste homolog 1 (Drosophila)			1.3				1.3	**1.3**	-1.4	**-1.4**	2.0	**2.0**
EZH2	enhancer of zeste homolog 2 (Drosophila)					-1.3		-1.6	**-1.6**	-1.3		-1.3	**-1.3**
F2RL3	coagulation factor II (thrombin) receptor-like 3									2.1	**2.1**		
F8A1	coagulation factor VIII-associated (intronic transcript) 1	1.2	**1.2**					1.6	**1.6**			-2.1	**-2.1**
FABP5	fatty acid binding protein 5 (psoriasis-associated)	-1.2		-1.3				-2.2	**-2.2**	-4.4	**-4.4**	-2.5	**-2.5**
FADD	Fas (TNFRSF6)-associated via death domain					-1.9	**-1.9**					-1.3	**-1.3**
FADS1	fatty acid desaturase 1	-1.5		-4.1	**-4.1**	-1.9	**-1.9**	-1.5	**-1.5**	-1.5	**-1.5**	-1.8	**-1.8**
FADS2	fatty acid desaturase 2	-1.4		-1.7	**-1.7**	-1.3		-1.9	**-1.9**	-2.0	**-2.0**		
FADS3	fatty acid desaturase 3	-1.2	**-1.2**					-3.1	**-3.1**	-1.3		5.6	**5.6**
FAH	fumarylacetoacetate hydrolase (fumarylacetoacetase)	-1.5	**-1.5**					1.6	**1.6**	-1.9		-2.8	**-2.8**
FALZ	bromodomain PHD finger transcription factor	1.3						1.6	**1.6**	1.6	**-1.5**	-1.5	**-1.3**
FAM38A	family with sequence similarity 38, member A	-1.3		-1.3	**-1.3**			2.6	**2.6**	2.6	**2.6**	1.8	**1.8**
FAM3C	family with sequence similarity 3, member C			-1.3		-1.4		-2.2	**-2.2**	2.0	**2.0**	12.1	**12.1**
FAM89B	family with sequence similarity 89, member B					1.2		-2.5	**-2.5**			5.2	**5.2**
FANCG	Fanconi anemia, complementation group G	1.2								-1.3	**-1.3**	-2.0	**-2.0**
FANCL	Fanconi anemia, complementation group L			1.2				1.6	**1.6**			-2.8	**-2.8**
FARSLA	phenylalanine-tRNA synthetase-like, alpha subunit	-1.9	**-1.9**	-2.1	**-2.1**	-2.0	**-2.0**	1.6	**1.6**	-2.1	**-2.1**	-4.1	**-4.1**
FAS	Fas (TNF receptor superfamily, member 6)			1.5								1.6	
FASN	fatty acid synthase	-1.6	**-1.6**	-2.8	**-2.8**			-1.4		-3.2	**-3.2**	-2.2	**-2.2**
FASTK	Fas-activated serine/threonine kinase												
FAT	FAT tumor suppressor homolog 1 (Drosophila)	1.8	**1.8**	2.3	**2.3**	1.6							
FAU	Finkel-Biskis-Reilly murine sarcoma virus (FBR-MuSV) ubiquitously expressed (fox derived); ribosomal protein S30												
FBL	fibrillarin	-1.4	**-1.4**	-1.4	**-1.4**	-1.4	**-1.4**	1.4	**1.4**	-1.2	**-1.2**	-2.1	**-2.1**
FBLN2	fibulin 2	1.4	**1.4**	1.6	**1.6**	3.5	**3.5**						
FBN1	fibrillin 1	1.6		1.2		1.2	**1.2**						
FBXO7	F-box protein 7									1.7		1.3	**1.3**
FBXO9	F-box protein 9							2.2	**2.2**	-1.4	**-1.4**	-1.9	**-1.9**
FBXW11	F-box and WD-40 domain protein 11					-1.4		1.3	**1.3**	1.3	**1.3**		
FCGR2A	Fc fragment of IgG, low affinity IIa, receptor (CD32)					1.3		-2.0	**-2.0**				
FCGR2B	Fc fragment of IgG, low affinity IIb, receptor (CD32)												
FCHSD2	FCH and double SH3 domains 2							1.9	**1.9**	3.0	**3.0**	4.0	**4.0**
FDFT1	farnesyl-diphosphate farnesyltransferase 1			-1.8	**-1.8**	-1.4		1.5	**1.4**	-1.4		-1.3	**-1.2**
FDPS	farnesyl diphosphate synthase (farnesyl pyrophosphate synthetase, dimethylallyltranstransferase, geranyltranstransferase)	-1.3		-1.5	**-1.5**	-1.5		-2.3	**-2.3**			1.6	**1.6**
FDXR	ferredoxin reductase									-1.4			
FECH	ferrochelatase (protoporphyria)			2.0	**2.0**	-1.3						-1.8	**-1.8**
FEN1	flap structure-specific endonuclease 1					-1.4		-1.3	**-1.3**	-1.8	**-1.8**	-2.9	**-2.9**
FES	feline sarcoma oncogene												
FEZ2	fasciculation and elongation protein zeta 2 (zygin II)	1.2						1.5	**1.5**	1.7		1.6	**1.6**
FGF9	fibroblast growth factor 9 (glia-activating factor)	1.4		1.3				-45.7	**-45.7**	-2.1	**-2.1**	39.2	**39.2**
FGFR1	fibroblast growth factor receptor 1 (fms-related tyrosine kinase 2, Pfeiffer syndrome)	1.6	**1.6**	3.1	**3.1**	1.4		7.4	**7.4**			-1.6	**-1.6**
FGFR1OP	FGFR1 oncogene partner							1.5	**1.5**			-2.7	**-2.7**
FH	fumarate hydratase	-1.2		-1.4	**-1.4**	-1.2		-1.4	**-1.4**	-2.1	**-2.1**	-2.6	**-2.6**
FHL1	four and a half LIM domains 1	3.3	**3.3**	6.6	**6.6**	13.7	**13.7**	-2.1	**-2.1**	1.8	**1.8**	9.6	**9.6**

FIBP	fibroblast growth factor (acidic) intracellular binding protein							-1.8	**-1.8**	-1.3	**-1.3**		
FKBP1A	FK506 binding protein 1A, 12kDa					1.3	**1.3**	1.5	**1.5**	-1.3	**-1.3**	-1.7	**-1.7**
FKBP2	FK506 binding protein 2, 13kDa					-1.3				1.3			
FKBP4	FK506 binding protein 4, 59kDa	-1.9		-2.1	**-2.1**	-1.3		3.0	**3.0**			-8.6	**-8.6**
FKBP5	FK506 binding protein 5	6.6	**6.6**	11.2	**11.2**	4.4	**4.4**	7.0	**7.0**	21.4	**21.4**	13.7	**13.7**
FKBP8	FK506 binding protein 8, 38kDa											1.2	
FLI1	Friend leukemia virus integration 1	1.9		2.1	**2.1**	1.4				1.7	**1.6**	1.9	**1.9**
FLII	flightless I homolog (Drosophila)			1.3		-1.3		-1.4	**-1.4**	1.3		1.8	**1.8**
FLJ12443	acyltransferase like 2					1.5		-1.6	**-1.6**			-1.3	
FLJ30092	AF-1 specific protein phosphatase	1.3		1.3						1.6	**1.6**	2.8	**2.8**
FLN29	TRAF-type zinc finger domain containing 1					1.4		1.5		1.4		1.4	**1.4**
FLNA	filamin A, alpha (actin binding protein 280)			-1.6				-2.2	**-2.2**	-1.3		3.1	**3.1**
FLNB	filamin B, beta (actin binding protein 278)			-1.2	**-1.2**			1.6	**1.6**			1.4	
FLT1	fms-related tyrosine kinase 1 (vascular endothelial growth factor/vascular permeability factor receptor)	1.4				3.8	**3.8**			2.6	**2.6**	1.5	
FLT3LG	fms-related tyrosine kinase 3 ligand			-1.8	**-1.8**								
FMO5	flavin containing monooxygenase 5					9.7		4.8	**4.8**	1.9	**1.9**	121.2	**121.2**
FMR1	fragile X mental retardation 1			1.2				-2.3	**-2.3**	1.4	**1.4**	2.8	**2.8**
FNBP1	formin binding protein 1	1.4		1.2				-17.0	**-17.0**	-2.9	**-2.9**	13.3	**13.3**
FNBP1L	formin binding protein 1-like	1.9	**1.9**	1.5		1.8		1.4	**1.4**	3.0	**3.0**	3.5	**3.5**
FNBP3	PRP40 pre-mRNA processing factor 40 homolog A (yeast)			1.6		-1.5		2.2	**2.2**	-2.1	**-2.1**	-2.4	**-2.4**
FNBP4	formin binding protein 4	-1.2						-1.4	**-1.4**	-1.6	**-1.6**		
FNTA	farnesyltransferase, CAAX box, alpha					-1.3		1.5	**1.4**			-1.2	**-1.2**
FNTB	farnesyltransferase, CAAX box, beta	-1.4											
FOS	v-fos FBJ murine osteosarcoma viral oncogene homolog			1.4									
FOSL2	FOS-like antigen 2					1.4	**1.4**	7.1	**7.1**	4.0	**4.0**	-1.9	**-1.9**
FOXJ2	forkhead box J2							-2.0	**-2.0**	-1.3		2.0	**2.0**
FOXK2	forkhead box K2	-1.3		-1.4		-1.4	**-1.4**	1.8	**1.8**	1.4		-1.8	**-1.8**
FOXM1	forkhead box M1	1.2						-3.5	**-3.5**	1.4	**1.4**	2.2	**2.2**
FOXO3A	forkhead box O3A	1.3		1.4		1.2		1.9	**1.7**	2.7	**2.7**	2.3	**2.3**
FPGT	fucose-1-phosphate guanylyltransferase			1.5		-1.2				-1.2	**-1.2**	-1.2	
FPRL1	formyl peptide receptor-like 1					1.3							
FRAP1	FK506 binding protein 12-rapamycin associated protein 1			1.2									
FRG1	FSHD region gene 1			-1.4						1.2		-1.4	**-1.4**
FRYL	FRY-like												
FSCN1	fascin homolog 1, actin-bundling protein (Strongylocentrotus purpuratus)	-1.5	**-1.5**	-2.1	**-2.1**	-1.3		2.0	**1.7**	1.6	**1.6**	-3.9	**-3.9**
FSHB	follicle stimulating hormone, beta polypeptide	1.4		1.6	**1.6**								
FSHR	follicle stimulating hormone receptor	1.5		-1.3		1.4							
FTH1	ferritin, heavy polypeptide 1	-1.2		-1.3	**-1.3**	-1.5		-2.0	**-2.0**	1.5	**1.5**	2.9	**2.9**
FTL	ferritin, light polypeptide			-1.2	**-1.2**			1.9	**1.9**	-1.4	**-1.4**	-2.3	**-2.3**
FTSJ1	FtsJ homolog 1 (E. coli)			-1.2		-1.7		1.2	**1.2**	-1.3	**-1.3**	-2.4	**-2.4**
FUBP1	far upstream element (FUSE) binding protein 1	1.3		-1.4		-1.7		1.7	**1.7**	-1.8	**-1.8**	-1.9	**-1.9**
FUBP3	far upstream element (FUSE) binding protein 3	-1.4				-1.4		1.2		1.4			
FUS	fusion (involved in t(12;16) in malignant liposarcoma)			-1.3	**-1.3**			-2.6	**-2.6**	-1.5	**-1.5**	1.5	**1.5**
FUSIP1	FUS interacting protein (serine/arginine-rich) 1			1.3	**1.3**			-1.5	**-1.5**	-1.9	**-1.9**	-1.2	
FUT4	fucosyltransferase 4 (alpha (1,3) fucosyltransferase, myeloid-specific)							1.3	**1.3**	1.7	**1.7**		
FUT7	fucosyltransferase 7 (alpha (1,3) fucosyltransferase)									-1.8	**-1.8**	54.2	**54.2**
FUT8	fucosyltransferase 8 (alpha (1,6) fucosyltransferase)	1.2		1.3	**1.3**			-1.4	**-1.4**				
FXR1	fragile X mental retardation, autosomal homolog 1	1.3		-1.2				1.4	**1.4**	1.4	**1.4**	1.4	**1.4**
FXR2	fragile X mental retardation, autosomal homolog 2									1.4	**1.4**	1.6	**1.6**
FXYD2	FXYD domain containing ion transport regulator 2	1.4											
FYB	FYN binding protein (FYB-120/130)			1.3				11.8	**11.8**			-7.9	**-7.9**
FYN	FYN oncogene related to SRC, FGR, YES	1.2	**1.2**	1.4	**1.4**	-1.2		-8.6	**-8.6**	1.4		2.0	**2.0**
FZD6	frizzled homolog 6 (Drosophila)	1.5	**1.5**	1.9	**1.9**	1.3		10.1	**10.1**	2.1	**2.1**	-2.9	**-2.9**
FZR1	fizzy/cell division cycle 20 related 1 (Drosophila)					1.4		-1.9	**-1.9**	1.5		2.3	**2.3**
G1P2	ISG15 ubiquitin-like modifier			1.2		-1.3		-3.1	**-3.1**			2.0	**2.0**
G22P1	X-ray repair complementing defective repair in Chinese hamster cells 6 (Ku autoantigen, 70kDa)							-1.4		-1.4	**-1.4**	-1.6	**-1.6**
G3BP	GTPase activating protein (SH3 domain) binding protein 1	-1.3				-1.6		1.5	**1.4**	-2.2	**-2.2**	-4.3	**-4.3**
G3BP2	GTPase activating protein (SH3 domain) binding protein 2							1.3	**1.3**			-1.7	**-1.7**

GAB1	GRB2-associated binding protein 1			2.1				-2.4	**-2.4**	2.0	**2.0**	7.5	**7.5**
GABARAP	GABA(A) receptor-associated protein	1.2				1.3		-1.3	**-1.3**			2.1	**2.1**
GABARAPL2	GABA(A) receptor-associated protein-like 2									1.2		1.7	**1.7**
GABPB2	GA binding protein transcription factor, beta subunit 2	-1.2		1.9		-1.4		-1.7	**-1.7**	1.5	**1.5**	1.8	**1.8**
GABRE	gamma-aminobutyric acid (GABA) A receptor, epsilon			1.4									
GADD45B	growth arrest and DNA-damage-inducible, beta			-1.2		2.0		1.3	**1.3**	1.5	**1.5**	1.4	**1.4**
GAK	cyclin G associated kinase							1.4	**1.4**	1.2		-1.4	
GALC	galactosylceramidase	1.5		1.6	**1.6**			-2.1	**-2.1**			1.5	**1.5**
GALK2 (includ	galactokinase 2	1.3						1.3	**1.3**			-1.7	**-1.7**
GALNT1	UDP-N-acetyl-alpha-D-galactosamine:polypeptide N-acetylgalactosaminyltransferase 1 (GalNAc-T1)					-1.3		2.0	**2.0**			-2.2	**-2.2**
GALNT10	UDP-N-acetyl-alpha-D-galactosamine:polypeptide N-acetylgalactosaminyltransferase 10 (GalNAc-T10)									1.3		2.2	**2.2**
GALNT2	UDP-N-acetyl-alpha-D-galactosamine:polypeptide N-acetylgalactosaminyltransferase 2 (GalNAc-T2)	-1.4		-1.2				-2.2	**-2.2**	1.4	**1.4**	1.9	**1.9**
GAMT	guanidinoacetate N-methyltransferase			-2.2	**-2.2**					-2.2	**-2.2**	-2.9	**-2.9**
GANAB	glucosidase, alpha; neutral AB									-1.3	**-1.3**	-1.2	
GAPD	glyceraldehyde-3-phosphate dehydrogenase	-1.3		-1.5		1.3		-1.2	**-1.2**			1.3	**1.3**
GAPDHS	glyceraldehyde-3-phosphate dehydrogenase, spermatogenic							1.8	**1.8**			-2.4	**-2.4**
GARNL1	GTPase activating Rap/RanGAP domain-like 1					-1.5				1.6	**1.6**	1.7	**1.7**
GARS	glycyl-tRNA synthetase	-1.4	**-1.4**	-1.7	**-1.7**	-1.7	**-1.7**	1.9	**1.9**	-2.1	**-2.1**	-4.3	**-4.3**
GART	phosphoribosylglycinamide formyltransferase, phosphoribosylglycinamide synthetase, phosphoribosylaminoimidazole synthetase	-1.6	**-1.6**	-1.6	**-1.6**	-1.6		1.4	**1.4**	-2.4	**-2.4**	-4.2	**-4.2**
GAS2L1	growth arrest-specific 2 like 1					1.8				15.6	**15.6**	17.6	**17.6**
GAS7	growth arrest-specific 7			-1.3				-10.7	**-10.7**	1.5	**1.5**	7.5	**7.5**
GATA2	GATA binding protein 2					1.4		3.6	**3.6**			-4.0	**-4.0**
GATA3	GATA binding protein 3			1.4	**1.4**			-3.1	**-3.1**			3.3	**3.3**
GBAS	glioblastoma amplified sequence					-1.5		4.0	**4.0**			-3.2	**-3.2**
GBE1	glucan (1,4-alpha-), branching enzyme 1 (glycogen branching enzyme, Andersen disease, glycogen storage disease type IV)					-1.3				1.3	**1.3**	1.4	**1.4**
GBF1	golgi-specific brefeldin A resistance factor 1	1.2						1.2				1.6	**1.6**
GBP1	guanylate binding protein 1, interferon-inducible, 67kDa	1.2						-26.9	**-26.9**	-5.8	**-5.8**	49.3	**49.3**
GCAT	glycine C-acetyltransferase (2-amino-3-ketobutyrate coenzyme A ligase)			-1.4	**-1.4**			9.1	**9.1**	-1.4	**-1.4**	-10.8	**-10.8**
GCDH	glutaryl-Coenzyme A dehydrogenase	-1.3	**-1.3**	-1.3	**-1.3**					-1.5		-1.3	
GCH1	GTP cyclohydrolase 1 (dopa-responsive dystonia)			-1.3	**-1.3**			-1.7	**-1.7**	-1.7	**-1.7**	1.5	**1.5**
GCHFR	GTP cyclohydrolase I feedback regulator									-1.8	**-1.8**	10.3	**10.3**
GCLC	glutamate-cysteine ligase, catalytic subunit					-1.2		2.0	**2.0**	1.7	**1.7**	-1.3	**-1.3**
GCLM	glutamate-cysteine ligase, modifier subunit							-2.5	**-2.5**	2.4	**2.4**	3.6	**3.6**
GCN1L1	GCN1 general control of amino-acid synthesis 1-like 1 (yeast)			-1.3	**-1.3**			1.3	**1.3**	-1.4	**-1.4**	-1.5	**-1.5**
GCN5L2	GCN5 general control of amino-acid synthesis 5-like 2 (yeast)	-1.4	**-1.4**	-1.5	**-1.5**			-1.4		-1.3	**-1.3**	-1.3	
GCS1	glucosidase I									-1.3	**-1.3**	-1.7	**-1.7**
GCSH	glycine cleavage system protein H (aminomethyl carrier)	-1.6	**-1.6**	-1.5	**-1.5**	-1.7		2.1	**2.1**	-3.0	**-3.0**	-4.6	**-4.6**
GDF10	growth differentiation factor 10					-2.8	**-2.8**						
GDI1	GDP dissociation inhibitor 1					1.5		-1.6	**-1.6**	1.4	**1.4**	2.4	**2.4**
GDI2	GDP dissociation inhibitor 2					-1.3		1.7	**1.7**	1.4	**1.4**		
GEMIN4	gem (nuclear organelle) associated protein 4	-1.3		-1.5	**-1.5**	-1.5	**-1.5**	-1.6	**-1.6**			-3.0	**-3.0**
GFI1	growth factor independent 1	1.3				1.4		65.2	**65.2**			-5.9	**-5.9**
GFPT1	glutamine-fructose-6-phosphate transaminase 1	-1.4				-1.4		-2.1	**-2.1**				
GGA3	golgi associated, gamma adaptin ear containing, ARF binding protein 3					-1.5	**-1.5**	2.0	**2.0**	-1.4		-1.2	
GGH	gamma-glutamyl hydrolase (conjugase, folylpolygammaglutamyl hydrolase)							-2.7	**-2.7**	1.2	**1.2**	1.3	**1.3**
GGPS1	geranylgeranyl diphosphate synthase 1							-1.5	**-1.5**	1.3	**1.2**	1.3	**-1.2**
GGT1	gamma-glutamyltransferase 1			-1.6	**-1.6**	1.5							
GIT2	G protein-coupled receptor kinase interactor 2			1.3	**1.3**	-1.3		12.8	**12.8**			-2.8	**-2.8**
GJA7	gap junction protein, alpha 7, 45kDa (connexin 45)							-13.5	**-13.5**	1.6	**1.6**	43.1	**43.1**
GK	glycerol kinase			1.9								-1.8	
GLA	galactosidase, alpha			1.2		1.2		2.3	**2.3**	-1.2		-3.6	**-3.6**
GLB1	galactosidase, beta 1	1.4	**1.4**	2.3	**2.3**	1.9	**1.9**	2.3	**2.3**			-1.9	**-1.9**
GLE1L	GLE1 RNA export mediator-like (yeast)									-1.2	**-1.2**	-1.4	**-1.4**
GLG1	golgi apparatus protein 1					1.2						1.9	**1.9**
GLMN	glomulin, FKBP associated protein			-1.3	**-1.3**			3.5	**3.5**			-2.2	**-2.2**
GLO1	glyoxalase I	-1.3		-1.3	**-1.3**	-1.5		1.3	**1.3**	-1.5	**-1.5**	-2.0	**-2.0**
GLRX	glutaredoxin (thioltransferase)	3.4	**3.4**	5.2	**5.2**	2.1		-4.0	**-4.0**	1.6	**1.6**	4.4	**4.4**
GLS	glutaminase			1.3	**1.3**	1.7	**1.7**	2.1	**2.1**	-2.1	**-2.1**	-2.2	**-2.2**

GLUD1	glutamate dehydrogenase 1			-1.3		-1.3	**-1.3**	1.2		-1.4	**-1.3**	-1.5	**-1.5**
GLUD2	glutamate dehydrogenase 2							1.2				-1.5	**-1.5**
GLUL	glutamate-ammonia ligase (glutamine synthetase)	1.9	**1.9**	2.2	**2.2**	3.4		2.3	**2.3**	2.5	**2.5**	1.8	**1.8**
GM2A	GM2 ganglioside activator	1.3	**1.3**	2.1	**2.1**	1.5		1.4		1.5		1.6	**1.6**
GMDS	GDP-mannose 4,6-dehydratase			-1.2								-1.4	**-1.4**
GMFB	glia maturation factor, beta									1.2		1.6	**1.6**
GMFG	glia maturation factor, gamma			1.3	**1.3**					1.7	**1.7**		
GMPS	guanine monphosphate synthetase	-1.3		-1.3	**-1.3**	-1.4		-1.4	**-1.4**	-1.4	**-1.4**	-2.1	**-2.1**
GNA11	guanine nucleotide binding protein (G protein), alpha 11 (Gq class)	1.4						1.6	**1.6**	-1.4		-4.6	**-4.6**
GNA13	guanine nucleotide binding protein (G protein), alpha 13	-1.2		2.1				1.6	**1.6**	1.4		-1.5	**-1.5**
GNA15	guanine nucleotide binding protein (G protein), alpha 15 (Gq class)					-1.3		-3.2	**-3.2**	1.4	**1.4**		
GNAI2	guanine nucleotide binding protein (G protein), alpha inhibiting activity polypeptide 2												
GNAI3	guanine nucleotide binding protein (G protein), alpha inhibiting activity polypeptide 3			1.4						2.0	**2.0**	1.4	**1.4**
GNAQ	guanine nucleotide binding protein (G protein), q polypeptide	-1.3		1.3				5.0	**5.0**	1.3	**1.3**	-1.6	**-1.6**
GNAS	GNAS complex locus			1.3		1.3		-1.7	**-1.7**	1.7	**1.7**	2.4	**2.4**
GNB1	guanine nucleotide binding protein (G protein), beta polypeptide 1			-1.2				-1.4	**-1.4**	1.3		1.7	**1.7**
GNB2	guanine nucleotide binding protein (G protein), beta polypeptide 2							-1.7	**-1.7**	1.4	**1.4**	1.7	**1.7**
GNB2L1	guanine nucleotide binding protein (G protein), beta polypeptide 2-like 1									-1.6	**-1.6**	-1.5	
GNB5	guanine nucleotide binding protein (G protein), beta 5							-2.8	**-2.8**	-1.3	**-1.3**	1.3	
GNE	glucosamine (UDP-N-acetyl)-2-epimerase/N-acetylmannosamine kinase	-1.4		-1.6	**-1.6**	-1.4	**-1.4**	-3.9	**-3.9**			2.5	**2.5**
GNG10	guanine nucleotide binding protein (G protein), gamma 10	1.2		1.3	**1.3**								
GNG5	guanine nucleotide binding protein (G protein), gamma 5					-1.3							
GNL1	guanine nucleotide binding protein-like 1			-1.3		1.8		-1.3	**-1.3**	-1.2		1.3	
GNL2	guanine nucleotide binding protein-like 2 (nucleolar)					-1.4				-1.4	**-1.4**	-2.0	**-2.0**
GNPAT	glyceronephosphate O-acyltransferase	1.4	**1.4**									1.4	**1.4**
GNPDA1	glucosamine-6-phosphate deaminase 1							11.5	**11.5**			-59.9	**-59.9**
GNS	glucosamine (N-acetyl)-6-sulfatase (Sanfilippo disease IIID)	-1.5						2.8	**2.8**			-2.0	**-2.0**
GOLGA1	golgi autoantigen, golgin subfamily a, 1											1.5	**1.5**
GOLGA2	golgi autoantigen, golgin subfamily a, 2					-1.3		1.7	**1.7**				
GOLGA3	golgi autoantigen, golgin subfamily a, 3					1.3						-1.6	
GOLGA4	golgi autoantigen, golgin subfamily a, 4			1.4				1.5		-1.2		-1.5	**-1.5**
GORASP2	golgi reassembly stacking protein 2, 55kDa			-1.4		-1.2		1.8	**1.8**	-1.3	**-1.3**	-2.0	**-2.0**
GOSR1	golgi SNAP receptor complex member 1					1.3		1.5	**1.5**			-1.7	**-1.7**
GOSR2	golgi SNAP receptor complex member 2			1.6	**1.6**			1.2		-1.3		-1.5	**-1.5**
GOT1	glutamic-oxaloacetic transaminase 1, soluble (aspartate aminotransferase 1)							1.2		-1.5		-1.4	**-1.4**
GOT2	glutamic-oxaloacetic transaminase 2, mitochondrial (aspartate aminotransferase 2)	-1.3	**-1.3**	-1.3	**-1.3**	-1.4		-1.3				-1.5	**-1.5**
GP5	glycoprotein V (platelet)												
GPA33	glycoprotein A33 (transmembrane)			1.4		-1.5	**-1.5**						
GPAA1	glycosylphosphatidylinositol anchor attachment protein 1 homolog (yeast)							1.7				-2.2	**-1.4**
GPATCH8	G patch domain containing 8	1.2		1.6	**1.6**			1.4		1.7	**1.7**	3.5	**3.5**
GPC1	glypican 1	-1.2				1.4		-1.2		1.4	**1.4**	1.6	
GPC3	glypican 3												
GPI	glucose phosphate isomerase	-1.2		-1.2						-3.3	**-3.3**	-2.0	**-2.0**
GPLD1	glycosylphosphatidylinositol specific phospholipase D1			1.2				-2.3	**-2.3**	-1.7	**-1.7**	-1.4	
GPR125	G protein-coupled receptor 125	-1.8	**-1.8**	-1.5		-1.6		-1.2				-4.2	**-4.2**
GPR161	G protein-coupled receptor 161	-1.2						-1.2					
GPR30	G protein-coupled receptor 30												
GPR56	G protein-coupled receptor 56	1.3				1.2				25.3	**25.3**		
GPS2	G protein pathway suppressor 2							-4.7	**-4.7**			3.8	**3.8**
GPSM2	G-protein signalling modulator 2 (AGS3-like, C. elegans)	1.7	**1.7**					-1.8	**-1.8**	1.9	**1.6**	2.7	**2.7**
GPSM3	G-protein signalling modulator 3 (AGS3-like, C. elegans)							-1.9	**-1.9**			3.0	**3.0**
GPSN2	glycoprotein, synaptic 2			-1.2				-1.3	**-1.3**	-1.2		-1.5	**-1.5**
GPX1	glutathione peroxidase 1			-1.2	**-1.2**			1.7	**1.7**	1.7	**1.7**		
GPX4	glutathione peroxidase 4 (phospholipid hydroperoxidase)			-1.4	**-1.4**	-1.3		1.8	**1.8**			-2.2	**-2.2**
GPX7	glutathione peroxidase 7							1.9	**1.9**			-1.5	**-1.5**
GRAP2	GRB2-related adaptor protein 2	2.4	**2.4**	2.9	**2.9**	3.6	**3.6**						
GRB10	growth factor receptor-bound protein 10			-1.6	**-1.6**			-12.4	**-12.4**	-1.4	**-1.4**	11.1	**11.1**
GRB2	growth factor receptor-bound protein 2					-1.4		1.9	**1.9**	-1.8	**-1.8**	-3.3	**-3.3**

GREB1	GREB1 protein					2.6	**2.6**	8.1	**8.1**			-11.0	**-11.0**
GREM1	gremlin 1, cysteine knot superfamily, homolog (Xenopus laevis)							-42.1	**-42.1**				
GRHPR	glyoxylate reductase/hydroxypyruvate reductase							1.4	**1.4**	-1.6	**-1.6**	-1.3	**-1.3**
GRIK5	glutamate receptor, ionotropic, kainate 5					1.4		4.2	**4.2**			-14.5	**-14.5**
GRINA	glutamate receptor, ionotropic, N-methyl D-asparate-associated protein 1 (glutamate binding)									-1.8	**-1.8**		
GRIP2	glutamate receptor interacting protein 2												
GRK5	G protein-coupled receptor kinase 5	1.3						14.8	**14.8**	2.8	**2.8**	-3.8	**-3.8**
GRK6	G protein-coupled receptor kinase 6							-3.3	**-3.3**			2.2	**2.2**
GRLF1	glucocorticoid receptor DNA binding factor 1			-1.3		1.5		-1.5	**1.4**	1.5		1.3	**-1.2**
GRM4	glutamate receptor, metabotropic 4			1.4		1.7							
GRSF1	G-rich RNA sequence binding factor 1	-1.2				-1.5		-1.2				-2.7	**-2.7**
GSK3A	glycogen synthase kinase 3 alpha			-1.2						-1.3			
GSK3B	glycogen synthase kinase 3 beta	1.6		1.9	**1.9**	1.2		2.1	**2.1**	1.5	**1.3**	-2.3	**-2.3**
GSPT1	G1 to S phase transition 1	-1.5	**-1.5**	-1.4	**-1.4**	-1.8	**-1.8**	-1.6	**-1.6**	-1.2	**-1.2**	-1.4	**-1.4**
GSR	glutathione reductase			1.2		1.3		-1.6	**-1.6**	1.4		1.2	**1.2**
GSS	glutathione synthetase							1.3		-1.3	**-1.3**	-2.2	**-2.2**
GSTM1	glutathione S-transferase M1					1.3		-1.6	**-1.6**	-1.7	**-1.7**	2.3	**2.3**
GSTO1	glutathione S-transferase omega 1							1.8	**1.8**	-1.3	**-1.3**	-3.0	**-3.0**
GSTP1	glutathione S-transferase pi							-1.2	**-1.2**	-1.3		-1.7	**-1.7**
GSTZ1	glutathione transferase zeta 1 (maleylacetoacetate isomerase)	1.2											
GTF2A2	general transcription factor IIA, 2, 12kDa	1.6		1.8	**1.8**	1.5	**1.5**	-2.8	**-2.8**	-1.3	**-1.2**	1.6	**1.6**
GTF2B	general transcription factor IIB			1.4				-1.2	**-1.2**				
GTF2E1	general transcription factor IIE, polypeptide 1, alpha 56kDa							1.2		-1.2		-1.5	**-1.5**
GTF2E2	general transcription factor IIE, polypeptide 2, beta 34kDa	-1.4	**-1.4**			-1.3		-1.5	**-1.5**	-1.3	**-1.3**		
GTF2F1	general transcription factor IIF, polypeptide 1, 74kDa	1.2	**1.2**					1.3		-1.7	**-1.7**		
GTF2F2	general transcription factor IIF, polypeptide 2, 30kDa	-1.5		-1.5								-1.3	
GTF2H1	general transcription factor IIH, polypeptide 1, 62kDa					-1.2		1.3	**1.3**	-1.4	**-1.3**	-2.0	**-2.0**
GTF2H2	general transcription factor IIH, polypeptide 2, 44kDa	-2.1				-1.9		2.1	**2.1**	1.3		-3.2	**-3.2**
GTF2H3	general transcription factor IIH, polypeptide 3, 34kDa			1.3		-1.2				-1.4	**-1.4**	-1.5	
GTF2H4	general transcription factor IIH, polypeptide 4, 52kDa					-1.3	**-1.3**	1.7	**1.7**				
GTF2H5	general transcription factor IIH, polypeptide 5					-1.4						1.4	**1.4**
GTF2I	general transcription factor II, i	-1.2		1.4	**1.4**	-1.3		-2.1	**-2.1**			2.3	**2.3**
GTF3A	general transcription factor IIIA	-1.3		-1.6	**-1.6**	-1.5		-1.7	**-1.7**	-1.4	**-1.4**	-2.0	**-2.0**
GTF3C1	general transcription factor IIIC, polypeptide 1, alpha 220kDa							-1.3	**-1.3**	-1.5		1.7	**1.7**
GTF3C2	general transcription factor IIIC, polypeptide 2, beta 110kDa									-2.0	**-2.0**	-1.5	**-1.2**
GTPBP6	GTP binding protein 6 (putative)	-1.4		-1.8	**-1.8**	-1.3		-2.2	**-2.2**			1.8	**1.8**
GTSE1	G-2 and S-phase expressed 1							-1.7	**-1.7**	2.1	**1.6**	2.0	**2.0**
GUK1	guanylate kinase 1												
GUSB	glucuronidase, beta							1.2	**1.2**				
GYG1	glycogenin 1			1.3						3.8	**3.8**	2.7	**2.7**
GYPC	glycophorin C (Gerbich blood group)					2.8	**2.8**	1.8	**1.8**			-1.8	**-1.8**
GYS1	glycogen synthase 1 (muscle)	1.3						1.5	**1.5**	-1.3	**-1.3**		
H1F0	H1 histone family, member 0	1.4		2.0	**2.0**	2.5	**2.5**	8.1	**8.1**	2.4	**2.4**	-5.6	**-5.6**
H1FX	H1 histone family, member X	1.5	**1.5**	1.4		1.3	**1.3**	-3.2	**-3.2**			3.7	**3.7**
H2AFX	H2A histone family, member X	1.2						-2.6	**-2.6**			1.3	**1.3**
H2AFY	H2A histone family, member Y							-1.9	**-1.9**	-1.6	**-1.6**	-2.8	**-2.8**
H2AFZ	H2A histone family, member Z							-1.2				-1.9	**-1.9**
H3F3A	H3 histone, family 3A							-1.2	**-1.2**			1.3	**1.3**
H3F3B	H3 histone, family 3B (H3.3B)					-1.3		4.1	**4.1**	1.3	**1.3**	-2.2	**-2.2**
H41	CDV3 homolog (mouse)	1.2		1.5	**1.5**	1.2		1.3	**1.3**	-1.2	**-1.2**	-1.4	**-1.4**
H6PD	hexose-6-phosphate dehydrogenase (glucose 1-dehydrogenase)							-2.4	**-2.4**			2.5	
HADH	hydroxyacyl-Coenzyme A dehydrogenase												
HADH2	hydroxysteroid (17-beta) dehydrogenase 10	-1.2						-1.2	**-1.2**			-1.4	
HADHA	hydroxyacyl-Coenzyme A dehydrogenase/3-ketoacyl-Coenzyme A thiolase/enoyl-Coenzyme A hydratase (trifunctional protein), alpha subunit							1.3		1.5	**1.5**	-1.3	
HADHB	hydroxyacyl-Coenzyme A dehydrogenase/3-ketoacyl-Coenzyme A thiolase/enoyl-Coenzyme A hydratase (trifunctional protein), beta subunit							1.8	**1.8**			-1.4	**-1.4**
HADHSC	--			1.3		-1.3				-1.7	**-1.7**	-1.3	**-1.3**
HAGH	hydroxyacylglutathione hydrolase									1.3		2.2	**2.2**
HAN11	WD repeat domain 68	1.2		1.3				1.8	**1.8**	1.3	**-1.2**	-1.7	**-1.7**

HARSL	histidyl-tRNA synthetase-like					1.3		1.2					
HAT1	histone acetyltransferase 1					-1.3		1.6	**1.6**			-4.0	**-4.0**
HAX1	HCLS1 associated protein X-1	-1.2				-1.3	**-1.3**	-1.3	**-1.3**	-1.7	**-1.7**	-1.3	**-1.3**
HBP1	HMG-box transcription factor 1	1.5		1.8	**1.8**	1.6	**1.6**	1.6	**1.6**	1.4	**1.4**	1.5	**1.5**
HBXIP	hepatitis B virus x interacting protein	-1.2		-1.3		-1.3				1.2	**1.2**		
HCAP-D3	non-SMC condensin II complex, subunit D3									-2.1	**-2.1**	-2.9	**-2.9**
HCCS	holocytochrome c synthase (cytochrome c heme-lyase)									-1.2	**-1.2**	-2.0	**-2.0**
HCFC1	host cell factor C1 (VP16-accessory protein)					-1.4		-1.5	**-1.5**			1.2	**1.2**
HCK	hemopoietic cell kinase			1.3									
HCLS1	hematopoietic cell-specific Lyn substrate 1	1.3		1.2	**1.2**			-1.6	**-1.6**	-1.4	**-1.4**	1.8	**1.8**
HCRT	hypocretin (orexin) neuropeptide precursor	1.3											
HDAC1	histone deacetylase 1	-1.4	**-1.4**	-1.2		-1.2						-1.5	**-1.5**
HDAC2	histone deacetylase 2	-1.3		-1.5	**-1.5**	-1.5	**-1.5**	1.3		-1.5	**-1.5**	-1.6	**-1.6**
HDAC3	histone deacetylase 3	-1.3						1.5	**1.5**	-1.4			
HDAC4	histone deacetylase 4	1.2						2.6	**2.6**	-1.8	**-1.8**	1.3	**1.3**
HDAC6	histone deacetylase 6			1.2						-1.3		1.4	**1.4**
HDGF	hepatoma-derived growth factor (high-mobility group protein 1-like)			-1.3		1.6				-1.5	**-1.5**	-1.3	**-1.3**
HDLBP	high density lipoprotein binding protein (vigilin)					-1.3		-1.7	**-1.7**	-1.6	**-1.6**		
HEAB	CLP1, cleavage and polyadenylation factor I subunit, homolog (S. cerevisiae)											-1.2	**-1.2**
HELZ	helicase with zinc finger	1.2		1.4				1.5	**1.5**			-1.3	**-1.3**
HERPUD1	homocysteine-inducible, endoplasmic reticulum stress-inducible, ubiquitin-like domain member 1	-1.4				-1.6		-2.1	**-2.1**			2.1	**2.1**
HES1	hairy and enhancer of split 1, (Drosophila)	-5.5	**-5.5**	-12.8	**-12.8**	-9.3	**-9.3**					-3.1	
HEXA	hexosaminidase A (alpha polypeptide)	1.2						1.7	**1.7**			-1.4	**-1.4**
HEXIM1	hexamethylene bis-acetamide inducible 1	-1.4		1.5		1.6		4.5	**4.5**	1.8	**1.8**	-2.2	**-2.2**
HGS	hepatocyte growth factor-regulated tyrosine kinase substrate			-1.5	**-1.5**			1.5	**1.5**			-1.7	**-1.7**
HHEX	homeobox, hematopoietically expressed			1.3		-1.3		3.4	**3.4**	-2.0	**-2.0**	-5.4	**-5.4**
HIF1A	hypoxia-inducible factor 1, alpha subunit (basic helix-loop-helix transcription factor)									1.2	**1.2**		
HINT1	histidine triad nucleotide binding protein 1							1.3	**1.3**	1.4	**1.4**		
HIP1R	huntingtin interacting protein 1 related							-1.5	**-1.5**	2.3	**2.3**	1.6	**1.6**
HIP2	huntingtin interacting protein 2	-1.3		1.3		-1.3		-1.6	**-1.6**	-1.2	**-1.2**	-2.2	**-2.2**
HIPK1	homeodomain interacting protein kinase 1	1.6	**1.6**	2.2	**2.2**			-1.2		-1.5	**-1.5**	1.6	**1.3**
HIPK3	homeodomain interacting protein kinase 3	1.2				2.1		1.5	**1.3**	1.5	**1.5**	1.5	**1.5**
HIRA	HIR histone cell cycle regulation defective homolog A (S. cerevisiae)	-1.5		-1.3		-1.8	**-1.8**					-1.8	
HIRIP3	HIRA interacting protein 3							-1.3		-1.2		1.4	
HISPPD2A	histidine acid phosphatase domain containing 2A							-3.1	**-3.1**	-1.3		2.5	**2.5**
HIST1H2BJ	histone cluster 1, H2bj	1.4											
HIST1H3D	histone cluster 1, H3d	1.2											
HIST1H4C	histone cluster 1, H4c			-1.6				-1.3					
HIST2H2AA	histone cluster 2, H2aa3			2.1	**2.1**	1.2		-2.0	**-2.0**	1.3		2.9	**2.9**
HIST2H2BE	histone cluster 2, H2be					1.8				-2.3			
HIVEP1	human immunodeficiency virus type I enhancer binding protein 1	1.4						1.4	**1.4**	2.4	**2.4**	-1.5	**-1.5**
HIVEP2	human immunodeficiency virus type I enhancer binding protein 2	1.3		1.5	**1.5**	-1.5		-6.5	**-6.5**	-1.3		10.1	**10.1**
HK2	hexokinase 2	-2.0	**-2.0**	-2.3		1.2		1.4	**1.4**	-3.1	**-3.1**	-2.3	**-2.3**
HKE2	prefoldin subunit 6					-1.2						-1.3	
HLA-A	major histocompatibility complex, class I, A	1.5	**1.5**	1.7	**1.7**	1.3		-2.5	**-2.5**			2.7	**2.7**
HLA-B	major histocompatibility complex, class I, B							-3.4	**-3.4**	1.2	**1.2**	3.7	**3.7**
HLA-DMB	major histocompatibility complex, class II, DM beta	1.2		1.3				-1.7	**-1.7**			1.3	
HLA-DOB	major histocompatibility complex, class II, DO beta			-1.4									
HLA-DRB3	major histocompatibility complex, class II, DR beta 3					1.4							
HLA-E	major histocompatibility complex, class I, E	1.3						-1.3	**-1.3**	2.6	**2.6**	1.5	**1.5**
HLA-F	major histocompatibility complex, class I, F	1.3						-3.0	**-3.0**	2.2	**2.2**	4.7	**4.7**
HLA-G	HLA-G histocompatibility antigen, class I, G	1.5	**1.5**	1.3		1.3	**1.3**	-2.0	**-2.0**	1.5	**1.5**	1.9	**1.9**
HLXB9	homeobox HB9												
HMBS	hydroxymethylbilane synthase	-1.3		-1.9	**-1.9**	-1.6		-1.6	**-1.6**	-1.7		-1.9	**-1.9**
HMG20B	high-mobility group 20B							1.4				1.5	**1.5**
HMGA1	high mobility group AT-hook 1			-1.7				-1.7		-1.6	**-1.6**	-1.6	**-1.6**
HMGB1	high-mobility group box 1							-3.0	**-3.0**	1.4		1.9	**1.9**
HMGB2	high-mobility group box 2							-1.2		1.3	**1.3**	1.5	**1.5**

HMGB3	high-mobility group box 3			1.4		1.3				1.4	**1.4**	-1.5	**-1.5**
HMGCR	3-hydroxy-3-methylglutaryl-Coenzyme A reductase	-1.4	**-1.4**					-1.9	**-1.9**			2.0	**2.0**
HMGCS1	3-hydroxy-3-methylglutaryl-Coenzyme A synthase 1 (soluble)	-2.3	**-2.3**	-3.5	**-3.5**	-1.5	**-1.5**	-3.5	**-3.5**	-1.5		2.4	**2.4**
HMGCS2	3-hydroxy-3-methylglutaryl-Coenzyme A synthase 2 (mitochondrial)			1.3	**1.3**								
HMGN1	high-mobility group nucleosome binding domain 1											-1.6	**-1.6**
HMGN2	high-mobility group nucleosomal binding domain 2	-1.2											
HMGN3	high mobility group nucleosomal binding domain 3									1.3		1.5	**1.5**
HMMR	hyaluronan-mediated motility receptor (RHAMM)			1.4	**1.4**			-1.5	**-1.5**	1.8	**1.8**	3.3	**3.3**
HMOX2	heme oxygenase (decycling) 2					-1.2	**-1.2**					1.6	**1.6**
HNRPA0	heterogeneous nuclear ribonucleoprotein A0							1.5	**1.5**			-1.5	**-1.5**
HNRPA1	heterogeneous nuclear ribonucleoprotein A1			-1.2				1.9	**1.9**	1.4	**1.4**	-1.8	**-1.8**
HNRPA2B1	heterogeneous nuclear ribonucleoprotein A2/B1							5.0	**5.0**	1.3		-2.4	**-2.4**
HNRPA3	heterogeneous nuclear ribonucleoprotein A3					-1.4		1.8	**1.8**	-1.4	**-1.4**	-2.4	**-2.4**
HNRPAB	heterogeneous nuclear ribonucleoprotein A/B	-1.5	**-1.5**	-1.8	**-1.8**	-1.4	**-1.4**	-1.4	**-1.4**	-1.6	**-1.6**	-1.8	**-1.8**
HNRPC	heterogeneous nuclear ribonucleoprotein C (C1/C2)	-1.2						-1.2	**-1.2**	-1.5	**-1.5**	-1.9	**-1.9**
HNRPD	heterogeneous nuclear ribonucleoprotein D (AU-rich element RNA binding protein 1, 37kDa)							3.1	**3.1**	1.5		-1.8	**-1.8**
HNRPDL	heterogeneous nuclear ribonucleoprotein D-like	-1.4		-1.6	**-1.6**	-1.2		1.7	**1.7**	-1.6	**-1.6**	-2.4	**-2.4**
HNRPF	heterogeneous nuclear ribonucleoprotein F			-1.2				1.3	**1.3**	-1.3		-2.5	**-2.5**
HNRPH1	heterogeneous nuclear ribonucleoprotein H1 (H)					-1.3		-2.9	**-2.9**	1.3	**1.3**	2.1	**2.1**
HNRPH2	heterogeneous nuclear ribonucleoprotein H2 (H')	1.3		1.3						1.5	**1.5**		
HNRPH3	heterogeneous nuclear ribonucleoprotein H3 (2H9)			-1.3	**-1.3**			-1.4	**-1.4**	1.2		-1.3	**-1.3**
HNRPK	heterogeneous nuclear ribonucleoprotein K									-1.4	**-1.4**		
HNRPL	heterogeneous nuclear ribonucleoprotein L							-1.4	**-1.4**	-2.0	**-2.0**	1.3	**1.3**
HNRPM	heterogeneous nuclear ribonucleoprotein M							1.8	**1.8**	1.8	**1.8**	-2.2	**-2.2**
HNRPR	heterogeneous nuclear ribonucleoprotein R					-1.4						-2.0	**-2.0**
HNRPU	heterogeneous nuclear ribonucleoprotein U (scaffold attachment factor A)	-1.3				-1.3		-1.4	**-1.4**	-1.4	**-1.4**	-2.9	**-2.9**
HNRPUL1	heterogeneous nuclear ribonucleoprotein U-like 1							-1.4	**-1.4**			3.4	**3.4**
HOMER1	homer homolog 1 (Drosophila)			-1.3		-1.4		-2.3	**-2.3**	-1.3		-1.3	
HOM-TES-103	hypothetical protein LOC25900	1.2		1.4	**1.4**					2.3	**2.3**	1.6	**1.4**
HOXA9	homeobox A9							-91.3	**-91.3**			194.0	**194.0**
HOXB6	homeobox B6					1.6		-1.4		1.5		1.8	
HOXB7	homeobox B7					1.8							
HOXD4	homeobox D4			1.2									
HPCAL1	hippocalcin-like 1	1.3	**1.3**	1.4		1.7		1.3				1.5	**1.3**
HPGD	hydroxyprostaglandin dehydrogenase 15-(NAD)	1.3	**1.3**	1.5						2.6	**2.6**		
HPRP8BP	--			-1.2		-1.5		-1.3	**-1.3**	-1.5	**-1.5**	-1.5	**-1.5**
HPRT1	hypoxanthine phosphoribosyltransferase 1 (Lesch-Nyhan syndrome)			-1.3	**-1.3**	-1.5				-1.6	**-1.6**		
HPS5	Hermansky-Pudlak syndrome 5												
HRAS	v-Ha-ras Harvey rat sarcoma viral oncogene homolog	-1.3	**-1.3**	-1.4		-1.3		-1.8	**-1.8**	1.3			
HRB	HIV-1 Rev binding protein							-1.7	**-1.7**	1.5	**1.5**	3.8	**3.8**
HRB2	KRR1, small subunit (SSU) processome component, homolog (yeast)	-1.4				-1.7				-1.3	**-1.3**	-1.2	**-1.2**
HRMT1L2	protein arginine methyltransferase 1	-1.5		-2.2	**-2.2**	-1.7	**-1.7**	-1.2	**-1.2**	-2.6	**-2.6**	-2.6	**-2.6**
HRSP12	heat-responsive protein 12					-1.4				-2.0	**-2.0**	-2.7	**-2.7**
HS2ST1	heparan sulfate 2-O-sulfotransferase 1							1.8	**1.8**	1.5		-1.4	**-1.4**
HS6ST1	heparan sulfate 6-O-sulfotransferase 1	1.3		2.1	**2.1**	2.2	**2.2**	1.3	**1.3**	1.8	**1.8**	1.5	**1.5**
HSBP1	heat shock factor binding protein 1							-1.4	**-1.4**	-1.3	**-1.3**		
HSD17B4	hydroxysteroid (17-beta) dehydrogenase 4			1.9	**1.9**	1.9		2.0	**2.0**	1.9	**1.9**	2.0	**2.0**
HSD17B8	hydroxysteroid (17-beta) dehydrogenase 8			-1.2									
HSF1	heat shock transcription factor 1							-1.4		-1.2		-1.5	**-1.5**
HSF2	heat shock transcription factor 2							1.2	**1.2**				
HSF4	heat shock transcription factor 4	1.2				1.8							
HSPA1A	heat shock 70kDa protein 1A	-2.0						6.3	**6.3**			-8.1	**-8.1**
HSPA4	heat shock 70kDa protein 4					-1.6	**-1.6**	1.3		-1.4	**-1.4**	-1.8	**-1.8**
HSPA4L	heat shock 70kDa protein 4-like			-1.6		-1.6		25.8	**25.8**			-42.0	**-42.0**
HSPA5	heat shock 70kDa protein 5 (glucose-regulated protein, 78kDa)	-1.3				-1.5	**-1.5**	1.6	**1.6**			-3.2	**-3.2**
HSPA8	heat shock 70kDa protein 8	-1.3	**-1.3**			-1.9	**-1.9**			-1.3	**-1.3**		
HSPA9B	heat shock 70kDa protein 9 (mortalin)	-1.5		-1.5	**-1.4**	-1.6	**-1.6**			-2.8	**-2.8**	-3.8	**-3.8**
HSPB1	heat shock 27kDa protein 1							-2.7	**-2.7**	-1.3	**-1.3**	1.6	**1.6**

HSPB2	heat shock 27kDa protein 2	1.5											
HSPBP1	hsp70-interacting protein	-1.4	**-1.4**	-1.8	**-1.8**							-1.5	**-1.5**
HSPCA	heat shock protein 90kDa alpha (cytosolic), class A member 1			-1.3		-1.2	**-1.2**	-1.2	**-1.2**	-1.7			
HSPCB	heat shock protein 90kDa alpha (cytosolic), class B member 1	-1.5	**-1.4**	-1.8	**-1.8**	-1.3	**-1.3**	1.4	**1.4**	-2.0	**-2.0**	-1.8	**-1.8**
HSPD1	heat shock 60kDa protein 1 (chaperonin)	-1.4	**-1.4**	-1.5	**-1.5**	-1.3				-3.0	**-3.0**	-2.3	**-2.3**
HSPE1	heat shock 10kDa protein 1 (chaperonin 10)	-1.8	**-1.8**	-2.1	**-2.1**	-2.1	**-2.1**			-2.2	**-2.2**	-3.7	**-3.7**
HTATSF1	HIV-1 Tat specific factor 1	-1.5		-1.3				1.4	**1.4**			-2.0	**-2.0**
HTR1B	5-hydroxytryptamine (serotonin) receptor 1B			-1.5									
HTR7	5-hydroxytryptamine (serotonin) receptor 7 (adenylate cyclase-coupled)							-1.4	**-1.4**	-1.3		1.2	
HUWE1	HECT, UBA and WWE domain containing 1			1.6		-1.2		1.4	**1.4**			-1.3	**-1.3**
HYAL2	hyaluronoglucosaminidase 2	-1.4		-1.6	**-1.6**							-6.2	**-6.2**
HYOU1	hypoxia up-regulated 1			-1.2				1.8	**1.8**	-1.7	**-1.7**	-2.3	**-2.3**
HYPB	SET domain containing 2	1.2		1.4						1.4	**1.3**	1.3	**1.3**
IAPP	islet amyloid polypeptide												
IARS	isoleucine-tRNA synthetase	-1.6	**-1.6**	-1.7	**-1.7**	-1.7		-1.4	**-1.4**	-2.2	**-2.2**	-3.8	**-3.8**
IARS2	isoleucine-tRNA synthetase 2, mitochondrial	-1.2		-1.3		-1.5		1.4	**1.4**	-1.2	**-1.2**	-1.4	**-1.4**
IBRDC3	IBR domain containing 3							-2.0	**-2.0**			2.3	**2.3**
IBTK	inhibitor of Bruton agammaglobulinemia tyrosine kinase			-1.4				1.5	**1.5**	1.6	**1.6**	-1.4	**-1.4**
ICAM2	intercellular adhesion molecule 2	-1.8	**-1.8**	-2.0	**-2.0**	-1.7	**-1.7**	-17.5	**-17.5**	-2.4	**-2.4**	3.3	**3.3**
ICAM3	intercellular adhesion molecule 3											1.3	**1.3**
ID1	inhibitor of DNA binding 1, dominant negative helix-loop-helix protein	-2.6		-1.7	**-1.7**	-1.2		9.9				-17.8	**-17.8**
ID2	inhibitor of DNA binding 2, dominant negative helix-loop-helix protein	1.8	**1.8**	2.5	**2.5**			5.8	**5.8**	3.0	**3.0**	3.3	**3.3**
ID3	inhibitor of DNA binding 3, dominant negative helix-loop-helix protein	-1.6		-1.6	**-1.6**			-33.8	**-33.8**	-1.9	**-1.9**		
IDE	insulin-degrading enzyme							1.4	**1.4**	-1.4		-1.7	**-1.7**
IDH1	isocitrate dehydrogenase 1 (NADP+), soluble							-1.3	**-1.3**	-1.6	**-1.6**	1.8	**1.8**
IDH2	isocitrate dehydrogenase 2 (NADP+), mitochondrial			-1.3		-1.4		-1.4	**-1.4**	-1.4	**-1.4**	-2.3	**-2.3**
IDH3A	isocitrate dehydrogenase 3 (NAD+) alpha	-1.5	**-1.5**	-1.3	**-1.3**	-1.5	**-1.5**	-1.6	**-1.6**	-1.7	**-1.7**	-2.4	**-2.4**
IDH3B	isocitrate dehydrogenase 3 (NAD+) beta							1.5	**1.5**			-2.1	**-2.1**
IDH3G	isocitrate dehydrogenase 3 (NAD+) gamma											-1.3	**-1.3**
IDI1	isopentenyl-diphosphate delta isomerase 1			-1.7	**-1.7**	-1.3		1.5	**1.5**	1.7	**1.7**	2.1	**2.1**
IDS	iduronate 2-sulfatase (Hunter syndrome)	1.4		1.9	**1.9**	1.2		-1.6	**-1.6**			1.5	**1.5**
IER2	immediate early response 2	-1.3		-1.4		-1.4	**-1.4**	1.6	**1.6**			-2.7	**-2.7**
IER3	immediate early response 3			-1.9									
IFI16	interferon, gamma-inducible protein 16	-1.3	**-1.3**	1.2		1.4		2.0	**2.0**	2.4	**2.4**	-1.3	**-1.3**
IFI44	interferon-induced protein 44	1.5		1.8	**1.8**	1.4						1.9	**1.9**
IFITM1	interferon induced transmembrane protein 1 (9-27)							4.6	**4.6**	3.2	**3.2**	-14.3	**-14.3**
IFITM2	interferon induced transmembrane protein 2 (1-8D)			1.5		1.5		-3.1	**-3.1**	2.2	**2.2**	1.6	**1.6**
IFITM3	interferon induced transmembrane protein 3 (1-8U)	1.4	**1.4**	1.5	**1.5**					1.8	**1.8**		
IFNAR2	interferon (alpha, beta and omega) receptor 2			1.4	**1.4**	-1.2		-2.9	**-2.9**	-1.4		1.2	
IFNGR1	interferon gamma receptor 1	1.7	**1.7**	2.0	**2.0**	1.2		5.7	**5.7**	3.3	**3.3**	2.6	**2.6**
IFRD1	interferon-related developmental regulator 1	-1.8	**-1.8**	-1.2		-1.6	**-1.3**			-1.3		-1.4	**-1.4**
IFRD2	interferon-related developmental regulator 2	-1.8	**-1.8**	-1.7	**-1.7**	-1.7	**-1.7**			-3.4	**-3.4**	-3.7	**-3.7**
IFT20	intraflagellar transport 20 homolog (Chlamydomonas)			1.3				1.4		1.5	**1.5**		
IGBP1	immunoglobulin (CD79A) binding protein 1	1.2		1.4	**1.4**					1.2		1.4	**1.4**
IGF2	insulin-like growth factor 2 (somatomedin A)												
IGF2R	insulin-like growth factor 2 receptor			-1.4				-1.3	**1.3**	1.7	**1.7**	1.7	**1.7**
IGFBP1	insulin-like growth factor binding protein 1												
IGFBP2	insulin-like growth factor binding protein 2, 36kDa			-1.6	**-1.6**	-1.7		-1.2				-5.1	**-5.1**
IGHG1	immunoglobulin heavy constant gamma 1 (G1m marker)					1.3		-2.6	**-2.6**	1.3	**1.3**	3.7	**3.7**
IGHM	immunoglobulin heavy constant mu	1.4				1.6		-39.4	**-39.4**	1.4	**1.4**	80.1	**80.1**
IGHMBP2	immunoglobulin mu binding protein 2	1.3											
IGL@	immunoglobulin lambda locus												
IGLC1	immunoglobulin lambda constant 1 (Mcg marker)			-1.4				-10.6	**-10.6**	-1.2		6.5	**6.5**
IGLL1	immunoglobulin lambda-like polypeptide 1	-1.4	**-1.4**			-1.3		-44.6	**-44.6**	-1.2	**-1.2**	9.6	**9.6**
IHPK1	inositol hexaphosphate kinase 1			1.4	**1.4**			1.8	**1.8**	-1.4	**-1.4**	-1.7	**-1.7**
IK	IK cytokine, down-regulator of HLA II	1.3		1.6	**1.4**								
IKBKAP	inhibitor of kappa light polypeptide gene enhancer in B-cells, kinase complex-associated protein					-1.3						-1.3	**-1.3**
IKBKB	inhibitor of kappa light polypeptide gene enhancer in B-cells, kinase beta			1.4		1.8	**1.8**						

IKBKE	inhibitor of kappa light polypeptide gene enhancer in B-cells, kinase epsilon			1.2		1.2						-2.0	**-2.0**
IKBKG	inhibitor of kappa light polypeptide gene enhancer in B-cells, kinase gamma					1.4		-1.5	**-1.5**			1.6	**1.6**
IL10RB	interleukin 10 receptor, beta	1.6	**1.6**	1.9	**1.9**	1.4						1.3	
IL11RA	interleukin 11 receptor, alpha	1.8	**1.8**										
IL16	interleukin 16 (lymphocyte chemoattractant factor)			1.2									
IL17R	interleukin 17 receptor A	1.5	**1.5**	2.0						1.6		1.3	**1.3**
IL27RA	interleukin 27 receptor, alpha					-1.3		20.9	**20.9**			-7.5	**-7.5**
IL2RG	interleukin 2 receptor, gamma (severe combined immunodeficiency)							-1.4	**-1.4**	-1.4	**-1.4**		
IL32	interleukin 32	-1.5		-1.9	**-1.9**	-1.9	**-1.9**						
IL4	interleukin 4			1.4									
IL4R	interleukin 4 receptor	1.2											
IL5RA	interleukin 5 receptor, alpha	-1.2											
IL6ST	interleukin 6 signal transducer (gp130, oncostatin M receptor)	1.4		1.8				4.6	**4.6**	4.7	**4.7**	4.8	**4.8**
IL7R	interleukin 7 receptor	8.3	**8.3**	18.0	**18.0**	6.3	**6.3**	-56.6	**-56.6**	-3.1	**-3.1**	43.1	**43.1**
IL9R	interleukin 9 receptor							-1.2				1.9	**1.9**
ILF2	interleukin enhancer binding factor 2, 45kDa					-1.3						-1.6	**-1.6**
ILF3	interleukin enhancer binding factor 3, 90kDa	-1.4	**-1.3**	-1.2		-1.3		1.7	**1.7**	-2.2	**-2.2**	-4.0	**-4.0**
ILK	integrin-linked kinase	1.3	**1.3**	1.2				1.4	**1.4**			1.3	
ILVBL	ilvB (bacterial acetolactate synthase)-like			-1.2	**-1.2**					-1.2			
IMMT	inner membrane protein, mitochondrial (mitofilin)							1.4	**1.4**	-1.3		-1.6	**-1.6**
IMP-3	insulin-like growth factor 2 mRNA binding protein 3					1.4		1.9	**1.9**	2.0	**2.0**	-1.7	**-1.7**
IMP4	IMP4, U3 small nucleolar ribonucleoprotein, homolog (yeast)	-1.6	**-1.6**	-1.5	**-1.5**	-1.4						-1.4	**-1.4**
IMPA1	inositol(myo)-1(or 4)-monophosphatase 1			-1.2				-1.7	**-1.7**	1.2	**1.2**	2.3	**2.3**
IMPA2	inositol(myo)-1(or 4)-monophosphatase 2			-1.4		-1.6		1.8	**1.8**	-12.4	**-12.4**	-7.9	**-7.9**
IMPDH1	IMP (inosine monophosphate) dehydrogenase 1	-1.6	**-1.6**	-1.9	**-1.9**	-1.4	**-1.4**	-2.0	**-2.0**				
IMPDH2	IMP (inosine monophosphate) dehydrogenase 2	-1.3				-1.5	**-1.5**	1.3	**1.3**	-1.4	**-1.4**	-1.5	**-1.5**
INA	internexin neuronal intermediate filament protein, alpha					1.4							
INDO	indoleamine-pyrrole 2,3 dioxygenase												
ING1	inhibitor of growth family, member 1							-1.3				1.6	**1.6**
ING2	inhibitor of growth family, member 2			-1.3				1.4		-2.4	**-2.4**	-2.7	**-2.7**
ING3	inhibitor of growth family, member 3							-1.9	**-1.9**	1.3	**1.3**	3.0	**3.0**
INPP1	inositol polyphosphate-1-phosphatase	3.5	**3.5**	4.5	**4.5**	17.6	**17.6**	1.4	**1.4**	2.7	**2.7**	13.6	**13.6**
INPP4A	inositol polyphosphate-4-phosphatase, type I, 107kDa	-1.3		1.3		-1.4							
INPP5B	inositol polyphosphate-5-phosphatase, 75kDa					-1.2						1.7	
INPP5D	inositol polyphosphate-5-phosphatase, 145kDa							-1.6	**-1.6**	2.0	**2.0**	1.5	**1.5**
INPP5E	inositol polyphosphate-5-phosphatase, 72 kDa												
INPP5F	inositol polyphosphate-5-phosphatase F	1.3								2.1	**2.1**	1.5	**1.5**
INPPL1	inositol polyphosphate phosphatase-like 1			1.6	**1.6**	1.3				-1.3			
INSIG1	insulin induced gene 1							-5.5	**-5.5**	1.7	**1.7**	3.7	**3.7**
INSIG2	insulin induced gene 2	1.2		1.7	**1.7**	-1.2		-1.4		1.3		1.3	
IPO7	importin 7	-1.4	**-1.4**			-1.3		1.5	**1.5**	-1.7	**-1.7**	-4.6	**-4.6**
IQCB1	IQ motif containing B1					-1.5	**-1.5**	-1.3	**-1.3**			-1.5	**-1.5**
IQGAP1	IQ motif containing GTPase activating protein 1	1.3		1.7	**1.7**	1.3		-2.4	**-2.4**	2.2	**2.2**	4.4	**4.4**
IQGAP2	IQ motif containing GTPase activating protein 2	1.4	**1.4**	1.9	**1.9**	1.9	**1.9**			2.6	**2.6**	2.0	**2.0**
IQSEC1	IQ motif and Sec7 domain 1	1.6		1.5	**1.5**	1.9				1.8	**1.8**	3.1	**3.1**
IRAK1	interleukin-1 receptor-associated kinase 1			-1.4	**-1.4**			-1.3	**-1.3**				
IRAK1BP1	interleukin-1 receptor-associated kinase 1 binding protein 1			-1.5		-1.2		-1.6	**-1.6**	-3.0	**-3.0**	1.6	**1.6**
IREB2	iron-responsive element binding protein 2	-1.6				-1.4		1.5	**1.5**	1.4		-1.9	**-1.9**
IRF2	interferon regulatory factor 2			1.5	**1.5**			-2.3	**-2.3**	-1.2		2.2	**2.2**
IRF3	interferon regulatory factor 3							-1.2	**-1.2**				
IRF6	interferon regulatory factor 6	1.4				1.9							
IRS1	insulin receptor substrate 1	1.4		1.4				-4.8	**-4.8**	1.5	**1.5**	7.7	**7.7**
IRX5	iroquois homeobox protein 5					1.3		5.0	**5.0**			-12.2	**-12.2**
ISG20	interferon stimulated exonuclease gene 20kDa	9.5	**9.5**	9.0	**9.0**	7.6	**7.6**			91.9	**91.9**	274.3	**274.3**
ISLR	immunoglobulin superfamily containing leucine-rich repeat			1.2									
ITCH	itchy homolog E3 ubiquitin protein ligase (mouse)					-1.3		2.0	**2.0**	1.8	**1.8**	1.6	**1.6**
ITGA2B	integrin, alpha 2b (platelet glycoprotein IIb of IIb/IIIa complex, antigen CD41)	1.2				1.7							
ITGA3	integrin, alpha 3 (antigen CD49C, alpha 3 subunit of VLA-3 receptor)	1.3		1.2		1.3							

ITGA4	integrin, alpha 4 (antigen CD49D, alpha 4 subunit of VLA-4 receptor)	-2.4	**-2.1**	-1.5		-2.3		-1.4				-5.7	**-5.7**
ITGA6	integrin, alpha 6	5.0	**5.0**	10.2	**10.2**	3.5	**2.4**	6.1	**6.1**	7.2	**7.2**	-1.9	**-1.9**
ITGAE	integrin, alpha E (antigen CD103, human mucosal lymphocyte antigen 1; alpha polypeptide)	1.3		1.6	**1.6**			-2.9	**-2.9**	1.5	**1.5**	3.1	**3.1**
ITGAL	integrin, alpha L (antigen CD11A (p180), lymphocyte function-associated antigen 1; alpha polypeptide)			-1.6	**-1.6**			-1.7	**-1.7**	-1.4	**-1.4**	1.4	
ITGB1	integrin, beta 1 (fibronectin receptor, beta polypeptide, antigen CD29 includes MDF2, MSK12)							3.1	**3.1**	1.4	**1.4**	-2.1	**-2.1**
ITGB1BP1	integrin beta 1 binding protein 1					-1.5		-1.2	**-1.2**	1.4			
ITGB2	integrin, beta 2 (complement component 3 receptor 3 and 4 subunit)							2.6	**2.6**	-5.6	**-5.6**	-8.7	**-8.7**
ITGB3BP	integrin beta 3 binding protein (beta3-endonexin)	1.2				1.4				-1.2	**-1.2**	-1.7	**-1.7**
ITGB4BP	integrin beta 4 binding protein			-1.2								-1.4	**-1.4**
ITK	IL2-inducible T-cell kinase	1.5	**1.5**	1.7	**1.7**								
ITM1	STT3, subunit of the oligosaccharyltransferase complex, homolog A (S. cerevisiae)	-1.2		-1.3	**-1.3**	-1.3	**-1.3**			-1.3		-1.4	**-1.4**
ITM2A	integral membrane protein 2A			-1.4				64.4	**64.4**			-138.3	**-138.3**
ITM2B	integral membrane protein 2B			1.8	**1.8**	1.3		1.7	**1.7**	2.0	**2.0**	1.9	**1.9**
ITPA	inosine triphosphatase (nucleoside triphosphate pyrophosphatase)			-1.3	**-1.3**								
ITPK1	inositol 1,3,4-triphosphate 5/6 kinase					-1.5							
ITPKB	inositol 1,4,5-trisphosphate 3-kinase B							-4.6	**-4.6**			1.7	**1.7**
ITPR1	inositol 1,4,5-triphosphate receptor, type 1	1.9		2.7	**2.7**			3.8	**3.8**	3.5	**2.4**	-18.1	**-18.1**
ITPR2	inositol 1,4,5-triphosphate receptor, type 2	1.5	**1.5**	1.7	**1.7**	1.5	**1.5**	4.3	**4.3**	1.8	**1.8**	-2.4	**-2.4**
ITPR3	inositol 1,4,5-triphosphate receptor, type 3			-1.6	**-1.6**	-1.4	**-1.3**						
ITSN1	intersectin 1 (SH3 domain protein)					1.2		-3.8	**-3.8**	-1.3	**-1.3**	8.7	**8.7**
ITSN2	intersectin 2							1.3		-1.5	**1.4**	1.4	**1.4**
IVD	isovaleryl Coenzyme A dehydrogenase	1.3				1.3							
IVNS1ABP	influenza virus NS1A binding protein	-1.3		-1.4				-1.3	**-1.3**	-1.3	**-1.3**	-1.9	**-1.9**
JAK1	Janus kinase 1 (a protein tyrosine kinase)	2.2	**2.2**	4.8	**4.8**	2.6	**2.6**	1.9	**1.9**	1.8	**1.8**	3.4	**3.4**
JAK2	Janus kinase 2 (a protein tyrosine kinase)			2.8	**2.8**			1.6	**1.6**	2.3	**2.3**	1.8	**1.5**
JAK3	Janus kinase 3 (a protein tyrosine kinase, leukocyte)			1.6	**1.6**	1.5				-1.5	**-1.5**		
JAM3	junctional adhesion molecule 3	2.4	**2.4**	3.7	**3.7**	3.6	**3.6**	12.9	**12.9**			-12.3	**-12.3**
JARID1A	jumonji, AT rich interactive domain 1A	1.3		1.7	**1.7**			-2.3	**-2.3**	1.2	**1.2**	3.7	**3.7**
JARID1B	jumonji, AT rich interactive domain 1B							37.6	**37.6**			-8.0	**-8.0**
JARID1C	jumonji, AT rich interactive domain 1C							-1.2		-1.3		1.3	**1.3**
JARID2	jumonji, AT rich interactive domain 2	-1.3	**-1.3**			-1.3		-1.4	**-1.4**			1.9	**1.9**
JMJD1A	jumonji domain containing 1A							1.6	**1.6**				
JMJD1C	jumonji domain containing 1C			1.9				-2.0	**-2.0**	1.9	**1.9**	3.6	**3.6**
JRK	jerky homolog (mouse)							2.1	**2.1**			-1.8	**-1.8**
JTV1	JTV1 gene	-1.9	**-1.9**	-2.1	**-2.1**	-2.1				-2.3	**-2.3**	-1.7	**-1.7**
JUN	jun oncogene	4.4	**4.4**	4.8	**4.8**			42.1	**42.1**	18.9	**18.9**	-6.2	**-6.2**
JUNB	jun B proto-oncogene					-1.4						-1.8	**-1.8**
JUND	jun D proto-oncogene							1.3	**1.3**	1.8	**1.8**	1.4	**1.4**
KAB	centrosomal protein 170kDa							3.8	**3.8**	1.2	**1.2**	-4.0	**-4.0**
K-ALPHA-1	alpha tubulin												
KARS	lysyl-tRNA synthetase	-1.3	**-1.3**			-1.5	**-1.5**			-1.6	**-1.6**	-1.7	**-1.7**
KATNA1	katanin p60 (ATPase-containing) subunit A 1							1.2		1.5	**1.5**		
KATNB1	katanin p80 (WD repeat containing) subunit B 1	-1.4		-3.5	**-1.2**	-1.5		-1.4	**-1.4**	-2.1	**-2.1**	-1.3	
KCNA5	potassium voltage-gated channel, shaker-related subfamily, member 5	1.4				1.4				5.9	**5.9**		
KCNAB2	potassium voltage-gated channel, shaker-related subfamily, beta member 2	1.3				1.6				-1.6	**-1.6**	1.3	
KCNH2	potassium voltage-gated channel, subfamily H (eag-related), member 2												
KCNN4	potassium intermediate/small conductance calcium-activated channel, subfamily N, member 4					-1.2		23.1	**23.1**	-13.3	**-13.3**	-19.5	**-19.5**
KDELR1	KDEL (Lys-Asp-Glu-Leu) endoplasmic reticulum protein retention receptor 1									-1.2		1.3	**1.3**
KDELR2	KDEL (Lys-Asp-Glu-Leu) endoplasmic reticulum protein retention receptor 2					1.3		1.3	**1.3**	-1.3		-2.0	**-2.0**
KDR	kinase insert domain receptor (a type III receptor tyrosine kinase)	1.3								1.3			
KEAP1	kelch-like ECH-associated protein 1							-1.4	**-1.4**				
KHDRBS1	KH domain containing, RNA binding, signal transduction associated 1					-1.2		-1.3	**-1.3**	-1.3	**-1.3**		
KHDRBS3	KH domain containing, RNA binding, signal transduction associated 3					-1.3		9.0	**9.0**	2.3	**2.3**	-14.3	**-14.3**
KHSRP	KH-type splicing regulatory protein (FUSE binding protein 2)			-1.4	**-1.4**	-1.4	**-1.4**			-2.3	**-2.3**	-2.2	**-2.2**
KIAA0020	KIAA0020	-1.7	**-1.7**	-3.6	**-3.6**	-2.2	**-2.2**	-1.9	**-1.9**	-2.0	**-2.0**		
KIAA0101	KIAA0101									-1.9	**-1.9**	-3.2	**-3.2**
KIAA0133	KIAA0133	-1.5		-1.4	**-1.4**	-1.3		-1.5	**-1.5**	-1.4	**-1.4**	-1.6	**-1.6**
KIAA0143	KIAA0143 protein							1.9	**1.9**	1.4	**1.4**	-1.5	**-1.5**

KIAA0247	KIAA0247	1.4		1.4				1.7	**1.7**			-1.4	**-1.4**
KIAA0310	KIAA0310							1.5	**1.5**			-1.3	**-1.3**
KIAA0368	KIAA0368	-1.3				1.5		1.8	**1.8**	1.8	**1.8**	-1.4	**-1.4**
KIAA0514	G protein regulated inducer of neurite outgrowth 2			1.2	**1.2**	1.5							
KIAA0685	SAPS domain family, member 2	1.2								2.1	**2.1**	2.3	**2.3**
KIAA0828	adenosylhomocysteinase 3							1.9	**1.9**				
KIAA0922	KIAA0922	1.5		1.9	**1.9**	1.5		-2.6	**-2.6**			1.9	**1.9**
KIAA0992	palladin, cytoskeletal associated protein	1.2	**1.2**	1.5		1.3	**1.3**	1.6	**1.6**			1.2	
KIAA0999	KIAA0999 protein	1.3		1.5		-1.2		2.2	**2.2**	-1.4	**-1.4**	-2.7	**-2.7**
KIAA1008	KIAA1008			-1.5		1.2		1.7	**1.7**			-1.9	**-1.9**
KIAA1009	KIAA1009					1.2						1.7	**1.7**
KIAA1115	SAPS domain family, member 1							1.4	**1.4**	1.3		-1.7	**-1.7**
KIAA1128	KIAA1128					-1.3	**-1.3**			1.4		1.8	**1.8**
KIAA1279	KIAA1279					-1.2		-1.3	**-1.3**			1.3	
KIAA1539	KIAA1539	1.3		1.4	**1.4**					1.7	**1.7**	2.8	**2.8**
KIAA1794	KIAA1794					-1.2		-1.9	**-1.9**	-1.3		-1.7	**-1.7**
KIDINS220	kinase D-interacting substance of 220 kDa							1.5	**1.5**	1.4	**1.4**	1.5	**1.5**
KIF11	kinesin family member 11							-1.6	**-1.6**	1.5	**1.5**	1.4	**1.4**
KIF13B	kinesin family member 13B					2.8	**2.8**						
KIF14	kinesin family member 14	1.3		1.2		1.3		-1.4	**-1.4**	1.4	**1.4**	1.8	**1.8**
KIF2	kinesin heavy chain member 2A	-1.2	**-1.2**			-1.3		-1.3	**-1.3**	-1.3		1.6	**1.6**
KIF21B	kinesin family member 21B	-1.3											
KIF22	kinesin family member 22									1.2	**1.2**		
KIF23	kinesin family member 23									1.9	**1.9**	1.2	**1.2**
KIF2C	kinesin family member 2C	1.3	**1.3**							1.3	**1.3**	-1.8	**-1.8**
KIF3B	kinesin family member 3B	1.3		1.4				1.4	**1.4**	1.5		-1.3	
KIF3C	kinesin family member 3C			1.4								2.3	**2.3**
KIF5B	kinesin family member 5B	-1.2		1.5		-1.2		1.3	**1.2**	1.5	**1.5**		
KIFAP3	kinesin-associated protein 3			1.5	**1.5**	-1.2							
KIFC1	kinesin family member C1	1.2		1.2	**1.2**	1.4		-1.6	**-1.6**				
KIR2DL4	killer cell immunoglobulin-like receptor, two domains, long cytoplasmic tail, 4			1.8	**1.8**	2.1						1.6	
KIR3DL1	killer cell immunoglobulin-like receptor, three domains, long cytoplasmic tail, 1	1.8	**1.8**	2.1	**2.1**	3.3	**3.3**						
KIR3DL2	killer cell immunoglobulin-like receptor, three domains, long cytoplasmic tail, 2	1.8	**1.8**	2.0	**2.0**	3.0	**3.0**	-1.3		1.5	**1.5**	1.3	
KIR3DL3	killer cell immunoglobulin-like receptor, three domains, long cytoplasmic tail, 3	1.6	**1.6**	1.3		2.8	**2.8**						
KLF10	Kruppel-like factor 10			-1.5		-1.5		-2.3	**-2.3**	2.4	**2.4**	1.9	**1.9**
KLF6	Kruppel-like factor 6	1.5	**1.5**					1.8	**1.8**	7.2	**7.2**	3.5	**3.5**
KLHDC3	kelch domain containing 3							-1.3		-1.5	**-1.5**		
KLRC3	killer cell lectin-like receptor subfamily C, member 3					1.8							
KNS2	kinesin 2	1.2				1.3				1.8	**1.8**	1.7	**1.7**
KNTC1	kinetochore associated 1	1.2						-1.5	**-1.5**			-1.3	**-1.3**
KNTC2	kinetochore associated 2	1.4		1.3				-1.3	**-1.3**	1.9	**1.9**		
KPNA2	karyopherin alpha 2 (RAG cohort 1, importin alpha 1)							1.3	**1.3**	1.5	**1.5**		
KPNA3	karyopherin alpha 3 (importin alpha 4)	-1.3						-1.5	**-1.5**	-1.3	**-1.3**	-1.8	**-1.8**
KPNA6	karyopherin alpha 6 (importin alpha 7)							2.0	**2.0**	1.4		-2.1	**-2.1**
KPNB1	karyopherin (importin) beta 1			-1.2		-1.7		1.6	**1.5**	-1.6	**-1.6**	-2.8	**-2.8**
KRAS	v-Ki-ras2 Kirsten rat sarcoma viral oncogene homolog	1.2						-2.1	**-2.1**	13.3	**13.3**	13.4	**13.4**
KRIT1	KRIT1, ankyrin repeat containing	1.3		1.4	**1.4**			-1.4		1.5		1.9	**1.3**
KRT10	keratin 10 (epidermolytic hyperkeratosis; keratosis palmaris et plantaris)							-2.8	**-2.8**	1.2	**1.2**	2.1	**2.1**
KRT8	keratin 8	1.2						-1.4	**-1.4**			1.6	**1.6**
KTN1	kinectin 1 (kinesin receptor)					-1.4		1.8	**1.8**			-1.4	**-1.4**
KYNU	kynureninase (L-kynurenine hydrolase)			-1.4				14.0	**14.0**				
LAGE3	L antigen family, member 3			-1.4	**-1.4**	-1.2		1.2		-1.9	**-1.9**	-3.3	**-3.3**
LAIR1	leukocyte-associated immunoglobulin-like receptor 1	1.7	**1.7**	2.1	**2.1**	1.6	**1.6**	6.7	**6.7**	2.7	**2.7**	-1.9	**-1.9**
LAMP1	lysosomal-associated membrane protein 1							1.8	**1.8**	1.5	**1.5**	-1.5	**-1.4**
LAMP2	lysosomal-associated membrane protein 2			1.4	**1.4**			2.1	**2.1**	1.7	**1.7**	-2.3	**-2.3**
LANCL1	LanC lantibiotic synthetase component C-like 1 (bacterial)			1.3	**1.3**			-1.7	**-1.7**			1.4	**1.4**
LAP1B	torsin A interacting protein 1			1.3	**1.2**	-1.2		1.4	**1.4**	-1.4		-2.0	**-2.0**
LAPTM4A	lysosomal-associated protein transmembrane 4 alpha												

LAPTM5	lysosomal associated multispanning membrane protein 5							-4.3	**-4.3**	2.0	**2.0**	10.9	**10.9**
LARP1	La ribonucleoprotein domain family, member 1	-1.4		-1.7		-1.9				-2.0	**-2.0**	-2.2	**-2.2**
LARS2	leucyl-tRNA synthetase 2, mitochondrial							-1.2	**-1.2**	-1.5	**-1.5**	-1.4	**-1.4**
LAS1L	LAS1-like (S. cerevisiae)			-1.3		-1.4	**-1.4**					-2.0	**-2.0**
LASP1	LIM and SH3 protein 1							1.6	**1.6**				
LASS1	LAG1 homolog, ceramide synthase 1 (S. cerevisiae)					1.6							
LAT	linker for activation of T cells	1.4	**1.4**	1.3	**1.3**	1.2							
LBR	lamin B receptor									1.4	**1.4**		
LCK	lymphocyte-specific protein tyrosine kinase					-1.7	**-1.7**	-49.3	**-49.3**			18.5	**18.5**
LCP1	lymphocyte cytosolic protein 1 (L-plastin)					-1.3		-4.1	**-4.1**	-2.5	**-2.5**	7.2	**7.2**
LCP2	lymphocyte cytosolic protein 2 (SH2 domain containing leukocyte protein of 76kDa)					1.5		1.6	**1.6**	-2.4	**-2.4**	-2.2	**-2.2**
LCT	lactase	-1.4		-1.4		-2.4							
LDB1	LIM domain binding 1			1.3	**1.3**			1.7	**1.7**	-1.2	**-1.2**	-1.5	**-1.5**
LDHA	lactate dehydrogenase A			-1.4						-1.7	**-1.7**	-1.8	**-1.8**
LDHB	lactate dehydrogenase B									-1.6	**-1.6**		
LDLR	low density lipoprotein receptor (familial hypercholesterolemia)			-1.9	**-1.9**			-3.6	**-3.6**	-1.3	**-1.3**	3.0	**3.0**
LEF1	lymphoid enhancer-binding factor 1	1.5	**1.5**	1.7	**1.7**	1.7		-288.8	**-288.8**	-2.2	**-1.3**	826.7	**826.7**
LEPR	leptin receptor			1.4		1.4		1.8	**1.8**	2.2	**2.2**	4.6	**4.6**
LETMD1	LETM1 domain containing 1	1.2				1.2		3.1	**3.1**			-7.5	**-7.5**
LGALS1	lectin, galactoside-binding, soluble, 1 (galectin 1)	1.6		1.7	**1.7**	-1.3		-22.2	**-22.2**	2.9	**2.9**	82.1	**82.1**
LGALS3BP	lectin, galactoside-binding, soluble, 3 binding protein							-4.3	**-4.3**	-2.3	**-2.3**	2.8	**2.8**
LGALS9	lectin, galactoside-binding, soluble, 9 (galectin 9)	-1.5	**-1.5**	-1.4	**-1.4**	-2.1	**-2.1**	1.4		-1.5		-2.4	**-2.4**
LGMN	legumain	-1.6	**-1.6**	-1.8	**-1.8**	-1.4							
LIG1	ligase I, DNA, ATP-dependent			-1.7	**-1.7**							-5.5	**-5.5**
LIG3	ligase III, DNA, ATP-dependent			-1.4	**-1.4**							-1.4	**-1.4**
LIG4	ligase IV, DNA, ATP-dependent	-1.4		1.5		-1.6		1.9	**1.9**	1.8	**1.8**		
LIMK2	LIM domain kinase 2	-1.4	**-1.4**	-2.6	**-2.6**	-1.6	**-1.6**						
LIMS1	LIM and senescent cell antigen-like domains 1			-1.4				1.3	**1.3**	1.4	**1.3**	3.7	**3.7**
LIPA	lipase A, lysosomal acid, cholesterol esterase (Wolman disease)							1.2	**1.2**	-2.0	**-2.0**		
LMAN1	lectin, mannose-binding, 1	-1.5	**-1.5**			-1.5		2.1	**1.6**			-15.2	**-15.2**
LMNB1	lamin B1			1.3		-1.2		-2.0	**-2.0**			-1.2	
LMNB2	lamin B2			-1.4	**-1.4**							-1.5	**-1.5**
LMO2	LIM domain only 2 (rhombotin-like 1)	-1.7		-2.0	**-2.0**			7.9	**7.9**	4.4	**4.4**	-6.9	**-6.9**
LMO4	LIM domain only 4			1.5				-3.8	**-3.8**	-1.3		1.9	**1.9**
LNK	SH2B adaptor protein 3							1.3	**1.3**	-1.2	**-1.2**	-2.8	**-2.8**
LNPEP	leucyl/cystinyl aminopeptidase			2.9	**2.9**			-2.2	**-2.2**	-2.0		2.9	**2.9**
LOC161527	hypothetical protein LOC161527			-1.3								1.8	**1.8**
LOC162427	hypothetical protein LOC162427	1.4										1.5	**1.5**
LOC51035	unknown protein LOC51035					1.5	**1.5**	1.3	**1.3**	1.4		1.2	
LOC93081	chromosome 13 open reading frame 27			-1.4	**-1.4**	-1.4		2.0	**2.0**			-1.4	**-1.4**
LPIN1	lipin 1	1.3	**1.3**	1.3				-1.6	**-1.6**			2.3	**2.3**
LPO	lactoperoxidase	1.3				1.9							
LPXN	leupaxin							-3.2	**-3.2**	-1.7	**-1.7**	1.7	**1.7**
LRBA	LPS-responsive vesicle trafficking, beach and anchor containing	1.2		1.6				1.4	**1.4**	-2.1	**-2.1**	-1.6	**-1.6**
LRCH4	leucine-rich repeats and calponin homology (CH) domain containing 4	1.3				1.7		-1.9	**-1.9**	1.9	**1.9**	4.1	**4.1**
LRMP	lymphoid-restricted membrane protein							-18.6	**-18.6**	-1.4	**-1.4**	7.8	**7.8**
LRP4	low density lipoprotein receptor-related protein 4					-1.4						-16.6	**-16.6**
LRP8	low density lipoprotein receptor-related protein 8, apolipoprotein e receptor	-2.2	**-2.2**	-3.6		-2.8	**-2.8**	-1.6	**-1.6**	-2.1	**-2.1**	-2.6	**-2.5**
LRPAP1	low density lipoprotein receptor-related protein associated protein 1			1.3				3.2	**3.2**			-2.1	**-2.1**
LRPPRC	leucine-rich PPR-motif containing	-1.8	**-1.8**	-1.4	**-1.4**	-1.7	**-1.7**	1.7	**1.7**	-5.1	**-5.1**	-2.8	**-2.8**
LRRFIP1	leucine rich repeat (in FLII) interacting protein 1					-1.4		-6.0	**-6.0**	2.5	**2.5**	11.3	**11.3**
LSM1	LSM1 homolog, U6 small nuclear RNA associated (S. cerevisiae)							2.2	**2.2**			-1.6	
LSM2	LSM2 homolog, U6 small nuclear RNA associated (S. cerevisiae)			-1.2		-1.2						1.3	**-1.3**
LSM3	LSM3 homolog, U6 small nuclear RNA associated (S. cerevisiae)					-1.3		-1.3					
LSM4	LSM4 homolog, U6 small nuclear RNA associated (S. cerevisiae)	1.3		-1.3				-1.6	**-1.6**	-1.4	**-1.4**	-1.9	**-1.9**
LSM5	LSM5 homolog, U6 small nuclear RNA associated (S. cerevisiae)							2.9	**2.9**	1.3		-4.5	**-4.5**
LSM6	LSM6 homolog, U6 small nuclear RNA associated (S. cerevisiae)			-1.3		-1.5		-1.5		-1.4	**-1.4**	2.2	**2.2**
LSM7	LSM7 homolog, U6 small nuclear RNA associated (S. cerevisiae)	-1.2		-1.4	**-1.4**	-1.4		-1.5	**-1.5**	-1.5	**-1.5**	-1.4	**-1.4**

LSP1	lymphocyte-specific protein 1												
LSS	lanosterol synthase (2,3-oxidosqualene-lanosterol cyclase)	-1.5	**-1.5**	-3.4	**-3.4**	-1.3				-1.2	**-1.2**	1.8	
LST1	leukocyte specific transcript 1					-1.9							
LTA4H	leukotriene A4 hydrolase			1.3	**1.3**			2.6	**2.6**	-1.5		-2.9	**-2.9**
LTB	lymphotoxin beta (TNF superfamily, member 3)	-1.6		-1.7	**-1.7**	-1.8				-1.8			
LTBP4	latent transforming growth factor beta binding protein 4	1.4				1.7		-1.5				1.4	
LTK	leukocyte tyrosine kinase							-2.3	**-2.3**			2.4	**2.4**
LY6E	lymphocyte antigen 6 complex, locus E	-1.3		-1.8				2.0	**2.0**			-12.6	**-12.6**
LY9	lymphocyte antigen 9			-1.3				-9.6	**-9.6**	2.2	**2.2**	2.7	**2.7**
LY96	lymphocyte antigen 96	3.5						-9.8	**-9.8**	3.1	**3.1**	24.5	**24.5**
LYL1	lymphoblastic leukemia derived sequence 1					1.4	**1.4**	3.5	**3.5**	-1.6		-6.7	**-6.7**
LYPLA1	lysophospholipase I							-1.3	**-1.3**	1.3	**1.3**	1.2	**1.2**
LYPLA2	lysophospholipase II					1.3		-1.5		-1.5	**-1.5**	-1.4	
LZTR1	leucine-zipper-like transcription regulator 1	1.2										1.2	
M11S1	GPI-anchored membrane protein 1	-1.2		-1.3		-1.3		-2.0	**-2.0**	-1.3	**-1.3**	-1.5	**-1.5**
M6PR	mannose-6-phosphate receptor (cation dependent)					1.3				-2.3	**-2.3**	-2.2	**-2.2**
M6PRBP1	mannose-6-phosphate receptor binding protein 1	1.4		1.6						1.3		1.4	
MAC30	transmembrane protein 97	-1.3	**-1.3**	-2.3	**-2.3**	-1.4		1.4	**1.4**	-3.0	**-3.0**	-3.2	**-3.2**
MACF1	microtubule-actin crosslinking factor 1	1.2		1.6	**1.6**			-1.5	**-1.5**	-1.3	**-1.3**	2.7	**2.7**
MAD2L1	MAD2 mitotic arrest deficient-like 1 (yeast)			-1.5		-1.4		-2.0	**-2.0**	-1.3			
MADD	MAP-kinase activating death domain											1.4	**1.4**
MAEA	macrophage erythroblast attacher	1.3	**1.3**	1.3	**1.3**					1.6	**1.6**		
MAG	myelin associated glycoprotein	1.2				1.8		-40.4	**-40.4**	-2.3	**-2.3**	2.0	**2.0**
MAGED1	melanoma antigen family D, 1			1.4				2.4	**2.4**	-2.1	**-2.1**	-4.1	**-4.1**
MAGOH	mago-nashi homolog, proliferation-associated (Drosophila)					-1.3		-1.2	**-1.2**			-1.5	**-1.5**
MALT1	mucosa associated lymphoid tissue lymphoma translocation gene 1							-1.7	**-1.7**	-1.7	**-1.7**	2.2	**2.2**
MAML1	mastermind-like 1 (Drosophila)			1.3									
MAN1A1	mannosidase, alpha, class 1A, member 1			1.4						4.4	**4.4**	5.5	**5.5**
MAN2A1	mannosidase, alpha, class 2A, member 1					1.2		2.5	**2.5**	1.7	**1.7**	-1.9	**-1.9**
MAN2A2	mannosidase, alpha, class 2A, member 2			-1.4				8.4	**8.4**	1.3	**1.3**	-4.8	**-4.8**
MAN2B1	mannosidase, alpha, class 2B, member 1							2.4	**2.4**	-1.4		-2.9	**-2.9**
MAN2C1	mannosidase, alpha, class 2C, member 1					1.2		1.7				1.9	
MAP1A	microtubule-associated protein 1A	2.0	**2.0**	1.6	**1.6**	1.7	**1.7**					10.8	**10.8**
MAP1LC3B	microtubule-associated protein 1 light chain 3 beta	1.2		1.5	**1.5**	1.3				3.2	**3.2**	3.2	**3.2**
MAP2K1	mitogen-activated protein kinase kinase 1	1.5	**1.5**	1.7	**1.7**	1.7	**1.7**	1.5	**1.5**	1.9	**1.9**	2.1	**2.1**
MAP2K2	mitogen-activated protein kinase kinase 2							1.3	**1.3**			-1.6	**-1.6**
MAP2K3	mitogen-activated protein kinase kinase 3	1.3		1.4				-2.3	**-2.3**	-1.2		1.9	**1.9**
MAP2K4	mitogen-activated protein kinase kinase 4					-1.6		-1.4	**-1.4**			1.7	**1.7**
MAP2K5	mitogen-activated protein kinase kinase 5											2.1	**2.1**
MAP2K6	mitogen-activated protein kinase kinase 6	1.2						-2.5	**-2.5**	-1.6			
MAP3K1	mitogen-activated protein kinase kinase kinase 1			1.2				-1.3	**-1.3**			-1.5	**-1.5**
MAP3K11	mitogen-activated protein kinase kinase kinase 11							1.3	**1.3**			-1.7	**-1.7**
MAP3K12	mitogen-activated protein kinase kinase kinase 12			-1.2				2.1	**2.1**	-1.3		-2.1	**-2.1**
MAP3K4	mitogen-activated protein kinase kinase kinase 4												
MAP3K7	mitogen-activated protein kinase kinase kinase 7			1.3				1.3	**1.3**	-1.2		-1.3	
MAP3K7IP1	mitogen-activated protein kinase kinase kinase 7 interacting protein 1											1.4	
MAP3K7IP2	mitogen-activated protein kinase kinase kinase 7 interacting protein 2			1.4	**1.4**			-1.4	**-1.4**	1.6	**1.5**	2.1	**2.1**
MAP4	microtubule-associated protein 4	-1.3	**-1.3**	-1.8	**-1.8**	-1.7				-1.4		1.6	**1.6**
MAP4K1	mitogen-activated protein kinase kinase kinase kinase 1							-2.6	**-2.6**	-2.7	**-2.7**	2.4	**2.4**
MAP4K2	mitogen-activated protein kinase kinase kinase kinase 2			-1.4								-5.4	**-5.4**
MAP4K4	mitogen-activated protein kinase kinase kinase kinase 4					-1.4		-1.5	**-1.5**	1.7	**1.7**	1.7	**1.7**
MAP4K5	mitogen-activated protein kinase kinase kinase kinase 5							1.9	**1.9**	2.2	**2.2**	1.4	
MAPK1	mitogen-activated protein kinase 1			1.7				-1.4	**-1.4**	2.3	**2.3**	2.0	**2.0**
MAPK14	mitogen-activated protein kinase 14			1.9	**1.6**	1.4	**1.4**	4.1	**4.1**	-1.7		-2.1	**-2.1**
MAPK3	mitogen-activated protein kinase 3											4.6	**4.6**
MAPK6	mitogen-activated protein kinase 6	-1.5	**-1.5**	-2.0	**-2.0**	-1.8	**-1.8**	1.2				-1.4	**-1.4**
MAPK7	mitogen-activated protein kinase 7					1.3							
MAPK9	mitogen-activated protein kinase 9	-1.5	**-1.5**	-1.3		-1.9	**-1.9**					-1.4	**-1.4**

MAPKAPK2	mitogen-activated protein kinase-activated protein kinase 2	1.3	**1.3**	1.5	**1.3**	1.5				1.5	**1.5**	1.4	**1.3**
MAPKAPK3	mitogen-activated protein kinase-activated protein kinase 3	-1.4	**-1.4**	-1.6	**-1.6**	-1.5		10.5	**10.5**	-1.5	**-1.5**	-20.4	**-20.4**
MAPKAPK5	mitogen-activated protein kinase-activated protein kinase 5			-1.6		-1.3		-1.6	**-1.6**			-1.4	
MAPRE1	microtubule-associated protein, RP/EB family, member 1			1.3	**1.3**			-1.4	**-1.4**	1.5	**1.5**	1.6	**1.6**
MAPRE2	microtubule-associated protein, RP/EB family, member 2	1.4		1.4				-3.4	**-3.4**	-2.5	**-2.5**	3.0	**3.0**
MARCH7	membrane-associated ring finger (C3HC4) 7	1.3						2.3	**2.3**	2.1	**2.1**	1.2	
MARCKSL1	MARCKS-like 1	-1.2		-1.4		-1.6	**-1.6**	-1.6	**-1.6**	-1.4	**-1.4**		
MARK3	MAP/microtubule affinity-regulating kinase 3							1.5	**1.5**	1.3		-1.3	
MARS	methionine-tRNA synthetase	-1.5		-1.6	**-1.6**	-1.5		-1.5	**-1.5**	-2.2	**-2.2**	-2.2	**-2.2**
MAT2A	methionine adenosyltransferase II, alpha	-1.8						-1.4					
MATR3	matrin 3					-1.5		1.8		-14.6	**-14.6**	-10.9	**-10.9**
MAX	MYC associated factor X			1.5				-1.2	**-1.2**	-1.7	**-1.7**		
MAZ	MYC-associated zinc finger protein (purine-binding transcription factor)	-1.5		-1.8				-3.2	**-3.2**	-1.7	**-1.7**	1.7	**1.7**
MB	myoglobin					24.0	**24.0**						
MBD1	methyl-CpG binding domain protein 1	1.2						-1.4	**1.3**	-1.2		-1.8	**-1.8**
MBD2	methyl-CpG binding domain protein 2					-1.4		-1.6	**-1.6**			1.4	**1.3**
MBD3	methyl-CpG binding domain protein 3			-1.3				-1.6	**-1.6**	-2.7	**-1.4**	-1.7	
MBD4	methyl-CpG binding domain protein 4	1.2						1.7	**1.7**			-2.2	**-2.2**
MBNL1	muscleblind-like (Drosophila)					-1.3		-4.4	**-4.4**	2.0	**2.0**	9.2	**9.2**
MBTPS1	membrane-bound transcription factor peptidase, site 1					-1.2		1.2				-1.6	**-1.6**
MC2R	melanocortin 2 receptor (adrenocorticotropic hormone)												
MC5R	melanocortin 5 receptor			1.4	**1.4**								
MCCC2	methylcrotonoyl-Coenzyme A carboxylase 2 (beta)	-1.2						1.4	**1.4**	-1.5	**-1.5**	-2.1	**-2.1**
MCFD2	multiple coagulation factor deficiency 2							1.6	**1.6**			-1.7	**-1.7**
MCL1	myeloid cell leukemia sequence 1 (BCL2-related)	1.2	**1.2**	1.5	**1.5**			1.4	**1.4**	1.7	**1.7**	1.3	**1.3**
MCM2	MCM2 minichromosome maintenance deficient 2, mitotin (S. cerevisiae)			-1.3	**-1.3**	-1.2				-3.1	**-3.1**	-4.2	**-4.2**
MCM3	MCM3 minichromosome maintenance deficient 3 (S. cerevisiae)					-1.3	**-1.3**	-1.4	**-1.4**	-3.1	**-3.1**	-4.2	**-4.2**
MCM3AP	MCM3 minichromosome maintenance deficient 3 (S. cerevisiae) associated protein	1.3	**1.3**									1.9	**1.9**
MCM4	MCM4 minichromosome maintenance deficient 4 (S. cerevisiae)					-1.5		-2.1	**-2.1**	-3.3	**-3.3**	-6.9	**-6.9**
MCM5	MCM5 minichromosome maintenance deficient 5, cell division cycle 46 (S. cerevisiae)							-1.5	**-1.5**	-1.5	**-1.5**	-3.2	**-3.2**
MCM6	minichromosome maintenance deficient 6 homolog (S. cerevisiae)			-1.3	**-1.3**	-1.4		1.3	**1.3**	-2.1	**-2.1**	-6.8	**-6.8**
MCM7	MCM7 minichromosome maintenance deficient 7 (S. cerevisiae)	-1.2		-1.4	**-1.4**			-1.9	**-1.9**	-1.3	**-1.3**	-2.7	**-2.7**
MCP	CD46 molecule, complement regulatory protein					-1.3		1.6	**1.6**	1.4	**1.4**		
MCRS1	microspherule protein 1							-1.7	**-1.7**				
MDC1	mediator of DNA damage checkpoint 1			1.2	**1.2**			-4.7	**-4.7**			2.0	**2.0**
MDFIC	MyoD family inhibitor domain containing			1.3				-3.1	**-3.1**	1.3		2.4	**2.4**
MDH1	malate dehydrogenase 1, NAD (soluble)							1.9	**1.9**	-1.3	**-1.3**	-2.2	**-2.2**
MDH2	malate dehydrogenase 2, NAD (mitochondrial)					-1.4		-2.3	**-2.3**	-1.5	**-1.5**	3.7	**3.7**
MDM4	Mdm4, transformed 3T3 cell double minute 4, p53 binding protein (mouse)			1.6		1.5		-2.4	**-2.4**	2.2	**2.2**	2.5	**2.5**
ME2	malic enzyme 2, NAD(+)-dependent, mitochondrial	-1.5		-1.4		-1.8	**-1.8**	1.2		-1.6	**-1.6**	-2.2	**-2.2**
MECP2	methyl CpG binding protein 2 (Rett syndrome)	1.3		1.3									
MED12	mediator of RNA polymerase II transcription, subunit 12 homolog (S. cerevisiae)							1.4	**1.4**				
MED6	mediator of RNA polymerase II transcription, subunit 6 homolog (S. cerevisiae)	1.2		1.4		-1.3		-1.4	**-1.4**			1.3	
MED8	mediator of RNA polymerase II transcription, subunit 8 homolog (S. cerevisiae)					1.3		1.7	**1.7**	-1.7		-1.2	
MEF2A	MADS box transcription enhancer factor 2, polypeptide A (myocyte enhancer factor 2A)					-1.3		-3.6	**-3.6**	1.7	**1.7**	11.0	**11.0**
MEF2C	MADS box transcription enhancer factor 2, polypeptide C (myocyte enhancer factor 2C)	-1.6		-1.6				-3.5	**-3.5**	1.6	**-1.5**	3.6	**3.6**
MEF2D	MADS box transcription enhancer factor 2, polypeptide D (myocyte enhancer factor 2D)							-5.7	**-5.7**	1.8		2.0	**2.0**
MEIS2	Meis1, myeloid ecotropic viral integration site 1 homolog 2 (mouse)	1.4				-1.5		3.6	**3.6**	-2.1	**-2.1**	-4.4	**-4.4**
MELK	maternal embryonic leucine zipper kinase	1.4	**1.4**			-1.3		-1.3	**-1.3**	1.4	**1.4**		
MEN1	multiple endocrine neoplasia I			-1.4	**-1.4**			1.3	**1.3**	-1.5	**-1.5**	-1.3	**-1.3**
MEOX2	mesenchyme homeobox 2												
MEP50	WD repeat domain 77	-1.7	**-1.7**	-1.4	**-1.4**	-1.8		-1.8	**-1.8**	-1.5	**-1.5**	-1.4	**-1.4**
METAP2	methionyl aminopeptidase 2												
METAP2	--	-1.2						1.4	**1.4**	-1.3	**-1.3**	-1.7	**-1.7**
METTL1	methyltransferase like 1	-1.3	**-1.3**										
MFAP1	microfibrillar-associated protein 1	1.2		1.2	**1.2**			-1.6	**-1.6**	-1.4	**-1.4**		
MFAP4	microfibrillar-associated protein 4					2.2							
MFGE8	milk fat globule-EGF factor 8 protein	1.3		2.3	**2.3**	1.3		-1.5		1.7			

MFHAS1	malignant fibrous histiocytoma amplified sequence 1			1.3		-1.2		1.9		-1.5	**-1.5**	-4.1	**-4.1**
MFN1	mitofusin 1			1.4	**1.4**			1.7				1.9	**1.9**
MFN2	mitofusin 2					-1.2				1.6		-1.6	**-1.6**
MFNG	MFNG O-fucosylpeptide 3-beta-N-acetylglucosaminyltransferase			-1.3	**-1.3**			3.0	**3.0**	-1.8	**-1.8**	-9.2	**-9.2**
MGA	MAX gene associated			-1.3		-1.2		3.0	**3.0**	-1.8	**-1.8**	-7.2	**-7.2**
MGAT1	mannosyl (alpha-1,3-)-glycoprotein beta-1,2-N-acetylglucosaminyltransferase					1.2		-1.3					
MGC17330	HGFL gene	7.3	**7.3**	6.8	**6.8**	5.5	**5.5**	34.8	**34.8**	7.3	**7.3**	12.3	**12.3**
MGC5508	transmembrane protein 109	-1.4	**-1.4**	-1.5	**-1.5**	-1.6	**-1.6**	-1.4	**-1.4**	-1.7	**-1.7**	-1.5	**-1.5**
MGEA5	meningioma expressed antigen 5 (hyaluronidase)			1.6		1.5		1.7	**1.7**	1.3	**1.3**	1.9	**1.9**
MGMT	O-6-methylguanine-DNA methyltransferase									-1.9	**-1.9**		
MIB1	mindbomb homolog 1 (Drosophila)					1.2		4.1	**4.1**	1.2		-7.8	**-7.8**
MICB	MHC class I polypeptide-related sequence B					-1.3		-1.4	**-1.4**				
MIF	macrophage migration inhibitory factor (glycosylation-inhibiting factor)	-1.2								-1.9	**-1.9**	-2.6	**-2.6**
MINA	MYC induced nuclear antigen			-1.7				-1.2	**-1.2**	-1.7	**-1.7**	-1.9	**-1.9**
MINPP1	multiple inositol polyphosphate histidine phosphatase, 1	1.2		1.4				1.6	**1.6**			-1.7	**-1.7**
MIPEP	mitochondrial intermediate peptidase												
MIR16	membrane interacting protein of RGS16			1.3		-1.3		2.1	**2.1**	-1.4	**-1.4**	-2.9	**-2.9**
MIZF	MBD2-interacting zinc finger												
MKI67	antigen identified by monoclonal antibody Ki-67	1.3		1.3				-2.2	**-2.2**	2.1	**2.1**	2.2	**2.2**
MKL1	megakaryoblastic leukemia (translocation) 1	1.4		1.4	**1.4**			1.5	**1.5**	-1.5	**-1.5**	-1.6	**-1.6**
MKNK1	MAP kinase interacting serine/threonine kinase 1	1.4		1.3		1.9	**1.9**	1.7	**1.3**			1.3	**1.3**
MKRN1	makorin, ring finger protein, 1	1.2						-1.5	**-1.5**			2.1	**2.1**
MLC1	megalencephalic leukoencephalopathy with subcortical cysts 1					-1.5	**-1.5**	1.7	**1.7**			-5.1	**-5.1**
MLF2	myeloid leukemia factor 2	-1.3											
MLH1	mutL homolog 1, colon cancer, nonpolyposis type 2 (E. coli)									-1.6	**-1.6**	-1.5	**-1.5**
MLH3	mutL homolog 3 (E. coli)	-1.4				-1.4		2.2	**2.2**	-1.3	**-1.3**	-2.4	**-2.4**
MLL	myeloid/lymphoid or mixed-lineage leukemia (trithorax homolog, Drosophila)							1.8	**1.8**	-1.8	**-1.8**	2.0	**2.0**
MLL4	myeloid/lymphoid or mixed-lineage leukemia 4	1.2											
MLLT10	myeloid/lymphoid or mixed-lineage leukemia (trithorax homolog, Drosophila); translocated to, 10					-1.7		2.7	**2.7**	1.2		-2.7	**-2.2**
MLLT7	myeloid/lymphoid or mixed-lineage leukemia (trithorax homolog, Drosophila); translocated to, 7					5.6	**5.6**						
MLX	MAX-like protein X	-1.3	**-1.3**	-1.2		-1.5		1.2					
MMD	monocyte to macrophage differentiation-associated			-1.3		-1.3				-2.6	**-2.6**	-2.7	**-2.7**
MME	membrane metallo-endopeptidase (neutral endopeptidase, enkephalinase)	1.4	**1.4**			1.6				-1.3	**-1.3**		
MMP11	matrix metallopeptidase 11 (stromelysin 3)	1.3											
MMP14	matrix metallopeptidase 14 (membrane-inserted)					1.7				-1.3	**-1.3**		
MMP25	matrix metallopeptidase 25												
MMS19L	MMS19-like (MET18 homolog, S. cerevisiae)					-1.3	**-1.3**	1.7	**1.7**			-1.4	**-1.4**
MNAT1	menage a trois homolog 1, cyclin H assembly factor (Xenopus laevis)	-1.4		1.2		-1.5	**-1.5**						
MNT	MAX binding protein	-1.5		-1.5	**-1.5**	-1.6		-1.5	**-1.5**				
MOAP1	modulator of apoptosis 1	1.9	**1.9**	1.4						1.5	**1.5**	1.5	**1.5**
MOBK1B	MOB1, Mps One Binder kinase activator-like 1B (yeast)							-1.6	**-1.6**	-1.2		1.4	**1.4**
MONDOA	MLX interacting protein	1.3		1.4				1.5	**1.5**	1.7	**1.7**	-1.2	
MORF4L2	mortality factor 4 like 2							1.5	**1.2**				
MPG	N-methylpurine-DNA glycosylase							-4.1	**-4.1**			4.1	**4.1**
MPHOSPH1	M-phase phosphoprotein 1							1.9	**1.9**			-3.2	**-3.2**
MPHOSPH10	M-phase phosphoprotein 10 (U3 small nucleolar ribonucleoprotein)	-1.4		-1.5	**-1.5**	-1.5				-1.3	**-1.3**	-1.3	**-1.3**
MPHOSPH6	M-phase phosphoprotein 6	-1.2				-1.4		-1.5	**-1.3**	-1.9	**-1.9**	-2.3	**-2.3**
MPHOSPH9 (	M-phase phosphoprotein 9	1.4						1.2		-1.3	**-1.3**	-1.4	**-1.4**
MPI	mannose phosphate isomerase	-1.3	**-1.3**	-1.7	**-1.7**	-1.4							
MPP1	membrane protein, palmitoylated 1, 55kDa	1.4				1.3		-1.9	**-1.9**	-1.3	**-1.3**		
MPP2	membrane protein, palmitoylated 2 (MAGUK p55 subfamily member 2)			-1.2		1.5							
MPP6	membrane protein, palmitoylated 6 (MAGUK p55 subfamily member 6)	-1.6		-1.2		-1.7		38.6	**38.6**			-21.9	**-21.9**
MPST	mercaptopyruvate sulfurtransferase	-1.4								1.3	**1.3**		
MPV17	MpV17 mitochondrial inner membrane protein									-1.4	**-1.4**	-1.9	**-1.9**
MPZ	myelin protein zero (Charcot-Marie-Tooth neuropathy 1B)			1.2									
MPZL1	myelin protein zero-like 1			1.7		1.4		-7.5	**-7.5**	-1.8	**-1.8**	9.8	**9.8**
MR1	major histocompatibility complex, class I-related	1.2				1.4		-1.9				3.5	**3.5**
MRC2	mannose receptor, C type 2							2.0	**2.0**			-8.0	**-8.0**

MRCL3	myosin regulatory light chain MRCL3			1.4	**1.4**			-2.0	**-2.0**	2.2	**2.2**	5.8	**5.8**
MRE11A	MRE11 meiotic recombination 11 homolog A (S. cerevisiae)			1.4				-1.3				-1.7	**-1.7**
M-RIP	myosin phosphatase-Rho interacting protein							-5.4	**-5.4**	1.2	**1.2**	26.4	**26.4**
MRLC2	myosin regulatory light chain MRLC2												
MRPL12	mitochondrial ribosomal protein L12	-1.3		-2.0	**-2.0**					-1.7	**-1.7**	-3.2	**-3.2**
MRPS12	mitochondrial ribosomal protein S12	-1.4	**-1.4**	-1.3	**-1.2**	-2.0	**-1.7**	-1.5	**-1.5**			-2.0	**-1.7**
MRPS27	mitochondrial ribosomal protein S27					-1.4				-1.5	**-1.5**	-1.5	**-1.5**
MSC	musculin (activated B-cell factor-1)					1.5							
MSH2	mutS homolog 2, colon cancer, nonpolyposis type 1 (E. coli)			-1.3	**-1.3**	-1.5	**-1.5**	-1.5	**-1.5**	-1.6	**-1.6**	-1.8	**-1.8**
MSH3	mutS homolog 3 (E. coli)	1.3						-1.3		-1.5	**-1.5**	1.7	**1.7**
MSH6	mutS homolog 6 (E. coli)	1.5	**-1.2**	-1.3		-1.7	**-1.7**	-2.5	**-2.5**	-2.7	**-2.7**	-2.4	**-2.4**
MSN	moesin	1.2		1.3		1.2		1.4	**1.4**	1.4	**1.2**	1.7	**1.7**
MST1	macrophage stimulating 1 (hepatocyte growth factor-like)	1.4											
MSX1	msh homeobox 1	1.5	**1.5**			1.3		-1.4		-1.6	**-1.6**	-1.4	**-1.4**
MT1A	metallothionein 1A (functional)	1.3		1.3	**1.3**	2.2							
MT1B	metallothionein 1B (functional)	1.3		1.5	**1.5**	1.6							
MT1E	metallothionein 1E (functional)			1.9	**1.9**	2.1	**2.1**	1.8	**1.8**	2.3	**2.3**	1.3	
MT1G	metallothionein 1G					1.4				1.7	**1.7**		
MT1H	metallothionein 1H	1.3		1.5	**1.5**	2.1	**2.1**	1.4	**1.4**	3.0	**3.0**	1.3	**1.3**
MT1X	metallothionein 1X	1.4		2.1	**2.1**	2.2		1.6	**1.6**	1.8	**1.8**	1.7	**1.7**
MT2A	metallothionein 2A					1.5		3.2	**3.2**	3.0	**3.0**	1.2	**1.2**
MT3	metallothionein 3 (growth inhibitory factor (neurotrophic))	1.4		1.5	**1.5**	1.7	**1.7**						
MTA1	metastasis associated 1			-1.6		-1.2		1.4	**1.4**	-1.4	**-1.4**	-1.6	**-1.6**
MTCP1	mature T-cell proliferation 1			1.3	**1.3**			-1.4	**-1.4**	-1.8		1.4	
MTF1	metal-regulatory transcription factor 1									-1.4		-1.3	
MTHFD1	methylenetetrahydrofolate dehydrogenase (NADP+ dependent) 1, methenyltetrahydrofolate cyclohydrolase, formyltetrahydrofolate synthetase	-1.4	**-1.4**	-1.9	**-1.9**	-1.5	**-1.5**	1.5	**1.5**	-2.2	**-2.2**	-5.0	**-5.0**
MTHFD2	methylenetetrahydrofolate dehydrogenase (NADP+ dependent) 2, methenyltetrahydrofolate cyclohydrolase	-2.1	**-2.1**	-2.8	**-2.8**	-1.7	**-1.7**			-2.0	**-2.0**	-2.7	**-2.7**
MTHFS	5,10-methenyltetrahydrofolate synthetase (5-formyltetrahydrofolate cyclo-ligase)					-1.7	**1.3**	-1.4	**-1.4**			1.3	
MTIF2	mitochondrial translational initiation factor 2	1.4				-1.3	**-1.3**					-1.3	**-1.3**
MTM1	myotubularin 1	1.6		2.4	**2.4**	1.5		2.5	**2.5**	1.4		-2.5	**-2.5**
MTMR2	myotubularin related protein 2							-1.2	**-1.2**	-1.3	**-1.3**	1.5	
MTMR3	myotubularin related protein 3	1.4		1.4	**1.4**			-1.4	**-1.4**	1.5	**1.5**	1.9	**1.9**
MTMR4	myotubularin related protein 4	-1.3	**-1.3**	-1.5		-1.8	**-1.8**						
MTMR6	myotubularin related protein 6							-1.4	**-1.4**	2.2	**1.7**	1.5	**1.5**
MTMR9	myotubularin related protein 9			1.3						1.3			
MTR	5-methyltetrahydrofolate-homocysteine methyltransferase	-1.2				-1.3	**-1.3**			1.4	**1.4**	-1.7	**-1.7**
MTRR	5-methyltetrahydrofolate-homocysteine methyltransferase reductase	-1.7		-1.4		-1.4				-1.2	**-1.2**	-1.8	**-1.8**
MTSS1	metastasis suppressor 1					2.0		13.4	**13.4**	6.6	**6.6**	-4.9	**-4.9**
MTX1	metaxin 1			-1.2	**-1.2**			-1.3		-1.3		-1.2	
MTX2	metaxin 2	-1.5		-1.5	**-1.5**	-1.5		1.3	**1.3**			-2.1	**-2.1**
MUC1	mucin 1, cell surface associated					1.4							
MUT	methylmalonyl Coenzyme A mutase			1.3	**1.3**			-1.6	**-1.6**			2.0	**2.0**
MUTYH	mutY homolog (E. coli)			1.4	**1.4**								
MVK	mevalonate kinase (mevalonic aciduria)					1.2		-1.2				1.4	**1.4**
MX2	myxovirus (influenza virus) resistance 2 (mouse)			1.2									
MXI1	MAX interactor 1	-2.7	**-2.7**	-3.3	**-3.3**	-1.6		6.4	**6.4**	-1.7	**-1.7**	-6.2	**-6.2**
MYB	v-myb myeloblastosis viral oncogene homolog (avian)	-1.3		2.4	**2.4**	-1.4		2.2	**2.2**	1.8	**1.8**	-2.0	**-2.0**
MYBL1	v-myb myeloblastosis viral oncogene homolog (avian)-like 1	1.3											
MYBL2	v-myb myeloblastosis viral oncogene homolog (avian)-like 2											-2.5	**-2.5**
MYBPC1	myosin binding protein C, slow type					1.4							
MYC	v-myc myelocytomatosis viral oncogene homolog (avian)	-3.8	**-3.8**	-4.6	**-4.6**	-3.6	**-3.6**	1.6	**1.6**	-2.8	**-2.8**	-4.8	**-4.8**
MYCBP	c-myc binding protein							1.3	**1.3**	-1.2	**-1.2**	-1.6	**-1.6**
MYCBP2	MYC binding protein 2	-1.2				-1.3		-1.5	**-1.5**	-1.4	**-1.4**	1.2	
MYD88	myeloid differentiation primary response gene (88)	-1.3								-1.6	**-1.6**		
MYEF2	myelin expression factor 2	1.2								1.3			
MYH10	myosin, heavy chain 10, non-muscle			-1.3				-3.7	**-3.7**	-1.4	**-1.3**	3.8	**3.8**
MYH9	myosin, heavy chain 9, non-muscle	1.4		1.3		1.6	**1.6**	-1.8	**-1.8**	1.3		2.2	**2.2**
MYL6	myosin, light chain 6, alkali, smooth muscle and non-muscle					1.2				1.5	**1.5**	1.4	**1.4**

MYL6B	myosin, light chain 6B, alkali, smooth muscle and non-muscle							-1.7	**-1.7**			1.5	
MYO1B	myosin IB	11.8	**11.8**	18.0	**18.0**			3.5	**3.5**	2.3	**2.3**	-6.5	**-6.5**
MYO1C	myosin IC												
MYO5A	myosin VA (heavy chain 12, myoxin)					-1.5		-8.2	**-8.2**			6.2	**6.2**
MYO9B	myosin IXB	1.4				1.4	**1.3**	-1.2		-1.3	**-1.3**	-1.3	**-1.3**
MYOM2	myomesin (M-protein) 2, 165kDa	-1.2											
MYOZ3	myozenin 3			1.4									
MYST1	MYST histone acetyltransferase 1			1.2	**1.2**			-1.9	**-1.9**			2.3	**2.3**
MYST2	MYST histone acetyltransferase 2					1.2				-1.3	**-1.3**	-1.3	**-1.3**
MYST3	MYST histone acetyltransferase (monocytic leukemia) 3	-1.5						1.2		1.9	**1.4**	1.4	**1.4**
MYST4	MYST histone acetyltransferase (monocytic leukemia) 4	-1.3		-1.2	**-1.2**			2.2	**2.2**	-1.9	**-1.9**	-2.6	**-2.6**
MYT1L	myelin transcription factor 1-like	1.4		1.8	**1.8**								
N4BP1	Nedd4 binding protein 1	-1.3				-1.2							
NAB1	NGFI-A binding protein 1 (EGR1 binding protein 1)			-1.7	**-1.7**							-3.2	**-3.2**
NACA	nascent-polypeptide-associated complex alpha polypeptide					-1.3		1.3	**1.3**			-1.9	**-1.9**
NADK	NAD kinase							-1.3		1.3	**1.3**	-1.9	**-1.9**
NAGA	N-acetylgalactosaminidase, alpha-			1.3				1.6	**1.6**	-1.2		-2.4	**-2.4**
NAGPA	N-acetylglucosamine-1-phosphodiester alpha-N-acetylglucosaminidase	-1.2											
NAP1L1	nucleosome assembly protein 1-like 1	-1.3	**-1.3**			-1.2		1.9	**1.9**	-1.3	**-1.2**	-2.9	**-2.9**
NAP1L4	nucleosome assembly protein 1-like 4							-1.5	**-1.5**			1.4	**-1.3**
NAPA	N-ethylmaleimide-sensitive factor attachment protein, alpha							-1.5	**-1.5**				
NAPG	N-ethylmaleimide-sensitive factor attachment protein, gamma					1.7		-1.5		1.5	**1.5**	1.3	
NARS	asparaginyl-tRNA synthetase	-1.6	**-1.6**	-1.7	**-1.7**	-1.4				-1.4	**-1.4**	-1.6	**-1.6**
NASP	nuclear autoantigenic sperm protein (histone-binding)									-2.4	**-2.4**	-5.5	**-5.5**
NAT1	N-acetyltransferase 1 (arylamine N-acetyltransferase)			1.5	**1.5**			1.2	**1.2**	1.3	**1.3**		
NBL1	neuroblastoma, suppression of tumorigenicity 1					2.2		2.3	**2.3**			-3.0	**-3.0**
NBR1	neighbor of BRCA1 gene 1			2.1	**2.1**	1.3		2.6	**2.6**	1.5	**1.5**	1.4	**-1.2**
NBS1	nibrin	1.9	**1.9**	3.0	**3.0**	1.7	**1.7**	-1.6	**-1.6**	-1.2	**-1.2**	1.8	**1.8**
NCBP1	nuclear cap binding protein subunit 1, 80kDa			1.2				-1.3	**-1.3**	-1.6	**-1.2**		
NCBP2	nuclear cap binding protein subunit 2, 20kDa	-1.6	**-1.6**	-1.5	**-1.5**	-1.5				-1.2		-1.3	
NCF4	neutrophil cytosolic factor 4, 40kDa							-1.9	**-1.9**	-1.5	**-1.5**	1.6	**1.6**
NCK1	NCK adaptor protein 1	1.5	**1.4**	2.3	**2.3**	2.3	**2.3**	1.7	**1.7**	1.5	**1.5**	1.5	
NCKAP1	NCK-associated protein 1							6.0	**6.0**			-5.9	**-5.9**
NCKAP1L	NCK-associated protein 1-like	1.4	**1.4**	1.4				1.3	**1.3**				
NCKIPSD	NCK interacting protein with SH3 domain					1.6				-1.3		-1.8	**-1.8**
NCL	nucleolin	-1.3	**-1.3**	-1.5	**-1.5**	-1.2		-1.6	**-1.6**	-1.9	**-1.9**	-1.2	**-1.2**
NCLN	nicalin homolog (zebrafish)	-1.5	**-1.5**	-1.6	**-1.6**	-1.8	**-1.8**	1.4				-3.0	**-3.0**
NCOA1	nuclear receptor coactivator 1	1.4								5.1	**5.1**	1.4	
NCOA2	nuclear receptor coactivator 2			1.4	**1.4**			-1.5	**-1.5**	1.9	**1.9**	5.5	**5.5**
NCOA3	nuclear receptor coactivator 3	1.9		1.3	**1.3**			-1.4	**-1.4**	2.1	**2.1**	3.8	**3.8**
NCOA4	nuclear receptor coactivator 4	1.5	**1.5**	1.5	**1.5**			1.9	**1.9**	1.3	**1.3**	-1.4	**-1.4**
NCOA6	nuclear receptor coactivator 6												
NCOR2	nuclear receptor co-repressor 2			-1.3	**-1.3**					1.5	**1.5**	1.3	**1.3**
NCSTN	nicastrin	1.3		1.3	**1.3**			1.9	**1.9**			-1.3	
NDN	necdin homolog (mouse)												
NDP52	calcium binding and coiled-coil domain 2							-1.4		-1.4		2.7	**2.7**
NDRG1	N-myc downstream regulated gene 1	1.6	**1.6**	1.7	**1.7**	1.4		9.2	**9.2**	3.1	**3.1**	2.3	**2.3**
NDST1	N-deacetylase/N-sulfotransferase (heparan glucosaminyl) 1			-1.4				2.5	**2.5**			-4.3	**-4.3**
NDUFA1	NADH dehydrogenase (ubiquinone) 1 alpha subcomplex, 1, 7.5kDa							-1.2				-1.2	
NDUFA2	NADH dehydrogenase (ubiquinone) 1 alpha subcomplex, 2, 8kDa											-1.3	**-1.3**
NDUFA5	NADH dehydrogenase (ubiquinone) 1 alpha subcomplex, 5, 13kDa					-1.2		-2.4	**-2.4**			1.9	**1.9**
NDUFA7	NADH dehydrogenase (ubiquinone) 1 alpha subcomplex, 7, 14.5kDa												
NDUFA9	NADH dehydrogenase (ubiquinone) 1 alpha subcomplex, 9, 39kDa			-1.2		-1.8		-2.1	**-2.1**	-1.5			
NDUFAB1	NADH dehydrogenase (ubiquinone) 1, alpha/beta subcomplex, 1, 8kDa			-1.4	**-1.4**	-1.5		-1.9	**-1.9**	-1.5	**-1.5**		
NDUFAF1	NADH dehydrogenase (ubiquinone) 1 alpha subcomplex, assembly factor 1	-1.8		-1.2		-1.5	**-1.2**	-3.0	**-3.0**	-1.8	**-1.8**	2.5	**2.5**
NDUFB1	NADH dehydrogenase (ubiquinone) 1 beta subcomplex, 1, 7kDa	1.3						1.4		-1.5		-1.3	**-1.3**
NDUFB3	NADH dehydrogenase (ubiquinone) 1 beta subcomplex, 3, 12kDa							1.2	**1.2**			-1.8	**-1.8**
NDUFB5	NADH dehydrogenase (ubiquinone) 1 beta subcomplex, 5, 16kDa			1.2	**1.2**			1.7	**1.7**			-1.8	**-1.8**

NDUFB7	NADH dehydrogenase (ubiquinone) 1 beta subcomplex, 7, 18kDa	-1.3						-1.3	**-1.3**	1.9	**1.9**	1.7	
NDUFB8	NADH dehydrogenase (ubiquinone) 1 beta subcomplex, 8, 19kDa							1.2	**1.2**	-1.2		-2.1	**-2.1**
NDUFC1	NADH dehydrogenase (ubiquinone) 1, subcomplex unknown, 1, 6kDa			-1.2				1.4	**1.4**			-2.0	**-2.0**
NDUFS1	NADH dehydrogenase (ubiquinone) Fe-S protein 1, 75kDa (NADH-coenzyme Q reductase)					-1.2		-1.6	**-1.6**	-1.5	**-1.5**	1.4	**1.4**
NDUFS2	NADH dehydrogenase (ubiquinone) Fe-S protein 2, 49kDa (NADH-coenzyme Q reductase)							1.9	**1.9**	-1.3		-1.7	**-1.7**
NDUFS3	NADH dehydrogenase (ubiquinone) Fe-S protein 3, 30kDa (NADH-coenzyme Q reductase)									-1.4	**-1.4**	-1.7	**-1.7**
NDUFS4	NADH dehydrogenase (ubiquinone) Fe-S protein 4, 18kDa (NADH-coenzyme Q reductase)					-1.3						1.6	**1.6**
NDUFS5	NADH dehydrogenase (ubiquinone) Fe-S protein 5, 15kDa (NADH-coenzyme Q reductase)					-1.2		-1.9	**-1.9**	-1.5	**-1.5**	-1.3	
NDUFS6	NADH dehydrogenase (ubiquinone) Fe-S protein 6, 13kDa (NADH-coenzyme Q reductase)	1.2						-1.5	**-1.5**			-1.8	**-1.8**
NDUFS7	NADH dehydrogenase (ubiquinone) Fe-S protein 7, 20kDa (NADH-coenzyme Q reductase)							-1.3		-1.6	**-1.6**	-1.7	**-1.7**
NDUFS8	NADH dehydrogenase (ubiquinone) Fe-S protein 8, 23kDa (NADH-coenzyme Q reductase)			-1.2						1.7	**-1.5**	-2.2	**-2.2**
NDUFV1	NADH dehydrogenase (ubiquinone) flavoprotein 1, 51kDa							-1.6	**-1.6**	-1.4	**-1.4**	-2.0	**-2.0**
NDUFV2	NADH dehydrogenase (ubiquinone) flavoprotein 2, 24kDa	-1.2		-1.3	**-1.3**	-1.4							
NEBL	nebulette							-1.3		-1.6		1.8	**1.8**
NECAP1	NECAP endocytosis associated 1							-1.3	**-1.3**	-1.3	**-1.3**	1.6	**1.6**
NEDD4	neural precursor cell expressed, developmentally down-regulated 4							2.8	**2.8**	1.2		-3.0	**-3.0**
NEDD8	neural precursor cell expressed, developmentally down-regulated 8									-1.3		-1.2	
NEK1	NIMA (never in mitosis gene a)-related kinase 1							-1.5	**-1.5**	-2.4	**-2.4**	-1.3	
NEK2	NIMA (never in mitosis gene a)-related kinase 2			1.5				-1.9	**-1.9**	1.5	**1.5**	2.1	**2.1**
NEK4	NIMA (never in mitosis gene a)-related kinase 4			1.5						1.3	**1.3**		
NEK9	NIMA (never in mitosis gene a)- related kinase 9												
NEURL	neuralized homolog (Drosophila)											-2.0	
NF1	neurofibromin 1 (neurofibromatosis, von Recklinghausen disease, Watson disease)			1.7		1.7				2.0	**2.0**	-1.4	**-1.4**
NF2	neurofibromin 2 (bilateral acoustic neuroma)	-1.3				-1.2		-1.3	**-1.3**	-1.4		1.4	
NFATC2IP	nuclear factor of activated T-cells, cytoplasmic, calcineurin-dependent 2 interacting protein	1.4		1.4	**1.4**	1.2		-2.4	**-2.4**	-1.3	**-1.3**	1.3	
NFATC3	nuclear factor of activated T-cells, cytoplasmic, calcineurin-dependent 3	-1.4	**-1.4**	-1.4	**-1.3**	-1.9	**-1.9**	-1.8	**-1.8**	-1.4	**-1.4**	1.2	
NFE2	nuclear factor (erythroid-derived 2), 45kDa					-3.3							
NFE2L1	nuclear factor (erythroid-derived 2)-like 1					-1.4	**-1.4**	1.6	**1.6**	1.2		-1.5	**-1.5**
NFE2L2	nuclear factor (erythroid-derived 2)-like 2							2.2	**2.2**	-1.4	**1.3**	-2.1	**-2.1**
NFIC	nuclear factor I/C (CCAAT-binding transcription factor)					1.2		5.9	**5.9**	-1.9	**-1.9**	-8.4	**-8.4**
NFIL3	nuclear factor, interleukin 3 regulated	6.0	**6.0**	7.4	**7.4**	3.6	**3.6**	-2.5	**-2.5**	5.0	**5.0**	19.1	**19.1**
NFKB1	nuclear factor of kappa light polypeptide gene enhancer in B-cells 1 (p105)	1.4	**1.4**	1.6	**1.5**	-1.3				-2.2	**-2.2**		
NFKB2	nuclear factor of kappa light polypeptide gene enhancer in B-cells 2 (p49/p100)							-1.5	**-1.5**			1.7	**1.7**
NFKBIA	nuclear factor of kappa light polypeptide gene enhancer in B-cells inhibitor, alpha	3.0	**3.0**	3.4	**3.4**	2.8	**2.8**	1.9	**1.9**	2.5	**2.5**	5.5	**5.5**
NFKBIB	nuclear factor of kappa light polypeptide gene enhancer in B-cells inhibitor, beta												
NFKBIE	nuclear factor of kappa light polypeptide gene enhancer in B-cells inhibitor, epsilon					-1.5							
NFRKB	nuclear factor related to kappaB binding protein					1.3		2.2	**2.2**			-6.3	**-6.3**
NFX1	nuclear transcription factor, X-box binding 1									1.5		-1.3	**-1.3**
NFYA	nuclear transcription factor Y, alpha					-1.4		1.6	**1.6**			-1.9	**-1.9**
NFYB	nuclear transcription factor Y, beta			1.2				-1.6	**-1.6**			1.3	**1.3**
NFYC	nuclear transcription factor Y, gamma			-1.3	**-1.3**			-1.4	**-1.4**	-1.3	**-1.3**	-1.2	
NGFR	nerve growth factor receptor (TNFR superfamily, member 16)					1.3				6.7	**6.7**		
NHP2L1	NHP2 non-histone chromosome protein 2-like 1 (S. cerevisiae)									-1.6	**-1.6**	-1.9	**-1.9**
NIPSNAP1	nipsnap homolog 1 (C. elegans)			-1.3		-1.8	**-1.8**			-2.9	**-2.9**	-1.8	**-1.8**
NISCH	nischarin			-1.2								3.1	**3.1**
NIT1	nitrilase 1	1.4										1.3	**1.3**
NKRF	NF-kappaB repressing factor	-1.4		-1.5	**-1.5**							-1.3	**-1.3**
NKTR	natural killer-tumor recognition sequence			1.3		1.3		2.0	**2.0**			-1.7	
NKX2-5	NK2 transcription factor related, locus 5 (Drosophila)	-2.0	**-2.0**	-2.8	**-2.8**	-2.4	**-2.4**						
NMB	neuromedin B							1.4	**1.4**			-2.0	**-2.0**
NME1	non-metastatic cells 1, protein (NM23A) expressed in	-1.5	**-1.5**	-2.2	**-2.2**	-1.7	**-1.7**			-4.4	**-4.4**	-7.6	**-7.6**
NME2	non-metastatic cells 2, protein (NM23B) expressed in			-1.3		-1.3				-1.3		-1.6	**-1.6**
NME4	non-metastatic cells 4, protein expressed in	-1.3		-2.3	**-2.3**	-1.6				-2.1	**-2.1**	-2.5	**-2.5**
NME6	non-metastatic cells 6, protein expressed in (nucleoside-diphosphate kinase)					1.2		1.6	**1.6**			-1.6	**-1.6**
NMI	N-myc (and STAT) interactor					-1.3		1.2	**1.2**			-1.8	**-1.8**
NMT1	N-myristoyltransferase 1			-1.2						1.4	**-1.3**	-1.3	**-1.3**
NMT2	N-myristoyltransferase 2	1.2						1.3		-2.4	**-2.4**		
NNT	nicotinamide nucleotide transhydrogenase	1.3		1.3				1.3	**1.3**			-1.3	**-1.3**

NOC2L	nucleolar complex associated 2 homolog (S. cerevisiae)	-1.5		-1.7	**-1.7**	-1.3	**-1.3**			-1.8	**-1.8**	-1.4	**-1.4**
NOL1	nucleolar protein 1, 120kDa	-1.4		-1.7	**-1.7**	-1.6		-1.4		-1.5	**-1.5**		
NOL5A	nucleolar protein 5A (56kDa with KKE/D repeat)	-1.9	**-1.9**	-2.7	**-2.7**	-1.8		1.3		-2.0	**-2.0**	-3.0	**-3.0**
NOLA2	nucleolar protein family A, member 2 (H/ACA small nucleolar RNPs)	-1.3		-1.9	**-1.9**	-1.6		-1.5	**-1.5**	-1.8	**-1.8**	-2.0	**-2.0**
NOLC1	nucleolar and coiled-body phosphoprotein 1	-2.1	**-2.1**	-2.5	**-2.5**	-2.0		1.5	**1.5**	-2.0	**-2.0**	-3.9	**-3.9**
NONO	non-POU domain containing, octamer-binding									-1.2			
NOS3	nitric oxide synthase 3 (endothelial cell)												
NOVA1	neuro-oncological ventral antigen 1			1.3		1.7	**1.7**						
NP	nucleoside phosphorylase	-1.8	**-1.8**	-1.6	**-1.6**	-2.0	**-2.0**			-1.3	**-1.3**	-2.7	**-2.7**
NPAT	nuclear protein, ataxia-telangiectasia locus			1.3		-1.4		-1.2		-1.5	**-1.2**	-1.7	
NPC1	Niemann-Pick disease, type C1									1.5	**1.5**	2.7	**2.7**
NPC2	Niemann-Pick disease, type C2	1.3	**1.3**	1.4	**1.4**	1.4		1.8	**1.8**				
NPM1	nucleophosmin (nucleolar phosphoprotein B23, numatrin)			-1.5	**-1.5**			1.2	**1.2**	-1.9	**-1.9**	-2.6	**-2.6**
NPM3	nucleophosmin/nucleoplasmin, 3	-1.4	**-1.4**	-1.8	**-1.8**	-1.3		2.5	**2.5**	-1.7		-4.8	**-4.8**
NPPA	natriuretic peptide precursor A												
NQO1	NAD(P)H dehydrogenase, quinone 1									-1.3		1.3	
NQO2	NAD(P)H dehydrogenase, quinone 2							2.2	**2.2**	-1.5	**-1.5**	-3.4	**-3.4**
NR1H2	nuclear receptor subfamily 1, group H, member 2			-1.4						-1.3		1.4	
NR2C1	nuclear receptor subfamily 2, group C, member 1							1.6	**1.6**	1.3	**1.3**		
NR2F6	nuclear receptor subfamily 2, group F, member 6												
NR3C1	nuclear receptor subfamily 3, group C, member 1 (glucocorticoid receptor)	4.2	**4.2**	6.6	**6.6**	2.1	**2.1**	-1.8	**-1.8**	3.1	**3.1**	6.5	**6.5**
NRAS	neuroblastoma RAS viral (v-ras) oncogene homolog			2.5	**2.5**			-1.4	**-1.4**			1.8	**1.8**
NRD1	nardilysin (N-arginine dibasic convertase)			-1.2				1.7	**1.7**	1.3		-1.6	**-1.6**
NRIP1	nuclear receptor interacting protein 1					-1.7		-1.4	**-1.4**	1.5	**1.5**		
NRTN	neurturin	1.3				1.8	**1.8**						
NSDHL	NAD(P) dependent steroid dehydrogenase-like			-1.6	**-1.6**	1.3						1.5	**1.5**
NSEP1	Y box binding protein 1					-1.4				-1.7	**-1.7**	-1.8	**-1.8**
NSF	N-ethylmaleimide-sensitive factor					1.3		1.4	**1.4**	-1.3	**-1.3**	-1.8	**-1.8**
NSFL1C	NSFL1 (p97) cofactor (p47)			-1.3	**-1.3**			1.5	**1.5**	1.5	**1.5**	-2.2	**-2.2**
NSMAF	neutral sphingomyelinase (N-SMase) activation associated factor			-1.2		-1.4		-1.3	**-1.3**	-2.7	**-2.7**	-1.3	
NSUN5C	NOL1/NOP2/Sun domain family, member 5C							-1.7	**-1.7**			1.2	**1.2**
NT5C2	5'-nucleotidase, cytosolic II			1.2				2.3	**2.3**	1.5	**1.5**	-1.6	**-1.5**
NTE	patatin-like phospholipase domain containing 6									-1.4		1.3	**1.3**
NTHL1	nth endonuclease III-like 1 (E. coli)	-1.7	**-1.7**	-1.6	**-1.6**			-2.2	**-2.2**	-1.7	**-1.7**		
NTSR2	neurotensin receptor 2	1.2				1.3							
NUBP1	nucleotide binding protein 1 (MinD homolog, E. coli)			-1.2				-1.9	**-1.9**				
NUCB2	nucleobindin 2							1.3	**1.3**	-1.3	**-1.3**	-3.1	**-3.1**
NUDC	nuclear distribution gene C homolog (A. nidulans)			-1.4		-1.2		-1.2	**-1.2**	-1.5	**-1.5**	-2.1	**-2.1**
NUDT1	nudix (nucleoside diphosphate linked moiety X)-type motif 1	1.2						-2.3	**-2.3**	1.3		1.8	**1.8**
NUDT3	nudix (nucleoside diphosphate linked moiety X)-type motif 3					-1.4		1.9	**1.9**	-1.2	**-1.2**	-3.0	**-3.0**
NUFIP1	nuclear fragile X mental retardation protein interacting protein 1	-1.4		-1.4				-1.8	**-1.8**	-1.8	**-1.8**	-2.2	**-2.2**
NUMA1	nuclear mitotic apparatus protein 1	1.3				1.2		-2.1	**-2.1**			1.9	**1.9**
NUMB	numb homolog (Drosophila)	1.4		1.3				1.3		1.4		1.6	**1.6**
NUP133	nucleoporin 133kDa	1.2		1.4		-1.5		-1.3				-1.3	**-1.3**
NUP153	nucleoporin 153kDa	-1.5	**-1.5**	-1.3		-1.7		-2.0	**-2.0**	-1.4	**-1.4**	1.4	**1.4**
NUP155	nucleoporin 155kDa					-1.9		-1.5	**-1.5**			-2.3	**-2.3**
NUP160	nucleoporin 160kDa	1.2				-1.4						-2.0	**-2.0**
NUP205	nucleoporin 205kDa			-1.2		-1.3		-1.2	**-1.2**	-1.5	**-1.5**	-1.3	**-1.3**
NUP210	nucleoporin 210kDa							-3.4	**-3.4**	-1.7	**-1.7**	-1.5	**-1.5**
NUP50	nucleoporin 50kDa					1.3		-1.3	**-1.3**				
NUP62	nucleoporin 62kDa	-1.4	**-1.4**	-1.4		-1.3		-1.4	**-1.4**	-1.4		-2.6	**-2.6**
NUP88	nucleoporin 88kDa			-1.7	**-1.7**	-1.6		-2.2	**-2.2**	-1.2		1.2	
NUP93	nucleoporin 93kDa			-1.5	**-1.5**	-1.4				-1.2		-2.3	**-2.3**
NUP98	nucleoporin 98kDa	-1.2		-1.2		-1.3	**-1.3**	-1.7	**-1.7**	-2.0	**-2.0**	-1.4	**1.4**
NUPL1	nucleoporin like 1	-1.3						-2.9	**-2.9**			1.3	
NUPL2	nucleoporin like 2							3.8	**3.8**	1.2		-3.5	**-3.5**
NUTF2	nuclear transport factor 2					-1.4		-1.5	**-1.5**	-1.6	**-1.6**		
NVL	nuclear VCP-like												

NXF1	nuclear RNA export factor 1					1.3		1.3	**1.3**				
NXT2	nuclear transport factor 2-like export factor 2	1.5	**1.5**	1.3									
OAS2	2'-5'-oligoadenylate synthetase 2, 69/71kDa					1.3		-3.2	**-3.2**	-2.8	**-2.8**	2.9	**2.9**
OAZ1	ornithine decarboxylase antizyme 1							-1.3	**-1.3**	1.4	**1.4**	1.3	**1.3**
OAZ2	ornithine decarboxylase antizyme 2	1.4	**1.4**									1.4	**1.4**
OAZIN	antizyme inhibitor 1			1.4				-2.2	**-2.2**			1.9	**1.9**
OCRL	oculocerebrorenal syndrome of Lowe											-2.2	**-2.2**
ODC1	ornithine decarboxylase 1	-2.1	**-2.1**	-2.7	**-2.7**	-2.2	**-2.2**	-1.2	**-1.2**	-1.5	**-1.5**	-6.6	**-6.6**
OGDH	oxoglutarate (alpha-ketoglutarate) dehydrogenase (lipoamide)							1.8	**1.8**	-1.7	**-1.7**	-2.7	**-2.7**
OGFR	opioid growth factor receptor			-1.7									
OGG1	8-oxoguanine DNA glycosylase	1.5				1.4	**1.4**	-3.9	**-3.9**			6.2	**6.2**
OGT	O-linked N-acetylglucosamine (GlcNAc) transferase (UDP-N-acetylglucosamine:polypeptide-N-acetylglucosaminyl transferase)	1.3		1.7	**1.7**	2.0	**2.0**	2.0	**2.0**	1.3	**1.3**	2.3	**2.3**
OIP5	Opa interacting protein 5							-2.6	**-2.6**			1.7	**1.7**
OPA1	optic atrophy 1 (autosomal dominant)					-1.3		1.5	**1.5**	-1.3		-1.6	**-1.6**
OPRS1	opioid receptor, sigma 1	-1.3		-2.0	**-2.0**					-1.5		-2.6	**-2.6**
ORC1L	origin recognition complex, subunit 1-like (yeast)							1.7	**1.7**			-22.9	**-22.9**
ORC2L	origin recognition complex, subunit 2-like (yeast)							2.6	**2.6**			-3.0	**-3.0**
ORC3L	origin recognition complex, subunit 3-like (yeast)							1.3	**1.3**	1.3		-1.8	**-1.8**
ORC5L	origin recognition complex, subunit 5-like (yeast)	-1.3	**-1.3**			-1.5	**-1.5**	1.3	**1.3**			-1.9	**-1.9**
ORM1	orosomucoid 1	-1.3											
OS9	amplified in osteosarcoma	1.5	**1.5**	1.3	**1.3**	1.4	**1.4**			1.3		1.7	**1.7**
OSBP	oxysterol binding protein			1.3				1.4	**1.4**	1.4		1.7	**1.7**
OSBPL8	oxysterol binding protein-like 8							-1.3	**-1.3**			1.4	**1.4**
OSGEP	O-sialoglycoprotein endopeptidase							-1.3		-1.3		-2.0	**-2.0**
OSTF1	osteoclast stimulating factor 1			1.4		-1.4		-1.3	**-1.3**	1.2	**1.2**	1.8	**1.6**
OXA1L	oxidase (cytochrome c) assembly 1-like			1.2				1.4		-1.5	**-1.5**		
OXCT1	3-oxoacid CoA transferase 1									1.4		-1.5	**-1.5**
OXSR1	oxidative-stress responsive 1	-1.2				-1.3		1.3	**1.3**			-1.7	**-1.7**
P29	SYF2 homolog, RNA splicing factor (S. cerevisiae)					1.2				1.4	**1.4**	1.4	**1.4**
P2RX4	purinergic receptor P2X, ligand-gated ion channel, 4							2.0	**2.0**			-1.6	
P2RX5	purinergic receptor P2X, ligand-gated ion channel, 5	1.3		1.4	**1.4**	2.7	**2.7**	-2.0	**-2.0**	5.5	**5.5**	6.6	**6.6**
P2RX7	purinergic receptor P2X, ligand-gated ion channel, 7	1.3											
P4HA1	procollagen-proline, 2-oxoglutarate 4-dioxygenase (proline 4-hydroxylase), alpha polypeptide I	-1.3	**-1.3**					3.0	**3.0**	-1.2		-1.5	**-1.5**
P4HB	procollagen-proline, 2-oxoglutarate 4-dioxygenase (proline 4-hydroxylase), beta polypeptide					1.5	**1.5**	3.7	**3.7**			-3.0	**-3.0**
P53CSV	TP53 regulated inhibitor of apoptosis 1	-1.3		-1.4		-1.3				-1.4	**-1.4**	-2.1	**-2.1**
P8	nuclear protein 1	-1.5											
PA2G4	proliferation-associated 2G4, 38kDa	-1.7	**-1.7**	-1.9	**-1.9**	-1.7		-1.3		-2.5	**-2.5**	-3.5	**-3.5**
PABPC1	poly(A) binding protein, cytoplasmic 1			1.2				-2.2	**-2.2**	-1.4	**-1.4**	1.7	**1.7**
PABPC4	poly(A) binding protein, cytoplasmic 4 (inducible form)									-1.8	**-1.8**	-1.4	**-1.4**
PABPN1	poly(A) binding protein, nuclear 1			-1.4				1.6	**1.6**	-1.3	**-1.3**	-1.5	**-1.5**
PACS2	phosphofurin acidic cluster sorting protein 2	1.3								1.6	**1.4**	1.4	
PACSIN2	protein kinase C and casein kinase substrate in neurons 2					2.3	**2.3**			1.4	**1.4**	1.3	**1.3**
PAFAH1B1	platelet-activating factor acetylhydrolase, isoform Ib, alpha subunit 45kDa			1.3				-2.4	**-2.4**	1.3	**1.2**	2.9	**2.9**
PAFAH1B2	platelet-activating factor acetylhydrolase, isoform Ib, beta subunit 30kDa	-1.5								1.3			
PAFAH1B3	platelet-activating factor acetylhydrolase, isoform Ib, gamma subunit 29kDa	-1.5	**-1.5**	-1.5	**-1.5**	-1.2						-1.8	**-1.8**
PAICS	phosphoribosylaminoimidazole carboxylase, phosphoribosylaminoimidazole succinocarboxamide synthetase	-1.7	**-1.7**	-2.6	**-2.6**	-1.7		1.7	**1.7**	-3.3	**-3.3**	-12.5	**-12.5**
PAIP1	poly(A) binding protein interacting protein 1							1.2		1.4	**-1.4**	-1.8	**-1.8**
PAI-RBP1	SERPINE1 mRNA binding protein 1	-1.6	**-1.6**	-1.5		-1.6		-1.5	**-1.5**	-1.9	**-1.9**	-2.2	**-2.2**
PAK1	p21/Cdc42/Rac1-activated kinase 1 (STE20 homolog, yeast)	1.5	**1.5**			1.3		2.5	**2.5**	-1.3	**-1.3**	-3.4	**-3.4**
PAK2	p21 (CDKN1A)-activated kinase 2	-1.5	**-1.2**	1.6	**-1.2**	-1.2		-1.6	**-1.6**	1.5	**1.5**	2.0	**2.0**
PAK4	p21(CDKN1A)-activated kinase 4			-1.3	**-1.3**					1.4	**1.4**		
PAM	peptidylglycine alpha-amidating monooxygenase	1.5	**1.5**	-1.2		1.2		1.3	**1.3**	-1.4	**-1.4**	-1.6	**-1.6**
PANK3	pantothenate kinase 3			3.0	**3.0**					1.4	**1.3**	2.6	**2.6**
PAPOLA	poly(A) polymerase alpha	-1.3		1.8		-1.3		1.4	**1.4**	1.4		-1.4	**-1.4**
PAPSS1	3'-phosphoadenosine 5'-phosphosulfate synthase 1			-1.3				1.2		-1.4	**-1.4**		
PARD3	par-3 partitioning defective 3 homolog (C. elegans)	1.6		1.7	**1.7**	1.4		2.5	**2.5**	1.5		3.0	**3.0**
PARD6A	par-6 partitioning defective 6 homolog alpha (C. elegans)			1.3									
PARG	poly (ADP-ribose) glycohydrolase					-1.4				-1.3		-1.7	**-1.7**

PARG1	Rho GTPase activating protein 29					2.2	2.2						
PARK7	Parkinson disease (autosomal recessive, early onset) 7											-1.3	-1.3
PARN	poly(A)-specific ribonuclease (deadenylation nuclease)							-2.1	-2.1			2.2	2.2
PARP1	poly (ADP-ribose) polymerase family, member 1					-1.2		-2.5	-2.5	1.7	1.7	2.2	2.2
PARP2	poly (ADP-ribose) polymerase family, member 2	1.3				-1.7	-1.7			-1.6	-1.6	-1.7	-1.7
PASK	PAS domain containing serine/threonine kinase									-1.7			
PAWR	PRKC, apoptosis, WT1, regulator	-1.2	-1.2	1.3		-1.3		42.6	42.6	-2.4	-2.4	-46.4	-46.4
PAX6	paired box gene 6 (aniridia, keratitis)	2.3	2.3	1.7		1.5	1.5						
PAX7	paired box gene 7					1.4							
PAX8	paired box gene 8					1.3		3.7	3.7	-1.2		-3.2	-3.2
PAX9	paired box gene 9					1.2		1.4		1.4			
PAXIP1L	PAX interacting (with transcription-activation domain) protein 1	1.4	1.4	1.5	1.5	1.3	1.3	-3.5	-3.5	1.2	1.2	12.7	12.7
PBEF1	pre-B-cell colony enhancing factor 1							1.7	1.7			-1.6	-1.6
PBP	phosphatidylethanolamine binding protein 1			-1.3						-2.0	-2.0	-2.4	-2.4
PBX1	pre-B-cell leukemia transcription factor 1	1.4		1.8	1.8			-1.8					
PBX3	pre-B-cell leukemia transcription factor 3							-172.9	-172.9	-1.5	-1.5	135.1	135.1
PC4	SUB1 homolog (S. cerevisiae)					-1.4		2.4	2.4	-1.4	-1.4	-4.6	-4.6
PCBD1	pterin-4 alpha-carbinolamine dehydratase/dimerization cofactor of hepatocyte nuclear factor 1 alpha (TCF1)			1.4								-1.3	
PCBP1	poly(rC) binding protein 1												
PCBP2	poly(rC) binding protein 2							2.0	2.0	1.2		-1.9	-1.9
PCCA	propionyl Coenzyme A carboxylase, alpha polypeptide	1.4		1.7	1.7	-1.3	-1.3			-1.2	-1.2	1.7	1.7
PCCB	propionyl Coenzyme A carboxylase, beta polypeptide	-1.2		-1.3	-1.3	-1.8		1.3	1.3			-1.7	-1.7
PCF11	PCF11, cleavage and polyadenylation factor subunit, homolog (S. cerevisiae)					1.3		-2.1	-2.1	1.2		2.6	2.6
PCGF1	polycomb group ring finger 1	-1.4		-2.1		-1.9	-1.9						
PCGF2	polycomb group ring finger 2							1.3		-1.5		1.3	
PCGF4	B lymphoma Mo-MLV insertion region (mouse)					-1.2		-1.5	-1.5	1.3	1.3	1.2	1.2
PCID1	PCI domain containing 1 (herpesvirus entry mediator)					-1.5		1.6	1.2			-1.6	-1.6
PCK2	phosphoenolpyruvate carboxykinase 2 (mitochondrial)			-1.3	-1.3			-1.2		-1.7	-1.7	-1.4	-1.4
PCM1	pericentriolar material 1	-1.5				-1.2		2.3	2.3	1.8	1.8	-1.9	-1.9
PCMT1	protein-L-isoaspartate (D-aspartate) O-methyltransferase	-1.2				-1.3		-1.3	-1.3	1.4	1.4	1.4	1.4
PCNA	proliferating cell nuclear antigen							-1.7	-1.7	-1.6	-1.6	-3.7	-3.7
PCNT2	pericentrin (kendrin)							1.4	1.4			-1.3	
PCOLCE	procollagen C-endopeptidase enhancer					1.4							
PCOLN3	procollagen (type III) N-endopeptidase									-1.7	-1.7	1.2	
PCTK1	PCTAIRE protein kinase 1			-1.3				-1.4	-1.4			1.2	
PCTK2	PCTAIRE protein kinase 2			1.4		-1.3		-1.2	-1.2			1.8	1.8
PCYT1A	phosphate cytidylyltransferase 1, choline, alpha			1.6	1.6			1.2					
PCYT2	phosphate cytidylyltransferase 2, ethanolamine			-1.6	-1.6	-1.6						1.2	
PDAP1	PDGFA associated protein 1			-1.2				-3.1	-3.1			3.2	3.2
PDCD10	programmed cell death 10			-1.3		-1.3						1.3	1.3
PDCD11	programmed cell death 11	-1.6						1.3		-1.9	-1.9	-10.1	-10.1
PDCD2	programmed cell death 2	-1.3				1.3		2.0	2.0	1.3	1.3	-2.0	-2.0
PDCD6	programmed cell death 6					-1.2		-11.8	-11.8	2.0	2.0	20.7	20.7
PDCD8	apoptosis-inducing factor, mitochondrion-associated, 1									-1.6	-1.6	-1.5	
PDCL	phosducin-like			1.5	1.5			-1.7	-1.7			1.4	
PDE3B	phosphodiesterase 3B, cGMP-inhibited							6.1	6.1	-1.4	-1.4	-25.2	-25.2
PDE6D	phosphodiesterase 6D, cGMP-specific, rod, delta							-2.1	-2.1	1.2	1.2	2.3	2.3
PDE8A	phosphodiesterase 8A							-1.7	-1.5	1.7	1.6	1.6	1.6
PDHB	pyruvate dehydrogenase (lipoamide) beta					-1.4				-1.2		-1.4	-1.4
PDHX	pyruvate dehydrogenase complex, component X	-1.3				-1.4		-1.5	-1.5			1.2	1.2
PDIA3	protein disulfide isomerase family A, member 3							-1.3	-1.3			-1.4	-1.2
PDIA4	protein disulfide isomerase family A, member 4							-1.2	-1.2	-1.3		-1.7	-1.7
PDIA6	protein disulfide isomerase family A, member 6							-1.5	-1.5	-1.3	-1.3	-1.7	-1.7
PDK1	pyruvate dehydrogenase kinase, isozyme 1	-1.8				-1.3		2.8	2.8	-1.3		-5.8	-5.8
PDK2	pyruvate dehydrogenase kinase, isozyme 2					1.2	1.2	3.1	3.1			-1.4	
PDK3	pyruvate dehydrogenase kinase, isozyme 3	1.4		-1.4		1.4		-1.3	-1.3	-1.6	-1.6		
PDLIM5	PDZ and LIM domain 5			1.5		1.3		1.6	1.6	-1.6	-1.6	-1.2	
PDLIM7	PDZ and LIM domain 7 (enigma)			-1.3						-2.1	-2.1		

PDPK1	3-phosphoinositide dependent protein kinase-1							-1.8	**-1.8**	1.4		2.3	**2.3**
PDXK	pyridoxal (pyridoxine, vitamin B6) kinase					-1.4		2.8	**2.8**	1.2		-4.7	**-4.7**
PEA15	phosphoprotein enriched in astrocytes 15					-1.4		-1.3	**-1.3**	-1.4			
PECAM1	platelet/endothelial cell adhesion molecule (CD31 antigen)					-1.5	**-1.5**	-1.2		-1.7	**-1.7**	1.2	
PELP1	proline, glutamic acid and leucine rich protein 1			-1.3						1.4			
PEMT	phosphatidylethanolamine N-methyltransferase			-1.8	**-1.8**			-3.7	**-3.7**				
PEPD	peptidase D									-1.8	**-1.8**	-1.3	**-1.3**
PER1	period homolog 1 (Drosophila)	1.3		1.3		1.9				1.3		19.4	**19.4**
PER2	period homolog 2 (Drosophila)	-1.6		-1.3		-1.6		-2.0	**-2.0**	-1.7	**-1.7**		
PES1	pescadillo homolog 1, containing BRCT domain (zebrafish)	-1.6	**-1.6**	-1.8	**-1.8**	-1.6	**-1.6**					-1.6	**-1.6**
PEX1	peroxisome biogenesis factor 1	1.5		1.4						1.5	**1.5**	3.2	**3.2**
PEX10	peroxisome biogenesis factor 10	-1.3		-1.3		1.6		-1.3		-1.5			
PEX11B	peroxisomal biogenesis factor 11B	1.2		1.3	**1.3**			-1.4	**-1.4**	1.4	**1.4**	1.9	**1.9**
PEX14	peroxisomal biogenesis factor 14					-1.7	**-1.7**	1.3	**1.3**			-1.3	**-1.3**
PEX19	peroxisomal biogenesis factor 19	-1.3				-1.3							
PEX3	peroxisomal biogenesis factor 3					-1.2		1.4	**1.3**	-2.4	**-2.4**	-2.3	**-2.3**
PEX5	peroxisomal biogenesis factor 5	-1.3	**-1.3**	-1.3		-1.6	**-1.6**					-1.3	**-1.3**
PEX6	peroxisomal biogenesis factor 6					-1.2		1.5				-1.4	
PFAS	phosphoribosylformylglycinamidine synthase (FGAR amidotransferase)	-1.4		-2.1	**-2.1**	-1.2		-2.4	**-2.4**	-2.7	**-2.7**	-2.3	**-2.3**
PFDN1	prefoldin subunit 1							-1.4				1.7	
PFDN4	prefoldin subunit 4									1.3		1.6	
PFDN5	prefoldin subunit 5							1.4	**1.4**	1.4	**1.4**	-1.2	
PFKFB2	6-phosphofructo-2-kinase/fructose-2,6-biphosphatase 2					1.3						2.4	**2.4**
PFKFB3	6-phosphofructo-2-kinase/fructose-2,6-biphosphatase 3	-1.3											
PFKM	phosphofructokinase, muscle					-1.5		-26.2	**-26.2**	-4.3	**-4.3**	7.9	**7.9**
PFKP	phosphofructokinase, platelet							1.5	**1.5**	1.7	**1.7**	-1.6	**-1.6**
PFN1	profilin 1	-1.3		-1.2				-2.1	**-2.1**			1.8	**1.8**
PFN2	profilin 2					-1.4							
PGAM1	phosphoglycerate mutase 1 (brain)	-1.2	**-1.2**	-1.3	**-1.3**			1.5	**1.5**	-1.2	**-1.2**	-2.4	**-2.4**
PGD	phosphogluconate dehydrogenase			-1.2	**-1.2**			1.8	**1.8**	-1.3	**-1.3**	-2.1	**-2.1**
PGGT1B	protein geranylgeranyltransferase type I, beta subunit			1.7				-3.2	**-3.2**	1.4		3.9	**3.9**
PGK1	phosphoglycerate kinase 1	-1.3		-1.6	**-1.6**	-1.5		1.3	**1.3**	-1.9	**-1.9**	-2.6	**-2.6**
PGM1	phosphoglucomutase 1							1.7	**1.7**	-1.8	**-1.8**	-3.2	**-3.2**
PGM3	phosphoglucomutase 3	1.4		1.2		1.4		-2.6	**-2.6**			1.5	
PGRMC1	progesterone receptor membrane component 1							1.3		-1.5	**-1.5**	-1.8	**-1.8**
PGRMC2	progesterone receptor membrane component 2	-1.2	**-1.2**	-1.3	**-1.3**			1.7	**1.7**			-2.6	**-2.6**
PHB	prohibitin	-1.4		-1.5								-1.9	**-1.9**
PHB2	prohibitin 2					-1.3		-1.7	**-1.7**			1.5	**1.5**
PHC2	polyhomeotic homolog 2 (Drosophila)							-1.6	**-1.6**			1.7	**1.7**
PHF1	PHD finger protein 1					-1.2		1.8	**1.8**			-1.6	**-1.6**
PHF21A	PHD finger protein 21A					1.4		2.0	**2.0**	-1.3		-1.5	**-1.5**
PHGDH	phosphoglycerate dehydrogenase	-1.3						-1.7	**-1.7**	-2.4	**-2.4**	-2.3	**-2.3**
PHKA1	phosphorylase kinase, alpha 1 (muscle)							3.0	**3.0**			-4.3	**-4.3**
PHKA2	phosphorylase kinase, alpha 2 (liver)	1.3						1.7	**1.7**	-1.3	**-1.3**		
PHKB	phosphorylase kinase, beta	1.4				1.3		-1.7	**-1.7**	1.4	**1.4**	2.0	**2.0**
PHYH	phytanoyl-CoA 2-hydroxylase									1.7	**1.7**		
PIAS1	protein inhibitor of activated STAT, 1							1.7	**1.7**	1.7	**1.4**	2.2	**2.2**
PIAS2	protein inhibitor of activated STAT, 2	1.2		1.4		1.5		-1.4		-1.7	**-1.7**	-1.9	**-1.9**
PICALM	phosphatidylinositol binding clathrin assembly protein	1.7	**1.7**	1.7	**1.7**	1.5	**1.5**	2.1	**2.1**	2.0	**2.0**	2.1	**2.1**
PIG8	centrosomal protein 57kDa					-1.3		1.6	**1.6**	-1.3		-2.3	**-2.3**
PIGA	phosphatidylinositol glycan anchor biosynthesis, class A (paroxysmal nocturnal hemoglobinuria)									1.5	**1.5**		
PIGB	phosphatidylinositol glycan anchor biosynthesis, class B	1.4						1.2				-1.6	**-1.6**
PIGC	phosphatidylinositol glycan anchor biosynthesis, class C			1.3	**1.3**			1.9	**1.9**	2.5	**2.5**	-1.3	**-1.3**
PIGF	phosphatidylinositol glycan anchor biosynthesis, class F					-1.2				-1.3			
PIGH	phosphatidylinositol glycan anchor biosynthesis, class H											1.4	
PIGR	polymeric immunoglobulin receptor					1.4							
PIK3C2A	phosphoinositide-3-kinase, class 2, alpha polypeptide					-1.2		2.5	**2.5**	1.7	**1.7**	1.4	**1.4**
PIK3C2B	phosphoinositide-3-kinase, class 2, beta polypeptide											-5.3	**-5.3**

PIK3C3	phosphoinositide-3-kinase, class 3	1.4		1.8	**1.8**	-1.2							
PIK3CA	phosphoinositide-3-kinase, catalytic, alpha polypeptide							-2.5	**-2.5**	1.3	**1.3**	3.6	**3.6**
PIK3CB	phosphoinositide-3-kinase, catalytic, beta polypeptide			1.2		1.4		3.3	**3.3**	1.2	**1.2**	-2.7	**-2.7**
PIK3CG	phosphoinositide-3-kinase, catalytic, gamma polypeptide	1.6		1.4		-1.2		-1.6		2.0	**2.0**		
PIK3R1	phosphoinositide-3-kinase, regulatory subunit 1 (p85 alpha)	2.5	**2.5**	2.4	**2.4**	1.7		2.1	**2.1**	1.4		-1.3	
PIK3R3	phosphoinositide-3-kinase, regulatory subunit 3 (p55, gamma)	-1.3		1.8						-1.3			
PIK3R4	phosphoinositide-3-kinase, regulatory subunit 4, p150							1.3	**1.3**	-1.2		-1.3	**-1.3**
PIK4CB	phosphatidylinositol 4-kinase, catalytic, beta polypeptide			1.2	**1.2**					-1.4	**-1.4**	-1.2	**-1.2**
PIM1	pim-1 oncogene									1.3			
PIM2	pim-2 oncogene	2.1	**2.1**	2.8	**2.8**								
PIN1	protein (peptidylprolyl cis/trans isomerase) NIMA-interacting 1					-1.3		-1.6		1.6	**1.6**	-1.7	**-1.7**
PINK1	PTEN induced putative kinase 1	1.6	**1.6**					1.3	**1.3**			2.2	**2.2**
PIP3-E	phosphoinositide-binding protein PIP3-E			1.7				9.5	**9.5**			-7.5	**-7.5**
PIP5K1A	phosphatidylinositol-4-phosphate 5-kinase, type I, alpha					1.2				-1.5		1.3	**1.3**
PIP5K1C	phosphatidylinositol-4-phosphate 5-kinase, type I, gamma	1.6						2.7	**2.7**				
PIP5K2A	phosphatidylinositol-4-phosphate 5-kinase, type II, alpha			1.5	**1.5**	1.3		1.3	**1.3**				
PIP5K2B	phosphatidylinositol-4-phosphate 5-kinase, type II, beta							-1.4		-1.6	**-1.6**	1.8	**1.8**
PIP5K3	phosphatidylinositol-3-phosphate/phosphatidylinositol 5-kinase, type III			1.4				-1.6	**-1.6**			1.3	**1.3**
PIR	pirin (iron-binding nuclear protein)					3.8	**3.8**	-1.2					
PISD	phosphatidylserine decarboxylase	-1.3		-1.3								-1.5	**-1.5**
PITPNA	phosphatidylinositol transfer protein, alpha			1.4				-2.0	**-2.0**	-1.3	**-1.3**	2.0	**2.0**
PITPNB	phosphatidylinositol transfer protein, beta	-1.3						-1.5	**-1.5**	-1.5		-1.3	**-1.3**
PITPNM1	phosphatidylinositol transfer protein, membrane-associated 1					1.2							
PITRM1	pitrilysin metallopeptidase 1	1.8	**1.8**	2.6	**2.6**	1.4		1.6	**1.6**			-2.0	**-2.0**
PKD1	polycystic kidney disease 1 (autosomal dominant)			-1.2				-3.0	**-3.0**	3.2	**3.2**	18.6	**18.6**
PKD2	polycystic kidney disease 2 (autosomal dominant)			1.2				-2.0	**-2.0**			2.5	**2.5**
PKIA	protein kinase (cAMP-dependent, catalytic) inhibitor alpha	1.5	**1.5**	1.4	**1.4**	-1.7	**-1.5**	-6.9	**-6.9**			7.8	**7.8**
PKM2	pyruvate kinase, muscle			-1.3				-3.2	**-3.2**	-2.3	**-2.3**	1.2	
PKMYT1	protein kinase, membrane associated tyrosine/threonine 1			-1.4		1.2		-2.2	**-2.2**	1.2	**1.2**		
PKN1	protein kinase N1							1.2					
PKN2	protein kinase N2	1.3		2.4				1.4	**1.4**	1.4	**1.4**	1.7	**1.3**
PKP4	plakophilin 4					-1.2		2.5	**2.5**	-1.3		-2.9	**-2.9**
PLAG1	pleiomorphic adenoma gene 1			1.4		1.2		-5.2	**-5.2**	1.4	**1.4**	11.9	**11.9**
PLAGL1	pleiomorphic adenoma gene-like 1	1.6	**1.6**	1.2	**1.2**			15.8	**15.8**			-13.0	**-13.0**
PLAGL2	pleiomorphic adenoma gene-like 2									-1.2		-1.3	**-1.3**
PLCB1	phospholipase C, beta 1 (phosphoinositide-specific)	-1.3		-1.3		-1.2		-7.4	**-7.4**	-1.5	**-1.5**	4.0	**4.0**
PLCB2	phospholipase C, beta 2	1.3	**1.3**										
PLCG1	phospholipase C, gamma 1			1.3				1.9	**1.9**	1.9	**1.9**	1.2	
PLCG2	phospholipase C, gamma 2 (phosphatidylinositol-specific)							-1.3	**-1.3**	-1.6	**-1.6**	-1.5	**-1.5**
PLCH2	phospholipase C, eta 2												
PLCL1	phospholipase C-like 1			1.9									
PLCL2	phospholipase C-like 2	1.5						-1.6	**-1.6**	2.2	**1.7**		
PLD3	phospholipase D family, member 3	1.3	**1.3**					2.5				1.3	
PLEKHB2	pleckstrin homology domain containing, family B (evectins) member 2					-1.4		1.3	**1.3**	1.3	**1.3**	-1.5	**-1.5**
PLEKHC1	pleckstrin homology domain containing, family C (with FERM domain) member 1	1.6	**1.6**	1.4	**1.4**					3.1	**3.1**	65.9	**65.9**
PLEKHE1	PH domain and leucine rich repeat protein phosphatase	1.3						-1.6	**-1.6**			-1.3	**-1.3**
PLK1	polo-like kinase 1 (Drosophila)					1.4		-1.6	**-1.6**	1.5	**1.5**	2.3	**2.3**
PLK4	polo-like kinase 4 (Drosophila)			1.4						1.7	**1.7**	-1.6	**-1.6**
PLOD3	procollagen-lysine, 2-oxoglutarate 5-dioxygenase 3							1.2					
PLP2	proteolipid protein 2 (colonic epithelium-enriched)			1.3	**1.3**			-3.3	**-3.3**	1.5	**1.5**	8.3	**8.3**
PLS1	plastin 1 (I isoform)					-1.2		9.1	**9.1**	-3.2	**-3.2**	-21.7	**-21.7**
PLSCR1	phospholipid scramblase 1	1.3				-1.4		1.8	**1.8**	1.5	**1.5**	-1.4	**-1.4**
PLTP	phospholipid transfer protein	1.3		1.3	**1.3**			13.4	**13.4**			-30.8	**-30.8**
PLXNB2	plexin B2					1.5		-3.9	**-3.9**	1.3	**1.3**	8.5	**8.5**
PMAIP1	phorbol-12-myristate-13-acetate-induced protein 1	-1.5		-1.7		-2.1	**-2.1**	-3.7	**-3.7**	1.5	**1.5**	2.3	**2.3**
PMF1	polyamine-modulated factor 1							-1.6	**-1.6**				
PML	promyelocytic leukemia							1.8	**1.8**			1.4	**1.4**
PMM1	phosphomannomutase 1	1.3											

PMM2	phosphomannomutase 2	-1.5		-1.4	**-1.4**								
PMPCA	peptidase (mitochondrial processing) alpha							1.2		-1.4	**-1.4**	-1.6	**-1.6**
PMPCB	peptidase (mitochondrial processing) beta									1.2		1.4	**1.4**
PMS1	PMS1 postmeiotic segregation increased 1 (S. cerevisiae)	-1.4		-1.5		-1.3		2.5	**2.5**	-1.3	**-1.3**	-1.7	**-1.7**
PMS2	PMS2 postmeiotic segregation increased 2 (S. cerevisiae)			-1.6	**-1.6**					-1.3	**-1.3**		
PMS2L3	postmeiotic segregation increased 2-like 3			-1.4		-1.6		-1.7	**-1.7**			1.7	**1.7**
PMVK	phosphomevalonate kinase	-1.3		-1.2	**-1.2**	-1.2		-1.3	**-1.3**			-1.4	**-1.4**
PNMA2	paraneoplastic antigen MA2												
PNMT	phenylethanolamine N-methyltransferase					3.9	**3.9**						
PNN	pinin, desmosome associated protein	-1.3		-1.5	**-1.5**	-1.5	**-1.5**	1.8	**1.8**	-1.5	**-1.5**	-2.6	**-2.6**
PNRC1	proline-rich nuclear receptor coactivator 1	1.2		2.2	**2.2**			2.0	**2.0**	1.4		1.5	**1.5**
PODXL	podocalyxin-like	-1.3	**-1.3**					-1.9	**-1.9**	-2.8	**-2.8**	-1.4	**-1.4**
POGZ	pogo transposable element with ZNF domain									1.3		2.6	**2.6**
POLA	polymerase (DNA directed), alpha 1							-1.5	**-1.5**	-2.7	**-2.7**	-2.7	**-2.7**
POLA2	polymerase (DNA directed), alpha 2 (70kD subunit)					-1.3		-1.4	**-1.4**	1.3	**1.3**		
POLB	polymerase (DNA directed), beta			1.5		-1.2		1.3	**1.3**	1.8	**-1.5**	1.5	**-1.4**
POLD1	polymerase (DNA directed), delta 1, catalytic subunit 125kDa			-2.8						-1.4	**-1.4**	-2.5	**-2.5**
POLD2	polymerase (DNA directed), delta 2, regulatory subunit 50kDa							1.9	**1.9**	-1.9	**-1.9**	-13.9	**-13.9**
POLD3	polymerase (DNA-directed), delta 3, accessory subunit			-1.2				-1.6	**-1.6**	-1.8	**-1.8**	-2.2	**-2.2**
POLD4	polymerase (DNA-directed), delta 4	1.2		1.4	**1.4**			-1.4				3.1	**3.1**
POLDIP3	polymerase (DNA-directed), delta interacting protein 3							-1.2					
POLE	polymerase (DNA directed), epsilon			-1.2				-1.4	**-1.4**			-3.9	**-3.9**
POLE2	polymerase (DNA directed), epsilon 2 (p59 subunit)	-1.2		-1.3		-1.7		-1.8	**-1.8**	-2.1	**-2.1**	-2.0	**-2.0**
POLE3	polymerase (DNA directed), epsilon 3 (p17 subunit)					-1.3		-1.5	**-1.5**	-2.0	**-2.0**	-2.0	**-2.0**
POLG	polymerase (DNA directed), gamma			-1.6	**-1.6**	-1.5				1.4	**1.4**	1.5	**1.5**
POLG2	polymerase (DNA directed), gamma 2, accessory subunit							1.7	**1.7**	-1.3	**-1.3**	-2.6	**-2.6**
POLR1C	polymerase (RNA) I polypeptide C, 30kDa	-1.6		-4.2	**-4.2**	-1.7		1.3		-1.7	**-1.7**	-2.9	**-2.9**
POLR2A	polymerase (RNA) II (DNA directed) polypeptide A, 220kDa							-2.4	**-2.4**			2.2	**2.2**
POLR2B	polymerase (RNA) II (DNA directed) polypeptide B, 140kDa							2.6	**2.6**			-3.0	**-3.0**
POLR2C	polymerase (RNA) II (DNA directed) polypeptide C, 33kDa	1.3				1.3				-1.4	**-1.4**	1.6	
POLR2D	polymerase (RNA) II (DNA directed) polypeptide D	-1.3		-1.3	**-1.3**	-1.8	**-1.8**	-1.2		-1.3		-2.0	**-2.0**
POLR2E	polymerase (RNA) II (DNA directed) polypeptide E, 25kDa			-1.4	**-1.4**	-1.4		-1.6	**-1.5**	-1.9	**-1.9**	-1.6	**-1.6**
POLR2F	polymerase (RNA) II (DNA directed) polypeptide F	-1.3		-1.3	**-1.3**	-1.2						-2.3	**-2.3**
POLR2G	polymerase (RNA) II (DNA directed) polypeptide G					-1.3		-1.7	**-1.7**	-1.4	**-1.4**		
POLR2H	polymerase (RNA) II (DNA directed) polypeptide H	-1.6	**-1.6**	-1.5	**-1.5**	-1.4		1.4	**1.4**	-1.9	**-1.9**	-2.9	**-2.9**
POLR2I	polymerase (RNA) II (DNA directed) polypeptide I, 14.5kDa	-1.4		-1.9	**-1.9**	-1.5		-1.6	**-1.6**	-1.8	**-1.8**	-1.7	**-1.7**
POLR2J	polymerase (RNA) II (DNA directed) polypeptide J, 13.3kDa			-1.3				-1.3	**-1.3**	-1.2			
POLR2K	polymerase (RNA) II (DNA directed) polypeptide K, 7.0kDa					-1.5		-2.5	**-2.5**			1.7	**1.7**
POLR2L	polymerase (RNA) II (DNA directed) polypeptide L, 7.6kDa			-1.2		-1.2						-2.4	**-2.4**
POLR3C	polymerase (RNA) III (DNA directed) polypeptide C (62kD)	-1.3		-1.6		-1.2		-1.8	**-1.8**			-1.4	**-1.4**
POLR3D	polymerase (RNA) III (DNA directed) polypeptide D, 44kDa											-1.2	
POLR3F	polymerase (RNA) III (DNA directed) polypeptide F, 39 kDa	-1.4				-1.6	**-1.6**	1.3				-1.8	**-1.8**
POLR3G	polymerase (RNA) III (DNA directed) polypeptide G (32kD)	-1.7				-1.3		1.3	**1.3**	-3.0	**-3.0**	-3.8	**-3.8**
POLRMT	polymerase (RNA) mitochondrial (DNA directed)	-1.2		-1.5								-1.8	**-1.8**
POLS	polymerase (DNA directed) sigma	1.3		3.6	**3.6**	1.9						1.4	**1.4**
PON2	paraoxonase 2	1.4	**1.4**	1.3		1.3		43.3	**43.3**	19.7	**19.7**	-40.8	**-40.8**
POP4	processing of precursor 4, ribonuclease P/MRP subunit (S. cerevisiae)			-1.4	**-1.4**			-1.3	**-1.3**				
POP5	processing of precursor 5, ribonuclease P/MRP subunit (S. cerevisiae)									-1.6	**-1.6**	-1.3	
POP7	processing of precursor 7, ribonuclease P subunit (S. cerevisiae)							-1.5		-2.0	**-2.0**	-3.0	**-3.0**
PORIMIN	transmembrane protein 123	1.3	**1.3**	1.3	**1.3**	1.3		1.2					
POU2AF1	POU domain, class 2, associating factor 1					-1.9	**-1.9**	-56.3	**-56.3**			25.8	**25.8**
POU2F1	POU domain, class 2, transcription factor 1									-2.0	**-2.0**	-1.7	**-1.7**
POU3F2	POU domain, class 3, transcription factor 2					-1.9	**-1.9**						
POU4F1	POU domain, class 4, transcription factor 1					1.4				-1.7	**-1.7**	-1.2	
PPAP2A	phosphatidic acid phosphatase type 2A									1.2		2.0	**2.0**
PPARBP	PPAR binding protein	-1.3				-1.2		1.8	**1.8**	-1.5	**-1.5**	-2.0	**-2.0**
PPAT	phosphoribosyl pyrophosphate amidotransferase	-1.7	**-1.7**	-3.0	**-3.0**	-2.2		2.0	**2.0**	-2.0	**-2.0**	-4.7	**-4.7**
PPFIA1	protein tyrosine phosphatase, receptor type, f polypeptide (PTPRF), interacting protein (liprin), alpha 1	1.2		1.2				1.3	**1.3**			-1.4	**-1.4**

PPGB	cathepsin A			1.3	**1.3**								
PPIA	peptidylprolyl isomerase A (cyclophilin A)							1.9	**1.9**	1.5	**-1.2**	-2.8	**-2.8**
PPIB	peptidylprolyl isomerase B (cyclophilin B)							1.5	**1.5**			-1.7	**-1.7**
PPID	peptidylprolyl isomerase D (cyclophilin D)					-1.3		1.5		-1.5		-1.6	**-1.6**
PPIE	peptidylprolyl isomerase E (cyclophilin E)			-1.2		-2.2						-1.6	**-1.6**
PPIF	peptidylprolyl isomerase F (cyclophilin F)			-1.5	**-1.5**	-1.4		-1.3	**-1.3**	-2.6	**-2.6**	-4.9	**-4.9**
PPIG	peptidylprolyl isomerase G (cyclophilin G)					-1.4		1.8	**1.8**	-1.3	**-1.3**	-2.7	**-2.7**
PPIH	peptidylprolyl isomerase H (cyclophilin H)	-1.3		-1.2	**-1.2**					-1.7	**-1.7**	-2.0	**-2.0**
PPIL2	peptidylprolyl isomerase (cyclophilin)-like 2			1.9		1.4							
PPM1A	protein phosphatase 1A (formerly 2C), magnesium-dependent, alpha isoform							-1.7	**-1.7**	1.5	**1.5**	1.9	**1.9**
PPM1B	protein phosphatase 1B (formerly 2C), magnesium-dependent, beta isoform			1.3				1.3	**1.3**	1.4	**1.4**	1.7	**1.7**
PPM1D	protein phosphatase 1D magnesium-dependent, delta isoform					1.3						-1.4	**-1.4**
PPM1E	protein phosphatase 1E (PP2C domain containing)					-1.4		-1.2					
PPM1G	protein phosphatase 1G (formerly 2C), magnesium-dependent, gamma isoform	-1.2										-2.2	**-2.2**
PPOX	protoporphyrinogen oxidase									-1.2			
PPP1CA	protein phosphatase 1, catalytic subunit, alpha isoform							-2.1	**-2.1**			1.5	**1.5**
PPP1CC	protein phosphatase 1, catalytic subunit, gamma isoform	-1.2				-1.3						-1.5	**-1.5**
PPP1R10	protein phosphatase 1, regulatory subunit 10					1.2	**1.2**	1.4	**1.4**	1.7			
PPP1R11	protein phosphatase 1, regulatory (inhibitor) subunit 11					-1.2		-1.6	**-1.6**			1.6	**1.6**
PPP1R12A	protein phosphatase 1, regulatory (inhibitor) subunit 12A							1.4	**1.4**	1.7	**1.7**	1.8	**1.8**
PPP1R12B	protein phosphatase 1, regulatory (inhibitor) subunit 12B					1.3							
PPP1R13B	protein phosphatase 1, regulatory (inhibitor) subunit 13B	1.3		1.9	**1.9**	3.6	**3.6**						
PPP1R2	protein phosphatase 1, regulatory (inhibitor) subunit 2							2.1	**2.1**	1.4	**1.4**	1.4	**1.4**
PPP1R3D	protein phosphatase 1, regulatory subunit 3D					1.2		2.1	**2.1**			-1.7	**-1.7**
PPP1R7	protein phosphatase 1, regulatory subunit 7							-2.5	**-2.5**	1.3		2.0	**2.0**
PPP1R8	protein phosphatase 1, regulatory (inhibitor) subunit 8					-1.7				-1.4		-1.3	
PPP2CA	protein phosphatase 2 (formerly 2A), catalytic subunit, alpha isoform							-1.4	**-1.4**				
PPP2CB	protein phosphatase 2 (formerly 2A), catalytic subunit, beta isoform			1.4	**1.4**			1.5	**1.5**	1.2	**1.2**		
PPP2R1A	protein phosphatase 2 (formerly 2A), regulatory subunit A (PR 65), alpha isoform							-1.2		-1.4	**-1.4**	1.3	
PPP2R1B	protein phosphatase 2 (formerly 2A), regulatory subunit A (PR 65), beta isoform												
PPP2R1B	--	-1.5				-1.4		-1.7	**-1.7**	-1.4	**-1.4**	-1.3	
PPP2R2A	protein phosphatase 2 (formerly 2A), regulatory subunit B (PR 52), alpha isoform	-1.3				-1.4		2.4	**2.4**	1.7	**1.7**	-1.9	**-1.9**
PPP2R4	protein phosphatase 2A, regulatory subunit B' (PR 53)							-1.5	**-1.2**	1.3		1.3	
PPP2R5A	protein phosphatase 2, regulatory subunit B (B56), alpha isoform							-1.2	**-1.2**	1.9	**1.9**	1.3	**1.3**
PPP2R5C	protein phosphatase 2, regulatory subunit B (B56), gamma isoform	1.5		1.7	**1.7**	1.5		-25.3	**-25.3**			5.3	**5.3**
PPP2R5D	protein phosphatase 2, regulatory subunit B (B56), delta isoform	1.2				1.4	**1.4**						
PPP2R5E	protein phosphatase 2, regulatory subunit B (B56), epsilon isoform					-1.2		2.4	**2.4**	1.2	**1.2**	-4.4	**-4.4**
PPP3CA	protein phosphatase 3 (formerly 2B), catalytic subunit, alpha isoform (calcineurin A alpha)			1.2				1.9	**1.9**	-1.3	**-1.3**	1.7	**1.7**
PPP3CB	protein phosphatase 3 (formerly 2B), catalytic subunit, beta isoform (calcineurin A beta)	-1.3				-1.4		1.7	**1.7**	1.6	**1.6**	-1.8	**-1.8**
PPP3CC	protein phosphatase 3 (formerly 2B), catalytic subunit, gamma isoform (calcineurin A gamma)					1.7				1.8	**1.8**	1.8	**1.8**
PPP4C	protein phosphatase 4 (formerly X), catalytic subunit			1.3	**1.3**			-1.8	**-1.8**			1.8	**1.8**
PPP4R1	protein phosphatase 4, regulatory subunit 1									1.2		1.5	**1.5**
PPP5C	protein phosphatase 5, catalytic subunit			-1.4	**-1.4**	1.4						1.3	
PPP6C	protein phosphatase 6, catalytic subunit					-1.2		-1.8	**-1.8**			1.3	**1.3**
PPRC1	peroxisome proliferator-activated receptor gamma, coactivator-related 1	-1.8		-1.5				1.7	**1.7**	-2.4	**-2.4**	-4.7	**-4.7**
PPT1	palmitoyl-protein thioesterase 1 (ceroid-lipofuscinosis, neuronal 1, infantile)					-1.3		1.7	**1.7**	-1.3	**-1.3**	-2.2	**-2.2**
PPT2	palmitoyl-protein thioesterase 2	1.3				1.4						-2.3	**-2.3**
PQBP1	polyglutamine binding protein 1							1.3	**1.3**	1.3	**1.3**	-1.5	**-1.5**
PRCC	papillary renal cell carcinoma (translocation-associated)												
PRDM2	PR domain containing 2, with ZNF domain									1.3		1.9	**1.9**
PRDX1	peroxiredoxin 1	-1.3	**-1.3**	-1.6	**-1.6**	-1.4		-2.5	**-2.5**	-1.7	**-1.7**		
PRDX2	peroxiredoxin 2							-8.5	**-8.5**	-3.1	**-3.1**	3.4	**3.4**
PRDX3	peroxiredoxin 3	-1.2		-1.3	**-1.3**	-1.6	**-1.6**			-1.8	**-1.8**	-1.8	**-1.8**
PRDX4	peroxiredoxin 4	-1.2	**-1.2**	-1.5	**-1.5**	-1.4		1.3	**1.3**	-2.5	**-2.5**	-3.3	**-3.3**
PRDX6	peroxiredoxin 6			1.2				1.5	**1.5**	1.4	**1.4**	1.4	**-1.3**
PREI3	preimplantation protein 3			-1.2		-1.2		1.5	**1.5**	1.3	**1.3**	-1.7	**-1.7**
PREP	prolyl endopeptidase							1.5	**1.5**			-1.8	**-1.8**
PRG1	proteoglycan 1, secretory granule	2.6		4.5	**4.5**	3.2	**3.2**	2.7	**2.7**	1.5	**1.5**	2.7	**2.7**

PRIM1	primase, polypeptide 1, 49kDa			-1.5	**-1.5**	-1.2		-1.3	**-1.3**	-1.8	**-1.8**	-2.3	**-2.3**
PRIM2A	primase, polypeptide 2A, 58kDa			-1.2	**-1.2**			1.4	**1.4**			-1.4	**-1.4**
PRKAA1	protein kinase, AMP-activated, alpha 1 catalytic subunit							1.3	**1.3**	1.6	**1.6**	-1.3	
PRKAB1	protein kinase, AMP-activated, beta 1 non-catalytic subunit	-1.4						1.5	**1.5**				
PRKAB2	protein kinase, AMP-activated, beta 2 non-catalytic subunit	1.7						5.1	**5.1**	-1.5		2.3	**2.3**
PRKACB	protein kinase, cAMP-dependent, catalytic, beta			1.8	**1.8**			2.4	**2.4**	-2.7	**-2.7**	-1.6	**-1.6**
PRKAG1	protein kinase, AMP-activated, gamma 1 non-catalytic subunit							1.2	**1.2**			1.3	
PRKAR1A	protein kinase, cAMP-dependent, regulatory, type I, alpha (tissue specific extinguisher 1)	1.2		1.8	**1.8**			2.0	**2.0**	1.4	**1.4**	-1.3	
PRKAR1B	protein kinase, cAMP-dependent, regulatory, type I, beta	-1.6	**-1.6**	-1.3	**-1.3**	-1.2							
PRKAR2B	protein kinase, cAMP-dependent, regulatory, type II, beta	1.4	**1.4**	2.0	**2.0**			-1.4		1.4		1.9	**1.9**
PRKCA	protein kinase C, alpha	1.6	**1.6**	1.5				6.6	**6.6**	1.3	**1.3**	-7.0	**-7.0**
PRKCABP	protein interacting with PRKCA 1					1.3							
PRKCB1	protein kinase C, beta 1	1.2		2.3	**2.3**	-1.2		20.7	**20.7**	1.6	**1.6**	-18.3	**-18.3**
PRKCBP1	protein kinase C binding protein 1					1.7		-1.2	**-1.2**	1.4	**1.4**	1.6	**1.6**
PRKCD	protein kinase C, delta	1.6										6.3	**6.3**
PRKCH	protein kinase C, eta	-1.6						3.4	**3.4**	3.7	**3.7**	-2.4	**-2.4**
PRKCI	protein kinase C, iota	1.2		2.0	**2.0**	-1.4	**-1.4**	1.2				3.5	**3.5**
PRKCQ	protein kinase C, theta							1.6	**1.6**			-2.4	**-2.4**
PRKCSH	protein kinase C substrate 80K-H												
PRKD2	protein kinase D2							-3.2	**-3.2**	1.8	**1.8**	4.8	**4.8**
PRKDC	protein kinase, DNA-activated, catalytic polypeptide							-1.3	**-1.2**	-1.6	**-1.6**	-2.1	**-2.1**
PRKRA	protein kinase, interferon-inducible double stranded RNA dependent activator	-1.3		-1.8	**-1.8**	-1.5		-1.9	**-1.9**	2.5	**2.5**	5.0	**5.0**
PRKRIR	protein-kinase, interferon-inducible double stranded RNA dependent inhibitor, repressor of (P58 repressor)	-1.2	**-1.2**	1.3						-1.6	**-1.6**	-1.4	**-1.4**
PRKX	protein kinase, X-linked	2.0				-1.2		-2.3	**-2.3**	3.4	**3.4**	5.3	**5.3**
PRKY	protein kinase, Y-linked			-1.5								1.4	
PRMT3	protein arginine methyltransferase 3	-1.6	**-1.6**	-2.4	**-2.4**	-2.1	**-2.1**	-1.3	**-1.3**	-2.4	**-2.4**	-2.8	**-2.8**
PRNP	prion protein (p27-30) (Creutzfeldt-Jakob disease, Gerstmann-Strausler-Scheinker syndrome, fatal familial insomnia)							-1.4	**-1.4**	-1.3			
PRNPIP	prion protein interacting protein			-1.6	**-1.6**					-1.3		-2.5	**-2.5**
PROCR	protein C receptor, endothelial (EPCR)					-2.2	**-2.2**	6.6	**6.6**	-1.5	**-1.5**	-12.1	**-12.1**
PROSC	proline synthetase co-transcribed homolog (bacterial)	-1.3		1.3		-1.2						-1.5	**-1.5**
PRPF18	PRP18 pre-mRNA processing factor 18 homolog (S. cerevisiae)			1.2		-1.3							
PRPF19	PRP19/PSO4 pre-mRNA processing factor 19 homolog (S. cerevisiae)	-1.3						-1.3	**-1.3**	-1.9	**-1.9**	-2.2	**-2.2**
PRPF3	PRP3 pre-mRNA processing factor 3 homolog (S. cerevisiae)					-1.6							
PRPF31	PRP31 pre-mRNA processing factor 31 homolog (S. cerevisiae)			1.3	**1.3**	-1.6	**-1.6**	-1.6	**-1.2**			-2.1	**-2.1**
PRPF4	PRP4 pre-mRNA processing factor 4 homolog (yeast)			-1.4	**-1.3**	-1.5		-1.3	**-1.3**	-2.2	**-2.2**	-2.1	**-2.1**
PRPF4B	PRP4 pre-mRNA processing factor 4 homolog B (yeast)	1.4						1.3				-1.3	**-1.3**
PRPF8	PRP8 pre-mRNA processing factor 8 homolog (S. cerevisiae)			-1.3	**-1.3**			-1.4	**-1.4**	-1.3	**-1.3**		
PRPH	peripherin			1.4		1.3							
PRPS1	phosphoribosyl pyrophosphate synthetase 1	-1.5	**-1.5**			-1.4		-2.1	**-2.1**	-2.6	**-2.6**	-2.3	**-2.3**
PRPS2	phosphoribosyl pyrophosphate synthetase 2					-1.4		1.3	**1.3**			-2.1	**-2.1**
PRPSAP1	phosphoribosyl pyrophosphate synthetase-associated protein 1			1.3				1.9	**1.9**			-1.3	**-1.3**
PRPSAP2	phosphoribosyl pyrophosphate synthetase-associated protein 2					-1.3		-1.8	**-1.8**	-1.5		1.3	
PRSS25	HtrA serine peptidase 2							1.5	**1.5**	-1.5	**-1.5**	-1.9	**-1.9**
PRSS3	protease, serine, 3 (mesotrypsin)					1.4							
PRSS8	protease, serine, 8 (prostasin)			1.3	**1.3**	1.5							
PRUNE	prune homolog (Drosophila)					-1.3		-1.5	**-1.5**	-1.9	**-1.9**		
PSAP	prosaposin (variant Gaucher disease and variant metachromatic leukodystrophy)					1.3		3.1	**3.1**	-1.4	**-1.4**	-1.4	
PSCD1	pleckstrin homology, Sec7 and coiled-coil domains 1(cytohesin 1)							-1.2		1.3		1.9	**1.9**
PSCD2	pleckstrin homology, Sec7 and coiled-coil domains 2 (cytohesin-2)					1.3		-1.3		1.4	**1.4**	1.8	**1.8**
PSEN1	presenilin 1 (Alzheimer disease 3)	1.7	**1.7**	1.8	**1.8**	1.5	**1.5**	1.3	**1.3**	1.5	**1.5**	2.0	**2.0**
PSF1	GINS complex subunit 1 (Psf1 homolog)			-1.5		-1.3		-1.9	**-1.9**	-1.6	**-1.6**	-2.3	**-2.3**
PSG1	pregnancy specific beta-1-glycoprotein 1			1.3				-1.2		-1.4			
PSIP1	PC4 and SFRS1 interacting protein 1	1.3						-2.0	**-2.0**	-1.5	**-1.5**	2.5	**2.5**
PSKH1	protein serine kinase H1			1.3		1.4							
PSMA1	proteasome (prosome, macropain) subunit, alpha type, 1	1.3						-1.2	**-1.2**	-1.2		-1.3	**-1.3**
PSMA2	proteasome (prosome, macropain) subunit, alpha type, 2	-1.3				-1.2		3.5	**3.5**	-1.3	**-1.3**	-5.0	**-5.0**
PSMA3	proteasome (prosome, macropain) subunit, alpha type, 3					-1.4		1.3	**1.3**	-1.7	**-1.7**	-1.7	**-1.7**
PSMA4	proteasome (prosome, macropain) subunit, alpha type, 4					-1.3				-1.3	**-1.3**	-1.5	**-1.5**

PSMA5	proteasome (prosome, macropain) subunit, alpha type, 5	-1.2		-1.2		-1.4	**-1.4**			-1.4	**-1.4**	-2.3	**-2.3**
PSMA6	proteasome (prosome, macropain) subunit, alpha type, 6	1.2		1.4				-1.3	**-1.3**			1.3	**1.3**
PSMB1	proteasome (prosome, macropain) subunit, beta type, 1					-1.2						-1.5	**-1.5**
PSMB10	proteasome (prosome, macropain) subunit, beta type, 10			-1.2	**-1.2**	-1.2		-3.7	**-3.7**			1.5	**1.5**
PSMB2	proteasome (prosome, macropain) subunit, beta type, 2							-1.2	**-1.2**	-1.6	**-1.6**	-1.5	**-1.5**
PSMB3	proteasome (prosome, macropain) subunit, beta type, 3									-1.2	**-1.2**	-1.8	**-1.8**
PSMB4	proteasome (prosome, macropain) subunit, beta type, 4			1.2		-1.3		1.3	**1.3**	-1.4	**-1.4**	-1.3	**-1.2**
PSMB5	proteasome (prosome, macropain) subunit, beta type, 5	-1.4	**-1.4**	-1.4	**-1.4**	-1.4	**-1.4**	1.5	**1.5**	-1.4	**-1.4**	-2.2	**-2.2**
PSMB6	proteasome (prosome, macropain) subunit, beta type, 6			-1.2		-1.3		-1.6	**-1.6**				
PSMB7	proteasome (prosome, macropain) subunit, beta type, 7					-1.3						-1.8	**-1.8**
PSMB8	proteasome (prosome, macropain) subunit, beta type, 8 (large multifunctional peptidase 7)					-1.2		-18.9	**-18.9**			16.2	**16.2**
PSMB9	proteasome (prosome, macropain) subunit, beta type, 9 (large multifunctional peptidase 2)					-1.2		-202.7	**-202.7**	-1.3	**-1.3**	22.2	**22.2**
PSMC1	proteasome (prosome, macropain) 26S subunit, ATPase, 1					-1.5							
PSMC2	proteasome (prosome, macropain) 26S subunit, ATPase, 2			-1.2		-1.3						-1.5	**-1.5**
PSMC3	proteasome (prosome, macropain) 26S subunit, ATPase, 3			-1.2		-1.5		-1.4	**-1.4**			-1.5	**-1.5**
PSMC4	proteasome (prosome, macropain) 26S subunit, ATPase, 4					-1.3		1.3		-1.3	**-1.3**	-2.9	**-2.9**
PSMC5	proteasome (prosome, macropain) 26S subunit, ATPase, 5							1.6	**1.6**			-2.6	**-2.6**
PSMC6	proteasome (prosome, macropain) 26S subunit, ATPase, 6					-1.2		1.3	**1.3**	1.3		-1.4	**-1.4**
PSMD1	proteasome (prosome, macropain) 26S subunit, non-ATPase, 1							-1.6	**-1.6**	-1.4	**-1.4**	-1.4	
PSMD10	proteasome (prosome, macropain) 26S subunit, non-ATPase, 10	-1.2		-1.2		-1.4		-1.2	**-1.2**			-1.2	**-1.2**
PSMD11	proteasome (prosome, macropain) 26S subunit, non-ATPase, 11					-1.4						-2.1	**-1.6**
PSMD12	proteasome (prosome, macropain) 26S subunit, non-ATPase, 12			-1.5		-1.4		1.6	**1.6**			-2.2	**-2.2**
PSMD13	proteasome (prosome, macropain) 26S subunit, non-ATPase, 13									1.4		-1.9	**-1.9**
PSMD14	proteasome (prosome, macropain) 26S subunit, non-ATPase, 14					-1.4		1.3	**1.3**			-2.4	**-2.4**
PSMD2	proteasome (prosome, macropain) 26S subunit, non-ATPase, 2							1.3	**1.3**			-1.8	**-1.8**
PSMD3	proteasome (prosome, macropain) 26S subunit, non-ATPase, 3			-1.4						-1.5	**-1.5**	-2.0	**-2.0**
PSMD4	proteasome (prosome, macropain) 26S subunit, non-ATPase, 4					-1.3		1.3	**1.3**			-1.6	**-1.6**
PSMD5	proteasome (prosome, macropain) 26S subunit, non-ATPase, 5							3.0	**3.0**	-1.2		-2.5	**-2.5**
PSMD6	proteasome (prosome, macropain) 26S subunit, non-ATPase, 6					-1.6				-1.2	**-1.2**		
PSMD7	proteasome (prosome, macropain) 26S subunit, non-ATPase, 7 (Mov34 homolog)					-1.4				-1.3		-1.2	
PSMD8	proteasome (prosome, macropain) 26S subunit, non-ATPase, 8	-1.2		-1.3	**-1.3**			-1.5	**-1.5**	-1.3	**-1.3**		
PSMD9	proteasome (prosome, macropain) 26S subunit, non-ATPase, 9	-1.2				-1.2				1.3			
PSME1	proteasome (prosome, macropain) activator subunit 1 (PA28 alpha)					-1.2		-1.9	**-1.9**	-1.8	**-1.8**		
PSME2	proteasome (prosome, macropain) activator subunit 2 (PA28 beta)			-1.3	**-1.3**			-2.4	**-2.4**	-1.8	**-1.8**		
PSME3	proteasome (prosome, macropain) activator subunit 3 (PA28 gamma; Ki)			-1.4	**-1.4**	-1.4	**-1.4**	1.5	**1.5**	-2.2	**-2.2**	-2.6	**-2.6**
PSPH	phosphoserine phosphatase	-1.6				-1.9		3.3	**3.3**	-2.0	**-2.0**	-8.6	**-8.6**
PTBP1	polypyrimidine tract binding protein 1									-1.5	**-1.5**	-2.1	**-2.1**
PTDSR	phosphatidylserine receptor	1.3		1.5	**1.5**	-1.4				1.4	**1.4**	1.3	
PTEN	phosphatase and tensin homolog (mutated in multiple advanced cancers 1)	2.3	**2.3**	2.3	**2.3**	1.5		4.2	**4.2**	-1.5	**-1.3**	-4.7	**-4.7**
PTGER3	prostaglandin E receptor 3 (subtype EP3)			1.2	**1.2**			-1.5		1.7	**1.7**	1.2	
PTK2B	PTK2B protein tyrosine kinase 2 beta	2.1	**2.1**	3.1	**3.1**	4.2	**4.2**	-1.3	**-1.3**			1.9	**1.9**
PTK7	PTK7 protein tyrosine kinase 7	1.2	**1.2**							-1.2			
PTK9L	twinfilin, actin-binding protein, homolog 2 (Drosophila)											1.5	
PTMA	prothymosin, alpha (gene sequence 28)			-1.2		-1.4		-1.7	**-1.7**	-1.3	**-1.3**	-1.2	
PTOV1	prostate tumor overexpressed gene 1			-1.5				-1.5		-1.8	**-1.8**		
PTP4A1	protein tyrosine phosphatase type IVA, member 1	-1.4				-1.4		-1.7	**-1.7**	-1.8	**-1.8**	-1.9	**-1.9**
PTP4A2	protein tyrosine phosphatase type IVA, member 2			1.3	**1.3**	1.2		1.3	**1.2**	1.5	**1.5**	-1.9	**-1.9**
PTPLB	protein tyrosine phosphatase-like (proline instead of catalytic arginine), member b			1.2				2.2	**2.2**	2.3	**2.3**	2.2	**2.2**
PTPN1	protein tyrosine phosphatase, non-receptor type 1	1.4		1.5	**1.5**	1.6	**1.6**	-1.8				3.0	**3.0**
PTPN11	protein tyrosine phosphatase, non-receptor type 11 (Noonan syndrome 1)	-1.6		1.5		-1.9		1.3	**1.3**	-1.6		-1.3	**-1.3**
PTPN12	protein tyrosine phosphatase, non-receptor type 12	1.3	**1.3**	1.3	**1.3**			-10.9	**-10.9**	2.4	**2.4**	75.8	**75.8**
PTPN18	protein tyrosine phosphatase, non-receptor type 18 (brain-derived)	1.4	**1.4**	1.5	**1.5**					1.3		1.4	**1.4**
PTPN2	protein tyrosine phosphatase, non-receptor type 2	-1.6	**-1.3**	-1.3		-2.1	**-2.1**	1.3	**1.3**	-1.2	**-1.2**	-1.6	**-1.6**
PTPN22	protein tyrosine phosphatase, non-receptor type 22 (lymphoid)							2.9	**2.9**	-2.0		-8.4	**-8.4**
PTPN3	protein tyrosine phosphatase, non-receptor type 3	1.3		2.4	**2.4**	1.3				1.4		15.7	**15.7**
PTPN4	protein tyrosine phosphatase, non-receptor type 4 (megakaryocyte)			1.3	**1.2**			-1.6	**-1.6**	-1.6	**-1.6**		
PTPN6	protein tyrosine phosphatase, non-receptor type 6	1.3	**1.3**	1.8		1.4		-5.5	**-5.5**			7.8	**7.8**
PTPN7	protein tyrosine phosphatase, non-receptor type 7							3.3	**3.3**	-1.5	**-1.5**	-4.7	**-4.7**

PTPN9	protein tyrosine phosphatase, non-receptor type 9	1.2		1.5	**1.5**							2.9	**2.9**
PTPRA	protein tyrosine phosphatase, receptor type, A							1.3	**1.3**	1.2		1.3	
PTPRC	protein tyrosine phosphatase, receptor type, C			2.4	**2.4**			-6.4	**-6.4**	-2.4		11.4	**11.4**
PTPRCAP	protein tyrosine phosphatase, receptor type, C-associated protein	1.2						-2.1	**-2.1**	1.5	**1.5**	3.6	**3.6**
PTPRD	protein tyrosine phosphatase, receptor type, D												
PTPRF	protein tyrosine phosphatase, receptor type, F			-1.2				91.0	**91.0**			-122.2	**-122.2**
PTPRK	protein tyrosine phosphatase, receptor type, K	-1.2						2.0	**2.0**	-2.9	**-2.9**	-3.3	**-3.3**
PTPRM	protein tyrosine phosphatase, receptor type, M	3.8	**3.8**	6.6	**6.6**	13.6	**13.6**	8.5	**8.5**			-19.3	**-19.3**
PTPRN2	protein tyrosine phosphatase, receptor type, N polypeptide 2					1.3							
PTS	6-pyruvoyltetrahydropterin synthase	-1.6	**-1.6**	-1.5	**-1.5**	-1.5		-2.4	**-2.4**	-1.3	**-1.3**	1.8	**1.8**
PTTG1	pituitary tumor-transforming 1					1.2		-2.1	**-2.1**			1.4	**1.4**
PTTG1IP	pituitary tumor-transforming 1 interacting protein			1.4	**1.4**	1.4		1.4	**1.4**	1.5	**1.5**		
PTTG2	pituitary tumor-transforming 2	1.3	**1.3**										
PUM2	pumilio homolog 2 (Drosophila)			1.4	**1.4**	1.3		-1.4	**-1.4**	-2.0		1.4	**1.4**
PURA	purine-rich element binding protein A					1.3		-1.3	**-1.3**			1.3	**1.3**
PVRL3	poliovirus receptor-related 3					-1.6	**-1.6**						
PXDN	peroxidasin homolog (Drosophila)	1.3								2.0	**2.0**		
PXMP3	peroxisomal membrane protein 3, 35kDa (Zellweger syndrome)			1.5	**1.5**	4.3	**4.3**	-1.7	**-1.7**	1.7	**1.7**	2.6	**2.6**
PYCR1	pyrroline-5-carboxylate reductase 1	-1.8		-2.1				-8.7	**-8.7**	-2.8	**-2.8**		
PYGB	phosphorylase, glycogen; brain			-1.3	**-1.3**								
QDPR	quinoid dihydropteridine reductase	1.2						2.2	**2.2**	-1.4		-3.8	**-3.8**
QKI	quaking homolog, KH domain RNA binding (mouse)					-1.5	**-1.5**	2.7	**2.7**	2.5	**2.5**	-1.7	**-1.7**
QP-C	ubiquinol-cytochrome c reductase, complex III subunit VII, 9.5kDa							-1.3		-1.2	**-1.2**	-1.4	**-1.4**
QPCT	glutaminyl-peptide cyclotransferase (glutaminyl cyclase)					1.8							
QPRT	quinolinate phosphoribosyltransferase (nicotinate-nucleotide pyrophosphorylase (carboxylating))					-1.3		-2.1	**-2.1**	-3.6	**-3.6**	1.5	**1.4**
QRICH1	glutamine-rich 1												
QSCN6	quiescin Q6												
RAB11A	RAB11A, member RAS oncogene family	-1.6				-1.3		-2.2	**-2.2**	1.3	**1.3**	1.5	**1.5**
RAB11B	RAB11B, member RAS oncogene family	1.3				1.2		-1.4	**-1.4**			1.3	
RAB11FIP2	RAB11 family interacting protein 2 (class I)	-1.2								-2.0	**-2.0**	1.2	
RAB14	RAB14, member RAS oncogene family							1.3	**1.3**	1.3	**1.3**		
RAB1A	RAB1A, member RAS oncogene family			1.4				1.5	**1.5**	1.3	**1.3**	-1.3	**-1.3**
RAB2	RAB2, member RAS oncogene family			1.2				-1.9	**-1.9**	1.2		2.1	**2.1**
RAB21	RAB21, member RAS oncogene family							1.3		1.5	**1.5**	1.3	**1.3**
RAB22A	RAB22A, member RAS oncogene family									1.2		2.1	**2.1**
RAB27A	RAB27A, member RAS oncogene family			1.3		-1.3		2.2	**2.2**	-1.8	**-1.8**	-3.5	**-3.5**
RAB31	RAB31, member RAS oncogene family			1.4				3.8	**3.8**			-13.0	**-13.0**
RAB33A	RAB33A, member RAS oncogene family	-1.2		-1.7	**-1.7**			6.8	**6.8**			-9.8	**-9.8**
RAB3GAP	RAB3 GTPase activating protein subunit 1 (catalytic)	1.5		1.4				1.8	**1.8**	-1.5	**-1.5**	-1.3	
RAB3-GAP150	RAB3 GTPase activating protein subunit 2 (non-catalytic)			-1.3		1.5		1.3	**1.3**			1.5	
RAB40B	RAB40B, member RAS oncogene family	-1.3	**-1.3**	-1.2				-1.7	**-1.7**	-1.5		-1.5	**-1.5**
RAB4A	RAB4A, member RAS oncogene family			1.3				3.8	**3.8**			-4.7	**-4.7**
RAB5A	RAB5A, member RAS oncogene family			1.4		-1.2		1.2	**1.2**	1.3	**1.3**	1.4	
RAB5B	RAB5B, member RAS oncogene family	1.6	**1.6**	1.8	**1.8**	1.5							
RAB5C	RAB5C, member RAS oncogene family			-1.3				1.2	**1.2**			-1.2	
RAB6A	RAB6A, member RAS oncogene family	-1.3		1.4	**1.4**	-1.2		-1.4	**-1.4**	1.5	**1.5**	1.7	**1.7**
RAB6IP2	ELKS/RAB6-interacting/CAST family member 1	1.8	**1.8**	1.6	**1.6**	1.4		-1.3	**-1.3**			5.3	**5.3**
RAB7L1	RAB7, member RAS oncogene family-like 1	1.2		1.4				2.1	**2.1**	-1.5	**-1.4**	-2.9	**-2.9**
RAB8A	RAB8A, member RAS oncogene family			1.3		-1.2		-1.5		-1.2			
RAB9A	RAB9, member RAS oncogene family					1.5		1.3	**1.3**				
RAB9P40	Rab9 effector protein with kelch motifs	-1.8	**-1.8**	-1.6	**-1.6**	-2.1	**-2.1**	1.9	**1.9**	-3.7	**-3.7**	-12.1	**-12.1**
RABAC1	Rab acceptor 1 (prenylated)							-1.5	**-1.5**			3.1	**3.1**
RABEP1	rabaptin, RAB GTPase binding effector protein 1	-1.2		-1.4				-2.1	**-2.1**			2.4	**2.4**
RABGGTA	Rab geranylgeranyltransferase, alpha subunit			-1.2				1.4		-1.2	**-1.2**	-1.5	**-1.5**
RABGGTB	Rab geranylgeranyltransferase, beta subunit	-1.3	**-1.3**	-1.3	**-1.3**	-1.7	**-1.7**	2.1	**2.1**	-1.8	**-1.8**	-1.8	**-1.8**
RABIF	RAB interacting factor	1.2		1.2				-1.2	**-1.2**			1.5	**1.5**
RAC1	ras-related C3 botulinum toxin substrate 1 (rho family, small GTP binding protein Rac1)					-1.2		-1.9	**-1.9**	1.4	**1.4**	2.0	**2.0**
RAC2	ras-related C3 botulinum toxin substrate 2 (rho family, small GTP binding protein Rac2)			1.6	**1.6**			-2.7	**-2.7**	1.2		1.3	**1.3**

RAC3	ras-related C3 botulinum toxin substrate 3 (rho family, small GTP binding protein Rac3)					1.7		9.3				-17.1	**-17.1**
RAD1	RAD1 homolog (S. pombe)					-1.5		-1.6	**-1.6**	-2.0	**-1.7**	-3.2	**-3.2**
RAD17	RAD17 homolog (S. pombe)	-1.3				-1.4		1.3		-2.1	**-2.1**	-1.3	**-1.2**
RAD21	RAD21 homolog (S. pombe)							-2.3	**-2.3**	1.3	**1.3**	2.5	**2.5**
RAD23A	RAD23 homolog A (S. cerevisiae)	-1.4	**-1.2**	-1.6	**-1.6**	-1.3		-1.2		-1.4	**-1.4**	-1.4	
RAD23B	RAD23 homolog B (S. cerevisiae)			-1.3	**-1.3**	-1.3		-1.3	**-1.3**			-1.2	
RAD51AP1	RAD51 associated protein 1			1.4				-2.8	**-2.8**	-1.4	**-1.4**	-1.3	**-1.3**
RAD51C	RAD51 homolog C (S. cerevisiae)			-1.6		-1.5				-1.9	**-1.9**	-1.9	**-1.9**
RAD51L1	RAD51-like 1 (S. cerevisiae)					-1.8	**-1.8**						
RAD51L3	RAD51-like 3 (S. cerevisiae)			-1.7		1.8		-1.2		-1.7	**-1.7**		
RAD52	RAD52 homolog (S. cerevisiae)			1.3		1.4		-1.6	**-1.6**	1.5	**1.5**	4.0	**4.0**
RAD54L	RAD54-like (S. cerevisiae)			-1.3		-1.2		-1.3		-1.5	**-1.5**	-1.9	**-1.9**
RAE1	RAE1 RNA export 1 homolog (S. pombe)					-1.3		-1.3	**-1.3**	-1.4	**-1.4**	-1.4	**-1.4**
RAF1	v-raf-1 murine leukemia viral oncogene homolog 1			1.4				-1.3	**-1.3**	-1.6			
RAFTLIN	raftlin, lipid raft linker 1			-1.2		-1.5		-1.5	**-1.5**	-1.5	**-1.5**		
RAG1	recombination activating gene 1	-3.6	**-3.6**	-3.2	**-3.2**	-4.2	**-4.2**	-32.2	**-32.2**	-1.3		14.5	**14.5**
RAG2	recombination activating gene 2			1.3				-7.4	**-7.4**			6.4	**6.4**
RAGE	renal tumor antigen	1.5		-1.4		1.3							
RAI17	zinc finger, MIZ-type containing 1	-1.3				-1.5				-1.3	**-1.3**	-1.6	**-1.6**
RALA	v-ral simian leukemia viral oncogene homolog A (ras related)	-1.3				-1.2		1.7	**1.7**	-1.3	**-1.3**	-2.1	**-2.1**
RALB	v-ral simian leukemia viral oncogene homolog B (ras related; GTP binding protein)	1.8						1.5	**1.5**	1.9	**1.9**	1.6	**1.6**
RALBP1	ralA binding protein 1	-1.2				-1.6		-1.8	**-1.8**	-1.4	**-1.4**	1.2	**1.2**
RALY	RNA binding protein, autoantigenic (hnRNP-associated with lethal yellow homolog (mouse))							-1.4	**-1.4**	1.3		1.2	**1.2**
RAMP3	receptor (G protein-coupled) activity modifying protein 3												
RAN	RAN, member RAS oncogene family							-1.2	**-1.2**	1.2	**-1.2**	-1.4	**-1.4**
RANBP1	RAN binding protein 1	-1.3		-1.8	**-1.8**	-1.5		-1.9	**-1.9**	-1.6		-2.8	**-2.8**
RANBP2	RAN binding protein 2							2.5	**2.5**	-1.4	**-1.4**	-1.9	**-1.9**
RANBP3	RAN binding protein 3												
RANBP5	RAN binding protein 5					-1.5	**-1.5**	-1.3	**-1.3**	-2.0	**-2.0**	-2.3	**-2.3**
RANBP9	RAN binding protein 9			-1.5	**-1.5**			-1.6	**-1.6**	-1.4	**-1.3**	2.1	**2.1**
RANGAP1	Ran GTPase activating protein 1												
RANGAP1	--	-1.2		-1.6				-1.4		-1.4	**-1.4**	-1.5	**-1.5**
RAP1A	RAP1A, member of RAS oncogene family			1.2				2.0	**2.0**			-2.2	**-2.2**
RAP1B	RAP1B, member of RAS oncogene family	1.4		1.5	**1.5**					1.3	**1.3**		
RAP1GDS1	RAP1, GTP-GDP dissociation stimulator 1							-1.5	**-1.5**			2.0	**2.0**
RAP2A	RAP2A, member of RAS oncogene family			3.9	**3.9**			-1.4	**-1.4**	1.6	**1.6**	4.0	**4.0**
RAPGEF2	Rap guanine nucleotide exchange factor (GEF) 2	1.7		1.7	**1.7**	1.4		1.8	**1.8**	2.0	**2.0**	4.2	**4.2**
RARRES3	retinoic acid receptor responder (tazarotene induced) 3			1.2						-1.7		1.6	**1.6**
RARS	arginyl-tRNA synthetase	-1.2	**-1.2**	-1.3	**-1.3**	-1.4						-1.9	**-1.9**
RASA1	RAS p21 protein activator (GTPase activating protein) 1	1.9	**1.9**	2.2	**2.2**	2.1	**2.0**	1.9	**1.9**	2.3	**2.3**	3.5	**3.5**
RASA3	RAS p21 protein activator 3			2.4	**2.4**			-2.6	**-2.6**	3.9	**3.9**	4.0	**4.0**
RASA4	RAS p21 protein activator 4	-1.3		-1.4				2.0	**2.0**	-1.3		-3.4	**-3.4**
RASGRP1	RAS guanyl releasing protein 1 (calcium and DAG-regulated)			1.4		-1.8		-111.1	**-111.1**	-7.2	**-7.2**		
RASGRP2	RAS guanyl releasing protein 2 (calcium and DAG-regulated)							-2.3	**-2.3**	2.1	**2.1**	4.4	**4.4**
RASSF1	Ras association (RalGDS/AF-6) domain family 1												
RASSF2	Ras association (RalGDS/AF-6) domain family 2	1.4		2.1	**2.1**	-1.2		-6.4	**-6.4**	-2.5	**-2.5**	3.5	**3.5**
RASSF7	Ras association (RalGDS/AF-6) domain family 7			-1.3									
RB1	retinoblastoma 1 (including osteosarcoma)			1.6		-2.0		-2.6	**-2.6**	1.5	**1.5**	3.1	**3.1**
RB1CC1	RB1-inducible coiled-coil 1	-1.2				-1.6		-1.4	**-1.4**	1.7	**1.7**	1.7	**1.7**
RBBP4	retinoblastoma binding protein 4			1.4		-1.4		-1.3	**-1.3**	-1.4	**-1.4**	-1.5	**-1.5**
RBBP5	retinoblastoma binding protein 5					-1.5		-1.3	**-1.3**			1.3	**1.3**
RBBP6	retinoblastoma binding protein 6	-1.2				-1.2		-1.8	**-1.8**	1.4		2.5	**2.5**
RBBP8	retinoblastoma binding protein 8	-1.3	**-1.3**			-1.3		-1.9	**-1.9**	-1.7	**-1.7**	-1.5	**-1.5**
RBL1	retinoblastoma-like 1 (p107)			1.4				-1.8	**-1.8**	1.3		-1.4	
RBL2	retinoblastoma-like 2 (p130)	1.3		2.5	**2.5**	1.7		1.5	**1.5**	1.5	**1.5**	-1.3	**-1.3**
RBM10	RNA binding motif protein 10					-1.3				-1.3	**-1.3**		
RBM13	RNA binding motif protein 13	-1.6	**-1.6**	-1.2		-1.3	**-1.3**	1.2		-1.8	**-1.8**	-2.1	**-2.1**
RBM14	RNA binding motif protein 14	-1.2	**-1.2**	-1.3	**-1.3**			-1.6				-1.4	

RBM16	RNA binding motif protein 16							1.2				1.2	**1.2**
RBM3	RNA binding motif (RNP1, RRM) protein 3												
RBM4	RNA binding motif protein 4					-1.3		1.7	**1.7**	1.3	**1.3**	-1.5	
RBM4B	RNA binding motif protein 4B							-1.7	**-1.7**	-1.2			
RBM5	RNA binding motif protein 5	1.4	**1.4**	1.5	**1.5**	1.3	**1.3**	1.6	**1.6**	-1.3		2.2	**2.2**
RBM8A	RNA binding motif protein 8A	1.2		-1.3		1.5		2.3	**2.3**	1.5	**1.5**	-3.3	**-3.3**
RBMS1	RNA binding motif, single stranded interacting protein 1	1.4		2.0		1.7		3.0	**3.0**	4.3	**4.3**	2.3	**2.3**
RBMS2	RNA binding motif, single stranded interacting protein 2	1.2				1.3						1.4	**1.4**
RBMX	RNA binding motif protein, X-linked					-1.2		1.4	**1.4**			-1.9	**-1.9**
RBMX2	RNA binding motif protein, X-linked 2	1.7		1.5	**1.5**								
RBPSUH	recombining binding protein suppressor of hairless (Drosophila)							1.9	**1.9**	1.4	**1.4**	-1.6	**1.2**
RCE1	RCE1 homolog, prenyl protein peptidase (S. cerevisiae)	-1.3											
RCHY1	ring finger and CHY zinc finger domain containing 1					-1.4		1.5	**1.5**			-1.6	**-1.6**
RCN1	reticulocalbin 1, EF-hand calcium binding domain	1.5	**1.5**	2.3	**2.3**	1.5		-1.5	**-1.5**	1.2	**1.2**	2.3	**2.3**
RCN2	reticulocalbin 2, EF-hand calcium binding domain	-1.3						1.4	**1.4**	2.0	**2.0**		
RCOR1	REST corepressor 1			1.4				-1.3	**-1.3**			1.3	**1.3**
RCP9	calcitonin gene-related peptide-receptor component protein	-1.2		-1.2		-1.2		-1.5	**-1.5**	-1.5	**-1.5**		
RDBP	RD RNA binding protein					-1.3		-1.2	**-1.2**			-1.6	**-1.6**
RDH11	retinol dehydrogenase 11 (all-trans/9-cis/11-cis)	-1.3		-1.6	**-1.6**	-1.4		-1.3	**-1.3**	-1.8	**-1.8**	-1.6	**-1.6**
RDX	radixin	1.6	**1.6**	2.2	**1.3**							2.5	**2.5**
REC14	WD repeat domain 61			1.2								-1.4	**-1.4**
RECK	reversion-inducing-cysteine-rich protein with kazal motifs	2.2		1.6	**1.6**	1.4				2.9	**2.9**	5.2	**5.2**
RECQL	RecQ protein-like (DNA helicase Q1-like)	1.6	**1.6**	1.8	**1.8**			-1.4	**-1.4**	1.2		1.4	**1.4**
RECQL5	RecQ protein-like 5	-1.4				2.1		2.3	**2.3**			-1.3	
REL	v-rel reticuloendotheliosis viral oncogene homolog (avian)	1.6		1.4		2.4	**2.4**	2.4	**2.4**	1.5	**1.5**	-2.1	**-2.1**
RELA	v-rel reticuloendotheliosis viral oncogene homolog A, nuclear factor of kappa light polypeptide gene enhancer in B-cells 3, p65 (avian)	-1.6	**-1.6**			-1.3				-1.2			
RENT1	UPF1 regulator of nonsense transcripts homolog (yeast)			-1.2	**-1.2**			1.3		-1.3		-1.7	
REV3L	REV3-like, catalytic subunit of DNA polymerase zeta (yeast)					1.2		-2.1	**-2.1**	1.5	**1.5**	2.2	**2.2**
RFC1	replication factor C (activator 1) 1, 145kDa	1.5	**1.5**			1.6		-1.2	**-1.2**	-1.4	**-1.4**	-2.1	**-2.1**
RFC2	replication factor C (activator 1) 2, 40kDa			-1.2		-1.3		-3.9	**-3.9**	-1.5	**-1.5**	-1.5	**-1.5**
RFC3	replication factor C (activator 1) 3, 38kDa	-1.3				-1.3		-2.1	**-2.1**	-1.5	**-1.5**	-1.5	**-1.5**
RFC4	replication factor C (activator 1) 4, 37kDa			-1.3		-1.7	**-1.7**			-1.6	**-1.6**	-4.2	**-4.2**
RFC5	replication factor C (activator 1) 5, 36.5kDa					-1.6		-1.3	**-1.3**	-1.7	**-1.7**	-2.9	**-2.9**
RFK	riboflavin kinase			-1.6	**-1.6**	-1.4		-1.9	**-1.9**	-2.1	**-2.1**	-1.5	**-1.3**
RFP	tripartite motif-containing 27					1.2				-1.5	**-1.5**	-1.8	**-1.8**
RFX5	regulatory factor X, 5 (influences HLA class II expression)							-1.6	**-1.6**	-1.4		-1.6	**-1.6**
RGL2	ral guanine nucleotide dissociation stimulator-like 2	1.6	**1.6**	1.9		1.6		1.3		1.2			
RGS1	regulator of G-protein signalling 1			2.0	**2.0**								
RGS10	regulator of G-protein signalling 10			1.3				19.3	**19.3**	-2.5	**-2.5**	-19.8	**-19.8**
RGS16	regulator of G-protein signalling 16	-2.4	**-2.4**										
RGS19	regulator of G-protein signalling 19	-1.3	**-1.3**	-1.4	**-1.4**	-1.6	**-1.6**	-1.8	**-1.8**	-1.3	**-1.3**	2.2	**2.2**
RGS19IP1	GIPC PDZ domain containing family, member 1							1.2					
RGS9	regulator of G-protein signalling 9					1.6							
RHEB	Ras homolog enriched in brain					-1.3		-1.7	**-1.6**	1.9	**1.9**	2.9	**2.9**
RHOA	ras homolog gene family, member A	1.2	**1.2**					-1.4	**-1.4**	1.4	**1.4**	1.4	**1.4**
RHOC	ras homolog gene family, member C			1.3	**1.3**			3.0	**3.0**	3.2	**3.2**	2.3	**2.3**
RHOG	ras homolog gene family, member G (rho G)							1.9	**1.9**			-1.6	**-1.6**
RHOH	ras homolog gene family, member H			1.3	**1.3**	-1.4		-15.8	**-15.8**	1.3	**1.3**	38.1	**38.1**
RHOQ	ras homolog gene family, member Q					-1.2		1.8	**1.8**	1.3	**1.3**	-1.7	**-1.7**
RICS	Rho GTPase-activating protein	1.6						-3.3	**-3.3**				
RIF1	RAP1 interacting factor homolog (yeast)					-1.3		2.0	**2.0**	-1.3	**-1.3**	-6.8	**-6.8**
RIMS2	regulating synaptic membrane exocytosis 2					-1.2							
RIMS3	regulating synaptic membrane exocytosis 3	1.4		1.4				-6.8	**-6.8**			12.1	**12.1**
RING1	ring finger protein 1												
RIOK3	RIO kinase 3 (yeast)	1.2						1.7	**1.7**	1.3		1.5	**1.5**
RIPK1	receptor (TNFRSF)-interacting serine-threonine kinase 1			1.2				1.5	**1.5**	1.4			
RIT1	Ras-like without CAAX 1			1.4	**1.4**			1.6	**-1.4**	1.8	**1.8**	2.1	**2.1**
RNASE3	ribonuclease, RNase A family, 3 (eosinophil cationic protein)												

RNASEH1	ribonuclease H1	-1.2						-1.4		-1.3	**-1.3**	-1.9	**-1.9**
RNF10	ring finger protein 10			1.2									
RNF103	ring finger protein 103	1.3	**1.3**	1.7	**1.7**			-1.2		1.5	**1.5**	1.7	**1.7**
RNF11	ring finger protein 11	-1.3		1.3	**1.3**	-1.2				1.5	**1.5**	1.5	**1.5**
RNF113A	ring finger protein 113A												
RNF13	ring finger protein 13					-1.5		1.4	**1.3**	-1.5	**-1.5**		
RNF139	ring finger protein 139							1.4	**1.4**				
RNF14	ring finger protein 14							1.3		1.4	**1.4**	2.3	**2.3**
RNF144	ring finger protein 144	1.5		1.2				3.5	**3.5**			-4.3	**-4.3**
RNF2	ring finger protein 2	-1.2		-1.3		-1.3		-1.5					
RNF4	ring finger protein 4					-1.4						-1.3	**-1.3**
RNF40	ring finger protein 40												
RNF5	ring finger protein 5			1.3		-1.3						-1.5	**-1.5**
RNF6	ring finger protein (C3H2C3 type) 6			-1.3		-1.2		-2.2	**-2.2**	1.2	**1.2**	2.2	**2.2**
RNGTT	RNA guanylyltransferase and 5'-phosphatase							-1.6	**-1.6**	-1.4		1.3	
RNH	ribonuclease/angiogenin inhibitor 1			-1.4		-1.3	**-1.3**	2.4	**2.4**	-1.5	**-1.5**	-4.2	**-4.2**
RNMT	RNA (guanine-7-) methyltransferase					-1.5		1.3	**1.3**	-2.1		-1.5	**-1.5**
RNPC1	RNA binding motif protein 38	1.5						-1.9	**-1.9**	1.4	**1.4**	1.9	**1.9**
RNPC2	RNA binding motif protein 39					1.2		1.8	**1.8**	2.3	**2.3**	1.3	**1.3**
RNPEP	arginyl aminopeptidase (aminopeptidase B)	1.3		1.3	**1.3**								
RNPS1	RNA binding protein S1, serine-rich domain			-1.4				-5.0	**-5.0**	-1.3	**-1.2**	2.5	**2.5**
RNUT1	snurportin 1					-1.4		1.3	**1.3**			-1.5	**-1.5**
ROCK1	Rho-associated, coiled-coil containing protein kinase 1			1.4						1.2		1.8	**1.8**
ROCK2	Rho-associated, coiled-coil containing protein kinase 2			1.2				-1.3		1.3	**1.3**		
ROD1	ROD1 regulator of differentiation 1 (S. pombe)			1.3				-1.5		1.2		1.9	**1.9**
RORB	RAR-related orphan receptor B	-1.2											
RP2	retinitis pigmentosa 2 (X-linked recessive)									1.2	**1.2**		
RPA1	replication protein A1, 70kDa			1.6	**1.6**	-1.2		-2.8	**-2.8**	-1.6	**-1.6**	1.5	**1.5**
RPA2	replication protein A2, 32kDa									-1.4	**-1.4**	-1.5	**-1.5**
RPA3	replication protein A3, 14kDa	1.2						-2.0	**-2.0**	-1.4		-1.3	
RPE	ribulose-5-phosphate-3-epimerase	-1.2						-2.1	**-2.1**	-1.5	**-1.5**	1.4	**1.4**
RPGR	retinitis pigmentosa GTPase regulator	-1.6	**-1.6**	-2.1	**-2.1**	-1.3		6.5	**6.5**			1.2	
RPH3A	rabphilin 3A homolog (mouse)												
RPIA	ribose 5-phosphate isomerase A (ribose 5-phosphate epimerase)	-1.3		-1.3	**-1.3**	-1.4				-1.7	**-1.7**	-2.7	**-2.7**
RPL10	ribosomal protein L10									1.7			
RPL10A	ribosomal protein L10a												
RPL11	ribosomal protein L11												
RPL12	ribosomal protein L12							1.5	**1.5**				
RPL13	ribosomal protein L13							1.4	**1.4**			-3.0	**-3.0**
RPL13A	ribosomal protein L13a							1.4	**1.4**	-1.3		-1.2	**-1.2**
RPL14	ribosomal protein L14					-1.2							
RPL15	ribosomal protein L15							1.5	**1.5**	2.1	**2.1**	-1.4	**-1.4**
RPL17	ribosomal protein L17					-1.3		2.1	**2.1**	-1.8	**-1.8**	-2.3	**-2.3**
RPL18	ribosomal protein L18							1.7		2.4	**2.4**		
RPL18A	ribosomal protein L18a							1.4	**1.4**	1.3	**1.3**		
RPL19	ribosomal protein L19									-1.4			
RPL21	ribosomal protein L21												
RPL22	ribosomal protein L22							1.2	**1.2**				
RPL23	ribosomal protein L23					-1.3		1.3	**1.3**	-1.3	**-1.3**	-1.3	
RPL23A	ribosomal protein L23a												
RPL24	ribosomal protein L24												
RPL27	ribosomal protein L27												
RPL27A	ribosomal protein L27a			1.2									
RPL28	ribosomal protein L28											-1.4	
RPL29	ribosomal protein L29							2.0	**2.0**	1.4	**1.4**	-1.5	**-1.5**
RPL3	ribosomal protein L3							1.3	**1.3**				
RPL30	ribosomal protein L30												
RPL31	ribosomal protein L31							2.9	**2.9**	1.3	**1.3**	-3.4	**-3.4**

RPL32	ribosomal protein L32												
RPL34	ribosomal protein L34												
RPL35	ribosomal protein L35	-1.2		-1.3	**-1.3**	-1.3		2.2	**2.2**	2.4	**2.4**	-1.8	**-1.8**
RPL36A	ribosomal protein L36a												
RPL36AL	ribosomal protein L36a-like									1.4	**1.4**		
RPL37	ribosomal protein L37							1.6	**1.6**	-1.5	**-1.5**	-1.8	**-1.8**
RPL37A	ribosomal protein L37a									-1.3		-2.2	**-2.2**
RPL38	ribosomal protein L38							2.0	**2.0**			-1.5	**-1.5**
RPL4	ribosomal protein L4	-1.2				-1.4		1.3	**1.3**				
RPL41	ribosomal protein L41												
RPL5	ribosomal protein L5			-1.5		2.7		1.3	**1.3**	1.7	**1.2**	1.3	
RPL6	ribosomal protein L6												
RPL7	ribosomal protein L7							1.3		1.3			
RPL8	ribosomal protein L8												
RPL9	ribosomal protein L9												
RPLP0	ribosomal protein, large, P0												
RPLP1	ribosomal protein, large, P1	-1.3				1.2							
RPLP2	ribosomal protein, large, P2			1.2				1.4	**1.4**			-1.7	**-1.7**
RPN1	ribophorin I							1.5	**1.5**			-2.3	**-2.3**
RPN2	ribophorin II							1.7	**1.7**	-1.5	**-1.5**	-2.6	**-2.6**
RPP14	ribonuclease P 14kDa subunit							1.9	**1.9**	-1.7	**-1.7**	-2.7	**-2.7**
RPP30	ribonuclease P/MRP 30kDa subunit					-1.6				-1.6	**-1.6**	-1.6	**-1.6**
RPP38	ribonuclease P/MRP 38kDa subunit	-1.2	**-1.2**			-1.4		1.7	**1.7**	-1.5	**-1.5**	-1.8	**-1.8**
RPP40	ribonuclease P 40kDa subunit	-1.9	**-1.9**	-1.8		-1.4				-1.9	**-1.9**	-2.3	**-2.3**
RPS10	ribosomal protein S10							1.3	**1.3**	1.4	**1.4**	1.3	
RPS11	ribosomal protein S11												
RPS12	ribosomal protein S12												
RPS13	ribosomal protein S13												
RPS14	ribosomal protein S14							1.5	**1.5**	1.6	**1.6**		
RPS15	ribosomal protein S15	-1.2											
RPS15A	ribosomal protein S15a					-1.2		1.5	**1.5**			1.2	**1.2**
RPS16	ribosomal protein S16												
RPS17	ribosomal protein S17							1.5	**1.5**	1.5	**1.5**		
RPS18	ribosomal protein S18							2.2	**2.2**				
RPS19	ribosomal protein S19							1.3	**1.3**	-1.6	**-1.6**		
RPS2	ribosomal protein S2	-1.2						2.6	**2.6**			-1.9	**-1.9**
RPS20	ribosomal protein S20												
RPS21	ribosomal protein S21	-1.3		1.4				3.1	**3.1**	1.2	**1.2**	-3.3	**-3.3**
RPS23	ribosomal protein S23							2.0	**2.0**	-1.3		-2.1	**-2.1**
RPS24	ribosomal protein S24							2.2	**2.2**			-3.2	**-3.2**
RPS25	ribosomal protein S25									1.3	**1.3**		
RPS26	ribosomal protein S26												
RPS27	ribosomal protein S27 (metallopanstimulin 1)							1.3		-1.3		-1.7	**-1.7**
RPS27A	ribosomal protein S27a											-1.2	
RPS28	ribosomal protein S28							1.2	**1.2**				
RPS3	ribosomal protein S3							1.2	**1.2**				
RPS3A	ribosomal protein S3A												
RPS4X	ribosomal protein S4, X-linked									1.2		1.5	**1.5**
RPS5	ribosomal protein S5												
RPS6	ribosomal protein S6							-1.7	**-1.7**	-1.9	**-1.9**	-1.3	**1.3**
RPS6KA1	ribosomal protein S6 kinase, 90kDa, polypeptide 1							-2.9	**-2.9**	-1.3	**-1.3**	3.6	**3.6**
RPS6KA2	ribosomal protein S6 kinase, 90kDa, polypeptide 2			2.9	**2.9**			7.9	**7.9**	6.0	**6.0**	14.2	**14.2**
RPS6KA3	ribosomal protein S6 kinase, 90kDa, polypeptide 3	1.2		1.6						-1.5	**-1.5**	-1.7	**-1.7**
RPS6KA4	ribosomal protein S6 kinase, 90kDa, polypeptide 4			-1.9	**-1.9**					-1.3	**-1.3**	-1.5	**-1.5**
RPS6KA5	ribosomal protein S6 kinase, 90kDa, polypeptide 5	-1.3		1.5		1.2							
RPS6KB1	ribosomal protein S6 kinase, 70kDa, polypeptide 1	-1.3				-1.3		1.7	**1.7**			-1.7	**-1.7**
RPS6KB2	ribosomal protein S6 kinase, 70kDa, polypeptide 2											-1.3	
RPS7	ribosomal protein S7					-1.2							

RPS8	ribosomal protein S8							1.3	**1.3**	1.2			
RPS9	ribosomal protein S9							-1.4		2.5	**2.5**	2.6	**2.6**
RPSA	ribosomal protein SA	-1.3											
RPUSD2	RNA pseudouridylate synthase domain containing 2												
RQCD1	RCD1 required for cell differentiation1 homolog (S. pombe)			1.2		-1.4		-2.3	**-2.3**	-1.7	**-1.7**	-1.4	**-1.4**
RRAGA	Ras-related GTP binding A	1.4	**1.4**	1.5	**1.5**			-2.6	**-2.6**	1.3	**1.3**	3.2	**3.2**
RRAGD	Ras-related GTP binding D			-1.2		-1.3		3.0	**3.0**	-2.4	**-2.4**	-8.9	**-8.9**
RRAS	related RAS viral (r-ras) oncogene homolog	1.3				1.6		1.7	**1.7**	2.6	**2.6**	2.4	**2.4**
RRM1	ribonucleotide reductase M1 polypeptide					-1.4		-1.6	**-1.6**	-1.4	**-1.4**	-2.1	**-2.1**
RRM2	ribonucleotide reductase M2 polypeptide			1.4				-2.3	**-2.3**			-1.8	**-1.8**
RRP9	RRP9, small subunit (SSU) processome component, homolog (yeast)	-1.4		-1.4	**-1.4**							-7.8	**-7.8**
RRS1	RRS1 ribosome biogenesis regulator homolog (S. cerevisiae)	-1.9		-2.7	**-2.7**	-1.9		-1.4	**-1.4**			-1.6	**-1.6**
RSAD2	radical S-adenosyl methionine domain containing 2					2.2		1.9	**1.9**	2.2		-2.7	**-2.7**
RSL1D1	ribosomal L1 domain containing 1	-1.4		-1.6	**-1.6**	-1.4		-3.8	**-3.8**	-2.1	**-2.1**	2.1	
RSN	CAP-GLY domain containing linker protein 1							-1.2		1.9		7.4	**7.4**
RSU1	Ras suppressor protein 1	-1.4				-1.2						-1.8	**-1.8**
RTN2	reticulon 2			1.4				1.6	**1.6**	1.4			
RTN4	reticulon 4	-1.2				-1.3				2.3	**2.3**	2.4	**2.4**
RUNX1	runt-related transcription factor 1 (acute myeloid leukemia 1; aml1 oncogene)												
RUNX1	--	-1.3	**-1.3**	1.5		-1.4		-1.5	**-1.5**	-2.5	**-2.5**	1.4	**-1.4**
RUNX3	runt-related transcription factor 3					-1.3		9.2	**9.2**	-1.5		-27.7	**-27.7**
RUSC1	RUN and SH3 domain containing 1											-1.3	
RUVBL1	RuvB-like 1 (E. coli)	-1.6		-1.9	**-1.9**	-1.4		-1.6	**-1.6**			-1.7	**-1.7**
RUVBL2	RuvB-like 2 (E. coli)			-1.3	**-1.3**			1.2	**1.2**	-1.4	**-1.4**	-2.0	**-2.0**
RW1	transmembrane protein 131			1.3				1.4	**1.4**	1.3	**1.3**		
RXRA	retinoid X receptor, alpha					1.3		2.4	**2.4**	1.6		-3.3	**-3.3**
RXRB	retinoid X receptor, beta			1.2									
RXRG	retinoid X receptor, gamma			1.2	**1.2**								
RYBP	RING1 and YY1 binding protein					-1.3		1.4	**1.4**	1.3	**1.3**	2.9	**2.9**
RYK	RYK receptor-like tyrosine kinase	1.3		1.5		1.6		11.1	**11.1**			-22.2	**-22.2**
S100A10	S100 calcium binding protein A10	1.4		1.7	**1.7**	1.2		-26.4	**-26.4**			40.6	**40.6**
S100A11	S100 calcium binding protein A11					-1.8	**-1.8**	1.5	**1.5**	1.4			
S100A4	S100 calcium binding protein A4							-7.7	**-7.7**	-4.0	**-4.0**	5.4	**5.4**
SAC3D1	SAC3 domain containing 1	-1.6	**-1.6**	-1.6	**-1.6**	-1.5	**-1.5**						
SACM1L	SAC1 suppressor of actin mutations 1-like (yeast)	-1.4		-1.5	**-1.5**	-1.4		1.8	**1.8**	1.2		-1.6	**-1.6**
SACS	spastic ataxia of Charlevoix-Saguenay (sacsin)	-1.5		-2.1	**-2.1**	-2.0	**-2.0**	-1.7	**-1.7**	-1.4	**-1.4**	-1.4	**-1.4**
SAFB	scaffold attachment factor B					-1.3						-1.7	**-1.3**
SAFB2	scaffold attachment factor B2									1.4	**1.4**		
SALL2	sal-like 2 (Drosophila)												
SAP18	Sin3A-associated protein, 18kDa	1.3						-2.2	**-2.2**	-1.3		1.7	**1.7**
SAP30	Sin3A-associated protein, 30kDa	2.2	**2.2**	1.8	**1.8**	1.7		1.7	**1.7**	2.3	**2.3**	3.8	**3.8**
SARA1	SAR1 gene homolog A (S. cerevisiae)	1.2		1.2				1.6	**1.6**			-1.7	**-1.7**
SARS	seryl-tRNA synthetase	-1.4	**-1.4**			-1.3		-2.7	**-2.7**	-1.4	**-1.4**	1.8	**1.8**
SART1	squamous cell carcinoma antigen recognized by T cells					-1.2							
SART3	squamous cell carcinoma antigen recognized by T cells 3			-1.3		-1.2		1.3		-1.4	**-1.4**	-1.9	**-1.9**
SAS	tetraspanin 31	1.8	**1.8**	1.7	**1.7**			1.3				2.0	**2.0**
SAT	spermidine/spermine N1-acetyltransferase 1							5.5	**5.5**	3.9	**3.9**	1.3	**1.3**
SATB1	special AT-rich sequence binding protein 1 (binds to nuclear matrix/scaffold-associating DNA's)	-1.9	**-1.9**	-1.8	**-1.8**	-2.3	**-2.3**	6.4	**6.4**	-1.8	**-1.8**	-3.7	**-3.7**
SATB2	SATB family member 2	-1.5	**-1.5**	1.7				-1.2				2.8	**2.8**
SBF1	SET binding factor 1					1.3		-1.9	**-1.9**	3.1	**3.1**	6.9	**6.9**
SC4MOL	sterol-C4-methyl oxidase-like	-1.6		-1.8	**-1.8**	-1.6	**-1.6**	-3.2	**-3.2**	1.6	**1.6**	4.6	**4.6**
SC5DL	sterol-C5-desaturase (ERG3 delta-5-desaturase homolog, fungal)-like			-1.3				-1.7	**-1.7**			2.6	**2.6**
SCAMP1	secretory carrier membrane protein 1	-1.4		-1.3		-1.4		2.0	**1.7**	-1.3		-1.7	**-1.7**
SCAMP3	secretory carrier membrane protein 3	-1.4		-1.3	**-1.3**	-1.4				-1.3		-1.4	**-1.4**
SCAMP5	secretory carrier membrane protein 5							4.4	**4.4**			-2.6	**-2.6**
SCAP	SREBF chaperone	-1.3		-1.5	**-1.5**			1.2	**1.2**	-1.2		-1.4	**-1.4**
SCAP1	src kinase associated phosphoprotein 1					-2.5	**-2.5**						
SCARB1	scavenger receptor class B, member 1	-1.7	**-1.7**	-2.2	**-2.2**	-1.2		-1.4	**-1.4**	-4.9	**-4.9**	-33.3	**-33.3**

SCC-112	SCC-112 protein							-1.4	**-1.4**	1.3	**1.3**	1.5	**1.5**
SCD	stearoyl-CoA desaturase (delta-9-desaturase)	-2.0				-1.5				-3.7	**-3.7**	-3.6	**-3.6**
SCFD1	sec1 family domain containing 1					-1.2		1.3		2.2	**2.2**	1.9	**1.9**
SCHIP1	schwannomin interacting protein 1					-1.2		-5.1	**-5.1**	1.4	**1.4**	1.9	**1.9**
SCP2	sterol carrier protein 2	1.3		1.3				-12.0	**-12.0**	1.6	**1.6**	175.1	**175.1**
SCRIB	scribbled homolog (Drosophila)			1.2		1.7	**1.7**	1.5	**1.5**	1.2			
SCYE1	small inducible cytokine subfamily E, member 1 (endothelial monocyte-activating)	-1.7	**-1.7**	-1.6		-1.9	**-1.9**	1.6	**1.6**	-1.4	**-1.4**	-2.0	**-2.0**
SDC1	syndecan 1											-14.8	**-14.8**
SDC3	syndecan 3 (N-syndecan)					1.4							
SDCBP	syndecan binding protein (syntenin)	1.8	**1.8**	2.0	**2.0**			-1.5	**-1.5**	1.5	**1.5**	2.5	**2.5**
SDF2	stromal cell-derived factor 2											1.3	
SDFR1	neuroplastin			-1.3				1.5	**1.5**			-1.5	**-1.5**
SDHA	succinate dehydrogenase complex, subunit A, flavoprotein (Fp)					-1.2		-1.5	**-1.5**	2.0	**2.0**	1.9	**1.9**
SDHB	succinate dehydrogenase complex, subunit B, iron sulfur (Ip)	-1.2				-1.6				-1.4	**-1.4**	-1.9	**-1.9**
SDHC	succinate dehydrogenase complex, subunit C, integral membrane protein, 15kDa			1.4				-1.3	**-1.3**			1.2	**1.2**
SDHD	succinate dehydrogenase complex, subunit D, integral membrane protein	-1.3										-1.2	
SEC13L1	SEC13 homolog (S. cerevisiae)			-1.2		-1.2		-1.2	**-1.2**				
SEC14L1	SEC14-like 1 (S. cerevisiae)			1.4		-1.2		1.6		24.5	**24.5**	-1.9	**-1.9**
SEC22L1	SEC22 vesicle trafficking protein homolog B (S. cerevisiae)	1.4		1.4	**1.4**			-1.9	**-1.9**	1.3		4.1	**4.1**
SEC23A	Sec23 homolog A (S. cerevisiae)									1.9	**1.9**	1.8	**1.8**
SEC23B	Sec23 homolog B (S. cerevisiae)					-1.4				-1.5	**-1.5**		
SEC23IP	SEC23 interacting protein	-1.2				-1.7		1.4	**1.4**	-1.5		-1.6	**-1.6**
SEC24A	SEC24 related gene family, member A (S. cerevisiae)							1.3		-1.4		1.5	**1.5**
SEC24B	SEC24 related gene family, member B (S. cerevisiae)					-1.3				1.2		1.3	**1.3**
SEC24C	SEC24 related gene family, member C (S. cerevisiae)					1.2		1.4	**1.4**			-1.3	**-1.3**
SEC24D	SEC24 related gene family, member D (S. cerevisiae)					1.3		1.5	**1.5**				
SEC31L1	SEC31 homolog A (S. cerevisiae)	1.3		1.3	**1.3**			1.2		1.5		1.6	**1.6**
SEC61B	Sec61 beta subunit			1.2				1.5		-1.6			
SEC61G	Sec61 gamma subunit			-1.2		-1.3		2.2	**2.2**			-2.6	**-2.6**
SEC63	SEC63 homolog (S. cerevisiae)					-1.2	**-1.2**	1.5	**1.5**	-1.3	**-1.3**	-2.5	**-2.5**
SEC6L1	exocyst complex component 3			1.2						1.8	**1.8**		
SEC8L1	exocyst complex component 4					1.6				1.8	**1.8**	1.8	**1.8**
SEDLP	spondyloepiphyseal dysplasia, late, pseudogene			1.6									
SELL	selectin L (lymphocyte adhesion molecule 1)					-1.2		-7.7	**-7.7**	-3.1	**-3.1**	1.4	**1.4**
SELPLG	selectin P ligand	1.3		1.4	**1.4**	1.2							
SEMA3F	sema domain, immunoglobulin domain (Ig), short basic domain, secreted, (semaphorin) 3F							-1.2					
SEMA4D	sema domain, immunoglobulin domain (Ig), transmembrane domain (TM) and short cytoplasmic domain, (semaphorin) 4D	1.3				1.6	**1.6**	-1.5	**-1.5**			2.1	**2.1**
SENP3	SUMO1/sentrin/SMT3 specific peptidase 3	-1.3	**-1.3**	-1.3		-1.2	**-1.2**	-2.9	**-2.9**	-1.7	**-1.7**	1.4	
SENP6	SUMO1/sentrin specific peptidase 6					-1.8		2.0	**2.0**	1.6	**1.5**	-1.3	**1.2**
SEP15	15 kDa selenoprotein					-1.2				1.2			
SEPHS1	selenophosphate synthetase 1			-1.4	**-1.4**	-1.5		-1.6	**-1.6**	-2.1	**-2.1**	-1.7	**-1.7**
SEPHS2	selenophosphate synthetase 2			1.4									
SEPT2	septin 2							-3.6	**-3.6**	-1.5	**-1.5**	2.4	**2.4**
SEPT6	septin 6	-1.3				-1.6	**-1.6**	-1.4	**-1.4**	-2.0	**-2.0**	-2.7	**-2.7**
SEPT7	septin 7							2.6	**2.6**	1.3	**1.3**	-2.3	**-2.3**
SEPT8	septin 8							1.5	**1.5**	-1.4	**-1.4**	4.5	**4.5**
SEPT9	septin 9							-2.8	**-2.8**			2.6	**2.6**
SEPW1	selenoprotein W, 1					1.3		2.4	**2.4**	1.3	**1.3**	-1.5	**-1.5**
SERP1	stress-associated endoplasmic reticulum protein 1							1.5	**1.5**	-1.7	**-1.7**	-1.6	**-1.6**
SERPINB1	serpin peptidase inhibitor, clade B (ovalbumin), member 1							40.4	**40.4**	3.1	**3.1**	-7.4	**-7.4**
SERPINB8	serpin peptidase inhibitor, clade B (ovalbumin), member 8					-1.3						1.4	**1.4**
SERPINE2	serpin peptidase inhibitor, clade E (nexin, plasminogen activator inhibitor type 1), member 2	1.2				1.9						-1.3	**-1.3**
SERPINF1	serpin peptidase inhibitor, clade F (alpha-2 antiplasmin, pigment epithelium derived factor), member 1	1.3						-4.7	**-4.7**	1.8		3.3	**3.3**
SERPINH1	serpin peptidase inhibitor, clade H (heat shock protein 47), member 1, (collagen binding protein 1)			1.4	**1.4**	1.7		18.1	**18.1**			-17.3	**-17.3**
SERTAD2	SERTA domain containing 2	1.2		1.8	**1.8**	1.8		1.5	**1.5**			-1.8	**-1.8**
SET	SET translocation (myeloid leukemia-associated)					-1.5		-1.3	**-1.3**	-1.9	**-1.9**	-1.5	**-1.5**
SETD1A	SET domain containing 1A			-1.2									
SETDB1	SET domain, bifurcated 1											-1.2	

SF1	splicing factor 1	1.4		-1.4	**-1.4**			-1.6	**-1.6**	-1.4	**-1.4**		
SF3A1	splicing factor 3a, subunit 1, 120kDa					-1.3		-1.4	**-1.4**	-1.5		-1.2	
SF3A2	splicing factor 3a, subunit 2, 66kDa			-1.5	**-1.5**	-1.2		-1.5	**-1.5**	-1.6	**-1.6**	1.2	
SF3A3	splicing factor 3a, subunit 3, 60kDa	-1.3		-1.6	**-1.6**	-1.6	**-1.6**			-1.4	**-1.4**	-1.9	**-1.9**
SF3B1	splicing factor 3b, subunit 1, 155kDa	1.3	**1.3**					1.8	**1.8**			1.3	
SF3B2	splicing factor 3b, subunit 2, 145kDa					-1.3				-1.4		-1.5	**-1.5**
SF3B3	splicing factor 3b, subunit 3, 130kDa					-1.4		2.2	**-1.4**	-1.7	**-1.7**	-2.7	**-2.7**
SF3B4	splicing factor 3b, subunit 4, 49kDa			1.2	**1.2**	1.2	**1.2**						
SFI1	--	-2.0				1.8		-1.4	**-1.4**				
SFI1	Sfi1 homolog, spindle assembly associated (yeast)												
SFN	stratifin			1.2		1.4		-1.7	**-1.7**	-1.8	**-1.8**		
SFPQ	splicing factor proline/glutamine-rich (polypyrimidine tract binding protein associated)	1.4		1.4		1.2		-1.5	**1.4**	1.2	**1.2**	-1.3	
SFRS1	splicing factor, arginine/serine-rich 1 (splicing factor 2, alternate splicing factor)			-1.2	**-1.2**	-1.4		-1.6	**-1.6**	-1.7	**-1.7**	-2.4	**-2.4**
SFRS10	splicing factor, arginine/serine-rich 10 (transformer 2 homolog, Drosophila)					-1.4		-1.7	**-1.7**	-1.2	**-1.2**	-1.3	**-1.3**
SFRS11	splicing factor, arginine/serine-rich 11					-1.2		2.7	**2.7**	4.2	**4.2**	-4.1	**-4.1**
SFRS12	splicing factor, arginine/serine-rich 12							4.5	**4.5**	-1.7	**-1.7**	-3.5	**-3.5**
SFRS2	splicing factor, arginine/serine-rich 2	-1.2		-1.6	**-1.2**	-1.2		-1.3	**-1.3**	-1.4	**-1.4**	-1.5	**-1.5**
SFRS2B	splicing factor, arginine/serine-rich 2B			1.3				-1.3	**-1.3**	-1.3	**-1.3**	1.5	**1.3**
SFRS2IP	splicing factor, arginine/serine-rich 2, interacting protein	1.3		1.6									
SFRS3	splicing factor, arginine/serine-rich 3			1.2		-1.3		-1.4	**-1.4**	-1.4	**1.3**	1.5	**1.5**
SFRS4	splicing factor, arginine/serine-rich 4							-2.1	**-1.2**	1.3		1.3	
SFRS5	splicing factor, arginine/serine-rich 5	1.2											
SFRS6	splicing factor, arginine/serine-rich 6			-1.7	**-1.7**			-3.6	**-3.6**			3.4	**3.4**
SFRS7	splicing factor, arginine/serine-rich 7, 35kDa			-1.6	**-1.6**			-1.8	**-1.8**	-1.5	**-1.5**	-1.9	**-1.9**
SFRS8	splicing factor, arginine/serine-rich 8 (suppressor-of-white-apricot homolog, Drosophila)	1.3				1.3				-1.3	**-1.3**		
SFRS9	splicing factor, arginine/serine-rich 9					-1.2		-2.7	**-2.7**	1.2	**1.2**	2.0	**2.0**
SGNE1	secretogranin V (7B2 protein)	1.3											
SGPL1	sphingosine-1-phosphate lyase 1	-1.6								-1.3	**-1.3**	1.3	
SGSH	N-sulfoglucosamine sulfohydrolase (sulfamidase)	1.8	**1.8**									2.1	**2.1**
SGTA	small glutamine-rich tetratricopeptide repeat (TPR)-containing, alpha												
SH2B	SH2B adaptor protein 1											1.8	**1.8**
SH2D1A	SH2 domain protein 1A, Duncan's disease (lymphoproliferative syndrome)	-1.5		-1.3	**-1.3**	-1.5							
SH3BP1	SH3-domain binding protein 1			-1.2									
SH3BP5	SH3-domain binding protein 5 (BTK-associated)	-1.8	**-1.8**	-2.5	**-2.5**					2.6	**2.6**		
SH3GL1	SH3-domain GRB2-like 1			-1.4				1.4				-1.4	
SH3GLB1	SH3-domain GRB2-like endophilin B1							1.5	**1.5**	2.2	**2.2**	2.5	**2.5**
SHC1	SHC (Src homology 2 domain containing) transforming protein 1							-1.7	**-1.7**			1.3	
SHFM1	split hand/foot malformation (ectrodactyly) type 1	1.2						-1.8	**-1.8**			1.2	
SHMT1	serine hydroxymethyltransferase 1 (soluble)					-1.2		-1.5	**-1.5**	-1.8	**-1.8**	1.4	**1.4**
SHMT2	serine hydroxymethyltransferase 2 (mitochondrial)							-2.3	**-2.3**	-1.8	**-1.8**	1.4	
SHOC2	soc-2 suppressor of clear homolog (C. elegans)												
SIAH1	seven in absentia homolog 1 (Drosophila)			-1.3		-1.3		-1.5	**-1.5**			2.7	**1.5**
SIAH2	seven in absentia homolog 2 (Drosophila)	-1.4	**-1.4**	-1.4	**-1.4**	-1.3							
SIAHBP1	fuse-binding protein-interacting repressor					-1.3				-1.2	**-1.2**	-1.4	**-1.4**
SIM2	single-minded homolog 2 (Drosophila)	1.3		1.3		1.3		2.4	**2.4**			-3.1	**-3.1**
SIP1	survival of motor neuron protein interacting protein 1					-1.9				-1.2		-1.5	**-1.5**
SIPA1	signal-induced proliferation-associated gene 1	1.2						-4.6	**-4.6**	-1.3		4.9	**4.9**
SIT	signaling threshold regulating transmembrane adaptor 1					-1.2		-18.5	**-18.5**				
SIVA	SIVA1, apoptosis-inducing factor	-1.4		-1.3		-1.3		-1.6	**-1.6**	-1.7	**-1.7**	-2.9	**-2.9**
SKI	v-ski sarcoma viral oncogene homolog (avian)					1.5		7.9	**7.9**			-5.0	**-5.0**
SKIIP	SNW domain containing 1											1.3	**1.3**
SKIP (C62)	skeletal muscle and kidney enriched inositol phosphatase					1.3		-1.7	**-1.7**	1.3		2.3	**2.3**
SKIV2L	superkiller viralicidic activity 2-like (S. cerevisiae)												
SKP1A	S-phase kinase-associated protein 1A (p19A)	-1.3				-1.4						-1.5	**-1.5**
SLA	Src-like-adaptor	2.8	**2.8**	3.1	**3.1**	3.1	**3.1**	2.1	**2.1**	4.4	**4.4**	10.8	**10.8**
SLBP	stem-loop (histone) binding protein					-1.7		-1.8	**-1.8**	-1.3	**-1.3**	-1.8	**-1.8**
SLC11A2	solute carrier family 11 (proton-coupled divalent metal ion transporters), member 2	1.4		1.3				2.1	**2.1**	-2.1	**-2.1**	-2.1	**-2.1**
SLC12A2	solute carrier family 12 (sodium/potassium/chloride transporters), member 2	1.3						-1.9	**-1.9**	-1.8		1.9	**1.9**

SLC12A4	solute carrier family 12 (potassium/chloride transporters), member 4												
SLC13A2	solute carrier family 13 (sodium-dependent dicarboxylate transporter), member 2					1.4							
SLC16A1	solute carrier family 16, member 1 (monocarboxylic acid transporter 1)	-1.3		-1.3		-1.5		-1.2	**-1.2**	-2.8	**-2.8**	-3.9	**-3.9**
SLC16A3	solute carrier family 16, member 3 (monocarboxylic acid transporter 4)							9.6	**9.6**			-28.1	**-28.1**
SLC16A5	solute carrier family 16, member 5 (monocarboxylic acid transporter 6)			1.4		1.3		2.4	**2.4**				
SLC16A6	solute carrier family 16, member 6 (monocarboxylic acid transporter 7)	-1.3		-1.6				1.3		-1.6	**-1.6**	-1.4	**-1.4**
SLC18A2	solute carrier family 18 (vesicular monoamine), member 2	4.1	**4.1**			4.2	**4.2**	4.3	**4.3**	1.7	**1.6**	-6.1	**-6.1**
SLC19A1	solute carrier family 19 (folate transporter), member 1	-1.6	**-1.6**	-2.1	**-2.1**	-1.7	**-1.7**			-1.7	**-1.7**	-4.6	**-4.6**
SLC1A4	solute carrier family 1 (glutamate/neutral amino acid transporter), member 4	1.6	**1.6**	1.7	**1.7**			-11.7	**-11.7**	-1.8	**-1.8**	2.2	**2.2**
SLC1A5	solute carrier family 1 (neutral amino acid transporter), member 5	-1.7	**-1.7**					-2.0	**-2.0**	-2.3	**-2.3**		
SLC20A1	solute carrier family 20 (phosphate transporter), member 1			-1.3	**-1.3**	-2.0		-2.2	**-2.2**	-1.4	**-1.4**	-2.7	**-2.7**
SLC20A2	solute carrier family 20 (phosphate transporter), member 2												
SLC23A2	solute carrier family 23 (nucleobase transporters), member 2							1.3	**1.3**	-1.8	**-1.8**	-2.5	**-2.5**
SLC25A1	solute carrier family 25 (mitochondrial carrier; citrate transporter), member 1			-1.8	**-1.8**					-1.3	**-1.3**	-1.4	**-1.4**
SLC25A11	solute carrier family 25 (mitochondrial carrier; oxoglutarate carrier), member 11			-1.2				-2.4	**-2.4**			1.7	**1.7**
SLC25A12	solute carrier family 25 (mitochondrial carrier, Aralar), member 12					-1.9		1.4				-1.8	**-1.8**
SLC25A3	solute carrier family 25 (mitochondrial carrier; phosphate carrier), member 3							1.2	**1.2**				
SLC25A4	solute carrier family 25 (mitochondrial carrier; adenine nucleotide translocator), member 4					-1.7	**-1.7**						
SLC25A5	solute carrier family 25 (mitochondrial carrier; adenine nucleotide translocator), member 5									-1.2		-1.3	**-1.3**
SLC26A2	solute carrier family 26 (sulfate transporter), member 2			1.6				2.8	**2.8**	1.3	**1.3**		
SLC29A1	solute carrier family 29 (nucleoside transporters), member 1	-1.7	**-1.7**	-2.1	**-2.1**	-1.5		-1.3		-2.6	**-2.6**	-2.0	**-2.0**
SLC2A1	solute carrier family 2 (facilitated glucose transporter), member 1									-1.3		-1.3	
SLC2A3	solute carrier family 2 (facilitated glucose transporter), member 3							-1.9	**-1.9**	6.0	**6.0**	-1.7	**-1.7**
SLC30A1	solute carrier family 30 (zinc transporter), member 1							2.2	**2.2**			-1.8	**-1.8**
SLC30A3	solute carrier family 30 (zinc transporter), member 3					1.5							
SLC30A9	solute carrier family 30 (zinc transporter), member 9					-1.2		2.1	**2.1**	1.3		-1.5	**-1.5**
SLC31A1	solute carrier family 31 (copper transporters), member 1					-1.4		-1.2		-1.8	**-1.8**	-2.3	**-2.3**
SLC35B1	solute carrier family 35, member B1							-1.2		-1.4		-1.4	**-1.4**
SLC39A14	solute carrier family 39 (zinc transporter), member 14	-1.4	**-1.4**	-1.4	**-1.4**	-1.3	**-1.3**	1.9	**1.9**	-3.8	**-3.8**	-5.4	**-5.4**
SLC39A6	solute carrier family 39 (zinc transporter), member 6	-1.4		-1.4	**-1.4**	-1.5	**-1.5**	-1.7	**-1.7**	-1.7	**-1.7**		
SLC39A7	solute carrier family 39 (zinc transporter), member 7									-1.3		-1.4	**-1.4**
SLC39A8	solute carrier family 39 (zinc transporter), member 8	-1.3				-1.4				-3.1	**-3.1**	-3.1	**-3.1**
SLC3A2	solute carrier family 3 (activators of dibasic and neutral amino acid transport), member 2	-1.3		-1.4				-1.3	**-1.3**	-1.3			
SLC43A1	solute carrier family 43, member 1	-1.2						1.8	**1.8**	-2.0	**-2.0**	-2.5	**-2.5**
SLC4A2	solute carrier family 4, anion exchanger, member 2 (erythrocyte membrane protein band 3-like 1)					1.3		-1.5					
SLC6A2	solute carrier family 6 (neurotransmitter transporter, noradrenalin), member 2												
SLC6A6	solute carrier family 6 (neurotransmitter transporter, taurine), member 6	-1.4				1.6		8.8	**8.8**	-1.3		-18.0	**-18.0**
SLC7A1	solute carrier family 7 (cationic amino acid transporter, y+ system), member 1	-1.7		-2.0	**-2.0**	-2.1	**-2.1**	-2.4	**-2.4**	-2.7	**-2.7**	-2.3	**-2.3**
SLC7A5	solute carrier family 7 (cationic amino acid transporter, y+ system), member 5			-1.3				-3.9	**-3.9**			1.5	**1.5**
SLC9A1	solute carrier family 9 (sodium/hydrogen exchanger), member 1 (antiporter, Na+/H+, amiloride sensitive)												
SLC9A3R1	solute carrier family 9 (sodium/hydrogen exchanger), member 3 regulator 1	-1.2		-1.2		-1.9	**-1.9**	1.6	**1.6**	-1.4	**-1.4**	-2.3	**-2.3**
SLCO3A1	solute carrier organic anion transporter family, member 3A1			-1.2									
SLK	STE20-like kinase (yeast)			1.4				4.3	**4.3**	1.4		-2.9	**-2.9**
SMA5	SMA5	-1.3										-1.3	
SMAD1	SMAD family member 1			1.4		-1.6		1.3	**1.2**	2.7	**2.7**	1.3	**1.3**
SMAD2	SMAD family member 2			1.3	**1.2**	-1.2		-1.7	**-1.7**	-1.3	**-1.3**	1.9	**1.9**
SMAD3	SMAD family member 3					1.3				3.8	**3.8**	6.2	**6.2**
SMAD4	SMAD family member 4			1.7	**1.3**	-1.4	**-1.4**	-1.5	**-1.5**	1.6	**1.6**	2.2	**2.2**
SMAD5	SMAD family member 5	1.3		2.4		1.3		-1.5	**-1.5**	-2.1		1.2	
SMAD6	SMAD family member 6							5.6	**5.6**			-6.8	**-6.8**
SMARCA1	SWI/SNF related, matrix associated, actin dependent regulator of chromatin, subfamily a, member 1					-1.3							
SMARCA2	SWI/SNF related, matrix associated, actin dependent regulator of chromatin, subfamily a, member 2	1.7	**1.7**	2.3	**2.3**	1.4	**1.4**	-1.2	**-1.2**	2.2	**2.2**	7.2	**7.2**
SMARCA3	helicase-like transcription factor	-1.3		1.4		-1.3		-1.4	**-1.4**	-1.4	**-1.4**	-1.4	**-1.4**
SMARCA4	SWI/SNF related, matrix associated, actin dependent regulator of chromatin, subfamily a, member 4	-1.3		-1.2	**-1.2**	-1.6		-1.9	**-1.9**	-1.6	**-1.6**	-1.4	
SMARCA5	SWI/SNF related, matrix associated, actin dependent regulator of chromatin, subfamily a, member 5									-1.5	**-1.5**	-1.2	
SMARCB1	SWI/SNF related, matrix associated, actin dependent regulator of chromatin, subfamily b, member 1							-1.3	**-1.3**				
SMARCC1	SWI/SNF related, matrix associated, actin dependent regulator of chromatin, subfamily c, member 1			-1.5	**-1.5**	-1.4		-1.4	**-1.4**	-2.2	**-2.2**	-2.4	**-2.4**
SMARCC2	SWI/SNF related, matrix associated, actin dependent regulator of chromatin, subfamily c, member 2	1.3				1.3		-1.5	**-1.5**	1.4		1.3	**1.2**

SMARCD1	SWI/SNF related, matrix associated, actin dependent regulator of chromatin, subfamily d, member 1							-1.9	**-1.9**	-1.3		1.7	**1.7**
SMARCD2	SWI/SNF related, matrix associated, actin dependent regulator of chromatin, subfamily d, member 2							1.3	**1.3**	-1.2	**-1.2**		
SMARCE1	SWI/SNF related, matrix associated, actin dependent regulator of chromatin, subfamily e, member 1							-2.2	**-2.2**			2.0	**2.0**
SMC1L1	structural maintenance of chromosomes 1A	1.4		-1.2				-2.2	**-2.2**	-1.2	**-1.2**		
SMC2L1	structural maintenance of chromosomes 2			1.3				-2.3	**-2.3**	1.3	**1.3**	-1.3	**-1.3**
SMC4L1	structural maintenance of chromosomes 4	1.7	**1.7**	1.9		1.7	**1.7**	-1.5	**-1.5**	2.0	**1.4**	6.8	**6.8**
SMC5L1	structural maintenance of chromosomes 5	1.2								1.2		-1.5	**-1.5**
SMG1	PI-3-kinase-related kinase SMG-1					-1.2		-1.8	**-1.8**	-1.2		1.8	**1.8**
SMN1	survival of motor neuron 1, telomeric	-1.6	**-1.6**	-1.4		-1.8				-1.2		-1.7	**-1.7**
SMNDC1	survival motor neuron domain containing 1					-1.2		1.6	**1.6**			-1.5	**-1.5**
SMOX	spermine oxidase					10.6	**10.6**	-1.4		2.1	**2.1**	2.7	**2.7**
SMPD1	sphingomyelin phosphodiesterase 1, acid lysosomal (acid sphingomyelinase)	2.3	**2.3**	2.8	**2.8**	1.3							
SMPD2	sphingomyelin phosphodiesterase 2, neutral membrane (neutral sphingomyelinase)												
SMPD4	sphingomyelin phosphodiesterase 4, neutral membrane (neutral sphingomyelinase-3)			-1.2		-1.2		-1.4	**-1.4**				
SMPDL3B	sphingomyelin phosphodiesterase, acid-like 3B											-43.6	**-43.6**
SMS	spermine synthase	1.2				-1.2				-1.4	**-1.4**		
SMTN	smoothelin	1.9	**1.9**							2.0	**2.0**		
SMURF2	SMAD specific E3 ubiquitin protein ligase 2	1.4						1.6	**1.6**	1.6	**1.6**	1.5	**1.5**
SMYD5	SMYD family member 5	-1.4		-1.8	**-1.8**	-1.3							
SNAP23	synaptosomal-associated protein, 23kDa	1.3		1.4								1.5	**1.5**
SNAPC1	small nuclear RNA activating complex, polypeptide 1, 43kDa	-1.4				-1.5		-1.3		-1.5	**-1.5**	-1.2	
SNAPC2	small nuclear RNA activating complex, polypeptide 2, 45kDa					1.5							
SNAPC3	small nuclear RNA activating complex, polypeptide 3, 50kDa					-1.3		-3.2	**-3.2**	1.3	**1.3**	3.3	**3.3**
SNAPC5	small nuclear RNA activating complex, polypeptide 5, 19kDa	1.4	**1.4**			-1.5	**-1.5**			-1.2		-1.7	**-1.7**
SND1	staphylococcal nuclease and tudor domain containing 1							-1.3	**-1.3**				
SNF1LK	SNF1-like kinase											2.5	**2.5**
SNF1LK2	SNF1-like kinase 2					1.7	**1.7**	-1.2					
SNRK	SNF related kinase	1.3		1.8	**1.8**	-1.2		1.2	**1.2**	1.3		1.3	
SNRP70	small nuclear ribonucleoprotein 70kDa polypeptide (RNP antigen)			-1.4	**-1.4**	-1.4				-1.5	**-1.5**	-1.4	**-1.4**
SNRPA	small nuclear ribonucleoprotein polypeptide A									-1.9	**-1.9**	-1.8	**-1.8**
SNRPA1	small nuclear ribonucleoprotein polypeptide A'	-1.3		-1.3	**-1.3**	-1.5		-1.4	**-1.4**	-1.4	**-1.4**	-2.4	**-2.4**
SNRPB	small nuclear ribonucleoprotein polypeptides B and B1			-1.2				-1.6	**-1.6**	-1.7	**-1.7**	-2.4	**-2.4**
SNRPB2	small nuclear ribonucleoprotein polypeptide B''			-1.3	**-1.3**	-1.3		-1.6		-1.4		1.7	**1.7**
SNRPC	small nuclear ribonucleoprotein polypeptide C							-1.3	**-1.3**	-1.5	**-1.5**	-1.3	**-1.3**
SNRPD1	small nuclear ribonucleoprotein D1 polypeptide 16kDa	-1.4	**-1.4**	-3.0	**-3.0**			-1.9	**-1.9**	-1.7	**-1.7**	-2.0	**-2.0**
SNRPD2	small nuclear ribonucleoprotein D2 polypeptide 16.5kDa							-1.2	**-1.2**	-1.2	**-1.2**		
SNRPD3	small nuclear ribonucleoprotein D3 polypeptide 18kDa							-1.6	**-1.6**			-1.4	**-1.4**
SNRPE	small nuclear ribonucleoprotein polypeptide E			-1.4		-1.2		-1.3	**-1.3**	-1.3	**-1.3**		
SNRPG	small nuclear ribonucleoprotein polypeptide G					-1.3						-1.8	**-1.8**
SNRPN	small nuclear ribonucleoprotein polypeptide N	1.3								1.2	**-1.2**	1.5	**1.5**
SNTA1	syntrophin, alpha 1 (dystrophin-associated protein A1, 59kDa, acidic component)	1.4		1.4	**1.4**								
SNTB2	syntrophin, beta 2 (dystrophin-associated protein A1, 59kDa, basic component 2)	3.0	**3.0**	2.9	**2.9**	2.3	**2.3**	-5.9	**-5.9**	3.5	**3.5**	8.7	**8.7**
SNX1	sorting nexin 1							3.6	**3.6**	-1.4		-2.4	**-2.4**
SNX17	sorting nexin 17												
SNX19	sorting nexin 19			1.2								1.7	**1.7**
SNX2	sorting nexin 2			-1.3		-1.3		-1.6	**-1.6**	-1.4	**-1.4**	1.4	**1.4**
SNX3	sorting nexin 3							1.3	**1.3**	-1.5	**-1.5**		
SNX4	sorting nexin 4			1.6		-1.5	**-1.5**	-1.2	**-1.2**	1.6		1.3	**1.3**
SOCS1	suppressor of cytokine signaling 1	15.6	**15.6**	9.9	**9.9**	29.3	**29.3**	-1.6	**-1.6**	8.3	**8.3**	40.4	**40.4**
SOCS2	suppressor of cytokine signaling 2					2.8	**2.8**	-1.8	**-1.8**	2.8	**2.8**	4.7	**4.7**
SOCS5	suppressor of cytokine signaling 5							1.7	**1.7**	1.3	**1.3**		
SOCS6	suppressor of cytokine signaling 6			-1.3		-1.9		1.3	**1.3**	1.9	**1.9**		
SOD1	superoxide dismutase 1, soluble (amyotrophic lateral sclerosis 1 (adult))			-1.2								-1.6	**-1.6**
SON	SON DNA binding protein			1.5		-1.3		1.2		1.7	**1.7**		
SORD	sorbitol dehydrogenase	-1.4		-1.8	**-1.8**	-1.5		-1.3	**-1.3**	-1.6	**-1.6**	-2.9	**-2.9**
SOSTDC1	sclerostin domain containing 1	-1.8		-1.9	**-1.9**								
SOX4	SRY (sex determining region Y)-box 4	-1.2				-1.9		-5.7	**-5.7**	-1.5	**-1.3**	3.0	**3.0**
SP100	SP100 nuclear antigen			1.4				-1.9	**-1.7**	2.3	**2.3**	6.4	**6.4**

SP2	Sp2 transcription factor												
SP3	Sp3 transcription factor					-1.3		-2.8	**-2.8**			2.2	**2.2**
SPA17	sperm autoantigenic protein 17	1.4				1.8		-2.0	**-2.0**	1.4		1.8	**1.8**
SPAG11	sperm associated antigen 11					1.7				-1.3		2.2	**2.2**
SPAG5	sperm associated antigen 5			-1.2						1.5	**1.5**		
SPAG7	sperm associated antigen 7							-1.6	**-1.6**			1.6	
SPAG9	sperm associated antigen 9	1.4				1.3		2.0	**2.0**	1.3	**1.3**	1.3	**1.3**
SPAST	spastin							1.3	**1.3**	1.4		-1.2	**-1.2**
SPBC25	spindle pole body component 25 homolog (S. cerevisiae)	1.5				2.1		1.4	**1.4**			-3.1	**-3.1**
SPCS2	signal peptidase complex subunit 2 homolog (S. cerevisiae)			1.4		-1.4	**-1.4**	1.3	**1.3**	-1.3	**-1.3**	-1.6	**-1.6**
SPEN	spen homolog, transcriptional regulator (Drosophila)					-1.3				-1.3	**-1.3**	1.3	**1.3**
SPHAR	S-phase response (cyclin-related)							3.4	**3.4**	1.3		-3.2	**-3.2**
SPHK2	sphingosine kinase 2							-1.7	**-1.7**			1.6	**1.6**
SPINT1	serine peptidase inhibitor, Kunitz type 1					1.8							
SPINT2	serine peptidase inhibitor, Kunitz type, 2	1.4	**1.4**	1.3	**1.3**	1.3		4.2	**4.2**			-2.6	**-2.6**
SPN	sialophorin (leukosialin, CD43)					-1.3		-1.9	**-1.9**	-1.2	**-1.2**	1.7	**1.7**
SPOCK2	sparc/osteonectin, cwcv and kazal-like domains proteoglycan (testican) 2	3.7	**3.7**	2.4	**2.4**	2.4							
SPOP	speckle-type POZ protein			1.4				1.2					
SPRED2	sprouty-related, EVH1 domain containing 2	1.6		1.6	**1.6**					1.6	**1.6**		
SPRR2C	small proline-rich protein 2C					2.2				-1.7			
SPRY1	sprouty homolog 1, antagonist of FGF signaling (Drosophila)			1.7	**1.7**	1.7	**1.7**			11.8	**11.8**	4.7	**4.7**
SPTAN1	spectrin, alpha, non-erythrocytic 1 (alpha-fodrin)							1.3		-1.3	**-1.3**		
SPTBN1	spectrin, beta, non-erythrocytic 1	1.3						-1.4	**-1.4**	2.4	**2.4**		
SPTLC1	serine palmitoyltransferase, long chain base subunit 1			-1.4				1.7	**1.7**			-1.2	**-1.2**
SQLE	squalene epoxidase	-1.2		-1.4				-2.0	**-2.0**	-1.4	**-1.4**	1.8	**1.8**
SRCAP	Snf2-related CBP activator protein												
SRD5A1	steroid-5-alpha-reductase, alpha polypeptide 1 (3-oxo-5 alpha-steroid delta 4-dehydrogenase alpha 1)	2.7		4.6	**4.6**	3.0	**3.0**	2.0	**2.0**	1.2	**1.2**	4.9	**4.9**
SREBF1	sterol regulatory element binding transcription factor 1	-1.4										1.3	
SREBF2	sterol regulatory element binding transcription factor 2			-1.8	**-1.8**			-1.8	**-1.8**	-1.2		1.7	**1.7**
SRF	serum response factor (c-fos serum response element-binding transcription factor)	-1.5	**-1.5**	-1.3	**-1.3**	-1.3		-1.3	**-1.3**			1.3	
SRGAP2	SLIT-ROBO Rho GTPase activating protein 2	1.3	**1.3**	1.5				1.3		2.3	**2.3**	1.5	**1.5**
SRI	sorcin	-1.4		1.4		-1.6	**-1.6**	1.5	**1.5**			-1.9	**-1.9**
SRM	spermidine synthase	-1.8	**-1.8**	-3.3	**-3.3**	-1.7	**-1.7**			-3.4	**-3.4**	-4.0	**-4.0**
SRP14	signal recognition particle 14kDa (homologous Alu RNA binding protein)			1.3	**1.3**			-1.4	**-1.4**			1.5	**1.5**
SRP19	signal recognition particle 19kDa					-1.3							
SRP54	signal recognition particle 54kDa			1.3	**1.3**	-1.3				1.7	**1.7**	1.5	**1.5**
SRP72	signal recognition particle 72kDa	-1.2		-1.2		-1.6		3.8	**3.8**	-1.5	**-1.5**	-5.1	**-5.1**
SRP9	signal recognition particle 9kDa												
SRPK1	SFRS protein kinase 1	-1.3		-1.2		-1.7	**-1.7**			-1.5	**-1.5**	-1.8	**-1.8**
SRPK2	SFRS protein kinase 2	1.2				1.2		-1.3	**-1.3**	1.2	**1.2**	1.8	**1.8**
SRPR	signal recognition particle receptor ('docking protein')									-1.2		-1.2	**-1.2**
SRR	serine racemase							-2.1	**-2.1**	-2.2	**-2.2**	-1.2	
SRRM1	serine/arginine repetitive matrix 1			-1.3	**-1.3**	-1.3		1.3		-1.5	**-1.5**	-1.6	**-1.6**
SRRM2	serine/arginine repetitive matrix 2			-1.5	**-1.5**			-1.9	**-1.9**	-1.4	**-1.4**	1.7	**1.4**
SS18	synovial sarcoma translocation, chromosome 18	1.3		1.6	**1.6**	1.2		1.5	**1.5**			-1.5	**-1.5**
SS18L1	synovial sarcoma translocation gene on chromosome 18-like 1					1.3		1.6	**1.6**	-1.5	**-1.5**	-2.4	**-2.4**
SSA2	TROVE domain family, member 2			1.5		-1.2				1.4	**1.4**	-1.8	**-1.8**
SSB	Sjogren syndrome antigen B (autoantigen La)	-1.3		-1.6	**-1.6**	-1.6	**-1.6**	1.7	**1.7**	-1.7	**-1.7**	-3.1	**-3.1**
SSBP1	single-stranded DNA binding protein 1	-1.3		-1.2		-1.4		-1.4	**1.3**	-1.8	**-1.8**	-1.4	**-1.4**
SSBP2	single-stranded DNA binding protein 2					-1.6		-7.6	**-7.6**	-1.3		7.2	**7.2**
SSH1	slingshot homolog 1 (Drosophila)			-2.2				2.3	**2.3**	1.5	**-1.4**	-2.2	**-2.2**
SSR1	signal sequence receptor, alpha (translocon-associated protein alpha)	1.2		1.5	**1.5**	1.2		1.2	**1.2**	1.2		-1.4	**-1.4**
SSR2	signal sequence receptor, beta (translocon-associated protein beta)			1.2	**1.2**							1.3	**1.3**
SSR4	signal sequence receptor, delta (translocon-associated protein delta)							1.7	**1.7**	-1.3	**-1.3**	-2.1	**-2.1**
SSRP1	structure specific recognition protein 1			-1.3				-1.8	**-1.8**	-2.2	**-2.2**	-1.9	**-1.9**
SSSCA1	Sjogren's syndrome/scleroderma autoantigen 1			-1.6	**-1.6**	-1.4		-1.6	**-1.6**				
SSTR5	somatostatin receptor 5					1.2							
ST13	suppression of tumorigenicity 13 (colon carcinoma) (Hsp70 interacting protein)	-1.3		-1.2		-1.2		1.5	**1.5**	-1.5	**-1.5**	-2.2	**-2.2**

ST14	suppression of tumorigenicity 14 (colon carcinoma)					1.3							
ST3GAL1	ST3 beta-galactoside alpha-2,3-sialyltransferase 1	-1.5								1.9	**1.9**	1.3	**1.2**
ST3GAL5	ST3 beta-galactoside alpha-2,3-sialyltransferase 5	1.6	**1.6**	1.6	**1.6**			4.7	**4.7**			-10.8	**-10.8**
ST3GAL6	ST3 beta-galactoside alpha-2,3-sialyltransferase 6	2.1	**2.1**	2.3	**2.3**			13.7	**13.7**	1.9	**1.9**	-28.6	**-28.6**
ST5	suppression of tumorigenicity 5	-1.4											
ST6GAL1	ST6 beta-galactosamide alpha-2,6-sialyltranferase 1					1.5		-1.7	**-1.7**	-1.4		1.5	**1.5**
ST6GALNAC4	ST6 (alpha-N-acetyl-neuraminyl-2,3-beta-galactosyl-1,3)-N-acetylgalactosaminide alpha-2,6-sialyltransferase 4					1.4	**1.4**	-1.7	**-1.7**			1.7	**1.7**
ST7	suppression of tumorigenicity 7			-1.2								-2.0	**-2.0**
STAG1	stromal antigen 1			1.5				-1.4	**-1.4**	1.4		1.9	**1.2**
STAG2	stromal antigen 2			1.4				-1.6	**-1.6**	1.3		1.7	**1.7**
STAM	signal transducing adaptor molecule (SH3 domain and ITAM motif) 1	-1.5	**-1.5**			1.3		1.6	**1.6**			-1.9	**-1.9**
STAMBP	STAM binding protein			1.2		-1.5		-1.2	**-1.2**			1.6	**1.6**
STARD3	START domain containing 3					1.3				1.7			
STAT1	signal transducer and activator of transcription 1, 91kDa	1.3		1.4				1.7	**1.7**	-1.5	**-1.5**	-4.2	**-4.2**
STAT2	signal transducer and activator of transcription 2, 113kDa	1.4		1.3	**1.3**	1.4		1.3		1.2	**1.2**	1.5	
STAT3	signal transducer and activator of transcription 3 (acute-phase response factor)	1.3	**1.3**	2.5	**2.1**			3.8	**3.8**	1.3		-1.4	**-1.4**
STAT4	signal transducer and activator of transcription 4	4.2	**4.2**	3.6	**3.6**								
STAT5A	signal transducer and activator of transcription 5A	-1.3	**-1.3**			-1.7	**-1.7**	2.7	**2.7**			-3.2	**-3.2**
STAT5B	signal transducer and activator of transcription 5B			1.4		-1.3				1.5		-1.4	**-1.4**
STAT6	signal transducer and activator of transcription 6, interleukin-4 induced									-1.4	**-1.4**	1.5	**1.5**
STAU	staufen, RNA binding protein, homolog 1 (Drosophila)	-1.5		-1.4		-1.4		-1.3	**-1.2**			1.4	**1.2**
STAU2	staufen, RNA binding protein, homolog 2 (Drosophila)					-1.4		-2.9	**-2.9**	1.6	**1.6**	2.5	**2.5**
STCH	stress 70 protein chaperone, microsome-associated, 60kDa			1.4		-1.2		1.6	**1.6**			-2.5	**-2.5**
STIM1	stromal interaction molecule 1	1.9	**1.9**	2.1	**2.1**	2.0	**2.0**	-1.4	**-1.4**	1.9	**1.9**	3.4	**3.4**
STIP1	stress-induced-phosphoprotein 1 (Hsp70/Hsp90-organizing protein)	-1.8	**-1.8**	-1.4	**-1.4**	-1.6		-1.6	**-1.6**	-1.4	**-1.4**	-1.3	**-1.3**
STK10	serine/threonine kinase 10					1.3		-1.4	**-1.4**			1.7	**1.7**
STK16	serine/threonine kinase 16	2.0	**2.0**	1.9	**1.9**							6.9	**6.9**
STK17B	serine/threonine kinase 17b (apoptosis-inducing)			2.1	**2.1**	-1.5		1.4		1.6	**1.6**	4.3	**4.3**
STK19	serine/threonine kinase 19												
STK24	serine/threonine kinase 24 (STE20 homolog, yeast)							-2.2	**-2.2**	1.3	**1.3**	1.8	**1.8**
STK25	serine/threonine kinase 25 (STE20 homolog, yeast)			1.4		-1.2		-1.3		1.5	**1.5**	1.6	**1.6**
STK3	serine/threonine kinase 3 (STE20 homolog, yeast)							-2.2	**-2.2**	2.3	**1.5**	3.6	**3.6**
STK38	serine/threonine kinase 38			2.3	**2.3**	1.3		-2.3	**-2.3**	1.7	**1.7**	2.1	**2.1**
STK39	serine threonine kinase 39 (STE20/SPS1 homolog, yeast)	-1.2		1.2		-1.3		-2.5	**-2.5**	1.8	**1.8**	3.1	**3.1**
STK4	serine/threonine kinase 4			1.4				-1.4		-1.5	**-1.5**	-1.2	**-1.2**
STK6	aurora kinase A							1.3		1.7	**1.7**	-1.6	**-1.6**
STMN1	stathmin 1/oncoprotein 18							-1.3		-1.4	**-1.4**	-1.8	**-1.8**
STOM	stomatin					1.7		6.2	**6.2**	-1.8	**-1.8**	-13.8	**-13.8**
STOML2	stomatin (EPB72)-like 2					-1.2				-1.5	**-1.5**	-2.8	**-2.8**
STRA13	stimulated by retinoic acid 13 homolog (mouse)	-1.8		-2.2	**-2.2**	-1.9	**-1.9**					-28.6	**-28.6**
STRAP	serine/threonine kinase receptor associated protein					-1.4		-1.4	**-1.4**			2.1	**2.1**
STRN3	striatin, calmodulin binding protein 3									1.5	**1.5**	1.7	
STS	steroid sulfatase (microsomal), arylsulfatase C, isozyme S	-1.6	**-1.6**	-1.3		-1.4		-3.6	**-3.6**	-1.3		2.4	**2.4**
STX12	syntaxin 12	1.3											
STX16	syntaxin 16			1.8	**1.8**	1.2		1.5	**1.5**			1.5	**1.5**
STX3A	syntaxin 3	1.3		1.4		1.2		1.7	**1.7**	2.1			
STX4A	syntaxin 4					-1.2				-1.2		1.8	**1.8**
STX5A	syntaxin 5			1.5	**1.5**					1.2		1.3	
STX6	syntaxin 6							-1.9	**-1.9**			1.7	**1.7**
STX7	syntaxin 7							1.5	**1.5**	1.5	**1.3**	1.7	**1.4**
STX8	syntaxin 8			1.3		-1.4		-2.6	**-2.6**			3.2	**3.2**
STXBP1	syntaxin binding protein 1	2.0	**2.0**	2.0	**2.0**	3.8	**3.8**	-13.5	**-13.5**	1.2		11.7	**11.7**
STXBP2	syntaxin binding protein 2	1.3				1.3							
STXBP3	syntaxin binding protein 3							-1.2	**-1.2**	1.7	**1.7**	1.9	**1.9**
SUCLA2	succinate-CoA ligase, ADP-forming, beta subunit					-1.6				-1.3	**-1.3**		
SULT1A1	sulfotransferase family, cytosolic, 1A, phenol-preferring, member 1	1.7		1.5		1.5		-1.5	**-1.5**			2.2	**2.2**
SULT4A1	sulfotransferase family 4A, member 1												
SUMO1	SMT3 suppressor of mif two 3 homolog 1 (S. cerevisiae)					-1.5		1.6	**1.2**	1.4	**-1.3**	-2.1	**-2.1**

SUMO2	SMT3 suppressor of mif two 3 homolog 2 (S. cerevisiae)							3.0	**3.0**	1.4		-3.1	**-3.1**
SUMO3	SMT3 suppressor of mif two 3 homolog 3 (S. cerevisiae)							-1.3		-1.3	**-1.3**	-1.4	**-1.4**
SUMO4	SMT3 suppressor of mif two 3 homolog 4 (S. cerevisiae)												
SUPT3H	suppressor of Ty 3 homolog (S. cerevisiae)	1.3						-6.4	**-6.4**			10.6	**10.6**
SUPT4H1	suppressor of Ty 4 homolog 1 (S. cerevisiae)							-1.7	**-1.7**	1.6	**1.6**	2.0	**2.0**
SUPT5H	suppressor of Ty 5 homolog (S. cerevisiae)					1.2							
SUPT6H	suppressor of Ty 6 homolog (S. cerevisiae)							1.8	**1.8**	1.5		1.8	**-1.5**
SURB7	SRB7 suppressor of RNA polymerase B homolog (yeast)			1.6	**1.6**	-1.3		1.3				-1.4	**-1.4**
SURF1	surfeit 1												
SURF5	surfeit 5	-1.2						-1.4	**-1.4**				
SUZ12	suppressor of zeste 12 homolog (Drosophila)					-1.3				-1.3		-1.8	**-1.8**
SV2A	synaptic vesicle glycoprotein 2A	1.7	**1.7**	1.8	**1.8**	1.4	**1.4**	10.1	**10.1**	1.9	**1.9**	-14.8	**-14.8**
SWAP70	SWAP-70 protein	1.3	**1.3**	1.4				-1.3	**-1.3**	1.4	**1.4**	1.6	**1.6**
SYBL1	synaptobrevin-like 1							1.3	**1.3**	1.2	**1.2**		
SYCP1	synaptonemal complex protein 1					2.9	**2.9**					1.5	
SYMPK	symplekin									-1.7	**-1.7**	-1.5	**-1.5**
SYNCRIP	synaptotagmin binding, cytoplasmic RNA interacting protein	-1.3		-1.5	**-1.5**	-1.4	**-1.4**	-1.7	**-1.7**	-1.6	**-1.6**	-1.9	**-1.9**
SYNE1	spectrin repeat containing, nuclear envelope 1					1.2							
SYNE2	spectrin repeat containing, nuclear envelope 2	1.5	**1.5**			-1.5		-10.7	**-10.7**	6.9	**6.9**	38.9	**38.9**
SYNGR2	synaptogyrin 2							1.9	**1.9**			-2.4	**-2.4**
SYNJ1	synaptojanin 1	1.5		1.8				-1.3		1.2			
SYNJ2	synaptojanin 2	1.9	**1.9**	2.4	**2.4**	1.5		-13.7	**-13.7**			48.6	**48.6**
SYNPO	synaptopodin	1.2				1.9							
SYPL	synaptophysin-like 1							1.3				-1.9	**-1.9**
SYT1	synaptotagmin I	1.7						-16.7	**-16.7**	-1.5	**-1.5**	12.7	**12.7**
T	T, brachyury homolog (mouse)	1.2		1.5		1.6							
TACC1	transforming, acidic coiled-coil containing protein 1							2.5	**2.5**	1.6		1.7	**1.7**
TADA3L	transcriptional adaptor 3 (NGG1 homolog, yeast)-like			1.4	**1.4**							1.3	**1.3**
TAF1	TAF1 RNA polymerase II, TATA box binding protein (TBP)-associated factor, 250kDa			1.7				1.9	**1.9**	1.2		-1.4	**-1.4**
TAF10	TAF10 RNA polymerase II, TATA box binding protein (TBP)-associated factor, 30kDa							-1.4	**-1.4**			1.4	**1.4**
TAF11	TAF11 RNA polymerase II, TATA box binding protein (TBP)-associated factor, 28kDa	1.3		1.8	**1.8**					1.2	**1.2**	-1.2	**-1.2**
TAF12	TAF12 RNA polymerase II, TATA box binding protein (TBP)-associated factor, 20kDa			-1.2		-1.3							
TAF15	TAF15 RNA polymerase II, TATA box binding protein (TBP)-associated factor, 68kDa							1.6	**1.6**	-1.4	**-1.4**	-1.7	**-1.7**
TAF1A	TATA box binding protein (TBP)-associated factor, RNA polymerase I, A, 48kDa					-1.4		-2.1	**-2.1**	-1.8	**-1.8**		
TAF1C	TATA box binding protein (TBP)-associated factor, RNA polymerase I, C, 110kDa							1.8	**1.8**			-1.6	**-1.6**
TAF2	TAF2 RNA polymerase II, TATA box binding protein (TBP)-associated factor, 150kDa							-1.3	**-1.3**	-1.7		1.6	**1.6**
TAF4	TAF4 RNA polymerase II, TATA box binding protein (TBP)-associated factor, 135kDa			1.3		1.2						-1.2	
TAF4B	TAF4b RNA polymerase II, TATA box binding protein (TBP)-associated factor, 105kDa							-1.3	**-1.3**			-2.3	**-2.3**
TAF5	TAF5 RNA polymerase II, TATA box binding protein (TBP)-associated factor, 100kDa									-1.4	**-1.4**	-1.7	**-1.7**
TAF5L	TAF5-like RNA polymerase II, p300/CBP-associated factor (PCAF)-associated factor, 65kDa			-1.3				-1.4					
TAF6	TAF6 RNA polymerase II, TATA box binding protein (TBP)-associated factor, 80kDa							-1.4				1.4	
TAF6L	TAF6-like RNA polymerase II, p300/CBP-associated factor (PCAF)-associated factor, 65kDa			-1.2		1.3						-1.4	
TAF7	TAF7 RNA polymerase II, TATA box binding protein (TBP)-associated factor, 55kDa			1.5	**1.5**							1.3	**1.3**
TAF9	TAF9 RNA polymerase II, TATA box binding protein (TBP)-associated factor, 32kDa			-1.3				2.2	**2.2**			-3.2	**-3.2**
TAGLN2	transgelin 2	-1.4	**-1.4**	-2.0	**-2.0**	-1.3		-3.0	**-3.0**			-1.3	
TAGLN3	transgelin 3	1.3											
TAL1	T-cell acute lymphocytic leukemia 1	1.6	**1.6**					1.7	**1.7**			-2.3	**-2.3**
TALDO1	transaldolase 1							1.8	**1.8**	1.4		-1.7	**-1.7**
TANK	TRAF family member-associated NFKB activator							2.1	**2.1**	1.4	**1.4**	-1.3	**-1.3**
TAOK3	TAO kinase 3	1.3						2.3	**2.3**	-1.4	**-1.4**	-2.9	**-2.9**
TAP1	transporter 1, ATP-binding cassette, sub-family B (MDR/TAP)			1.6	**1.6**	1.7				1.9	**1.9**		
TAPBP	TAP binding protein (tapasin)							-1.8	**-1.8**	-1.3	**-1.3**	1.2	**1.2**
TARBP1	Tar (HIV-1) RNA binding protein 1	-1.6	**-1.6**	-1.6	**-1.6**	-1.8	**-1.8**	1.3		-1.3		-3.5	**-3.5**
TARBP2	Tar (HIV-1) RNA binding protein 2			-1.5						-1.4	**-1.4**		
TARDBP	TAR DNA binding protein			-1.2	**-1.2**	-1.3		1.9	**1.9**	-1.5	**-1.2**	-1.3	**-1.3**
TARS	threonyl-tRNA synthetase			-1.5	**-1.5**	-1.6	**-1.6**	1.4		-1.9	**-1.9**	-1.8	**-1.8**
TAX1BP1	Tax1 (human T-cell leukemia virus type I) binding protein 1	1.5	**1.5**	1.7	**1.7**			4.5	**4.5**	1.4	**1.4**	-2.6	**-2.6**
TAX1BP3	Tax1 (human T-cell leukemia virus type I) binding protein 3	1.6		1.5	**1.5**	1.4		-2.1	**-2.1**	3.3	**3.3**	4.2	**4.2**

TAZ	tafazzin (cardiomyopathy, dilated 3A (X-linked); endocardial fibroelastosis 2; Barth syndrome)							-1.2				1.4	**1.4**
TBC1D1	TBC1 (tre-2/USP6, BUB2, cdc16) domain family, member 1									2.7	**2.7**	7.0	**7.0**
TBC1D22A	TBC1 domain family, member 22A					1.8		-1.5	**-1.5**	1.3		-1.2	**-1.2**
TBC1D4	TBC1 domain family, member 4	-1.4				-1.3	**-1.3**	-1.4		-1.8	**-1.8**	-1.6	**-1.6**
TBC1D8	TBC1 domain family, member 8 (with GRAM domain)	1.4						1.7	**1.7**	-1.6	**-1.6**	-2.7	**-2.7**
TBCA	tubulin folding cofactor A	-1.3		-1.4		-1.3				1.3			
TBCC	tubulin folding cofactor C			1.3	**1.3**							1.5	**1.5**
TBCD	tubulin folding cofactor D	1.7	**1.7**	1.5	**1.5**	3.4	**3.4**			2.4	**2.4**	2.4	**2.4**
TBCE	tubulin folding cofactor E			1.2	**1.2**	-1.2		1.3	**1.3**	1.3		-1.3	**-1.3**
TBL1X	transducin (beta)-like 1X-linked	1.5		1.8	**1.8**			2.2	**2.2**	1.3	**1.3**	2.3	**2.3**
TBP	TATA box binding protein									-1.3	**-1.3**		
TBPIP	PSMC3 interacting protein							-1.3		1.4		-2.0	
TBPL1	TBP-like 1							-1.6	**-1.6**			1.5	**1.5**
TBX19	T-box 19					1.4							
TBX5	T-box 5												
TCEA1	transcription elongation factor A (SII), 1			-1.3	**-1.3**			-1.7	**-1.7**	1.3		1.5	**1.5**
TCEAL1	transcription elongation factor A (SII)-like 1					1.6		1.8	**1.8**			-1.4	**-1.4**
TCEB1	transcription elongation factor B (SIII), polypeptide 1 (15kDa, elongin C)			-1.4		-1.4		-1.6	**-1.6**	1.4	**1.4**		
TCEB2	transcription elongation factor B (SIII), polypeptide 2 (18kDa, elongin B)							-2.9	**-2.9**			1.4	**1.4**
TCERG1	transcription elongation regulator 1			-1.5	**-1.5**	-1.4	**-1.4**	-1.4	**-1.4**	1.4	**-1.4**	1.8	**-1.4**
TCF12	transcription factor 12 (HTF4, helix-loop-helix transcription factors 4)					-1.2		-2.5	**-2.5**	-2.0	**-2.0**	2.8	**2.8**
TCF20	transcription factor 20 (AR1)					-1.4				-1.5			
TCF3	transcription factor 3 (E2A immunoglobulin enhancer binding factors E12/E47)			-1.4	**-1.4**	-2.0		-4.7	**-4.7**	-1.6		4.7	**4.7**
TCF7	transcription factor 7 (T-cell specific, HMG-box)					-1.3	**-1.3**						
TCFL1	vacuolar protein sorting 72 homolog (S. cerevisiae)	-1.3	**-1.3**			-1.5	**-1.5**			-1.3	**-1.3**	-1.6	**-1.6**
TCFL5	transcription factor-like 5 (basic helix-loop-helix)							-2.5	**-2.5**	2.1	**2.1**	2.8	**2.8**
TCOF1	Treacher Collins-Franceschetti syndrome 1	-1.3		-1.3						-1.5	**-1.5**	-1.2	
TCP1	t-complex 1	-1.4		-1.5	**-1.5**	-1.7	**-1.7**	-1.3	**-1.3**	-1.5	**-1.5**	-1.6	**-1.6**
TCTE1L	dynein, light chain, Tctex-type 3	1.2						-1.2	**-1.2**	-1.9		1.9	**1.9**
TCTEL1	dynein, light chain, Tctex-type 1							1.2	**1.2**	1.5	**1.5**	1.6	
TDE1	serine incorporator 3	1.3		1.7	**1.7**			1.4		1.6	**1.6**	1.5	**1.4**
TDE2	serine incorporator 1	1.2		1.5						2.0	**2.0**	2.1	**2.1**
TDG	thymine-DNA glycosylase	-1.3				-1.5		-1.3				1.3	**1.3**
TDRD3	tudor domain containing 3					-1.4	**-1.4**	-1.2		1.2			
TDRD7	tudor domain containing 7							-2.6	**-2.6**	1.3		5.6	**5.6**
TEBP	prostaglandin E synthase 3 (cytosolic)					-1.4				-1.4	**-1.4**	-1.3	**-1.3**
TEGT	testis enhanced gene transcript (BAX inhibitor 1)			1.7		1.3		1.2	**1.2**	1.4	**1.4**	1.3	**1.3**
TERF1	telomeric repeat binding factor (NIMA-interacting) 1			1.7		1.5		-1.6	**-1.6**			2.0	**2.0**
TERF2	telomeric repeat binding factor 2							-1.5	**-1.5**			1.4	**1.4**
TERF2IP	telomeric repeat binding factor 2, interacting protein					1.2		-1.3				1.4	**1.4**
TES	testis derived transcript (3 LIM domains)	1.4		1.6	**1.6**	-1.2		1.4	**1.4**			-1.2	
TFAM	transcription factor A, mitochondrial	-1.3		-1.5		-1.8	**-1.8**	-1.4	**-1.4**	-1.8	**-1.8**	-2.0	**-2.0**
TFAP4	transcription factor AP-4 (activating enhancer binding protein 4)	-1.6	**-1.6**	-3.0	**-3.0**	-1.5	**-1.5**	-2.6					
TFCP2	transcription factor CP2			1.4				-1.5	**-1.5**	-1.9	**-1.9**	1.8	**1.8**
TFDP1	transcription factor Dp-1					-1.3		-1.8	**-1.8**	-1.3	**-1.3**	-1.6	**-1.6**
TFDP2	transcription factor Dp-2 (E2F dimerization partner 2)	-1.6	**-1.5**	-1.3		-1.5		3.1	**3.1**	-1.4	**-1.4**	1.3	
TFE3	transcription factor binding to IGHM enhancer 3	1.2										2.1	**2.1**
TFF3	trefoil factor 3 (intestinal)					1.5							
TFPI	tissue factor pathway inhibitor (lipoprotein-associated coagulation inhibitor)	3.1	**3.1**	5.4	**5.4**	1.9	**1.9**	4.9	**4.9**	9.6	**9.6**	24.6	**24.6**
TFRC	transferrin receptor (p90, CD71)									-4.1	**-4.1**	-3.0	**-3.0**
TGDS	TDP-glucose 4,6-dehydratase	-1.3	**-1.3**	-1.4								-1.3	**-1.3**
TGFB1	transforming growth factor, beta 1 (Camurati-Engelmann disease)					1.3		32.0	**32.0**			-3.3	**-3.3**
TGFB3	transforming growth factor, beta 3					9.9	**9.9**						
TGFBR1	transforming growth factor, beta receptor I (activin A receptor type II-like kinase, 53kDa)			1.5				-1.5				1.7	**1.7**
TGFBR2	transforming growth factor, beta receptor II (70/80kDa)	2.8	**2.8**	6.1	**6.1**	4.0	**4.0**	-3.3	**-3.3**	1.8	**1.8**	6.9	**6.9**
TGIF2	TGFB-induced factor 2 (TALE family homeobox)	-1.3		-1.7	**-1.7**	-1.4		1.9	**1.9**	-1.6	**-1.6**	-2.9	**-2.9**
TGOLN2	trans-golgi network protein 2			1.2		2.2		1.6	**1.3**	1.4	**1.2**	-1.6	**-1.6**
THBS1	thrombospondin 1	-1.3								13.1	**13.1**		

THOC1	THO complex 1											-1.8	**-1.8**
THOC2	THO complex 2	1.5						1.5	**1.5**	1.6	**1.6**	-1.5	**-1.5**
THOP1	thimet oligopeptidase 1							-1.4		-1.4		-2.1	**-2.1**
THRA	thyroid hormone receptor, alpha (erythroblastic leukemia viral (v-erb-a) oncogene homolog, avian)	-1.3		1.2		1.5		1.3		1.2		2.3	**2.3**
THRAP1	thyroid hormone receptor associated protein 1			1.4		-1.4		1.9	**1.9**	1.5	**1.5**	-1.3	**-1.3**
THRAP2	thyroid hormone receptor associated protein 2							-1.5	**-1.5**	4.2	**4.2**	2.3	**2.3**
TIA1	TIA1 cytotoxic granule-associated RNA binding protein							-1.7	**-1.7**	1.4	**-1.3**	1.8	**1.8**
TIAL1	TIA1 cytotoxic granule-associated RNA binding protein-like 1	1.3				2.1		1.7	**1.7**	-1.4	**-1.4**	-2.0	**-2.0**
TIAM1	T-cell lymphoma invasion and metastasis 1					-1.3				2.2	**2.2**		
TIF1	tripartite motif-containing 24					2.5		1.6	**1.6**			-1.5	**-1.5**
TIMELESS	timeless homolog (Drosophila)							-1.2		-1.5	**-1.5**	-2.2	**-2.2**
TIMM17A	translocase of inner mitochondrial membrane 17 homolog A (yeast)	-1.7		-1.3		-1.6	**-1.5**	-1.2	**-1.2**	-1.5	**-1.5**	-1.6	**-1.6**
TIMM44	translocase of inner mitochondrial membrane 44 homolog (yeast)			-1.6				-1.2				-4.0	**-4.0**
TIMP1	TIMP metallopeptidase inhibitor 1	1.7		1.5	**1.5**	1.7		1.4				-1.2	**-1.2**
TIMP2	TIMP metallopeptidase inhibitor 2					-1.3		2.1	**2.1**	1.3		-3.0	**-3.0**
TIP120A	cullin-associated and neddylation-dissociated 1					-1.3		1.6	**1.6**	-1.3	**-1.3**	-1.4	**-1.4**
TIPARP	TCDD-inducible poly(ADP-ribose) polymerase	1.3								4.0	**4.0**	6.1	**6.1**
TITF1	thyroid transcription factor 1	1.2											
TJP2	tight junction protein 2 (zona occludens 2)	1.3						1.6		-1.8	**-1.8**	-4.0	**-4.0**
TK1	thymidine kinase 1, soluble	1.4	**1.4**	1.6	**1.6**							-3.6	**-3.6**
TKT	transketolase (Wernicke-Korsakoff syndrome)	-1.4		-1.3	**-1.3**			1.5	**1.5**	-1.3	**-1.3**	-1.8	**-1.8**
TLE1	transducin-like enhancer of split 1 (E(sp1) homolog, Drosophila)	1.4		2.0	**2.0**			-2.0	**-2.0**	5.6	**5.6**	7.4	**7.4**
TLE4	transducin-like enhancer of split 4 (E(sp1) homolog, Drosophila)			1.3	**1.3**	-1.2		-1.4	**-1.4**	-1.5	**-1.5**	1.7	**1.7**
TLK1	tousled-like kinase 1			1.3				1.7	**1.7**	1.4	**1.4**	1.4	
TLK2	tousled-like kinase 2							1.7	**1.7**				
TLN1	talin 1	1.4	**1.4**	1.4	**1.4**			-1.6	**-1.6**			2.6	**2.6**
TM2D1	TM2 domain containing 1	1.2						1.6	**1.6**	1.8	**1.8**	1.8	**1.8**
TM4SF2	tetraspanin 7					-1.3		35.8	**35.8**	4.8	**4.8**	-11.4	**-11.4**
TM4SF7	tetraspanin 4									-1.6	**-1.6**	-1.3	**-1.3**
TM4SF8	tetraspanin 3			1.3	**1.3**								
TM7SF2	transmembrane 7 superfamily member 2					1.4		2.6	**2.6**			-1.6	**-1.6**
TMED2	transmembrane emp24 domain trafficking protein 2							-1.3					
TMED3	transmembrane emp24 protein transport domain containing 3	-1.7	**-1.7**	-1.9	**-1.9**					-1.3		-1.4	**-1.4**
TMED7	transmembrane emp24 protein transport domain containing 7			1.9	**1.9**	1.5		1.6	**1.6**	1.7	**1.7**		
TMED9	transmembrane emp24 protein transport domain containing 9					1.4		1.8	**1.8**			-2.4	**-2.4**
TMEM1	transmembrane protein 1			2.0	**2.0**			-1.4	**-1.4**	1.4		1.7	**1.5**
TMEM11	transmembrane protein 11			-1.2	**-1.2**	-1.2		-1.7	**-1.7**			1.2	
TMEM4	transmembrane protein 4					-1.2		1.9	**1.9**	-1.5	**-1.5**	-3.3	**-3.3**
TMEM87A	transmembrane protein 87A							1.2	**1.2**	1.3	**1.3**	1.4	
TMF1	TATA element modulatory factor 1	-1.2	**-1.2**			-1.3		2.1	**2.1**	1.7	**1.7**	-2.2	**-2.2**
TMP21	transmembrane emp24-like trafficking protein 10 (yeast)			1.3	**1.3**	-1.4		-1.5	**1.5**			2.0	**2.0**
TMPO	thymopoietin							-2.2	**-2.2**	1.5	**1.4**	1.7	**1.7**
TMSB10	thymosin, beta 10	1.4	**1.4**	1.4	**1.4**	2.2	**2.2**	-1.9	**-1.9**	1.6	**1.6**	3.2	**3.2**
TMSB4X	thymosin, beta 4, X-linked									1.7	**1.7**	1.6	**1.6**
TMSL8	thymosin-like 8	1.5						-5.4	**-5.4**			2.0	**2.0**
TNF	tumor necrosis factor (TNF superfamily, member 2)												
TNFAIP1	tumor necrosis factor, alpha-induced protein 1 (endothelial)											1.2	
TNFAIP3	tumor necrosis factor, alpha-induced protein 3					-1.7	**-1.7**					4.1	**4.1**
TNFAIP8	tumor necrosis factor, alpha-induced protein 8	-1.3	**-1.3**			-1.7		-1.5	**-1.5**	-1.9	**-1.9**	-1.9	**-1.9**
TNFRSF10D	tumor necrosis factor receptor superfamily, member 10d, decoy with truncated death domain					1.4		1.3	**1.3**			-2.0	**-2.0**
TNFRSF1A	tumor necrosis factor receptor superfamily, member 1A							2.9	**2.9**	1.2		-5.7	**-5.7**
TNFRSF21	tumor necrosis factor receptor superfamily, member 21	-1.2								6.3	**6.3**	4.2	**4.2**
TNFRSF25	tumor necrosis factor receptor superfamily, member 25					1.3							
TNFRSF7	CD27 molecule					3.2	**3.2**			2.3	**2.3**		
TNFSF10	tumor necrosis factor (ligand) superfamily, member 10	1.5		1.9				-1.4		-5.3	**-5.3**	-4.1	**-4.1**
TNFSF8	tumor necrosis factor (ligand) superfamily, member 8	5.2	**5.2**	22.4	**22.4**							18.3	**18.3**
TNK2	tyrosine kinase, non-receptor, 2					1.3				1.6	**1.6**	1.5	
TNKS	tankyrase, TRF1-interacting ankyrin-related ADP-ribose polymerase							-1.7	**-1.7**	2.5	**2.5**	1.5	**1.3**

TNP1	transition protein 1 (during histone to protamine replacement)					1.4							
TNPO1	transportin 1					-1.6		-1.4	**-1.4**	1.4	**1.4**	-1.6	**-1.6**
TNPO3	transportin 3			1.2		-1.2		1.4	**1.4**			-1.4	
TOB1	transducer of ERBB2, 1	1.6	**1.6**	2.3	**2.3**	1.5		1.9	**1.9**	-1.6	**-1.6**		
TOB2	transducer of ERBB2, 2							1.9	**1.9**	1.4	**1.4**		
TOE1	target of EGR1, member 1 (nuclear)			-1.4		-1.5	**-1.5**					-1.4	**-1.4**
TOMM20	translocase of outer mitochondrial membrane 20 homolog (yeast)	-1.5	**-1.5**	-1.2		-1.4		2.0	**2.0**			-2.1	**-2.1**
TOMM34	translocase of outer mitochondrial membrane 34	-1.3		-1.3								-1.4	**-1.4**
TOMM40	translocase of outer mitochondrial membrane 40 homolog (yeast)	-1.5		-2.0	**-2.0**	-1.3		-1.2		-1.4	**-1.4**	-1.6	**-1.6**
TOMM7	translocase of outer mitochondrial membrane 7 homolog (yeast)												
TOMM70A	translocase of outer mitochondrial membrane 70 homolog A (S. cerevisiae)	-1.4	**-1.4**	-1.2		-1.4				-1.5	**-1.5**	-1.8	**-1.8**
TOP1	topoisomerase (DNA) I			1.2		-1.3							
TOP2A	topoisomerase (DNA) II alpha 170kDa	1.4	**1.4**	1.8	**1.8**	1.2				1.8	**1.8**	1.3	**1.3**
TOP2B	topoisomerase (DNA) II beta 180kDa			2.2	**1.3**	-1.4		-1.9	**-1.9**			1.4	**1.4**
TOP3B	topoisomerase (DNA) III beta					1.6							
TOPBP1	topoisomerase (DNA) II binding protein 1							-1.2	**-1.2**			-1.5	**-1.5**
TOPORS	topoisomerase I binding, arginine/serine-rich							1.4				-1.2	
TOR1A	torsin family 1, member A (torsin A)	1.4				1.3							
TOX	thymus high mobility group box protein TOX	-1.3	**-1.3**			-1.3		26.1	**26.1**	-1.6	**-1.6**	-32.2	**-32.2**
TP53	tumor protein p53 (Li-Fraumeni syndrome)					-1.3		-2.6	**-2.6**	-2.5	**-2.5**		
TP53BP1	tumor protein p53 binding protein, 1			1.3									
TP53BP2	tumor protein p53 binding protein, 2			1.4		1.7	**1.7**	2.0	**2.0**	1.3			
TP53I11	tumor protein p53 inducible protein 11												
TPD52L2	tumor protein D52-like 2			-1.2				-1.6	**-1.6**			1.4	**1.4**
TPI1	triosephosphate isomerase 1			-1.5	**-1.5**			-1.5	**-1.5**	-1.5	**-1.5**		
TPM1	tropomyosin 1 (alpha)	1.3		1.4		1.6		-3.2	**-3.2**			8.6	**8.6**
TPM4	tropomyosin 4					-1.7		-2.5	**-2.5**	1.8	**1.8**	2.6	**2.6**
TPMT	thiopurine S-methyltransferase			1.3				1.8	**1.8**			-2.2	**-2.2**
TPO	thyroid peroxidase	2.1		2.8	**2.8**	5.0	**5.0**						
TPP1	tripeptidyl peptidase I	1.2		1.5	**1.5**	1.3		1.7	**1.7**	1.4	**1.4**	-1.3	**-1.3**
TPP2	tripeptidyl peptidase II							-1.3	**-1.3**			-1.3	**-1.3**
TPR	translocated promoter region (to activated MET oncogene)							1.4	**1.4**	-1.3	**-1.3**	-1.6	**-1.6**
TPSAB1	tryptase alpha/beta 1												
TPST2	tyrosylprotein sulfotransferase 2	1.3				1.3		-1.7	**-1.7**	2.0	**2.0**	2.9	**2.9**
TPT1	tumor protein, translationally-controlled 1												
TPX2	TPX2, microtubule-associated, homolog (Xenopus laevis)												
TRA@	T cell receptor alpha locus			2.0	**2.0**	2.9	**2.9**	-38.6	**-38.6**	-2.8	**-1.9**	24.7	**24.7**
TRA1	heat shock protein 90kDa beta (Grp94), member 1	-1.3		-1.2	**-1.2**	-1.5		1.4	**1.4**			-2.2	**-2.2**
TRA2A	transformer-2 alpha					-1.2		2.6	**2.6**			-2.3	**-2.3**
TRAF2	TNF receptor-associated factor 2												
TRAF3	TNF receptor-associated factor 3	1.3		1.7				2.8	**2.8**			-7.4	**-7.4**
TRAF3IP2	TRAF3 interacting protein 2	1.6		2.1	**2.1**	1.8		-5.3	**-5.3**	-1.2		3.8	**3.8**
TRAF4	TNF receptor-associated factor 4	-1.7	**-1.7**	-1.6	**-1.6**	-1.5	**-1.5**	-13.8	**-13.8**	-1.7	**-1.7**	1.3	
TRAF5	TNF receptor-associated factor 5					1.3							
TRAM1	translocation associated membrane protein 1			-1.4				3.3	**3.3**	1.2		-2.3	**-2.3**
TRAM2	translocation associated membrane protein 2	1.5	**1.5**	1.5		1.7	**1.7**	-2.0	**-2.0**	2.4	**2.4**	2.0	**2.0**
TRAP1	TNF receptor-associated protein 1	-1.6	**-1.6**	-1.8	**-1.8**	-1.6		-1.3		-1.9	**-1.9**	-1.8	**-1.8**
TRAPPC3	trafficking protein particle complex 3	-1.2				-1.3		1.4	**1.4**			-1.4	**-1.4**
TRAT1	T cell receptor associated transmembrane adaptor 1	1.3											
TRFP	Trf (TATA binding protein-related factor)-proximal homolog (Drosophila)							-1.7	**-1.7**	-1.4	**-1.4**		
TRIB2	tribbles homolog 2 (Drosophila)					1.7		6.1	**6.1**			-6.0	**-6.0**
TRIM14	tripartite motif-containing 14							3.2	**3.2**	-2.4	**-2.4**	-4.7	**-4.7**
TRIM16	tripartite motif-containing 16					1.5							
TRIM21	tripartite motif-containing 21	1.4	**1.4**							-1.6	**-1.6**	1.4	**1.4**
TRIM23	tripartite motif-containing 23			1.6						1.8	**1.8**	1.3	**1.3**
TRIM28	tripartite motif-containing 28	-1.2		-1.5						-1.3	**-1.3**	-2.0	**-2.0**
TRIM32	tripartite motif-containing 32	1.3		1.7								1.5	**1.5**
TRIM33	tripartite motif-containing 33							-1.6	**-1.6**	1.3		-1.2	**-1.2**

TRIM37	tripartite motif-containing 37			-1.2		-1.3		1.8	**1.8**	-1.3	**-1.3**	-14.1	**-14.1**
TRIM44	tripartite motif-containing 44	-1.3				-1.3		-1.2					
TRIM9	tripartite motif-containing 9									1.7	**1.7**		
TRIP	TRAF interacting protein												
TRIP12	thyroid hormone receptor interactor 12	1.4		1.5	**1.5**			1.6	**1.6**	1.5	**1.5**	2.3	**2.3**
TRIP13	thyroid hormone receptor interactor 13	-1.5	**-1.5**	-1.4	**-1.4**	-1.6	**-1.6**	1.2				-2.4	**-2.4**
TROAP	trophinin associated protein (tastin)	1.5	**1.5**										
TRPC4AP	transient receptor potential cation channel, subfamily C, member 4 associated protein												
TRRAP	transformation/transcription domain-associated protein							-1.7	**-1.7**			2.1	**2.1**
TSC1	tuberous sclerosis 1	1.5	**1.5**							1.2			
TSC2	tuberous sclerosis 2							-1.8	**-1.8**	1.5	**1.5**	6.8	**6.8**
TSC22D1	TSC22 domain family, member 1	1.3						2.0	**2.0**	2.9	**2.9**	-2.4	**-2.4**
TSC22D3	TSC22 domain family, member 3	33.1	**33.1**	74.0	**74.0**	20.4	**20.4**	5.2	**5.2**	17.5	**17.5**	20.0	**20.0**
TSC22D4	TSC22 domain family, member 4									1.4		2.8	**2.8**
TSFM	Ts translation elongation factor, mitochondrial	-1.5	**-1.5**	-1.4	**-1.4**	-1.6	**-1.6**	-1.2	**-1.2**	-2.5	**-2.5**	-1.6	**-1.6**
TSG101	tumor susceptibility gene 101							-1.2				1.6	**1.6**
TSHR	thyroid stimulating hormone receptor					-4.1	**-4.1**						
TSN	translin									-1.3	**-1.3**	-1.4	**-1.4**
TSNAX	translin-associated factor X	2.1	**2.1**	2.6	**2.6**	2.2	**2.2**	1.2	**1.2**	1.9	**1.9**	2.0	**2.0**
TSPAN5	tetraspanin 5	2.1	**2.1**	1.9	**1.9**			3.2	**3.2**			-1.5	
TSPYL2	TSPY-like 2	1.3		1.6	**1.6**	2.4	**2.4**						
TSR1	TSR1, 20S rRNA accumulation, homolog (S. cerevisiae)	-2.0	**-2.0**	-1.6		-2.0	**-2.0**	-2.0	**-2.0**	-2.4	**-2.4**	-1.8	**-1.8**
TSTA3	tissue specific transplantation antigen P35B					1.3				1.6	**1.6**	-1.3	
TTC1	tetratricopeptide repeat domain 1												
TTC3	tetratricopeptide repeat domain 3	-1.4		-1.2		-1.2		2.3	**2.3**	-1.6	**-1.6**	-5.1	**-5.1**
TTF1	transcription termination factor, RNA polymerase I			1.6				-2.7	**-2.7**	-1.3	**-1.3**	1.8	**1.8**
TTF2	transcription termination factor, RNA polymerase II			-1.4	**-1.4**	-1.2		-1.2	**-1.2**			1.2	
TTK	TTK protein kinase			-1.4		-1.2				1.3	**1.3**		
TTLL12	tubulin tyrosine ligase-like family, member 12	-1.4	**-1.4**	-1.4	**-1.4**	-1.3				-2.7	**-2.7**	-4.9	**-4.9**
TTN	titin									-1.4	**-1.4**	-8.8	**-8.8**
TTRAP	TRAF and TNF receptor associated protein			1.3									
TUB	tubby homolog (mouse)												
TUBA1	tubulin, alpha 1	10.4	**10.4**	8.9	**8.9**	3.0	**3.0**	-4.4	**-4.4**	2.1	**2.1**	13.5	**13.5**
TUBA2	tubulin, alpha 2	1.2		1.3	**1.3**	3.6	**3.6**	-1.4					
TUBA3	tubulin, alpha 3					-1.6	**-1.6**	-10.6	**-10.6**	1.7	**1.7**	15.1	**15.1**
TUBB	tubulin, beta	-1.4				1.3		-1.6	**-1.6**				
TUBB2A	tubulin, beta 2A					-1.4		-25.5	**-25.5**	3.2	**3.2**	54.6	**54.6**
TUBB2B	tubulin, beta 2B			-1.3				-1.2		1.8		1.3	
TUBB2C	tubulin, beta 2C							-1.3	**-1.3**	1.4	**1.4**		
TUBB3	tubulin, beta 3							-1.8	**-1.8**				
TUBG1	tubulin, gamma 1					-1.4	**-1.4**					-3.2	**-3.2**
TUBGCP2	tubulin, gamma complex associated protein 2					1.6	**-1.4**			-1.3	**-1.3**	-1.5	**-1.5**
TUBGCP3	tubulin, gamma complex associated protein 3							-1.4	**-1.4**	-1.3		-1.7	**-1.7**
TUFM	Tu translation elongation factor, mitochondrial	-1.2		-1.4	**-1.4**	-1.3	**-1.3**	-1.5	**-1.5**	-1.5	**-1.5**		
TUSC3	tumor suppressor candidate 3			-1.9									
TWF1	twinfilin, actin-binding protein, homolog 1 (Drosophila)												
TXK	TXK tyrosine kinase					1.6							
TXLNA	taxilin alpha	-1.3		-1.4	**-1.4**			1.2	**1.2**			-2.0	**-2.0**
TXN	thioredoxin			1.6	**1.6**	1.7	**1.7**	1.8	**1.8**	1.3	**1.3**	-1.5	**-1.5**
TXN2	thioredoxin 2	-1.3						-1.4	**-1.4**			-1.3	
TXNDC1	thioredoxin domain containing 1			-1.3						-1.6	**-1.6**	-2.0	**-2.0**
TXNDC9	thioredoxin domain containing 9					-1.3		1.5	**1.5**			-1.2	**-1.2**
TXNIP	thioredoxin interacting protein	2.8	**2.8**	3.4	**3.4**	3.7	**3.7**	10.0	**10.0**	7.7	**7.7**	2.1	**2.1**
TXNL1	thioredoxin-like 1			-1.2		-1.4		1.3	**1.3**	-2.1	**-2.1**	-1.4	**-1.4**
TXNRD1	thioredoxin reductase 1	-1.3		-1.4	**-1.4**	-1.4	**-1.4**	-1.3	**-1.3**	-1.3			
TYK2	tyrosine kinase 2	1.4				1.2							
TYMS	thymidylate synthetase							2.1	**2.1**	-1.2	**-1.2**	-4.8	**-4.8**
TYRO3	TYRO3 protein tyrosine kinase	-1.2	**-1.2**			-1.7	**-1.7**	-1.9				1.8	

U2AF1	U2 small nuclear RNA auxiliary factor 1									-1.3		-1.3	
U2AF2	U2 small nuclear RNA auxiliary factor 2	-1.5		-1.3		-1.3		-1.3	**-1.3**				
UAP1	UDP-N-acteylglucosamine pyrophosphorylase 1	-1.3	**-1.3**	-1.6	**-1.6**	-1.5	**-1.5**	1.8	**1.8**	-2.3	**-2.3**	-3.9	**-3.9**
UBA2	SUMO1 activating enzyme subunit 2					-1.4				-1.6	**-1.6**	-1.9	**-1.9**
UBB	ubiquitin B							-1.6	**-1.6**	1.7	**1.7**	1.5	**1.5**
UBC	ubiquitin C	-1.2		-1.4						1.6	**1.6**	1.5	**1.5**
UBE1	ubiquitin-activating enzyme E1 (A1S9T and BN75 temperature sensitivity complementing)							-1.3	**-1.3**	-1.4	**-1.4**	1.2	
UBE1C	ubiquitin-activating enzyme E1C (UBA3 homolog, yeast)									-1.2	**1.2**	1.5	**1.5**
UBE1L	ubiquitin-activating enzyme E1-like			1.2				-1.5	**-1.5**	-1.4	**-1.4**	1.8	**1.8**
UBE2A	ubiquitin-conjugating enzyme E2A (RAD6 homolog)					-1.3				1.4	**1.4**		
UBE2C	ubiquitin-conjugating enzyme E2C							1.3	**1.3**	1.7	**1.7**		
UBE2D1	ubiquitin-conjugating enzyme E2D 1 (UBC4/5 homolog, yeast)					-1.4		-2.2	**-1.3**	1.3		1.3	**1.3**
UBE2D2	ubiquitin-conjugating enzyme E2D 2 (UBC4/5 homolog, yeast)							-1.9	**-1.9**	1.6	**1.6**	4.1	**4.1**
UBE2D3	ubiquitin-conjugating enzyme E2D 3 (UBC4/5 homolog, yeast)			1.2		-1.3	**-1.3**	-1.3	**-1.3**	1.3	**1.3**	1.6	**1.6**
UBE2E1	ubiquitin-conjugating enzyme E2E 1 (UBC4/5 homolog, yeast)					-1.4		1.4	**1.4**			-1.8	**-1.8**
UBE2E3	ubiquitin-conjugating enzyme E2E 3 (UBC4/5 homolog, yeast)							2.2	**2.2**	1.2		-2.1	**-2.1**
UBE2G1	ubiquitin-conjugating enzyme E2G 1 (UBC7 homolog, yeast)	-1.3		1.6	**1.6**	-1.5		-1.6	**-1.6**				
UBE2G2	ubiquitin-conjugating enzyme E2G 2 (UBC7 homolog, yeast)			-1.2				1.2		-1.9	**-1.9**	-3.2	**-3.2**
UBE2I	ubiquitin-conjugating enzyme E2I (UBC9 homolog, yeast)	-1.2		-1.2	**-1.2**	1.4		-4.5	**-4.5**	-1.2	**-1.2**	2.8	**2.8**
UBE2J1	ubiquitin-conjugating enzyme E2, J1 (UBC6 homolog, yeast)					-1.4		1.5	**1.5**	1.5	**1.5**	-1.5	**-1.5**
UBE2L3	ubiquitin-conjugating enzyme E2L 3					-1.3		1.5	**1.5**	-2.1	**-2.1**	-2.1	**-2.1**
UBE2L6	ubiquitin-conjugating enzyme E2L 6	-1.2	**-1.2**			-1.3	**-1.3**	-2.0	**-2.0**	-1.7	**-1.7**		
UBE2M	ubiquitin-conjugating enzyme E2M (UBC12 homolog, yeast)	-1.4	**-1.4**	-1.3	**-1.3**	-1.3						-2.6	**-2.6**
UBE2N	ubiquitin-conjugating enzyme E2N (UBC13 homolog, yeast)	-1.2				-1.2		-1.4	**-1.4**	-1.3	**-1.3**		
UBE2S	ubiquitin-conjugating enzyme E2S	-1.6		-1.6	**-1.6**	-1.3		-1.2		-2.1	**-2.1**		
UBE2V2	ubiquitin-conjugating enzyme E2 variant 2							-1.4	**-1.4**	-1.3	**-1.3**	-1.7	**-1.7**
UBE3A	ubiquitin protein ligase E3A (human papilloma virus E6-associated protein, Angelman syndrome)	-1.4	**-1.4**					-5.8	**-5.8**	-1.5	**1.4**	8.6	**8.6**
UBE3B	ubiquitin protein ligase E3B									-1.4			
UBE3C	ubiquitin protein ligase E3C							-1.3	**-1.3**	1.4		1.3	
UBE4A	ubiquitination factor E4A (UFD2 homolog, yeast)			1.5		-1.4						1.3	**1.3**
UBE4B	ubiquitination factor E4B (UFD2 homolog, yeast)			1.3		1.3		1.3	**1.3**			-1.3	**-1.3**
UBN1	ubinuclein 1							-1.8	**-1.8**	-1.2	**-1.2**	1.3	**1.3**
UBQLN2	ubiquilin 2					-1.2							
UBR2	ubiquitin protein ligase E3 component n-recognin 2	1.2										1.3	**1.3**
UBTF	upstream binding transcription factor, RNA polymerase I	-1.3	**-1.3**	-1.3	**-1.3**	-1.3		-1.4	**-1.2**	-1.3	**-1.3**	-1.4	**-1.4**
UCHL3	ubiquitin carboxyl-terminal esterase L3 (ubiquitin thiolesterase)	-1.4		-1.5	**-1.5**	-1.5				-1.7	**-1.7**	-2.8	**-2.8**
UCK2	uridine-cytidine kinase 2			-1.3				-1.7	**-1.7**	-1.4	**-1.4**	-3.0	**-3.0**
UCKL1	uridine-cytidine kinase 1-like 1									2.1	**2.1**	1.7	**1.7**
UCP2	uncoupling protein 2 (mitochondrial, proton carrier)	1.2				1.4		1.3	**1.3**				
UFD1L	ubiquitin fusion degradation 1 like (yeast)					-1.2				-1.4	**-1.4**	-2.3	**-2.3**
UGCG	UDP-glucose ceramide glucosyltransferase			1.6	**1.6**			-2.4	**-2.4**	1.2	**1.2**	3.0	**3.0**
UGDH	UDP-glucose dehydrogenase	1.3				1.3				-1.5	**-1.5**		
UGP2	UDP-glucose pyrophosphorylase 2	1.6	**1.6**	1.4						1.3	**1.3**		
ULK1	unc-51-like kinase 1 (C. elegans)	1.4		1.7	**1.7**	1.3						3.8	**3.8**
UMPS	uridine monophosphate synthetase (orotate phosphoribosyl transferase and orotidine-5'-decarboxylase)	-1.3	**-1.3**			-1.4		-1.3	**-1.3**	-1.3		-1.6	**-1.6**
UNC119	unc-119 homolog (C. elegans)												
UNC5B	unc-5 homolog B (C. elegans)	1.3				1.5							
UNC84A	unc-84 homolog A (C. elegans)			-1.3		-1.2				1.2	**1.2**	-1.3	
UNG	uracil-DNA glycosylase			-1.4	**-1.4**	-1.4				1.5		-10.0	**-10.0**
UPF2	UPF2 regulator of nonsense transcripts homolog (yeast)							2.2	**2.2**			-2.0	**-2.0**
UPF3A	UPF3 regulator of nonsense transcripts homolog A (yeast)							-1.4	**-1.4**			1.4	**1.4**
UQCR	ubiquinol-cytochrome c reductase, 6.4kDa subunit							-1.3	**-1.3**			-1.2	
UQCRB	ubiquinol-cytochrome c reductase binding protein			1.2	**1.2**							1.3	**1.3**
UQCRC1	ubiquinol-cytochrome c reductase core protein I									-2.0	**-2.0**	-1.3	**-1.3**
UQCRC2	ubiquinol-cytochrome c reductase core protein II	-1.2		-1.7	**-1.7**	-1.5		-1.6	**-1.6**	-1.2		1.8	**1.8**
UQCRFS1	ubiquinol-cytochrome c reductase, Rieske iron-sulfur polypeptide 1					-1.3		1.2	**1.2**			-1.6	**-1.6**
UQCRH	ubiquinol-cytochrome c reductase hinge protein							1.5	**1.5**			-1.9	**-1.9**
UROD	uroporphyrinogen decarboxylase							2.2	**2.2**			-2.4	**-2.4**

UROS	uroporphyrinogen III synthase (congenital erythropoietic porphyria)					-1.2		2.4	**2.4**	1.6	**1.6**	-6.2	**-6.2**
USF2	upstream transcription factor 2, c-fos interacting							-1.5	**-1.5**			1.6	**1.6**
USP1	ubiquitin specific peptidase 1					-1.4	**-1.4**	-1.5	**-1.5**				
USP10	ubiquitin specific peptidase 10			-1.5		-1.7				-1.7	**-1.7**	-1.6	**-1.6**
USP11	ubiquitin specific peptidase 11					1.3		-1.6	**-1.6**	1.3	**1.3**	1.4	**1.4**
USP12	ubiquitin specific peptidase 12							-2.0	**-2.0**	2.3	**2.3**	3.7	**3.7**
USP14	ubiquitin specific peptidase 14 (tRNA-guanine transglycosylase)			-1.4	**-1.4**			1.3	**1.3**	-1.3	**-1.3**	-1.7	**-1.7**
USP19	ubiquitin specific peptidase 19												
USP20	ubiquitin specific peptidase 20	1.5	**1.5**	1.3	**1.3**	2.1	**2.1**						
USP22	ubiquitin specific peptidase 22					1.4		-2.6	**-2.6**			2.7	**2.7**
USP24	ubiquitin specific peptidase 24							1.4	**1.4**	1.3	**1.3**	-1.3	**-1.3**
USP32	ubiquitin specific peptidase 32							3.1	**3.1**	1.3		-2.2	**-2.2**
USP33	ubiquitin specific peptidase 33			1.3				1.5	**1.5**	1.3	**1.3**		
USP34	ubiquitin specific peptidase 34			1.4				-1.3	**1.3**	1.8	**1.8**	2.6	**2.6**
USP4	ubiquitin specific peptidase 4 (proto-oncogene)			1.3				-1.7	**-1.7**	1.3	**1.3**	2.4	**2.4**
USP46	ubiquitin specific peptidase 46	-1.3		-1.4				3.5	**3.5**	1.5		-3.0	**-3.0**
USP5	ubiquitin specific peptidase 5 (isopeptidase T)												
USP52	ubiquitin specific peptidase 52	1.4		1.4	**1.4**					1.4		1.5	**1.5**
USP6	ubiquitin specific peptidase 6 (Tre-2 oncogene)							1.4	**1.4**	-1.3	**-1.3**		
USP6NL	USP6 N-terminal like					1.5	**1.5**			2.1	**2.1**	1.4	
USP7	ubiquitin specific peptidase 7 (herpes virus-associated)							-3.0	**-3.0**			2.6	**2.6**
USP8	ubiquitin specific peptidase 8			1.3						1.5	**1.5**	1.5	**1.5**
USP9X	ubiquitin specific peptidase 9, X-linked			1.5		3.4				1.4	**1.4**	1.3	**1.3**
UTRN	utrophin	1.7		2.9	**2.9**	2.5		-1.4	**-1.4**	1.6	**1.6**	2.0	**2.0**
UTX	ubiquitously transcribed tetratricopeptide repeat, X chromosome					-1.3		-1.6	**-1.6**	1.4	**1.4**	2.9	**2.9**
UVRAG	UV radiation resistance associated gene			1.4	**1.4**	1.5	**1.5**	1.6	**1.6**	-1.3			
VAMP1	vesicle-associated membrane protein 1 (synaptobrevin 1)	1.3								1.3	**1.3**	2.1	**2.1**
VAMP2	vesicle-associated membrane protein 2 (synaptobrevin 2)	1.3						-4.2	**-4.2**	-1.3	**-1.3**	4.9	**4.9**
VAMP3	vesicle-associated membrane protein 3 (cellubrevin)			1.5				1.3	**1.3**	1.3	**1.3**	1.5	**1.5**
VAMP4	vesicle-associated membrane protein 4	1.4				-1.4		-2.6	**-2.6**	1.3		2.7	**2.7**
VAMP8	vesicle-associated membrane protein 8 (endobrevin)	-1.3		-1.3	**-1.3**	-1.2		2.0	**2.0**			-4.1	**-4.1**
VAPA	VAMP (vesicle-associated membrane protein)-associated protein A, 33kDa					-1.3		1.7	**1.3**	-1.4		-2.9	**-1.7**
VAPB	VAMP (vesicle-associated membrane protein)-associated protein B and C							1.4		1.2		-1.9	**-1.9**
VARS	valyl-tRNA synthetase	-1.5		-1.3		-1.3		-1.3		-2.1	**-2.1**	-2.1	**-2.1**
VASP	vasodilator-stimulated phosphoprotein	1.3						1.8	**1.8**	-1.3	**-1.3**	-1.6	**-1.6**
VAV1	vav 1 oncogene			-1.3								-1.2	**-1.2**
VBP1	von Hippel-Lindau binding protein 1			-1.3	**-1.3**	-1.3				1.2		-1.9	**-1.9**
VCL	vinculin	1.8		1.6	**1.6**	1.9	**1.9**	1.9	**1.9**	-1.5	**-1.5**	-1.4	**-1.4**
VCP	valosin-containing protein	-1.4						1.3	**1.3**	-1.3	**-1.3**	-1.4	**-1.4**
VDAC1	voltage-dependent anion channel 1	-1.6	**-1.6**	-1.5	**-1.5**	-1.7	**-1.7**	1.6	**1.6**			-3.1	**-3.1**
VDAC2	voltage-dependent anion channel 2	-1.3		-1.4	**-1.4**	-1.5	**-1.5**	1.9	**1.9**			-2.1	**-2.1**
VDAC3	voltage-dependent anion channel 3					-1.3		1.8	**1.8**			-1.9	**-1.9**
VDP	vesicle docking protein p115							2.0	**2.0**	1.6		-1.6	**-1.6**
VEGF	vascular endothelial growth factor A	-1.5	**-1.5**	-1.6	**-1.6**			8.2	**8.2**	-2.4	**-2.4**	-13.8	**-13.8**
VEGFB	vascular endothelial growth factor B	-1.5		-2.3	**-2.3**			-2.5	**-2.5**	-1.5		1.6	
VGLL4	vestigial like 4 (Drosophila)							1.4	**1.3**			-1.2	
VHL	von Hippel-Lindau tumor suppressor			1.8									
VIL2	villin 2 (ezrin)					-1.2		-2.2	**-2.2**	2.4	**2.4**	9.8	**9.8**
VIM	vimentin			-1.2				-275.9	**-275.9**	1.2	**1.2**	360.4	**360.4**
VKORC1	vitamin K epoxide reductase complex, subunit 1	1.4											
VLDLR	very low density lipoprotein receptor	-2.2		-1.4	**-1.4**			-4.7	**-4.7**	-1.2	**-1.2**	2.6	**2.6**
VPS11	vacuolar protein sorting 11 homolog (S. cerevisiae)			1.2		1.2		1.6	**1.6**	-1.2			
VPS13A	vacuolar protein sorting 13 homolog A (S. cerevisiae)			-1.9		-1.3	**-1.3**	3.7	**3.7**	-1.4		-2.8	**-2.8**
VPS26	vacuolar protein sorting 26 homolog A (yeast)	1.3				1.2		1.5	**1.5**	1.3	**1.3**		
VPS45A	vacuolar protein sorting 45 homolog (S. cerevisiae)	1.2						1.4				-1.3	
VPS4B	vacuolar protein sorting 4 homolog B (S. cerevisiae)					-1.4						1.3	**1.3**
VPS52	vacuolar protein sorting 52 homolog (S. cerevisiae)											1.3	**1.3**
VRK1	vaccinia related kinase 1			-1.2	**-1.2**							-1.7	**-1.7**

VTI1B	vesicle transport through interaction with t-SNAREs homolog 1B (yeast)			-1.3				1.7	**1.7**	1.3	**1.3**	-1.7	**-1.7**
WARS	tryptophanyl-tRNA synthetase			1.2				-1.5	**-1.5**	-3.3	**-3.3**	-1.8	**-1.8**
WAS	Wiskott-Aldrich syndrome (eczema-thrombocytopenia)					2.7						3.4	**3.4**
WASF1	WAS protein family, member 1											-1.3	**-1.3**
WBP2	WW domain binding protein 2			-1.2									
WBP4	WW domain binding protein 4 (formin binding protein 21)	-1.2				-1.3		-1.2	**-1.2**	1.5		1.5	**1.5**
WBSCR1	eukaryotic translation initiation factor 4H							-2.4	**-2.4**			1.5	**1.5**
WDHD1	WD repeat and HMG-box DNA binding protein 1									-1.6		-5.6	**-5.6**
WDR1	WD repeat domain 1					-1.5		-2.5	**-2.5**	1.2		2.8	**2.8**
WDR23	WD repeat domain 23												
WDR39	cytosolic iron-sulfur protein assembly 1 homolog (S. cerevisiae)									-1.9	**-1.9**	-2.3	**-2.3**
WDR46	WD repeat domain 46	-1.5				-1.4						-1.6	**-1.6**
WDR57	WD repeat domain 57 (U5 snRNP specific)												
WEE1	WEE1 homolog (S. pombe)			2.5	**2.5**			-1.4	**-1.4**	1.2	**1.2**		
WFS1	Wolfram syndrome 1 (wolframin)	2.6	**2.6**	2.2	**2.2**	2.8	**2.8**						
WHSC2	Wolf-Hirschhorn syndrome candidate 2			-1.6	**-1.6**	-1.3		-1.2				-1.4	**-1.4**
WIT-1	Wilms tumor upstream neighbor 1							-2.4	**-2.4**			2.5	**2.5**
WIZ	WIZ zinc finger							-1.3				1.3	
WNK1	WNK lysine deficient protein kinase 1			1.5				-1.5				4.2	**4.2**
WRB	tryptophan rich basic protein	-1.3										1.2	**1.2**
WRN	Werner syndrome	-1.8	**-1.8**					1.2		-1.6	**-1.6**	-1.7	**-1.7**
WSB1	WD repeat and SOCS box-containing 1			1.2		-1.3		9.0	**9.0**	2.0	**2.0**	-2.0	**-2.0**
WSB2	WD repeat and SOCS box-containing 2					-1.2		1.4	**1.4**	1.3	**1.3**	-1.7	**-1.7**
WT1	Wilms tumor 1	-1.5	**-1.5**	-1.3	**-1.3**	-1.8	**-1.8**	-1.6	**-1.6**	1.4	**1.4**	-1.7	**-1.7**
WTAP	Wilms tumor 1 associated protein	1.2						-1.5	**-1.5**	1.7	**1.7**	1.8	**1.8**
WWOX	WW domain containing oxidoreductase			1.2				-1.4	**-1.4**	-1.7	**-1.7**	-1.4	
WWP1	WW domain containing E3 ubiquitin protein ligase 1	-1.3		1.3		-1.3		-1.2		1.6	**1.6**	2.1	**2.1**
XAB1	XPA binding protein 1, GTPase							-1.6	**-1.6**			1.6	**1.6**
XBP1	X-box binding protein 1					-1.3				1.4	**1.4**	1.4	**1.4**
XDH	xanthine dehydrogenase									-2.2	**-2.2**		
XIST	X (inactive)-specific transcript			1.6				-808.5	**-808.5**	-1.5		1094.1	**1094.1**
XPA	xeroderma pigmentosum, complementation group A			1.3						-1.3		-1.2	
XPC	xeroderma pigmentosum, complementation group C												
XPNPEP1	X-prolyl aminopeptidase (aminopeptidase P) 1, soluble							3.2	**3.2**			-3.9	**-3.9**
XPO1	exportin 1 (CRM1 homolog, yeast)					-1.3		-1.4	**-1.4**	1.5		4.3	**4.3**
XPO7	exportin 7			-1.2				4.8	**4.8**	1.3	**1.3**	-2.7	**-2.7**
XPOT	exportin, tRNA (nuclear export receptor for tRNAs)	-1.4		-1.8	**-1.8**	-1.5		1.6	**1.6**	-3.2	**-3.2**	-6.4	**-6.4**
XRCC2	X-ray repair complementing defective repair in Chinese hamster cells 2			-1.2				-1.2				1.5	
XRCC3	X-ray repair complementing defective repair in Chinese hamster cells 3			-1.8		-1.6		-1.7	**-1.7**	-1.3			
XRCC4	X-ray repair complementing defective repair in Chinese hamster cells 4			-1.2				-1.8	**-1.8**	1.4	**1.4**	1.6	**1.6**
XRCC5	X-ray repair complementing defective repair in Chinese hamster cells 5 (double-strand-break rejoining; Ku autoantigen, 80kDa)			-1.3		-1.2		-2.0	**-2.0**	-1.7	**-1.7**	1.3	
XYLT1	xylosyltransferase I												
YAF2	YY1 associated factor 2	1.8	**1.8**	2.4	**2.4**	1.6	**1.6**	1.7	**1.7**	1.9	**1.9**	5.8	**5.8**
YARS	tyrosyl-tRNA synthetase	-1.6		-1.5	**-1.5**	-1.3		-1.9	**-1.9**	-2.6	**-2.6**	-2.4	**-2.4**
YES1	v-yes-1 Yamaguchi sarcoma viral oncogene homolog 1					1.3	**1.3**	49.1	**49.1**			-63.8	**-63.8**
YIF1	Yip1 interacting factor homolog A (S. cerevisiae)			-1.3	**-1.3**					-1.5	**-1.5**	-2.1	**-2.1**
YME1L1	YME1-like 1 (S. cerevisiae)					-1.2		1.7	**1.7**	-1.2	**-1.2**	-1.7	**-1.7**
YTHDC1	YTH domain containing 1												
YWHAB	tyrosine 3-monooxygenase/tryptophan 5-monooxygenase activation protein, beta polypeptide							1.2				-1.5	**-1.5**
YWHAE	tyrosine 3-monooxygenase/tryptophan 5-monooxygenase activation protein, epsilon polypeptide			-1.4				-2.5	**-2.5**	-1.6	**-1.6**	2.2	**2.2**
YWHAH	tyrosine 3-monooxygenase/tryptophan 5-monooxygenase activation protein, eta polypeptide											1.6	**1.6**
YWHAQ	tyrosine 3-monooxygenase/tryptophan 5-monooxygenase activation protein, theta polypeptide							-1.7	**-1.7**	1.6	**1.6**	1.5	**1.5**
YWHAZ	tyrosine 3-monooxygenase/tryptophan 5-monooxygenase activation protein, zeta polypeptide			1.9		1.8		-2.5	**-2.5**	1.7	**1.7**	3.9	**3.9**
YY1	YY1 transcription factor	-1.4		-1.7				2.7	**2.7**			-2.7	**-2.7**
ZA20D2	zinc finger, AN1-type domain 5			1.4				-1.6		1.9	**1.9**	1.5	**1.5**
ZAP70	zeta-chain (TCR) associated protein kinase 70kDa					-1.3	**-1.3**	-10.1	**-10.1**			13.8	**13.8**
ZBED4	zinc finger, BED-type containing 4							-1.4				1.3	
ZBTB1	zinc finger and BTB domain containing 1							-1.4	**-1.4**			1.6	**1.6**

ZBTB11	zinc finger and BTB domain containing 11									-1.3	**-1.3**	-1.6	**-1.6**
ZBTB16	zinc finger and BTB domain containing 16					7.0		5.9	**5.9**	2.8	**2.8**	8.3	**8.3**
ZBTB17	zinc finger and BTB domain containing 17											1.3	
ZBTB33	zinc finger and BTB domain containing 33			2.0									
ZBTB5	zinc finger and BTB domain containing 5	-1.2										-1.4	**-1.4**
ZC3H13	zinc finger CCCH-type containing 13												
ZC3HAV1	zinc finger CCCH-type, antiviral 1							-1.7	**-1.7**	1.4	**1.4**	2.6	**2.6**
ZDHHC17	zinc finger, DHHC-type containing 17									1.3		1.2	**1.2**
ZFP161	zinc finger protein 161 homolog (mouse)												
ZFP36	zinc finger protein 36, C3H type, homolog (mouse)			1.7	**1.7**	1.5		-1.3		3.2	**3.2**	4.1	**4.1**
ZFP36L1	zinc finger protein 36, C3H type-like 1			-1.4				-24.5	**-24.5**			11.5	**11.5**
ZFP36L2	zinc finger protein 36, C3H type-like 2	3.0	**3.0**	2.5	**2.5**	2.5	**2.5**	2.2	**2.2**	1.8	**1.8**		
ZFX	zinc finger protein, X-linked	1.8		1.3		1.3		-1.6	**-1.6**	1.4		2.8	**1.7**
ZFYVE16	zinc finger, FYVE domain containing 16							1.6		1.6	**1.6**	2.7	**2.7**
ZHX2	zinc fingers and homeoboxes 2	2.6		2.4	**2.4**			-5.1	**-5.1**	1.8	**1.3**	6.9	**6.9**
ZHX3	zinc fingers and homeoboxes 3	1.3		1.4	**1.4**	1.3		2.0	**2.0**	2.0	**2.0**	1.4	
ZMPSTE24	zinc metallopeptidase (STE24 homolog, yeast)			-1.4				1.4	**1.4**			-1.8	**-1.8**
ZMYM3	zinc finger, MYM-type 3							1.6		-1.3		-1.8	**-1.8**
ZMYND11	zinc finger, MYND domain containing 11	-1.4		1.5				2.6	**2.6**	1.5	**1.3**		
ZNF124	zinc finger protein 124							1.3		-1.4	**-1.4**	-1.7	**-1.7**
ZNF133	zinc finger protein 133					1.2						1.3	
ZNF143	zinc finger protein 143					-1.3		-1.4	**-1.4**				
ZNF148	zinc finger protein 148	1.3	**1.3**	1.2		-1.7		1.8	**1.8**	1.8	**1.8**	-1.6	**-1.6**
ZNF160	zinc finger protein 160							-1.7	**-1.7**	1.6	**1.5**	1.6	**1.6**
ZNF161	vascular endothelial zinc finger 1	1.3		1.2				-1.9	**-1.9**	1.4		2.2	**2.2**
ZNF175	zinc finger protein 175	1.2						2.9	**2.9**			-2.6	**-2.6**
ZNF183	--			1.2		-1.3		1.4	**1.4**	-2.0		-1.5	**-1.5**
ZNF198	zinc finger, MYM-type 2							4.3	**4.3**	1.3	**-1.3**	-2.5	**-2.5**
ZNF202	zinc finger protein 202	1.2		-2.1				-1.4	**-1.4**	-1.8	**-1.8**		
ZNF207	zinc finger protein 207							2.4	**2.4**	-1.4	**-1.4**		
ZNF212	zinc finger protein 212							-1.2		1.3		1.3	
ZNF217	zinc finger protein 217											1.3	**1.3**
ZNF22	zinc finger protein 22 (KOX 15)	-1.3				-1.4		-1.3	**-1.3**			1.3	**1.3**
ZNF238	zinc finger protein 238	-1.4		2.1		-1.3		-5.5	**-5.5**			6.3	**6.3**
ZNF24	zinc finger protein 24	-1.3		1.5						-1.5	**-1.2**	1.3	
ZNF259	zinc finger protein 259	-1.4	**-1.4**	-1.5		-1.8	**-1.8**	-1.2	**-1.2**	-1.3		-1.6	**-1.6**
ZNF263	zinc finger protein 263	-1.2		-1.3		-1.6		-1.7	**-1.7**	-1.7	**-1.7**	1.8	**1.8**
ZNF267	zinc finger protein 267			1.3				1.9	**1.9**			-1.9	**-1.9**
ZNF268	zinc finger protein 268							-1.2		-1.4		-1.8	**-1.8**
ZNF274	zinc finger protein 274	-1.6						1.8				-1.5	**-1.5**
ZNF278	POZ (BTB) and AT hook containing zinc finger 1	1.8	**1.5**	1.7	**1.7**	1.3		1.4		-1.4	**-1.4**	1.3	
ZNF291	zinc finger protein 291							1.4	**1.4**	1.6	**1.6**	2.1	**2.1**
ZNF318	zinc finger protein 318			1.3	**1.3**			2.3	**2.3**	1.3	**1.3**		
ZNF330	zinc finger protein 330			-1.5		-1.6	**-1.6**					-1.8	**-1.8**
ZNF354A	zinc finger protein 354A							2.6	**2.6**	1.2		-1.5	
ZNF364	zinc finger protein 364	1.2				1.2		1.3	**1.3**	1.8	**1.8**	3.0	**3.0**
ZNF384	zinc finger protein 384			1.2				-1.6	**-1.6**	-1.6	**-1.6**	1.4	
ZNF410	zinc finger protein 410	1.2	**1.2**							1.3			
ZNF42	myeloid zinc finger 1					1.2				-1.4	**-1.4**	-1.2	
ZNF423	zinc finger protein 423					-2.4		-3.6	**-3.6**	-2.2	**-2.2**	5.6	**5.6**
ZNF43	zinc finger protein 43							-1.4				1.6	
ZNF45	zinc finger protein 45	1.2		1.4									
ZNF451	zinc finger protein 451							2.5	**2.5**			-1.6	**-1.6**
ZNF592	zinc finger protein 592					1.2		-1.7	**-1.7**	-1.3	**1.3**	1.5	**1.4**
ZNF638	zinc finger protein 638	1.3						-1.3		1.3		1.4	
ZNF84	zinc finger protein 84							-1.2				-1.4	**-1.4**
ZNF9	CCHC-type zinc finger, nucleic acid binding protein	-1.5		-1.9	**-1.9**	-1.6				-1.4	**-1.4**	-1.5	**-1.5**
ZNF91	zinc finger protein 91	1.3		1.4	**1.4**			-2.2	**-2.2**	1.2		2.2	**2.2**

ZNF96	zinc finger protein 96							1.2				-1.9	**-1.9**
ZNFN1A1	IKAROS family zinc finger 1 (Ikaros)			2.0	**2.0**			2.2	**2.2**	1.6	**1.3**	2.2	**2.2**
ZNHIT3	zinc finger, HIT type 3					-1.4							
ZRF1	zuotin related factor 1	-1.6		-1.2		-1.6		1.4	**1.4**	-2.3	**-1.5**	-2.9	**-2.9**
ZRSR2	zinc finger (CCCH type), RNA-binding motif and serine/arginine rich 2							-1.3				1.8	**1.8**
ZUBR1	zinc finger, UBR1 type 1							2.0	**2.0**			-1.9	**-1.9**
ZW10	ZW10, kinetochore associated, homolog (Drosophila)	1.4								-1.4	**-1.4**	-1.3	
ZWINT	ZW10 interactor									-1.3	**-1.3**	-2.3	**-2.3**
ZWINTAS	ZW10 interactor antisense	1.2				1.5							
