# Supplementary material for: Gene expression profiling of leukemic cells and primary thymocytes predicts a signature for apoptotic sensitivity to glucocorticoids
Source: Cancer Cell Int. 2007 Nov 28;7:18. doi: 10.1186/1475-2867-7-18 (PMC2228275; doi:10.1186/1475-2867-7-18)
Supplement: Additional file 5 — Adult ALL vs. Pediatric ALL and AML. Genes statistically significantly regulated by Dex in adult B-cell ALL RS4;11 cells compared to other GC-sensitive pediatric leukemias. Blank = gene "absent" by selection criteria. [file 1475-2867-7-18-S5.pdf]

Additional file 5: Adult B-ALL vs. Pediatric B-ALL, T-ALL, and AML.						
Blank = "absent"						
GC-response		Sensitive	Sensitive	Sensitive	Sensitive	Sensitive
Patient-derived cell line		Adult	Pediatric	Pediatric	Pediatric	Pediatric
Cell lineage		B-cell	B-cell	T-cell	T-cell	Myeloid
Sub-type of leukemia		ALL	ALL	ALL	ALL	AML
Name	Description	RS4 Dx	SUP Dx	C7-14 Dx	C1-6 Dx	Kas Dx
		Stat. sign	Stat. sign	Stat. sign	Stat. sign	Stat. sign
76P	gamma tubulin ring complex protein (76p gene)	-1.9	-1.6			1.6
AARS	alanyl-tRNA synthetase	-2.0	-1.9	-1.3		-1.5
AASDHPPT	aminoadipate-semialdehyde dehydrogenase-phosphopantetheinyl transferase	-1.4	-1.3			
AATF	apoptosis antagonizing transcription factor	-1.3			-1.4	
ABAT	4-aminobutyrate aminotransferase	-21.9				14.5
ABCB6	ATP-binding cassette, sub-family B (MDR/TAP), member 6	-1.6		1.3		3.4
ABCB7	ATP-binding cassette, sub-family B (MDR/TAP), member 7	1.3				
ABCC1	ATP-binding cassette, sub-family C (CFTR/MRP), member 1	1.9	-2.0		1.5	-3.3
ABCC5	ATP-binding cassette, sub-family C (CFTR/MRP), member 5	-1.4	-1.8	2.1		
ABCE1	ATP-binding cassette, sub-family E (OABP), member 1	1.4	-2.0	-1.5		-4.2
ABI1	abl-interactor 1	1.4	1.7			2.8
ABI2	abl interactor 2	1.3	-1.9			-2.7
ABLIM1	actin binding LIM protein 1	-5.8	-3.1			
ACADM	acyl-Coenzyme A dehydrogenase, C-4 to C-12 straight chain	-1.8				1.7
ACAT2	acetyl-Coenzyme A acetyltransferase 2 (acetoacetyl Coenzyme A thiolase)	-2.4	-1.5			
ACBD3	acyl-Coenzyme A binding domain containing 3	-2.0	1.4			1.5
ACD	adrenocortical dysplasia homolog (mouse)	-1.3				
ACLY	ATP citrate lyase	-1.4	-1.7			-1.3
ACO1	aconitase 1, soluble	1.4		1.3	1.4	-1.2
ACOT2	acyl-CoA thioesterase 2	5.0				-7.8
ACOT7	acyl-CoA thioesterase 7	-1.6	-1.4			-1.3
ACOX1	acyl-Coenzyme A oxidase 1, palmitoyl	5.2				-3.5
ACP1	acid phosphatase 1, soluble	-2.1				-1.8
ACSL3	acyl-CoA synthetase long-chain family member 3	-1.8	-1.3			2.2
ACSL4	acyl-CoA synthetase long-chain family member 4	-1.7				
ACTB	actin, beta	-1.5				1.6
ACTL6A	actin-like 6A	-1.5	-1.3			-1.6
ACTN1	actinin, alpha 1	5.4	-1.2			-3.5
ACTR2	ARP2 actin-related protein 2 homolog (yeast)	-1.4	1.3	1.2		1.6
ACTR3	ARP3 actin-related protein 3 homolog (yeast)	2.0				-1.6
ACVR1B	activin A receptor, type IB	1.7				-1.5
ACY1	aminoacylase 1	1.7				-4.3
ADA	adenosine deaminase	-9.1	1.3	-1.3	-1.5	3.4
ADAM10	ADAM metallopeptidase domain 10	-3.0	2.1			2.8

ADAM17	ADAM metallopeptidase domain 17 (tumor necrosis factor, alpha, converting enzyme)	**-1.7**	**1.5**			**1.8**
ADAM9	ADAM metallopeptidase domain 9 (meltrin gamma)	**2.4**	**1.6**			**2.1**
ADARB1	adenosine deaminase, RNA-specific, B1 (RED1 homolog rat)	**-34.5**				**1.7**
ADCY7	adenylate cyclase 7	**-3.1**	**-1.5**			**3.7**
ADD1	adducin 1 (alpha)	**1.2**	**1.8**			**2.0**
ADFP	adipose differentiation-related protein	**3.3**	**1.5**			**-2.0**
ADH5	alcohol dehydrogenase 5 (class III), chi polypeptide	**2.4**				**-2.1**
ADIPOR2	adiponectin receptor 2	**-1.5**	**1.9**			
ADK	adenosine kinase	**1.6**	**-2.0**			**-4.1**
ADNP	activity-dependent neuroprotector	**-1.4**	**-1.2**			**1.5**
ADRBK1	adrenergic, beta, receptor kinase 1	**-2.0**				**-1.3**
ADRBK2	adrenergic, beta, receptor kinase 2	**1.6**	**-1.5**			**-2.2**
ADRM1	adhesion regulating molecule 1	**2.2**				**-3.5**
ADSL	adenylosuccinate lyase	**1.5**	**-1.5**			**-2.4**
AEBP1	AE binding protein 1	**-1.6**	**1.5**			**2.1**
AES	amino-terminal enhancer of split	**-2.1**		**1.2**		**3.0**
AFF1	AF4/FMR2 family, member 1	**-3.5**	**2.8**			**8.6**
AFG3L2	AFG3 ATPase family gene 3-like 2 (yeast)	**1.3**		**-1.3**		**-2.0**
AGA	aspartylglucosaminidase	**-1.3**				**-1.7**
AGGF1	angiogenic factor with G patch and FHA domains 1	**1.8**				**-1.8**
AGL	amylo-1, 6-glucosidase, 4-alpha-glucanotransferase (glycogen debranching enzyme, glycogen storage disease type III)	**-2.0**				**3.3**
AGPAT1	1-acylglycerol-3-phosphate O-acyltransferase 1 (lysophosphatidic acid acyltransferase, alpha)	**1.8**	**-1.4**	**1.4**		**1.3**
AGPAT2	1-acylglycerol-3-phosphate O-acyltransferase 2 (lysophosphatidic acid acyltransferase, beta)	**1.5**				**-1.4**
AGPS	alkylglycerone phosphate synthase	**5.5**	**5.0**			**-2.6**
AGRN	agrin	**-1.6**	**-2.8**			
AHCY	S-adenosylhomocysteine hydrolase	**1.4**	**-1.4**			**-2.7**
AHCYL1	S-adenosylhomocysteine hydrolase-like 1	**1.6**	**-1.4**			**-1.6**
AIF1	allograft inflammatory factor 1	**2.5**				**-39.1**
AIP	aryl hydrocarbon receptor interacting protein	**-1.4**				
AK2	adenylate kinase 2	**-1.4**	**-2.2**		**-1.6**	**-3.3**
AKAP1	A kinase (PRKA) anchor protein 1	**-1.8**	**-2.4**	**-1.6**		**-2.2**
AKAP10	A kinase (PRKA) anchor protein 10	**-2.7**				**2.9**
AKAP11	A kinase (PRKA) anchor protein 11	**-1.2**	**-1.3**			**-1.5**
AKR1A1	aldo-keto reductase family 1, member A1 (aldehyde reductase)	**-2.6**	**-2.3**			
AKR1B1	aldo-keto reductase family 1, member B1 (aldose reductase)	**-4.2**	**-1.2**			**8.1**
AKR7A2	aldo-keto reductase family 7, member A2 (aflatoxin aldehyde reductase)	**-1.6**				**-1.3**
AKT1	v-akt murine thymoma viral oncogene homolog 1	**-1.5**				**1.3**
ALAS1	aminolevulinate, delta-, synthase 1	**2.4**				**-1.9**
ALDH4A1	aldehyde dehydrogenase 4 family, member A1	**1.9**		**1.6**		**-3.5**
ALDH5A1	aldehyde dehydrogenase 5 family, member A1 (succinate-semialdehyde dehydrogenase)	**-1.5**	**-1.4**			**-2.2**
ALDH6A1	aldehyde dehydrogenase 6 family, member A1	**1.6**	**-1.7**		**1.6**	
ALDOA	aldolase A, fructose-bisphosphate	**-1.5**	**-1.3**			**-1.3**

ALS2CR3	trafficking protein, kinesin binding 2	**1.7**	**2.4**			**2.8**
AMD1	adenosylmethionine decarboxylase 1	**1.4**		**-1.4**		**-2.4**
AMPD2	adenosine monophosphate deaminase 2 (isoform L)	**1.9**	**-2.5**			**-2.9**
ANAPC10	anaphase promoting complex subunit 10	**1.8**				**-1.3**
ANAPC13	anaphase promoting complex subunit 13	**1.2**	**-1.3**			**-1.7**
ANAPC5	anaphase promoting complex subunit 5	**1.5**	**-1.8**			**-2.3**
ANGPT1	angiopoietin 1	**1.8**				**-8.8**
ANKRD28	ankyrin repeat domain 28	**2.2**	**2.2**		**-2.0**	**-4.1**
ANKRD40	ankyrin repeat domain 40	**-1.5**				**1.4**
ANP32A	acidic (leucine-rich) nuclear phosphoprotein 32 family, member A	**2.1**	**-1.3**			**-1.8**
ANXA11	annexin A11	**1.8**	**1.8**			**-1.2**
ANXA6	annexin A6	**2.4**			**1.3**	**-1.3**
ANXA7	annexin A7	**1.6**				**-1.3**
AP1B1	adaptor-related protein complex 1, beta 1 subunit	**-1.4**				**2.2**
AP1S2	adaptor-related protein complex 1, sigma 2 subunit	**-27.2**	**1.6**	**1.8**		**21.8**
AP3D1	adaptor-related protein complex 3, delta 1 subunit	**1.5**	**1.3**			
AP3S1	adaptor-related protein complex 3, sigma 1 subunit	**1.4**	**2.4**	**1.6**	**1.9**	**2.1**
AP3S2	adaptor-related protein complex 3, sigma 2 subunit	**2.2**				**-1.8**
APG12L	ATG12 autophagy related 12 homolog (S. cerevisiae)	**1.3**	**1.9**	**1.6**	**1.9**	**2.5**
API5	apoptosis inhibitor 5	**1.6**	**-2.1**			**-2.0**
APLP2	amyloid beta (A4) precursor-like protein 2	**4.2**	**-1.7**			**-12.0**
APOBEC3B	apolipoprotein B mRNA editing enzyme, catalytic polypeptide-like 3B	**-1.9**				**2.0**
APPBP1	amyloid beta precursor protein binding protein 1	**-1.2**	**-1.3**			**-1.2**
APPBP2	amyloid beta precursor protein (cytoplasmic tail) binding protein 2	**2.0**	**1.4**			**-1.3**
ARF1	ADP-ribosylation factor 1	**1.3**	**-1.2**			**-1.2**
ARF3	ADP-ribosylation factor 3	**1.4**	**-1.3**	**1.3**		**-1.3**
ARF4	ADP-ribosylation factor 4	**1.3**				**-1.6**
ARF5	ADP-ribosylation factor 5	**-2.4**				**2.0**
ARF6	ADP-ribosylation factor 6	**1.4**	**1.7**			**1.7**
ARFGEF1	ADP-ribosylation factor guanine nucleotide-exchange factor 1(brefeldin A-inhibited)	**-1.6**	**1.5**			**1.8**
ARFGEF2	ADP-ribosylation factor guanine nucleotide-exchange factor 2 (brefeldin A-inhibited)	**1.3**				**-1.2**
ARHGAP12	Rho GTPase activating protein 12	**1.7**			**-1.3**	
ARHGAP5	Rho GTPase activating protein 5	**-1.4**				**2.3**
ARHGDIA	Rho GDP dissociation inhibitor (GDI) alpha	**-1.3**				
ARHGDIB	Rho GDP dissociation inhibitor (GDI) beta	**-1.4**				**1.5**
ARHGEF18	rho/rac guanine nucleotide exchange factor (GEF) 18	**1.5**	**2.5**	**1.3**		**3.3**
ARHGEF2	rho/rac guanine nucleotide exchange factor (GEF) 2	**1.3**	**1.3**			**-1.7**
ARHGEF6	Rac/Cdc42 guanine nucleotide exchange factor (GEF) 6	**-1.4**	**-1.3**	**1.3**		
ARHGEF7	Rho guanine nucleotide exchange factor (GEF) 7	**-1.8**	**3.6**			**5.1**
ARID4A	AT rich interactive domain 4A (RBP1-like)	**2.9**				
ARID5B	AT rich interactive domain 5B (MRF1-like)	**-34.1**	**-2.2**			**8.6**
ARIH1	ariadne homolog, ubiquitin-conjugating enzyme E2 binding protein, 1 (Drosophila)	**1.7**	**1.6**			**1.8**

ARIH2	ariadne homolog 2 (Drosophila)	**-1.5**	**1.6**			**-2.4**
ARL1	ADP-ribosylation factor-like 1	**1.3**		**-1.2**		**-1.5**
ARL4A	ADP-ribosylation factor-like 4A	**26.8**				**-33.8**
ARL6IP	ADP-ribosylation factor-like 6 interacting protein 1	**-2.2**	**1.7**			**2.7**
ARPC1A	actin related protein 2/3 complex, subunit 1A, 41kDa	**-2.8**				**2.2**
ARPC1B	actin related protein 2/3 complex, subunit 1B, 41kDa	**1.3**			**-1.3**	
ARPC2	actin related protein 2/3 complex, subunit 2, 34kDa	**-1.3**				**1.4**
ARPC4	actin related protein 2/3 complex, subunit 4, 20kDa	**-4.7**				**4.1**
ARPC5	actin related protein 2/3 complex, subunit 5, 16kDa	**-1.4**	**1.4**			**2.3**
ARPP-19	cyclic AMP phosphoprotein, 19 kD	**-2.5**				**2.4**
ARRB2	arrestin, beta 2	**-2.1**				**1.3**
ASAH1	N-acylsphingosine amidohydrolase (acid ceramidase) 1	**4.4**	**-1.3**			**-3.8**
ASB1	ankyrin repeat and SOCS box-containing 1	**-1.8**				**1.4**
ASCC3L1	activating signal cointegrator 1 complex subunit 3-like 1	**-1.5**	**-1.4**			
ASK	DBF4 homolog (S. cerevisiae)	**-1.7**	**1.5**			
ASMTL	acetylserotonin O-methyltransferase-like	**-2.7**				**3.2**
ASNS	asparagine synthetase	**2.9**				**-9.0**
ASS	argininosuccinate synthetase 1	**-1.4**	**-3.8**			**-2.0**
ASXL1	additional sex combs like 1 (Drosophila)	**-1.6**	**-1.4**			**-2.2**
ATF2	activating transcription factor 2	**1.6**	**1.5**			**-1.5**
ATF4	activating transcription factor 4 (tax-responsive enhancer element B67)	**-1.3**	**-1.4**	**-1.5**		
ATF5	activating transcription factor 5	**-1.6**	**-2.0**			
ATIC	5-aminoimidazole-4-carboxamide ribonucleotide formyltransferase/IMP cyclohydrolase	**-2.1**	**-3.2**	**-1.4**	**-2.0**	**-1.3**
ATOX1	ATX1 antioxidant protein 1 homolog (yeast)	**5.6**				**-13.3**
ATP11B	ATPase, Class VI, type 11B	**-1.6**	**5.1**		**1.5**	**4.7**
ATP1A1	ATPase, Na+/K+ transporting, alpha 1 polypeptide	**1.3**	**-1.6**			**-2.4**
ATP1A3	ATPase, Na+/K+ transporting, alpha 3 polypeptide	**-13.1**				
ATP2A2	ATPase, Ca++ transporting, cardiac muscle, slow twitch 2	**1.3**	**-1.5**			**-2.5**
ATP2A3	ATPase, Ca++ transporting, ubiquitous	**2.5**	**1.2**			**-2.0**
ATP2B1	ATPase, Ca++ transporting, plasma membrane 1	**-2.1**	**-2.1**			
ATP2B4	ATPase, Ca++ transporting, plasma membrane 4	**-3.8**	**2.8**			**3.9**
ATP2C1	ATPase, Ca++ transporting, type 2C, member 1	**1.9**				**1.5**
ATP5A1	ATP synthase, H+ transporting, mitochondrial F1 complex, alpha subunit 1, cardiac muscle	**1.3**				**-1.5**
ATP5C1	ATP synthase, H+ transporting, mitochondrial F1 complex, gamma polypeptide 1	**1.4**				**-1.5**
ATP5G3	ATP synthase, H+ transporting, mitochondrial F0 complex, subunit C3 (subunit 9)	**2.3**				**-3.8**
ATP5H	ATP synthase, H+ transporting, mitochondrial F0 complex, subunit d	**1.5**	**-1.2**			**-2.4**
ATP5J	ATP synthase, H+ transporting, mitochondrial F0 complex, subunit F6	**-1.8**				**3.0**
ATP5J2	ATP synthase, H+ transporting, mitochondrial F0 complex, subunit F2	**-1.3**				
ATP5L	ATP synthase, H+ transporting, mitochondrial F0 complex, subunit G	**1.7**	**1.6**			**-1.6**
ATP6AP2	ATPase, H+ transporting, lysosomal accessory protein 2	**2.2**	**1.2**			**-1.8**
ATP6V0C	ATPase, H+ transporting, lysosomal 16kDa, V0 subunit c	**-1.4**	**1.3**			**1.9**
ATP6V0D1	ATPase, H+ transporting, lysosomal 38kDa, V0 subunit d1	**1.4**		**1.3**		**-1.3**

ATP6V0E	ATPase, H+ transporting, lysosomal 9kDa, V0 subunit e1	1.3				-1.4
ATP6V1A	ATPase, H+ transporting, lysosomal 70kDa, V1 subunit A	1.9		1.5		-1.9
ATP6V1B2	ATPase, H+ transporting, lysosomal 56/58kDa, V1 subunit B2	1.5				
ATP6V1C1	ATPase, H+ transporting, lysosomal 42kDa, V1 subunit C1	-2.1		-1.4		2.1
ATP6V1D	ATPase, H+ transporting, lysosomal 34kDa, V1 subunit D	-1.3	1.3			1.9
ATP9B	ATPase, Class II, type 9B	-1.7				
ATRX	alpha thalassemia/mental retardation syndrome X-linked (RAD54 homolog, S. cerevisiae)	1.4				1.7
ATXN2L	ataxin 2-like	-2.2				1.8
AURKB	aurora kinase B	-3.3	1.4			3.0
B2M	beta-2-microglobulin	-1.6				3.1
B3GNT1	UDP-GlcNAc:betaGal beta-1,3-N-acetylglucosaminyltransferase 1	-2.7				
B4GALT6	UDP-Gal:betaGlcNAc beta 1,4- galactosyltransferase, polypeptide 6	2.5				-6.7
B930013M22RIK	ras responsive element binding protein 1	3.1	2.1			1.5
BACH1	BTB and CNC homology 1, basic leucine zipper transcription factor 1	1.2	1.3			1.2
BAG2	BCL2-associated athanogene 2	-1.2	-1.8			-1.5
BAMBI	BMP and activin membrane-bound inhibitor homolog (Xenopus laevis)	-2.7			-1.7	1.5
BANF1	barrier to autointegration factor 1	-2.0	-1.5			
BARD1	BRCA1 associated RING domain 1	-3.6	1.3	1.3		3.4
BAT1	HLA-B associated transcript 1	1.5	-1.6			
BAT3	HLA-B associated transcript 3	-1.4	-1.4			
BAX	BCL2-associated X protein	-1.8				
BAZ1B	bromodomain adjacent to zinc finger domain, 1B	-1.9	-1.2			
BC-2	chromatin modifying protein 2A	-1.4	1.5			1.5
BCAP29	B-cell receptor-associated protein 29	2.0	1.5			
BCAT2	branched chain aminotransferase 2, mitochondrial	1.7		-1.3		-2.7
BCKDHB	branched chain keto acid dehydrogenase E1, beta polypeptide (maple syrup urine disease)	2.2				-2.5
BCL10	B-cell CLL/lymphoma 10	1.2				
BCL11A	B-cell CLL/lymphoma 11A (zinc finger protein)	-1.8				1.2
BCL2	B-cell CLL/lymphoma 2	-11.2	-2.8			1.5
BCL2L11	BCL2-like 11 (apoptosis facilitator)	1.2	3.1	3.1	5.9	5.1
BCL7A	B-cell CLL/lymphoma 7A	-3.3	-1.3			1.5
BCL7B	B-cell CLL/lymphoma 7B	-1.4				
BCLAF1	BCL2-associated transcription factor 1	2.0	-1.7	-1.3		-1.8
BCR	breakpoint cluster region	-1.4				4.7
BDH1	3-hydroxybutyrate dehydrogenase, type 1	-1.3		-1.3	-1.5	-1.6
BET1	BET1 homolog (S. cerevisiae)	-2.2				1.6
BICD2	bicaudal D homolog 2 (Drosophila)	-1.2				-1.2
BID	BH3 interacting domain death agonist	-3.4	-1.9	1.4		-1.3
BIN1	bridging integrator 1	-1.3			1.6	9.6
BIRC2	baculoviral IAP repeat-containing 2	1.4	1.9	1.9		1.6
BIRC5	baculoviral IAP repeat-containing 5 (survivin)	-1.8				2.4
BLCAP	bladder cancer associated protein	1.4	-1.5			-1.4

BLMH	bleomycin hydrolase	**1.3**				
BLOC1S1	biogenesis of lysosome-related organelles complex-1, subunit 1	**-1.9**				**1.6**
BMP2K	BMP2 inducible kinase	**1.5**				**-1.6**
BNIP2	BCL2/adenovirus E1B 19kDa interacting protein 2	**-1.4**	**1.7**	**1.5**		**1.4**
BNIP3	BCL2/adenovirus E1B 19kDa interacting protein 3	**312.5**		**1.4**		**-331.2**
BNIP3L	BCL2/adenovirus E1B 19kDa interacting protein 3-like	**2.0**	**-1.3**			**1.9**
BPGM	2,3-bisphosphoglycerate mutase	**-1.5**				
BRCA1	breast cancer 1, early onset	**-1.5**	**-1.4**			**-2.6**
BRCA2	breast cancer 2, early onset	**-2.7**				
BRD4	bromodomain containing 4	**-1.3**				**1.4**
BRMS1	breast cancer metastasis suppressor 1	**-1.6**				
BRRN1	non-SMC condensin I complex, subunit H	**-1.9**				**-1.8**
BST2	bone marrow stromal cell antigen 2	**-1.5**				**1.4**
BTAF1	BTAF1 RNA polymerase II, B-TFIID transcription factor-associated, 170kDa (Mot1 homolog, S. cerevisiae)	**1.6**				**-2.0**
BTG3	BTG family, member 3	**-1.6**				**-2.2**
BTN3A2	butyrophilin, subfamily 3, member A2	**-5.8**				**3.9**
BTN3A3	butyrophilin, subfamily 3, member A3	**-5.9**	**-1.4**			**4.2**
BUB1	BUB1 budding uninhibited by benzimidazoles 1 homolog (yeast)	**-1.3**	**1.4**			**-1.3**
BUB1B	BUB1 budding uninhibited by benzimidazoles 1 homolog beta (yeast)	**-1.9**				
BUB3	BUB3 budding uninhibited by benzimidazoles 3 homolog (yeast)	**-1.4**	**1.3**			**-1.4**
BYSL	bystin-like	**-6.2**	**-8.4**	**-2.2**	**-2.3**	**-4.0**
C13ORF24	chromosome 13 open reading frame 24	**1.2**				**-1.5**
C19ORF10	chromosome 19 open reading frame 10	**1.3**				**-2.2**
C19ORF2	chromosome 19 open reading frame 2	**1.8**	**-1.3**			**-2.7**
C1D	nuclear DNA-binding protein	**-1.6**				
C1ORF48	NSL1, MIND kinetochore complex component, homolog (S. cerevisiae)	**-1.5**				**1.4**
C1QBP	complement component 1, q subcomponent binding protein	**-2.0**	**-1.9**	**-1.8**		
C21ORF33	chromosome 21 open reading frame 33	**1.8**		**1.2**		**-2.5**
C5ORF13	chromosome 5 open reading frame 13	**-3.7**	**-3.3**	**-1.3**		**4.7**
C5ORF18	receptor accessory protein 5	**3.6**	**1.4**			**-4.6**
C5ORF22	chromosome 5 open reading frame 22	**1.2**				**-1.3**
C6ORF108	chromosome 6 open reading frame 108	**2.7**	**-3.3**			**-51.4**
C6ORF69	potassium channel tetramerisation domain containing 20	**-1.3**				**1.2**
C7ORF44	chromosome 7 open reading frame 44	**2.8**				**-4.8**
CABIN1	calcineurin binding protein 1	**-1.3**	**-1.4**	**1.7**	**2.0**	
CALM1	calmodulin 1 (phosphorylase kinase, delta)	**-1.4**	**1.6**			**4.4**
CALM2	calmodulin 2 (phosphorylase kinase, delta)	**-1.2**	**1.2**			
CALM3	calmodulin 3 (phosphorylase kinase, delta)	**-1.5**	**1.2**			**1.3**
CALR	calreticulin	**4.7**	**-1.4**	**-1.9**		**-5.3**
CAMK2G	calcium/calmodulin-dependent protein kinase (CaM kinase) II gamma	**2.1**				**-2.6**
CAMK4	calcium/calmodulin-dependent protein kinase IV	**-5.8**				**8.0**
CAMKK2	calcium/calmodulin-dependent protein kinase kinase 2, beta	**1.7**	**-2.1**			**-3.7**

CAMLG	calcium modulating ligand	2.9	-1.5			-2.0
CAMTA2	calmodulin binding transcription activator 2	-1.5		1.4		2.0
CANX	calnexin	1.6	-1.3			-2.5
CAP1	CAP, adenylate cyclase-associated protein 1 (yeast)	1.3	1.3			1.7
CAP350	centrosomal protein 350kDa	1.6	2.3			1.5
CAPG	capping protein (actin filament), gelsolin-like	-49.5				61.6
CAPN2	calpain 2, (m/II) large subunit	2.4				-2.2
CAPN7	calpain 7	1.4				
CAPNS1	calpain, small subunit 1	-1.3				1.6
CAPZA2	capping protein (actin filament) muscle Z-line, alpha 2	-2.1	1.8			1.9
CARM1	coactivator-associated arginine methyltransferase 1	-1.8	-1.7	-1.6		-1.4
CARS	cysteinyl-tRNA synthetase	-1.7	-1.2		-1.4	
CASK	calcium/calmodulin-dependent serine protein kinase (MAGUK family)	-1.3				1.2
CASP2	caspase 2, apoptosis-related cysteine peptidase (neural precursor cell expressed, developmentally down-regulated 2)	-3.1	-1.8			2.2
CASP6	caspase 6, apoptosis-related cysteine peptidase	2.4	1.4			-1.8
CASP8	caspase 8, apoptosis-related cysteine peptidase	3.2	1.5			-1.5
CASP9	caspase 9, apoptosis-related cysteine peptidase	-1.3				1.3
CAT	catalase	4.5	-1.3	1.3		1.4
CBFA2T2	core-binding factor, runt domain, alpha subunit 2; translocated to, 2	1.5				-1.3
CBFB	core-binding factor, beta subunit	-2.3				1.5
CBL	Cas-Br-M (murine) ecotropic retroviral transforming sequence	1.6				
CBLB	Cas-Br-M (murine) ecotropic retroviral transforming sequence b	-1.6	1.8			4.1
CBX3	chromobox homolog 3 (HP1 gamma homolog, Drosophila)	2.2	1.2			-2.1
CBX6	chromobox homolog 6	-1.4	-2.1			-2.0
CCDC28A	coiled-coil domain containing 28A	-1.3		1.6		1.8
CCDC6	coiled-coil domain containing 6	2.6	1.9			-4.5
CCNA2	cyclin A2	-2.2	1.3			1.5
CCNB1	cyclin B1	-1.5	1.9			2.6
CCNC	cyclin C	1.3				-1.4
CCND3	cyclin D3	-10.6			-1.8	7.2
CCNE1	cyclin E1	2.3				-14.9
CCNE2	cyclin E2	-2.9	-1.3	1.2		2.2
CCNF	cyclin F	-2.6	2.0			2.7
CCNG1	cyclin G1	1.8	1.3			
CCNH	cyclin H	1.6	1.3			-1.5
CCNI	cyclin I	-1.7	1.5			1.6
CCNT2	cyclin T2	2.6	2.0			-2.0
CCT3	chaperonin containing TCP1, subunit 3 (gamma)	1.2	-1.6			-1.7
CCT6A	chaperonin containing TCP1, subunit 6A (zeta 1)	4.9		-1.6	-1.7	-5.1
CCT8	chaperonin containing TCP1, subunit 8 (theta)	1.2	-1.6			-1.3
CD164	CD164 molecule, sialomucin	-1.7	1.8			2.6
CD34	CD34 molecule	12.3	-3.0			-18.1

CD37	CD37 molecule	**2.5**				**-4.9**
CD38	CD38 molecule	**-2.9**	**-2.4**			**8.4**
CD40	CD40 molecule, TNF receptor superfamily member 5	**-1.4**				**1.3**
CD44	CD44 molecule (Indian blood group)	**3.2**	**-3.0**	**-1.7**		**-13.1**
CD47	CD47 molecule	**-2.4**	**-1.7**	**1.7**		**2.0**
CD48	CD48 molecule	**-14.5**	**-2.5**	**1.9**		**4.3**
CD53	CD53 molecule	**1.4**	**3.1**	**3.1**	**3.2**	**7.2**
CD59	CD59 molecule, complement regulatory protein	**-3.7**			**1.8**	**10.6**
CD63	CD63 molecule	**1.4**	**1.2**			
CD69	CD69 molecule	**6.3**	**3.8**	**4.9**	**2.0**	**-1.5**
CD72	CD72 molecule	**-23.3**	**1.6**			**31.2**
CD79A	CD79a molecule, immunoglobulin-associated alpha	**-24.9**	**1.3**	**2.0**	**3.6**	**24.6**
CD81	CD81 molecule	**-1.9**				**1.3**
CD83	CD83 molecule	**2.1**				
CD99	CD99 molecule	**1.7**	**3.4**		**1.8**	**2.6**
CDC16	cell division cycle 16 homolog (S. cerevisiae)	**1.6**	**1.2**			
CDC2	cell division cycle 2, G1 to S and G2 to M	**-1.6**	**2.0**			
CDC23	cell division cycle 23 homolog (S. cerevisiae)	**-1.9**	**-1.3**			**-2.1**
CDC25A	cell division cycle 25 homolog A (S. cerevisiae)	**-1.6**	**-1.4**	**-1.4**		**-19.4**
CDC25B	cell division cycle 25 homolog B (S. cerevisiae)	**-1.2**	**1.6**			**1.6**
CDC2L6	cell division cycle 2-like 6 (CDK8-like)	**-1.4**	**3.3**		**1.8**	**3.8**
CDC6	cell division cycle 6 homolog (S. cerevisiae)	**-1.7**	**-2.9**	**-1.3**	**-1.5**	**-4.7**
CDC7	cell division cycle 7 homolog (S. cerevisiae)	**-1.4**	**-1.4**	**1.3**		**-2.4**
CDH2	cadherin 2, type 1, N-cadherin (neuronal)	**30.0**				**-72.0**
CDIPT	CDP-diacylglycerol--inositol 3-phosphatidyltransferase (phosphatidylinositol synthase)	**-1.6**	**1.4**			**2.8**
CDK2	cyclin-dependent kinase 2	**-2.3**	**-1.5**			**-1.6**
CDK6	cyclin-dependent kinase 6	**-3.0**	**-1.5**			**2.1**
CDK7	cyclin-dependent kinase 7 (MO15 homolog, Xenopus laevis, cdk-activating kinase)	**2.1**				**-1.6**
CDK8	cyclin-dependent kinase 8	**-1.5**				
CDK9	cyclin-dependent kinase 9 (CDC2-related kinase)	**-7.3**	**1.7**			**10.9**
CDKN1B	cyclin-dependent kinase inhibitor 1B (p27, Kip1)	**-1.6**	**1.6**	**1.7**		**3.6**
CDKN3	cyclin-dependent kinase inhibitor 3 (CDK2-associated dual specificity phosphatase)	**-1.4**	**1.5**			**1.2**
CEBPB	CCAAT/enhancer binding protein (C/EBP), beta	**1.5**	**-1.5**			**-3.1**
CEBPG	CCAAT/enhancer binding protein (C/EBP), gamma	**-1.3**				**-1.6**
CEBPZ	CCAAT/enhancer binding protein zeta	**-1.7**	**-1.5**	**-1.5**	**-1.8**	**-2.5**
CENPA	centromere protein A	**-1.3**	**1.7**			**1.4**
CENPF	centromere protein F, 350/400ka (mitosin)	**-2.3**	**1.5**			**2.3**
CENTB1	centaurin, beta 1	**-21.1**				**42.8**
CENTB2	centaurin, beta 2	**1.5**	**1.5**		**1.3**	
CETN3	centrin, EF-hand protein, 3 (CDC31 homolog, yeast)	**-1.5**				
CFL1	cofilin 1 (non-muscle)	**-1.6**				**1.3**
CFLAR	CASP8 and FADD-like apoptosis regulator	**3.8**	**2.1**		**1.5**	

CHAF1B	chromatin assembly factor 1, subunit B (p60)	**-1.7**				**-2.4**
CHD3	chromodomain helicase DNA binding protein 3	**-9.4**	**-1.3**			**5.0**
CHEK1	CHK1 checkpoint homolog (S. pombe)	**-1.8**	**-1.3**			**-2.1**
CHES1	checkpoint suppressor 1	**1.8**	**1.6**	**1.5**		**1.5**
CHI3L2	chitinase 3-like 2	**-3.1**			**-1.8**	
CHP	calcium binding protein P22	**2.4**				**1.6**
CHRNA5	cholinergic receptor, nicotinic, alpha 5	**-2.2**				**1.7**
CHST10	carbohydrate sulfotransferase 10	**2.3**				**-2.6**
CHSY1	carbohydrate (chondroitin) synthase 1	**1.8**	**2.5**			**-2.1**
CHUK	conserved helix-loop-helix ubiquitous kinase	**1.5**				**-1.5**
CIAPIN1	cytokine induced apoptosis inhibitor 1	**1.3**	**-1.6**			**-1.8**
CITED2	Cbp/p300-interacting transactivator, with Glu/Asp-rich carboxy-terminal domain, 2	**10.5**	**2.8**			**-7.0**
CKB	creatine kinase, brain	**3.2**				**-5.0**
CKS1B	CDC28 protein kinase regulatory subunit 1B	**-1.6**				**-1.4**
CKS2	CDC28 protein kinase regulatory subunit 2	**-1.3**	**1.2**			
CLASP1	cytoplasmic linker associated protein 1	**1.5**			**1.7**	**1.8**
CLASP2	cytoplasmic linker associated protein 2	**-1.7**	**1.7**			**2.6**
CLCN3	chloride channel 3	**-3.0**				**3.5**
CLEC11A	C-type lectin domain family 11, member A	**1.5**	**1.4**			**-10.9**
CLIC1	chloride intracellular channel 1	**-1.3**	**1.3**			
CLIC4	chloride intracellular channel 4	**-2.2**				**2.0**
CLK1	CDC-like kinase 1	**1.5**	**1.7**			**1.6**
CLSTN1	calsyntenin 1	**1.3**		**1.3**		**-1.4**
CLTA	clathrin, light chain (Lca)	**1.4**				**-1.3**
CLTB	clathrin, light chain (Lcb)	**-2.0**	**-1.4**			**-1.3**
CLTC	clathrin, heavy chain (Hc)	**1.4**			**-1.5**	**-1.3**
CNAP1	non-SMC condensin I complex, subunit D2	**-2.7**				**2.2**
CNNM2	cyclin M2	**1.7**				
CNOT4	CCR4-NOT transcription complex, subunit 4	**-1.3**	**1.4**			
CNOT8	CCR4-NOT transcription complex, subunit 8	**-1.4**				
CNP	2',3'-cyclic nucleotide 3' phosphodiesterase	**-1.3**		**-1.3**		
COBRA1	cofactor of BRCA1	**-1.3**				
COG5	component of oligomeric golgi complex 5	**1.3**	**-1.3**			
COMT	catechol-O-methyltransferase	**-2.1**				
COPA	coatomer protein complex, subunit alpha	**1.2**				**1.9**
COPB	coatomer protein complex, subunit beta 1	**1.4**				
COPB2	coatomer protein complex, subunit beta 2 (beta prime)	**1.2**				
COPS2	COP9 constitutive photomorphogenic homolog subunit 2 (Arabidopsis)	**1.6**	**-1.2**			**-2.4**
COPS3	COP9 constitutive photomorphogenic homolog subunit 3 (Arabidopsis)	**-2.0**	**-1.2**			
COPS6	COP9 constitutive photomorphogenic homolog subunit 6 (Arabidopsis)	**-1.7**	**-1.5**			**-1.8**
COPS7A	COP9 constitutive photomorphogenic homolog subunit 7A (Arabidopsis)	**-1.4**				**1.3**
COPS8	COP9 constitutive photomorphogenic homolog subunit 8 (Arabidopsis)	**-3.2**				**2.6**

COX11	COX11 homolog, cytochrome c oxidase assembly protein (yeast)	**4.1**	**-1.3**			**-2.9**
COX6A1	cytochrome c oxidase subunit VIa polypeptide 1	**-1.2**				
COX8A	cytochrome c oxidase subunit 8A (ubiquitous)	**-1.5**				
CPD	carboxypeptidase D	**25.6**		**1.4**		**-4.0**
CPNE1	copine I	**-1.5**				**2.1**
CPNE3	copine III	**-1.5**				**2.7**
CPOX	coproporphyrinogen oxidase	**1.8**		**1.5**		**-2.6**
CPSF1	cleavage and polyadenylation specific factor 1, 160kDa	**2.3**				**-2.3**
CPSF5	nudix (nucleoside diphosphate linked moiety X)-type motif 21	**-1.8**	**-1.3**			**-2.2**
CPSF6	cleavage and polyadenylation specific factor 6, 68kDa	**-1.6**				**-2.0**
CPT1A	carnitine palmitoyltransferase 1A (liver)	**1.6**				**-2.0**
CREB1	cAMP responsive element binding protein 1	**-1.5**	**1.4**			**2.6**
CREBBP	CREB binding protein (Rubinstein-Taybi syndrome)	**-10.0**	**1.3**			**14.0**
CREBL2	cAMP responsive element binding protein-like 2	**-2.6**				**3.0**
CREBZF	CREB/ATF bZIP transcription factor	**-1.5**				**-1.9**
CREG1	cellular repressor of E1A-stimulated genes 1	**1.8**	**-1.5**	**1.6**		**-2.3**
CREM	cAMP responsive element modulator	**7.7**				**-1.9**
CRI1	EP300 interacting inhibitor of differentiation 1	**1.9**				**-2.5**
CRIP1	cysteine-rich protein 1 (intestinal)	**-16.0**				**9.6**
CROP	cisplatin resistance-associated overexpressed protein	**1.9**	**-1.5**			**-1.7**
CRTAP	cartilage associated protein	**3.6**	**-2.2**			**-3.4**
CRYZ	crystallin, zeta (quinone reductase)	**-1.7**	**-1.4**		**-1.6**	**1.3**
CSE1L	CSE1 chromosome segregation 1-like (yeast)	**-1.6**	**-1.4**			**-2.5**
CSK	c-src tyrosine kinase	**-1.6**		**-1.8**		**-1.7**
CSNK1A1	casein kinase 1, alpha 1	**2.0**	**1.5**			**1.3**
CSNK1D	casein kinase 1, delta	**1.4**	**1.3**			
CSNK1G2	casein kinase 1, gamma 2	**-1.2**				
CSNK2A1	casein kinase 2, alpha 1 polypeptide	**1.5**	**-1.4**			**-2.0**
CSNK2A2	casein kinase 2, alpha prime polypeptide	**1.9**	**1.9**			
CSNK2B	casein kinase 2, beta polypeptide	**-1.3**				
CSPG6	structural maintenance of chromosomes 3	**2.0**	**-1.7**		**-1.6**	**-2.9**
CST7	cystatin F (leukocystatin)	**-26.4**	**2.4**			**190.4**
CSTB	cystatin B (stefin B)	**1.5**				
CSTF1	cleavage stimulation factor, 3' pre-RNA, subunit 1, 50kDa	**1.6**	**1.3**			**-1.7**
CSTF3	cleavage stimulation factor, 3' pre-RNA, subunit 3, 77kDa	**2.2**				**-4.0**
CTBP1	C-terminal binding protein 1	**-1.5**	**1.3**	**1.3**	**1.3**	**2.2**
CTCF	CCCTC-binding factor (zinc finger protein)	**-1.2**				
CTDSP2	CTD (carboxy-terminal domain, RNA polymerase II, polypeptide A) small phosphatase 2	**1.9**	**-1.3**	**1.4**		**-1.2**
CTNNAL1	catenin (cadherin-associated protein), alpha-like 1	**-2.0**			**-1.3**	
CTNNB1	catenin (cadherin-associated protein), beta 1, 88kDa	**1.4**	**1.4**			**2.5**
CTPS	CTP synthase	**-1.5**	**-1.4**			**-2.4**
CTR9	Ctr9, Paf1/RNA polymerase II complex component, homolog (S. cerevisiae)	**1.4**				**-1.9**

CTSB	cathepsin B	**1.6**	**2.1**			**2.4**
CTSC	--	**2.7**	**-5.2**	**-1.5**		**-7.0**
CTSH	cathepsin H	**-1.4**				
CUGBP1	CUG triplet repeat, RNA binding protein 1	**-1.8**	**-1.3**			**-1.4**
CUGBP2	CUG triplet repeat, RNA binding protein 2	**5.7**	**2.4**	**2.0**	**1.8**	**1.7**
CUL1	cullin 1	**-1.3**		**1.7**		
CUL2	cullin 2	**3.6**				**-3.9**
CUL4A	cullin 4A	**1.5**	**1.4**			**-1.5**
CUL5	cullin 5	**1.4**				
CUTL1	cut-like 1, CCAAT displacement protein (Drosophila)	**-1.5**	**1.7**			**1.9**
CXCR4	chemokine (C-X-C motif) receptor 4	**-2.5**	**1.8**	**1.3**	**1.5**	**63.4**
CXORF40A	chromosome X open reading frame 40A	**-1.2**				
CYB5	cytochrome b5 type A (microsomal)	**-1.3**	**2.3**			**-1.5**
CYB5-M	cytochrome b5 type B (outer mitochondrial membrane)	**-1.4**				
CYC1	cytochrome c-1	**1.5**	**-1.6**	**-1.3**		**-4.4**
CYCS	cytochrome c, somatic	**4.9**	**-1.6**	**-1.6**	**-1.9**	**-13.5**
CYFIP1	cytoplasmic FMR1 interacting protein 1	**-2.0**	**2.3**			**2.9**
CYFIP2	cytoplasmic FMR1 interacting protein 2	**-44.2**	**-2.8**		**-1.3**	**50.3**
CYP51A1	cytochrome P450, family 51, subfamily A, polypeptide 1	**1.3**			**-1.4**	**-2.1**
DAAM1	dishevelled associated activator of morphogenesis 1	**-3.4**	**1.7**			**8.5**
DAD1	defender against cell death 1	**-4.1**				**2.7**
DAG1	dystroglycan 1 (dystrophin-associated glycoprotein 1)	**1.5**				
DAP3	death associated protein 3	**1.9**		**-1.4**	**-1.6**	**-1.7**
DAPK1	death-associated protein kinase 1	**3.4**				**-4.0**
DAPK3	death-associated protein kinase 3	**1.5**	**-1.3**	**1.2**		**-1.8**
DARS	aspartyl-tRNA synthetase	**1.3**				**-1.5**
DATF1	death inducer-obliterator 1	**1.3**	**1.5**			**1.8**
DAZAP2	DAZ associated protein 2	**-1.7**				**1.7**
DBI	diazepam binding inhibitor (GABA receptor modulator, acyl-Coenzyme A binding protein)	**-1.9**				**1.5**
DBN1	drebrin 1	**-1.5**		**-1.5**		
DCK	deoxycytidine kinase	**-1.9**	**1.3**			**2.4**
DCP2	DCP2 decapping enzyme homolog (S. cerevisiae)	**-1.5**	**1.3**			
DCTN1	dynactin 1 (p150, glued homolog, Drosophila)	**1.3**				**1.2**
DCTN2	dynactin 2 (p50)	**1.4**	**1.5**			**1.6**
DDB2	damage-specific DNA binding protein 2, 48kDa	**-3.2**				**2.1**
DDIT4	DNA-damage-inducible transcript 4	**5.5**	**4.6**	**4.4**		**22.4**
DDOST	dolichyl-diphosphooligosaccharide-protein glycosyltransferase	**1.8**				**-1.9**
DDT	D-dopachrome tautomerase	**-1.9**	**1.3**			**1.6**
DDX1	DEAD (Asp-Glu-Ala-Asp) box polypeptide 1	**1.2**	**-1.3**			**-1.8**
DDX10	DEAD (Asp-Glu-Ala-Asp) box polypeptide 10	**1.2**	**-2.1**			**-2.8**
DDX11	DEAD/H (Asp-Glu-Ala-Asp/His) box polypeptide 11 (CHL1-like helicase homolog, S. cerevisiae)	**-2.3**	**-1.5**			**-2.2**
DDX17	DEAD (Asp-Glu-Ala-Asp) box polypeptide 17	**7.2**				**-4.4**

DDX18	DEAD (Asp-Glu-Ala-Asp) box polypeptide 18	**-1.3**	**-1.7**			**-2.6**
DDX21	DEAD (Asp-Glu-Ala-Asp) box polypeptide 21	**1.7**	**-2.1**	**-1.7**		**-3.9**
DDX39	DEAD (Asp-Glu-Ala-Asp) box polypeptide 39	**-1.3**				
DDX3X	DEAD (Asp-Glu-Ala-Asp) box polypeptide 3, X-linked	**-1.8**			**-1.4**	**2.0**
DDX42	DEAD (Asp-Glu-Ala-Asp) box polypeptide 42	**1.6**				**-1.6**
DDX52	DEAD (Asp-Glu-Ala-Asp) box polypeptide 52	**1.7**	**-1.5**			**-2.0**
DECR1	2,4-dienoyl CoA reductase 1, mitochondrial	**1.6**				**5.2**
DEK	DEK oncogene (DNA binding)	**-1.9**				**1.4**
DEXI	dexamethasone-induced transcript	**-1.5**				**1.6**
DFFA	DNA fragmentation factor, 45kDa, alpha polypeptide	**1.6**	**-2.0**			**-1.8**
DGKD	diacylglycerol kinase, delta 130kDa	**-2.5**				**3.2**
DGKG	diacylglycerol kinase, gamma 90kDa	**4.1**			**-4.6**	**-2.6**
DHFR	dihydrofolate reductase	**-1.7**	**-1.9**			**-2.5**
DHPS	deoxyhypusine synthase	**-1.5**				
DHRS1	dehydrogenase/reductase (SDR family) member 1	**2.3**		**1.7**		**-2.0**
DHRS7	dehydrogenase/reductase (SDR family) member 7	**3.4**				**-1.8**
DHX15	DEAH (Asp-Glu-Ala-His) box polypeptide 15	**-1.3**	**-1.6**			**-1.4**
DHX30	DEAH (Asp-Glu-Ala-His) box polypeptide 30	**-2.1**	**-1.7**			**1.8**
DHX9	DEAH (Asp-Glu-Ala-His) box polypeptide 9	**-1.4**				**2.3**
DIAPH1	diaphanous homolog 1 (Drosophila)	**-1.7**	**-1.4**		**-1.4**	**-1.5**
DICER1	Dicer1, Dcr-1 homolog (Drosophila)	**-1.6**				**1.8**
DIP	death-inducing-protein	**-2.3**	**-1.4**		**1.8**	**3.5**
DLAT	dihydrolipoamide S-acetyltransferase (E2 component of pyruvate dehydrogenase complex)	**-1.6**	**-2.1**			**-2.2**
DLEU1	deleted in lymphocytic leukemia, 1	**1.5**	**-1.8**			**-2.0**
DLG1	discs, large homolog 1 (Drosophila)	**-1.5**	**-1.9**			**1.8**
DLG5	discs, large homolog 5 (Drosophila)	**2.2**	**2.0**		**1.3**	**3.1**
DLG7	discs, large homolog 7 (Drosophila)	**-1.3**				**1.8**
DMXL1	Dmx-like 1	**2.2**	**1.8**			**1.6**
DMXL2	Dmx-like 2	**1.7**		**1.6**		**-1.2**
DNAJA1	DnaJ (Hsp40) homolog, subfamily A, member 1	**-1.7**	**-1.4**	**-1.3**	**-1.7**	
DNAJA2	DnaJ (Hsp40) homolog, subfamily A, member 2	**15.8**				**-25.2**
DNAJB1	DnaJ (Hsp40) homolog, subfamily B, member 1	**-1.9**			**-1.4**	**1.5**
DNAJB6	DnaJ (Hsp40) homolog, subfamily B, member 6	**-1.7**				**2.0**
DNAJC11	DnaJ (Hsp40) homolog, subfamily C, member 11	**1.4**	**1.9**	**-1.2**		**-1.8**
DNAJC13	DnaJ (Hsp40) homolog, subfamily C, member 13	**2.0**				
DNAJC7	DnaJ (Hsp40) homolog, subfamily C, member 7	**1.3**	**-1.4**			**-1.6**
DNCI2	dynein, cytoplasmic 1, intermediate chain 2	**2.9**	**1.4**			**-1.5**
DNPEP	aspartyl aminopeptidase	**-4.5**	**-1.6**	**-1.2**		**1.6**
DOC-1R	CDK2-associated protein 2	**-2.0**	**2.0**			**2.5**
DOCK2	dedicator of cytokinesis 2	**1.8**				**-2.8**
DOK1	docking protein 1, 62kDa (downstream of tyrosine kinase 1)	**2.3**				**-2.3**
DPH2L1	DPH1 homolog (S. cerevisiae)	**-1.7**				**1.6**

DPT	dermatopontin	-22.5				
DPYD	dihydropyrimidine dehydrogenase	-8.8				7.7
DPYSL2	dihydropyrimidinase-like 2	-4.2	3.1			3.7
DR1	down-regulator of transcription 1, TBP-binding (negative cofactor 2)	-1.6				
DRAP1	DR1-associated protein 1 (negative cofactor 2 alpha)	-1.5	-2.2			
DSCR1	Down syndrome critical region gene 1	1.5	7.7	5.0	4.8	7.9
DST	dystonin	-8.3				15.8
DSTN	destrin (actin depolymerizing factor)	3.9				
DTX4	deltex 4 homolog (Drosophila)	-2.2				3.3
DTYMK	deoxythymidylate kinase (thymidylate kinase)	-1.9				1.2
DULLARD	dullard homolog (Xenopus laevis)	-1.4	1.8			1.4
DUSP10	dual specificity phosphatase 10	-1.4		2.7		1.9
DUSP3	dual specificity phosphatase 3 (vaccinia virus phosphatase VH1-related)	6.4				-17.5
DUSP6	dual specificity phosphatase 6	15.8	1.7			-28.2
DVL3	dishevelled, dsh homolog 3 (Drosophila)	-1.6				1.9
DZIP3	zinc finger DAZ interacting protein 3	-1.3				2.5
E2F1	E2F transcription factor 1	-1.7			-1.2	-2.7
EBAG9	estrogen receptor binding site associated, antigen, 9	-1.8				2.5
EBP	emopamil binding protein (sterol isomerase)	-2.1		-1.2		
EDD1	E3 ubiquitin protein ligase, HECT domain containing, 1	-1.4	1.4	1.3	-1.4	1.9
EDEM1	ER degradation enhancer, mannosidase alpha-like 1	-1.5	-1.2			1.4
EED	embryonic ectoderm development	-1.9				1.2
EEF1A1	eukaryotic translation elongation factor 1 alpha 1	4.9				-5.1
EEF1D	eukaryotic translation elongation factor 1 delta (guanine nucleotide exchange protein)	1.6				-1.3
EEF1E1	eukaryotic translation elongation factor 1 epsilon 1	-1.6	-3.3	-1.7		-2.4
EEF2	eukaryotic translation elongation factor 2	1.3				
EFNB2	ephrin-B2	-5.9	-2.8			5.8
EHBP1	EH domain binding protein 1	1.4				-2.6
EHD1	EH-domain containing 1	1.6				-1.4
EI24	etoposide induced 2.4 mRNA	1.3	-1.5			-2.1
EIF1AX	eukaryotic translation initiation factor 1A, X-linked	-2.2	-1.5			-2.8
EIF2A	eukaryotic translation initiation factor 2A, 65kDa	1.3				1.2
EIF2C2	eukaryotic translation initiation factor 2C, 2	2.6	-1.6			-4.3
EIF2S1	eukaryotic translation initiation factor 2, subunit 1 alpha, 35kDa	-1.2	-1.6	-1.4	-1.4	-1.8
EIF2S3	eukaryotic translation initiation factor 2, subunit 3 gamma, 52kDa	1.3				
EIF3S10	eukaryotic translation initiation factor 3, subunit 10 theta, 150/170kDa	3.9	-1.3		-1.7	-5.4
EIF3S6	eukaryotic translation initiation factor 3, subunit 6 48kDa	-1.5	-1.6			
EIF3S7	eukaryotic translation initiation factor 3, subunit 7 zeta, 66/67kDa	1.3				-1.3
EIF3S8	eukaryotic translation initiation factor 3, subunit 8, 110kDa	-3.1	-1.4	-1.2		3.4
EIF3S9	eukaryotic translation initiation factor 3, subunit 9 eta, 116kDa	-1.2	-2.0	-1.5		-2.2
EIF4A2	eukaryotic translation initiation factor 4A, isoform 2	1.4				
EIF4B	eukaryotic translation initiation factor 4B	1.8	-2.4			-1.8

EIF4E	eukaryotic translation initiation factor 4E	**-1.3**				**-1.4**
EIF4E2	eukaryotic translation initiation factor 4E family member 2	**-1.6**	**1.2**			**1.5**
EIF5	eukaryotic translation initiation factor 5	**1.2**				**-1.8**
EIF5A	eukaryotic translation initiation factor 5A	**-10.2**	**-2.0**			**3.5**
EIF5B	eukaryotic translation initiation factor 5B	**2.5**	**-1.6**			**-4.3**
ELF1	E74-like factor 1 (ets domain transcription factor)	**-1.8**	**1.4**			**2.7**
ELF2	E74-like factor 2 (ets domain transcription factor)	**-1.8**				**1.4**
ELK3	ELK3, ETS-domain protein (SRF accessory protein 2)	**5.2**				**-2.4**
ELMO1	engulfment and cell motility 1	**3.6**	**2.1**			
ELOVL5	ELOVL family member 5, elongation of long chain fatty acids (FEN1/Elo2, SUR4/Elo3-like, yeast)	**-1.6**	**1.3**			**1.8**
ENTH	clathrin interactor 1	**2.5**	**1.9**			**-1.5**
ENTPD4	ectonucleoside triphosphate diphosphohydrolase 4	**1.5**	**1.3**			
EP400	E1A binding protein p400	**1.3**	**1.4**			
EPB41L2	erythrocyte membrane protein band 4.1-like 2	**1.8**				**-1.9**
EPIM	syntaxin 2	**1.3**				
EPM2A	epilepsy, progressive myoclonus type 2A, Lafora disease (laforin)	**-2.2**				
EPOR	erythropoietin receptor	**16.6**				**-6.1**
EPRS	glutamyl-prolyl-tRNA synthetase	**1.3**	**-1.7**			**-2.4**
EPS15	epidermal growth factor receptor pathway substrate 15	**1.6**	**1.8**	**1.3**		**1.5**
ERCC1	excision repair cross-complementing rodent repair deficiency, complementation group 1 (includes overlapping antisense sequence)	**-1.8**	**1.3**	**-1.3**		
ERCC5	excision repair cross-complementing rodent repair deficiency, complementation group 5 (xeroderma pigmentosum, complementation group G (Cockayne syndrome))	**-1.4**				**1.8**
ERF	Ets2 repressor factor	**2.1**	**-1.5**			**-2.5**
ERH	enhancer of rudimentary homolog (Drosophila)	**-1.2**				
ERP29	endoplasmic reticulum protein 29	**1.4**	**-1.3**			**-1.8**
ESD	esterase D/formylglutathione hydrolase	**2.3**	**1.7**			**-1.9**
ETF1	eukaryotic translation termination factor 1	**1.3**	**-1.6**			**-2.4**
ETFA	electron-transfer-flavoprotein, alpha polypeptide (glutaric aciduria II)	**-1.3**				
ETHE1	ethylmalonic encephalopathy 1	**1.6**	**-1.3**			**-1.6**
ETS1	v-ets erythroblastosis virus E26 oncogene homolog 1 (avian)	**-29.1**				**26.5**
ETS2	v-ets erythroblastosis virus E26 oncogene homolog 2 (avian)	**-1.5**	**5.1**	**-1.3**	**-1.7**	**4.1**
ETV6	ets variant gene 6 (TEL oncogene)	**2.5**	**-1.6**			**-3.1**
EXO1	exonuclease 1	**-1.6**	**-2.1**			**-4.6**
EXOSC8	exosome component 8	**-2.4**				
EXTL2	exostoses (multiple)-like 2	**1.5**	**-2.0**			**-1.9**
EZH1	enhancer of zeste homolog 1 (Drosophila)	**1.3**	**-1.4**			**2.0**
EZH2	enhancer of zeste homolog 2 (Drosophila)	**-1.6**				**-1.3**
F8A1	coagulation factor VIII-associated (intronic transcript) 1	**1.6**		**1.2**		**-2.1**
FABP5	fatty acid binding protein 5 (psoriasis-associated)	**-2.2**	**-4.4**			**-2.5**
FADS1	fatty acid desaturase 1	**-1.5**	**-1.5**		**-1.9**	**-1.8**
FADS2	fatty acid desaturase 2	**-1.9**	**-2.0**			
FADS3	fatty acid desaturase 3	**-3.1**		**-1.2**		**5.6**
FAH	fumarylacetoacetate hydrolase (fumarylacetoacetase)	**1.6**		**-1.5**		**-2.8**

FALZ	bromodomain PHD finger transcription factor	1.6	-1.5			-1.3
FAM38A	family with sequence similarity 38, member A	2.6	2.6			1.8
FAM89B	family with sequence similarity 89, member B	-2.5				5.2
FANCL	Fanconi anemia, complementation group L	1.6				-2.8
FARSLA	phenylalanine-tRNA synthetase-like, alpha subunit	1.6	-2.1	-1.9	-2.0	-4.1
FBL	fibrillarin	1.4	-1.2	-1.4	-1.4	-2.1
FBXO9	F-box protein 9	2.2	-1.4			-1.9
FBXW11	F-box and WD-40 domain protein 11	1.3	1.3			
FCHSD2	FCH and double SH3 domains 2	1.9	3.0			4.0
FDFT1	farnesyl-diphosphate farnesyltransferase 1	1.4				-1.2
FDPS	farnesyl diphosphate synthase (farnesyl pyrophosphate synthetase, dimethylallyltranstransferase, geranyltranstransferase)	-2.3				1.6
FEN1	flap structure-specific endonuclease 1	-1.3	-1.8			-2.9
FEZ2	fasciculation and elongation protein zeta 2 (zygin II)	1.5				1.6
FGF9	fibroblast growth factor 9 (glia-activating factor)	-45.7	-2.1			39.2
FGFR1	fibroblast growth factor receptor 1 (fms-related tyrosine kinase 2, Pfeiffer syndrome)	7.4		1.6		-1.6
FGFR1OP	FGFR1 oncogene partner	1.5				-2.7
FH	fumarate hydratase	-1.4	-2.1			-2.6
FHL1	four and a half LIM domains 1	-2.1	1.8	3.3	13.7	9.6
FIBP	fibroblast growth factor (acidic) intracellular binding protein	-1.8	-1.3			
FKBP1A	FK506 binding protein 1A, 12kDa	1.5	-1.3		1.3	-1.7
FKBP4	FK506 binding protein 4, 59kDa	3.0				-8.6
FKBP5	FK506 binding protein 5	7.0	21.4	6.6	4.4	13.7
FLII	flightless I homolog (Drosophila)	-1.4				1.8
FLJ12443	acyltransferase like 2	-1.6				
FLNA	filamin A, alpha (actin binding protein 280)	-2.2				3.1
FLNB	filamin B, beta (actin binding protein 278)	1.6				
FMR1	fragile X mental retardation 1	-2.3	1.4			2.8
FNBP1	formin binding protein 1	-17.0	-2.9			13.3
FNBP1L	formin binding protein 1-like	1.4	3.0	1.9		3.5
FNBP3	PRP40 pre-mRNA processing factor 40 homolog A (yeast)	2.2	-2.1			-2.4
FNBP4	formin binding protein 4	-1.4	-1.6			
FNTA	farnesyltransferase, CAAX box, alpha	1.4				-1.2
FOSL2	FOS-like antigen 2	7.1	4.0		1.4	-1.9
FOXJ2	forkhead box J2	-2.0				2.0
FOXK2	forkhead box K2	1.8			-1.4	-1.8
FOXM1	forkhead box M1	-3.5	1.4			2.2
FOXO3A	forkhead box O3A	1.7	2.7			2.3
FSCN1	fascin homolog 1, actin-bundling protein (Strongylocentrotus purpuratus)	1.7	1.6	-1.5		-3.9
FTH1	ferritin, heavy polypeptide 1	-2.0	1.5			2.9
FTL	ferritin, light polypeptide	1.9	-1.4			-2.3
FTSJ1	FtsJ homolog 1 (E. coli)	1.2	-1.3			-2.4
FUBP1	far upstream element (FUSE) binding protein 1	1.7	-1.8			-1.9

FUS	fusion (involved in t(12;16) in malignant liposarcoma)	**-2.6**	**-1.5**			**1.5**
FUSIP1	FUS interacting protein (serine/arginine-rich) 1	**-1.5**	**-1.9**			
FUT4	fucosyltransferase 4 (alpha (1,3) fucosyltransferase, myeloid-specific)	**1.3**	**1.7**			
FUT8	fucosyltransferase 8 (alpha (1,6) fucosyltransferase)	**-1.4**				
FXR1	fragile X mental retardation, autosomal homolog 1	**1.4**	**1.4**			**1.4**
FYB	FYN binding protein (FYB-120/130)	**11.8**				**-7.9**
FYN	FYN oncogene related to SRC, FGR, YES	**-8.6**		**1.2**		**2.0**
FZD6	frizzled homolog 6 (Drosophila)	**10.1**	**2.1**	**1.5**		**-2.9**
FZR1	fizzy/cell division cycle 20 related 1 (Drosophila)	**-1.9**				**2.3**
G1P2	ISG15 ubiquitin-like modifier	**-3.1**				**2.0**
G3BP	GTPase activating protein (SH3 domain) binding protein 1	**1.4**	**-2.2**			**-4.3**
G3BP2	GTPase activating protein (SH3 domain) binding protein 2	**1.3**				**-1.7**
GABARAP	GABA(A) receptor-associated protein	**-1.3**				**2.1**
GABPB2	GA binding protein transcription factor, beta subunit 2	**-1.7**	**1.5**			**1.8**
GADD45B	growth arrest and DNA-damage-inducible, beta	**1.3**	**1.5**			**1.4**
GAK	cyclin G associated kinase	**1.4**				
GALC	galactosylceramidase	**-2.1**				**1.5**
GALK2	galactokinase 2	**1.3**				**-1.7**
GALNT1	UDP-N-acetyl-alpha-D-galactosamine:polypeptide N-acetylgalactosaminyltransferase 1 (GalNAc-T1)	**2.0**				**-2.2**
GALNT2	UDP-N-acetyl-alpha-D-galactosamine:polypeptide N-acetylgalactosaminyltransferase 2 (GalNAc-T2)	**-2.2**	**1.4**			**1.9**
GAPD	glyceraldehyde-3-phosphate dehydrogenase	**-1.2**				**1.3**
GAPDHS	glyceraldehyde-3-phosphate dehydrogenase, spermatogenic	**1.8**				**-2.4**
GARS	glycyl-tRNA synthetase	**1.9**	**-2.1**	**-1.4**	**-1.7**	**-4.3**
GART	phosphoribosylglycinamide formyltransferase, phosphoribosylglycinamide synthetase, phosphoribosylaminoimidazole synthetase	**1.4**	**-2.4**	**-1.6**		**-4.2**
GAS7	growth arrest-specific 7	**-10.7**	**1.5**			**7.5**
GATA3	GATA binding protein 3	**-3.1**				**3.3**
GBAS	glioblastoma amplified sequence	**4.0**				**-3.2**
GCAT	glycine C-acetyltransferase (2-amino-3-ketobutyrate coenzyme A ligase)	**9.1**	**-1.4**			**-10.8**
GCH1	GTP cyclohydrolase 1 (dopa-responsive dystonia)	**-1.7**	**-1.7**			**1.5**
GCLC	glutamate-cysteine ligase, catalytic subunit	**2.0**	**1.7**			**-1.3**
GCLM	glutamate-cysteine ligase, modifier subunit	**-2.5**	**2.4**			**3.6**
GCN1L1	GCN1 general control of amino-acid synthesis 1-like 1 (yeast)	**1.3**	**-1.4**			**-1.5**
GCSH	glycine cleavage system protein H (aminomethyl carrier)	**2.1**	**-3.0**	**-1.6**		**-4.6**
GDI1	GDP dissociation inhibitor 1	**-1.6**	**1.4**			**2.4**
GDI2	GDP dissociation inhibitor 2	**1.7**	**1.4**			
GEMIN4	gem (nuclear organelle) associated protein 4	**-1.6**			**-1.5**	**-3.0**
GFI1	growth factor independent 1	**65.2**				**-5.9**
GFPT1	glutamine-fructose-6-phosphate transaminase 1	**-2.1**				
GGA3	golgi associated, gamma adaptin ear containing, ARF binding protein 3	**2.0**			**-1.5**	
GGH	gamma-glutamyl hydrolase (conjugase, folylpolygammaglutamyl hydrolase)	**-2.7**	**1.2**			**1.3**
GGPS1	geranylgeranyl diphosphate synthase 1	**-1.5**	**1.2**			**-1.2**
GIT2	G protein-coupled receptor kinase interactor 2	**12.8**				**-2.8**

GJA7	gap junction protein, alpha 7, 45kDa (connexin 45)	**-13.5**	**1.6**			**43.1**
GLA	galactosidase, alpha	**2.3**				**-3.6**
GLB1	galactosidase, beta 1	**2.3**		**1.4**	**1.9**	**-1.9**
GLMN	glomulin, FKBP associated protein	**3.5**				**-2.2**
GLO1	glyoxalase I	**1.3**	**-1.5**			**-2.0**
GLRX	glutaredoxin (thioltransferase)	**-4.0**	**1.6**	**3.4**		**4.4**
GLS	glutaminase	**2.1**	**-2.1**		**1.7**	**-2.2**
GLUL	glutamate-ammonia ligase (glutamine synthetase)	**2.3**	**2.5**	**1.9**		**1.8**
GMPS	guanine monphosphate synthetase	**-1.4**	**-1.4**			**-2.1**
GNA11	guanine nucleotide binding protein (G protein), alpha 11 (Gq class)	**1.6**				**-4.6**
GNA13	guanine nucleotide binding protein (G protein), alpha 13	**1.6**				**-1.5**
GNA15	guanine nucleotide binding protein (G protein), alpha 15 (Gq class)	**-3.2**	**1.4**			
GNAQ	guanine nucleotide binding protein (G protein), q polypeptide	**5.0**	**1.3**			**-1.6**
GNAS	GNAS complex locus	**-1.7**	**1.7**			**2.4**
GNB1	guanine nucleotide binding protein (G protein), beta polypeptide 1	**-1.4**				**1.7**
GNB2	guanine nucleotide binding protein (G protein), beta polypeptide 2	**-1.7**	**1.4**			**1.7**
GNB5	guanine nucleotide binding protein (G protein), beta 5	**-2.8**	**-1.3**			
GNE	glucosamine (UDP-N-acetyl)-2-epimerase/N-acetylmannosamine kinase	**-3.9**			**-1.4**	**2.5**
GNL1	guanine nucleotide binding protein-like 1	**-1.3**				
GNPDA1	glucosamine-6-phosphate deaminase 1	**11.5**				**-59.9**
GNS	glucosamine (N-acetyl)-6-sulfatase (Sanfilippo disease IIID)	**2.8**				**-2.0**
GOLGA2	golgi autoantigen, golgin subfamily a, 2	**1.7**				
GORASP2	golgi reassembly stacking protein 2, 55kDa	**1.8**	**-1.3**			**-2.0**
GOSR1	golgi SNAP receptor complex member 1	**1.5**				**-1.7**
GPS2	G protein pathway suppressor 2	**-4.7**				**3.8**
GPSM2	G-protein signalling modulator 2 (AGS3-like, C. elegans)	**-1.8**	**1.6**	**1.7**		**2.7**
GPSM3	G-protein signalling modulator 3 (AGS3-like, C. elegans)	**-1.9**				**3.0**
GPSN2	glycoprotein, synaptic 2	**-1.3**				**-1.5**
GPX1	glutathione peroxidase 1	**1.7**	**1.7**			
GPX4	glutathione peroxidase 4 (phospholipid hydroperoxidase)	**1.8**				**-2.2**
GPX7	glutathione peroxidase 7	**1.9**				**-1.5**
GRB10	growth factor receptor-bound protein 10	**-12.4**	**-1.4**			**11.1**
GRB2	growth factor receptor-bound protein 2	**1.9**	**-1.8**			**-3.3**
GREB1	GREB1 protein	**8.1**			**2.6**	**-11.0**
GREM1	gremlin 1, cysteine knot superfamily, homolog (Xenopus laevis)	**-42.1**				
GRHPR	glyoxylate reductase/hydroxypyruvate reductase	**1.4**	**-1.6**			**-1.3**
GRIK5	glutamate receptor, ionotropic, kainate 5	**4.2**				**-14.5**
GRK5	G protein-coupled receptor kinase 5	**14.8**	**2.8**			**-3.8**
GRK6	G protein-coupled receptor kinase 6	**-3.3**				**2.2**
GRLF1	glucocorticoid receptor DNA binding factor 1	**1.4**				**-1.2**
GSK3B	glycogen synthase kinase 3 beta	**2.1**	**1.3**			**-2.3**
GSPT1	G1 to S phase transition 1	**-1.6**	**-1.2**	**-1.5**	**-1.8**	**-1.4**

GSR	glutathione reductase	-1.6				1.2
GSTO1	glutathione S-transferase omega 1	1.8	-1.3			-3.0
GSTP1	glutathione S-transferase pi	-1.2				-1.7
GTF2A2	general transcription factor IIA, 2, 12kDa	-2.8	-1.2		1.5	1.6
GTF2B	general transcription factor IIB	-1.2				
GTF2E2	general transcription factor IIE, polypeptide 2, beta 34kDa	-1.5	-1.3	-1.4		
GTF2H1	general transcription factor IIH, polypeptide 1, 62kDa	1.3	-1.3			-2.0
GTF2H2	general transcription factor IIH, polypeptide 2, 44kDa	2.1				-3.2
GTF2H4	general transcription factor IIH, polypeptide 4, 52kDa	1.7			-1.3	
GTF2I	general transcription factor II, i	-2.1				2.3
GTF3A	general transcription factor IIIA	-1.7	-1.4			-2.0
GTF3C1	general transcription factor IIIC, polypeptide 1, alpha 220kDa	-1.3				1.7
GTPBP6	GTP binding protein 6 (putative)	-2.2				1.8
GTSE1	G-2 and S-phase expressed 1	-1.7	1.6			2.0
GUSB	glucuronidase, beta	1.2				
GYPC	glycophorin C (Gerbich blood group)	1.8			2.8	-1.8
GYS1	glycogen synthase 1 (muscle)	1.5	-1.3			
H1F0	H1 histone family, member 0	8.1	2.4		2.5	-5.6
H1FX	H1 histone family, member X	-3.2		1.5	1.3	3.7
H2AFX	H2A histone family, member X	-2.6				1.3
H2AFY	H2A histone family, member Y	-1.9	-1.6			-2.8
H3F3A	H3 histone, family 3A	-1.2				1.3
H3F3B	H3 histone, family 3B (H3.3B)	4.1	1.3			-2.2
H41	CDV3 homolog (mouse)	1.3	-1.2			-1.4
H6PD	hexose-6-phosphate dehydrogenase (glucose 1-dehydrogenase)	-2.4				
HADH2	hydroxysteroid (17-beta) dehydrogenase 10	-1.2				
HADHB	hydroxyacyl-Coenzyme A dehydrogenase/3-ketoacyl-Coenzyme A thiolase/enoyl-Coenzyme A hydratase (trifunctional protein), beta subunit	1.8				-1.4
HAN11	WD repeat domain 68	1.8	-1.2			-1.7
HAT1	histone acetyltransferase 1	1.6				-4.0
HAX1	HCLS1 associated protein X-1	-1.3	-1.7		-1.3	-1.3
HBP1	HMG-box transcription factor 1	1.6	1.4		1.6	1.5
HCFC1	host cell factor C1 (VP16-accessory protein)	-1.5				1.2
HCLS1	hematopoietic cell-specific Lyn substrate 1	-1.6	-1.4			1.8
HDAC3	histone deacetylase 3	1.5				
HDAC4	histone deacetylase 4	2.6	-1.8			1.3
HDLBP	high density lipoprotein binding protein (vigilin)	-1.7	-1.6			
HELZ	helicase with zinc finger	1.5				-1.3
HERPUD1	homocysteine-inducible, endoplasmic reticulum stress-inducible, ubiquitin-like domain member 1	-2.1				2.1
HEXIM1	hexamethylene bis-acetamide inducible 1	4.5	1.8			-2.2
HGS	hepatocyte growth factor-regulated tyrosine kinase substrate	1.5				-1.7
HHEX	homeobox, hematopoietically expressed	3.4	-2.0			-5.4
HINT1	histidine triad nucleotide binding protein 1	1.3	1.4			

HIP1R	huntingtin interacting protein 1 related	-1.5	2.3			1.6
HIP2	huntingtin interacting protein 2	-1.6	-1.2			-2.2
HIPK3	homeodomain interacting protein kinase 3	1.3	1.5			1.5
HISPPD2A	histidine acid phosphatase domain containing 2A	-3.1				2.5
HIST2H2AA	histone cluster 2, H2aa3	-2.0				2.9
HIVEP1	human immunodeficiency virus type I enhancer binding protein 1	1.4	2.4			-1.5
HIVEP2	human immunodeficiency virus type I enhancer binding protein 2	-6.5				10.1
HK2	hexokinase 2	1.4	-3.1	-2.0		-2.3
HLA-A	major histocompatibility complex, class I, A	-2.5		1.5		2.7
HLA-B	major histocompatibility complex, class I, B	-3.4	1.2			3.7
HLA-DMB	major histocompatibility complex, class II, DM beta	-1.7				
HLA-F	major histocompatibility complex, class I, F	-3.0	2.2			4.7
HLA-G	HLA-G histocompatibility antigen, class I, G	-2.0	1.5	1.5	1.3	1.9
HMBS	hydroxymethylbilane synthase	-1.6				-1.9
HMGB1	high-mobility group box 1	-3.0				1.9
HMGCR	3-hydroxy-3-methylglutaryl-Coenzyme A reductase	-1.9		-1.4		2.0
HMGCS1	3-hydroxy-3-methylglutaryl-Coenzyme A synthase 1 (soluble)	-3.5		-2.3	-1.5	2.4
HMMR	hyaluronan-mediated motility receptor (RHAMM)	-1.5	1.8			3.3
HNRPA0	heterogeneous nuclear ribonucleoprotein A0	1.5				-1.5
HNRPA1	heterogeneous nuclear ribonucleoprotein A1	1.9	1.4			-1.8
HNRPA2B1	heterogeneous nuclear ribonucleoprotein A2/B1	5.0				-2.4
HNRPA3	heterogeneous nuclear ribonucleoprotein A3	1.8	-1.4			-2.4
HNRPAB	heterogeneous nuclear ribonucleoprotein A/B	-1.4	-1.6	-1.5	-1.4	-1.8
HNRPC	heterogeneous nuclear ribonucleoprotein C (C1/C2)	-1.2	-1.5			-1.9
HNRPD	heterogeneous nuclear ribonucleoprotein D (AU-rich element RNA binding protein 1, 37kDa)	3.1				-1.8
HNRPDL	heterogeneous nuclear ribonucleoprotein D-like	1.7	-1.6			-2.4
HNRPF	heterogeneous nuclear ribonucleoprotein F	1.3				-2.5
HNRPH1	heterogeneous nuclear ribonucleoprotein H1 (H)	-2.9	1.3			2.1
HNRPH3	heterogeneous nuclear ribonucleoprotein H3 (2H9)	-1.4				-1.3
HNRPL	heterogeneous nuclear ribonucleoprotein L	-1.4	-2.0			1.3
HNRPM	heterogeneous nuclear ribonucleoprotein M	1.8	1.8			-2.2
HNRPU	heterogeneous nuclear ribonucleoprotein U (scaffold attachment factor A)	-1.4	-1.4			-2.9
HNRPUL1	heterogeneous nuclear ribonucleoprotein U-like 1	-1.4				3.4
HOMER1	homer homolog 1 (Drosophila)	-2.3				
HOXA9	homeobox A9	-91.3				194.0
HRAS	v-Ha-ras Harvey rat sarcoma viral oncogene homolog	-1.8		-1.3		
HRB	HIV-1 Rev binding protein	-1.7	1.5			3.8
HRMT1L2	protein arginine methyltransferase 1	-1.2	-2.6		-1.7	-2.6
HS2ST1	heparan sulfate 2-O-sulfotransferase 1	1.8				-1.4
HS6ST1	heparan sulfate 6-O-sulfotransferase 1	1.3	1.8		2.2	1.5
HSBP1	heat shock factor binding protein 1	-1.4	-1.3			
HSD17B4	hydroxysteroid (17-beta) dehydrogenase 4	2.0	1.9			2.0

HSF2	heat shock transcription factor 2	**1.2**				
HSPA4L	heat shock 70kDa protein 4-like	**25.8**				**-42.0**
HSPA5	heat shock 70kDa protein 5 (glucose-regulated protein, 78kDa)	**1.6**			**-1.5**	**-3.2**
HSPB1	heat shock 27kDa protein 1	**-2.7**	**-1.3**			**1.6**
HSPCA	heat shock protein 90kDa alpha (cytosolic), class A member 1	**-1.2**			**-1.2**	
HSPCB	heat shock protein 90kDa alpha (cytosolic), class B member 1	**1.4**	**-2.0**	**-1.4**	**-1.3**	**-1.8**
HTATSF1	HIV-1 Tat specific factor 1	**1.4**				**-2.0**
HTR7	5-hydroxytryptamine (serotonin) receptor 7 (adenylate cyclase-coupled)	**-1.4**				
HUWE1	HECT, UBA and WWE domain containing 1	**1.4**				**-1.3**
HYOU1	hypoxia up-regulated 1	**1.8**	**-1.7**			**-2.3**
IARS	isoleucine-tRNA synthetase	**-1.4**	**-2.2**	**-1.6**		**-3.8**
IARS2	isoleucine-tRNA synthetase 2, mitochondrial	**1.4**	**-1.2**			**-1.4**
IBRDC3	IBR domain containing 3	**-2.0**				**2.3**
IBTK	inhibitor of Bruton agammaglobulinemia tyrosine kinase	**1.5**	**1.6**			**-1.4**
ICAM2	intercellular adhesion molecule 2	**-17.5**	**-2.4**	**-1.8**	**-1.7**	**3.3**
ID2	inhibitor of DNA binding 2, dominant negative helix-loop-helix protein	**5.8**	**3.0**	**1.8**		**3.3**
ID3	inhibitor of DNA binding 3, dominant negative helix-loop-helix protein	**-33.8**	**-1.9**			
IDE	insulin-degrading enzyme	**1.4**				**-1.7**
IDH1	isocitrate dehydrogenase 1 (NADP+), soluble	**-1.3**	**-1.6**			**1.8**
IDH2	isocitrate dehydrogenase 2 (NADP+), mitochondrial	**-1.4**	**-1.4**			**-2.3**
IDH3A	isocitrate dehydrogenase 3 (NAD+) alpha	**-1.6**	**-1.7**	**-1.5**	**-1.5**	**-2.4**
IDH3B	isocitrate dehydrogenase 3 (NAD+) beta	**1.5**				**-2.1**
IDI1	isopentenyl-diphosphate delta isomerase 1	**1.5**	**1.7**			**2.1**
IDS	iduronate 2-sulfatase (Hunter syndrome)	**-1.6**				**1.5**
IER2	immediate early response 2	**1.6**			**-1.4**	**-2.7**
IFI16	interferon, gamma-inducible protein 16	**2.0**	**2.4**	**-1.3**		**-1.3**
IFITM1	interferon induced transmembrane protein 1 (9-27)	**4.6**	**3.2**			**-14.3**
IFITM2	interferon induced transmembrane protein 2 (1-8D)	**-3.1**	**2.2**			**1.6**
IFNAR2	interferon (alpha, beta and omega) receptor 2	**-2.9**				
IFNGR1	interferon gamma receptor 1	**5.7**	**3.3**	**1.7**		**2.6**
IGF2R	insulin-like growth factor 2 receptor	**1.3**	**1.7**			**1.7**
IGHG1	immunoglobulin heavy constant gamma 1 (G1m marker)	**-2.6**	**1.3**			**3.7**
IGLL1	immunoglobulin lambda-like polypeptide 1	**-44.6**	**-1.2**	**-1.4**		**9.6**
IHPK1	inositol hexaphosphate kinase 1	**1.8**	**-1.4**			**-1.7**
IKBKG	inhibitor of kappa light polypeptide gene enhancer in B-cells, kinase gamma	**-1.5**				**1.6**
IL27RA	interleukin 27 receptor, alpha	**20.9**				**-7.5**
IL2RG	interleukin 2 receptor, gamma (severe combined immunodeficiency)	**-1.4**	**-1.4**			
IL7R	interleukin 7 receptor	**-56.6**	**-3.1**	**8.3**	**6.3**	**43.1**
ILF3	interleukin enhancer binding factor 3, 90kDa	**1.7**	**-2.2**	**-1.3**		**-4.0**
ILK	integrin-linked kinase	**1.4**		**1.3**		
IMMT	inner membrane protein, mitochondrial (mitofilin)	**1.4**				**-1.6**
IMPA1	inositol(myo)-1(or 4)-monophosphatase 1	**-1.7**	**1.2**			**2.3**

IMPA2	inositol(myo)-1(or 4)-monophosphatase 2	**1.8**	**-12.4**			**-7.9**
IMPDH1	IMP (inosine monophosphate) dehydrogenase 1	**-2.0**		**-1.6**	**-1.4**	
IMPDH2	IMP (inosine monophosphate) dehydrogenase 2	**1.3**	**-1.4**		**-1.5**	**-1.5**
ING3	inhibitor of growth family, member 3	**-1.9**	**1.3**			**3.0**
INPP1	inositol polyphosphate-1-phosphatase	**1.4**	**2.7**	**3.5**	**17.6**	**13.6**
INPP5D	inositol polyphosphate-5-phosphatase, 145kDa	**-1.6**	**2.0**			**1.5**
INSIG1	insulin induced gene 1	**-5.5**	**1.7**			**3.7**
IPO7	importin 7	**1.5**	**-1.7**	**-1.4**		**-4.6**
IQCB1	IQ motif containing B1	**-1.3**			**-1.5**	**-1.5**
IQGAP1	IQ motif containing GTPase activating protein 1	**-2.4**	**2.2**			**4.4**
IRAK1	interleukin-1 receptor-associated kinase 1	**-1.3**				
IRAK1BP1	interleukin-1 receptor-associated kinase 1 binding protein 1	**-1.6**	**-3.0**			**1.6**
IREB2	iron-responsive element binding protein 2	**1.5**				**-1.9**
IRF2	interferon regulatory factor 2	**-2.3**				**2.2**
IRF3	interferon regulatory factor 3	**-1.2**				
IRS1	insulin receptor substrate 1	**-4.8**	**1.5**			**7.7**
ITCH	itchy homolog E3 ubiquitin protein ligase (mouse)	**2.0**	**1.8**			**1.6**
ITGA6	integrin, alpha 6	**6.1**	**7.2**	**5.0**	**2.4**	**-1.9**
ITGAE	integrin, alpha E (antigen CD103, human mucosal lymphocyte antigen 1; alpha polypeptide)	**-2.9**	**1.5**			**3.1**
ITGB1	integrin, beta 1 (fibronectin receptor, beta polypeptide, antigen CD29 includes MDF2, MSK12)	**3.1**	**1.4**			**-2.1**
ITGB1BP1	integrin beta 1 binding protein 1	**-1.2**				
ITGB2	integrin, beta 2 (complement component 3 receptor 3 and 4 subunit)	**2.6**	**-5.6**			**-8.7**
ITM2A	integral membrane protein 2A	**64.4**				**-138.3**
ITM2B	integral membrane protein 2B	**1.7**	**2.0**			**1.9**
ITPKB	inositol 1,4,5-trisphosphate 3-kinase B	**-4.6**				**1.7**
ITPR1	inositol 1,4,5-triphosphate receptor, type 1	**3.8**	**2.4**			**-18.1**
ITPR2	inositol 1,4,5-triphosphate receptor, type 2	**4.3**	**1.8**	**1.5**	**1.5**	**-2.4**
ITSN1	intersectin 1 (SH3 domain protein)	**-3.8**	**-1.3**			**8.7**
IVNS1ABP	influenza virus NS1A binding protein	**-1.3**	**-1.3**			**-1.9**
JAK1	Janus kinase 1 (a protein tyrosine kinase)	**1.9**	**1.8**	**2.2**	**2.6**	**3.4**
JAM3	junctional adhesion molecule 3	**12.9**		**2.4**	**3.6**	**-12.3**
JARID1A	jumonji, AT rich interactive domain 1A	**-2.3**	**1.2**			**3.7**
JARID1B	jumonji, AT rich interactive domain 1B	**37.6**				**-8.0**
JARID2	jumonji, AT rich interactive domain 2	**-1.4**		**-1.3**		**1.9**
JMJD1A	jumonji domain containing 1A	**1.6**				
JMJD1C	jumonji domain containing 1C	**-2.0**	**1.9**			**3.6**
JRK	jerky homolog (mouse)	**2.1**				**-1.8**
JUN	jun oncogene	**42.1**	**18.9**	**4.4**		**-6.2**
JUND	jun D proto-oncogene	**1.3**	**1.8**			**1.4**
KAB	centrosomal protein 170kDa	**3.8**	**1.2**			**-4.0**
KATNB1	katanin p80 (WD repeat containing) subunit B 1	**-1.4**	**-2.1**			
KCNN4	potassium intermediate/small conductance calcium-activated channel, subfamily N, member 4	**23.1**	**-13.3**			**-19.5**

KDELR2	KDEL (Lys-Asp-Glu-Leu) endoplasmic reticulum protein retention receptor 2	**1.3**				**-2.0**
KEAP1	kelch-like ECH-associated protein 1	**-1.4**				
KHDRBS1	KH domain containing, RNA binding, signal transduction associated 1	**-1.3**	**-1.3**			
KHDRBS3	KH domain containing, RNA binding, signal transduction associated 3	**9.0**	**2.3**			**-14.3**
KIAA0020	KIAA0020	**-1.9**	**-2.0**	**-1.7**	**-2.2**	
KIAA0133	KIAA0133	**-1.5**	**-1.4**			**-1.6**
KIAA0143	KIAA0143 protein	**1.9**	**1.4**			**-1.5**
KIAA0247	KIAA0247	**1.7**				**-1.4**
KIAA0310	KIAA0310	**1.5**				**-1.3**
KIAA0368	KIAA0368	**1.8**	**1.8**			**-1.4**
KIAA0828	adenosylhomocysteinase 3	**1.9**				
KIAA0922	KIAA0922	**-2.6**				**1.9**
KIAA0992	palladin, cytoskeletal associated protein	**1.6**		**1.2**	**1.3**	
KIAA0999	KIAA0999 protein	**2.2**	**-1.4**			**-2.7**
KIAA1008	KIAA1008	**1.7**				**-1.9**
KIAA1115	SAPS domain family, member 1	**1.4**				**-1.7**
KIAA1279	KIAA1279	**-1.3**				
KIAA1794	KIAA1794	**-1.9**				**-1.7**
KIDINS220	kinase D-interacting substance of 220 kDa	**1.5**	**1.4**			**1.5**
KIF11	kinesin family member 11	**-1.6**	**1.5**			**1.4**
KIF14	kinesin family member 14	**-1.4**	**1.4**			**1.8**
KIF2	kinesin heavy chain member 2A	**-1.3**		**-1.2**		**1.6**
KIF3B	kinesin family member 3B	**1.4**				
KIF5B	kinesin family member 5B	**1.2**	**1.5**			
KIFC1	kinesin family member C1	**-1.6**				
KLF10	Kruppel-like factor 10	**-2.3**	**2.4**			**1.9**
KLF6	Kruppel-like factor 6	**1.8**	**7.2**	**1.5**		**3.5**
KNTC1	kinetochore associated 1	**-1.5**				**-1.3**
KNTC2	kinetochore associated 2	**-1.3**	**1.9**			
KPNA2	karyopherin alpha 2 (RAG cohort 1, importin alpha 1)	**1.3**	**1.5**			
KPNA3	karyopherin alpha 3 (importin alpha 4)	**-1.5**	**-1.3**			**-1.8**
KPNA6	karyopherin alpha 6 (importin alpha 7)	**2.0**				**-2.1**
KPNB1	karyopherin (importin) beta 1	**1.5**	**-1.6**			**-2.8**
KRAS	v-Ki-ras2 Kirsten rat sarcoma viral oncogene homolog	**-2.1**	**13.3**			**13.4**
KRT10	keratin 10 (epidermolytic hyperkeratosis; keratosis palmaris et plantaris)	**-2.8**	**1.2**			**2.1**
KRT8	keratin 8	**-1.4**				**1.6**
KTN1	kinectin 1 (kinesin receptor)	**1.8**				**-1.4**
LAIR1	leukocyte-associated immunoglobulin-like receptor 1	**6.7**	**2.7**	**1.7**	**1.6**	**-1.9**
LAMP1	lysosomal-associated membrane protein 1	**1.8**	**1.5**			**-1.4**
LAMP2	lysosomal-associated membrane protein 2	**2.1**	**1.7**			**-2.3**
LANCL1	LanC lantibiotic synthetase component C-like 1 (bacterial)	**-1.7**				**1.4**
LAP1B	torsin A interacting protein 1	**1.4**				**-2.0**

LAPTM5	lysosomal associated multispanning membrane protein 5	**-4.3**	**2.0**			**10.9**
LARS2	leucyl-tRNA synthetase 2, mitochondrial	**-1.2**	**-1.5**			**-1.4**
LASP1	LIM and SH3 protein 1	**1.6**				
LCK	lymphocyte-specific protein tyrosine kinase	**-49.3**			**-1.7**	**18.5**
LCP1	lymphocyte cytosolic protein 1 (L-plastin)	**-4.1**	**-2.5**			**7.2**
LCP2	lymphocyte cytosolic protein 2 (SH2 domain containing leukocyte protein of 76kDa)	**1.6**	**-2.4**			**-2.2**
LDB1	LIM domain binding 1	**1.7**	**-1.2**			**-1.5**
LDLR	low density lipoprotein receptor (familial hypercholesterolemia)	**-3.6**	**-1.3**			**3.0**
LEF1	lymphoid enhancer-binding factor 1	**-288.8**	**-1.3**	**1.5**		**826.7**
LEPR	leptin receptor	**1.8**	**2.2**			**4.6**
LETMD1	LETM1 domain containing 1	**3.1**				**-7.5**
LGALS1	lectin, galactoside-binding, soluble, 1 (galectin 1)	**-22.2**	**2.9**			**82.1**
LGALS3BP	lectin, galactoside-binding, soluble, 3 binding protein	**-4.3**	**-2.3**			**2.8**
LIG4	ligase IV, DNA, ATP-dependent	**1.9**	**1.8**			
LIMS1	LIM and senescent cell antigen-like domains 1	**1.3**	**1.3**			**3.7**
LIPA	lipase A, lysosomal acid, cholesterol esterase (Wolman disease)	**1.2**	**-2.0**			
LMAN1	lectin, mannose-binding, 1	**1.6**		**-1.5**		**-15.2**
LMNB1	lamin B1	**-2.0**				
LMO2	LIM domain only 2 (rhombotin-like 1)	**7.9**	**4.4**			**-6.9**
LNK	SH2B adaptor protein 3	**1.3**	**-1.2**			**-2.8**
LOC51035	unknown protein LOC51035	**1.3**			**1.5**	
LOC93081	chromosome 13 open reading frame 27	**2.0**				**-1.4**
LPIN1	lipin 1	**-1.6**		**1.3**		**2.3**
LPXN	leupaxin	**-3.2**	**-1.7**			**1.7**
LRBA	LPS-responsive vesicle trafficking, beach and anchor containing	**1.4**	**-2.1**			**-1.6**
LRCH4	leucine-rich repeats and calponin homology (CH) domain containing 4	**-1.9**	**1.9**			**4.1**
LRMP	lymphoid-restricted membrane protein	**-18.6**	**-1.4**			**7.8**
LRP8	low density lipoprotein receptor-related protein 8, apolipoprotein e receptor	**-1.6**	**-2.1**	**-2.2**	**-2.8**	**-2.5**
LRPAP1	low density lipoprotein receptor-related protein associated protein 1	**3.2**				**-2.1**
LRPPRC	leucine-rich PPR-motif containing	**1.7**	**-5.1**	**-1.8**	**-1.7**	**-2.8**
LRRFIP1	leucine rich repeat (in FLII) interacting protein 1	**-6.0**	**2.5**			**11.3**
LSM1	LSM1 homolog, U6 small nuclear RNA associated (S. cerevisiae)	**2.2**				
LSM4	LSM4 homolog, U6 small nuclear RNA associated (S. cerevisiae)	**-1.6**	**-1.4**			**-1.9**
LSM5	LSM5 homolog, U6 small nuclear RNA associated (S. cerevisiae)	**2.9**				**-4.5**
LSM7	LSM7 homolog, U6 small nuclear RNA associated (S. cerevisiae)	**-1.5**	**-1.5**			**-1.4**
LTA4H	leukotriene A4 hydrolase	**2.6**				**-2.9**
LTK	leukocyte tyrosine kinase	**-2.3**				**2.4**
LY6E	lymphocyte antigen 6 complex, locus E	**2.0**				**-12.6**
LYL1	lymphoblastic leukemia derived sequence 1	**3.5**			**1.4**	**-6.7**
LYPLA1	lysophospholipase I	**-1.3**	**1.3**			**1.2**
M11S1	GPI-anchored membrane protein 1	**-2.0**	**-1.3**			**-1.5**
MAC30	transmembrane protein 97	**1.4**	**-3.0**	**-1.3**		**-3.2**

MACF1	microtubule-actin crosslinking factor 1	-1.5	-1.3			2.7
MAD2L1	MAD2 mitotic arrest deficient-like 1 (yeast)	-2.0				
MAG	myelin associated glycoprotein	-40.4	-2.3			2.0
MAGED1	melanoma antigen family D, 1	2.4	-2.1			-4.1
MAGOH	mago-nashi homolog, proliferation-associated (Drosophila)	-1.2				-1.5
MALT1	mucosa associated lymphoid tissue lymphoma translocation gene 1	-1.7	-1.7			2.2
MAN2A1	mannosidase, alpha, class 2A, member 1	2.5	1.7			-1.9
MAN2A2	mannosidase, alpha, class 2A, member 2	8.4	1.3			-4.8
MAN2B1	mannosidase, alpha, class 2B, member 1	2.4				-2.9
MAP2K1	mitogen-activated protein kinase kinase 1	1.5	1.9	1.5	1.7	2.1
MAP2K2	mitogen-activated protein kinase kinase 2	1.3				-1.6
MAP2K3	mitogen-activated protein kinase kinase 3	-2.3				1.9
MAP2K4	mitogen-activated protein kinase kinase 4	-1.4				1.7
MAP2K6	mitogen-activated protein kinase kinase 6	-2.5				
MAP3K11	mitogen-activated protein kinase kinase kinase 11	1.3				-1.7
MAP3K7	mitogen-activated protein kinase kinase kinase 7	1.3				
MAP3K7IP2	mitogen-activated protein kinase kinase kinase 7 interacting protein 2	-1.4	1.5			2.1
MAP4K1	mitogen-activated protein kinase kinase kinase kinase 1	-2.6	-2.7			2.4
MAP4K4	mitogen-activated protein kinase kinase kinase kinase 4	-1.5	1.7			1.7
MAP4K5	mitogen-activated protein kinase kinase kinase kinase 5	1.9	2.2			
MAPK1	mitogen-activated protein kinase 1	-1.4	2.3			2.0
MAPK14	mitogen-activated protein kinase 14	4.1			1.4	-2.1
MAPKAPK3	mitogen-activated protein kinase-activated protein kinase 3	10.5	-1.5	-1.4		-20.4
MAPKAPK5	mitogen-activated protein kinase-activated protein kinase 5	-1.6				
MAPRE1	microtubule-associated protein, RP/EB family, member 1	-1.4	1.5			1.6
MAPRE2	microtubule-associated protein, RP/EB family, member 2	-3.4	-2.5			3.0
MARCH7	membrane-associated ring finger (C3HC4) 7	2.3	2.1			
MARCKSL1	MARCKS-like 1	-1.6	-1.4		-1.6	
MARK3	MAP/microtubule affinity-regulating kinase 3	1.5				
MARS	methionine-tRNA synthetase	-1.5	-2.2			-2.2
MAX	MYC associated factor X	-1.2	-1.7			
MAZ	MYC-associated zinc finger protein (purine-binding transcription factor)	-3.2	-1.7			1.7
MBD1	methyl-CpG binding domain protein 1	1.3				-1.8
MBD2	methyl-CpG binding domain protein 2	-1.6				1.3
MBD3	methyl-CpG binding domain protein 3	-1.6	-1.4			
MBD4	methyl-CpG binding domain protein 4	1.7				-2.2
MBNL1	muscleblind-like (Drosophila)	-4.4	2.0			9.2
MCCC2	methylcrotonoyl-Coenzyme A carboxylase 2 (beta)	1.4	-1.5			-2.1
MCFD2	multiple coagulation factor deficiency 2	1.6				-1.7
MCL1	myeloid cell leukemia sequence 1 (BCL2-related)	1.4	1.7	1.2		1.3
MCM3	MCM3 minichromosome maintenance deficient 3 (S. cerevisiae)	-1.4	-3.1		-1.3	-4.2
MCM4	MCM4 minichromosome maintenance deficient 4 (S. cerevisiae)	-2.1	-3.3			-6.9

MCM5	MCM5 minichromosome maintenance deficient 5, cell division cycle 46 (S. cerevisiae)	-1.5	-1.5			-3.2
MCM6	minichromosome maintenance deficient 6 homolog (S. cerevisiae)	1.3	-2.1			-6.8
MCM7	MCM7 minichromosome maintenance deficient 7 (S. cerevisiae)	-1.9	-1.3			-2.7
MCP	CD46 molecule, complement regulatory protein	1.6	1.4			
MCRS1	microspherule protein 1	-1.7				
MDC1	mediator of DNA damage checkpoint 1	-4.7				2.0
MDH1	malate dehydrogenase 1, NAD (soluble)	1.9	-1.3			-2.2
MDH2	malate dehydrogenase 2, NAD (mitochondrial)	-2.3	-1.5			3.7
MDM4	Mdm4, transformed 3T3 cell double minute 4, p53 binding protein (mouse)	-2.4	2.2			2.5
MED12	mediator of RNA polymerase II transcription, subunit 12 homolog (S. cerevisiae)	1.4				
MED6	mediator of RNA polymerase II transcription, subunit 6 homolog (S. cerevisiae)	-1.4				
MED8	mediator of RNA polymerase II transcription, subunit 8 homolog (S. cerevisiae)	1.7				
MEF2A	MADS box transcription enhancer factor 2, polypeptide A (myocyte enhancer factor 2A)	-3.6	1.7			11.0
MEF2D	MADS box transcription enhancer factor 2, polypeptide D (myocyte enhancer factor 2D)	-5.7				2.0
MEIS2	Meis1, myeloid ecotropic viral integration site 1 homolog 2 (mouse)	3.6	-2.1			-4.4
MELK	maternal embryonic leucine zipper kinase	-1.3	1.4	1.4		
MEN1	multiple endocrine neoplasia I	1.3	-1.5			-1.3
MEP50	WD repeat domain 77	-1.8	-1.5	-1.7		-1.4
MFAP1	microfibrillar-associated protein 1	-1.6	-1.4			
MFNG	MFNG O-fucosylpeptide 3-beta-N-acetylglucosaminyltransferase	3.0	-1.8			-9.2
MGA	MAX gene associated	3.0	-1.8			-7.2
MGC17330	HGFL gene	34.8	7.3	7.3	5.5	12.3
MGC5508	transmembrane protein 109	-1.4	-1.7	-1.4	-1.6	-1.5
MGEA5	meningioma expressed antigen 5 (hyaluronidase)	1.7	1.3			1.9
MICB	MHC class I polypeptide-related sequence B	-1.4				
MINA	MYC induced nuclear antigen	-1.2	-1.7			-1.9
MINPP1	multiple inositol polyphosphate histidine phosphatase, 1	1.6				-1.7
MIR16	membrane interacting protein of RGS16	2.1	-1.4			-2.9
MKI67	antigen identified by monoclonal antibody Ki-67	-2.2	2.1			2.2
MKL1	megakaryoblastic leukemia (translocation) 1	1.5	-1.5			-1.6
MKNK1	MAP kinase interacting serine/threonine kinase 1	1.3			1.9	1.3
MKRN1	makorin, ring finger protein, 1	-1.5				2.1
MLC1	megalencephalic leukoencephalopathy with subcortical cysts 1	1.7			-1.5	-5.1
MLH3	mutL homolog 3 (E. coli)	2.2	-1.3			-2.4
MLL	myeloid/lymphoid or mixed-lineage leukemia (trithorax homolog, Drosophila)	1.8	-1.8			2.0
MLLT10	myeloid/lymphoid or mixed-lineage leukemia (trithorax homolog, Drosophila); translocated to, 10	2.7				-2.2
MMS19L	MMS19-like (MET18 homolog, S. cerevisiae)	1.7			-1.3	-1.4
MNT	MAX binding protein	-1.5				
MOBK1B	MOB1, Mps One Binder kinase activator-like 1B (yeast)	-1.6				1.4
MONDOA	MLX interacting protein	1.5	1.7			
MORF4L2	mortality factor 4 like 2	1.2				
MPG	N-methylpurine-DNA glycosylase	-4.1				4.1

MPHOSPH1	M-phase phosphoprotein 1	**1.9**				**-3.2**
MPHOSPH6	M-phase phosphoprotein 6	**-1.3**	**-1.9**			**-2.3**
MPP1	membrane protein, palmitoylated 1, 55kDa	**-1.9**	**-1.3**			
MPP6	membrane protein, palmitoylated 6 (MAGUK p55 subfamily member 6)	**38.6**				**-21.9**
MPZL1	myelin protein zero-like 1	**-7.5**	**-1.8**			**9.8**
MRC2	mannose receptor, C type 2	**2.0**				**-8.0**
MRCL3	myosin regulatory light chain MRCL3	**-2.0**	**2.2**			**5.8**
M-RIP	myosin phosphatase-Rho interacting protein	**-5.4**	**1.2**			**26.4**
MRPS12	mitochondrial ribosomal protein S12	**-1.5**		**-1.4**	**-1.7**	**-1.7**
MSH2	mutS homolog 2, colon cancer, nonpolyposis type 1 (E. coli)	**-1.5**	**-1.6**		**-1.5**	**-1.8**
MSH6	mutS homolog 6 (E. coli)	**-2.5**	**-2.7**	**-1.2**	**-1.7**	**-2.4**
MSN	moesin	**1.4**	**1.2**			**1.7**
MT1E	metallothionein 1E (functional)	**1.8**	**2.3**		**2.1**	
MT1H	metallothionein 1H	**1.4**	**3.0**		**2.1**	**1.3**
MT1X	metallothionein 1X	**1.6**	**1.8**			**1.7**
MT2A	metallothionein 2A	**3.2**	**3.0**			**1.2**
MTA1	metastasis associated 1	**1.4**	**-1.4**			**-1.6**
MTCP1	mature T-cell proliferation 1	**-1.4**				
MTHFD1	methylenetetrahydrofolate dehydrogenase (NADP+ dependent) 1, methenyltetrahydrofolate cyclohydrolase, formyltetrahydrofolate synthetase	**1.5**	**-2.2**	**-1.4**	**-1.5**	**-5.0**
MTHFS	5,10-methenyltetrahydrofolate synthetase (5-formyltetrahydrofolate cyclo-ligase)	**-1.4**			**1.3**	
MTM1	myotubularin 1	**2.5**				**-2.5**
MTMR2	myotubularin related protein 2	**-1.2**	**-1.3**			
MTMR3	myotubularin related protein 3	**-1.4**	**1.5**			**1.9**
MTMR6	myotubularin related protein 6	**-1.4**	**1.7**			**1.5**
MTX2	metaxin 2	**1.3**				**-2.1**
MUT	methylmalonyl Coenzyme A mutase	**-1.6**				**2.0**
MXI1	MAX interactor 1	**6.4**	**-1.7**	**-2.7**		**-6.2**
MYB	v-myb myeloblastosis viral oncogene homolog (avian)	**2.2**	**1.8**			**-2.0**
MYC	v-myc myelocytomatosis viral oncogene homolog (avian)	**1.6**	**-2.8**	**-3.8**	**-3.6**	**-4.8**
MYCBP	c-myc binding protein	**1.3**	**-1.2**			**-1.6**
MYCBP2	MYC binding protein 2	**-1.5**	**-1.4**			
MYH10	myosin, heavy chain 10, non-muscle	**-3.7**	**-1.3**			**3.8**
MYH9	myosin, heavy chain 9, non-muscle	**-1.8**			**1.6**	**2.2**
MYL6B	myosin, light chain 6B, alkali, smooth muscle and non-muscle	**-1.7**				
MYO1B	myosin IB	**3.5**	**2.3**	**11.8**		**-6.5**
MYO5A	myosin VA (heavy chain 12, myoxin)	**-8.2**				**6.2**
MYST1	MYST histone acetyltransferase 1	**-1.9**				**2.3**
MYST4	MYST histone acetyltransferase (monocytic leukemia) 4	**2.2**	**-1.9**			**-2.6**
NACA	nascent-polypeptide-associated complex alpha polypeptide	**1.3**				**-1.9**
NAGA	N-acetylgalactosaminidase, alpha-	**1.6**				**-2.4**
NAP1L1	nucleosome assembly protein 1-like 1	**1.9**	**-1.2**	**-1.3**		**-2.9**
NAP1L4	nucleosome assembly protein 1-like 4	**-1.5**				**-1.3**

NAPA	N-ethylmaleimide-sensitive factor attachment protein, alpha	**-1.5**				
NAT1	N-acetyltransferase 1 (arylamine N-acetyltransferase)	**1.2**	**1.3**			
NBL1	neuroblastoma, suppression of tumorigenicity 1	**2.3**				**-3.0**
NBR1	neighbor of BRCA1 gene 1	**2.6**	**1.5**			**-1.2**
NBS1	nibrin	**-1.6**	**-1.2**	**1.9**	**1.7**	**1.8**
NCBP1	nuclear cap binding protein subunit 1, 80kDa	**-1.3**	**-1.2**			
NCF4	neutrophil cytosolic factor 4, 40kDa	**-1.9**	**-1.5**			**1.6**
NCK1	NCK adaptor protein 1	**1.7**	**1.5**	**1.4**	**2.3**	
NCKAP1	NCK-associated protein 1	**6.0**				**-5.9**
NCKAP1L	NCK-associated protein 1-like	**1.3**		**1.4**		
NCL	nucleolin	**-1.6**	**-1.9**	**-1.3**		**-1.2**
NCOA3	nuclear receptor coactivator 3	**-1.4**	**2.1**			**3.8**
NCOA4	nuclear receptor coactivator 4	**1.9**	**1.3**	**1.5**		**-1.4**
NCSTN	nicastrin	**1.9**				
NDRG1	N-myc downstream regulated gene 1	**9.2**	**3.1**	**1.6**		**2.3**
NDUFA5	NADH dehydrogenase (ubiquinone) 1 alpha subcomplex, 5, 13kDa	**-2.4**				**1.9**
NDUFA9	NADH dehydrogenase (ubiquinone) 1 alpha subcomplex, 9, 39kDa	**-2.1**				
NDUFAB1	NADH dehydrogenase (ubiquinone) 1, alpha/beta subcomplex, 1, 8kDa	**-1.9**	**-1.5**			
NDUFAF1	NADH dehydrogenase (ubiquinone) 1 alpha subcomplex, assembly factor 1	**-3.0**	**-1.8**		**-1.2**	**2.5**
NDUFB3	NADH dehydrogenase (ubiquinone) 1 beta subcomplex, 3, 12kDa	**1.2**				**-1.8**
NDUFB5	NADH dehydrogenase (ubiquinone) 1 beta subcomplex, 5, 16kDa	**1.7**				**-1.8**
NDUFB7	NADH dehydrogenase (ubiquinone) 1 beta subcomplex, 7, 18kDa	**-1.3**	**1.9**			
NDUFB8	NADH dehydrogenase (ubiquinone) 1 beta subcomplex, 8, 19kDa	**1.2**				**-2.1**
NDUFC1	NADH dehydrogenase (ubiquinone) 1, subcomplex unknown, 1, 6kDa	**1.4**				**-2.0**
NDUFS1	NADH dehydrogenase (ubiquinone) Fe-S protein 1, 75kDa (NADH-coenzyme Q reductase)	**-1.6**	**-1.5**			**1.4**
NDUFS2	NADH dehydrogenase (ubiquinone) Fe-S protein 2, 49kDa (NADH-coenzyme Q reductase)	**1.9**				**-1.7**
NDUFS5	NADH dehydrogenase (ubiquinone) Fe-S protein 5, 15kDa (NADH-coenzyme Q reductase)	**-1.9**	**-1.5**			
NDUFS6	NADH dehydrogenase (ubiquinone) Fe-S protein 6, 13kDa (NADH-coenzyme Q reductase)	**-1.5**				**-1.8**
NDUFV1	NADH dehydrogenase (ubiquinone) flavoprotein 1, 51kDa	**-1.6**	**-1.4**			**-2.0**
NECAP1	NECAP endocytosis associated 1	**-1.3**	**-1.3**			**1.6**
NEDD4	neural precursor cell expressed, developmentally down-regulated 4	**2.8**				**-3.0**
NEK1	NIMA (never in mitosis gene a)-related kinase 1	**-1.5**	**-2.4**			
NEK2	NIMA (never in mitosis gene a)-related kinase 2	**-1.9**	**1.5**			**2.1**
NF2	neurofibromin 2 (bilateral acoustic neuroma)	**-1.3**				
NFATC2IP	nuclear factor of activated T-cells, cytoplasmic, calcineurin-dependent 2 interacting protein	**-2.4**	**-1.3**			
NFATC3	nuclear factor of activated T-cells, cytoplasmic, calcineurin-dependent 3	**-1.8**	**-1.4**	**-1.4**	**-1.9**	
NFE2L1	nuclear factor (erythroid-derived 2)-like 1	**1.6**			**-1.4**	**-1.5**
NFE2L2	nuclear factor (erythroid-derived 2)-like 2	**2.2**	**1.3**			**-2.1**
NFIC	nuclear factor I/C (CCAAT-binding transcription factor)	**5.9**	**-1.9**			**-8.4**
NFIL3	nuclear factor, interleukin 3 regulated	**-2.5**	**5.0**	**6.0**	**3.6**	**19.1**
NFKB2	nuclear factor of kappa light polypeptide gene enhancer in B-cells 2 (p49/p100)	**-1.5**				**1.7**
NFKBIA	nuclear factor of kappa light polypeptide gene enhancer in B-cells inhibitor, alpha	**1.9**	**2.5**	**3.0**	**2.8**	**5.5**

NFRKB	nuclear factor related to kappaB binding protein	**2.2**				**-6.3**
NFYA	nuclear transcription factor Y, alpha	**1.6**				**-1.9**
NFYB	nuclear transcription factor Y, beta	**-1.6**				**1.3**
NFYC	nuclear transcription factor Y, gamma	**-1.4**	**-1.3**			
NKTR	natural killer-tumor recognition sequence	**2.0**				
NMB	neuromedin B	**1.4**				**-2.0**
NME6	non-metastatic cells 6, protein expressed in (nucleoside-diphosphate kinase)	**1.6**				**-1.6**
NMI	N-myc (and STAT) interactor	**1.2**				**-1.8**
NNT	nicotinamide nucleotide transhydrogenase	**1.3**				**-1.3**
NOLA2	nucleolar protein family A, member 2 (H/ACA small nucleolar RNPs)	**-1.5**	**-1.8**			**-2.0**
NOLC1	nucleolar and coiled-body phosphoprotein 1	**1.5**	**-2.0**	**-2.1**		**-3.9**
NPC2	Niemann-Pick disease, type C2	**1.8**		**1.3**		
NPM1	nucleophosmin (nucleolar phosphoprotein B23, numatrin)	**1.2**	**-1.9**			**-2.6**
NPM3	nucleophosmin/nucleoplasmin, 3	**2.5**		**-1.4**		**-4.8**
NQO2	NAD(P)H dehydrogenase, quinone 2	**2.2**	**-1.5**			**-3.4**
NR2C1	nuclear receptor subfamily 2, group C, member 1	**1.6**	**1.3**			
NR3C1	nuclear receptor subfamily 3, group C, member 1 (glucocorticoid receptor)	**-1.8**	**3.1**	**4.2**	**2.1**	**6.5**
NRAS	neuroblastoma RAS viral (v-ras) oncogene homolog	**-1.4**				**1.8**
NRD1	nardilysin (N-arginine dibasic convertase)	**1.7**				**-1.6**
NRIP1	nuclear receptor interacting protein 1	**-1.4**	**1.5**			
NSF	N-ethylmaleimide-sensitive factor	**1.4**	**-1.3**			**-1.8**
NSFL1C	NSFL1 (p97) cofactor (p47)	**1.5**	**1.5**			**-2.2**
NSMAF	neutral sphingomyelinase (N-SMase) activation associated factor	**-1.3**	**-2.7**			
NSUN5C	NOL1/NOP2/Sun domain family, member 5C	**-1.7**				**1.2**
NT5C2	5'-nucleotidase, cytosolic II	**2.3**	**1.5**			**-1.5**
NTHL1	nth endonuclease III-like 1 (E. coli)	**-2.2**	**-1.7**	**-1.7**		
NUBP1	nucleotide binding protein 1 (MinD homolog, E. coli)	**-1.9**				
NUCB2	nucleobindin 2	**1.3**	**-1.3**			**-3.1**
NUDC	nuclear distribution gene C homolog (A. nidulans)	**-1.2**	**-1.5**			**-2.1**
NUDT1	nudix (nucleoside diphosphate linked moiety X)-type motif 1	**-2.3**				**1.8**
NUDT3	nudix (nucleoside diphosphate linked moiety X)-type motif 3	**1.9**	**-1.2**			**-3.0**
NUFIP1	nuclear fragile X mental retardation protein interacting protein 1	**-1.8**	**-1.8**			**-2.2**
NUMA1	nuclear mitotic apparatus protein 1	**-2.1**				**1.9**
NUP153	nucleoporin 153kDa	**-2.0**	**-1.4**	**-1.5**		**1.4**
NUP155	nucleoporin 155kDa	**-1.5**				**-2.3**
NUP205	nucleoporin 205kDa	**-1.2**	**-1.5**			**-1.3**
NUP210	nucleoporin 210kDa	**-3.4**	**-1.7**			**-1.5**
NUP50	nucleoporin 50kDa	**-1.3**				
NUP62	nucleoporin 62kDa	**-1.4**		**-1.4**		**-2.6**
NUP88	nucleoporin 88kDa	**-2.2**				
NUP98	nucleoporin 98kDa	**-1.7**	**-2.0**		**-1.3**	**1.4**
NUPL1	nucleoporin like 1	**-2.9**				

NUPL2	nucleoporin like 2	3.8				-3.5
NUTF2	nuclear transport factor 2	-1.5	-1.6			
NXF1	nuclear RNA export factor 1	1.3				
OAS2	2'-5'-oligoadenylate synthetase 2, 69/71kDa	-3.2	-2.8			2.9
OAZ1	ornithine decarboxylase antizyme 1	-1.3	1.4			1.3
OAZIN	antizyme inhibitor 1	-2.2				1.9
ODC1	ornithine decarboxylase 1	-1.2	-1.5	-2.1	-2.2	-6.6
OGDH	oxoglutarate (alpha-ketoglutarate) dehydrogenase (lipoamide)	1.8	-1.7			-2.7
OGG1	8-oxoguanine DNA glycosylase	-3.9			1.4	6.2
OGT	O-linked N-acetylglucosamine (GlcNAc) transferase (UDP-N-acetylglucosamine:polypeptide-N-acetylglucosaminyl transferase)	2.0	1.3		2.0	2.3
OIP5	Opa interacting protein 5	-2.6				1.7
OPA1	optic atrophy 1 (autosomal dominant)	1.5				-1.6
ORC1L	origin recognition complex, subunit 1-like (yeast)	1.7				-22.9
ORC2L	origin recognition complex, subunit 2-like (yeast)	2.6				-3.0
ORC3L	origin recognition complex, subunit 3-like (yeast)	1.3				-1.8
ORC5L	origin recognition complex, subunit 5-like (yeast)	1.3		-1.3	-1.5	-1.9
OSBP	oxysterol binding protein	1.4				1.7
OSBPL8	oxysterol binding protein-like 8	-1.3				1.4
OSTF1	osteoclast stimulating factor 1	-1.3	1.2			1.6
OXSR1	oxidative-stress responsive 1	1.3				-1.7
P2RX4	purinergic receptor P2X, ligand-gated ion channel, 4	2.0				
P2RX5	purinergic receptor P2X, ligand-gated ion channel, 5	-2.0	5.5		2.7	6.6
P4HA1	procollagen-proline, 2-oxoglutarate 4-dioxygenase (proline 4-hydroxylase), alpha polypeptide I	3.0		-1.3		-1.5
P4HB	procollagen-proline, 2-oxoglutarate 4-dioxygenase (proline 4-hydroxylase), beta polypeptide	3.7			1.5	-3.0
PABPC1	poly(A) binding protein, cytoplasmic 1	-2.2	-1.4			1.7
PABPN1	poly(A) binding protein, nuclear 1	1.6	-1.3			-1.5
PAFAH1B1	platelet-activating factor acetylhydrolase, isoform Ib, alpha subunit 45kDa	-2.4	1.2			2.9
PAICS	phosphoribosylaminoimidazole carboxylase, phosphoribosylaminoimidazole succinocarboxamide synthetase	1.7	-3.3	-1.7		-12.5
PAI-RBP1	SERPINE1 mRNA binding protein 1	-1.5	-1.9	-1.6		-2.2
PAK1	p21/Cdc42/Rac1-activated kinase 1 (STE20 homolog, yeast)	2.5	-1.3	1.5		-3.4
PAK2	p21 (CDKN1A)-activated kinase 2	-1.6	1.5	-1.2		2.0
PAM	peptidylglycine alpha-amidating monooxygenase	1.3	-1.4	1.5		-1.6
PAPOLA	poly(A) polymerase alpha	1.4				-1.4
PARD3	par-3 partitioning defective 3 homolog (C. elegans)	2.5				3.0
PARN	poly(A)-specific ribonuclease (deadenylation nuclease)	-2.1				2.2
PARP1	poly (ADP-ribose) polymerase family, member 1	-2.5	1.7			2.2
PAWR	PRKC, apoptosis, WT1, regulator	42.6	-2.4	-1.2		-46.4
PAX8	paired box gene 8	3.7				-3.2
PAXIP1L	PAX interacting (with transcription-activation domain) protein 1	-3.5	1.2	1.4	1.3	12.7
PBEF1	pre-B-cell colony enhancing factor 1	1.7				-1.6
PBX3	pre-B-cell leukemia transcription factor 3	-172.9	-1.5			135.1
PC4	SUB1 homolog (S. cerevisiae)	2.4	-1.4			-4.6

PCBP2	poly(rC) binding protein 2	**2.0**				**-1.9**
PCCB	propionyl Coenzyme A carboxylase, beta polypeptide	**1.3**				**-1.7**
PCF11	PCF11, cleavage and polyadenylation factor subunit, homolog (S. cerevisiae)	**-2.1**				**2.6**
PCGF4	B lymphoma Mo-MLV insertion region (mouse)	**-1.5**	**1.3**			**1.2**
PCID1	PCI domain containing 1 (herpesvirus entry mediator)	**1.2**				**-1.6**
PCM1	pericentriolar material 1	**2.3**	**1.8**			**-1.9**
PCMT1	protein-L-isoaspartate (D-aspartate) O-methyltransferase	**-1.3**	**1.4**			**1.4**
PCNA	proliferating cell nuclear antigen	**-1.7**	**-1.6**			**-3.7**
PCNT2	pericentrin (kendrin)	**1.4**				
PCTK1	PCTAIRE protein kinase 1	**-1.4**				
PCTK2	PCTAIRE protein kinase 2	**-1.2**				**1.8**
PDAP1	PDGFA associated protein 1	**-3.1**				**3.2**
PDCD2	programmed cell death 2	**2.0**	**1.3**			**-2.0**
PDCD6	programmed cell death 6	**-11.8**	**2.0**			**20.7**
PDCL	phosducin-like	**-1.7**				
PDE3B	phosphodiesterase 3B, cGMP-inhibited	**6.1**	**-1.4**			**-25.2**
PDE6D	phosphodiesterase 6D, cGMP-specific, rod, delta	**-2.1**	**1.2**			**2.3**
PDE8A	phosphodiesterase 8A	**-1.5**	**1.6**			**1.6**
PDHX	pyruvate dehydrogenase complex, component X	**-1.5**				**1.2**
PDIA3	protein disulfide isomerase family A, member 3	**-1.3**				**-1.2**
PDIA4	protein disulfide isomerase family A, member 4	**-1.2**				**-1.7**
PDIA6	protein disulfide isomerase family A, member 6	**-1.5**	**-1.3**			**-1.7**
PDK1	pyruvate dehydrogenase kinase, isozyme 1	**2.8**				**-5.8**
PDK2	pyruvate dehydrogenase kinase, isozyme 2	**3.1**			**1.2**	
PDK3	pyruvate dehydrogenase kinase, isozyme 3	**-1.3**	**-1.6**			
PDLIM5	PDZ and LIM domain 5	**1.6**	**-1.6**			
PDPK1	3-phosphoinositide dependent protein kinase-1	**-1.8**				**2.3**
PDXK	pyridoxal (pyridoxine, vitamin B6) kinase	**2.8**				**-4.7**
PEA15	phosphoprotein enriched in astrocytes 15	**-1.3**				
PEMT	phosphatidylethanolamine N-methyltransferase	**-3.7**				
PER2	period homolog 2 (Drosophila)	**-2.0**	**-1.7**			
PEX11B	peroxisomal biogenesis factor 11B	**-1.4**	**1.4**			**1.9**
PEX14	peroxisomal biogenesis factor 14	**1.3**			**-1.7**	**-1.3**
PEX3	peroxisomal biogenesis factor 3	**1.3**	**-2.4**			**-2.3**
PFAS	phosphoribosylformylglycinamidine synthase (FGAR amidotransferase)	**-2.4**	**-2.7**			**-2.3**
PFDN5	prefoldin subunit 5	**1.4**	**1.4**			
PFKP	phosphofructokinase, platelet	**1.5**	**1.7**			**-1.6**
PFN1	profilin 1	**-2.1**				**1.8**
PGAM1	phosphoglycerate mutase 1 (brain)	**1.5**	**-1.2**	**-1.2**		**-2.4**
PGD	phosphogluconate dehydrogenase	**1.8**	**-1.3**			**-2.1**
PGGT1B	protein geranylgeranyltransferase type I, beta subunit	**-3.2**				**3.9**
PGK1	phosphoglycerate kinase 1	**1.3**	**-1.9**			**-2.6**

PGM1	phosphoglucomutase 1	**1.7**	**-1.8**			**-3.2**
PGM3	phosphoglucomutase 3	**-2.6**				
PGRMC2	progesterone receptor membrane component 2	**1.7**		**-1.2**		**-2.6**
PHB2	prohibitin 2	**-1.7**				**1.5**
PHC2	polyhomeotic homolog 2 (Drosophila)	**-1.6**				**1.7**
PHF1	PHD finger protein 1	**1.8**				**-1.6**
PHF21A	PHD finger protein 21A	**2.0**				**-1.5**
PHGDH	phosphoglycerate dehydrogenase	**-1.7**	**-2.4**			**-2.3**
PHKA1	phosphorylase kinase, alpha 1 (muscle)	**3.0**				**-4.3**
PHKA2	phosphorylase kinase, alpha 2 (liver)	**1.7**	**-1.3**			
PHKB	phosphorylase kinase, beta	**-1.7**	**1.4**			**2.0**
PIAS1	protein inhibitor of activated STAT, 1	**1.7**	**1.4**			**2.2**
PICALM	phosphatidylinositol binding clathrin assembly protein	**2.1**	**2.0**	**1.7**	**1.5**	**2.1**
PIG8	centrosomal protein 57kDa	**1.6**				**-2.3**
PIGC	phosphatidylinositol glycan anchor biosynthesis, class C	**1.9**	**2.5**			**-1.3**
PIK3C2A	phosphoinositide-3-kinase, class 2, alpha polypeptide	**2.5**	**1.7**			**1.4**
PIK3CA	phosphoinositide-3-kinase, catalytic, alpha polypeptide	**-2.5**	**1.3**			**3.6**
PIK3CB	phosphoinositide-3-kinase, catalytic, beta polypeptide	**3.3**	**1.2**			**-2.7**
PIK3R1	phosphoinositide-3-kinase, regulatory subunit 1 (p85 alpha)	**2.1**		**2.5**		
PIK3R4	phosphoinositide-3-kinase, regulatory subunit 4, p150	**1.3**				**-1.3**
PINK1	PTEN induced putative kinase 1	**1.3**		**1.6**		**2.2**
PIP5K2A	phosphatidylinositol-4-phosphate 5-kinase, type II, alpha	**1.3**				
PIP5K3	phosphatidylinositol-3-phosphate/phosphatidylinositol 5-kinase, type III	**-1.6**				**1.3**
PITPNA	phosphatidylinositol transfer protein, alpha	**-2.0**	**-1.3**			**2.0**
PITPNB	phosphatidylinositol transfer protein, beta	**-1.5**				**-1.3**
PITRM1	pitrilysin metallopeptidase 1	**1.6**		**1.8**		**-2.0**
PKD2	polycystic kidney disease 2 (autosomal dominant)	**-2.0**				**2.5**
PKIA	protein kinase (cAMP-dependent, catalytic) inhibitor alpha	**-6.9**		**1.5**	**-1.5**	**7.8**
PKM2	pyruvate kinase, muscle	**-3.2**	**-2.3**			
PKMYT1	protein kinase, membrane associated tyrosine/threonine 1	**-2.2**	**1.2**			
PKN2	protein kinase N2	**1.4**	**1.4**			**1.3**
PKP4	plakophilin 4	**2.5**				**-2.9**
PLAG1	pleiomorphic adenoma gene 1	**-5.2**	**1.4**			**11.9**
PLAGL1	pleiomorphic adenoma gene-like 1	**15.8**		**1.6**		**-13.0**
PLCB1	phospholipase C, beta 1 (phosphoinositide-specific)	**-7.4**	**-1.5**			**4.0**
PLCG1	phospholipase C, gamma 1	**1.9**	**1.9**			
PLCG2	phospholipase C, gamma 2 (phosphatidylinositol-specific)	**-1.3**	**-1.6**			**-1.5**
PLCL2	phospholipase C-like 2	**-1.6**	**1.7**			
PLEKHB2	pleckstrin homology domain containing, family B (evectins) member 2	**1.3**	**1.3**			**-1.5**
PLEKHE1	PH domain and leucine rich repeat protein phosphatase	**-1.6**				**-1.3**
PLK1	polo-like kinase 1 (Drosophila)	**-1.6**	**1.5**			**2.3**
PLP2	proteolipid protein 2 (colonic epithelium-enriched)	**-3.3**	**1.5**			**8.3**

PLS1	plastin 1 (I isoform)	**9.1**	**-3.2**			**-21.7**
PLSCR1	phospholipid scramblase 1	**1.8**	**1.5**			**-1.4**
PLTP	phospholipid transfer protein	**13.4**				**-30.8**
PLXNB2	plexin B2	**-3.9**	**1.3**			**8.5**
PMAIP1	phorbol-12-myristate-13-acetate-induced protein 1	**-3.7**	**1.5**		**-2.1**	**2.3**
PMF1	polyamine-modulated factor 1	**-1.6**				
PML	promyelocytic leukemia	**1.8**				**1.4**
PMS1	PMS1 postmeiotic segregation increased 1 (S. cerevisiae)	**2.5**	**-1.3**			**-1.7**
PMS2L3	postmeiotic segregation increased 2-like 3	**-1.7**				**1.7**
PMVK	phosphomevalonate kinase	**-1.3**				**-1.4**
PNN	pinin, desmosome associated protein	**1.8**	**-1.5**		**-1.5**	**-2.6**
PODXL	podocalyxin-like	**-1.9**	**-2.8**	**-1.3**		**-1.4**
POLA	polymerase (DNA directed), alpha 1	**-1.5**	**-2.7**			**-2.7**
POLA2	polymerase (DNA directed), alpha 2 (70kD subunit)	**-1.4**	**1.3**			
POLB	polymerase (DNA directed), beta	**1.3**	**-1.5**			**-1.4**
POLD2	polymerase (DNA directed), delta 2, regulatory subunit 50kDa	**1.9**	**-1.9**			**-13.9**
POLD3	polymerase (DNA-directed), delta 3, accessory subunit	**-1.6**	**-1.8**			**-2.2**
POLE	polymerase (DNA directed), epsilon	**-1.4**				**-3.9**
POLE2	polymerase (DNA directed), epsilon 2 (p59 subunit)	**-1.8**	**-2.1**			**-2.0**
POLE3	polymerase (DNA directed), epsilon 3 (p17 subunit)	**-1.5**	**-2.0**			**-2.0**
POLG2	polymerase (DNA directed), gamma 2, accessory subunit	**1.7**	**-1.3**			**-2.6**
POLR2A	polymerase (RNA) II (DNA directed) polypeptide A, 220kDa	**-2.4**				**2.2**
POLR2B	polymerase (RNA) II (DNA directed) polypeptide B, 140kDa	**2.6**				**-3.0**
POLR2E	polymerase (RNA) II (DNA directed) polypeptide E, 25kDa	**-1.5**	**-1.9**			**-1.6**
POLR2G	polymerase (RNA) II (DNA directed) polypeptide G	**-1.7**	**-1.4**			
POLR2H	polymerase (RNA) II (DNA directed) polypeptide H	**1.4**	**-1.9**	**-1.6**		**-2.9**
POLR2I	polymerase (RNA) II (DNA directed) polypeptide I, 14.5kDa	**-1.6**	**-1.8**			**-1.7**
POLR2J	polymerase (RNA) II (DNA directed) polypeptide J, 13.3kDa	**-1.3**				
POLR3C	polymerase (RNA) III (DNA directed) polypeptide C (62kD)	**-1.8**				**-1.4**
POLR3G	polymerase (RNA) III (DNA directed) polypeptide G (32kD)	**1.3**	**-3.0**			**-3.8**
PON2	paraoxonase 2	**43.3**	**19.7**	**1.4**		**-40.8**
POP4	processing of precursor 4, ribonuclease P/MRP subunit (S. cerevisiae)	**-1.3**				
POU2AF1	POU domain, class 2, associating factor 1	**-56.3**			**-1.9**	**25.8**
PPARBP	PPAR binding protein	**1.8**	**-1.5**			**-2.0**
PPAT	phosphoribosyl pyrophosphate amidotransferase	**2.0**	**-2.0**	**-1.7**		**-4.7**
PPFIA1	protein tyrosine phosphatase, receptor type, f polypeptide (PTPRF), interacting protein (liprin), alpha 1	**1.3**				**-1.4**
PPIA	peptidylprolyl isomerase A (cyclophilin A)	**1.9**	**-1.2**			**-2.8**
PPIB	peptidylprolyl isomerase B (cyclophilin B)	**1.5**				**-1.7**
PPIF	peptidylprolyl isomerase F (cyclophilin F)	**-1.3**	**-2.6**			**-4.9**
PPIG	peptidylprolyl isomerase G (cyclophilin G)	**1.8**	**-1.3**			**-2.7**
PPM1A	protein phosphatase 1A (formerly 2C), magnesium-dependent, alpha isoform	**-1.7**	**1.5**			**1.9**
PPM1B	protein phosphatase 1B (formerly 2C), magnesium-dependent, beta isoform	**1.3**	**1.4**			**1.7**

PPP1CA	protein phosphatase 1, catalytic subunit, alpha isoform	**-2.1**				**1.5**
PPP1R10	protein phosphatase 1, regulatory subunit 10	**1.4**			**1.2**	
PPP1R11	protein phosphatase 1, regulatory (inhibitor) subunit 11	**-1.6**				**1.6**
PPP1R12A	protein phosphatase 1, regulatory (inhibitor) subunit 12A	**1.4**	**1.7**			**1.8**
PPP1R2	protein phosphatase 1, regulatory (inhibitor) subunit 2	**2.1**	**1.4**			**1.4**
PPP1R7	protein phosphatase 1, regulatory subunit 7	**-2.5**				**2.0**
PPP2CA	protein phosphatase 2 (formerly 2A), catalytic subunit, alpha isoform	**-1.4**				
PPP2CB	protein phosphatase 2 (formerly 2A), catalytic subunit, beta isoform	**1.5**	**1.2**			
PPP2R2A	protein phosphatase 2 (formerly 2A), regulatory subunit B (PR 52), alpha isoform	**2.4**	**1.7**			**-1.9**
PPP2R4	protein phosphatase 2A, regulatory subunit B' (PR 53)	**-1.2**				
PPP2R5A	protein phosphatase 2, regulatory subunit B (B56), alpha isoform	**-1.2**	**1.9**			**1.3**
PPP2R5C	protein phosphatase 2, regulatory subunit B (B56), gamma isoform	**-25.3**				**5.3**
PPP2R5E	protein phosphatase 2, regulatory subunit B (B56), epsilon isoform	**2.4**	**1.2**			**-4.4**
PPP3CB	protein phosphatase 3 (formerly 2B), catalytic subunit, beta isoform (calcineurin A beta)	**1.7**	**1.6**			**-1.8**
PPP4C	protein phosphatase 4 (formerly X), catalytic subunit	**-1.8**				**1.8**
PPP6C	protein phosphatase 6, catalytic subunit	**-1.8**				**1.3**
PPRC1	peroxisome proliferator-activated receptor gamma, coactivator-related 1	**1.7**	**-2.4**			**-4.7**
PPT1	palmitoyl-protein thioesterase 1 (ceroid-lipofuscinosis, neuronal 1, infantile)	**1.7**	**-1.3**			**-2.2**
PQBP1	polyglutamine binding protein 1	**1.3**	**1.3**			**-1.5**
PRDX1	peroxiredoxin 1	**-2.5**	**-1.7**	**-1.3**		
PRDX2	peroxiredoxin 2	**-8.5**	**-3.1**			**3.4**
PRDX4	peroxiredoxin 4	**1.3**	**-2.5**	**-1.2**		**-3.3**
PRDX6	peroxiredoxin 6	**1.5**	**1.4**			**-1.3**
PREI3	preimplantation protein 3	**1.5**	**1.3**			**-1.7**
PREP	prolyl endopeptidase	**1.5**				**-1.8**
PRG1	proteoglycan 1, secretory granule	**2.7**	**1.5**		**3.2**	**2.7**
PRIM1	primase, polypeptide 1, 49kDa	**-1.3**	**-1.8**			**-2.3**
PRIM2A	primase, polypeptide 2A, 58kDa	**1.4**				**-1.4**
PRKAA1	protein kinase, AMP-activated, alpha 1 catalytic subunit	**1.3**	**1.6**			
PRKAB1	protein kinase, AMP-activated, beta 1 non-catalytic subunit	**1.5**				
PRKAB2	protein kinase, AMP-activated, beta 2 non-catalytic subunit	**5.1**				**2.3**
PRKACB	protein kinase, cAMP-dependent, catalytic, beta	**2.4**	**-2.7**			**-1.6**
PRKAG1	protein kinase, AMP-activated, gamma 1 non-catalytic subunit	**1.2**				
PRKAR1A	protein kinase, cAMP-dependent, regulatory, type I, alpha (tissue specific extinguisher 1)	**2.0**	**1.4**			
PRKCA	protein kinase C, alpha	**6.6**	**1.3**	**1.6**		**-7.0**
PRKCB1	protein kinase C, beta 1	**20.7**	**1.6**			**-18.3**
PRKCBP1	protein kinase C binding protein 1	**-1.2**	**1.4**			**1.6**
PRKCH	protein kinase C, eta	**3.4**	**3.7**			**-2.4**
PRKCQ	protein kinase C, theta	**1.6**				**-2.4**
PRKD2	protein kinase D2	**-3.2**	**1.8**			**4.8**
PRKDC	protein kinase, DNA-activated, catalytic polypeptide	**-1.2**	**-1.6**			**-2.1**
PRKRA	protein kinase, interferon-inducible double stranded RNA dependent activator	**-1.9**	**2.5**			**5.0**

PRKX	protein kinase, X-linked	-2.3	3.4			5.3
PRMT3	protein arginine methyltransferase 3	-1.3	-2.4	-1.6	-2.1	-2.8
PRNP	prion protein (p27-30) (Creutzfeldt-Jakob disease, Gerstmann-Strausler-Scheinker syndrome, fatal familial insomnia)	-1.4				
PROCR	protein C receptor, endothelial (EPCR)	6.6	-1.5		-2.2	-12.1
PRPF19	PRP19/PSO4 pre-mRNA processing factor 19 homolog (S. cerevisiae)	-1.3	-1.9			-2.2
PRPF31	PRP31 pre-mRNA processing factor 31 homolog (S. cerevisiae)	-1.2			-1.6	-2.1
PRPF4	PRP4 pre-mRNA processing factor 4 homolog (yeast)	-1.3	-2.2			-2.1
PRPF8	PRP8 pre-mRNA processing factor 8 homolog (S. cerevisiae)	-1.4	-1.3			
PRPS1	phosphoribosyl pyrophosphate synthetase 1	-2.1	-2.6	-1.5		-2.3
PRPS2	phosphoribosyl pyrophosphate synthetase 2	1.3				-2.1
PRPSAP1	phosphoribosyl pyrophosphate synthetase-associated protein 1	1.9				-1.3
PRPSAP2	phosphoribosyl pyrophosphate synthetase-associated protein 2	-1.8				
PRSS25	HtrA serine peptidase 2	1.5	-1.5			-1.9
PRUNE	prune homolog (Drosophila)	-1.5	-1.9			
PSAP	prosaposin (variant Gaucher disease and variant metachromatic leukodystrophy)	3.1	-1.4			
PSEN1	presenilin 1 (Alzheimer disease 3)	1.3	1.5	1.7	1.5	2.0
PSF1	GINS complex subunit 1 (Psf1 homolog)	-1.9	-1.6			-2.3
PSIP1	PC4 and SFRS1 interacting protein 1	-2.0	-1.5			2.5
PSMA1	proteasome (prosome, macropain) subunit, alpha type, 1	-1.2				-1.3
PSMA2	proteasome (prosome, macropain) subunit, alpha type, 2	3.5	-1.3			-5.0
PSMA3	proteasome (prosome, macropain) subunit, alpha type, 3	1.3	-1.7			-1.7
PSMA6	proteasome (prosome, macropain) subunit, alpha type, 6	-1.3				1.3
PSMB2	proteasome (prosome, macropain) subunit, beta type, 2	-1.2	-1.6			-1.5
PSMB4	proteasome (prosome, macropain) subunit, beta type, 4	1.3	-1.4			-1.2
PSMB5	proteasome (prosome, macropain) subunit, beta type, 5	1.5	-1.4	-1.4	-1.4	-2.2
PSMB6	proteasome (prosome, macropain) subunit, beta type, 6	-1.6				
PSMB8	proteasome (prosome, macropain) subunit, beta type, 8 (large multifunctional peptidase 7)	-18.9				16.2
PSMB9	proteasome (prosome, macropain) subunit, beta type, 9 (large multifunctional peptidase 2)	-202.7	-1.3			22.2
PSMC3	proteasome (prosome, macropain) 26S subunit, ATPase, 3	-1.4				-1.5
PSMC5	proteasome (prosome, macropain) 26S subunit, ATPase, 5	1.6				-2.6
PSMC6	proteasome (prosome, macropain) 26S subunit, ATPase, 6	1.3				-1.4
PSMD1	proteasome (prosome, macropain) 26S subunit, non-ATPase, 1	-1.6	-1.4			
PSMD10	proteasome (prosome, macropain) 26S subunit, non-ATPase, 10	-1.2				-1.2
PSMD12	proteasome (prosome, macropain) 26S subunit, non-ATPase, 12	1.6				-2.2
PSMD14	proteasome (prosome, macropain) 26S subunit, non-ATPase, 14	1.3				-2.4
PSMD2	proteasome (prosome, macropain) 26S subunit, non-ATPase, 2	1.3				-1.8
PSMD4	proteasome (prosome, macropain) 26S subunit, non-ATPase, 4	1.3				-1.6
PSMD5	proteasome (prosome, macropain) 26S subunit, non-ATPase, 5	3.0				-2.5
PSMD8	proteasome (prosome, macropain) 26S subunit, non-ATPase, 8	-1.5	-1.3			
PSME1	proteasome (prosome, macropain) activator subunit 1 (PA28 alpha)	-1.9	-1.8			
PSME2	proteasome (prosome, macropain) activator subunit 2 (PA28 beta)	-2.4	-1.8			
PSME3	proteasome (prosome, macropain) activator subunit 3 (PA28 gamma; Ki)	1.5	-2.2		-1.4	-2.6

PSPH	phosphoserine phosphatase	3.3	-2.0			-8.6
PTEN	phosphatase and tensin homolog (mutated in multiple advanced cancers 1)	4.2	-1.3	2.3		-4.7
PTK2B	PTK2B protein tyrosine kinase 2 beta	-1.3		2.1	4.2	1.9
PTMA	prothymosin, alpha (gene sequence 28)	-1.7	-1.3			
PTP4A1	protein tyrosine phosphatase type IVA, member 1	-1.7	-1.8			-1.9
PTP4A2	protein tyrosine phosphatase type IVA, member 2	1.2	1.5			-1.9
PTPLB	protein tyrosine phosphatase-like (proline instead of catalytic arginine), member b	2.2	2.3			2.2
PTPN11	protein tyrosine phosphatase, non-receptor type 11 (Noonan syndrome 1)	1.3				-1.3
PTPN12	protein tyrosine phosphatase, non-receptor type 12	-10.9	2.4	1.3		75.8
PTPN2	protein tyrosine phosphatase, non-receptor type 2	1.3	-1.2	-1.3	-2.1	-1.6
PTPN22	protein tyrosine phosphatase, non-receptor type 22 (lymphoid)	2.9				-8.4
PTPN4	protein tyrosine phosphatase, non-receptor type 4 (megakaryocyte)	-1.6	-1.6			
PTPN6	protein tyrosine phosphatase, non-receptor type 6	-5.5		1.3		7.8
PTPN7	protein tyrosine phosphatase, non-receptor type 7	3.3	-1.5			-4.7
PTPRA	protein tyrosine phosphatase, receptor type, A	1.3				
PTPRC	protein tyrosine phosphatase, receptor type, C	-6.4				11.4
PTPRCAP	protein tyrosine phosphatase, receptor type, C-associated protein	-2.1	1.5			3.6
PTPRF	protein tyrosine phosphatase, receptor type, F	91.0				-122.2
PTPRM	protein tyrosine phosphatase, receptor type, M	8.5		3.8	13.6	-19.3
PTS	6-pyruvoyltetrahydropterin synthase	-2.4	-1.3	-1.6		1.8
PTTG1	pituitary tumor-transforming 1	-2.1				1.4
PTTG1IP	pituitary tumor-transforming 1 interacting protein	1.4	1.5			
PUM2	pumilio homolog 2 (Drosophila)	-1.4				1.4
PURA	purine-rich element binding protein A	-1.3				1.3
PXMP3	peroxisomal membrane protein 3, 35kDa (Zellweger syndrome)	-1.7	1.7		4.3	2.6
QDPR	quinoid dihydropteridine reductase	2.2				-3.8
QKI	quaking homolog, KH domain RNA binding (mouse)	2.7	2.5		-1.5	-1.7
QPRT	quinolinate phosphoribosyltransferase (nicotinate-nucleotide pyrophosphorylase (carboxylating))	-2.1	-3.6			1.4
RAB11A	RAB11A, member RAS oncogene family	-2.2	1.3			1.5
RAB11B	RAB11B, member RAS oncogene family	-1.4				
RAB14	RAB14, member RAS oncogene family	1.3	1.3			
RAB1A	RAB1A, member RAS oncogene family	1.5	1.3			-1.3
RAB2	RAB2, member RAS oncogene family	-1.9				2.1
RAB27A	RAB27A, member RAS oncogene family	2.2	-1.8			-3.5
RAB31	RAB31, member RAS oncogene family	3.8				-13.0
RAB3GAP	RAB3 GTPase activating protein subunit 1 (catalytic)	1.8	-1.5			
RAB3-GAP150	RAB3 GTPase activating protein subunit 2 (non-catalytic)	1.3				
RAB40B	RAB40B, member RAS oncogene family	-1.7		-1.3		-1.5
RAB4A	RAB4A, member RAS oncogene family	3.8				-4.7
RAB5A	RAB5A, member RAS oncogene family	1.2	1.3			
RAB5C	RAB5C, member RAS oncogene family	1.2				
RAB6A	RAB6A, member RAS oncogene family	-1.4	1.5			1.7

RAB6IP2	ELKS/RAB6-interacting/CAST family member 1	**-1.3**		**1.8**		**5.3**
RAB7L1	RAB7, member RAS oncogene family-like 1	**2.1**	**-1.4**			**-2.9**
RAB9A	RAB9, member RAS oncogene family	**1.3**				
RAB9P40	Rab9 effector protein with kelch motifs	**1.9**	**-3.7**	**-1.8**	**-2.1**	**-12.1**
RABAC1	Rab acceptor 1 (prenylated)	**-1.5**				**3.1**
RABEP1	rabaptin, RAB GTPase binding effector protein 1	**-2.1**				**2.4**
RABGGTB	Rab geranylgeranyltransferase, beta subunit	**2.1**	**-1.8**	**-1.3**	**-1.7**	**-1.8**
RABIF	RAB interacting factor	**-1.2**				**1.5**
RAC1	ras-related C3 botulinum toxin substrate 1 (rho family, small GTP binding protein Rac1)	**-1.9**	**1.4**			**2.0**
RAC2	ras-related C3 botulinum toxin substrate 2 (rho family, small GTP binding protein Rac2)	**-2.7**				**1.3**
RAD1	RAD1 homolog (S. pombe)	**-1.6**	**-1.7**			**-3.2**
RAD21	RAD21 homolog (S. pombe)	**-2.3**	**1.3**			**2.5**
RAD23B	RAD23 homolog B (S. cerevisiae)	**-1.3**				
RAD51AP1	RAD51 associated protein 1	**-2.8**	**-1.4**			**-1.3**
RAE1	RAE1 RNA export 1 homolog (S. pombe)	**-1.3**	**-1.4**			**-1.4**
RAF1	v-raf-1 murine leukemia viral oncogene homolog 1	**-1.3**				
RAFTLIN	raftlin, lipid raft linker 1	**-1.5**	**-1.5**			
RAG1	recombination activating gene 1	**-32.2**		**-3.6**	**-4.2**	**14.5**
RAG2	recombination activating gene 2	**-7.4**				**6.4**
RALA	v-ral simian leukemia viral oncogene homolog A (ras related)	**1.7**	**-1.3**			**-2.1**
RALBP1	ralA binding protein 1	**-1.8**	**-1.4**			**1.2**
RALY	RNA binding protein, autoantigenic (hnRNP-associated with lethal yellow homolog (mouse))	**-1.4**				**1.2**
RAN	RAN, member RAS oncogene family	**-1.2**	**-1.2**			**-1.4**
RANBP1	RAN binding protein 1	**-1.9**				**-2.8**
RANBP2	RAN binding protein 2	**2.5**	**-1.4**			**-1.9**
RANBP5	RAN binding protein 5	**-1.3**	**-2.0**		**-1.5**	**-2.3**
RANBP9	RAN binding protein 9	**-1.6**	**-1.3**			**2.1**
RAP1A	RAP1A, member of RAS oncogene family	**2.0**				**-2.2**
RAP1GDS1	RAP1, GTP-GDP dissociation stimulator 1	**-1.5**				**2.0**
RAP2A	RAP2A, member of RAS oncogene family	**-1.4**	**1.6**			**4.0**
RAPGEF2	Rap guanine nucleotide exchange factor (GEF) 2	**1.8**	**2.0**			**4.2**
RASA1	RAS p21 protein activator (GTPase activating protein) 1	**1.9**	**2.3**	**1.9**	**2.0**	**3.5**
RASA4	RAS p21 protein activator 4	**2.0**				**-3.4**
RASGRP1	RAS guanyl releasing protein 1 (calcium and DAG-regulated)	**-111.1**	**-7.2**			
RASGRP2	RAS guanyl releasing protein 2 (calcium and DAG-regulated)	**-2.3**	**2.1**			**4.4**
RASSF2	Ras association (RalGDS/AF-6) domain family 2	**-6.4**	**-2.5**			**3.5**
RB1	retinoblastoma 1 (including osteosarcoma)	**-2.6**	**1.5**			**3.1**
RB1CC1	RB1-inducible coiled-coil 1	**-1.4**	**1.7**			**1.7**
RBBP4	retinoblastoma binding protein 4	**-1.3**	**-1.4**			**-1.5**
RBBP5	retinoblastoma binding protein 5	**-1.3**				**1.3**
RBBP6	retinoblastoma binding protein 6	**-1.8**				**2.5**
RBBP8	retinoblastoma binding protein 8	**-1.9**	**-1.7**	**-1.3**		**-1.5**

RBL1	retinoblastoma-like 1 (p107)	**-1.8**				
RBL2	retinoblastoma-like 2 (p130)	**1.5**	**1.5**			**-1.3**
RBM4	RNA binding motif protein 4	**1.7**	**1.3**			
RBM4B	RNA binding motif protein 4B	**-1.7**				
RBM5	RNA binding motif protein 5	**1.6**		**1.4**	**1.3**	**2.2**
RBM8A	RNA binding motif protein 8A	**2.3**	**1.5**			**-3.3**
RBMS1	RNA binding motif, single stranded interacting protein 1	**3.0**	**4.3**			**2.3**
RBMX	RNA binding motif protein, X-linked	**1.4**				**-1.9**
RBPSUH	recombining binding protein suppressor of hairless (Drosophila)	**1.9**	**1.4**			**1.2**
RCHY1	ring finger and CHY zinc finger domain containing 1	**1.5**				**-1.6**
RCN1	reticulocalbin 1, EF-hand calcium binding domain	**-1.5**	**1.2**	**1.5**		**2.3**
RCN2	reticulocalbin 2, EF-hand calcium binding domain	**1.4**	**2.0**			
RCOR1	REST corepressor 1	**-1.3**				**1.3**
RCP9	calcitonin gene-related peptide-receptor component protein	**-1.5**	**-1.5**			
RDBP	RD RNA binding protein	**-1.2**				**-1.6**
RDH11	retinol dehydrogenase 11 (all-trans/9-cis/11-cis)	**-1.3**	**-1.8**			**-1.6**
RECQL	RecQ protein-like (DNA helicase Q1-like)	**-1.4**		**1.6**		**1.4**
REL	v-rel reticuloendotheliosis viral oncogene homolog (avian)	**2.4**	**1.5**		**2.4**	**-2.1**
REV3L	REV3-like, catalytic subunit of DNA polymerase zeta (yeast)	**-2.1**	**1.5**			**2.2**
RFC1	replication factor C (activator 1) 1, 145kDa	**-1.2**	**-1.4**	**1.5**		**-2.1**
RFC2	replication factor C (activator 1) 2, 40kDa	**-3.9**	**-1.5**			**-1.5**
RFC3	replication factor C (activator 1) 3, 38kDa	**-2.1**	**-1.5**			**-1.5**
RFC5	replication factor C (activator 1) 5, 36.5kDa	**-1.3**	**-1.7**			**-2.9**
RFK	riboflavin kinase	**-1.9**	**-2.1**			**-1.3**
RFX5	regulatory factor X, 5 (influences HLA class II expression)	**-1.6**				**-1.6**
RGS10	regulator of G-protein signalling 10	**19.3**	**-2.5**			**-19.8**
RGS19	regulator of G-protein signalling 19	**-1.8**	**-1.3**	**-1.3**	**-1.6**	**2.2**
RHEB	Ras homolog enriched in brain	**-1.6**	**1.9**			**2.9**
RHOA	ras homolog gene family, member A	**-1.4**	**1.4**	**1.2**		**1.4**
RHOC	ras homolog gene family, member C	**3.0**	**3.2**			**2.3**
RHOG	ras homolog gene family, member G (rho G)	**1.9**				**-1.6**
RHOH	ras homolog gene family, member H	**-15.8**	**1.3**			**38.1**
RHOQ	ras homolog gene family, member Q	**1.8**	**1.3**			**-1.7**
RIF1	RAP1 interacting factor homolog (yeast)	**2.0**	**-1.3**			**-6.8**
RIMS3	regulating synaptic membrane exocytosis 3	**-6.8**				**12.1**
RIOK3	RIO kinase 3 (yeast)	**1.7**				**1.5**
RIPK1	receptor (TNFRSF)-interacting serine-threonine kinase 1	**1.5**				
RIT1	Ras-like without CAAX 1	**-1.4**	**1.8**			**2.1**
RNF13	ring finger protein 13	**1.3**	**-1.5**			
RNF139	ring finger protein 139	**1.4**				
RNF144	ring finger protein 144	**3.5**				**-4.3**
RNF6	ring finger protein (C3H2C3 type) 6	**-2.2**	**1.2**			**2.2**

RNGTT	RNA guanylyltransferase and 5'-phosphatase	**-1.6**				
RNH	ribonuclease/angiogenin inhibitor 1	**2.4**	**-1.5**		**-1.3**	**-4.2**
RNMT	RNA (guanine-7-) methyltransferase	**1.3**				**-1.5**
RNPC2	RNA binding motif protein 39	**1.8**	**2.3**			**1.3**
RNPS1	RNA binding protein S1, serine-rich domain	**-5.0**	**-1.2**			**2.5**
RNUT1	snurportin 1	**1.3**				**-1.5**
RPA1	replication protein A1, 70kDa	**-2.8**	**-1.6**			**1.5**
RPA3	replication protein A3, 14kDa	**-2.0**				
RPE	ribulose-5-phosphate-3-epimerase	**-2.1**	**-1.5**			**1.4**
RPGR	retinitis pigmentosa GTPase regulator	**6.5**		**-1.6**		
RPL12	ribosomal protein L12	**1.5**				
RPL13	ribosomal protein L13	**1.4**				**-3.0**
RPL13A	ribosomal protein L13a	**1.4**				**-1.2**
RPL15	ribosomal protein L15	**1.5**	**2.1**			**-1.4**
RPL17	ribosomal protein L17	**2.1**	**-1.8**			**-2.3**
RPL18A	ribosomal protein L18a	**1.4**	**1.3**			
RPL22	ribosomal protein L22	**1.2**				
RPL23	ribosomal protein L23	**1.3**	**-1.3**			
RPL29	ribosomal protein L29	**2.0**	**1.4**			**-1.5**
RPL3	ribosomal protein L3	**1.3**				
RPL31	ribosomal protein L31	**2.9**	**1.3**			**-3.4**
RPL35	ribosomal protein L35	**2.2**	**2.4**			**-1.8**
RPL37	ribosomal protein L37	**1.6**	**-1.5**			**-1.8**
RPL38	ribosomal protein L38	**2.0**				**-1.5**
RPL4	ribosomal protein L4	**1.3**				
RPL5	ribosomal protein L5	**1.3**	**1.2**			
RPLP2	ribosomal protein, large, P2	**1.4**				**-1.7**
RPN1	ribophorin I	**1.5**				**-2.3**
RPN2	ribophorin II	**1.7**	**-1.5**			**-2.6**
RPP14	ribonuclease P 14kDa subunit	**1.9**	**-1.7**			**-2.7**
RPP38	ribonuclease P/MRP 38kDa subunit	**1.7**	**-1.5**	**-1.2**		**-1.8**
RPS10	ribosomal protein S10	**1.3**	**1.4**			
RPS14	ribosomal protein S14	**1.5**	**1.6**			
RPS15A	ribosomal protein S15a	**1.5**				**1.2**
RPS17	ribosomal protein S17	**1.5**	**1.5**			
RPS18	ribosomal protein S18	**2.2**				
RPS19	ribosomal protein S19	**1.3**	**-1.6**			
RPS2	ribosomal protein S2	**2.6**				**-1.9**
RPS21	ribosomal protein S21	**3.1**	**1.2**			**-3.3**
RPS23	ribosomal protein S23	**2.0**				**-2.1**
RPS24	ribosomal protein S24	**2.2**				**-3.2**
RPS28	ribosomal protein S28	**1.2**				

RPS3	ribosomal protein S3	**1.2**				
RPS6	ribosomal protein S6	**-1.7**	**-1.9**			**1.3**
RPS6KA1	ribosomal protein S6 kinase, 90kDa, polypeptide 1	**-2.9**	**-1.3**			**3.6**
RPS6KB1	ribosomal protein S6 kinase, 70kDa, polypeptide 1	**1.7**				**-1.7**
RPS8	ribosomal protein S8	**1.3**				
RQCD1	RCD1 required for cell differentiation1 homolog (S. pombe)	**-2.3**	**-1.7**			**-1.4**
RRAGA	Ras-related GTP binding A	**-2.6**	**1.3**	**1.4**		**3.2**
RRAGD	Ras-related GTP binding D	**3.0**	**-2.4**			**-8.9**
RRAS	related RAS viral (r-ras) oncogene homolog	**1.7**	**2.6**			**2.4**
RRM1	ribonucleotide reductase M1 polypeptide	**-1.6**	**-1.4**			**-2.1**
RRM2	ribonucleotide reductase M2 polypeptide	**-2.3**				**-1.8**
RRS1	RRS1 ribosome biogenesis regulator homolog (S. cerevisiae)	**-1.4**				**-1.6**
RSL1D1	ribosomal L1 domain containing 1	**-3.8**	**-2.1**			
RTN2	reticulon 2	**1.6**				
RUNX1	--	**-1.5**	**-2.5**	**-1.3**		**-1.4**
RUVBL1	RuvB-like 1 (E. coli)	**-1.6**				**-1.7**
RUVBL2	RuvB-like 2 (E. coli)	**1.2**	**-1.4**			**-2.0**
RW1	transmembrane protein 131	**1.4**	**1.3**			
RXRA	retinoid X receptor, alpha	**2.4**				**-3.3**
RYBP	RING1 and YY1 binding protein	**1.4**	**1.3**			**2.9**
RYK	RYK receptor-like tyrosine kinase	**11.1**				**-22.2**
S100A10	S100 calcium binding protein A10	**-26.4**				**40.6**
S100A11	S100 calcium binding protein A11	**1.5**			**-1.8**	
S100A4	S100 calcium binding protein A4	**-7.7**	**-4.0**			**5.4**
SACM1L	SAC1 suppressor of actin mutations 1-like (yeast)	**1.8**				**-1.6**
SACS	spastic ataxia of Charlevoix-Saguenay (sacsin)	**-1.7**	**-1.4**		**-2.0**	**-1.4**
SAP18	Sin3A-associated protein, 18kDa	**-2.2**				**1.7**
SAP30	Sin3A-associated protein, 30kDa	**1.7**	**2.3**	**2.2**		**3.8**
SARA1	SAR1 gene homolog A (S. cerevisiae)	**1.6**				**-1.7**
SARS	seryl-tRNA synthetase	**-2.7**	**-1.4**	**-1.4**		**1.8**
SAT	spermidine/spermine N1-acetyltransferase 1	**5.5**	**3.9**			**1.3**
SATB1	special AT-rich sequence binding protein 1 (binds to nuclear matrix/scaffold-associating DNA's)	**6.4**	**-1.8**	**-1.9**	**-2.3**	**-3.7**
SBF1	SET binding factor 1	**-1.9**	**3.1**			**6.9**
SC4MOL	sterol-C4-methyl oxidase-like	**-3.2**	**1.6**		**-1.6**	**4.6**
SC5DL	sterol-C5-desaturase (ERG3 delta-5-desaturase homolog, fungal)-like	**-1.7**				**2.6**
SCAMP1	secretory carrier membrane protein 1	**1.7**				**-1.7**
SCAMP5	secretory carrier membrane protein 5	**4.4**				**-2.6**
SCAP	SREBF chaperone	**1.2**				**-1.4**
SCARB1	scavenger receptor class B, member 1	**-1.4**	**-4.9**	**-1.7**		**-33.3**
SCC-112	SCC-112 protein	**-1.4**	**1.3**			**1.5**
SCHIP1	schwannomin interacting protein 1	**-5.1**	**1.4**			**1.9**
SCP2	sterol carrier protein 2	**-12.0**	**1.6**			**175.1**

SCRIB	scribbled homolog (Drosophila)	**1.5**			**1.7**	
SCYE1	small inducible cytokine subfamily E, member 1 (endothelial monocyte-activating)	**1.6**	**-1.4**	**-1.7**	**-1.9**	**-2.0**
SDCBP	syndecan binding protein (syntenin)	**-1.5**	**1.5**	**1.8**		**2.5**
SDFR1	neuroplastin	**1.5**				**-1.5**
SDHA	succinate dehydrogenase complex, subunit A, flavoprotein (Fp)	**-1.5**	**2.0**			**1.9**
SDHC	succinate dehydrogenase complex, subunit C, integral membrane protein, 15kDa	**-1.3**				**1.2**
SEC13L1	SEC13 homolog (S. cerevisiae)	**-1.2**				
SEC22L1	SEC22 vesicle trafficking protein homolog B (S. cerevisiae)	**-1.9**				**4.1**
SEC23IP	SEC23 interacting protein	**1.4**				**-1.6**
SEC24C	SEC24 related gene family, member C (S. cerevisiae)	**1.4**				**-1.3**
SEC24D	SEC24 related gene family, member D (S. cerevisiae)	**1.5**				
SEC61G	Sec61 gamma subunit	**2.2**				**-2.6**
SEC63	SEC63 homolog (S. cerevisiae)	**1.5**	**-1.3**		**-1.2**	**-2.5**
SEMA4D	sema domain, immunoglobulin domain (Ig), transmembrane domain (TM) and short cytoplasmic domain, (semaphorin) 4D	**-1.5**			**1.6**	**2.1**
SENP3	SUMO1/sentrin/SMT3 specific peptidase 3	**-2.9**	**-1.7**	**-1.3**	**-1.2**	
SENP6	SUMO1/sentrin specific peptidase 6	**2.0**	**1.5**			**1.2**
SEPHS1	selenophosphate synthetase 1	**-1.6**	**-2.1**			**-1.7**
SEPT2	septin 2	**-3.6**	**-1.5**			**2.4**
SEPT6	septin 6	**-1.4**	**-2.0**		**-1.6**	**-2.7**
SEPT7	septin 7	**2.6**	**1.3**			**-2.3**
SEPT8	septin 8	**1.5**	**-1.4**			**4.5**
SEPT9	septin 9	**-2.8**				**2.6**
SEPW1	selenoprotein W, 1	**2.4**	**1.3**			**-1.5**
SERP1	stress-associated endoplasmic reticulum protein 1	**1.5**	**-1.7**			**-1.6**
SERPINB1	serpin peptidase inhibitor, clade B (ovalbumin), member 1	**40.4**	**3.1**			**-7.4**
SERPINH1	serpin peptidase inhibitor, clade H (heat shock protein 47), member 1, (collagen binding protein 1)	**18.1**				**-17.3**
SERTAD2	SERTA domain containing 2	**1.5**				**-1.8**
SET	SET translocation (myeloid leukemia-associated)	**-1.3**	**-1.9**			**-1.5**
SF1	splicing factor 1	**-1.6**	**-1.4**			
SF3A1	splicing factor 3a, subunit 1, 120kDa	**-1.4**				
SF3A2	splicing factor 3a, subunit 2, 66kDa	**-1.5**	**-1.6**			
SF3B1	splicing factor 3b, subunit 1, 155kDa	**1.8**		**1.3**		
SF3B3	splicing factor 3b, subunit 3, 130kDa	**-1.4**	**-1.7**			**-2.7**
SFN	stratifin	**-1.7**	**-1.8**			
SFPQ	splicing factor proline/glutamine-rich (polypyrimidine tract binding protein associated)	**1.4**	**1.2**			
SFRS1	splicing factor, arginine/serine-rich 1 (splicing factor 2, alternate splicing factor)	**-1.6**	**-1.7**			**-2.4**
SFRS10	splicing factor, arginine/serine-rich 10 (transformer 2 homolog, Drosophila)	**-1.7**	**-1.2**			**-1.3**
SFRS11	splicing factor, arginine/serine-rich 11	**2.7**	**4.2**			**-4.1**
SFRS12	splicing factor, arginine/serine-rich 12	**4.5**	**-1.7**			**-3.5**
SFRS2	splicing factor, arginine/serine-rich 2	**-1.3**	**-1.4**			**-1.5**
SFRS2B	splicing factor, arginine/serine-rich 2B	**-1.3**	**-1.3**			**1.3**
SFRS3	splicing factor, arginine/serine-rich 3	**-1.4**	**1.3**			**1.5**

SFRS4	splicing factor, arginine/serine-rich 4	-1.2				
SFRS6	splicing factor, arginine/serine-rich 6	-3.6				3.4
SFRS7	splicing factor, arginine/serine-rich 7, 35kDa	-1.8	-1.5			-1.9
SFRS9	splicing factor, arginine/serine-rich 9	-2.7	1.2			2.0
SH3GLB1	SH3-domain GRB2-like endophilin B1	1.5	2.2			2.5
SHC1	SHC (Src homology 2 domain containing) transforming protein 1	-1.7				
SHFM1	split hand/foot malformation (ectrodactyly) type 1	-1.8				
SHMT1	serine hydroxymethyltransferase 1 (soluble)	-1.5	-1.8			1.4
SHMT2	serine hydroxymethyltransferase 2 (mitochondrial)	-2.3	-1.8			
SIAH1	seven in absentia homolog 1 (Drosophila)	-1.5				1.5
SIM2	single-minded homolog 2 (Drosophila)	2.4				-3.1
SIT	signaling threshold regulating transmembrane adaptor 1	-18.5				
SIVA	SIVA1, apoptosis-inducing factor	-1.6	-1.7			-2.9
SKI	v-ski sarcoma viral oncogene homolog (avian)	7.9				-5.0
SKIP (C62)	skeletal muscle and kidney enriched inositol phosphatase	-1.7				2.3
SLA	Src-like-adaptor	2.1	4.4	2.8	3.1	10.8
SLBP	stem-loop (histone) binding protein	-1.8	-1.3			-1.8
SLC11A2	solute carrier family 11 (proton-coupled divalent metal ion transporters), member 2	2.1	-2.1			-2.1
SLC12A2	solute carrier family 12 (sodium/potassium/chloride transporters), member 2	-1.9				1.9
SLC16A1	solute carrier family 16, member 1 (monocarboxylic acid transporter 1)	-1.2	-2.8			-3.9
SLC16A3	solute carrier family 16, member 3 (monocarboxylic acid transporter 4)	9.6				-28.1
SLC16A5	solute carrier family 16, member 5 (monocarboxylic acid transporter 6)	2.4				
SLC18A2	solute carrier family 18 (vesicular monoamine), member 2	4.3	1.6	4.1	4.2	-6.1
SLC1A4	solute carrier family 1 (glutamate/neutral amino acid transporter), member 4	-11.7	-1.8	1.6		2.2
SLC1A5	solute carrier family 1 (neutral amino acid transporter), member 5	-2.0	-2.3	-1.7		
SLC20A1	solute carrier family 20 (phosphate transporter), member 1	-2.2	-1.4			-2.7
SLC23A2	solute carrier family 23 (nucleobase transporters), member 2	1.3	-1.8			-2.5
SLC25A11	solute carrier family 25 (mitochondrial carrier; oxoglutarate carrier), member 11	-2.4				1.7
SLC25A3	solute carrier family 25 (mitochondrial carrier; phosphate carrier), member 3	1.2				
SLC26A2	solute carrier family 26 (sulfate transporter), member 2	2.8	1.3			
SLC2A3	solute carrier family 2 (facilitated glucose transporter), member 3	-1.9	6.0			-1.7
SLC30A1	solute carrier family 30 (zinc transporter), member 1	2.2				-1.8
SLC30A9	solute carrier family 30 (zinc transporter), member 9	2.1				-1.5
SLC39A14	solute carrier family 39 (zinc transporter), member 14	1.9	-3.8	-1.4	-1.3	-5.4
SLC39A6	solute carrier family 39 (zinc transporter), member 6	-1.7	-1.7		-1.5	
SLC3A2	solute carrier family 3 (activators of dibasic and neutral amino acid transport), member 2	-1.3				
SLC43A1	solute carrier family 43, member 1	1.8	-2.0			-2.5
SLC6A6	solute carrier family 6 (neurotransmitter transporter, taurine), member 6	8.8				-18.0
SLC7A1	solute carrier family 7 (cationic amino acid transporter, y+ system), member 1	-2.4	-2.7		-2.1	-2.3
SLC7A5	solute carrier family 7 (cationic amino acid transporter, y+ system), member 5	-3.9				1.5
SLC9A3R1	solute carrier family 9 (sodium/hydrogen exchanger), member 3 regulator 1	1.6	-1.4		-1.9	-2.3
SLK	STE20-like kinase (yeast)	4.3				-2.9

SMAD1	SMAD family member 1	**1.2**	**2.7**			**1.3**
SMAD2	SMAD family member 2	**-1.7**	**-1.3**			**1.9**
SMAD4	SMAD family member 4	**-1.5**	**1.6**		**-1.4**	**2.2**
SMAD5	SMAD family member 5	**-1.5**				
SMAD6	SMAD family member 6	**5.6**				**-6.8**
SMARCA2	SWI/SNF related, matrix associated, actin dependent regulator of chromatin, subfamily a, member 2	**-1.2**	**2.2**	**1.7**	**1.4**	**7.2**
SMARCA3	helicase-like transcription factor	**-1.4**	**-1.4**			**-1.4**
SMARCA4	SWI/SNF related, matrix associated, actin dependent regulator of chromatin, subfamily a, member 4	**-1.9**	**-1.6**			
SMARCB1	SWI/SNF related, matrix associated, actin dependent regulator of chromatin, subfamily b, member 1	**-1.3**				
SMARCC1	SWI/SNF related, matrix associated, actin dependent regulator of chromatin, subfamily c, member 1	**-1.4**	**-2.2**			**-2.4**
SMARCC2	SWI/SNF related, matrix associated, actin dependent regulator of chromatin, subfamily c, member 2	**-1.5**				**1.2**
SMARCD1	SWI/SNF related, matrix associated, actin dependent regulator of chromatin, subfamily d, member 1	**-1.9**				**1.7**
SMARCD2	SWI/SNF related, matrix associated, actin dependent regulator of chromatin, subfamily d, member 2	**1.3**	**-1.2**			
SMARCE1	SWI/SNF related, matrix associated, actin dependent regulator of chromatin, subfamily e, member 1	**-2.2**				**2.0**
SMC1L1	structural maintenance of chromosomes 1A	**-2.2**	**-1.2**			
SMC2L1	structural maintenance of chromosomes 2	**-2.3**	**1.3**			**-1.3**
SMC4L1	structural maintenance of chromosomes 4	**-1.5**	**1.4**	**1.7**	**1.7**	**6.8**
SMG1	PI-3-kinase-related kinase SMG-1	**-1.8**				**1.8**
SMNDC1	survival motor neuron domain containing 1	**1.6**				**-1.5**
SMPD4	sphingomyelin phosphodiesterase 4, neutral membrane (neutral sphingomyelinase-3)	**-1.4**				
SMURF2	SMAD specific E3 ubiquitin protein ligase 2	**1.6**	**1.6**			**1.5**
SNAPC3	small nuclear RNA activating complex, polypeptide 3, 50kDa	**-3.2**	**1.3**			**3.3**
SND1	staphylococcal nuclease and tudor domain containing 1	**-1.3**				
SNRK	SNF related kinase	**1.2**				
SNRPA1	small nuclear ribonucleoprotein polypeptide A'	**-1.4**	**-1.4**			**-2.4**
SNRPB	small nuclear ribonucleoprotein polypeptides B and B1	**-1.6**	**-1.7**			**-2.4**
SNRPC	small nuclear ribonucleoprotein polypeptide C	**-1.3**	**-1.5**			**-1.3**
SNRPD1	small nuclear ribonucleoprotein D1 polypeptide 16kDa	**-1.9**	**-1.7**	**-1.4**		**-2.0**
SNRPD2	small nuclear ribonucleoprotein D2 polypeptide 16.5kDa	**-1.2**	**-1.2**			
SNRPD3	small nuclear ribonucleoprotein D3 polypeptide 18kDa	**-1.6**				**-1.4**
SNRPE	small nuclear ribonucleoprotein polypeptide E	**-1.3**	**-1.3**			
SNTB2	syntrophin, beta 2 (dystrophin-associated protein A1, 59kDa, basic component 2)	**-5.9**	**3.5**	**3.0**	**2.3**	**8.7**
SNX1	sorting nexin 1	**3.6**				**-2.4**
SNX2	sorting nexin 2	**-1.6**	**-1.4**			**1.4**
SNX3	sorting nexin 3	**1.3**	**-1.5**			
SNX4	sorting nexin 4	**-1.2**			**-1.5**	**1.3**
SOCS1	suppressor of cytokine signaling 1	**-1.6**	**8.3**	**15.6**	**29.3**	**40.4**
SOCS2	suppressor of cytokine signaling 2	**-1.8**	**2.8**		**2.8**	**4.7**
SOCS5	suppressor of cytokine signaling 5	**1.7**	**1.3**			
SOCS6	suppressor of cytokine signaling 6	**1.3**	**1.9**			
SORD	sorbitol dehydrogenase	**-1.3**	**-1.6**			**-2.9**
SOX4	SRY (sex determining region Y)-box 4	**-5.7**	**-1.3**			**3.0**

SP100	SP100 nuclear antigen	**-1.7**	**2.3**			**6.4**
SP3	Sp3 transcription factor	**-2.8**				**2.2**
SPA17	sperm autoantigenic protein 17	**-2.0**				**1.8**
SPAG7	sperm associated antigen 7	**-1.6**				
SPAG9	sperm associated antigen 9	**2.0**	**1.3**			**1.3**
SPAST	spastin	**1.3**				**-1.2**
SPBC25	spindle pole body component 25 homolog (S. cerevisiae)	**1.4**				**-3.1**
SPCS2	signal peptidase complex subunit 2 homolog (S. cerevisiae)	**1.3**	**-1.3**		**-1.4**	**-1.6**
SPHAR	S-phase response (cyclin-related)	**3.4**				**-3.2**
SPHK2	sphingosine kinase 2	**-1.7**				**1.6**
SPINT2	serine peptidase inhibitor, Kunitz type, 2	**4.2**		**1.4**		**-2.6**
SPN	sialophorin (leukosialin, CD43)	**-1.9**	**-1.2**			**1.7**
SPTBN1	spectrin, beta, non-erythrocytic 1	**-1.4**	**2.4**			
SPTLC1	serine palmitoyltransferase, long chain base subunit 1	**1.7**				**-1.2**
SQLE	squalene epoxidase	**-2.0**	**-1.4**			**1.8**
SRD5A1	steroid-5-alpha-reductase, alpha polypeptide 1 (3-oxo-5 alpha-steroid delta 4-dehydrogenase alpha 1)	**2.0**	**1.2**		**3.0**	**4.9**
SRF	serum response factor (c-fos serum response element-binding transcription factor)	**-1.3**		**-1.5**		
SRI	sorcin	**1.5**			**-1.6**	**-1.9**
SRP14	signal recognition particle 14kDa (homologous Alu RNA binding protein)	**-1.4**				**1.5**
SRP72	signal recognition particle 72kDa	**3.8**	**-1.5**			**-5.1**
SRPK2	SFRS protein kinase 2	**-1.3**	**1.2**			**1.8**
SRR	serine racemase	**-2.1**	**-2.2**			
SRRM2	serine/arginine repetitive matrix 2	**-1.9**	**-1.4**			**1.4**
SS18	synovial sarcoma translocation, chromosome 18	**1.5**				**-1.5**
SS18L1	synovial sarcoma translocation gene on chromosome 18-like 1	**1.6**	**-1.5**			**-2.4**
SSB	Sjogren syndrome antigen B (autoantigen La)	**1.7**	**-1.7**		**-1.6**	**-3.1**
SSBP1	single-stranded DNA binding protein 1	**1.3**	**-1.8**			**-1.4**
SSBP2	single-stranded DNA binding protein 2	**-7.6**				**7.2**
SSR1	signal sequence receptor, alpha (translocon-associated protein alpha)	**1.2**				**-1.4**
SSR4	signal sequence receptor, delta (translocon-associated protein delta)	**1.7**	**-1.3**			**-2.1**
SSRP1	structure specific recognition protein 1	**-1.8**	**-2.2**			**-1.9**
ST13	suppression of tumorigenicity 13 (colon carcinoma) (Hsp70 interacting protein)	**1.5**	**-1.5**			**-2.2**
ST3GAL5	ST3 beta-galactoside alpha-2,3-sialyltransferase 5	**4.7**		**1.6**		**-10.8**
ST3GAL6	ST3 beta-galactoside alpha-2,3-sialyltransferase 6	**13.7**	**1.9**	**2.1**		**-28.6**
ST6GAL1	ST6 beta-galactosamide alpha-2,6-sialyltranferase 1	**-1.7**				**1.5**
ST6GALNAC4	ST6 (alpha-N-acetyl-neuraminyl-2,3-beta-galactosyl-1,3)-N-acetylgalactosaminide alpha-2,6-sialyltransferase 4	**-1.7**			**1.4**	**1.7**
STAG1	stromal antigen 1	**-1.4**				**1.2**
STAG2	stromal antigen 2	**-1.6**				**1.7**
STAM	signal transducing adaptor molecule (SH3 domain and ITAM motif) 1	**1.6**		**-1.5**		**-1.9**
STAMBP	STAM binding protein	**-1.2**				**1.6**
STAT1	signal transducer and activator of transcription 1, 91kDa	**1.7**	**-1.5**			**-4.2**
STAT3	signal transducer and activator of transcription 3 (acute-phase response factor)	**3.8**		**1.3**		**-1.4**

STAT5A	signal transducer and activator of transcription 5A	**2.7**		**-1.3**	**-1.7**	**-3.2**
STAU	staufen, RNA binding protein, homolog 1 (Drosophila)	**-1.2**				**1.2**
STAU2	staufen, RNA binding protein, homolog 2 (Drosophila)	**-2.9**	**1.6**			**2.5**
STCH	stress 70 protein chaperone, microsome-associated, 60kDa	**1.6**				**-2.5**
STIM1	stromal interaction molecule 1	**-1.4**	**1.9**	**1.9**	**2.0**	**3.4**
STIP1	stress-induced-phosphoprotein 1 (Hsp70/Hsp90-organizing protein)	**-1.6**	**-1.4**	**-1.8**		**-1.3**
STK10	serine/threonine kinase 10	**-1.4**				**1.7**
STK24	serine/threonine kinase 24 (STE20 homolog, yeast)	**-2.2**	**1.3**			**1.8**
STK3	serine/threonine kinase 3 (STE20 homolog, yeast)	**-2.2**	**1.5**			**3.6**
STK38	serine/threonine kinase 38	**-2.3**	**1.7**			**2.1**
STK39	serine threonine kinase 39 (STE20/SPS1 homolog, yeast)	**-2.5**	**1.8**			**3.1**
STRAP	serine/threonine kinase receptor associated protein	**-1.4**				**2.1**
STS	steroid sulfatase (microsomal), arylsulfatase C, isozyme S	**-3.6**		**-1.6**		**2.4**
STX16	syntaxin 16	**1.5**				**1.5**
STX3A	syntaxin 3	**1.7**				
STX6	syntaxin 6	**-1.9**				**1.7**
STX7	syntaxin 7	**1.5**	**1.3**			**1.4**
STX8	syntaxin 8	**-2.6**				**3.2**
STXBP1	syntaxin binding protein 1	**-13.5**		**2.0**	**3.8**	**11.7**
STXBP3	syntaxin binding protein 3	**-1.2**	**1.7**			**1.9**
SULT1A1	sulfotransferase family, cytosolic, 1A, phenol-preferring, member 1	**-1.5**				**2.2**
SUMO1	SMT3 suppressor of mif two 3 homolog 1 (S. cerevisiae)	**1.2**	**-1.3**			**-2.1**
SUMO2	SMT3 suppressor of mif two 3 homolog 2 (S. cerevisiae)	**3.0**				**-3.1**
SUPT3H	suppressor of Ty 3 homolog (S. cerevisiae)	**-6.4**				**10.6**
SUPT4H1	suppressor of Ty 4 homolog 1 (S. cerevisiae)	**-1.7**	**1.6**			**2.0**
SUPT6H	suppressor of Ty 6 homolog (S. cerevisiae)	**1.8**				**-1.5**
SURF5	surfeit 5	**-1.4**				
SV2A	synaptic vesicle glycoprotein 2A	**10.1**	**1.9**	**1.7**	**1.4**	**-14.8**
SWAP70	SWAP-70 protein	**-1.3**	**1.4**	**1.3**		**1.6**
SYBL1	synaptobrevin-like 1	**1.3**	**1.2**			
SYNCRIP	synaptotagmin binding, cytoplasmic RNA interacting protein	**-1.7**	**-1.6**		**-1.4**	**-1.9**
SYNE2	spectrin repeat containing, nuclear envelope 2	**-10.7**	**6.9**	**1.5**		**38.9**
SYNGR2	synaptogyrin 2	**1.9**				**-2.4**
SYNJ2	synaptojanin 2	**-13.7**		**1.9**		**48.6**
TACC1	transforming, acidic coiled-coil containing protein 1	**2.5**				**1.7**
TAF1	TAF1 RNA polymerase II, TATA box binding protein (TBP)-associated factor, 250kDa	**1.9**				**-1.4**
TAF10	TAF10 RNA polymerase II, TATA box binding protein (TBP)-associated factor, 30kDa	**-1.4**				**1.4**
TAF15	TAF15 RNA polymerase II, TATA box binding protein (TBP)-associated factor, 68kDa	**1.6**	**-1.4**			**-1.7**
TAF1A	TATA box binding protein (TBP)-associated factor, RNA polymerase I, A, 48kDa	**-2.1**	**-1.8**			
TAF1C	TATA box binding protein (TBP)-associated factor, RNA polymerase I, C, 110kDa	**1.8**				**-1.6**
TAF2	TAF2 RNA polymerase II, TATA box binding protein (TBP)-associated factor, 150kDa	**-1.3**				**1.6**
TAF4B	TAF4b RNA polymerase II, TATA box binding protein (TBP)-associated factor, 105kDa	**-1.3**				**-2.3**

TAF9	TAF9 RNA polymerase II, TATA box binding protein (TBP)-associated factor, 32kDa	**2.2**				**-3.2**
TAGLN2	transgelin 2	**-3.0**		**-1.4**		
TAL1	T-cell acute lymphocytic leukemia 1	**1.7**		**1.6**		**-2.3**
TALDO1	transaldolase 1	**1.8**				**-1.7**
TANK	TRAF family member-associated NFKB activator	**2.1**	**1.4**			**-1.3**
TAOK3	TAO kinase 3	**2.3**	**-1.4**			**-2.9**
TAPBP	TAP binding protein (tapasin)	**-1.8**	**-1.3**			**1.2**
TARDBP	TAR DNA binding protein	**1.9**	**-1.2**			**-1.3**
TAX1BP1	Tax1 (human T-cell leukemia virus type I) binding protein 1	**4.5**	**1.4**	**1.5**		**-2.6**
TAX1BP3	Tax1 (human T-cell leukemia virus type I) binding protein 3	**-2.1**	**3.3**			**4.2**
TBC1D22A	TBC1 domain family, member 22A	**-1.5**				**-1.2**
TBPL1	TBP-like 1	**-1.6**				**1.5**
TCEA1	transcription elongation factor A (SII), 1	**-1.7**				**1.5**
TCEAL1	transcription elongation factor A (SII)-like 1	**1.8**				**-1.4**
TCEB1	transcription elongation factor B (SIII), polypeptide 1 (15kDa, elongin C)	**-1.6**	**1.4**			
TCEB2	transcription elongation factor B (SIII), polypeptide 2 (18kDa, elongin B)	**-2.9**				**1.4**
TCERG1	transcription elongation regulator 1	**-1.4**	**-1.4**		**-1.4**	**-1.4**
TCF12	transcription factor 12 (HTF4, helix-loop-helix transcription factors 4)	**-2.5**	**-2.0**			**2.8**
TCF3	transcription factor 3 (E2A immunoglobulin enhancer binding factors E12/E47)	**-4.7**				**4.7**
TCFL5	transcription factor-like 5 (basic helix-loop-helix)	**-2.5**	**2.1**			**2.8**
TCP1	t-complex 1	**-1.3**	**-1.5**		**-1.7**	**-1.6**
TCTEL1	dynein, light chain, Tctex-type 1	**1.2**	**1.5**			
TDRD7	tudor domain containing 7	**-2.6**				**5.6**
TEGT	testis enhanced gene transcript (BAX inhibitor 1)	**1.2**	**1.4**			**1.3**
TERF1	telomeric repeat binding factor (NIMA-interacting) 1	**-1.6**				**2.0**
TERF2	telomeric repeat binding factor 2	**-1.5**				**1.4**
TES	testis derived transcript (3 LIM domains)	**1.4**				
TFAM	transcription factor A, mitochondrial	**-1.4**	**-1.8**		**-1.8**	**-2.0**
TFCP2	transcription factor CP2	**-1.5**	**-1.9**			**1.8**
TFDP1	transcription factor Dp-1	**-1.8**	**-1.3**			**-1.6**
TFDP2	transcription factor Dp-2 (E2F dimerization partner 2)	**3.1**	**-1.4**	**-1.5**		
TFPI	tissue factor pathway inhibitor (lipoprotein-associated coagulation inhibitor)	**4.9**	**9.6**	**3.1**	**1.9**	**24.6**
TGFB1	transforming growth factor, beta 1 (Camurati-Engelmann disease)	**32.0**				**-3.3**
TGFBR2	transforming growth factor, beta receptor II (70/80kDa)	**-3.3**	**1.8**	**2.8**	**4.0**	**6.9**
TGIF2	TGFB-induced factor 2 (TALE family homeobox)	**1.9**	**-1.6**			**-2.9**
TGOLN2	trans-golgi network protein 2	**1.3**	**1.2**			**-1.6**
THOC2	THO complex 2	**1.5**	**1.6**			**-1.5**
THRAP1	thyroid hormone receptor associated protein 1	**1.9**	**1.5**			**-1.3**
THRAP2	thyroid hormone receptor associated protein 2	**-1.5**	**4.2**			**2.3**
TIA1	TIA1 cytotoxic granule-associated RNA binding protein	**-1.7**	**-1.3**			**1.8**
TIAL1	TIA1 cytotoxic granule-associated RNA binding protein-like 1	**1.7**	**-1.4**			**-2.0**
TIF1	tripartite motif-containing 24	**1.6**				**-1.5**

TIMM17A	translocase of inner mitochondrial membrane 17 homolog A (yeast)	**-1.2**	**-1.5**		**-1.5**	**-1.6**
TIMP2	TIMP metallopeptidase inhibitor 2	**2.1**				**-3.0**
TIP120A	cullin-associated and neddylation-dissociated 1	**1.6**	**-1.3**			**-1.4**
TKT	transketolase (Wernicke-Korsakoff syndrome)	**1.5**	**-1.3**			**-1.8**
TLE1	transducin-like enhancer of split 1 (E(sp1) homolog, Drosophila)	**-2.0**	**5.6**			**7.4**
TLE4	transducin-like enhancer of split 4 (E(sp1) homolog, Drosophila)	**-1.4**	**-1.5**			**1.7**
TLK1	tousled-like kinase 1	**1.7**	**1.4**			
TLK2	tousled-like kinase 2	**1.7**				
TLN1	talin 1	**-1.6**		**1.4**		**2.6**
TM2D1	TM2 domain containing 1	**1.6**	**1.8**			**1.8**
TM4SF2	tetraspanin 7	**35.8**	**4.8**			**-11.4**
TMED7	transmembrane emp24 protein transport domain containing 7	**1.6**	**1.7**			
TMED9	transmembrane emp24 protein transport domain containing 9	**1.8**				**-2.4**
TMEM1	transmembrane protein 1	**-1.4**				**1.5**
TMEM11	transmembrane protein 11	**-1.7**				
TMEM4	transmembrane protein 4	**1.9**	**-1.5**			**-3.3**
TMEM87A	transmembrane protein 87A	**1.2**	**1.3**			
TMF1	TATA element modulatory factor 1	**2.1**	**1.7**	**-1.2**		**-2.2**
TMP21	transmembrane emp24-like trafficking protein 10 (yeast)	**1.5**				**2.0**
TMPO	thymopoietin	**-2.2**	**1.4**			**1.7**
TMSB10	thymosin, beta 10	**-1.9**	**1.6**	**1.4**	**2.2**	**3.2**
TMSL8	thymosin-like 8	**-5.4**				**2.0**
TNFAIP8	tumor necrosis factor, alpha-induced protein 8	**-1.5**	**-1.9**	**-1.3**		**-1.9**
TNFRSF10D	tumor necrosis factor receptor superfamily, member 10d, decoy with truncated death domain	**1.3**				**-2.0**
TNFRSF1A	tumor necrosis factor receptor superfamily, member 1A	**2.9**				**-5.7**
TNKS	tankyrase, TRF1-interacting ankyrin-related ADP-ribose polymerase	**-1.7**	**2.5**			**1.3**
TNPO1	transportin 1	**-1.4**	**1.4**			**-1.6**
TNPO3	transportin 3	**1.4**				
TOB1	transducer of ERBB2, 1	**1.9**	**-1.6**	**1.6**		
TOB2	transducer of ERBB2, 2	**1.9**	**1.4**			
TOMM20	translocase of outer mitochondrial membrane 20 homolog (yeast)	**2.0**		**-1.5**		**-2.1**
TOP2B	topoisomerase (DNA) II beta 180kDa	**-1.9**				**1.4**
TOPBP1	topoisomerase (DNA) II binding protein 1	**-1.2**				**-1.5**
TOX	thymus high mobility group box protein TOX	**26.1**	**-1.6**	**-1.3**		**-32.2**
TP53	tumor protein p53 (Li-Fraumeni syndrome)	**-2.6**	**-2.5**			
TP53BP2	tumor protein p53 binding protein, 2	**2.0**			**1.7**	
TPD52L2	tumor protein D52-like 2	**-1.6**				**1.4**
TPI1	triosephosphate isomerase 1	**-1.5**	**-1.5**			
TPMT	thiopurine S-methyltransferase	**1.8**				**-2.2**
TPP1	tripeptidyl peptidase I	**1.7**	**1.4**			**-1.3**
TPP2	tripeptidyl peptidase II	**-1.3**				**-1.3**
TPR	translocated promoter region (to activated MET oncogene)	**1.4**	**-1.3**			**-1.6**

TPST2	tyrosylprotein sulfotransferase 2	-1.7	2.0			2.9
TRA@	T cell receptor alpha locus	-38.6	-1.9		2.9	24.7
TRA1	heat shock protein 90kDa beta (Grp94), member 1	1.4				-2.2
TRA2A	transformer-2 alpha	2.6				-2.3
TRAF3	TNF receptor-associated factor 3	2.8				-7.4
TRAF3IP2	TRAF3 interacting protein 2	-5.3				3.8
TRAF4	TNF receptor-associated factor 4	-13.8	-1.7	-1.7	-1.5	
TRAM1	translocation associated membrane protein 1	3.3				-2.3
TRAM2	translocation associated membrane protein 2	-2.0	2.4	1.5	1.7	2.0
TRAPPC3	trafficking protein particle complex 3	1.4				-1.4
TRFP	Trf (TATA binding protein-related factor)-proximal homolog (Drosophila)	-1.7	-1.4			
TRIB2	tribbles homolog 2 (Drosophila)	6.1				-6.0
TRIM14	tripartite motif-containing 14	3.2	-2.4			-4.7
TRIM33	tripartite motif-containing 33	-1.6				-1.2
TRIM37	tripartite motif-containing 37	1.8	-1.3			-14.1
TRIP12	thyroid hormone receptor interactor 12	1.6	1.5			2.3
TRRAP	transformation/transcription domain-associated protein	-1.7				2.1
TSC2	tuberous sclerosis 2	-1.8	1.5			6.8
TSC22D1	TSC22 domain family, member 1	2.0	2.9			-2.4
TSC22D3	TSC22 domain family, member 3	5.2	17.5	33.1	20.4	20.0
TSFM	Ts translation elongation factor, mitochondrial	-1.2	-2.5	-1.5	-1.6	-1.6
TSNAX	translin-associated factor X	1.2	1.9	2.1	2.2	2.0
TSPAN5	tetraspanin 5	3.2		2.1		
TSR1	TSR1, 20S rRNA accumulation, homolog (S. cerevisiae)	-2.0	-2.4	-2.0	-2.0	-1.8
TTC3	tetratricopeptide repeat domain 3	2.3	-1.6			-5.1
TTF1	transcription termination factor, RNA polymerase I	-2.7	-1.3			1.8
TTF2	transcription termination factor, RNA polymerase II	-1.2				
TUBA1	tubulin, alpha 1	-4.4	2.1	10.4	3.0	13.5
TUBA3	tubulin, alpha 3	-10.6	1.7		-1.6	15.1
TUBB	tubulin, beta	-1.6				
TUBB2A	tubulin, beta 2A	-25.5	3.2			54.6
TUBB2C	tubulin, beta 2C	-1.3	1.4			
TUBB3	tubulin, beta 3	-1.8				
TUBGCP3	tubulin, gamma complex associated protein 3	-1.4				-1.7
TUFM	Tu translation elongation factor, mitochondrial	-1.5	-1.5		-1.3	
TXLNA	taxilin alpha	1.2				-2.0
TXN	thioredoxin	1.8	1.3		1.7	-1.5
TXN2	thioredoxin 2	-1.4				
TXNDC9	thioredoxin domain containing 9	1.5				-1.2
TXNIP	thioredoxin interacting protein	10.0	7.7	2.8	3.7	2.1
TXNL1	thioredoxin-like 1	1.3	-2.1			-1.4
TXNRD1	thioredoxin reductase 1	-1.3			-1.4	

TYMS	thymidylate synthetase	**2.1**	**-1.2**			**-4.8**
U2AF2	U2 small nuclear RNA auxiliary factor 2	**-1.3**				
UAP1	UDP-N-acteylglucosamine pyrophosphorylase 1	**1.8**	**-2.3**	**-1.3**	**-1.5**	**-3.9**
UBB	ubiquitin B	**-1.6**	**1.7**			**1.5**
UBE1	ubiquitin-activating enzyme E1 (A1S9T and BN75 temperature sensitivity complementing)	**-1.3**	**-1.4**			
UBE1L	ubiquitin-activating enzyme E1-like	**-1.5**	**-1.4**			**1.8**
UBE2C	ubiquitin-conjugating enzyme E2C	**1.3**	**1.7**			
UBE2D1	ubiquitin-conjugating enzyme E2D 1 (UBC4/5 homolog, yeast)	**-1.3**				**1.3**
UBE2D2	ubiquitin-conjugating enzyme E2D 2 (UBC4/5 homolog, yeast)	**-1.9**	**1.6**			**4.1**
UBE2D3	ubiquitin-conjugating enzyme E2D 3 (UBC4/5 homolog, yeast)	**-1.3**	**1.3**		**-1.3**	**1.6**
UBE2E1	ubiquitin-conjugating enzyme E2E 1 (UBC4/5 homolog, yeast)	**1.4**				**-1.8**
UBE2E3	ubiquitin-conjugating enzyme E2E 3 (UBC4/5 homolog, yeast)	**2.2**				**-2.1**
UBE2G1	ubiquitin-conjugating enzyme E2G 1 (UBC7 homolog, yeast)	**-1.6**				
UBE2I	ubiquitin-conjugating enzyme E2I (UBC9 homolog, yeast)	**-4.5**	**-1.2**			**2.8**
UBE2J1	ubiquitin-conjugating enzyme E2, J1 (UBC6 homolog, yeast)	**1.5**	**1.5**			**-1.5**
UBE2L3	ubiquitin-conjugating enzyme E2L 3	**1.5**	**-2.1**			**-2.1**
UBE2L6	ubiquitin-conjugating enzyme E2L 6	**-2.0**	**-1.7**	**-1.2**	**-1.3**	
UBE2N	ubiquitin-conjugating enzyme E2N (UBC13 homolog, yeast)	**-1.4**	**-1.3**			
UBE2V2	ubiquitin-conjugating enzyme E2 variant 2	**-1.4**	**-1.3**			**-1.7**
UBE3A	ubiquitin protein ligase E3A (human papilloma virus E6-associated protein, Angelman syndrome)	**-5.8**	**1.4**	**-1.4**		**8.6**
UBE3C	ubiquitin protein ligase E3C	**-1.3**				
UBE4B	ubiquitination factor E4B (UFD2 homolog, yeast)	**1.3**				**-1.3**
UBN1	ubinuclein 1	**-1.8**	**-1.2**			**1.3**
UBTF	upstream binding transcription factor, RNA polymerase I	**-1.2**	**-1.3**	**-1.3**		**-1.4**
UCK2	uridine-cytidine kinase 2	**-1.7**	**-1.4**			**-3.0**
UCP2	uncoupling protein 2 (mitochondrial, proton carrier)	**1.3**				
UGCG	UDP-glucose ceramide glucosyltransferase	**-2.4**	**1.2**			**3.0**
UMPS	uridine monophosphate synthetase (orotate phosphoribosyl transferase and orotidine-5'-decarboxylase)	**-1.3**		**-1.3**		**-1.6**
UPF2	UPF2 regulator of nonsense transcripts homolog (yeast)	**2.2**				**-2.0**
UPF3A	UPF3 regulator of nonsense transcripts homolog A (yeast)	**-1.4**				**1.4**
UQCR	ubiquinol-cytochrome c reductase, 6.4kDa subunit	**-1.3**				
UQCRC2	ubiquinol-cytochrome c reductase core protein II	**-1.6**				**1.8**
UQCRFS1	ubiquinol-cytochrome c reductase, Rieske iron-sulfur polypeptide 1	**1.2**				**-1.6**
UQCRH	ubiquinol-cytochrome c reductase hinge protein	**1.5**				**-1.9**
UROD	uroporphyrinogen decarboxylase	**2.2**				**-2.4**
UROS	uroporphyrinogen III synthase (congenital erythropoietic porphyria)	**2.4**	**1.6**			**-6.2**
USF2	upstream transcription factor 2, c-fos interacting	**-1.5**				**1.6**
USP1	ubiquitin specific peptidase 1	**-1.5**			**-1.4**	
USP11	ubiquitin specific peptidase 11	**-1.6**	**1.3**			**1.4**
USP12	ubiquitin specific peptidase 12	**-2.0**	**2.3**			**3.7**
USP14	ubiquitin specific peptidase 14 (tRNA-guanine transglycosylase)	**1.3**	**-1.3**			**-1.7**
USP22	ubiquitin specific peptidase 22	**-2.6**				**2.7**

USP24	ubiquitin specific peptidase 24	1.4	1.3			-1.3
USP32	ubiquitin specific peptidase 32	3.1				-2.2
USP33	ubiquitin specific peptidase 33	1.5	1.3			
USP34	ubiquitin specific peptidase 34	1.3	1.8			2.6
USP4	ubiquitin specific peptidase 4 (proto-oncogene)	-1.7	1.3			2.4
USP46	ubiquitin specific peptidase 46	3.5				-3.0
USP6	ubiquitin specific peptidase 6 (Tre-2 oncogene)	1.4	-1.3			
USP7	ubiquitin specific peptidase 7 (herpes virus-associated)	-3.0				2.6
UTX	ubiquitously transcribed tetratricopeptide repeat, X chromosome	-1.6	1.4			2.9
UVRAG	UV radiation resistance associated gene	1.6			1.5	
VAMP2	vesicle-associated membrane protein 2 (synaptobrevin 2)	-4.2	-1.3			4.9
VAMP3	vesicle-associated membrane protein 3 (cellubrevin)	1.3	1.3			1.5
VAMP4	vesicle-associated membrane protein 4	-2.6				2.7
VAMP8	vesicle-associated membrane protein 8 (endobrevin)	2.0				-4.1
VAPA	VAMP (vesicle-associated membrane protein)-associated protein A, 33kDa	1.3				-1.7
VCL	vinculin	1.9	-1.5		1.9	-1.4
VCP	valosin-containing protein	1.3	-1.3			-1.4
VDAC1	voltage-dependent anion channel 1	1.6		-1.6	-1.7	-3.1
VDAC2	voltage-dependent anion channel 2	1.9			-1.5	-2.1
VDAC3	voltage-dependent anion channel 3	1.8				-1.9
VDP	vesicle docking protein p115	2.0				-1.6
VEGF	vascular endothelial growth factor A	8.2	-2.4	-1.5		-13.8
VEGFB	vascular endothelial growth factor B	-2.5				
VGLL4	vestigial like 4 (Drosophila)	1.3				
VIL2	villin 2 (ezrin)	-2.2	2.4			9.8
VIM	vimentin	-275.9	1.2			360.4
VLDLR	very low density lipoprotein receptor	-4.7	-1.2			2.6
VPS11	vacuolar protein sorting 11 homolog (S. cerevisiae)	1.6				
VPS13A	vacuolar protein sorting 13 homolog A (S. cerevisiae)	3.7			-1.3	-2.8
VPS26	vacuolar protein sorting 26 homolog A (yeast)	1.5	1.3			
VTI1B	vesicle transport through interaction with t-SNAREs homolog 1B (yeast)	1.7	1.3			-1.7
WARS	tryptophanyl-tRNA synthetase	-1.5	-3.3			-1.8
WBP4	WW domain binding protein 4 (formin binding protein 21)	-1.2				1.5
WBSCR1	eukaryotic translation initiation factor 4H	-2.4				1.5
WDR1	WD repeat domain 1	-2.5				2.8
WEE1	WEE1 homolog (S. pombe)	-1.4	1.2			
WIT-1	Wilms tumor upstream neighbor 1	-2.4				2.5
WSB1	WD repeat and SOCS box-containing 1	9.0	2.0			-2.0
WSB2	WD repeat and SOCS box-containing 2	1.4	1.3			-1.7
WT1	Wilms tumor 1	-1.6	1.4	-1.5	-1.8	-1.7
WTAP	Wilms tumor 1 associated protein	-1.5	1.7			1.8
WWOX	WW domain containing oxidoreductase	-1.4	-1.7			

XAB1	XPA binding protein 1, GTPase	**-1.6**				**1.6**
XIST	X (inactive)-specific transcript	**-808.5**				**1094.1**
XPNPEP1	X-prolyl aminopeptidase (aminopeptidase P) 1, soluble	**3.2**				**-3.9**
XPO1	exportin 1 (CRM1 homolog, yeast)	**-1.4**				**4.3**
XPO7	exportin 7	**4.8**	**1.3**			**-2.7**
XPOT	exportin, tRNA (nuclear export receptor for tRNAs)	**1.6**	**-3.2**			**-6.4**
XRCC3	X-ray repair complementing defective repair in Chinese hamster cells 3	**-1.7**				
XRCC4	X-ray repair complementing defective repair in Chinese hamster cells 4	**-1.8**	**1.4**			**1.6**
XRCC5	X-ray repair complementing defective repair in Chinese hamster cells 5 (double-strand-break rejoining; Ku autoantigen, 80kDa)	**-2.0**	**-1.7**			
YAF2	YY1 associated factor 2	**1.7**	**1.9**	**1.8**	**1.6**	**5.8**
YARS	tyrosyl-tRNA synthetase	**-1.9**	**-2.6**			**-2.4**
YES1	v-yes-1 Yamaguchi sarcoma viral oncogene homolog 1	**49.1**			**1.3**	**-63.8**
YME1L1	YME1-like 1 (S. cerevisiae)	**1.7**	**-1.2**			**-1.7**
YWHAE	tyrosine 3-monooxygenase/tryptophan 5-monooxygenase activation protein, epsilon polypeptide	**-2.5**	**-1.6**			**2.2**
YWHAQ	tyrosine 3-monooxygenase/tryptophan 5-monooxygenase activation protein, theta polypeptide	**-1.7**	**1.6**			**1.5**
YWHAZ	tyrosine 3-monooxygenase/tryptophan 5-monooxygenase activation protein, zeta polypeptide	**-2.5**	**1.7**			**3.9**
YY1	YY1 transcription factor	**2.7**				**-2.7**
ZAP70	zeta-chain (TCR) associated protein kinase 70kDa	**-10.1**			**-1.3**	**13.8**
ZBTB1	zinc finger and BTB domain containing 1	**-1.4**				**1.6**
ZC3HAV1	zinc finger CCCH-type, antiviral 1	**-1.7**	**1.4**			**2.6**
ZFP36L1	zinc finger protein 36, C3H type-like 1	**-24.5**				**11.5**
ZFP36L2	zinc finger protein 36, C3H type-like 2	**2.2**	**1.8**	**3.0**	**2.5**	
ZFX	zinc finger protein, X-linked	**-1.6**				**1.7**
ZHX2	zinc fingers and homeoboxes 2	**-5.1**	**1.3**			**6.9**
ZHX3	zinc fingers and homeoboxes 3	**2.0**	**2.0**			
ZMPSTE24	zinc metallopeptidase (STE24 homolog, yeast)	**1.4**				**-1.8**
ZMYND11	zinc finger, MYND domain containing 11	**2.6**	**1.3**			
ZNF143	zinc finger protein 143	**-1.4**				
ZNF148	zinc finger protein 148	**1.8**	**1.8**	**1.3**		**-1.6**
ZNF160	zinc finger protein 160	**-1.7**	**1.5**			**1.6**
ZNF161	vascular endothelial zinc finger 1	**-1.9**				**2.2**
ZNF175	zinc finger protein 175	**2.9**				**-2.6**
ZNF198	zinc finger, MYM-type 2	**4.3**	**-1.3**			**-2.5**
ZNF202	zinc finger protein 202	**-1.4**	**-1.8**			
ZNF207	zinc finger protein 207	**2.4**	**-1.4**			
ZNF22	zinc finger protein 22 (KOX 15)	**-1.3**				**1.3**
ZNF238	zinc finger protein 238	**-5.5**				**6.3**
ZNF259	zinc finger protein 259	**-1.2**		**-1.4**	**-1.8**	**-1.6**
ZNF263	zinc finger protein 263	**-1.7**	**-1.7**			**1.8**
ZNF267	zinc finger protein 267	**1.9**				**-1.9**
ZNF291	zinc finger protein 291	**1.4**	**1.6**			**2.1**
ZNF318	zinc finger protein 318	**2.3**	**1.3**			

ZNF354A	zinc finger protein 354A	**2.6**				
ZNF364	zinc finger protein 364	**1.3**	**1.8**			**3.0**
ZNF384	zinc finger protein 384	**-1.6**	**-1.6**			
ZNF423	zinc finger protein 423	**-3.6**	**-2.2**			**5.6**
ZNF451	zinc finger protein 451	**2.5**				**-1.6**
ZNF592	zinc finger protein 592	**-1.7**	**1.3**			**1.4**
ZNF91	zinc finger protein 91	**-2.2**				**2.2**
ZNFN1A1	IKAROS family zinc finger 1 (Ikaros)	**2.2**	**1.3**			**2.2**
ZRF1	zuotin related factor 1	**1.4**	**-1.5**			**-2.9**
ZUBR1	zinc finger, UBR1 type 1	**2.0**				**-1.9**
