# Supplementary material for: Gene expression profiling of leukemic cells and primary thymocytes predicts a signature for apoptotic sensitivity to glucocorticoids
Source: Cancer Cell Int. 2007 Nov 28;7:18. doi: 10.1186/1475-2867-7-18 (PMC2228275; doi:10.1186/1475-2867-7-18)
Supplement: Additional file 6 — Myeloid AML vs. Lymphoid ALL. Genes statistically significantly regulated by Dex in Kasumi-1 AML cells compared to GC-sensitive lymphoid leukemic cells. Blank = gene "absent" by selection criteria. [file 1475-2867-7-18-S6.pdf]

**Additional file 6: Myeloid AML vs. Lymphoid ALL.**						
**Blank = "absent"**						
**GC-response**		**Sensitive**	**Sensitive**	**Sensitive**	**Sensitive**	**Sensitive**
**Patient-derived cell line**		**Pediatric**	**Adult**	**Pediatric**	**Pediatric**	**Pediatric**
**Cell lineage**		**Myeloid**	**B-cell**	**B-cell**	**T-cell**	**T-cell**
**Sub-type of leukemia**		**AML**	**ALL**	**ALL**	**ALL**	**ALL**
**Name**	**Description**	**Kas Dx**	**RS4 Dx**	**SUP Dx**	**C7-14 Dx**	**C1-6 Dx**
		**Stat. sign**	**Stat. sign**	**Stat. sign**	**Stat. sign**	**Stat. sign**
76P	gamma tubulin ring complex protein (76p gene)	**1.6**	**-1.9**	**-1.6**		
AARS	alanyl-tRNA synthetase	**-1.5**	**-2.0**	**-1.9**	**-1.3**	
ABAT	4-aminobutyrate aminotransferase	**14.5**	**-21.9**			
ABCB10	ATP-binding cassette, sub-family B (MDR/TAP), member 10	**-1.7**		**1.3**		
ABCB6	ATP-binding cassette, sub-family B (MDR/TAP), member 6	**3.4**	**-1.6**		**1.3**	
ABCC1	ATP-binding cassette, sub-family C (CFTR/MRP), member 1	**-3.3**	**1.9**	**-2.0**		**1.5**
ABCD3	ATP-binding cassette, sub-family D (ALD), member 3	**-1.8**				
ABCE1	ATP-binding cassette, sub-family E (OABP), member 1	**-4.2**	**1.4**	**-2.0**	**-1.5**	
ABCF1	ATP-binding cassette, sub-family F (GCN20), member 1	**-2.0**		**-1.5**		
ABCF2	ATP-binding cassette, sub-family F (GCN20), member 2	**-1.9**		**-1.9**		
ABI1	abl-interactor 1	**2.8**	**1.4**	**1.7**		
ABI2	abl interactor 2	**-2.7**	**1.3**	**-1.9**		
ABL1	v-abl Abelson murine leukemia viral oncogene homolog 1	**-1.4**				
ABR	active BCR-related gene	**1.4**				
ACAA1	acetyl-Coenzyme A acyltransferase 1 (peroxisomal 3-oxoacyl-Coenzyme A thiolase)	**1.2**		**-1.3**		
ACACA	acetyl-Coenzyme A carboxylase alpha	**-6.5**				
ACADM	acyl-Coenzyme A dehydrogenase, C-4 to C-12 straight chain	**1.7**	**-1.8**			
ACBD3	acyl-Coenzyme A binding domain containing 3	**1.5**	**-2.0**	**1.4**		
ACIN1	apoptotic chromatin condensation inducer 1	**1.6**				
ACLY	ATP citrate lyase	**-1.3**	**-1.4**	**-1.7**		
ACO1	aconitase 1, soluble	**-1.2**	**1.4**		**1.3**	**1.4**
ACO2	aconitase 2, mitochondrial	**-1.6**				
ACOT2	acyl-CoA thioesterase 2	**-7.8**	**5.0**			
ACOT7	acyl-CoA thioesterase 7	**-1.3**	**-1.6**	**-1.4**		
ACOX1	acyl-Coenzyme A oxidase 1, palmitoyl	**-3.5**	**5.2**			
ACP1	acid phosphatase 1, soluble	**-1.8**	**-2.1**			
ACSL3	acyl-CoA synthetase long-chain family member 3	**2.2**	**-1.8**	**-1.3**		
ACTB	actin, beta	**1.6**	**-1.5**			
ACTL6A	actin-like 6A	**-1.6**	**-1.5**	**-1.3**		
ACTN1	actinin, alpha 1	**-3.5**	**5.4**	**-1.2**		
ACTN4	actinin, alpha 4	**1.7**				
ACTR2	ARP2 actin-related protein 2 homolog (yeast)	**1.6**	**-1.4**	**1.3**	**1.2**	
ACTR3	ARP3 actin-related protein 3 homolog (yeast)	**-1.6**	**2.0**			
ACVR1B	activin A receptor, type IB	**-1.5**	**1.7**			

ACY1	aminoacylase 1	**-4.3**	**1.7**			
ACYP2	acylphosphatase 2, muscle type	**1.4**				
ADA	adenosine deaminase	**3.4**	**-9.1**	**1.3**	**-1.3**	**-1.5**
ADAM10	ADAM metallopeptidase domain 10	**2.8**	**-3.0**	**2.1**		
ADAM17	ADAM metallopeptidase domain 17 (tumor necrosis factor, alpha, converting enzyme)	**1.8**	**-1.7**	**1.5**		
ADAM9	ADAM metallopeptidase domain 9 (meltrin gamma)	**2.1**	**2.4**	**1.6**		
ADARB1	adenosine deaminase, RNA-specific, B1 (RED1 homolog rat)	**1.7**	**-34.5**			
ADCY3	adenylate cyclase 3	**-1.8**				
ADCY7	adenylate cyclase 7	**3.7**	**-3.1**	**-1.5**		
ADD1	adducin 1 (alpha)	**2.0**	**1.2**	**1.8**		
ADD3	adducin 3 (gamma)	**2.8**		**2.7**		
ADFP	adipose differentiation-related protein	**-2.0**	**3.3**	**1.5**		
ADH5	alcohol dehydrogenase 5 (class III), chi polypeptide	**-2.1**	**2.4**			
ADK	adenosine kinase	**-4.1**	**1.6**	**-2.0**		
ADNP	activity-dependent neuroprotector	**1.5**	**-1.4**	**-1.2**		
ADRBK1	adrenergic, beta, receptor kinase 1	**-1.3**	**-2.0**			
ADRBK2	adrenergic, beta, receptor kinase 2	**-2.2**	**1.6**	**-1.5**		
ADRM1	adhesion regulating molecule 1	**-3.5**	**2.2**			
ADSL	adenylosuccinate lyase	**-2.4**	**1.5**	**-1.5**		
AEBP1	AE binding protein 1	**2.1**	**-1.6**	**1.5**		
AES	amino-terminal enhancer of split	**3.0**	**-2.1**		**1.2**	
AFF1	AF4/FMR2 family, member 1	**8.6**	**-3.5**	**2.8**		
AFG3L2	AFG3 ATPase family gene 3-like 2 (yeast)	**-2.0**	**1.3**		**-1.3**	
AGA	aspartylglucosaminidase	**-1.7**	**-1.3**			
AGGF1	angiogenic factor with G patch and FHA domains 1	**-1.8**	**1.8**			
AGL	amylo-1, 6-glucosidase, 4-alpha-glucanotransferase (glycogen debranching enzyme, glycogen storage disease type III)	**3.3**	**-2.0**			
AGPAT1	1-acylglycerol-3-phosphate O-acyltransferase 1 (lysophosphatidic acid acyltransferase, alpha)	**1.3**	**1.8**	**-1.4**	**1.4**	
AGPAT2	1-acylglycerol-3-phosphate O-acyltransferase 2 (lysophosphatidic acid acyltransferase, beta)	**-1.4**	**1.5**			
AGPS	alkylglycerone phosphate synthase	**-2.6**	**5.5**	**5.0**		
AHCY	S-adenosylhomocysteine hydrolase	**-2.7**	**1.4**	**-1.4**		
AHCYL1	S-adenosylhomocysteine hydrolase-like 1	**-1.6**	**1.6**	**-1.4**		
AHSA1	AHA1, activator of heat shock 90kDa protein ATPase homolog 1 (yeast)	**-1.4**		**-1.7**		
AIF1	allograft inflammatory factor 1	**-39.1**	**2.5**			
AIM1	absent in melanoma 1	**8.3**			**3.5**	**13.8**
AK2	adenylate kinase 2	**-3.3**	**-1.4**	**-2.2**		**-1.6**
AKAP1	A kinase (PRKA) anchor protein 1	**-2.2**	**-1.8**	**-2.4**	**-1.6**	
AKAP10	A kinase (PRKA) anchor protein 10	**2.9**	**-2.7**			
AKAP11	A kinase (PRKA) anchor protein 11	**-1.5**	**-1.2**	**-1.3**		
AKAP2	A kinase (PRKA) anchor protein 2	**2.2**		**4.0**		
AKAP8	A kinase (PRKA) anchor protein 8	**-1.9**				
AKAP9	A kinase (PRKA) anchor protein (yotiao) 9	**1.6**				
AKR1B1	aldo-keto reductase family 1, member B1 (aldose reductase)	**8.1**	**-4.2**	**-1.2**		

AKR1C3	aldo-keto reductase family 1, member C3 (3-alpha hydroxysteroid dehydrogenase, type II)	**90.6**		**3.7**		
AKR7A2	aldo-keto reductase family 7, member A2 (aflatoxin aldehyde reductase)	**-1.3**	**-1.6**			
AKT1	v-akt murine thymoma viral oncogene homolog 1	**1.3**	**-1.5**			
ALAS1	aminolevulinate, delta-, synthase 1	**-1.9**	**2.4**			
ALDH4A1	aldehyde dehydrogenase 4 family, member A1	**-3.5**	**1.9**		**1.6**	
ALDH5A1	aldehyde dehydrogenase 5 family, member A1 (succinate-semialdehyde dehydrogenase)	**-2.2**	**-1.5**	**-1.4**		
ALDH9A1	aldehyde dehydrogenase 9 family, member A1	**-1.6**		**-1.6**		
ALDOA	aldolase A, fructose-bisphosphate	**-1.3**	**-1.5**	**-1.3**		
ALOX5AP	arachidonate 5-lipoxygenase-activating protein	**2.3**			**2.4**	**1.8**
ALS2CR3	trafficking protein, kinesin binding 2	**2.8**	**1.7**	**2.4**		
AMD1	adenosylmethionine decarboxylase 1	**-2.4**	**1.4**		**-1.4**	
AMFR	autocrine motility factor receptor	**1.3**				
AMPD2	adenosine monophosphate deaminase 2 (isoform L)	**-2.9**	**1.9**	**-2.5**		
ANAPC10	anaphase promoting complex subunit 10	**-1.3**	**1.8**			
ANAPC13	anaphase promoting complex subunit 13	**-1.7**	**1.2**	**-1.3**		
ANAPC5	anaphase promoting complex subunit 5	**-2.3**	**1.5**	**-1.8**		
ANGPT1	angiopoietin 1	**-8.8**	**1.8**			
ANKRD28	ankyrin repeat domain 28	**-4.1**	**2.2**	**2.2**		**-2.0**
ANKRD40	ankyrin repeat domain 40	**1.4**	**-1.5**			
ANKS1	ankyrin repeat and sterile alpha motif domain containing 1A	**-1.4**		**3.3**		
ANP32A	acidic (leucine-rich) nuclear phosphoprotein 32 family, member A	**-1.8**	**2.1**	**-1.3**		
ANP32B	acidic (leucine-rich) nuclear phosphoprotein 32 family, member B	**-1.6**		**-1.3**		
ANXA11	annexin A11	**-1.2**	**1.8**	**1.8**		
ANXA2	annexin A2	**2.7**		**1.4**		
ANXA4	annexin A4	**1.8**		**1.3**		
ANXA6	annexin A6	**-1.3**	**2.4**			**1.3**
ANXA7	annexin A7	**-1.3**	**1.6**			
AOF2	amine oxidase (flavin containing) domain 2	**-1.9**		**1.5**		
AP1B1	adaptor-related protein complex 1, beta 1 subunit	**2.2**	**-1.4**			
AP1G1	adaptor-related protein complex 1, gamma 1 subunit	**1.6**		**-1.2**		
AP1G2	adaptor-related protein complex 1, gamma 2 subunit	**2.3**				
AP1S2	adaptor-related protein complex 1, sigma 2 subunit	**21.8**	**-27.2**	**1.6**	**1.8**	
AP2B1	adaptor-related protein complex 2, beta 1 subunit	**1.6**		**1.6**		
AP2S1	adaptor-related protein complex 2, sigma 1 subunit	**-1.9**			**1.3**	
AP3B1	adaptor-related protein complex 3, beta 1 subunit	**1.6**				
AP3S1	adaptor-related protein complex 3, sigma 1 subunit	**2.1**	**1.4**	**2.4**	**1.6**	**1.9**
AP3S2	adaptor-related protein complex 3, sigma 2 subunit	**-1.8**	**2.2**			
APEX1	APEX nuclease (multifunctional DNA repair enzyme) 1	**-3.4**		**-2.2**		
APG12L	ATG12 autophagy related 12 homolog (S. cerevisiae)	**2.5**	**1.3**	**1.9**	**1.6**	**1.9**
API5	apoptosis inhibitor 5	**-2.0**	**1.6**	**-2.1**		
APLP2	amyloid beta (A4) precursor-like protein 2	**-12.0**	**4.2**	**-1.7**		
APOBEC3B	apolipoprotein B mRNA editing enzyme, catalytic polypeptide-like 3B	**2.0**	**-1.9**			

APPBP1	amyloid beta precursor protein binding protein 1	-1.2	-1.2	-1.3		
APPBP2	amyloid beta precursor protein (cytoplasmic tail) binding protein 2	-1.3	2.0	1.4		
APRT	adenine phosphoribosyltransferase	-2.6		-1.7		
ARF1	ADP-ribosylation factor 1	-1.2	1.3	-1.2		
ARF3	ADP-ribosylation factor 3	-1.3	1.4	-1.3	1.3	
ARF4	ADP-ribosylation factor 4	-1.6	1.3			
ARF5	ADP-ribosylation factor 5	2.0	-2.4			
ARF6	ADP-ribosylation factor 6	1.7	1.4	1.7		
ARFGEF1	ADP-ribosylation factor guanine nucleotide-exchange factor 1(brefeldin A-inhibited)	1.8	-1.6	1.5		
ARFGEF2	ADP-ribosylation factor guanine nucleotide-exchange factor 2 (brefeldin A-inhibited)	-1.2	1.3			
ARFIP2	ADP-ribosylation factor interacting protein 2 (arfaptin 2)	-1.9				
ARHGAP1	Rho GTPase activating protein 1	1.2				
ARHGAP5	Rho GTPase activating protein 5	2.3	-1.4			
ARHGDIB	Rho GDP dissociation inhibitor (GDI) beta	1.5	-1.4			
ARHGEF18	rho/rac guanine nucleotide exchange factor (GEF) 18	3.3	1.5	2.5	1.3	
ARHGEF2	rho/rac guanine nucleotide exchange factor (GEF) 2	-1.7	1.3	1.3		
ARHGEF7	Rho guanine nucleotide exchange factor (GEF) 7	5.1	-1.8	3.6		
ARID5B	AT rich interactive domain 5B (MRF1-like)	8.6	-34.1	-2.2		
ARIH1	ariadne homolog, ubiquitin-conjugating enzyme E2 binding protein, 1 (Drosophila)	1.8	1.7	1.6		
ARIH2	ariadne homolog 2 (Drosophila)	-2.4	-1.5	1.6		
ARL1	ADP-ribosylation factor-like 1	-1.5	1.3		-1.2	
ARL2	ADP-ribosylation factor-like 2	-1.7				
ARL2BP	ADP-ribosylation factor-like 2 binding protein	1.7				
ARL4A	ADP-ribosylation factor-like 4A	-33.8	26.8			
ARL6IP	ADP-ribosylation factor-like 6 interacting protein 1	2.7	-2.2	1.7		
ARL6IP5	ADP-ribosylation-like factor 6 interacting protein 5	2.2		1.4		
ARMET	arginine-rich, mutated in early stage tumors	-1.6		1.4		
ARPC1A	actin related protein 2/3 complex, subunit 1A, 41kDa	2.2	-2.8			
ARPC2	actin related protein 2/3 complex, subunit 2, 34kDa	1.4	-1.3			
ARPC4	actin related protein 2/3 complex, subunit 4, 20kDa	4.1	-4.7			
ARPC5	actin related protein 2/3 complex, subunit 5, 16kDa	2.3	-1.4	1.4		
ARPP-19	cyclic AMP phosphoprotein, 19 kD	2.4	-2.5			
ARRB2	arrestin, beta 2	1.3	-2.1			
ASAH1	N-acylsphingosine amidohydrolase (acid ceramidase) 1	-3.8	4.4	-1.3		
ASB1	ankyrin repeat and SOCS box-containing 1	1.4	-1.8			
ASCC3	activating signal cointegrator 1 complex subunit 3	-1.5				-2.2
ASF1A	ASF1 anti-silencing function 1 homolog A (S. cerevisiae)	-1.2				
ASMTL	acetylserotonin O-methyltransferase-like	3.2	-2.7			
ASNA1	arsA arsenite transporter, ATP-binding, homolog 1 (bacterial)	-1.3		-1.4		
ASNS	asparagine synthetase	-9.0	2.9			
ASS	argininosuccinate synthetase 1	-2.0	-1.4	-3.8		
ASXL1	additional sex combs like 1 (Drosophila)	-2.2	-1.6	-1.4		

ATF2	activating transcription factor 2	-1.5	1.6	1.5		
ATIC	5-aminoimidazole-4-carboxamide ribonucleotide formyltransferase/IMP cyclohydrolase	-1.3	-2.1	-3.2	-1.4	-2.0
ATOX1	ATX1 antioxidant protein 1 homolog (yeast)	-13.3	5.6			
ATP11B	ATPase, Class VI, type 11B	4.7	-1.6	5.1		1.5
ATP1A1	ATPase, Na+/K+ transporting, alpha 1 polypeptide	-2.4	1.3	-1.6		
ATP1B3	ATPase, Na+/K+ transporting, beta 3 polypeptide	1.9				
ATP2A2	ATPase, Ca++ transporting, cardiac muscle, slow twitch 2	-2.5	1.3	-1.5		
ATP2A3	ATPase, Ca++ transporting, ubiquitous	-2.0	2.5	1.2		
ATP2B4	ATPase, Ca++ transporting, plasma membrane 4	3.9	-3.8	2.8		
ATP2C1	ATPase, Ca++ transporting, type 2C, member 1	1.5	1.9			
ATP5A1	ATP synthase, H+ transporting, mitochondrial F1 complex, alpha subunit 1, cardiac muscle	-1.5	1.3			
ATP5C1	ATP synthase, H+ transporting, mitochondrial F1 complex, gamma polypeptide 1	-1.5	1.4			
ATP5D	ATP synthase, H+ transporting, mitochondrial F1 complex, delta subunit	-1.7				
ATP5F1	ATP synthase, H+ transporting, mitochondrial F0 complex, subunit B1	1.3				
ATP5G1	ATP synthase, H+ transporting, mitochondrial F0 complex, subunit C1 (subunit 9)	-3.9		-2.4		-1.3
ATP5G3	ATP synthase, H+ transporting, mitochondrial F0 complex, subunit C3 (subunit 9)	-3.8	2.3			
ATP5H	ATP synthase, H+ transporting, mitochondrial F0 complex, subunit d	-2.4	1.5	-1.2		
ATP5J	ATP synthase, H+ transporting, mitochondrial F0 complex, subunit F6	3.0	-1.8			
ATP5L	ATP synthase, H+ transporting, mitochondrial F0 complex, subunit G	-1.6	1.7	1.6		
ATP5O	ATP synthase, H+ transporting, mitochondrial F1 complex, O subunit (oligomycin sensitivity conferring protein)	-1.8		-1.2		
ATP5S	ATP synthase, H+ transporting, mitochondrial F0 complex, subunit s (factor B)	2.0		2.9		
ATP6AP2	ATPase, H+ transporting, lysosomal accessory protein 2	-1.8	2.2	1.2		
ATP6V0A2	ATPase, H+ transporting, lysosomal V0 subunit a2	-1.6				
ATP6V0B	ATPase, H+ transporting, lysosomal 21kDa, V0 subunit b	-1.6				
ATP6V0C	ATPase, H+ transporting, lysosomal 16kDa, V0 subunit c	1.9	-1.4	1.3		
ATP6V0D1	ATPase, H+ transporting, lysosomal 38kDa, V0 subunit d1	-1.3	1.4		1.3	
ATP6V0E	ATPase, H+ transporting, lysosomal 9kDa, V0 subunit e1	-1.4	1.3			
ATP6V1A	ATPase, H+ transporting, lysosomal 70kDa, V1 subunit A	-1.9	1.9		1.5	
ATP6V1C1	ATPase, H+ transporting, lysosomal 42kDa, V1 subunit C1	2.1	-2.1		-1.4	
ATP6V1D	ATPase, H+ transporting, lysosomal 34kDa, V1 subunit D	1.9	-1.3	1.3		
ATP6V1E1	ATPase, H+ transporting, lysosomal 31kDa, V1 subunit E1	1.4				
ATP6V1G1	ATPase, H+ transporting, lysosomal 13kDa, V1 subunit G1	1.3				
ATP6V1H	ATPase, H+ transporting, lysosomal 50/57kDa, V1 subunit H	1.4				
ATR	ataxia telangiectasia and Rad3 related	-1.3				
ATRN	attractin	-1.4				
ATRX	alpha thalassemia/mental retardation syndrome X-linked (RAD54 homolog, S. cerevisiae)	1.7	1.4			
ATXN10	ataxin 10	-1.7			-1.3	-1.5
ATXN2L	ataxin 2-like	1.8	-2.2			
ATXN3	ataxin 3	2.0		1.3		
AUH	AU RNA binding protein/enoyl-Coenzyme A hydratase	2.4			2.5	1.7
AURKB	aurora kinase B	3.0	-3.3	1.4		
B2M	beta-2-microglobulin	3.1	-1.6			

B4GALT1	UDP-Gal:betaGlcNAc beta 1,4- galactosyltransferase, polypeptide 1	**1.7**		**1.3**		
B4GALT2	UDP-Gal:betaGlcNAc beta 1,4- galactosyltransferase, polypeptide 2	**-2.4**		**-2.1**	**-1.8**	
B4GALT3	UDP-Gal:betaGlcNAc beta 1,4- galactosyltransferase, polypeptide 3	**-1.4**				
B4GALT4	UDP-Gal:betaGlcNAc beta 1,4- galactosyltransferase, polypeptide 4	**8.1**				
B4GALT6	UDP-Gal:betaGlcNAc beta 1,4- galactosyltransferase, polypeptide 6	**-6.7**	**2.5**			
B930013M22RIK	ras responsive element binding protein 1	**1.5**	**3.1**	**2.1**		
BACH1	BTB and CNC homology 1, basic leucine zipper transcription factor 1	**1.2**	**1.2**	**1.3**		
BAG1	BCL2-associated athanogene	**-2.0**		**-1.4**		
BAG2	BCL2-associated athanogene 2	**-1.5**	**-1.2**	**-1.8**		
BAG5	BCL2-associated athanogene 5	**-1.4**				
BAMBI	BMP and activin membrane-bound inhibitor homolog (Xenopus laevis)	**1.5**	**-2.7**			**-1.7**
BARD1	BRCA1 associated RING domain 1	**3.4**	**-3.6**	**1.3**	**1.3**	
BAT2D1	BAT2 domain containing 1	**-1.4**				
BAT8	euchromatic histone-lysine N-methyltransferase 2	**-2.8**		**-4.0**		
BAZ1A	bromodomain adjacent to zinc finger domain, 1A	**-1.6**				**-1.6**
BAZ2A	bromodomain adjacent to zinc finger domain, 2A	**-2.9**				
BC-2	chromatin modifying protein 2A	**1.5**	**-1.4**	**1.5**		
BCAT2	branched chain aminotransferase 2, mitochondrial	**-2.7**	**1.7**		**-1.3**	
BCKDHA	branched chain keto acid dehydrogenase E1, alpha polypeptide	**-2.2**				
BCKDHB	branched chain keto acid dehydrogenase E1, beta polypeptide (maple syrup urine disease)	**-2.5**	**2.2**			
BCL11A	B-cell CLL/lymphoma 11A (zinc finger protein)	**1.2**	**-1.8**			
BCL2	B-cell CLL/lymphoma 2	**1.5**	**-11.2**	**-2.8**		
BCL2L11	BCL2-like 11 (apoptosis facilitator)	**5.1**	**1.2**	**3.1**	**3.1**	**5.9**
BCL7A	B-cell CLL/lymphoma 7A	**1.5**	**-3.3**	**-1.3**		
BCLAF1	BCL2-associated transcription factor 1	**-1.8**	**2.0**	**-1.7**	**-1.3**	
BCR	breakpoint cluster region	**4.7**	**-1.4**			
BDH1	3-hydroxybutyrate dehydrogenase, type 1	**-1.6**	**-1.3**		**-1.3**	**-1.5**
BECN1	beclin 1 (coiled-coil, myosin-like BCL2 interacting protein)	**1.7**				
BET1	BET1 homolog (S. cerevisiae)	**1.6**	**-2.2**			
BICD2	bicaudal D homolog 2 (Drosophila)	**-1.2**	**-1.2**			
BID	BH3 interacting domain death agonist	**-1.3**	**-3.4**	**-1.9**	**1.4**	
BIN1	bridging integrator 1	**9.6**	**-1.3**			**1.6**
BIRC2	baculoviral IAP repeat-containing 2	**1.6**	**1.4**	**1.9**	**1.9**	
BIRC5	baculoviral IAP repeat-containing 5 (survivin)	**2.4**	**-1.8**			
BLCAP	bladder cancer associated protein	**-1.4**	**1.4**	**-1.5**		
BLM	Bloom syndrome	**-2.6**		**-1.8**		
BLOC1S1	biogenesis of lysosome-related organelles complex-1, subunit 1	**1.6**	**-1.9**			
BMP2K	BMP2 inducible kinase	**-1.6**	**1.5**			
BNIP2	BCL2/adenovirus E1B 19kDa interacting protein 2	**1.4**	**-1.4**	**1.7**	**1.5**	
BNIP3	BCL2/adenovirus E1B 19kDa interacting protein 3	**-331.2**	**312.5**		**1.4**	
BNIP3L	BCL2/adenovirus E1B 19kDa interacting protein 3-like	**1.9**	**2.0**	**-1.3**		
BOP1	block of proliferation 1	**-3.6**		**-2.6**	**-1.4**	

BRCA1	breast cancer 1, early onset	-2.6	-1.5	-1.4		
BRD2	bromodomain containing 2	1.5				
BRD4	bromodomain containing 4	1.4	-1.3			
BRE	brain and reproductive organ-expressed (TNFRSF1A modulator)	3.0		-2.1		
BRP44	brain protein 44	-1.4		-1.3		
BRRN1	non-SMC condensin I complex, subunit H	-1.8	-1.9			
BSG	basigin (Ok blood group)	-2.4		-1.4		
BST2	bone marrow stromal cell antigen 2	1.4	-1.5			
BTAF1	BTAF1 RNA polymerase II, B-TFIID transcription factor-associated, 170kDa (Mot1 homolog, S. cerevisiae)	-2.0	1.6			
BTBD2	BTB (POZ) domain containing 2	1.6		-1.8		
BTG1	B-cell translocation gene 1, anti-proliferative	8.8		3.9	9.3	4.4
BTG2	BTG family, member 2	58.4		3.9	3.1	2.3
BTG3	BTG family, member 3	-2.2	-1.6			
BTN3A2	butyrophilin, subfamily 3, member A2	3.9	-5.8			
BTN3A3	butyrophilin, subfamily 3, member A3	4.2	-5.9	-1.4		
BUB1	BUB1 budding uninhibited by benzimidazoles 1 homolog (yeast)	-1.3	-1.3	1.4		
BUB3	BUB3 budding uninhibited by benzimidazoles 3 homolog (yeast)	-1.4	-1.4	1.3		
BYSL	bystin-like	-4.0	-6.2	-8.4	-2.2	-2.3
BZRP	translocator protein (18kDa)	-1.4		-1.8		
C13ORF24	chromosome 13 open reading frame 24	-1.5	1.2			
C19ORF10	chromosome 19 open reading frame 10	-2.2	1.3			
C19ORF2	chromosome 19 open reading frame 2	-2.7	1.8	-1.3		
C1ORF16	Smg-7 homolog, nonsense mediated mRNA decay factor (C. elegans)	-2.0				
C1ORF48	NSL1, MIND kinetochore complex component, homolog (S. cerevisiae)	1.4	-1.5			
C20ORF18	RanBP-type and C3HC4-type zinc finger containing 1	1.3				
C21ORF33	chromosome 21 open reading frame 33	-2.5	1.8		1.2	
C5ORF13	chromosome 5 open reading frame 13	4.7	-3.7	-3.3	-1.3	
C5ORF18	receptor accessory protein 5	-4.6	3.6	1.4		
C5ORF22	chromosome 5 open reading frame 22	-1.3	1.2			
C6ORF108	chromosome 6 open reading frame 108	-51.4	2.7	-3.3		
C6ORF69	potassium channel tetramerisation domain containing 20	1.2	-1.3			
C7ORF44	chromosome 7 open reading frame 44	-4.8	2.8			
CACNB3	calcium channel, voltage-dependent, beta 3 subunit	1.2		1.4	1.9	
CAD	carbamoyl-phosphate synthetase 2, aspartate transcarbamylase, and dihydroorotase	-3.7		-2.8		
CALM1	calmodulin 1 (phosphorylase kinase, delta)	4.4	-1.4	1.6		
CALM3	calmodulin 3 (phosphorylase kinase, delta)	1.3	-1.5	1.2		
CALR	calreticulin	-5.3	4.7	-1.4	-1.9	
CALU	calumenin	-1.3		-2.6		
CAMK2G	calcium/calmodulin-dependent protein kinase (CaM kinase) II gamma	-2.6	2.1			
CAMK4	calcium/calmodulin-dependent protein kinase IV	8.0	-5.8			
CAMKK2	calcium/calmodulin-dependent protein kinase kinase 2, beta	-3.7	1.7	-2.1		
CAMLG	calcium modulating ligand	-2.0	2.9	-1.5		

CAMTA2	calmodulin binding transcription activator 2	**2.0**	**-1.5**		**1.4**	
CANX	calnexin	**-2.5**	**1.6**	**-1.3**		
CAP1	CAP, adenylate cyclase-associated protein 1 (yeast)	**1.7**	**1.3**	**1.3**		
CAP350	centrosomal protein 350kDa	**1.5**	**1.6**	**2.3**		
CAPG	capping protein (actin filament), gelsolin-like	**61.6**	**-49.5**			
CAPN1	calpain 1, (mu/I) large subunit	**2.8**				
CAPN2	calpain 2, (m/II) large subunit	**-2.2**	**2.4**			
CAPNS1	calpain, small subunit 1	**1.6**	**-1.3**			
CAPZA2	capping protein (actin filament) muscle Z-line, alpha 2	**1.9**	**-2.1**	**1.8**		
CAPZB	capping protein (actin filament) muscle Z-line, beta	**1.2**				
CARM1	coactivator-associated arginine methyltransferase 1	**-1.4**	**-1.8**	**-1.7**	**-1.6**	
CASK	calcium/calmodulin-dependent serine protein kinase (MAGUK family)	**1.2**	**-1.3**			
CASP2	caspase 2, apoptosis-related cysteine peptidase (neural precursor cell expressed, developmentally down-regulated 2)	**2.2**	**-3.1**	**-1.8**		
CASP4	caspase 4, apoptosis-related cysteine peptidase	**1.9**		**2.0**	**1.5**	
CASP6	caspase 6, apoptosis-related cysteine peptidase	**-1.8**	**2.4**	**1.4**		
CASP8	caspase 8, apoptosis-related cysteine peptidase	**-1.5**	**3.2**	**1.5**		
CASP9	caspase 9, apoptosis-related cysteine peptidase	**1.3**	**-1.3**			
CAT	catalase	**1.4**	**4.5**	**-1.3**	**1.3**	
CBFA2T2	core-binding factor, runt domain, alpha subunit 2; translocated to, 2	**-1.3**	**1.5**			
CBFA2T3	core-binding factor, runt domain, alpha subunit 2; translocated to, 3	**1.3**		**1.5**	**-2.1**	
CBFB	core-binding factor, beta subunit	**1.5**	**-2.3**			
CBLB	Cas-Br-M (murine) ecotropic retroviral transforming sequence b	**4.1**	**-1.6**	**1.8**		
CBX1	chromobox homolog 1 (HP1 beta homolog Drosophila)	**-1.6**				
CBX3	chromobox homolog 3 (HP1 gamma homolog, Drosophila)	**-2.1**	**2.2**	**1.2**		
CBX6	chromobox homolog 6	**-2.0**	**-1.4**	**-2.1**		
CCDC28A	coiled-coil domain containing 28A	**1.8**	**-1.3**		**1.6**	
CCDC6	coiled-coil domain containing 6	**-4.5**	**2.6**	**1.9**		
CCDC85B	coiled-coil domain containing 85B	**-4.1**		**-1.8**		
CCNA2	cyclin A2	**1.5**	**-2.2**	**1.3**		
CCNB1	cyclin B1	**2.6**	**-1.5**	**1.9**		
CCNB2	cyclin B2	**1.4**		**1.4**	**1.4**	
CCNC	cyclin C	**-1.4**	**1.3**			
CCND3	cyclin D3	**7.2**	**-10.6**			**-1.8**
CCNE1	cyclin E1	**-14.9**	**2.3**			
CCNE2	cyclin E2	**2.2**	**-2.9**	**-1.3**	**1.2**	
CCNF	cyclin F	**2.7**	**-2.6**	**2.0**		
CCNG2	cyclin G2	**3.1**		**2.2**	**2.3**	
CCNH	cyclin H	**-1.5**	**1.6**	**1.3**		
CCNI	cyclin I	**1.6**	**-1.7**	**1.5**		
CCNT2	cyclin T2	**-2.0**	**2.6**	**2.0**		
CCT2	chaperonin containing TCP1, subunit 2 (beta)	**-2.1**		**-2.2**	**-1.3**	
CCT3	chaperonin containing TCP1, subunit 3 (gamma)	**-1.7**	**1.2**	**-1.6**		

CCT4	chaperonin containing TCP1, subunit 4 (delta)	**-1.4**		**-1.4**		
CCT5	chaperonin containing TCP1, subunit 5 (epsilon)	**-1.5**		**-1.5**	**-1.6**	
CCT6A	chaperonin containing TCP1, subunit 6A (zeta 1)	**-5.1**	**4.9**		**-1.6**	**-1.7**
CCT7	chaperonin containing TCP1, subunit 7 (eta)	**-2.1**		**-1.5**		**-1.6**
CCT8	chaperonin containing TCP1, subunit 8 (theta)	**-1.3**	**1.2**	**-1.6**		
CD164	CD164 molecule, sialomucin	**2.6**	**-1.7**	**1.8**		
CD2AP	CD2-associated protein	**1.4**				
CD34	CD34 molecule	**-18.1**	**12.3**	**-3.0**		
CD37	CD37 molecule	**-4.9**	**2.5**			
CD38	CD38 molecule	**8.4**	**-2.9**	**-2.4**		
CD40	CD40 molecule, TNF receptor superfamily member 5	**1.3**	**-1.4**			
CD44	CD44 molecule (Indian blood group)	**-13.1**	**3.2**	**-3.0**	**-1.7**	
CD47	CD47 molecule	**2.0**	**-2.4**	**-1.7**	**1.7**	
CD48	CD48 molecule	**4.3**	**-14.5**	**-2.5**	**1.9**	
CD52	CD52 molecule	**98.6**				
CD53	CD53 molecule	**7.2**	**1.4**	**3.1**	**3.1**	**3.2**
CD58	CD58 molecule	**2.5**		**3.0**		
CD59	CD59 molecule, complement regulatory protein	**10.6**	**-3.7**			**1.8**
CD69	CD69 molecule	**-1.5**	**6.3**	**3.8**	**4.9**	**2.0**
CD72	CD72 molecule	**31.2**	**-23.3**	**1.6**		
CD79A	CD79a molecule, immunoglobulin-associated alpha	**24.6**	**-24.9**	**1.3**	**2.0**	**3.6**
CD81	CD81 molecule	**1.3**	**-1.9**			
CD99	CD99 molecule	**2.6**	**1.7**	**3.4**		**1.8**
CDC123	cell division cycle 123 homolog (S. cerevisiae)	**-2.0**		**-1.7**	**-1.3**	
CDC23	cell division cycle 23 homolog (S. cerevisiae)	**-2.1**	**-1.9**	**-1.3**		
CDC25A	cell division cycle 25 homolog A (S. cerevisiae)	**-19.4**	**-1.6**	**-1.4**	**-1.4**	
CDC25B	cell division cycle 25 homolog B (S. cerevisiae)	**1.6**	**-1.2**	**1.6**		
CDC27	cell division cycle 27 homolog (S. cerevisiae)	**-1.5**		**1.4**		
CDC2L6	cell division cycle 2-like 6 (CDK8-like)	**3.8**	**-1.4**	**3.3**		**1.8**
CDC5L	CDC5 cell division cycle 5-like (S. pombe)	**1.6**		**1.3**		
CDC6	cell division cycle 6 homolog (S. cerevisiae)	**-4.7**	**-1.7**	**-2.9**	**-1.3**	**-1.5**
CDC7	cell division cycle 7 homolog (S. cerevisiae)	**-2.4**	**-1.4**	**-1.4**	**1.3**	
CDH2	cadherin 2, type 1, N-cadherin (neuronal)	**-72.0**	**30.0**			
CDIPT	CDP-diacylglycerol--inositol 3-phosphatidyltransferase (phosphatidylinositol synthase)	**2.8**	**-1.6**	**1.4**		
CDK2	cyclin-dependent kinase 2	**-1.6**	**-2.3**	**-1.5**		
CDK4	cyclin-dependent kinase 4	**-4.4**		**-2.4**	**-1.4**	**-1.6**
CDK5	cyclin-dependent kinase 5	**1.6**				
CDK6	cyclin-dependent kinase 6	**2.1**	**-3.0**	**-1.5**		
CDK7	cyclin-dependent kinase 7 (MO15 homolog, Xenopus laevis, cdk-activating kinase)	**-1.6**	**2.1**			
CDK9	cyclin-dependent kinase 9 (CDC2-related kinase)	**10.9**	**-7.3**	**1.7**		
CDKN1B	cyclin-dependent kinase inhibitor 1B (p27, Kip1)	**3.6**	**-1.6**	**1.6**	**1.7**	
CDKN2C	cyclin-dependent kinase inhibitor 2C (p18, inhibits CDK4)	**1.6**		**1.3**		

CDKN2D	cyclin-dependent kinase inhibitor 2D (p19, inhibits CDK4)	11.1				
CDKN3	cyclin-dependent kinase inhibitor 3 (CDK2-associated dual specificity phosphatase)	1.2	-1.4	1.5		
CDS2	CDP-diacylglycerol synthase (phosphatidate cytidylyltransferase) 2	1.5		1.6		
CDT1	chromatin licensing and DNA replication factor 1	-4.6				
CEBPB	CCAAT/enhancer binding protein (C/EBP), beta	-3.1	1.5	-1.5		
CEBPG	CCAAT/enhancer binding protein (C/EBP), gamma	-1.6	-1.3			
CEBPZ	CCAAT/enhancer binding protein zeta	-2.5	-1.7	-1.5	-1.5	-1.8
CENPA	centromere protein A	1.4	-1.3	1.7		
CENPB	centromere protein B, 80kDa	-1.5				
CENPE	centromere protein E, 312kDa	2.0		1.6		1.4
CENPF	centromere protein F, 350/400ka (mitosin)	2.3	-2.3	1.5		
CENTB1	centaurin, beta 1	42.8	-21.1			
CENTD1	centaurin, delta 1	3.3		1.8		
CFL1	cofilin 1 (non-muscle)	1.3	-1.6			
CGGBP1	CGG triplet repeat binding protein 1	1.4				
CHAF1A	chromatin assembly factor 1, subunit A (p150)	-2.7		-2.3		
CHAF1B	chromatin assembly factor 1, subunit B (p60)	-2.4	-1.7			
CHC1	regulator of chromosome condensation 1	-1.7		-1.8	-1.7	-1.6
CHD1	chromodomain helicase DNA binding protein 1	-1.3		-1.3		
CHD3	chromodomain helicase DNA binding protein 3	5.0	-9.4	-1.3		
CHEK1	CHK1 checkpoint homolog (S. pombe)	-2.1	-1.8	-1.3		
CHERP	calcium homeostasis endoplasmic reticulum protein	-1.6		-1.4		
CHES1	checkpoint suppressor 1	1.5	1.8	1.6	1.5	
CHP	calcium binding protein P22	1.6	2.4			
CHRNA5	cholinergic receptor, nicotinic, alpha 5	1.7	-2.2			
CHST10	carbohydrate sulfotransferase 10	-2.6	2.3			
CHSY1	carbohydrate (chondroitin) synthase 1	-2.1	1.8	2.5		
CHUK	conserved helix-loop-helix ubiquitous kinase	-1.5	1.5			
CIAPIN1	cytokine induced apoptosis inhibitor 1	-1.8	1.3	-1.6		
CIC	capicua homolog (Drosophila)	2.4				
CITED2	Cbp/p300-interacting transactivator, with Glu/Asp-rich carboxy-terminal domain, 2	-7.0	10.5	2.8		
CKB	creatine kinase, brain	-5.0	3.2			
CKS1B	CDC28 protein kinase regulatory subunit 1B	-1.4	-1.6			
CLASP1	cytoplasmic linker associated protein 1	1.8	1.5			1.7
CLASP2	cytoplasmic linker associated protein 2	2.6	-1.7	1.7		
CLCN3	chloride channel 3	3.5	-3.0			
CLCN7	chloride channel 7	-1.3				
CLEC11A	C-type lectin domain family 11, member A	-10.9	1.5	1.4		
CLIC4	chloride intracellular channel 4	2.0	-2.2			
CLK1	CDC-like kinase 1	1.6	1.5	1.7		
CLOCK	clock homolog (mouse)	-1.4				
CLPP	ClpP caseinolytic peptidase, ATP-dependent, proteolytic subunit homolog (E. coli)	-1.3		-1.3	1.5	

CLSTN1	calsyntenin 1	**-1.4**	**1.3**		**1.3**	
CLTA	clathrin, light chain (Lca)	**-1.3**	**1.4**			
CLTB	clathrin, light chain (Lcb)	**-1.3**	**-2.0**	**-1.4**		
CLTC	clathrin, heavy chain (Hc)	**-1.3**	**1.4**			**-1.5**
CNAP1	non-SMC condensin I complex, subunit D2	**2.2**	**-2.7**			
CNOT1	CCR4-NOT transcription complex, subunit 1	**-1.4**		**-1.2**	**1.2**	
COG2	component of oligomeric golgi complex 2	**-1.7**				
COG4	component of oligomeric golgi complex 4	**1.4**				
COIL	coilin	**-2.0**				
COMMD4	COMM domain containing 4	**-1.5**				
COPA	coatomer protein complex, subunit alpha	**1.9**	**1.2**			
COPS2	COP9 constitutive photomorphogenic homolog subunit 2 (Arabidopsis)	**-2.4**	**1.6**	**-1.2**		
COPS6	COP9 constitutive photomorphogenic homolog subunit 6 (Arabidopsis)	**-1.8**	**-1.7**	**-1.5**		
COPS7A	COP9 constitutive photomorphogenic homolog subunit 7A (Arabidopsis)	**1.3**	**-1.4**			
COPS8	COP9 constitutive photomorphogenic homolog subunit 8 (Arabidopsis)	**2.6**	**-3.2**			
COX11	COX11 homolog, cytochrome c oxidase assembly protein (yeast)	**-2.9**	**4.1**	**-1.3**		
COX5A	cytochrome c oxidase subunit Va	**-1.6**				
COX5B	cytochrome c oxidase subunit Vb	**-1.6**		**-1.2**		
COX6B1	cytochrome c oxidase subunit Vib polypeptide 1 (ubiquitous)	**-1.5**				
COX7B	cytochrome c oxidase subunit VIIb	**-1.7**		**-1.3**		
CPD	carboxypeptidase D	**-4.0**	**25.6**		**1.4**	
CPNE1	copine I	**2.1**	**-1.5**			
CPNE3	copine III	**2.7**	**-1.5**			
CPOX	coproporphyrinogen oxidase	**-2.6**	**1.8**		**1.5**	
CPSF1	cleavage and polyadenylation specific factor 1, 160kDa	**-2.3**	**2.3**			
CPSF5	nudix (nucleoside diphosphate linked moiety X)-type motif 21	**-2.2**	**-1.8**	**-1.3**		
CPSF6	cleavage and polyadenylation specific factor 6, 68kDa	**-2.0**	**-1.6**			
CPT1A	carnitine palmitoyltransferase 1A (liver)	**-2.0**	**1.6**			
CRADD	CASP2 and RIPK1 domain containing adaptor with death domain	**1.8**		**1.5**		
CREB1	cAMP responsive element binding protein 1	**2.6**	**-1.5**	**1.4**		
CREBBP	CREB binding protein (Rubinstein-Taybi syndrome)	**14.0**	**-10.0**	**1.3**		
CREBL2	cAMP responsive element binding protein-like 2	**3.0**	**-2.6**			
CREBZF	CREB/ATF bZIP transcription factor	**-1.9**	**-1.5**			
CREG1	cellular repressor of E1A-stimulated genes 1	**-2.3**	**1.8**	**-1.5**	**1.6**	
CREM	cAMP responsive element modulator	**-1.9**	**7.7**			
CRI1	EP300 interacting inhibitor of differentiation 1	**-2.5**	**1.9**			
CRIP1	cysteine-rich protein 1 (intestinal)	**9.6**	**-16.0**			
CRK	v-crk sarcoma virus CT10 oncogene homolog (avian)	**2.0**		**1.8**		
CRKL	v-crk sarcoma virus CT10 oncogene homolog (avian)-like	**-1.4**				
CROP	cisplatin resistance-associated overexpressed protein	**-1.7**	**1.9**	**-1.5**		
CRSP9	cofactor required for Sp1 transcriptional activation, subunit 9, 33kDa	**-1.2**		**2.2**		
CRTAP	cartilage associated protein	**-3.4**	**3.6**	**-2.2**		

CRYZ	crystallin, zeta (quinone reductase)	**1.3**	**-1.7**	**-1.4**		**-1.6**
CSDA	cold shock domain protein A	**6.4**		**4.4**		
CSE1L	CSE1 chromosome segregation 1-like (yeast)	**-2.5**	**-1.6**	**-1.4**		
CSK	c-src tyrosine kinase	**-1.7**	**-1.6**		**-1.8**	
CSNK1A1	casein kinase 1, alpha 1	**1.3**	**2.0**	**1.5**		
CSNK2A1	casein kinase 2, alpha 1 polypeptide	**-2.0**	**1.5**	**-1.4**		
CSPG5	chondroitin sulfate proteoglycan 5 (neuroglycan C)	**-1.7**				
CSPG6	structural maintenance of chromosomes 3	**-2.9**	**2.0**	**-1.7**		**-1.6**
CSRP1	cysteine and glycine-rich protein 1	**-1.3**				
CST7	cystatin F (leukocystatin)	**190.4**	**-26.4**	**2.4**		
CSTF1	cleavage stimulation factor, 3' pre-RNA, subunit 1, 50kDa	**-1.7**	**1.6**	**1.3**		
CSTF3	cleavage stimulation factor, 3' pre-RNA, subunit 3, 77kDa	**-4.0**	**2.2**			
CTBP1	C-terminal binding protein 1	**2.2**	**-1.5**	**1.3**	**1.3**	**1.3**
CTDSP2	CTD (carboxy-terminal domain, RNA polymerase II, polypeptide A) small phosphatase 2	**-1.2**	**1.9**	**-1.3**	**1.4**	
CTNNA1	catenin (cadherin-associated protein), alpha 1, 102kDa	**1.7**		**1.7**	**1.3**	
CTNNB1	catenin (cadherin-associated protein), beta 1, 88kDa	**2.5**	**1.4**	**1.4**		
CTPS	CTP synthase	**-2.4**	**-1.5**	**-1.4**		
CTR9	Ctr9, Paf1/RNA polymerase II complex component, homolog (S. cerevisiae)	**-1.9**	**1.4**			
CTSB	cathepsin B	**2.4**	**1.6**	**2.1**		
CTSC	--	**-7.0**	**2.7**	**-5.2**	**-1.5**	
CUGBP1	CUG triplet repeat, RNA binding protein 1	**-1.4**	**-1.8**	**-1.3**		
CUGBP2	CUG triplet repeat, RNA binding protein 2	**1.7**	**5.7**	**2.4**	**2.0**	**1.8**
CUL2	cullin 2	**-3.9**	**3.6**			
CUL4A	cullin 4A	**-1.5**	**1.5**	**1.4**		
CUL4B	cullin 4B	**2.8**				
CUTL1	cut-like 1, CCAAT displacement protein (Drosophila)	**1.9**	**-1.5**	**1.7**		
CXCR4	chemokine (C-X-C motif) receptor 4	**63.4**	**-2.5**	**1.8**	**1.3**	**1.5**
CYB5	cytochrome b5 type A (microsomal)	**-1.5**	**-1.3**	**2.3**		
CYC1	cytochrome c-1	**-4.4**	**1.5**	**-1.6**	**-1.3**	
CYCS	cytochrome c, somatic	**-13.5**	**4.9**	**-1.6**	**-1.6**	**-1.9**
CYFIP1	cytoplasmic FMR1 interacting protein 1	**2.9**	**-2.0**	**2.3**		
CYFIP2	cytoplasmic FMR1 interacting protein 2	**50.3**	**-44.2**	**-2.8**		**-1.3**
CYP51A1	cytochrome P450, family 51, subfamily A, polypeptide 1	**-2.1**	**1.3**			**-1.4**
DAAM1	dishevelled associated activator of morphogenesis 1	**8.5**	**-3.4**	**1.7**		
DAD1	defender against cell death 1	**2.7**	**-4.1**			
DAP3	death associated protein 3	**-1.7**	**1.9**		**-1.4**	**-1.6**
DAPK1	death-associated protein kinase 1	**-4.0**	**3.4**			
DAPK3	death-associated protein kinase 3	**-1.8**	**1.5**	**-1.3**	**1.2**	
DARS	aspartyl-tRNA synthetase	**-1.5**	**1.3**			
DATF1	death inducer-obliterator 1	**1.8**	**1.3**	**1.5**		
DAZAP2	DAZ associated protein 2	**1.7**	**-1.7**			
DBI	diazepam binding inhibitor (GABA receptor modulator, acyl-Coenzyme A binding protein)	**1.5**	**-1.9**			

DCK	deoxycytidine kinase	2.4	-1.9	1.3		
DCTD	dCMP deaminase	-1.5			-1.3	
DCTN1	dynactin 1 (p150, glued homolog, Drosophila)	1.2	1.3			
DCTN2	dynactin 2 (p50)	1.6	1.4	1.5		
DCTN3	dynactin 3 (p22)	2.1		1.3	1.3	
DCTN6	dynactin 6	-1.4		-1.4		
DDB1	damage-specific DNA binding protein 1, 127kDa	-1.6		-1.3		
DDB2	damage-specific DNA binding protein 2, 48kDa	2.1	-3.2			
DDIT4	DNA-damage-inducible transcript 4	22.4	5.5	4.6	4.4	
DDOST	dolichyl-diphosphooligosaccharide-protein glycosyltransferase	-1.9	1.8			
DDT	D-dopachrome tautomerase	1.6	-1.9	1.3		
DDX1	DEAD (Asp-Glu-Ala-Asp) box polypeptide 1	-1.8	1.2	-1.3		
DDX10	DEAD (Asp-Glu-Ala-Asp) box polypeptide 10	-2.8	1.2	-2.1		
DDX11	DEAD/H (Asp-Glu-Ala-Asp/His) box polypeptide 11 (CHL1-like helicase homolog, S. cerevisiae)	-2.2	-2.3	-1.5		
DDX17	DEAD (Asp-Glu-Ala-Asp) box polypeptide 17	-4.4	7.2			
DDX18	DEAD (Asp-Glu-Ala-Asp) box polypeptide 18	-2.6	-1.3	-1.7		
DDX21	DEAD (Asp-Glu-Ala-Asp) box polypeptide 21	-3.9	1.7	-2.1	-1.7	
DDX3X	DEAD (Asp-Glu-Ala-Asp) box polypeptide 3, X-linked	2.0	-1.8			-1.4
DDX42	DEAD (Asp-Glu-Ala-Asp) box polypeptide 42	-1.6	1.6			
DDX52	DEAD (Asp-Glu-Ala-Asp) box polypeptide 52	-2.0	1.7	-1.5		
DEAF1	deformed epidermal autoregulatory factor 1 (Drosophila)	-2.1		-2.0		
DECR1	2,4-dienoyl CoA reductase 1, mitochondrial	5.2	1.6			
DEDD	death effector domain containing	1.4		1.5		
DEK	DEK oncogene (DNA binding)	1.4	-1.9			
DEXI	dexamethasone-induced transcript	1.6	-1.5			
DFFA	DNA fragmentation factor, 45kDa, alpha polypeptide	-1.8	1.6	-2.0		
DGCR14	DiGeorge syndrome critical region gene 14	1.3				
DGKA	diacylglycerol kinase, alpha 80kDa	2.3			1.6	1.5
DGKD	diacylglycerol kinase, delta 130kDa	3.2	-2.5			
DGKG	diacylglycerol kinase, gamma 90kDa	-2.6	4.1			-4.6
DGKZ	diacylglycerol kinase, zeta 104kDa	5.4				
DGUOK	deoxyguanosine kinase	1.4			-1.3	-1.3
DHCR7	7-dehydrocholesterol reductase	-2.6		-1.7		
DHFR	dihydrofolate reductase	-2.5	-1.7	-1.9		
DHODH	dihydroorotate dehydrogenase	-1.4		-1.5		
DHRS1	dehydrogenase/reductase (SDR family) member 1	-2.0	2.3		1.7	
DHRS7	dehydrogenase/reductase (SDR family) member 7	-1.8	3.4			
DHX15	DEAH (Asp-Glu-Ala-His) box polypeptide 15	-1.4	-1.3	-1.6		
DHX30	DEAH (Asp-Glu-Ala-His) box polypeptide 30	1.8	-2.1	-1.7		
DHX9	DEAH (Asp-Glu-Ala-His) box polypeptide 9	2.3	-1.4			
DIA1	cytochrome b5 reductase 3	1.6		1.3		
DIAPH1	diaphanous homolog 1 (Drosophila)	-1.5	-1.7	-1.4		-1.4

DICER1	Dicer1, Dcr-1 homolog (Drosophila)	**1.8**	**-1.6**			
DIP	death-inducing-protein	**3.5**	**-2.3**	**-1.4**		**1.8**
DKC1	dyskeratosis congenita 1, dyskerin	**-3.3**		**-2.6**	**-1.7**	**-1.3**
DLAT	dihydrolipoamide S-acetyltransferase (E2 component of pyruvate dehydrogenase complex)	**-2.2**	**-1.6**	**-2.1**		
DLEU1	deleted in lymphocytic leukemia, 1	**-2.0**	**1.5**	**-1.8**		
DLG1	discs, large homolog 1 (Drosophila)	**1.8**	**-1.5**	**-1.9**		
DLG5	discs, large homolog 5 (Drosophila)	**3.1**	**2.2**	**2.0**		**1.3**
DLG7	discs, large homolog 7 (Drosophila)	**1.8**	**-1.3**			
DLST	dihydrolipoamide S-succinyltransferase (E2 component of 2-oxo-glutarate complex)	**1.5**				
DMTF1	cyclin D binding myb-like transcription factor 1	**1.8**				
DMWD	dystrophia myotonica-containing WD repeat motif	**-1.5**				
DMXL1	Dmx-like 1	**1.6**	**2.2**	**1.8**		
DMXL2	Dmx-like 2	**-1.2**	**1.7**		**1.6**	
DNAJA2	DnaJ (Hsp40) homolog, subfamily A, member 2	**-25.2**	**15.8**			
DNAJB1	DnaJ (Hsp40) homolog, subfamily B, member 1	**1.5**	**-1.9**			**-1.4**
DNAJB6	DnaJ (Hsp40) homolog, subfamily B, member 6	**2.0**	**-1.7**			
DNAJC11	DnaJ (Hsp40) homolog, subfamily C, member 11	**-1.8**	**1.4**	**1.9**	**-1.2**	
DNAJC7	DnaJ (Hsp40) homolog, subfamily C, member 7	**-1.6**	**1.3**	**-1.4**		
DNCH1	dynein, cytoplasmic 1, heavy chain 1	**1.8**		**4.0**		
DNCI2	dynein, cytoplasmic 1, intermediate chain 2	**-1.5**	**2.9**	**1.4**		
DNM1L	dynamin 1-like	**1.2**				
DNMT1	DNA (cytosine-5-)-methyltransferase 1	**-2.8**		**-1.4**		
DNPEP	aspartyl aminopeptidase	**1.6**	**-4.5**	**-1.6**	**-1.2**	
DOC-1R	CDK2-associated protein 2	**2.5**	**-2.0**	**2.0**		
DOCK2	dedicator of cytokinesis 2	**-2.8**	**1.8**			
DOK1	docking protein 1, 62kDa (downstream of tyrosine kinase 1)	**-2.3**	**2.3**			
DPAGT1	dolichyl-phosphate (UDP-N-acetylglucosamine) N-acetylglucosaminephosphotransferase 1 (GlcNAc-1-P transferase)	**-1.6**		**-1.8**		
DPEP1	dipeptidase 1 (renal)	**15.1**		**13.0**	**1.9**	**4.0**
DPH2L1	DPH1 homolog (S. cerevisiae)	**1.6**	**-1.7**			
DPM1	dolichyl-phosphate mannosyltransferase polypeptide 1, catalytic subunit	**-1.4**				
DPM2	dolichyl-phosphate mannosyltransferase polypeptide 2, regulatory subunit	**-1.5**				
DPYD	dihydropyrimidine dehydrogenase	**7.7**	**-8.8**			
DPYSL2	dihydropyrimidinase-like 2	**3.7**	**-4.2**	**3.1**		
DRG1	developmentally regulated GTP binding protein 1	**-1.6**		**-1.4**		**-1.5**
DSCR1	Down syndrome critical region gene 1	**7.9**	**1.5**	**7.7**	**5.0**	**4.8**
DST	dystonin	**15.8**	**-8.3**			
DTX4	deltex 4 homolog (Drosophila)	**3.3**	**-2.2**			
DTYMK	deoxythymidylate kinase (thymidylate kinase)	**1.2**	**-1.9**			
DULLARD	dullard homolog (Xenopus laevis)	**1.4**	**-1.4**	**1.8**		
DUSP10	dual specificity phosphatase 10	**1.9**	**-1.4**		**2.7**	
DUSP11	dual specificity phosphatase 11 (RNA/RNP complex 1-interacting)	**1.4**		**-1.3**		
DUSP14	dual specificity phosphatase 14	**-1.6**				

DUSP3	dual specificity phosphatase 3 (vaccinia virus phosphatase VH1-related)	-17.5	6.4			
DUSP6	dual specificity phosphatase 6	-28.2	15.8	1.7		
DUSP7	dual specificity phosphatase 7	-1.4		-1.2		-2.0
DUT	dUTP pyrophosphatase	-3.9		-2.0		
DVL3	dishevelled, dsh homolog 3 (Drosophila)	1.9	-1.6			
DXYS155E	chromosome X and Y open reading frame 3	1.8				
DYRK1A	dual-specificity tyrosine-(Y)-phosphorylation regulated kinase 1A	-1.2		-1.6		
DYRK2	dual-specificity tyrosine-(Y)-phosphorylation regulated kinase 2	-1.4				
DZIP3	zinc finger DAZ interacting protein 3	2.5	-1.3			
E2F1	E2F transcription factor 1	-2.7	-1.7			-1.2
E2F5	E2F transcription factor 5, p130-binding	-3.5			-1.6	
E2F6	E2F transcription factor 6	-2.4		-1.3		
EBAG9	estrogen receptor binding site associated, antigen, 9	2.5	-1.8			
EBNA1BP2	EBNA1 binding protein 2	-2.6		-1.8	-1.5	
ECH1	enoyl Coenzyme A hydratase 1, peroxisomal	-1.6				
ECHS1	enoyl Coenzyme A hydratase, short chain, 1, mitochondrial	-1.6		-1.4		
EDD1	E3 ubiquitin protein ligase, HECT domain containing, 1	1.9	-1.4	1.4	1.3	-1.4
EDEM1	ER degradation enhancer, mannosidase alpha-like 1	1.4	-1.5	-1.2		
EED	embryonic ectoderm development	1.2	-1.9			
EEF1A1	eukaryotic translation elongation factor 1 alpha 1	-5.1	4.9			
EEF1D	eukaryotic translation elongation factor 1 delta (guanine nucleotide exchange protein)	-1.3	1.6			
EEF1E1	eukaryotic translation elongation factor 1 epsilon 1	-2.4	-1.6	-3.3	-1.7	
EFNB2	ephrin-B2	5.8	-5.9	-2.8		
EHBP1	EH domain binding protein 1	-2.6	1.4			
EHD1	EH-domain containing 1	-1.4	1.6			
EI24	etoposide induced 2.4 mRNA	-2.1	1.3	-1.5		
EIF1	eukaryotic translation initiation factor 1	1.3			-1.4	-1.7
EIF1AX	eukaryotic translation initiation factor 1A, X-linked	-2.8	-2.2	-1.5		
EIF2A	eukaryotic translation initiation factor 2A, 65kDa	1.2	1.3			
EIF2B2	eukaryotic translation initiation factor 2B, subunit 2 beta, 39kDa	-1.2				
EIF2B4	eukaryotic translation initiation factor 2B, subunit 4 delta, 67kDa	-1.4				
EIF2B5	eukaryotic translation initiation factor 2B, subunit 5 epsilon, 82kDa	-1.5				
EIF2C2	eukaryotic translation initiation factor 2C, 2	-4.3	2.6	-1.6		
EIF2S1	eukaryotic translation initiation factor 2, subunit 1 alpha, 35kDa	-1.8	-1.2	-1.6	-1.4	-1.4
EIF2S2	eukaryotic translation initiation factor 2, subunit 2 beta, 38kDa	-1.6				
EIF3S10	eukaryotic translation initiation factor 3, subunit 10 theta, 150/170kDa	-5.4	3.9	-1.3		-1.7
EIF3S2	eukaryotic translation initiation factor 3, subunit 2 beta, 36kDa	-1.6		-1.6		-1.5
EIF3S3	eukaryotic translation initiation factor 3, subunit 3 gamma, 40kDa	1.4				
EIF3S5	eukaryotic translation initiation factor 3, subunit 5 epsilon, 47kDa	1.3		1.2		
EIF3S7	eukaryotic translation initiation factor 3, subunit 7 zeta, 66/67kDa	-1.3	1.3			
EIF3S8	eukaryotic translation initiation factor 3, subunit 8, 110kDa	3.4	-3.1	-1.4	-1.2	
EIF3S9	eukaryotic translation initiation factor 3, subunit 9 eta, 116kDa	-2.2	-1.2	-2.0	-1.5	

EIF4B	eukaryotic translation initiation factor 4B	-1.8	1.8	-2.4		
EIF4E	eukaryotic translation initiation factor 4E	-1.4	-1.3			
EIF4E2	eukaryotic translation initiation factor 4E family member 2	1.5	-1.6	1.2		
EIF4EBP1	eukaryotic translation initiation factor 4E binding protein 1	-5.7		-3.1		
EIF4EBP2	eukaryotic translation initiation factor 4E binding protein 2	-1.3		-1.3		
EIF4G1	eukaryotic translation initiation factor 4 gamma, 1	-1.4				-1.4
EIF4G3	eukaryotic translation initiation factor 4 gamma, 3	-1.6		-1.2		
EIF5	eukaryotic translation initiation factor 5	-1.8	1.2			
EIF5A	eukaryotic translation initiation factor 5A	3.5	-10.2	-2.0		
EIF5B	eukaryotic translation initiation factor 5B	-4.3	2.5	-1.6		
ELF1	E74-like factor 1 (ets domain transcription factor)	2.7	-1.8	1.4		
ELF2	E74-like factor 2 (ets domain transcription factor)	1.4	-1.8			
ELK1	ELK1, member of ETS oncogene family	1.4				
ELK3	ELK3, ETS-domain protein (SRF accessory protein 2)	-2.4	5.2			
ELOVL5	ELOVL family member 5, elongation of long chain fatty acids (FEN1/Elo2, SUR4/Elo3-like, yeast)	1.8	-1.6	1.3		
ENO1	enolase 1, (alpha)	-2.6		-1.5	-1.3	
ENTH	clathrin interactor 1	-1.5	2.5	1.9		
ENTPD6	ectonucleoside triphosphate diphosphohydrolase 6 (putative function)	-2.0				
EP300	E1A binding protein p300	1.4				
EPB41	erythrocyte membrane protein band 4.1 (elliptocytosis 1, RH-linked)	-1.7				
EPB41L2	erythrocyte membrane protein band 4.1-like 2	-1.9	1.8			
EPOR	erythropoietin receptor	-6.1	16.6			
EPRS	glutamyl-prolyl-tRNA synthetase	-2.4	1.3	-1.7		
EPS15	epidermal growth factor receptor pathway substrate 15	1.5	1.6	1.8	1.3	
ERCC5	excision repair cross-complementing rodent repair deficiency, complementation group 5 (xeroderma pigmentosum, complementation group G (Cockayne syndrome))	1.8	-1.4			
ERCC8	excision repair cross-complementing rodent repair deficiency, complementation group 8	-1.4		-1.8		
ERF	Ets2 repressor factor	-2.5	2.1	-1.5		
ERP29	endoplasmic reticulum protein 29	-1.8	1.4	-1.3		
ESD	esterase D/formylglutathione hydrolase	-1.9	2.3	1.7		
ETF1	eukaryotic translation termination factor 1	-2.4	1.3	-1.6		
ETFB	electron-transfer-flavoprotein, beta polypeptide	-1.7		2.1		
ETHE1	ethylmalonic encephalopathy 1	-1.6	1.6	-1.3		
ETS1	v-ets erythroblastosis virus E26 oncogene homolog 1 (avian)	26.5	-29.1			
ETS2	v-ets erythroblastosis virus E26 oncogene homolog 2 (avian)	4.1	-1.5	5.1	-1.3	-1.7
ETV6	ets variant gene 6 (TEL oncogene)	-3.1	2.5	-1.6		
EVER1	transmembrane channel-like 6	-2.4		1.6		
EWSR1	Ewing sarcoma breakpoint region 1	-1.3				
EXO1	exonuclease 1	-4.6	-1.6	-2.1		
EXOSC2	exosome component 2	-2.6		-2.4	-1.7	
EXOSC7	exosome component 7	-2.1			-1.8	
EXT1	exostoses (multiple) 1	-2.5		-1.8		
EXTL2	exostoses (multiple)-like 2	-1.9	1.5	-2.0		

EZH1	enhancer of zeste homolog 1 (Drosophila)	**2.0**	**1.3**	**-1.4**		
EZH2	enhancer of zeste homolog 2 (Drosophila)	**-1.3**	**-1.6**			
F8A1	coagulation factor VIII-associated (intronic transcript) 1	**-2.1**	**1.6**		**1.2**	
FABP5	fatty acid binding protein 5 (psoriasis-associated)	**-2.5**	**-2.2**	**-4.4**		
FADD	Fas (TNFRSF6)-associated via death domain	**-1.3**				**-1.9**
FADS1	fatty acid desaturase 1	**-1.8**	**-1.5**	**-1.5**		**-1.9**
FADS3	fatty acid desaturase 3	**5.6**	**-3.1**		**-1.2**	
FAH	fumarylacetoacetate hydrolase (fumarylacetoacetase)	**-2.8**	**1.6**		**-1.5**	
FALZ	bromodomain PHD finger transcription factor	**-1.3**	**1.6**	**-1.5**		
FAM38A	family with sequence similarity 38, member A	**1.8**	**2.6**	**2.6**		
FAM89B	family with sequence similarity 89, member B	**5.2**	**-2.5**			
FANCG	Fanconi anemia, complementation group G	**-2.0**		**-1.3**		
FANCL	Fanconi anemia, complementation group L	**-2.8**	**1.6**			
FARSLA	phenylalanine-tRNA synthetase-like, alpha subunit	**-4.1**	**1.6**	**-2.1**	**-1.9**	**-2.0**
FASN	fatty acid synthase	**-2.2**		**-3.2**	**-1.6**	
FBL	fibrillarin	**-2.1**	**1.4**	**-1.2**	**-1.4**	**-1.4**
FBXO7	F-box protein 7	**1.3**				
FBXO9	F-box protein 9	**-1.9**	**2.2**	**-1.4**		
FCHSD2	FCH and double SH3 domains 2	**4.0**	**1.9**	**3.0**		
FDFT1	farnesyl-diphosphate farnesyltransferase 1	**-1.2**	**1.4**			
FDPS	farnesyl diphosphate synthase (farnesyl pyrophosphate synthetase, dimethylallyltranstransferase, geranyltranstransferase)	**1.6**	**-2.3**			
FECH	ferrochelatase (protoporphyria)	**-1.8**				
FEN1	flap structure-specific endonuclease 1	**-2.9**	**-1.3**	**-1.8**		
FEZ2	fasciculation and elongation protein zeta 2 (zygin II)	**1.6**	**1.5**			
FGF9	fibroblast growth factor 9 (glia-activating factor)	**39.2**	**-45.7**	**-2.1**		
FGFR1	fibroblast growth factor receptor 1 (fms-related tyrosine kinase 2, Pfeiffer syndrome)	**-1.6**	**7.4**		**1.6**	
FGFR1OP	FGFR1 oncogene partner	**-2.7**	**1.5**			
FH	fumarate hydratase	**-2.6**	**-1.4**	**-2.1**		
FHL1	four and a half LIM domains 1	**9.6**	**-2.1**	**1.8**	**3.3**	**13.7**
FKBP1A	FK506 binding protein 1A, 12kDa	**-1.7**	**1.5**	**-1.3**		**1.3**
FKBP4	FK506 binding protein 4, 59kDa	**-8.6**	**3.0**			
FKBP5	FK506 binding protein 5	**13.7**	**7.0**	**21.4**	**6.6**	**4.4**
FLI1	Friend leukemia virus integration 1	**1.9**		**1.6**		
FLII	flightless I homolog (Drosophila)	**1.8**	**-1.4**			
FLJ30092	AF-1 specific protein phosphatase	**2.8**		**1.6**		
FLN29	TRAF-type zinc finger domain containing 1	**1.4**				
FLNA	filamin A, alpha (actin binding protein 280)	**3.1**	**-2.2**			
FMR1	fragile X mental retardation 1	**2.8**	**-2.3**	**1.4**		
FNBP1	formin binding protein 1	**13.3**	**-17.0**	**-2.9**		
FNBP1L	formin binding protein 1-like	**3.5**	**1.4**	**3.0**	**1.9**	
FNBP3	PRP40 pre-mRNA processing factor 40 homolog A (yeast)	**-2.4**	**2.2**	**-2.1**		
FNTA	farnesyltransferase, CAAX box, alpha	**-1.2**	**1.4**			

FOSL2	FOS-like antigen 2	**-1.9**	**7.1**	**4.0**		**1.4**
FOXJ2	forkhead box J2	**2.0**	**-2.0**			
FOXK2	forkhead box K2	**-1.8**	**1.8**			**-1.4**
FOXM1	forkhead box M1	**2.2**	**-3.5**	**1.4**		
FOXO3A	forkhead box O3A	**2.3**	**1.7**	**2.7**		
FRG1	FSHD region gene 1	**-1.4**				
FSCN1	fascin homolog 1, actin-bundling protein (Strongylocentrotus purpuratus)	**-3.9**	**1.7**	**1.6**	**-1.5**	
FTH1	ferritin, heavy polypeptide 1	**2.9**	**-2.0**	**1.5**		
FTL	ferritin, light polypeptide	**-2.3**	**1.9**	**-1.4**		
FTSJ1	FtsJ homolog 1 (E. coli)	**-2.4**	**1.2**	**-1.3**		
FUBP1	far upstream element (FUSE) binding protein 1	**-1.9**	**1.7**	**-1.8**		
FUS	fusion (involved in t(12;16) in malignant liposarcoma)	**1.5**	**-2.6**	**-1.5**		
FUT7	fucosyltransferase 7 (alpha (1,3) fucosyltransferase)	**54.2**		**-1.8**		
FXR1	fragile X mental retardation, autosomal homolog 1	**1.4**	**1.4**	**1.4**		
FXR2	fragile X mental retardation, autosomal homolog 2	**1.6**		**1.4**		
FYB	FYN binding protein (FYB-120/130)	**-7.9**	**11.8**			
FYN	FYN oncogene related to SRC, FGR, YES	**2.0**	**-8.6**		**1.2**	
FZD6	frizzled homolog 6 (Drosophila)	**-2.9**	**10.1**	**2.1**	**1.5**	
FZR1	fizzy/cell division cycle 20 related 1 (Drosophila)	**2.3**	**-1.9**			
G1P2	ISG15 ubiquitin-like modifier	**2.0**	**-3.1**			
G22P1	X-ray repair complementing defective repair in Chinese hamster cells 6 (Ku autoantigen, 70kDa)	**-1.6**		**-1.4**		
G3BP	GTPase activating protein (SH3 domain) binding protein 1	**-4.3**	**1.4**	**-2.2**		
G3BP2	GTPase activating protein (SH3 domain) binding protein 2	**-1.7**	**1.3**			
GABARAP	GABA(A) receptor-associated protein	**2.1**	**-1.3**			
GABARAPL2	GABA(A) receptor-associated protein-like 2	**1.7**				
GABPB2	GA binding protein transcription factor, beta subunit 2	**1.8**	**-1.7**	**1.5**		
GADD45B	growth arrest and DNA-damage-inducible, beta	**1.4**	**1.3**	**1.5**		
GALC	galactosylceramidase	**1.5**	**-2.1**			
GALK2	galactokinase 2	**-1.7**	**1.3**			
GALNT1	UDP-N-acetyl-alpha-D-galactosamine:polypeptide N-acetylgalactosaminyltransferase 1 (GalNAc-T1)	**-2.2**	**2.0**			
GALNT10	UDP-N-acetyl-alpha-D-galactosamine:polypeptide N-acetylgalactosaminyltransferase 10 (GalNAc-T10)	**2.2**				
GALNT2	UDP-N-acetyl-alpha-D-galactosamine:polypeptide N-acetylgalactosaminyltransferase 2 (GalNAc-T2)	**1.9**	**-2.2**	**1.4**		
GAMT	guanidinoacetate N-methyltransferase	**-2.9**		**-2.2**		
GAPD	glyceraldehyde-3-phosphate dehydrogenase	**1.3**	**-1.2**			
GAPDHS	glyceraldehyde-3-phosphate dehydrogenase, spermatogenic	**-2.4**	**1.8**			
GARNL1	GTPase activating Rap/RanGAP domain-like 1	**1.7**		**1.6**		
GARS	glycyl-tRNA synthetase	**-4.3**	**1.9**	**-2.1**	**-1.4**	**-1.7**
GART	phosphoribosylglycinamide formyltransferase, phosphoribosylglycinamide synthetase, phosphoribosylaminoimidazole synthetase	**-4.2**	**1.4**	**-2.4**	**-1.6**	
GAS2L1	growth arrest-specific 2 like 1	**17.6**		**15.6**		
GAS7	growth arrest-specific 7	**7.5**	**-10.7**	**1.5**		
GATA3	GATA binding protein 3	**3.3**	**-3.1**			
GBAS	glioblastoma amplified sequence	**-3.2**	**4.0**			

GBE1	glucan (1,4-alpha-), branching enzyme 1 (glycogen branching enzyme, Andersen disease, glycogen storage disease type IV)	**1.4**		**1.3**		
GBF1	golgi-specific brefeldin A resistance factor 1	**1.6**				
GCAT	glycine C-acetyltransferase (2-amino-3-ketobutyrate coenzyme A ligase)	**-10.8**	**9.1**	**-1.4**		
GCH1	GTP cyclohydrolase 1 (dopa-responsive dystonia)	**1.5**	**-1.7**	**-1.7**		
GCHFR	GTP cyclohydrolase I feedback regulator	**10.3**		**-1.8**		
GCLC	glutamate-cysteine ligase, catalytic subunit	**-1.3**	**2.0**	**1.7**		
GCLM	glutamate-cysteine ligase, modifier subunit	**3.6**	**-2.5**	**2.4**		
GCN1L1	GCN1 general control of amino-acid synthesis 1-like 1 (yeast)	**-1.5**	**1.3**	**-1.4**		
GCS1	glucosidase I	**-1.7**		**-1.3**		
GCSH	glycine cleavage system protein H (aminomethyl carrier)	**-4.6**	**2.1**	**-3.0**	**-1.6**	
GDI1	GDP dissociation inhibitor 1	**2.4**	**-1.6**	**1.4**		
GEMIN4	gem (nuclear organelle) associated protein 4	**-3.0**	**-1.6**			**-1.5**
GFI1	growth factor independent 1	**-5.9**	**65.2**			
GGH	gamma-glutamyl hydrolase (conjugase, folylpolygammaglutamyl hydrolase)	**1.3**	**-2.7**	**1.2**		
GGPS1	geranylgeranyl diphosphate synthase 1	**-1.2**	**-1.5**	**1.2**		
GIT2	G protein-coupled receptor kinase interactor 2	**-2.8**	**12.8**			
GJA7	gap junction protein, alpha 7, 45kDa (connexin 45)	**43.1**	**-13.5**	**1.6**		
GLA	galactosidase, alpha	**-3.6**	**2.3**			
GLB1	galactosidase, beta 1	**-1.9**	**2.3**		**1.4**	**1.9**
GLE1L	GLE1 RNA export mediator-like (yeast)	**-1.4**		**-1.2**		
GLG1	golgi apparatus protein 1	**1.9**				
GLMN	glomulin, FKBP associated protein	**-2.2**	**3.5**			
GLO1	glyoxalase I	**-2.0**	**1.3**	**-1.5**		
GLRX	glutaredoxin (thioltransferase)	**4.4**	**-4.0**	**1.6**	**3.4**	
GLS	glutaminase	**-2.2**	**2.1**	**-2.1**		**1.7**
GLUD1	glutamate dehydrogenase 1	**-1.5**		**-1.3**		**-1.3**
GLUD2	glutamate dehydrogenase 2	**-1.5**				
GLUL	glutamate-ammonia ligase (glutamine synthetase)	**1.8**	**2.3**	**2.5**	**1.9**	
GM2A	GM2 ganglioside activator	**1.6**			**1.3**	
GMFB	glia maturation factor, beta	**1.6**				
GMPS	guanine monphosphate synthetase	**-2.1**	**-1.4**	**-1.4**		
GNA11	guanine nucleotide binding protein (G protein), alpha 11 (Gq class)	**-4.6**	**1.6**			
GNA13	guanine nucleotide binding protein (G protein), alpha 13	**-1.5**	**1.6**			
GNAI3	guanine nucleotide binding protein (G protein), alpha inhibiting activity polypeptide 3	**1.4**		**2.0**		
GNAQ	guanine nucleotide binding protein (G protein), q polypeptide	**-1.6**	**5.0**	**1.3**		
GNAS	GNAS complex locus	**2.4**	**-1.7**	**1.7**		
GNB1	guanine nucleotide binding protein (G protein), beta polypeptide 1	**1.7**	**-1.4**			
GNB2	guanine nucleotide binding protein (G protein), beta polypeptide 2	**1.7**	**-1.7**	**1.4**		
GNE	glucosamine (UDP-N-acetyl)-2-epimerase/N-acetylmannosamine kinase	**2.5**	**-3.9**			**-1.4**
GNL2	guanine nucleotide binding protein-like 2 (nucleolar)	**-2.0**		**-1.4**		
GNPAT	glyceronephosphate O-acyltransferase	**1.4**			**1.4**	
GNPDA1	glucosamine-6-phosphate deaminase 1	**-59.9**	**11.5**			

GNS	glucosamine (N-acetyl)-6-sulfatase (Sanfilippo disease IIID)	**-2.0**	**2.8**			
GOLGA1	golgi autoantigen, golgin subfamily a, 1	**1.5**				
GOLGA4	golgi autoantigen, golgin subfamily a, 4	**-1.5**				
GORASP2	golgi reassembly stacking protein 2, 55kDa	**-2.0**	**1.8**	**-1.3**		
GOSR1	golgi SNAP receptor complex member 1	**-1.7**	**1.5**			
GOSR2	golgi SNAP receptor complex member 2	**-1.5**				
GOT1	glutamic-oxaloacetic transaminase 1, soluble (aspartate aminotransferase 1)	**-1.4**				
GOT2	glutamic-oxaloacetic transaminase 2, mitochondrial (aspartate aminotransferase 2)	**-1.5**			**-1.3**	
GPAA1	glycosylphosphatidylinositol anchor attachment protein 1 homolog (yeast)	**-1.4**				
GPATCH8	G patch domain containing 8	**3.5**		**1.7**		
GPI	glucose phosphate isomerase	**-2.0**		**-3.3**		
GPR125	G protein-coupled receptor 125	**-4.2**			**-1.8**	
GPS2	G protein pathway suppressor 2	**3.8**	**-4.7**			
GPSM2	G-protein signalling modulator 2 (AGS3-like, C. elegans)	**2.7**	**-1.8**	**1.6**	**1.7**	
GPSM3	G-protein signalling modulator 3 (AGS3-like, C. elegans)	**3.0**	**-1.9**			
GPSN2	glycoprotein, synaptic 2	**-1.5**	**-1.3**			
GPX4	glutathione peroxidase 4 (phospholipid hydroperoxidase)	**-2.2**	**1.8**			
GPX7	glutathione peroxidase 7	**-1.5**	**1.9**			
GRB10	growth factor receptor-bound protein 10	**11.1**	**-12.4**	**-1.4**		
GRB2	growth factor receptor-bound protein 2	**-3.3**	**1.9**	**-1.8**		
GREB1	GREB1 protein	**-11.0**	**8.1**			**2.6**
GRHPR	glyoxylate reductase/hydroxypyruvate reductase	**-1.3**	**1.4**	**-1.6**		
GRIK5	glutamate receptor, ionotropic, kainate 5	**-14.5**	**4.2**			
GRK5	G protein-coupled receptor kinase 5	**-3.8**	**14.8**	**2.8**		
GRK6	G protein-coupled receptor kinase 6	**2.2**	**-3.3**			
GRLF1	glucocorticoid receptor DNA binding factor 1	**-1.2**	**1.4**			
GRSF1	G-rich RNA sequence binding factor 1	**-2.7**				
GSK3B	glycogen synthase kinase 3 beta	**-2.3**	**2.1**	**1.3**		
GSPT1	G1 to S phase transition 1	**-1.4**	**-1.6**	**-1.2**	**-1.5**	**-1.8**
GSR	glutathione reductase	**1.2**	**-1.6**			
GSS	glutathione synthetase	**-2.2**		**-1.3**		
GSTO1	glutathione S-transferase omega 1	**-3.0**	**1.8**	**-1.3**		
GSTP1	glutathione S-transferase pi	**-1.7**	**-1.2**			
GTF2A2	general transcription factor IIA, 2, 12kDa	**1.6**	**-2.8**	**-1.2**		**1.5**
GTF2E1	general transcription factor IIE, polypeptide 1, alpha 56kDa	**-1.5**				
GTF2H1	general transcription factor IIH, polypeptide 1, 62kDa	**-2.0**	**1.3**	**-1.3**		
GTF2H2	general transcription factor IIH, polypeptide 2, 44kDa	**-3.2**	**2.1**			
GTF2H5	general transcription factor IIH, polypeptide 5	**1.4**				
GTF2I	general transcription factor II, i	**2.3**	**-2.1**			
GTF3A	general transcription factor IIIA	**-2.0**	**-1.7**	**-1.4**		
GTF3C1	general transcription factor IIIC, polypeptide 1, alpha 220kDa	**1.7**	**-1.3**			
GTF3C2	general transcription factor IIIC, polypeptide 2, beta 110kDa	**-1.2**		**-2.0**		

GTPBP6	GTP binding protein 6 (putative)	**1.8**	**-2.2**			
GTSE1	G-2 and S-phase expressed 1	**2.0**	**-1.7**	**1.6**		
GYG1	glycogenin 1	**2.7**		**3.8**		
GYPC	glycophorin C (Gerbich blood group)	**-1.8**	**1.8**			**2.8**
H1F0	H1 histone family, member 0	**-5.6**	**8.1**	**2.4**		**2.5**
H1FX	H1 histone family, member X	**3.7**	**-3.2**		**1.5**	**1.3**
H2AFX	H2A histone family, member X	**1.3**	**-2.6**			
H2AFY	H2A histone family, member Y	**-2.8**	**-1.9**	**-1.6**		
H2AFZ	H2A histone family, member Z	**-1.9**				
H3F3A	H3 histone, family 3A	**1.3**	**-1.2**			
H3F3B	H3 histone, family 3B (H3.3B)	**-2.2**	**4.1**	**1.3**		
H41	CDV3 homolog (mouse)	**-1.4**	**1.3**	**-1.2**		
HADHB	hydroxyacyl-Coenzyme A dehydrogenase/3-ketoacyl-Coenzyme A thiolase/enoyl-Coenzyme A hydratase (trifunctional protein), beta subunit	**-1.4**	**1.8**			
HAGH	hydroxyacylglutathione hydrolase	**2.2**				
HAN11	WD repeat domain 68	**-1.7**	**1.8**	**-1.2**		
HAT1	histone acetyltransferase 1	**-4.0**	**1.6**			
HAX1	HCLS1 associated protein X-1	**-1.3**	**-1.3**	**-1.7**		**-1.3**
HBP1	HMG-box transcription factor 1	**1.5**	**1.6**	**1.4**		**1.6**
HCAP-D3	non-SMC condensin II complex, subunit D3	**-2.9**		**-2.1**		
HCCS	holocytochrome c synthase (cytochrome c heme-lyase)	**-2.0**		**-1.2**		
HCFC1	host cell factor C1 (VP16-accessory protein)	**1.2**	**-1.5**			
HCLS1	hematopoietic cell-specific Lyn substrate 1	**1.8**	**-1.6**	**-1.4**		
HDAC1	histone deacetylase 1	**-1.5**			**-1.4**	
HDAC2	histone deacetylase 2	**-1.6**		**-1.5**		**-1.5**
HDAC4	histone deacetylase 4	**1.3**	**2.6**	**-1.8**		
HDAC6	histone deacetylase 6	**1.4**				
HDGF	hepatoma-derived growth factor (high-mobility group protein 1-like)	**-1.3**		**-1.5**		
HEAB	CLP1, cleavage and polyadenylation factor I subunit, homolog (S. cerevisiae)	**-1.2**				
HELZ	helicase with zinc finger	**-1.3**	**1.5**			
HERPUD1	homocysteine-inducible, endoplasmic reticulum stress-inducible, ubiquitin-like domain member 1	**2.1**	**-2.1**			
HEXIM1	hexamethylene bis-acetamide inducible 1	**-2.2**	**4.5**	**1.8**		
HGS	hepatocyte growth factor-regulated tyrosine kinase substrate	**-1.7**	**1.5**			
HHEX	homeobox, hematopoietically expressed	**-5.4**	**3.4**	**-2.0**		
HIP1R	huntingtin interacting protein 1 related	**1.6**	**-1.5**	**2.3**		
HIP2	huntingtin interacting protein 2	**-2.2**	**-1.6**	**-1.2**		
HIPK1	homeodomain interacting protein kinase 1	**1.3**		**-1.5**	**1.6**	
HIPK3	homeodomain interacting protein kinase 3	**1.5**	**1.3**	**1.5**		
HISPPD2A	histidine acid phosphatase domain containing 2A	**2.5**	**-3.1**			
HIST2H2AA	histone cluster 2, H2aa3	**2.9**	**-2.0**			
HIVEP1	human immunodeficiency virus type I enhancer binding protein 1	**-1.5**	**1.4**	**2.4**		
HIVEP2	human immunodeficiency virus type I enhancer binding protein 2	**10.1**	**-6.5**			
HK2	hexokinase 2	**-2.3**	**1.4**	**-3.1**	**-2.0**	

HLA-A	major histocompatibility complex, class I, A	2.7	-2.5		1.5	
HLA-B	major histocompatibility complex, class I, B	3.7	-3.4	1.2		
HLA-F	major histocompatibility complex, class I, F	4.7	-3.0	2.2		
HLA-G	HLA-G histocompatibility antigen, class I, G	1.9	-2.0	1.5	1.5	1.3
HMBS	hydroxymethylbilane synthase	-1.9	-1.6			
HMG20B	high-mobility group 20B	1.5				
HMGA1	high mobility group AT-hook 1	-1.6		-1.6		
HMGB1	high-mobility group box 1	1.9	-3.0			
HMGB2	high-mobility group box 2	1.5		1.3		
HMGB3	high-mobility group box 3	-1.5		1.4		
HMGCR	3-hydroxy-3-methylglutaryl-Coenzyme A reductase	2.0	-1.9		-1.4	
HMGCS1	3-hydroxy-3-methylglutaryl-Coenzyme A synthase 1 (soluble)	2.4	-3.5		-2.3	-1.5
HMGN1	high-mobility group nucleosome binding domain 1	-1.6				
HMGN3	high mobility group nucleosomal binding domain 3	1.5				
HMMR	hyaluronan-mediated motility receptor (RHAMM)	3.3	-1.5	1.8		
HMOX2	heme oxygenase (decycling) 2	1.6				-1.2
HNRPA0	heterogeneous nuclear ribonucleoprotein A0	-1.5	1.5			
HNRPA1	heterogeneous nuclear ribonucleoprotein A1	-1.8	1.9	1.4		
HNRPA2B1	heterogeneous nuclear ribonucleoprotein A2/B1	-2.4	5.0			
HNRPA3	heterogeneous nuclear ribonucleoprotein A3	-2.4	1.8	-1.4		
HNRPAB	heterogeneous nuclear ribonucleoprotein A/B	-1.8	-1.4	-1.6	-1.5	-1.4
HNRPC	heterogeneous nuclear ribonucleoprotein C (C1/C2)	-1.9	-1.2	-1.5		
HNRPD	heterogeneous nuclear ribonucleoprotein D (AU-rich element RNA binding protein 1, 37kDa)	-1.8	3.1			
HNRPDL	heterogeneous nuclear ribonucleoprotein D-like	-2.4	1.7	-1.6		
HNRPF	heterogeneous nuclear ribonucleoprotein F	-2.5	1.3			
HNRPH1	heterogeneous nuclear ribonucleoprotein H1 (H)	2.1	-2.9	1.3		
HNRPH3	heterogeneous nuclear ribonucleoprotein H3 (2H9)	-1.3	-1.4			
HNRPL	heterogeneous nuclear ribonucleoprotein L	1.3	-1.4	-2.0		
HNRPM	heterogeneous nuclear ribonucleoprotein M	-2.2	1.8	1.8		
HNRPR	heterogeneous nuclear ribonucleoprotein R	-2.0				
HNRPU	heterogeneous nuclear ribonucleoprotein U (scaffold attachment factor A)	-2.9	-1.4	-1.4		
HNRPUL1	heterogeneous nuclear ribonucleoprotein U-like 1	3.4	-1.4			
HOM-TES-103	hypothetical protein LOC25900	1.4		2.3		
HOXA9	homeobox A9	194.0	-91.3			
HPCAL1	hippocalcin-like 1	1.3			1.3	
HRB	HIV-1 Rev binding protein	3.8	-1.7	1.5		
HRB2	KRR1, small subunit (SSU) processome component, homolog (yeast)	-1.2		-1.3		
HRMT1L2	protein arginine methyltransferase 1	-2.6	-1.2	-2.6		-1.7
HRSP12	heat-responsive protein 12	-2.7		-2.0		
HS2ST1	heparan sulfate 2-O-sulfotransferase 1	-1.4	1.8			
HS6ST1	heparan sulfate 6-O-sulfotransferase 1	1.5	1.3	1.8		2.2
HSD17B4	hydroxysteroid (17-beta) dehydrogenase 4	2.0	2.0	1.9		

HSF1	heat shock transcription factor 1	-1.5				
HSPA4	heat shock 70kDa protein 4	-1.8		-1.4		-1.6
HSPA4L	heat shock 70kDa protein 4-like	-42.0	25.8			
HSPA5	heat shock 70kDa protein 5 (glucose-regulated protein, 78kDa)	-3.2	1.6			-1.5
HSPA9B	heat shock 70kDa protein 9 (mortalin)	-3.8		-2.8		-1.6
HSPB1	heat shock 27kDa protein 1	1.6	-2.7	-1.3		
HSPBP1	hsp70-interacting protein	-1.5			-1.4	
HSPCB	heat shock protein 90kDa alpha (cytosolic), class B member 1	-1.8	1.4	-2.0	-1.4	-1.3
HSPD1	heat shock 60kDa protein 1 (chaperonin)	-2.3		-3.0	-1.4	
HSPE1	heat shock 10kDa protein 1 (chaperonin 10)	-3.7		-2.2	-1.8	-2.1
HTATSF1	HIV-1 Tat specific factor 1	-2.0	1.4			
HUWE1	HECT, UBA and WWE domain containing 1	-1.3	1.4			
HYAL2	hyaluronoglucosaminidase 2	-6.2				
HYOU1	hypoxia up-regulated 1	-2.3	1.8	-1.7		
IARS	isoleucine-tRNA synthetase	-3.8	-1.4	-2.2	-1.6	
IARS2	isoleucine-tRNA synthetase 2, mitochondrial	-1.4	1.4	-1.2		
IBRDC3	IBR domain containing 3	2.3	-2.0			
IBTK	inhibitor of Bruton agammaglobulinemia tyrosine kinase	-1.4	1.5	1.6		
ICAM2	intercellular adhesion molecule 2	3.3	-17.5	-2.4	-1.8	-1.7
ICAM3	intercellular adhesion molecule 3	1.3				
ID1	inhibitor of DNA binding 1, dominant negative helix-loop-helix protein	-17.8				
ID2	inhibitor of DNA binding 2, dominant negative helix-loop-helix protein	3.3	5.8	3.0	1.8	
IDE	insulin-degrading enzyme	-1.7	1.4			
IDH1	isocitrate dehydrogenase 1 (NADP+), soluble	1.8	-1.3	-1.6		
IDH2	isocitrate dehydrogenase 2 (NADP+), mitochondrial	-2.3	-1.4	-1.4		
IDH3A	isocitrate dehydrogenase 3 (NAD+) alpha	-2.4	-1.6	-1.7	-1.5	-1.5
IDH3B	isocitrate dehydrogenase 3 (NAD+) beta	-2.1	1.5			
IDH3G	isocitrate dehydrogenase 3 (NAD+) gamma	-1.3				
IDI1	isopentenyl-diphosphate delta isomerase 1	2.1	1.5	1.7		
IDS	iduronate 2-sulfatase (Hunter syndrome)	1.5	-1.6			
IER2	immediate early response 2	-2.7	1.6			-1.4
IFI16	interferon, gamma-inducible protein 16	-1.3	2.0	2.4	-1.3	
IFI44	interferon-induced protein 44	1.9				
IFITM1	interferon induced transmembrane protein 1 (9-27)	-14.3	4.6	3.2		
IFITM2	interferon induced transmembrane protein 2 (1-8D)	1.6	-3.1	2.2		
IFNGR1	interferon gamma receptor 1	2.6	5.7	3.3	1.7	
IFRD1	interferon-related developmental regulator 1	-1.4			-1.8	-1.3
IFRD2	interferon-related developmental regulator 2	-3.7		-3.4	-1.8	-1.7
IGBP1	immunoglobulin (CD79A) binding protein 1	1.4				
IGF2R	insulin-like growth factor 2 receptor	1.7	1.3	1.7		
IGFBP2	insulin-like growth factor binding protein 2, 36kDa	-5.1				
IGHG1	immunoglobulin heavy constant gamma 1 (G1m marker)	3.7	-2.6	1.3		

IGLL1	immunoglobulin lambda-like polypeptide 1	**9.6**	**-44.6**	**-1.2**	**-1.4**	
IHPK1	inositol hexaphosphate kinase 1	**-1.7**	**1.8**	**-1.4**		
IKBKAP	inhibitor of kappa light polypeptide gene enhancer in B-cells, kinase complex-associated protein	**-1.3**				
IKBKE	inhibitor of kappa light polypeptide gene enhancer in B-cells, kinase epsilon	**-2.0**				
IKBKG	inhibitor of kappa light polypeptide gene enhancer in B-cells, kinase gamma	**1.6**	**-1.5**			
IL17R	interleukin 17 receptor A	**1.3**			**1.5**	
IL27RA	interleukin 27 receptor, alpha	**-7.5**	**20.9**			
IL7R	interleukin 7 receptor	**43.1**	**-56.6**	**-3.1**	**8.3**	**6.3**
IL9R	interleukin 9 receptor	**1.9**				
ILF2	interleukin enhancer binding factor 2, 45kDa	**-1.6**				
ILF3	interleukin enhancer binding factor 3, 90kDa	**-4.0**	**1.7**	**-2.2**	**-1.3**	
IMMT	inner membrane protein, mitochondrial (mitofilin)	**-1.6**	**1.4**			
IMP4	IMP4, U3 small nucleolar ribonucleoprotein, homolog (yeast)	**-1.4**			**-1.6**	
IMPA1	inositol(myo)-1(or 4)-monophosphatase 1	**2.3**	**-1.7**	**1.2**		
IMPA2	inositol(myo)-1(or 4)-monophosphatase 2	**-7.9**	**1.8**	**-12.4**		
IMPDH2	IMP (inosine monophosphate) dehydrogenase 2	**-1.5**	**1.3**	**-1.4**		**-1.5**
ING1	inhibitor of growth family, member 1	**1.6**				
ING2	inhibitor of growth family, member 2	**-2.7**		**-2.4**		
ING3	inhibitor of growth family, member 3	**3.0**	**-1.9**	**1.3**		
INPP1	inositol polyphosphate-1-phosphatase	**13.6**	**1.4**	**2.7**	**3.5**	**17.6**
INPP5D	inositol polyphosphate-5-phosphatase, 145kDa	**1.5**	**-1.6**	**2.0**		
INPP5F	inositol polyphosphate-5-phosphatase F	**1.5**		**2.1**		
INSIG1	insulin induced gene 1	**3.7**	**-5.5**	**1.7**		
IPO7	importin 7	**-4.6**	**1.5**	**-1.7**	**-1.4**	
IQCB1	IQ motif containing B1	**-1.5**	**-1.3**			**-1.5**
IQGAP1	IQ motif containing GTPase activating protein 1	**4.4**	**-2.4**	**2.2**		
IQGAP2	IQ motif containing GTPase activating protein 2	**2.0**		**2.6**	**1.4**	**1.9**
IQSEC1	IQ motif and Sec7 domain 1	**3.1**		**1.8**		
IRAK1BP1	interleukin-1 receptor-associated kinase 1 binding protein 1	**1.6**	**-1.6**	**-3.0**		
IREB2	iron-responsive element binding protein 2	**-1.9**	**1.5**			
IRF2	interferon regulatory factor 2	**2.2**	**-2.3**			
IRS1	insulin receptor substrate 1	**7.7**	**-4.8**	**1.5**		
ISG20	interferon stimulated exonuclease gene 20kDa	**274.3**		**91.9**	**9.5**	**7.6**
ITCH	itchy homolog E3 ubiquitin protein ligase (mouse)	**1.6**	**2.0**	**1.8**		
ITGA4	integrin, alpha 4 (antigen CD49D, alpha 4 subunit of VLA-4 receptor)	**-5.7**			**-2.1**	
ITGA6	integrin, alpha 6	**-1.9**	**6.1**	**7.2**	**5.0**	**2.4**
ITGAE	integrin, alpha E (antigen CD103, human mucosal lymphocyte antigen 1; alpha polypeptide)	**3.1**	**-2.9**	**1.5**		
ITGB1	integrin, beta 1 (fibronectin receptor, beta polypeptide, antigen CD29 includes MDF2, MSK12)	**-2.1**	**3.1**	**1.4**		
ITGB2	integrin, beta 2 (complement component 3 receptor 3 and 4 subunit)	**-8.7**	**2.6**	**-5.6**		
ITGB3BP	integrin beta 3 binding protein (beta3-endonexin)	**-1.7**		**-1.2**		
ITGB4BP	integrin beta 4 binding protein	**-1.4**				
ITM1	STT3, subunit of the oligosaccharyltransferase complex, homolog A (S. cerevisiae)	**-1.4**				**-1.3**

ITM2A	integral membrane protein 2A	**-138.3**	**64.4**			
ITM2B	integral membrane protein 2B	**1.9**	**1.7**	**2.0**		
ITPKB	inositol 1,4,5-trisphosphate 3-kinase B	**1.7**	**-4.6**			
ITPR1	inositol 1,4,5-triphosphate receptor, type 1	**-18.1**	**3.8**	**2.4**		
ITPR2	inositol 1,4,5-triphosphate receptor, type 2	**-2.4**	**4.3**	**1.8**	**1.5**	**1.5**
ITSN1	intersectin 1 (SH3 domain protein)	**8.7**	**-3.8**	**-1.3**		
ITSN2	intersectin 2	**1.4**		**1.4**		
IVNS1ABP	influenza virus NS1A binding protein	**-1.9**	**-1.3**	**-1.3**		
JAK1	Janus kinase 1 (a protein tyrosine kinase)	**3.4**	**1.9**	**1.8**	**2.2**	**2.6**
JAM3	junctional adhesion molecule 3	**-12.3**	**12.9**		**2.4**	**3.6**
JARID1A	jumonji, AT rich interactive domain 1A	**3.7**	**-2.3**	**1.2**		
JARID1B	jumonji, AT rich interactive domain 1B	**-8.0**	**37.6**			
JARID1C	jumonji, AT rich interactive domain 1C	**1.3**				
JARID2	jumonji, AT rich interactive domain 2	**1.9**	**-1.4**		**-1.3**	
JMJD1C	jumonji domain containing 1C	**3.6**	**-2.0**	**1.9**		
JRK	jerky homolog (mouse)	**-1.8**	**2.1**			
JTV1	JTV1 gene	**-1.7**		**-2.3**	**-1.9**	
JUN	jun oncogene	**-6.2**	**42.1**	**18.9**	**4.4**	
JUNB	jun B proto-oncogene	**-1.8**				
JUND	jun D proto-oncogene	**1.4**	**1.3**	**1.8**		
KAB	centrosomal protein 170kDa	**-4.0**	**3.8**	**1.2**		
KARS	lysyl-tRNA synthetase	**-1.7**		**-1.6**	**-1.3**	**-1.5**
KCNN4	potassium intermediate/small conductance calcium-activated channel, subfamily N, member 4	**-19.5**	**23.1**	**-13.3**		
KDELR1	KDEL (Lys-Asp-Glu-Leu) endoplasmic reticulum protein retention receptor 1	**1.3**				
KDELR2	KDEL (Lys-Asp-Glu-Leu) endoplasmic reticulum protein retention receptor 2	**-2.0**	**1.3**			
KHDRBS3	KH domain containing, RNA binding, signal transduction associated 3	**-14.3**	**9.0**	**2.3**		
KHSRP	KH-type splicing regulatory protein (FUSE binding protein 2)	**-2.2**		**-2.3**		**-1.4**
KIAA0101	KIAA0101	**-3.2**		**-1.9**		
KIAA0133	KIAA0133	**-1.6**	**-1.5**	**-1.4**		
KIAA0143	KIAA0143 protein	**-1.5**	**1.9**	**1.4**		
KIAA0247	KIAA0247	**-1.4**	**1.7**			
KIAA0310	KIAA0310	**-1.3**	**1.5**			
KIAA0368	KIAA0368	**-1.4**	**1.8**	**1.8**		
KIAA0922	KIAA0922	**1.9**	**-2.6**			
KIAA0999	KIAA0999 protein	**-2.7**	**2.2**	**-1.4**		
KIAA1008	KIAA1008	**-1.9**	**1.7**			
KIAA1009	KIAA1009	**1.7**				
KIAA1115	SAPS domain family, member 1	**-1.7**	**1.4**			
KIAA1128	KIAA1128	**1.8**				**-1.3**
KIAA1539	KIAA1539	**2.8**		**1.7**		
KIAA1794	KIAA1794	**-1.7**	**-1.9**			
KIDINS220	kinase D-interacting substance of 220 kDa	**1.5**	**1.5**	**1.4**		

KIF11	kinesin family member 11	**1.4**	**-1.6**	**1.5**		
KIF14	kinesin family member 14	**1.8**	**-1.4**	**1.4**		
KIF2	kinesin heavy chain member 2A	**1.6**	**-1.3**		**-1.2**	
KIF23	kinesin family member 23	**1.2**		**1.9**		
KIF2C	kinesin family member 2C	**-1.8**		**1.3**	**1.3**	
KIF3C	kinesin family member 3C	**2.3**				
KLF10	Kruppel-like factor 10	**1.9**	**-2.3**	**2.4**		
KLF6	Kruppel-like factor 6	**3.5**	**1.8**	**7.2**	**1.5**	
KNS2	kinesin 2	**1.7**		**1.8**		
KNTC1	kinetochore associated 1	**-1.3**	**-1.5**			
KPNA3	karyopherin alpha 3 (importin alpha 4)	**-1.8**	**-1.5**	**-1.3**		
KPNA6	karyopherin alpha 6 (importin alpha 7)	**-2.1**	**2.0**			
KPNB1	karyopherin (importin) beta 1	**-2.8**	**1.5**	**-1.6**		
KRAS	v-Ki-ras2 Kirsten rat sarcoma viral oncogene homolog	**13.4**	**-2.1**	**13.3**		
KRIT1	KRIT1, ankyrin repeat containing	**1.3**				
KRT10	keratin 10 (epidermolytic hyperkeratosis; keratosis palmaris et plantaris)	**2.1**	**-2.8**	**1.2**		
KRT8	keratin 8	**1.6**	**-1.4**			
KTN1	kinectin 1 (kinesin receptor)	**-1.4**	**1.8**			
LAGE3	L antigen family, member 3	**-3.3**		**-1.9**		
LAIR1	leukocyte-associated immunoglobulin-like receptor 1	**-1.9**	**6.7**	**2.7**	**1.7**	**1.6**
LAMP1	lysosomal-associated membrane protein 1	**-1.4**	**1.8**	**1.5**		
LAMP2	lysosomal-associated membrane protein 2	**-2.3**	**2.1**	**1.7**		
LANCL1	LanC lantibiotic synthetase component C-like 1 (bacterial)	**1.4**	**-1.7**			
LAP1B	torsin A interacting protein 1	**-2.0**	**1.4**			
LAPTM5	lysosomal associated multispanning membrane protein 5	**10.9**	**-4.3**	**2.0**		
LARP1	La ribonucleoprotein domain family, member 1	**-2.2**		**-2.0**		
LARS2	leucyl-tRNA synthetase 2, mitochondrial	**-1.4**	**-1.2**	**-1.5**		
LAS1L	LAS1-like (S. cerevisiae)	**-2.0**				**-1.4**
LCK	lymphocyte-specific protein tyrosine kinase	**18.5**	**-49.3**			**-1.7**
LCP1	lymphocyte cytosolic protein 1 (L-plastin)	**7.2**	**-4.1**	**-2.5**		
LCP2	lymphocyte cytosolic protein 2 (SH2 domain containing leukocyte protein of 76kDa)	**-2.2**	**1.6**	**-2.4**		
LDB1	LIM domain binding 1	**-1.5**	**1.7**	**-1.2**		
LDHA	lactate dehydrogenase A	**-1.8**		**-1.7**		
LDLR	low density lipoprotein receptor (familial hypercholesterolemia)	**3.0**	**-3.6**	**-1.3**		
LEF1	lymphoid enhancer-binding factor 1	**826.7**	**-288.8**	**-1.3**	**1.5**	
LEPR	leptin receptor	**4.6**	**1.8**	**2.2**		
LETMD1	LETM1 domain containing 1	**-7.5**	**3.1**			
LGALS1	lectin, galactoside-binding, soluble, 1 (galectin 1)	**82.1**	**-22.2**	**2.9**		
LGALS3BP	lectin, galactoside-binding, soluble, 3 binding protein	**2.8**	**-4.3**	**-2.3**		
LGALS9	lectin, galactoside-binding, soluble, 9 (galectin 9)	**-2.4**			**-1.5**	**-2.1**
LIG1	ligase I, DNA, ATP-dependent	**-5.5**				
LIG3	ligase III, DNA, ATP-dependent	**-1.4**				

LIMS1	LIM and senescent cell antigen-like domains 1	3.7	1.3	1.3		
LMAN1	lectin, mannose-binding, 1	-15.2	1.6		-1.5	
LMNB2	lamin B2	-1.5				
LMO2	LIM domain only 2 (rhombotin-like 1)	-6.9	7.9	4.4		
LNK	SH2B adaptor protein 3	-2.8	1.3	-1.2		
LOC162427	hypothetical protein LOC162427	1.5				
LOC93081	chromosome 13 open reading frame 27	-1.4	2.0			
LPIN1	lipin 1	2.3	-1.6		1.3	
LPXN	leupaxin	1.7	-3.2	-1.7		
LRBA	LPS-responsive vesicle trafficking, beach and anchor containing	-1.6	1.4	-2.1		
LRCH4	leucine-rich repeats and calponin homology (CH) domain containing 4	4.1	-1.9	1.9		
LRMP	lymphoid-restricted membrane protein	7.8	-18.6	-1.4		
LRP4	low density lipoprotein receptor-related protein 4	-16.6				
LRP8	low density lipoprotein receptor-related protein 8, apolipoprotein e receptor	-2.5	-1.6	-2.1	-2.2	-2.8
LRPAP1	low density lipoprotein receptor-related protein associated protein 1	-2.1	3.2			
LRPPRC	leucine-rich PPR-motif containing	-2.8	1.7	-5.1	-1.8	-1.7
LRRFIP1	leucine rich repeat (in FLII) interacting protein 1	11.3	-6.0	2.5		
LSM2	LSM2 homolog, U6 small nuclear RNA associated (S. cerevisiae)	-1.3				
LSM4	LSM4 homolog, U6 small nuclear RNA associated (S. cerevisiae)	-1.9	-1.6	-1.4		
LSM5	LSM5 homolog, U6 small nuclear RNA associated (S. cerevisiae)	-4.5	2.9			
LSM6	LSM6 homolog, U6 small nuclear RNA associated (S. cerevisiae)	2.2		-1.4		
LSM7	LSM7 homolog, U6 small nuclear RNA associated (S. cerevisiae)	-1.4	-1.5	-1.5		
LTA4H	leukotriene A4 hydrolase	-2.9	2.6			
LTK	leukocyte tyrosine kinase	2.4	-2.3			
LY6E	lymphocyte antigen 6 complex, locus E	-12.6	2.0			
LYL1	lymphoblastic leukemia derived sequence 1	-6.7	3.5			1.4
LYPLA1	lysophospholipase I	1.2	-1.3	1.3		
M11S1	GPI-anchored membrane protein 1	-1.5	-2.0	-1.3		
M6PR	mannose-6-phosphate receptor (cation dependent)	-2.2		-2.3		
MAC30	transmembrane protein 97	-3.2	1.4	-3.0	-1.3	
MACF1	microtubule-actin crosslinking factor 1	2.7	-1.5	-1.3		
MADD	MAP-kinase activating death domain	1.4				
MAG	myelin associated glycoprotein	2.0	-40.4	-2.3		
MAGED1	melanoma antigen family D, 1	-4.1	2.4	-2.1		
MAGOH	mago-nashi homolog, proliferation-associated (Drosophila)	-1.5	-1.2			
MALT1	mucosa associated lymphoid tissue lymphoma translocation gene 1	2.2	-1.7	-1.7		
MAN2A1	mannosidase, alpha, class 2A, member 1	-1.9	2.5	1.7		
MAN2A2	mannosidase, alpha, class 2A, member 2	-4.8	8.4	1.3		
MAN2B1	mannosidase, alpha, class 2B, member 1	-2.9	2.4			
MAP1A	microtubule-associated protein 1A	10.8			2.0	1.7
MAP1LC3B	microtubule-associated protein 1 light chain 3 beta	3.2		3.2		
MAP2K1	mitogen-activated protein kinase kinase 1	2.1	1.5	1.9	1.5	1.7

MAP2K2	mitogen-activated protein kinase kinase 2	**-1.6**	**1.3**			
MAP2K3	mitogen-activated protein kinase kinase 3	**1.9**	**-2.3**			
MAP2K4	mitogen-activated protein kinase kinase 4	**1.7**	**-1.4**			
MAP2K5	mitogen-activated protein kinase kinase 5	**2.1**				
MAP3K11	mitogen-activated protein kinase kinase kinase 11	**-1.7**	**1.3**			
MAP3K7IP2	mitogen-activated protein kinase kinase kinase 7 interacting protein 2	**2.1**	**-1.4**	**1.5**		
MAP4	microtubule-associated protein 4	**1.6**			**-1.3**	
MAP4K1	mitogen-activated protein kinase kinase kinase kinase 1	**2.4**	**-2.6**	**-2.7**		
MAP4K2	mitogen-activated protein kinase kinase kinase kinase 2	**-5.4**				
MAP4K4	mitogen-activated protein kinase kinase kinase kinase 4	**1.7**	**-1.5**	**1.7**		
MAPK1	mitogen-activated protein kinase 1	**2.0**	**-1.4**	**2.3**		
MAPK14	mitogen-activated protein kinase 14	**-2.1**	**4.1**			**1.4**
MAPK3	mitogen-activated protein kinase 3	**4.6**				
MAPK6	mitogen-activated protein kinase 6	**-1.4**			**-1.5**	**-1.8**
MAPK9	mitogen-activated protein kinase 9	**-1.4**			**-1.5**	**-1.9**
MAPKAPK2	mitogen-activated protein kinase-activated protein kinase 2	**1.3**		**1.5**	**1.3**	
MAPKAPK3	mitogen-activated protein kinase-activated protein kinase 3	**-20.4**	**10.5**	**-1.5**	**-1.4**	
MAPRE1	microtubule-associated protein, RP/EB family, member 1	**1.6**	**-1.4**	**1.5**		
MAPRE2	microtubule-associated protein, RP/EB family, member 2	**3.0**	**-3.4**	**-2.5**		
MARS	methionine-tRNA synthetase	**-2.2**	**-1.5**	**-2.2**		
MATR3	matrin 3	**-10.9**		**-14.6**		
MAZ	MYC-associated zinc finger protein (purine-binding transcription factor)	**1.7**	**-3.2**	**-1.7**		
MBD1	methyl-CpG binding domain protein 1	**-1.8**	**1.3**			
MBD2	methyl-CpG binding domain protein 2	**1.3**	**-1.6**			
MBD4	methyl-CpG binding domain protein 4	**-2.2**	**1.7**			
MBNL1	muscleblind-like (Drosophila)	**9.2**	**-4.4**	**2.0**		
MBTPS1	membrane-bound transcription factor peptidase, site 1	**-1.6**				
MCCC2	methylcrotonoyl-Coenzyme A carboxylase 2 (beta)	**-2.1**	**1.4**	**-1.5**		
MCFD2	multiple coagulation factor deficiency 2	**-1.7**	**1.6**			
MCL1	myeloid cell leukemia sequence 1 (BCL2-related)	**1.3**	**1.4**	**1.7**	**1.2**	
MCM2	MCM2 minichromosome maintenance deficient 2, mitotin (S. cerevisiae)	**-4.2**		**-3.1**		
MCM3	MCM3 minichromosome maintenance deficient 3 (S. cerevisiae)	**-4.2**	**-1.4**	**-3.1**		**-1.3**
MCM3AP	MCM3 minichromosome maintenance deficient 3 (S. cerevisiae) associated protein	**1.9**			**1.3**	
MCM4	MCM4 minichromosome maintenance deficient 4 (S. cerevisiae)	**-6.9**	**-2.1**	**-3.3**		
MCM5	MCM5 minichromosome maintenance deficient 5, cell division cycle 46 (S. cerevisiae)	**-3.2**	**-1.5**	**-1.5**		
MCM6	minichromosome maintenance deficient 6 homolog (S. cerevisiae)	**-6.8**	**1.3**	**-2.1**		
MCM7	MCM7 minichromosome maintenance deficient 7 (S. cerevisiae)	**-2.7**	**-1.9**	**-1.3**		
MDC1	mediator of DNA damage checkpoint 1	**2.0**	**-4.7**			
MDH1	malate dehydrogenase 1, NAD (soluble)	**-2.2**	**1.9**	**-1.3**		
MDH2	malate dehydrogenase 2, NAD (mitochondrial)	**3.7**	**-2.3**	**-1.5**		
MDM4	Mdm4, transformed 3T3 cell double minute 4, p53 binding protein (mouse)	**2.5**	**-2.4**	**2.2**		
ME2	malic enzyme 2, NAD(+)-dependent, mitochondrial	**-2.2**		**-1.6**		**-1.8**

MEF2A	MADS box transcription enhancer factor 2, polypeptide A (myocyte enhancer factor 2A)	**11.0**	**-3.6**	**1.7**		
MEF2D	MADS box transcription enhancer factor 2, polypeptide D (myocyte enhancer factor 2D)	**2.0**	**-5.7**			
MEIS2	Meis1, myeloid ecotropic viral integration site 1 homolog 2 (mouse)	**-4.4**	**3.6**	**-2.1**		
MEN1	multiple endocrine neoplasia I	**-1.3**	**1.3**	**-1.5**		
MEP50	WD repeat domain 77	**-1.4**	**-1.8**	**-1.5**	**-1.7**	
MFHAS1	malignant fibrous histiocytoma amplified sequence 1	**-4.1**		**-1.5**		
MFN1	mitofusin 1	**1.9**				
MFN2	mitofusin 2	**-1.6**				
MFNG	MFNG O-fucosylpeptide 3-beta-N-acetylglucosaminyltransferase	**-9.2**	**3.0**	**-1.8**		
MGA	MAX gene associated	**-7.2**	**3.0**	**-1.8**		
MGC17330	HGFL gene	**12.3**	**34.8**	**7.3**	**7.3**	**5.5**
MGC5508	transmembrane protein 109	**-1.5**	**-1.4**	**-1.7**	**-1.4**	**-1.6**
MGEA5	meningioma expressed antigen 5 (hyaluronidase)	**1.9**	**1.7**	**1.3**		
MIF	macrophage migration inhibitory factor (glycosylation-inhibiting factor)	**-2.6**		**-1.9**		
MINA	MYC induced nuclear antigen	**-1.9**	**-1.2**	**-1.7**		
MINPP1	multiple inositol polyphosphate histidine phosphatase, 1	**-1.7**	**1.6**			
MIR16	membrane interacting protein of RGS16	**-2.9**	**2.1**	**-1.4**		
MKI67	antigen identified by monoclonal antibody Ki-67	**2.2**	**-2.2**	**2.1**		
MKL1	megakaryoblastic leukemia (translocation) 1	**-1.6**	**1.5**	**-1.5**		
MKNK1	MAP kinase interacting serine/threonine kinase 1	**1.3**	**1.3**			**1.9**
MKRN1	makorin, ring finger protein, 1	**2.1**	**-1.5**			
MLC1	megalencephalic leukoencephalopathy with subcortical cysts 1	**-5.1**	**1.7**			**-1.5**
MLH1	mutL homolog 1, colon cancer, nonpolyposis type 2 (E. coli)	**-1.5**		**-1.6**		
MLH3	mutL homolog 3 (E. coli)	**-2.4**	**2.2**	**-1.3**		
MLL	myeloid/lymphoid or mixed-lineage leukemia (trithorax homolog, Drosophila)	**2.0**	**1.8**	**-1.8**		
MLLT10	myeloid/lymphoid or mixed-lineage leukemia (trithorax homolog, Drosophila); translocated to, 10	**-2.2**	**2.7**			
MMD	monocyte to macrophage differentiation-associated	**-2.7**		**-2.6**		
MMS19L	MMS19-like (MET18 homolog, S. cerevisiae)	**-1.4**	**1.7**			**-1.3**
MOAP1	modulator of apoptosis 1	**1.5**		**1.5**	**1.9**	
MOBK1B	MOB1, Mps One Binder kinase activator-like 1B (yeast)	**1.4**	**-1.6**			
MPG	N-methylpurine-DNA glycosylase	**4.1**	**-4.1**			
MPHOSPH1	M-phase phosphoprotein 1	**-3.2**	**1.9**			
MPHOSPH10	M-phase phosphoprotein 10 (U3 small nucleolar ribonucleoprotein)	**-1.3**		**-1.3**		
MPHOSPH6	M-phase phosphoprotein 6	**-2.3**	**-1.3**	**-1.9**		
MPHOSPH9	M-phase phosphoprotein 9	**-1.4**		**-1.3**		
MPP6	membrane protein, palmitoylated 6 (MAGUK p55 subfamily member 6)	**-21.9**	**38.6**			
MPV17	MpV17 mitochondrial inner membrane protein	**-1.9**		**-1.4**		
MPZL1	myelin protein zero-like 1	**9.8**	**-7.5**	**-1.8**		
MR1	major histocompatibility complex, class I-related	**3.5**				
MRC2	mannose receptor, C type 2	**-8.0**	**2.0**			
MRCL3	myosin regulatory light chain MRCL3	**5.8**	**-2.0**	**2.2**		
MRE11A	MRE11 meiotic recombination 11 homolog A (S. cerevisiae)	**-1.7**				

M-RIP	myosin phosphatase-Rho interacting protein	**26.4**	**-5.4**	**1.2**		
MRPL12	mitochondrial ribosomal protein L12	**-3.2**		**-1.7**		
MRPS12	mitochondrial ribosomal protein S12	**-1.7**	**-1.5**		**-1.4**	**-1.7**
MRPS27	mitochondrial ribosomal protein S27	**-1.5**		**-1.5**		
MSH2	mutS homolog 2, colon cancer, nonpolyposis type 1 (E. coli)	**-1.8**	**-1.5**	**-1.6**		**-1.5**
MSH3	mutS homolog 3 (E. coli)	**1.7**		**-1.5**		
MSH6	mutS homolog 6 (E. coli)	**-2.4**	**-2.5**	**-2.7**	**-1.2**	**-1.7**
MSN	moesin	**1.7**	**1.4**	**1.2**		
MSX1	msh homeobox 1	**-1.4**		**-1.6**	**1.5**	
MT1H	metallothionein 1H	**1.3**	**1.4**	**3.0**		**2.1**
MT1X	metallothionein 1X	**1.7**	**1.6**	**1.8**		
MT2A	metallothionein 2A	**1.2**	**3.2**	**3.0**		
MTA1	metastasis associated 1	**-1.6**	**1.4**	**-1.4**		
MTHFD1	methylenetetrahydrofolate dehydrogenase (NADP+ dependent) 1, methenyltetrahydrofolate cyclohydrolase, formyltetrahydrofolate synthetase	**-5.0**	**1.5**	**-2.2**	**-1.4**	**-1.5**
MTHFD2	methylenetetrahydrofolate dehydrogenase (NADP+ dependent) 2, methenyltetrahydrofolate cyclohydrolase	**-2.7**		**-2.0**	**-2.1**	**-1.7**
MTIF2	mitochondrial translational initiation factor 2	**-1.3**				**-1.3**
MTM1	myotubularin 1	**-2.5**	**2.5**			
MTMR3	myotubularin related protein 3	**1.9**	**-1.4**	**1.5**		
MTMR6	myotubularin related protein 6	**1.5**	**-1.4**	**1.7**		
MTR	5-methyltetrahydrofolate-homocysteine methyltransferase	**-1.7**		**1.4**		**-1.3**
MTRR	5-methyltetrahydrofolate-homocysteine methyltransferase reductase	**-1.8**		**-1.2**		
MTX2	metaxin 2	**-2.1**	**1.3**			
MUT	methylmalonyl Coenzyme A mutase	**2.0**	**-1.6**			
MVK	mevalonate kinase (mevalonic aciduria)	**1.4**				
MXI1	MAX interactor 1	**-6.2**	**6.4**	**-1.7**	**-2.7**	
MYB	v-myb myeloblastosis viral oncogene homolog (avian)	**-2.0**	**2.2**	**1.8**		
MYBL2	v-myb myeloblastosis viral oncogene homolog (avian)-like 2	**-2.5**				
MYC	v-myc myelocytomatosis viral oncogene homolog (avian)	**-4.8**	**1.6**	**-2.8**	**-3.8**	**-3.6**
MYCBP	c-myc binding protein	**-1.6**	**1.3**	**-1.2**		
MYH10	myosin, heavy chain 10, non-muscle	**3.8**	**-3.7**	**-1.3**		
MYH9	myosin, heavy chain 9, non-muscle	**2.2**	**-1.8**			**1.6**
MYL6	myosin, light chain 6, alkali, smooth muscle and non-muscle	**1.4**		**1.5**		
MYO1B	myosin IB	**-6.5**	**3.5**	**2.3**	**11.8**	
MYO5A	myosin VA (heavy chain 12, myoxin)	**6.2**	**-8.2**			
MYO9B	myosin IXB	**-1.3**		**-1.3**		**1.3**
MYST1	MYST histone acetyltransferase 1	**2.3**	**-1.9**			
MYST2	MYST histone acetyltransferase 2	**-1.3**		**-1.3**		
MYST3	MYST histone acetyltransferase (monocytic leukemia) 3	**1.4**		**1.4**		
MYST4	MYST histone acetyltransferase (monocytic leukemia) 4	**-2.6**	**2.2**	**-1.9**		
NAB1	NGFI-A binding protein 1 (EGR1 binding protein 1)	**-3.2**				
NACA	nascent-polypeptide-associated complex alpha polypeptide	**-1.9**	**1.3**			
NADK	NAD kinase	**-1.9**		**1.3**		

NAGA	N-acetylgalactosaminidase, alpha-	**-2.4**	**1.6**			
NAP1L1	nucleosome assembly protein 1-like 1	**-2.9**	**1.9**	**-1.2**	**-1.3**	
NAP1L4	nucleosome assembly protein 1-like 4	**-1.3**	**-1.5**			
NARS	asparaginyl-tRNA synthetase	**-1.6**		**-1.4**	**-1.6**	
NASP	nuclear autoantigenic sperm protein (histone-binding)	**-5.5**		**-2.4**		
NBL1	neuroblastoma, suppression of tumorigenicity 1	**-3.0**	**2.3**			
NBR1	neighbor of BRCA1 gene 1	**-1.2**	**2.6**	**1.5**		
NBS1	nibrin	**1.8**	**-1.6**	**-1.2**	**1.9**	**1.7**
NCF4	neutrophil cytosolic factor 4, 40kDa	**1.6**	**-1.9**	**-1.5**		
NCKAP1	NCK-associated protein 1	**-5.9**	**6.0**			
NCKIPSD	NCK interacting protein with SH3 domain	**-1.8**				
NCL	nucleolin	**-1.2**	**-1.6**	**-1.9**	**-1.3**	
NCLN	nicalin homolog (zebrafish)	**-3.0**			**-1.5**	**-1.8**
NCOA3	nuclear receptor coactivator 3	**3.8**	**-1.4**	**2.1**		
NCOA4	nuclear receptor coactivator 4	**-1.4**	**1.9**	**1.3**	**1.5**	
NCOR2	nuclear receptor co-repressor 2	**1.3**		**1.5**		
NDP52	calcium binding and coiled-coil domain 2	**2.7**				
NDRG1	N-myc downstream regulated gene 1	**2.3**	**9.2**	**3.1**	**1.6**	
NDUFA2	NADH dehydrogenase (ubiquinone) 1 alpha subcomplex, 2, 8kDa	**-1.3**				
NDUFA5	NADH dehydrogenase (ubiquinone) 1 alpha subcomplex, 5, 13kDa	**1.9**	**-2.4**			
NDUFAF1	NADH dehydrogenase (ubiquinone) 1 alpha subcomplex, assembly factor 1	**2.5**	**-3.0**	**-1.8**		**-1.2**
NDUFB1	NADH dehydrogenase (ubiquinone) 1 beta subcomplex, 1, 7kDa	**-1.3**				
NDUFB3	NADH dehydrogenase (ubiquinone) 1 beta subcomplex, 3, 12kDa	**-1.8**	**1.2**			
NDUFB5	NADH dehydrogenase (ubiquinone) 1 beta subcomplex, 5, 16kDa	**-1.8**	**1.7**			
NDUFB8	NADH dehydrogenase (ubiquinone) 1 beta subcomplex, 8, 19kDa	**-2.1**	**1.2**			
NDUFC1	NADH dehydrogenase (ubiquinone) 1, subcomplex unknown, 1, 6kDa	**-2.0**	**1.4**			
NDUFS1	NADH dehydrogenase (ubiquinone) Fe-S protein 1, 75kDa (NADH-coenzyme Q reductase)	**1.4**	**-1.6**	**-1.5**		
NDUFS2	NADH dehydrogenase (ubiquinone) Fe-S protein 2, 49kDa (NADH-coenzyme Q reductase)	**-1.7**	**1.9**			
NDUFS3	NADH dehydrogenase (ubiquinone) Fe-S protein 3, 30kDa (NADH-coenzyme Q reductase)	**-1.7**		**-1.4**		
NDUFS4	NADH dehydrogenase (ubiquinone) Fe-S protein 4, 18kDa (NADH-coenzyme Q reductase)	**1.6**				
NDUFS6	NADH dehydrogenase (ubiquinone) Fe-S protein 6, 13kDa (NADH-coenzyme Q reductase)	**-1.8**	**-1.5**			
NDUFS7	NADH dehydrogenase (ubiquinone) Fe-S protein 7, 20kDa (NADH-coenzyme Q reductase)	**-1.7**		**-1.6**		
NDUFS8	NADH dehydrogenase (ubiquinone) Fe-S protein 8, 23kDa (NADH-coenzyme Q reductase)	**-2.2**		**-1.5**		
NDUFV1	NADH dehydrogenase (ubiquinone) flavoprotein 1, 51kDa	**-2.0**	**-1.6**	**-1.4**		
NEBL	nebulette	**1.8**				
NECAP1	NECAP endocytosis associated 1	**1.6**	**-1.3**	**-1.3**		
NEDD4	neural precursor cell expressed, developmentally down-regulated 4	**-3.0**	**2.8**			
NEK2	NIMA (never in mitosis gene a)-related kinase 2	**2.1**	**-1.9**	**1.5**		
NF1	neurofibromin 1 (neurofibromatosis, von Recklinghausen disease, Watson disease)	**-1.4**		**2.0**		
NFE2L1	nuclear factor (erythroid-derived 2)-like 1	**-1.5**	**1.6**			**-1.4**
NFE2L2	nuclear factor (erythroid-derived 2)-like 2	**-2.1**	**2.2**	**1.3**		
NFIC	nuclear factor I/C (CCAAT-binding transcription factor)	**-8.4**	**5.9**	**-1.9**		

NFIL3	nuclear factor, interleukin 3 regulated	19.1	-2.5	5.0	6.0	3.6
NFKB2	nuclear factor of kappa light polypeptide gene enhancer in B-cells 2 (p49/p100)	1.7	-1.5			
NFKBIA	nuclear factor of kappa light polypeptide gene enhancer in B-cells inhibitor, alpha	5.5	1.9	2.5	3.0	2.8
NFRKB	nuclear factor related to kappaB binding protein	-6.3	2.2			
NFX1	nuclear transcription factor, X-box binding 1	-1.3				
NFYA	nuclear transcription factor Y, alpha	-1.9	1.6			
NFYB	nuclear transcription factor Y, beta	1.3	-1.6			
NHP2L1	NHP2 non-histone chromosome protein 2-like 1 (S. cerevisiae)	-1.9		-1.6		
NIPSNAP1	nipsnap homolog 1 (C. elegans)	-1.8		-2.9		-1.8
NISCH	nischarin	3.1				
NIT1	nitrilase 1	1.3				
NKRF	NF-kappaB repressing factor	-1.3				
NMB	neuromedin B	-2.0	1.4			
NME1	non-metastatic cells 1, protein (NM23A) expressed in	-7.6		-4.4	-1.5	-1.7
NME2	non-metastatic cells 2, protein (NM23B) expressed in	-1.6				
NME4	non-metastatic cells 4, protein expressed in	-2.5		-2.1		
NME6	non-metastatic cells 6, protein expressed in (nucleoside-diphosphate kinase)	-1.6	1.6			
NMI	N-myc (and STAT) interactor	-1.8	1.2			
NMT1	N-myristoyltransferase 1	-1.3		-1.3		
NNT	nicotinamide nucleotide transhydrogenase	-1.3	1.3			
NOC2L	nucleolar complex associated 2 homolog (S. cerevisiae)	-1.4		-1.8		-1.3
NOL5A	nucleolar protein 5A (56kDa with KKE/D repeat)	-3.0		-2.0	-1.9	
NOLA2	nucleolar protein family A, member 2 (H/ACA small nucleolar RNPs)	-2.0	-1.5	-1.8		
NOLC1	nucleolar and coiled-body phosphoprotein 1	-3.9	1.5	-2.0	-2.1	
NP	nucleoside phosphorylase	-2.7		-1.3	-1.8	-2.0
NPC1	Niemann-Pick disease, type C1	2.7		1.5		
NPM1	nucleophosmin (nucleolar phosphoprotein B23, numatrin)	-2.6	1.2	-1.9		
NPM3	nucleophosmin/nucleoplasmin, 3	-4.8	2.5		-1.4	
NQO2	NAD(P)H dehydrogenase, quinone 2	-3.4	2.2	-1.5		
NR3C1	nuclear receptor subfamily 3, group C, member 1 (glucocorticoid receptor)	6.5	-1.8	3.1	4.2	2.1
NRAS	neuroblastoma RAS viral (v-ras) oncogene homolog	1.8	-1.4			
NRD1	nardilysin (N-arginine dibasic convertase)	-1.6	1.7			
NSDHL	NAD(P) dependent steroid dehydrogenase-like	1.5				
NSEP1	Y box binding protein 1	-1.8		-1.7		
NSF	N-ethylmaleimide-sensitive factor	-1.8	1.4	-1.3		
NSFL1C	NSFL1 (p97) cofactor (p47)	-2.2	1.5	1.5		
NSUN5C	NOL1/NOP2/Sun domain family, member 5C	1.2	-1.7			
NT5C2	5'-nucleotidase, cytosolic II	-1.5	2.3	1.5		
NTE	patatin-like phospholipase domain containing 6	1.3				
NUCB2	nucleobindin 2	-3.1	1.3	-1.3		
NUDC	nuclear distribution gene C homolog (A. nidulans)	-2.1	-1.2	-1.5		
NUDT1	nudix (nucleoside diphosphate linked moiety X)-type motif 1	1.8	-2.3			

NUDT3	nudix (nucleoside diphosphate linked moiety X)-type motif 3	**-3.0**	**1.9**	**-1.2**		
NUFIP1	nuclear fragile X mental retardation protein interacting protein 1	**-2.2**	**-1.8**	**-1.8**		
NUMA1	nuclear mitotic apparatus protein 1	**1.9**	**-2.1**			
NUMB	numb homolog (Drosophila)	**1.6**				
NUP133	nucleoporin 133kDa	**-1.3**				
NUP153	nucleoporin 153kDa	**1.4**	**-2.0**	**-1.4**	**-1.5**	
NUP155	nucleoporin 155kDa	**-2.3**	**-1.5**			
NUP160	nucleoporin 160kDa	**-2.0**				
NUP205	nucleoporin 205kDa	**-1.3**	**-1.2**	**-1.5**		
NUP210	nucleoporin 210kDa	**-1.5**	**-3.4**	**-1.7**		
NUP62	nucleoporin 62kDa	**-2.6**	**-1.4**		**-1.4**	
NUP93	nucleoporin 93kDa	**-2.3**				
NUP98	nucleoporin 98kDa	**1.4**	**-1.7**	**-2.0**		**-1.3**
NUPL2	nucleoporin like 2	**-3.5**	**3.8**			
OAS2	2'-5'-oligoadenylate synthetase 2, 69/71kDa	**2.9**	**-3.2**	**-2.8**		
OAZ1	ornithine decarboxylase antizyme 1	**1.3**	**-1.3**	**1.4**		
OAZ2	ornithine decarboxylase antizyme 2	**1.4**			**1.4**	
OAZIN	antizyme inhibitor 1	**1.9**	**-2.2**			
OCRL	oculocerebrorenal syndrome of Lowe	**-2.2**				
ODC1	ornithine decarboxylase 1	**-6.6**	**-1.2**	**-1.5**	**-2.1**	**-2.2**
OGDH	oxoglutarate (alpha-ketoglutarate) dehydrogenase (lipoamide)	**-2.7**	**1.8**	**-1.7**		
OGG1	8-oxoguanine DNA glycosylase	**6.2**	**-3.9**			**1.4**
OGT	O-linked N-acetylglucosamine (GlcNAc) transferase (UDP-N-acetylglucosamine:polypeptide-N-acetylglucosaminyl transferase)	**2.3**	**2.0**	**1.3**		**2.0**
OIP5	Opa interacting protein 5	**1.7**	**-2.6**			
OPA1	optic atrophy 1 (autosomal dominant)	**-1.6**	**1.5**			
OPRS1	opioid receptor, sigma 1	**-2.6**				
ORC1L	origin recognition complex, subunit 1-like (yeast)	**-22.9**	**1.7**			
ORC2L	origin recognition complex, subunit 2-like (yeast)	**-3.0**	**2.6**			
ORC3L	origin recognition complex, subunit 3-like (yeast)	**-1.8**	**1.3**			
ORC5L	origin recognition complex, subunit 5-like (yeast)	**-1.9**	**1.3**		**-1.3**	**-1.5**
OS9	amplified in osteosarcoma	**1.7**			**1.5**	**1.4**
OSBP	oxysterol binding protein	**1.7**	**1.4**			
OSBPL8	oxysterol binding protein-like 8	**1.4**	**-1.3**			
OSGEP	O-sialoglycoprotein endopeptidase	**-2.0**				
OSTF1	osteoclast stimulating factor 1	**1.6**	**-1.3**	**1.2**		
OXCT1	3-oxoacid CoA transferase 1	**-1.5**				
OXSR1	oxidative-stress responsive 1	**-1.7**	**1.3**			
P29	SYF2 homolog, RNA splicing factor (S. cerevisiae)	**1.4**		**1.4**		
P2RX5	purinergic receptor P2X, ligand-gated ion channel, 5	**6.6**	**-2.0**	**5.5**		**2.7**
P4HA1	procollagen-proline, 2-oxoglutarate 4-dioxygenase (proline 4-hydroxylase), alpha polypeptide I	**-1.5**	**3.0**		**-1.3**	
P4HB	procollagen-proline, 2-oxoglutarate 4-dioxygenase (proline 4-hydroxylase), beta polypeptide	**-3.0**	**3.7**			**1.5**
P53CSV	TP53 regulated inhibitor of apoptosis 1	**-2.1**		**-1.4**		

PA2G4	proliferation-associated 2G4, 38kDa	**-3.5**		**-2.5**	**-1.7**	
PABPC1	poly(A) binding protein, cytoplasmic 1	**1.7**	**-2.2**	**-1.4**		
PABPC4	poly(A) binding protein, cytoplasmic 4 (inducible form)	**-1.4**		**-1.8**		
PABPN1	poly(A) binding protein, nuclear 1	**-1.5**	**1.6**	**-1.3**		
PACSIN2	protein kinase C and casein kinase substrate in neurons 2	**1.3**		**1.4**		**2.3**
PAFAH1B1	platelet-activating factor acetylhydrolase, isoform Ib, alpha subunit 45kDa	**2.9**	**-2.4**	**1.2**		
PAFAH1B3	platelet-activating factor acetylhydrolase, isoform Ib, gamma subunit 29kDa	**-1.8**			**-1.5**	
PAICS	phosphoribosylaminoimidazole carboxylase, phosphoribosylaminoimidazole succinocarboxamide synthetase	**-12.5**	**1.7**	**-3.3**	**-1.7**	
PAIP1	poly(A) binding protein interacting protein 1	**-1.8**		**-1.4**		
PAI-RBP1	SERPINE1 mRNA binding protein 1	**-2.2**	**-1.5**	**-1.9**	**-1.6**	
PAK1	p21/Cdc42/Rac1-activated kinase 1 (STE20 homolog, yeast)	**-3.4**	**2.5**	**-1.3**	**1.5**	
PAK2	p21 (CDKN1A)-activated kinase 2	**2.0**	**-1.6**	**1.5**	**-1.2**	
PAM	peptidylglycine alpha-amidating monooxygenase	**-1.6**	**1.3**	**-1.4**	**1.5**	
PAPOLA	poly(A) polymerase alpha	**-1.4**	**1.4**			
PARD3	par-3 partitioning defective 3 homolog (C. elegans)	**3.0**	**2.5**			
PARG	poly (ADP-ribose) glycohydrolase	**-1.7**				
PARK7	Parkinson disease (autosomal recessive, early onset) 7	**-1.3**				
PARN	poly(A)-specific ribonuclease (deadenylation nuclease)	**2.2**	**-2.1**			
PARP1	poly (ADP-ribose) polymerase family, member 1	**2.2**	**-2.5**	**1.7**		
PARP2	poly (ADP-ribose) polymerase family, member 2	**-1.7**		**-1.6**		**-1.7**
PAWR	PRKC, apoptosis, WT1, regulator	**-46.4**	**42.6**	**-2.4**	**-1.2**	
PAX8	paired box gene 8	**-3.2**	**3.7**			
PAXIP1L	PAX interacting (with transcription-activation domain) protein 1	**12.7**	**-3.5**	**1.2**	**1.4**	**1.3**
PBEF1	pre-B-cell colony enhancing factor 1	**-1.6**	**1.7**			
PBP	phosphatidylethanolamine binding protein 1	**-2.4**		**-2.0**		
PBX3	pre-B-cell leukemia transcription factor 3	**135.1**	**-172.9**	**-1.5**		
PC4	SUB1 homolog (S. cerevisiae)	**-4.6**	**2.4**	**-1.4**		
PCBP2	poly(rC) binding protein 2	**-1.9**	**2.0**			
PCCA	propionyl Coenzyme A carboxylase, alpha polypeptide	**1.7**		**-1.2**		**-1.3**
PCCB	propionyl Coenzyme A carboxylase, beta polypeptide	**-1.7**	**1.3**			
PCF11	PCF11, cleavage and polyadenylation factor subunit, homolog (S. cerevisiae)	**2.6**	**-2.1**			
PCGF4	B lymphoma Mo-MLV insertion region (mouse)	**1.2**	**-1.5**	**1.3**		
PCID1	PCI domain containing 1 (herpesvirus entry mediator)	**-1.6**	**1.2**			
PCK2	phosphoenolpyruvate carboxykinase 2 (mitochondrial)	**-1.4**		**-1.7**		
PCM1	pericentriolar material 1	**-1.9**	**2.3**	**1.8**		
PCMT1	protein-L-isoaspartate (D-aspartate) O-methyltransferase	**1.4**	**-1.3**	**1.4**		
PCNA	proliferating cell nuclear antigen	**-3.7**	**-1.7**	**-1.6**		
PCTK2	PCTAIRE protein kinase 2	**1.8**	**-1.2**			
PDAP1	PDGFA associated protein 1	**3.2**	**-3.1**			
PDCD10	programmed cell death 10	**1.3**				
PDCD11	programmed cell death 11	**-10.1**		**-1.9**		
PDCD2	programmed cell death 2	**-2.0**	**2.0**	**1.3**		

PDCD6	programmed cell death 6	**20.7**	**-11.8**	**2.0**		
PDE3B	phosphodiesterase 3B, cGMP-inhibited	**-25.2**	**6.1**	**-1.4**		
PDE6D	phosphodiesterase 6D, cGMP-specific, rod, delta	**2.3**	**-2.1**	**1.2**		
PDE8A	phosphodiesterase 8A	**1.6**	**-1.5**	**1.6**		
PDHB	pyruvate dehydrogenase (lipoamide) beta	**-1.4**				
PDHX	pyruvate dehydrogenase complex, component X	**1.2**	**-1.5**			
PDIA3	protein disulfide isomerase family A, member 3	**-1.2**	**-1.3**			
PDIA4	protein disulfide isomerase family A, member 4	**-1.7**	**-1.2**			
PDIA6	protein disulfide isomerase family A, member 6	**-1.7**	**-1.5**	**-1.3**		
PDK1	pyruvate dehydrogenase kinase, isozyme 1	**-5.8**	**2.8**			
PDPK1	3-phosphoinositide dependent protein kinase-1	**2.3**	**-1.8**			
PDXK	pyridoxal (pyridoxine, vitamin B6) kinase	**-4.7**	**2.8**			
PEPD	peptidase D	**-1.3**		**-1.8**		
PER1	period homolog 1 (Drosophila)	**19.4**				
PES1	pescadillo homolog 1, containing BRCT domain (zebrafish)	**-1.6**			**-1.6**	**-1.6**
PEX1	peroxisome biogenesis factor 1	**3.2**		**1.5**		
PEX11B	peroxisomal biogenesis factor 11B	**1.9**	**-1.4**	**1.4**		
PEX14	peroxisomal biogenesis factor 14	**-1.3**	**1.3**			**-1.7**
PEX3	peroxisomal biogenesis factor 3	**-2.3**	**1.3**	**-2.4**		
PEX5	peroxisomal biogenesis factor 5	**-1.3**			**-1.3**	**-1.6**
PFAS	phosphoribosylformylglycinamidine synthase (FGAR amidotransferase)	**-2.3**	**-2.4**	**-2.7**		
PFKFB2	6-phosphofructo-2-kinase/fructose-2,6-biphosphatase 2	**2.4**				
PFKP	phosphofructokinase, platelet	**-1.6**	**1.5**	**1.7**		
PFN1	profilin 1	**1.8**	**-2.1**			
PGAM1	phosphoglycerate mutase 1 (brain)	**-2.4**	**1.5**	**-1.2**	**-1.2**	
PGD	phosphogluconate dehydrogenase	**-2.1**	**1.8**	**-1.3**		
PGGT1B	protein geranylgeranyltransferase type I, beta subunit	**3.9**	**-3.2**			
PGK1	phosphoglycerate kinase 1	**-2.6**	**1.3**	**-1.9**		
PGM1	phosphoglucomutase 1	**-3.2**	**1.7**	**-1.8**		
PGRMC1	progesterone receptor membrane component 1	**-1.8**		**-1.5**		
PGRMC2	progesterone receptor membrane component 2	**-2.6**	**1.7**		**-1.2**	
PHB	prohibitin	**-1.9**				
PHB2	prohibitin 2	**1.5**	**-1.7**			
PHC2	polyhomeotic homolog 2 (Drosophila)	**1.7**	**-1.6**			
PHF1	PHD finger protein 1	**-1.6**	**1.8**			
PHF21A	PHD finger protein 21A	**-1.5**	**2.0**			
PHGDH	phosphoglycerate dehydrogenase	**-2.3**	**-1.7**	**-2.4**		
PHKA1	phosphorylase kinase, alpha 1 (muscle)	**-4.3**	**3.0**			
PHKB	phosphorylase kinase, beta	**2.0**	**-1.7**	**1.4**		
PIAS1	protein inhibitor of activated STAT, 1	**2.2**	**1.7**	**1.4**		
PIAS2	protein inhibitor of activated STAT, 2	**-1.9**		**-1.7**		
PICALM	phosphatidylinositol binding clathrin assembly protein	**2.1**	**2.1**	**2.0**	**1.7**	**1.5**

PIG8	centrosomal protein 57kDa	**-2.3**	**1.6**			
PIGB	phosphatidylinositol glycan anchor biosynthesis, class B	**-1.6**				
PIGC	phosphatidylinositol glycan anchor biosynthesis, class C	**-1.3**	**1.9**	**2.5**		
PIK3C2A	phosphoinositide-3-kinase, class 2, alpha polypeptide	**1.4**	**2.5**	**1.7**		
PIK3C2B	phosphoinositide-3-kinase, class 2, beta polypeptide	**-5.3**				
PIK3CA	phosphoinositide-3-kinase, catalytic, alpha polypeptide	**3.6**	**-2.5**	**1.3**		
PIK3CB	phosphoinositide-3-kinase, catalytic, beta polypeptide	**-2.7**	**3.3**	**1.2**		
PIK3R4	phosphoinositide-3-kinase, regulatory subunit 4, p150	**-1.3**	**1.3**			
PIK4CB	phosphatidylinositol 4-kinase, catalytic, beta polypeptide	**-1.2**		**-1.4**		
PIN1	protein (peptidylprolyl cis/trans isomerase) NIMA-interacting 1	**-1.7**		**1.6**		
PINK1	PTEN induced putative kinase 1	**2.2**	**1.3**		**1.6**	
PIP5K1A	phosphatidylinositol-4-phosphate 5-kinase, type I, alpha	**1.3**				
PIP5K2B	phosphatidylinositol-4-phosphate 5-kinase, type II, beta	**1.8**		**-1.6**		
PIP5K3	phosphatidylinositol-3-phosphate/phosphatidylinositol 5-kinase, type III	**1.3**	**-1.6**			
PISD	phosphatidylserine decarboxylase	**-1.5**				
PITPNA	phosphatidylinositol transfer protein, alpha	**2.0**	**-2.0**	**-1.3**		
PITPNB	phosphatidylinositol transfer protein, beta	**-1.3**	**-1.5**			
PITRM1	pitrilysin metallopeptidase 1	**-2.0**	**1.6**		**1.8**	
PKD2	polycystic kidney disease 2 (autosomal dominant)	**2.5**	**-2.0**			
PKIA	protein kinase (cAMP-dependent, catalytic) inhibitor alpha	**7.8**	**-6.9**		**1.5**	**-1.5**
PKN2	protein kinase N2	**1.3**	**1.4**	**1.4**		
PKP4	plakophilin 4	**-2.9**	**2.5**			
PLAG1	pleiomorphic adenoma gene 1	**11.9**	**-5.2**	**1.4**		
PLAGL1	pleiomorphic adenoma gene-like 1	**-13.0**	**15.8**		**1.6**	
PLAGL2	pleiomorphic adenoma gene-like 2	**-1.3**				
PLCB1	phospholipase C, beta 1 (phosphoinositide-specific)	**4.0**	**-7.4**	**-1.5**		
PLCG2	phospholipase C, gamma 2 (phosphatidylinositol-specific)	**-1.5**	**-1.3**	**-1.6**		
PLEKHB2	pleckstrin homology domain containing, family B (evectins) member 2	**-1.5**	**1.3**	**1.3**		
PLEKHC1	pleckstrin homology domain containing, family C (with FERM domain) member 1	**65.9**		**3.1**	**1.6**	
PLEKHE1	PH domain and leucine rich repeat protein phosphatase	**-1.3**	**-1.6**			
PLK1	polo-like kinase 1 (Drosophila)	**2.3**	**-1.6**	**1.5**		
PLK4	polo-like kinase 4 (Drosophila)	**-1.6**		**1.7**		
PLP2	proteolipid protein 2 (colonic epithelium-enriched)	**8.3**	**-3.3**	**1.5**		
PLS1	plastin 1 (I isoform)	**-21.7**	**9.1**	**-3.2**		
PLSCR1	phospholipid scramblase 1	**-1.4**	**1.8**	**1.5**		
PLTP	phospholipid transfer protein	**-30.8**	**13.4**			
PLXNB2	plexin B2	**8.5**	**-3.9**	**1.3**		
PMAIP1	phorbol-12-myristate-13-acetate-induced protein 1	**2.3**	**-3.7**	**1.5**		**-2.1**
PML	promyelocytic leukemia	**1.4**	**1.8**			
PMPCA	peptidase (mitochondrial processing) alpha	**-1.6**		**-1.4**		
PMPCB	peptidase (mitochondrial processing) beta	**1.4**				
PMS1	PMS1 postmeiotic segregation increased 1 (S. cerevisiae)	**-1.7**	**2.5**	**-1.3**		

PMS2L3	postmeiotic segregation increased 2-like 3	**1.7**	**-1.7**			
PMVK	phosphomevalonate kinase	**-1.4**	**-1.3**			
PNN	pinin, desmosome associated protein	**-2.6**	**1.8**	**-1.5**		**-1.5**
PODXL	podocalyxin-like	**-1.4**	**-1.9**	**-2.8**	**-1.3**	
POGZ	pogo transposable element with ZNF domain	**2.6**				
POLA	polymerase (DNA directed), alpha 1	**-2.7**	**-1.5**	**-2.7**		
POLB	polymerase (DNA directed), beta	**-1.4**	**1.3**	**-1.5**		
POLD2	polymerase (DNA directed), delta 2, regulatory subunit 50kDa	**-13.9**	**1.9**	**-1.9**		
POLD3	polymerase (DNA-directed), delta 3, accessory subunit	**-2.2**	**-1.6**	**-1.8**		
POLD4	polymerase (DNA-directed), delta 4	**3.1**				
POLE	polymerase (DNA directed), epsilon	**-3.9**	**-1.4**			
POLE2	polymerase (DNA directed), epsilon 2 (p59 subunit)	**-2.0**	**-1.8**	**-2.1**		
POLE3	polymerase (DNA directed), epsilon 3 (p17 subunit)	**-2.0**	**-1.5**	**-2.0**		
POLG	polymerase (DNA directed), gamma	**1.5**		**1.4**		
POLG2	polymerase (DNA directed), gamma 2, accessory subunit	**-2.6**	**1.7**	**-1.3**		
POLR1C	polymerase (RNA) I polypeptide C, 30kDa	**-2.9**		**-1.7**		
POLR2A	polymerase (RNA) II (DNA directed) polypeptide A, 220kDa	**2.2**	**-2.4**			
POLR2B	polymerase (RNA) II (DNA directed) polypeptide B, 140kDa	**-3.0**	**2.6**			
POLR2D	polymerase (RNA) II (DNA directed) polypeptide D	**-2.0**				**-1.8**
POLR2E	polymerase (RNA) II (DNA directed) polypeptide E, 25kDa	**-1.6**	**-1.5**	**-1.9**		
POLR2F	polymerase (RNA) II (DNA directed) polypeptide F	**-2.3**				
POLR2H	polymerase (RNA) II (DNA directed) polypeptide H	**-2.9**	**1.4**	**-1.9**	**-1.6**	
POLR2I	polymerase (RNA) II (DNA directed) polypeptide I, 14.5kDa	**-1.7**	**-1.6**	**-1.8**		
POLR2L	polymerase (RNA) II (DNA directed) polypeptide L, 7.6kDa	**-2.4**				
POLR3C	polymerase (RNA) III (DNA directed) polypeptide C (62kD)	**-1.4**	**-1.8**			
POLR3F	polymerase (RNA) III (DNA directed) polypeptide F, 39 kDa	**-1.8**				**-1.6**
POLR3G	polymerase (RNA) III (DNA directed) polypeptide G (32kD)	**-3.8**	**1.3**	**-3.0**		
POLRMT	polymerase (RNA) mitochondrial (DNA directed)	**-1.8**				
POLS	polymerase (DNA directed) sigma	**1.4**				
PON2	paraoxonase 2	**-40.8**	**43.3**	**19.7**	**1.4**	
POP7	processing of precursor 7, ribonuclease P subunit (S. cerevisiae)	**-3.0**		**-2.0**		
POU2AF1	POU domain, class 2, associating factor 1	**25.8**	**-56.3**			**-1.9**
POU2F1	POU domain, class 2, transcription factor 1	**-1.7**		**-2.0**		
PPAP2A	phosphatidic acid phosphatase type 2A	**2.0**				
PPARBP	PPAR binding protein	**-2.0**	**1.8**	**-1.5**		
PPAT	phosphoribosyl pyrophosphate amidotransferase	**-4.7**	**2.0**	**-2.0**	**-1.7**	
PPFIA1	protein tyrosine phosphatase, receptor type, f polypeptide (PTPRF), interacting protein (liprin), alpha 1	**-1.4**	**1.3**			
PPIA	peptidylprolyl isomerase A (cyclophilin A)	**-2.8**	**1.9**	**-1.2**		
PPIB	peptidylprolyl isomerase B (cyclophilin B)	**-1.7**	**1.5**			
PPID	peptidylprolyl isomerase D (cyclophilin D)	**-1.6**				
PPIE	peptidylprolyl isomerase E (cyclophilin E)	**-1.6**				
PPIF	peptidylprolyl isomerase F (cyclophilin F)	**-4.9**	**-1.3**	**-2.6**		

PPIG	peptidylprolyl isomerase G (cyclophilin G)	-2.7	1.8	-1.3		
PPIH	peptidylprolyl isomerase H (cyclophilin H)	-2.0		-1.7		
PPM1A	protein phosphatase 1A (formerly 2C), magnesium-dependent, alpha isoform	1.9	-1.7	1.5		
PPM1B	protein phosphatase 1B (formerly 2C), magnesium-dependent, beta isoform	1.7	1.3	1.4		
PPM1D	protein phosphatase 1D magnesium-dependent, delta isoform	-1.4				
PPM1G	protein phosphatase 1G (formerly 2C), magnesium-dependent, gamma isoform	-2.2				
PPP1CA	protein phosphatase 1, catalytic subunit, alpha isoform	1.5	-2.1			
PPP1CC	protein phosphatase 1, catalytic subunit, gamma isoform	-1.5				
PPP1R11	protein phosphatase 1, regulatory (inhibitor) subunit 11	1.6	-1.6			
PPP1R12A	protein phosphatase 1, regulatory (inhibitor) subunit 12A	1.8	1.4	1.7		
PPP1R2	protein phosphatase 1, regulatory (inhibitor) subunit 2	1.4	2.1	1.4		
PPP1R7	protein phosphatase 1, regulatory subunit 7	2.0	-2.5			
PPP2R2A	protein phosphatase 2 (formerly 2A), regulatory subunit B (PR 52), alpha isoform	-1.9	2.4	1.7		
PPP2R5A	protein phosphatase 2, regulatory subunit B (B56), alpha isoform	1.3	-1.2	1.9		
PPP2R5C	protein phosphatase 2, regulatory subunit B (B56), gamma isoform	5.3	-25.3			
PPP2R5E	protein phosphatase 2, regulatory subunit B (B56), epsilon isoform	-4.4	2.4	1.2		
PPP3CB	protein phosphatase 3 (formerly 2B), catalytic subunit, beta isoform (calcineurin A beta)	-1.8	1.7	1.6		
PPP3CC	protein phosphatase 3 (formerly 2B), catalytic subunit, gamma isoform (calcineurin A gamma)	1.8		1.8		
PPP4C	protein phosphatase 4 (formerly X), catalytic subunit	1.8	-1.8			
PPP4R1	protein phosphatase 4, regulatory subunit 1	1.5				
PPP6C	protein phosphatase 6, catalytic subunit	1.3	-1.8			
PPRC1	peroxisome proliferator-activated receptor gamma, coactivator-related 1	-4.7	1.7	-2.4		
PPT1	palmitoyl-protein thioesterase 1 (ceroid-lipofuscinosis, neuronal 1, infantile)	-2.2	1.7	-1.3		
PPT2	palmitoyl-protein thioesterase 2	-2.3				
PQBP1	polyglutamine binding protein 1	-1.5	1.3	1.3		
PRDM2	PR domain containing 2, with ZNF domain	1.9				
PRDX2	peroxiredoxin 2	3.4	-8.5	-3.1		
PRDX3	peroxiredoxin 3	-1.8		-1.8		-1.6
PRDX4	peroxiredoxin 4	-3.3	1.3	-2.5	-1.2	
PRDX6	peroxiredoxin 6	-1.3	1.5	1.4		
PREI3	preimplantation protein 3	-1.7	1.5	1.3		
PREP	prolyl endopeptidase	-1.8	1.5			
PRG1	proteoglycan 1, secretory granule	2.7	2.7	1.5		3.2
PRIM1	primase, polypeptide 1, 49kDa	-2.3	-1.3	-1.8		
PRIM2A	primase, polypeptide 2A, 58kDa	-1.4	1.4			
PRKAB2	protein kinase, AMP-activated, beta 2 non-catalytic subunit	2.3	5.1			
PRKACB	protein kinase, cAMP-dependent, catalytic, beta	-1.6	2.4	-2.7		
PRKAR2B	protein kinase, cAMP-dependent, regulatory, type II, beta	1.9			1.4	
PRKCA	protein kinase C, alpha	-7.0	6.6	1.3	1.6	
PRKCB1	protein kinase C, beta 1	-18.3	20.7	1.6		
PRKCBP1	protein kinase C binding protein 1	1.6	-1.2	1.4		
PRKCH	protein kinase C, eta	-2.4	3.4	3.7		

PRKCI	protein kinase C, iota	**3.5**				**-1.4**
PRKCQ	protein kinase C, theta	**-2.4**	**1.6**			
PRKD2	protein kinase D2	**4.8**	**-3.2**	**1.8**		
PRKDC	protein kinase, DNA-activated, catalytic polypeptide	**-2.1**	**-1.2**	**-1.6**		
PRKRA	protein kinase, interferon-inducible double stranded RNA dependent activator	**5.0**	**-1.9**	**2.5**		
PRKRIR	protein-kinase, interferon-inducible double stranded RNA dependent inhibitor, repressor of (P58 repressor)	**-1.4**		**-1.6**	**-1.2**	
PRKX	protein kinase, X-linked	**5.3**	**-2.3**	**3.4**		
PRMT3	protein arginine methyltransferase 3	**-2.8**	**-1.3**	**-2.4**	**-1.6**	**-2.1**
PRNPIP	prion protein interacting protein	**-2.5**				
PROCR	protein C receptor, endothelial (EPCR)	**-12.1**	**6.6**	**-1.5**		**-2.2**
PROSC	proline synthetase co-transcribed homolog (bacterial)	**-1.5**				
PRPF19	PRP19/PSO4 pre-mRNA processing factor 19 homolog (S. cerevisiae)	**-2.2**	**-1.3**	**-1.9**		
PRPF31	PRP31 pre-mRNA processing factor 31 homolog (S. cerevisiae)	**-2.1**	**-1.2**			**-1.6**
PRPF4	PRP4 pre-mRNA processing factor 4 homolog (yeast)	**-2.1**	**-1.3**	**-2.2**		
PRPF4B	PRP4 pre-mRNA processing factor 4 homolog B (yeast)	**-1.3**				
PRPS1	phosphoribosyl pyrophosphate synthetase 1	**-2.3**	**-2.1**	**-2.6**	**-1.5**	
PRPS2	phosphoribosyl pyrophosphate synthetase 2	**-2.1**	**1.3**			
PRPSAP1	phosphoribosyl pyrophosphate synthetase-associated protein 1	**-1.3**	**1.9**			
PRSS25	HtrA serine peptidase 2	**-1.9**	**1.5**	**-1.5**		
PSCD1	pleckstrin homology, Sec7 and coiled-coil domains 1(cytohesin 1)	**1.9**				
PSCD2	pleckstrin homology, Sec7 and coiled-coil domains 2 (cytohesin-2)	**1.8**		**1.4**		
PSEN1	presenilin 1 (Alzheimer disease 3)	**2.0**	**1.3**	**1.5**	**1.7**	**1.5**
PSF1	GINS complex subunit 1 (Psf1 homolog)	**-2.3**	**-1.9**	**-1.6**		
PSIP1	PC4 and SFRS1 interacting protein 1	**2.5**	**-2.0**	**-1.5**		
PSMA1	proteasome (prosome, macropain) subunit, alpha type, 1	**-1.3**	**-1.2**			
PSMA2	proteasome (prosome, macropain) subunit, alpha type, 2	**-5.0**	**3.5**	**-1.3**		
PSMA3	proteasome (prosome, macropain) subunit, alpha type, 3	**-1.7**	**1.3**	**-1.7**		
PSMA4	proteasome (prosome, macropain) subunit, alpha type, 4	**-1.5**		**-1.3**		
PSMA5	proteasome (prosome, macropain) subunit, alpha type, 5	**-2.3**		**-1.4**		**-1.4**
PSMA6	proteasome (prosome, macropain) subunit, alpha type, 6	**1.3**	**-1.3**			
PSMB1	proteasome (prosome, macropain) subunit, beta type, 1	**-1.5**				
PSMB2	proteasome (prosome, macropain) subunit, beta type, 2	**-1.5**	**-1.2**	**-1.6**		
PSMB3	proteasome (prosome, macropain) subunit, beta type, 3	**-1.8**		**-1.2**		
PSMB4	proteasome (prosome, macropain) subunit, beta type, 4	**-1.2**	**1.3**	**-1.4**		
PSMB5	proteasome (prosome, macropain) subunit, beta type, 5	**-2.2**	**1.5**	**-1.4**	**-1.4**	**-1.4**
PSMB7	proteasome (prosome, macropain) subunit, beta type, 7	**-1.8**				
PSMB8	proteasome (prosome, macropain) subunit, beta type, 8 (large multifunctional peptidase 7)	**16.2**	**-18.9**			
PSMB9	proteasome (prosome, macropain) subunit, beta type, 9 (large multifunctional peptidase 2)	**22.2**	**-202.7**	**-1.3**		
PSMC2	proteasome (prosome, macropain) 26S subunit, ATPase, 2	**-1.5**				
PSMC3	proteasome (prosome, macropain) 26S subunit, ATPase, 3	**-1.5**	**-1.4**			
PSMC4	proteasome (prosome, macropain) 26S subunit, ATPase, 4	**-2.9**		**-1.3**		
PSMC5	proteasome (prosome, macropain) 26S subunit, ATPase, 5	**-2.6**	**1.6**			

PSMC6	proteasome (prosome, macropain) 26S subunit, ATPase, 6	**-1.4**	**1.3**			
PSMD10	proteasome (prosome, macropain) 26S subunit, non-ATPase, 10	**-1.2**	**-1.2**			
PSMD11	proteasome (prosome, macropain) 26S subunit, non-ATPase, 11	**-1.6**				
PSMD12	proteasome (prosome, macropain) 26S subunit, non-ATPase, 12	**-2.2**	**1.6**			
PSMD13	proteasome (prosome, macropain) 26S subunit, non-ATPase, 13	**-1.9**				
PSMD14	proteasome (prosome, macropain) 26S subunit, non-ATPase, 14	**-2.4**	**1.3**			
PSMD2	proteasome (prosome, macropain) 26S subunit, non-ATPase, 2	**-1.8**	**1.3**			
PSMD3	proteasome (prosome, macropain) 26S subunit, non-ATPase, 3	**-2.0**		**-1.5**		
PSMD4	proteasome (prosome, macropain) 26S subunit, non-ATPase, 4	**-1.6**	**1.3**			
PSMD5	proteasome (prosome, macropain) 26S subunit, non-ATPase, 5	**-2.5**	**3.0**			
PSME3	proteasome (prosome, macropain) activator subunit 3 (PA28 gamma; Ki)	**-2.6**	**1.5**	**-2.2**		**-1.4**
PSPH	phosphoserine phosphatase	**-8.6**	**3.3**	**-2.0**		
PTBP1	polypyrimidine tract binding protein 1	**-2.1**		**-1.5**		
PTEN	phosphatase and tensin homolog (mutated in multiple advanced cancers 1)	**-4.7**	**4.2**	**-1.3**	**2.3**	
PTK2B	PTK2B protein tyrosine kinase 2 beta	**1.9**	**-1.3**		**2.1**	**4.2**
PTP4A1	protein tyrosine phosphatase type IVA, member 1	**-1.9**	**-1.7**	**-1.8**		
PTP4A2	protein tyrosine phosphatase type IVA, member 2	**-1.9**	**1.2**	**1.5**		
PTPLB	protein tyrosine phosphatase-like (proline instead of catalytic arginine), member b	**2.2**	**2.2**	**2.3**		
PTPN1	protein tyrosine phosphatase, non-receptor type 1	**3.0**				**1.6**
PTPN11	protein tyrosine phosphatase, non-receptor type 11 (Noonan syndrome 1)	**-1.3**	**1.3**			
PTPN12	protein tyrosine phosphatase, non-receptor type 12	**75.8**	**-10.9**	**2.4**	**1.3**	
PTPN18	protein tyrosine phosphatase, non-receptor type 18 (brain-derived)	**1.4**			**1.4**	
PTPN2	protein tyrosine phosphatase, non-receptor type 2	**-1.6**	**1.3**	**-1.2**	**-1.3**	**-2.1**
PTPN22	protein tyrosine phosphatase, non-receptor type 22 (lymphoid)	**-8.4**	**2.9**			
PTPN3	protein tyrosine phosphatase, non-receptor type 3	**15.7**				
PTPN6	protein tyrosine phosphatase, non-receptor type 6	**7.8**	**-5.5**		**1.3**	
PTPN7	protein tyrosine phosphatase, non-receptor type 7	**-4.7**	**3.3**	**-1.5**		
PTPN9	protein tyrosine phosphatase, non-receptor type 9	**2.9**				
PTPRC	protein tyrosine phosphatase, receptor type, C	**11.4**	**-6.4**			
PTPRCAP	protein tyrosine phosphatase, receptor type, C-associated protein	**3.6**	**-2.1**	**1.5**		
PTPRF	protein tyrosine phosphatase, receptor type, F	**-122.2**	**91.0**			
PTPRM	protein tyrosine phosphatase, receptor type, M	**-19.3**	**8.5**		**3.8**	**13.6**
PTS	6-pyruvoyltetrahydropterin synthase	**1.8**	**-2.4**	**-1.3**	**-1.6**	
PTTG1	pituitary tumor-transforming 1	**1.4**	**-2.1**			
PUM2	pumilio homolog 2 (Drosophila)	**1.4**	**-1.4**			
PURA	purine-rich element binding protein A	**1.3**	**-1.3**			
PXMP3	peroxisomal membrane protein 3, 35kDa (Zellweger syndrome)	**2.6**	**-1.7**	**1.7**		**4.3**
QDPR	quinoid dihydropteridine reductase	**-3.8**	**2.2**			
QKI	quaking homolog, KH domain RNA binding (mouse)	**-1.7**	**2.7**	**2.5**		**-1.5**
QP-C	ubiquinol-cytochrome c reductase, complex III subunit VII, 9.5kDa	**-1.4**		**-1.2**		
QPRT	quinolinate phosphoribosyltransferase (nicotinate-nucleotide pyrophosphorylase (carboxylating))	**1.4**	**-2.1**	**-3.6**		
RAB11A	RAB11A, member RAS oncogene family	**1.5**	**-2.2**	**1.3**		

RAB1A	RAB1A, member RAS oncogene family	**-1.3**	**1.5**	**1.3**		
RAB2	RAB2, member RAS oncogene family	**2.1**	**-1.9**			
RAB21	RAB21, member RAS oncogene family	**1.3**		**1.5**		
RAB22A	RAB22A, member RAS oncogene family	**2.1**				
RAB27A	RAB27A, member RAS oncogene family	**-3.5**	**2.2**	**-1.8**		
RAB31	RAB31, member RAS oncogene family	**-13.0**	**3.8**			
RAB40B	RAB40B, member RAS oncogene family	**-1.5**	**-1.7**		**-1.3**	
RAB4A	RAB4A, member RAS oncogene family	**-4.7**	**3.8**			
RAB6A	RAB6A, member RAS oncogene family	**1.7**	**-1.4**	**1.5**		
RAB6IP2	ELKS/RAB6-interacting/CAST family member 1	**5.3**	**-1.3**		**1.8**	
RAB7L1	RAB7, member RAS oncogene family-like 1	**-2.9**	**2.1**	**-1.4**		
RAB9P40	Rab9 effector protein with kelch motifs	**-12.1**	**1.9**	**-3.7**	**-1.8**	**-2.1**
RABAC1	Rab acceptor 1 (prenylated)	**3.1**	**-1.5**			
RABEP1	rabaptin, RAB GTPase binding effector protein 1	**2.4**	**-2.1**			
RABGGTA	Rab geranylgeranyltransferase, alpha subunit	**-1.5**		**-1.2**		
RABGGTB	Rab geranylgeranyltransferase, beta subunit	**-1.8**	**2.1**	**-1.8**	**-1.3**	**-1.7**
RABIF	RAB interacting factor	**1.5**	**-1.2**			
RAC1	ras-related C3 botulinum toxin substrate 1 (rho family, small GTP binding protein Rac1)	**2.0**	**-1.9**	**1.4**		
RAC2	ras-related C3 botulinum toxin substrate 2 (rho family, small GTP binding protein Rac2)	**1.3**	**-2.7**			
RAC3	ras-related C3 botulinum toxin substrate 3 (rho family, small GTP binding protein Rac3)	**-17.1**				
RAD1	RAD1 homolog (S. pombe)	**-3.2**	**-1.6**	**-1.7**		
RAD17	RAD17 homolog (S. pombe)	**-1.2**		**-2.1**		
RAD21	RAD21 homolog (S. pombe)	**2.5**	**-2.3**	**1.3**		
RAD51AP1	RAD51 associated protein 1	**-1.3**	**-2.8**	**-1.4**		
RAD51C	RAD51 homolog C (S. cerevisiae)	**-1.9**		**-1.9**		
RAD54L	RAD54-like (S. cerevisiae)	**-1.9**		**-1.5**		
RAE1	RAE1 RNA export 1 homolog (S. pombe)	**-1.4**	**-1.3**	**-1.4**		
RAG1	recombination activating gene 1	**14.5**	**-32.2**		**-3.6**	**-4.2**
RAG2	recombination activating gene 2	**6.4**	**-7.4**			
RAI17	zinc finger, MIZ-type containing 1	**-1.6**		**-1.3**		
RALA	v-ral simian leukemia viral oncogene homolog A (ras related)	**-2.1**	**1.7**	**-1.3**		
RALBP1	ralA binding protein 1	**1.2**	**-1.8**	**-1.4**		
RALY	RNA binding protein, autoantigenic (hnRNP-associated with lethal yellow homolog (mouse))	**1.2**	**-1.4**			
RAN	RAN, member RAS oncogene family	**-1.4**	**-1.2**	**-1.2**		
RANBP1	RAN binding protein 1	**-2.8**	**-1.9**			
RANBP2	RAN binding protein 2	**-1.9**	**2.5**	**-1.4**		
RANBP5	RAN binding protein 5	**-2.3**	**-1.3**	**-2.0**		**-1.5**
RANBP9	RAN binding protein 9	**2.1**	**-1.6**	**-1.3**		
RAP1A	RAP1A, member of RAS oncogene family	**-2.2**	**2.0**			
RAP1GDS1	RAP1, GTP-GDP dissociation stimulator 1	**2.0**	**-1.5**			
RAP2A	RAP2A, member of RAS oncogene family	**4.0**	**-1.4**	**1.6**		
RAPGEF2	Rap guanine nucleotide exchange factor (GEF) 2	**4.2**	**1.8**	**2.0**		

RARS	arginyl-tRNA synthetase	-1.9			-1.2	
RASA1	RAS p21 protein activator (GTPase activating protein) 1	3.5	1.9	2.3	1.9	2.0
RASA4	RAS p21 protein activator 4	-3.4	2.0			
RASGRP2	RAS guanyl releasing protein 2 (calcium and DAG-regulated)	4.4	-2.3	2.1		
RASSF2	Ras association (RalGDS/AF-6) domain family 2	3.5	-6.4	-2.5		
RB1	retinoblastoma 1 (including osteosarcoma)	3.1	-2.6	1.5		
RB1CC1	RB1-inducible coiled-coil 1	1.7	-1.4	1.7		
RBBP4	retinoblastoma binding protein 4	-1.5	-1.3	-1.4		
RBBP5	retinoblastoma binding protein 5	1.3	-1.3			
RBBP6	retinoblastoma binding protein 6	2.5	-1.8			
RBBP8	retinoblastoma binding protein 8	-1.5	-1.9	-1.7	-1.3	
RBL2	retinoblastoma-like 2 (p130)	-1.3	1.5	1.5		
RBM13	RNA binding motif protein 13	-2.1		-1.8	-1.6	-1.3
RBM16	RNA binding motif protein 16	1.2				
RBM5	RNA binding motif protein 5	2.2	1.6		1.4	1.3
RBM8A	RNA binding motif protein 8A	-3.3	2.3	1.5		
RBMS1	RNA binding motif, single stranded interacting protein 1	2.3	3.0	4.3		
RBMS2	RNA binding motif, single stranded interacting protein 2	1.4				
RBMX	RNA binding motif protein, X-linked	-1.9	1.4			
RBPSUH	recombining binding protein suppressor of hairless (Drosophila)	1.2	1.9	1.4		
RCHY1	ring finger and CHY zinc finger domain containing 1	-1.6	1.5			
RCN1	reticulocalbin 1, EF-hand calcium binding domain	2.3	-1.5	1.2	1.5	
RCOR1	REST corepressor 1	1.3	-1.3			
RDBP	RD RNA binding protein	-1.6	-1.2			
RDH11	retinol dehydrogenase 11 (all-trans/9-cis/11-cis)	-1.6	-1.3	-1.8		
RDX	radixin	2.5			1.6	
REC14	WD repeat domain 61	-1.4				
RECK	reversion-inducing-cysteine-rich protein with kazal motifs	5.2		2.9		
RECQL	RecQ protein-like (DNA helicase Q1-like)	1.4	-1.4		1.6	
REL	v-rel reticuloendotheliosis viral oncogene homolog (avian)	-2.1	2.4	1.5		2.4
REV3L	REV3-like, catalytic subunit of DNA polymerase zeta (yeast)	2.2	-2.1	1.5		
RFC1	replication factor C (activator 1) 1, 145kDa	-2.1	-1.2	-1.4	1.5	
RFC2	replication factor C (activator 1) 2, 40kDa	-1.5	-3.9	-1.5		
RFC3	replication factor C (activator 1) 3, 38kDa	-1.5	-2.1	-1.5		
RFC4	replication factor C (activator 1) 4, 37kDa	-4.2		-1.6		-1.7
RFC5	replication factor C (activator 1) 5, 36.5kDa	-2.9	-1.3	-1.7		
RFK	riboflavin kinase	-1.3	-1.9	-2.1		
RFP	tripartite motif-containing 27	-1.8		-1.5		
RFX5	regulatory factor X, 5 (influences HLA class II expression)	-1.6	-1.6			
RGS10	regulator of G-protein signalling 10	-19.8	19.3	-2.5		
RGS19	regulator of G-protein signalling 19	2.2	-1.8	-1.3	-1.3	-1.6
RHEB	Ras homolog enriched in brain	2.9	-1.6	1.9		

RHOA	ras homolog gene family, member A	1.4	-1.4	1.4	1.2	
RHOC	ras homolog gene family, member C	2.3	3.0	3.2		
RHOG	ras homolog gene family, member G (rho G)	-1.6	1.9			
RHOH	ras homolog gene family, member H	38.1	-15.8	1.3		
RHOQ	ras homolog gene family, member Q	-1.7	1.8	1.3		
RIF1	RAP1 interacting factor homolog (yeast)	-6.8	2.0	-1.3		
RIMS3	regulating synaptic membrane exocytosis 3	12.1	-6.8			
RIOK3	RIO kinase 3 (yeast)	1.5	1.7			
RIT1	Ras-like without CAAX 1	2.1	-1.4	1.8		
RNASEH1	ribonuclease H1	-1.9		-1.3		
RNF103	ring finger protein 103	1.7		1.5	1.3	
RNF11	ring finger protein 11	1.5		1.5		
RNF14	ring finger protein 14	2.3		1.4		
RNF144	ring finger protein 144	-4.3	3.5			
RNF4	ring finger protein 4	-1.3				
RNF5	ring finger protein 5	-1.5				
RNF6	ring finger protein (C3H2C3 type) 6	2.2	-2.2	1.2		
RNH	ribonuclease/angiogenin inhibitor 1	-4.2	2.4	-1.5		-1.3
RNMT	RNA (guanine-7-) methyltransferase	-1.5	1.3			
RNPC2	RNA binding motif protein 39	1.3	1.8	2.3		
RNPS1	RNA binding protein S1, serine-rich domain	2.5	-5.0	-1.2		
RNUT1	snurportin 1	-1.5	1.3			
ROCK1	Rho-associated, coiled-coil containing protein kinase 1	1.8				
ROD1	ROD1 regulator of differentiation 1 (S. pombe)	1.9				
RPA1	replication protein A1, 70kDa	1.5	-2.8	-1.6		
RPA2	replication protein A2, 32kDa	-1.5		-1.4		
RPE	ribulose-5-phosphate-3-epimerase	1.4	-2.1	-1.5		
RPIA	ribose 5-phosphate isomerase A (ribose 5-phosphate epimerase)	-2.7		-1.7		
RPL13	ribosomal protein L13	-3.0	1.4			
RPL13A	ribosomal protein L13a	-1.2	1.4			
RPL15	ribosomal protein L15	-1.4	1.5	2.1		
RPL17	ribosomal protein L17	-2.3	2.1	-1.8		
RPL29	ribosomal protein L29	-1.5	2.0	1.4		
RPL31	ribosomal protein L31	-3.4	2.9	1.3		
RPL35	ribosomal protein L35	-1.8	2.2	2.4		
RPL37	ribosomal protein L37	-1.8	1.6	-1.5		
RPL37A	ribosomal protein L37a	-2.2				
RPL38	ribosomal protein L38	-1.5	2.0			
RPLP2	ribosomal protein, large, P2	-1.7	1.4			
RPN1	ribophorin I	-2.3	1.5			
RPN2	ribophorin II	-2.6	1.7	-1.5		
RPP14	ribonuclease P 14kDa subunit	-2.7	1.9	-1.7		

RPP30	ribonuclease P/MRP 30kDa subunit	**-1.6**		**-1.6**		
RPP38	ribonuclease P/MRP 38kDa subunit	**-1.8**	**1.7**	**-1.5**	**-1.2**	
RPP40	ribonuclease P 40kDa subunit	**-2.3**		**-1.9**	**-1.9**	
RPS15A	ribosomal protein S15a	**1.2**	**1.5**			
RPS2	ribosomal protein S2	**-1.9**	**2.6**			
RPS21	ribosomal protein S21	**-3.3**	**3.1**	**1.2**		
RPS23	ribosomal protein S23	**-2.1**	**2.0**			
RPS24	ribosomal protein S24	**-3.2**	**2.2**			
RPS27	ribosomal protein S27 (metallopanstimulin 1)	**-1.7**				
RPS4X	ribosomal protein S4, X-linked	**1.5**				
RPS6	ribosomal protein S6	**1.3**	**-1.7**	**-1.9**		
RPS6KA1	ribosomal protein S6 kinase, 90kDa, polypeptide 1	**3.6**	**-2.9**	**-1.3**		
RPS6KA3	ribosomal protein S6 kinase, 90kDa, polypeptide 3	**-1.7**		**-1.5**		
RPS6KB1	ribosomal protein S6 kinase, 70kDa, polypeptide 1	**-1.7**	**1.7**			
RPS9	ribosomal protein S9	**2.6**		**2.5**		
RQCD1	RCD1 required for cell differentiation1 homolog (S. pombe)	**-1.4**	**-2.3**	**-1.7**		
RRAGA	Ras-related GTP binding A	**3.2**	**-2.6**	**1.3**	**1.4**	
RRAGD	Ras-related GTP binding D	**-8.9**	**3.0**	**-2.4**		
RRAS	related RAS viral (r-ras) oncogene homolog	**2.4**	**1.7**	**2.6**		
RRM1	ribonucleotide reductase M1 polypeptide	**-2.1**	**-1.6**	**-1.4**		
RRM2	ribonucleotide reductase M2 polypeptide	**-1.8**	**-2.3**			
RRS1	RRS1 ribosome biogenesis regulator homolog (S. cerevisiae)	**-1.6**	**-1.4**			
RSN	CAP-GLY domain containing linker protein 1	**7.4**				
RSU1	Ras suppressor protein 1	**-1.8**				
RTN4	reticulon 4	**2.4**		**2.3**		
RUNX1	--	**-1.4**	**-1.5**	**-2.5**	**-1.3**	
RUVBL1	RuvB-like 1 (E. coli)	**-1.7**	**-1.6**			
RUVBL2	RuvB-like 2 (E. coli)	**-2.0**	**1.2**	**-1.4**		
RXRA	retinoid X receptor, alpha	**-3.3**	**2.4**			
RYBP	RING1 and YY1 binding protein	**2.9**	**1.4**	**1.3**		
RYK	RYK receptor-like tyrosine kinase	**-22.2**	**11.1**			
S100A10	S100 calcium binding protein A10	**40.6**	**-26.4**			
S100A4	S100 calcium binding protein A4	**5.4**	**-7.7**	**-4.0**		
SACM1L	SAC1 suppressor of actin mutations 1-like (yeast)	**-1.6**	**1.8**			
SACS	spastic ataxia of Charlevoix-Saguenay (sacsin)	**-1.4**	**-1.7**	**-1.4**		**-2.0**
SAFB	scaffold attachment factor B	**-1.3**				
SAP18	Sin3A-associated protein, 18kDa	**1.7**	**-2.2**			
SAP30	Sin3A-associated protein, 30kDa	**3.8**	**1.7**	**2.3**	**2.2**	
SARA1	SAR1 gene homolog A (S. cerevisiae)	**-1.7**	**1.6**			
SARS	seryl-tRNA synthetase	**1.8**	**-2.7**	**-1.4**	**-1.4**	
SART3	squamous cell carcinoma antigen recognized by T cells 3	**-1.9**		**-1.4**		
SAS	tetraspanin 31	**2.0**			**1.8**	

SAT	spermidine/spermine N1-acetyltransferase 1	1.3	5.5	3.9		
SATB1	special AT-rich sequence binding protein 1 (binds to nuclear matrix/scaffold-associating DNA's)	-3.7	6.4	-1.8	-1.9	-2.3
SATB2	SATB family member 2	2.8			-1.5	
SBF1	SET binding factor 1	6.9	-1.9	3.1		
SC4MOL	sterol-C4-methyl oxidase-like	4.6	-3.2	1.6		-1.6
SC5DL	sterol-C5-desaturase (ERG3 delta-5-desaturase homolog, fungal)-like	2.6	-1.7			
SCAMP1	secretory carrier membrane protein 1	-1.7	1.7			
SCAMP3	secretory carrier membrane protein 3	-1.4				
SCAMP5	secretory carrier membrane protein 5	-2.6	4.4			
SCAP	SREBF chaperone	-1.4	1.2			
SCARB1	scavenger receptor class B, member 1	-33.3	-1.4	-4.9	-1.7	
SCC-112	SCC-112 protein	1.5	-1.4	1.3		
SCD	stearoyl-CoA desaturase (delta-9-desaturase)	-3.6		-3.7		
SCFD1	sec1 family domain containing 1	1.9		2.2		
SCHIP1	schwannomin interacting protein 1	1.9	-5.1	1.4		
SCP2	sterol carrier protein 2	175.1	-12.0	1.6		
SCYE1	small inducible cytokine subfamily E, member 1 (endothelial monocyte-activating)	-2.0	1.6	-1.4	-1.7	-1.9
SDC1	syndecan 1	-14.8				
SDCBP	syndecan binding protein (syntenin)	2.5	-1.5	1.5	1.8	
SDFR1	neuroplastin	-1.5	1.5			
SDHA	succinate dehydrogenase complex, subunit A, flavoprotein (Fp)	1.9	-1.5	2.0		
SDHB	succinate dehydrogenase complex, subunit B, iron sulfur (Ip)	-1.9		-1.4		
SDHC	succinate dehydrogenase complex, subunit C, integral membrane protein, 15kDa	1.2	-1.3			
SEC14L1	SEC14-like 1 (S. cerevisiae)	-1.9		24.5		
SEC22L1	SEC22 vesicle trafficking protein homolog B (S. cerevisiae)	4.1	-1.9			
SEC23A	Sec23 homolog A (S. cerevisiae)	1.8		1.9		
SEC23IP	SEC23 interacting protein	-1.6	1.4			
SEC24A	SEC24 related gene family, member A (S. cerevisiae)	1.5				
SEC24B	SEC24 related gene family, member B (S. cerevisiae)	1.3				
SEC24C	SEC24 related gene family, member C (S. cerevisiae)	-1.3	1.4			
SEC31L1	SEC31 homolog A (S. cerevisiae)	1.6				
SEC61G	Sec61 gamma subunit	-2.6	2.2			
SEC63	SEC63 homolog (S. cerevisiae)	-2.5	1.5	-1.3		-1.2
SEC8L1	exocyst complex component 4	1.8		1.8		
SEMA4D	sema domain, immunoglobulin domain (Ig), transmembrane domain (TM) and short cytoplasmic domain, (semaphorin) 4D	2.1	-1.5			1.6
SENP6	SUMO1/sentrin specific peptidase 6	1.2	2.0	1.5		
SEPHS1	selenophosphate synthetase 1	-1.7	-1.6	-2.1		
SEPT2	septin 2	2.4	-3.6	-1.5		
SEPT6	septin 6	-2.7	-1.4	-2.0		-1.6
SEPT7	septin 7	-2.3	2.6	1.3		
SEPT8	septin 8	4.5	1.5	-1.4		
SEPT9	septin 9	2.6	-2.8			

SEPW1	selenoprotein W, 1	-1.5	2.4	1.3		
SERP1	stress-associated endoplasmic reticulum protein 1	-1.6	1.5	-1.7		
SERPINB1	serpin peptidase inhibitor, clade B (ovalbumin), member 1	-7.4	40.4	3.1		
SERPINE2	serpin peptidase inhibitor, clade E (nexin, plasminogen activator inhibitor type 1), member 2	-1.3				
SERPINH1	serpin peptidase inhibitor, clade H (heat shock protein 47), member 1, (collagen binding protein 1)	-17.3	18.1			
SERTAD2	SERTA domain containing 2	-1.8	1.5			
SET	SET translocation (myeloid leukemia-associated)	-1.5	-1.3	-1.9		
SF3A3	splicing factor 3a, subunit 3, 60kDa	-1.9		-1.4		-1.6
SF3B2	splicing factor 3b, subunit 2, 145kDa	-1.5				
SF3B3	splicing factor 3b, subunit 3, 130kDa	-2.7	-1.4	-1.7		
SFRS1	splicing factor, arginine/serine-rich 1 (splicing factor 2, alternate splicing factor)	-2.4	-1.6	-1.7		
SFRS10	splicing factor, arginine/serine-rich 10 (transformer 2 homolog, Drosophila)	-1.3	-1.7	-1.2		
SFRS11	splicing factor, arginine/serine-rich 11	-4.1	2.7	4.2		
SFRS12	splicing factor, arginine/serine-rich 12	-3.5	4.5	-1.7		
SFRS2	splicing factor, arginine/serine-rich 2	-1.5	-1.3	-1.4		
SFRS2B	splicing factor, arginine/serine-rich 2B	1.3	-1.3	-1.3		
SFRS3	splicing factor, arginine/serine-rich 3	1.5	-1.4	1.3		
SFRS6	splicing factor, arginine/serine-rich 6	3.4	-3.6			
SFRS7	splicing factor, arginine/serine-rich 7, 35kDa	-1.9	-1.8	-1.5		
SFRS9	splicing factor, arginine/serine-rich 9	2.0	-2.7	1.2		
SGSH	N-sulfoglucosamine sulfohydrolase (sulfamidase)	2.1			1.8	
SH2B	SH2B adaptor protein 1	1.8				
SH3GLB1	SH3-domain GRB2-like endophilin B1	2.5	1.5	2.2		
SHMT1	serine hydroxymethyltransferase 1 (soluble)	1.4	-1.5	-1.8		
SIAH1	seven in absentia homolog 1 (Drosophila)	1.5	-1.5			
SIAHBP1	fuse-binding protein-interacting repressor	-1.4		-1.2		
SIM2	single-minded homolog 2 (Drosophila)	-3.1	2.4			
SIP1	survival of motor neuron protein interacting protein 1	-1.5				
SIVA	SIVA1, apoptosis-inducing factor	-2.9	-1.6	-1.7		
SKI	v-ski sarcoma viral oncogene homolog (avian)	-5.0	7.9			
SKIIP	SNW domain containing 1	1.3				
SKIP (C62)	skeletal muscle and kidney enriched inositol phosphatase	2.3	-1.7			
SKP1A	S-phase kinase-associated protein 1A (p19A)	-1.5				
SLA	Src-like-adaptor	10.8	2.1	4.4	2.8	3.1
SLBP	stem-loop (histone) binding protein	-1.8	-1.8	-1.3		
SLC11A2	solute carrier family 11 (proton-coupled divalent metal ion transporters), member 2	-2.1	2.1	-2.1		
SLC12A2	solute carrier family 12 (sodium/potassium/chloride transporters), member 2	1.9	-1.9			
SLC16A1	solute carrier family 16, member 1 (monocarboxylic acid transporter 1)	-3.9	-1.2	-2.8		
SLC16A3	solute carrier family 16, member 3 (monocarboxylic acid transporter 4)	-28.1	9.6			
SLC16A6	solute carrier family 16, member 6 (monocarboxylic acid transporter 7)	-1.4		-1.6		
SLC18A2	solute carrier family 18 (vesicular monoamine), member 2	-6.1	4.3	1.6	4.1	4.2
SLC19A1	solute carrier family 19 (folate transporter), member 1	-4.6		-1.7	-1.6	-1.7

SLC1A4	solute carrier family 1 (glutamate/neutral amino acid transporter), member 4	**2.2**	**-11.7**	**-1.8**	**1.6**	
SLC20A1	solute carrier family 20 (phosphate transporter), member 1	**-2.7**	**-2.2**	**-1.4**		
SLC23A2	solute carrier family 23 (nucleobase transporters), member 2	**-2.5**	**1.3**	**-1.8**		
SLC25A1	solute carrier family 25 (mitochondrial carrier; citrate transporter), member 1	**-1.4**		**-1.3**		
SLC25A11	solute carrier family 25 (mitochondrial carrier; oxoglutarate carrier), member 11	**1.7**	**-2.4**			
SLC25A12	solute carrier family 25 (mitochondrial carrier, Aralar), member 12	**-1.8**				
SLC25A5	solute carrier family 25 (mitochondrial carrier; adenine nucleotide translocator), member 5	**-1.3**				
SLC29A1	solute carrier family 29 (nucleoside transporters), member 1	**-2.0**		**-2.6**	**-1.7**	
SLC2A3	solute carrier family 2 (facilitated glucose transporter), member 3	**-1.7**	**-1.9**	**6.0**		
SLC30A1	solute carrier family 30 (zinc transporter), member 1	**-1.8**	**2.2**			
SLC30A9	solute carrier family 30 (zinc transporter), member 9	**-1.5**	**2.1**			
SLC31A1	solute carrier family 31 (copper transporters), member 1	**-2.3**		**-1.8**		
SLC35B1	solute carrier family 35, member B1	**-1.4**				
SLC39A14	solute carrier family 39 (zinc transporter), member 14	**-5.4**	**1.9**	**-3.8**	**-1.4**	**-1.3**
SLC39A7	solute carrier family 39 (zinc transporter), member 7	**-1.4**				
SLC39A8	solute carrier family 39 (zinc transporter), member 8	**-3.1**		**-3.1**		
SLC43A1	solute carrier family 43, member 1	**-2.5**	**1.8**	**-2.0**		
SLC6A6	solute carrier family 6 (neurotransmitter transporter, taurine), member 6	**-18.0**	**8.8**			
SLC7A1	solute carrier family 7 (cationic amino acid transporter, y+ system), member 1	**-2.3**	**-2.4**	**-2.7**		**-2.1**
SLC7A5	solute carrier family 7 (cationic amino acid transporter, y+ system), member 5	**1.5**	**-3.9**			
SLC9A3R1	solute carrier family 9 (sodium/hydrogen exchanger), member 3 regulator 1	**-2.3**	**1.6**	**-1.4**		**-1.9**
SLK	STE20-like kinase (yeast)	**-2.9**	**4.3**			
SMAD1	SMAD family member 1	**1.3**	**1.2**	**2.7**		
SMAD2	SMAD family member 2	**1.9**	**-1.7**	**-1.3**		
SMAD4	SMAD family member 4	**2.2**	**-1.5**	**1.6**		**-1.4**
SMAD6	SMAD family member 6	**-6.8**	**5.6**			
SMARCA2	SWI/SNF related, matrix associated, actin dependent regulator of chromatin, subfamily a, member 2	**7.2**	**-1.2**	**2.2**	**1.7**	**1.4**
SMARCA3	helicase-like transcription factor	**-1.4**	**-1.4**	**-1.4**		
SMARCC1	SWI/SNF related, matrix associated, actin dependent regulator of chromatin, subfamily c, member 1	**-2.4**	**-1.4**	**-2.2**		
SMARCC2	SWI/SNF related, matrix associated, actin dependent regulator of chromatin, subfamily c, member 2	**1.2**	**-1.5**			
SMARCD1	SWI/SNF related, matrix associated, actin dependent regulator of chromatin, subfamily d, member 1	**1.7**	**-1.9**			
SMARCE1	SWI/SNF related, matrix associated, actin dependent regulator of chromatin, subfamily e, member 1	**2.0**	**-2.2**			
SMC2L1	structural maintenance of chromosomes 2	**-1.3**	**-2.3**	**1.3**		
SMC4L1	structural maintenance of chromosomes 4	**6.8**	**-1.5**	**1.4**	**1.7**	**1.7**
SMC5L1	structural maintenance of chromosomes 5	**-1.5**				
SMG1	PI-3-kinase-related kinase SMG-1	**1.8**	**-1.8**			
SMN1	survival of motor neuron 1, telomeric	**-1.7**			**-1.6**	
SMNDC1	survival motor neuron domain containing 1	**-1.5**	**1.6**			
SMOX	spermine oxidase	**2.7**		**2.1**		**10.6**
SMPDL3B	sphingomyelin phosphodiesterase, acid-like 3B	**-43.6**				
SMURF2	SMAD specific E3 ubiquitin protein ligase 2	**1.5**	**1.6**	**1.6**		
SNAP23	synaptosomal-associated protein, 23kDa	**1.5**				

SNAPC3	small nuclear RNA activating complex, polypeptide 3, 50kDa	**3.3**	**-3.2**	**1.3**		
SNAPC5	small nuclear RNA activating complex, polypeptide 5, 19kDa	**-1.7**			**1.4**	**-1.5**
SNF1LK	SNF1-like kinase	**2.5**				
SNRP70	small nuclear ribonucleoprotein 70kDa polypeptide (RNP antigen)	**-1.4**		**-1.5**		
SNRPA	small nuclear ribonucleoprotein polypeptide A	**-1.8**		**-1.9**		
SNRPA1	small nuclear ribonucleoprotein polypeptide A'	**-2.4**	**-1.4**	**-1.4**		
SNRPB	small nuclear ribonucleoprotein polypeptides B and B1	**-2.4**	**-1.6**	**-1.7**		
SNRPB2	small nuclear ribonucleoprotein polypeptide B''	**1.7**				
SNRPC	small nuclear ribonucleoprotein polypeptide C	**-1.3**	**-1.3**	**-1.5**		
SNRPD1	small nuclear ribonucleoprotein D1 polypeptide 16kDa	**-2.0**	**-1.9**	**-1.7**	**-1.4**	
SNRPD3	small nuclear ribonucleoprotein D3 polypeptide 18kDa	**-1.4**	**-1.6**			
SNRPG	small nuclear ribonucleoprotein polypeptide G	**-1.8**				
SNRPN	small nuclear ribonucleoprotein polypeptide N	**1.5**		**-1.2**		
SNTB2	syntrophin, beta 2 (dystrophin-associated protein A1, 59kDa, basic component 2)	**8.7**	**-5.9**	**3.5**	**3.0**	**2.3**
SNX1	sorting nexin 1	**-2.4**	**3.6**			
SNX19	sorting nexin 19	**1.7**				
SNX2	sorting nexin 2	**1.4**	**-1.6**	**-1.4**		
SNX4	sorting nexin 4	**1.3**	**-1.2**			**-1.5**
SOCS1	suppressor of cytokine signaling 1	**40.4**	**-1.6**	**8.3**	**15.6**	**29.3**
SOCS2	suppressor of cytokine signaling 2	**4.7**	**-1.8**	**2.8**		**2.8**
SOD1	superoxide dismutase 1, soluble (amyotrophic lateral sclerosis 1 (adult))	**-1.6**				
SORD	sorbitol dehydrogenase	**-2.9**	**-1.3**	**-1.6**		
SOX4	SRY (sex determining region Y)-box 4	**3.0**	**-5.7**	**-1.3**		
SP100	SP100 nuclear antigen	**6.4**	**-1.7**	**2.3**		
SP3	Sp3 transcription factor	**2.2**	**-2.8**			
SPA17	sperm autoantigenic protein 17	**1.8**	**-2.0**			
SPAG11	sperm associated antigen 11	**2.2**				
SPAG9	sperm associated antigen 9	**1.3**	**2.0**	**1.3**		
SPAST	spastin	**-1.2**	**1.3**			
SPBC25	spindle pole body component 25 homolog (S. cerevisiae)	**-3.1**	**1.4**			
SPCS2	signal peptidase complex subunit 2 homolog (S. cerevisiae)	**-1.6**	**1.3**	**-1.3**		**-1.4**
SPEN	spen homolog, transcriptional regulator (Drosophila)	**1.3**		**-1.3**		
SPHAR	S-phase response (cyclin-related)	**-3.2**	**3.4**			
SPHK2	sphingosine kinase 2	**1.6**	**-1.7**			
SPINT2	serine peptidase inhibitor, Kunitz type, 2	**-2.6**	**4.2**		**1.4**	
SPN	sialophorin (leukosialin, CD43)	**1.7**	**-1.9**	**-1.2**		
SPRY1	sprouty homolog 1, antagonist of FGF signaling (Drosophila)	**4.7**		**11.8**		**1.7**
SPTLC1	serine palmitoyltransferase, long chain base subunit 1	**-1.2**	**1.7**			
SQLE	squalene epoxidase	**1.8**	**-2.0**	**-1.4**		
SRD5A1	steroid-5-alpha-reductase, alpha polypeptide 1 (3-oxo-5 alpha-steroid delta 4-dehydrogenase alpha 1)	**4.9**	**2.0**	**1.2**		**3.0**
SRGAP2	SLIT-ROBO Rho GTPase activating protein 2	**1.5**		**2.3**	**1.3**	
SRI	sorcin	**-1.9**	**1.5**			**-1.6**

SRM	spermidine synthase	-4.0		-3.4	-1.8	-1.7
SRP14	signal recognition particle 14kDa (homologous Alu RNA binding protein)	1.5	-1.4			
SRP54	signal recognition particle 54kDa	1.5		1.7		
SRP72	signal recognition particle 72kDa	-5.1	3.8	-1.5		
SRPK1	SFRS protein kinase 1	-1.8		-1.5		-1.7
SRPK2	SFRS protein kinase 2	1.8	-1.3	1.2		
SRPR	signal recognition particle receptor ('docking protein')	-1.2				
SRRM1	serine/arginine repetitive matrix 1	-1.6		-1.5		
SRRM2	serine/arginine repetitive matrix 2	1.4	-1.9	-1.4		
SS18	synovial sarcoma translocation, chromosome 18	-1.5	1.5			
SS18L1	synovial sarcoma translocation gene on chromosome 18-like 1	-2.4	1.6	-1.5		
SSA2	TROVE domain family, member 2	-1.8		1.4		
SSB	Sjogren syndrome antigen B (autoantigen La)	-3.1	1.7	-1.7		-1.6
SSBP1	single-stranded DNA binding protein 1	-1.4	1.3	-1.8		
SSBP2	single-stranded DNA binding protein 2	7.2	-7.6			
SSR1	signal sequence receptor, alpha (translocon-associated protein alpha)	-1.4	1.2			
SSR4	signal sequence receptor, delta (translocon-associated protein delta)	-2.1	1.7	-1.3		
SSRP1	structure specific recognition protein 1	-1.9	-1.8	-2.2		
ST13	suppression of tumorigenicity 13 (colon carcinoma) (Hsp70 interacting protein)	-2.2	1.5	-1.5		
ST3GAL1	ST3 beta-galactoside alpha-2,3-sialyltransferase 1	1.2		1.9		
ST3GAL5	ST3 beta-galactoside alpha-2,3-sialyltransferase 5	-10.8	4.7		1.6	
ST3GAL6	ST3 beta-galactoside alpha-2,3-sialyltransferase 6	-28.6	13.7	1.9	2.1	
ST6GAL1	ST6 beta-galactosamide alpha-2,6-sialyltranferase 1	1.5	-1.7			
ST6GALNAC4	ST6 (alpha-N-acetyl-neuraminyl-2,3-beta-galactosyl-1,3)-N-acetylgalactosaminide alpha-2,6-sialyltransferase 4	1.7	-1.7			1.4
ST7	suppression of tumorigenicity 7	-2.0				
STAG1	stromal antigen 1	1.2	-1.4			
STAG2	stromal antigen 2	1.7	-1.6			
STAM	signal transducing adaptor molecule (SH3 domain and ITAM motif) 1	-1.9	1.6		-1.5	
STAMBP	STAM binding protein	1.6	-1.2			
STAT1	signal transducer and activator of transcription 1, 91kDa	-4.2	1.7	-1.5		
STAT3	signal transducer and activator of transcription 3 (acute-phase response factor)	-1.4	3.8		1.3	
STAT5A	signal transducer and activator of transcription 5A	-3.2	2.7		-1.3	-1.7
STAT5B	signal transducer and activator of transcription 5B	-1.4				
STAT6	signal transducer and activator of transcription 6, interleukin-4 induced	1.5		-1.4		
STAU	staufen, RNA binding protein, homolog 1 (Drosophila)	1.2	-1.2			
STAU2	staufen, RNA binding protein, homolog 2 (Drosophila)	2.5	-2.9	1.6		
STCH	stress 70 protein chaperone, microsome-associated, 60kDa	-2.5	1.6			
STIM1	stromal interaction molecule 1	3.4	-1.4	1.9	1.9	2.0
STIP1	stress-induced-phosphoprotein 1 (Hsp70/Hsp90-organizing protein)	-1.3	-1.6	-1.4	-1.8	
STK10	serine/threonine kinase 10	1.7	-1.4			
STK16	serine/threonine kinase 16	6.9			2.0	
STK17B	serine/threonine kinase 17b (apoptosis-inducing)	4.3		1.6		

STK24	serine/threonine kinase 24 (STE20 homolog, yeast)	1.8	-2.2	1.3		
STK25	serine/threonine kinase 25 (STE20 homolog, yeast)	1.6		1.5		
STK3	serine/threonine kinase 3 (STE20 homolog, yeast)	3.6	-2.2	1.5		
STK38	serine/threonine kinase 38	2.1	-2.3	1.7		
STK39	serine threonine kinase 39 (STE20/SPS1 homolog, yeast)	3.1	-2.5	1.8		
STK4	serine/threonine kinase 4	-1.2		-1.5		
STK6	aurora kinase A	-1.6		1.7		
STMN1	stathmin 1/oncoprotein 18	-1.8		-1.4		
STOML2	stomatin (EPB72)-like 2	-2.8		-1.5		
STRA13	stimulated by retinoic acid 13 homolog (mouse)	-28.6				-1.9
STRAP	serine/threonine kinase receptor associated protein	2.1	-1.4			
STS	steroid sulfatase (microsomal), arylsulfatase C, isozyme S	2.4	-3.6		-1.6	
STX16	syntaxin 16	1.5	1.5			
STX4A	syntaxin 4	1.8				
STX6	syntaxin 6	1.7	-1.9			
STX7	syntaxin 7	1.4	1.5	1.3		
STX8	syntaxin 8	3.2	-2.6			
STXBP1	syntaxin binding protein 1	11.7	-13.5		2.0	3.8
STXBP3	syntaxin binding protein 3	1.9	-1.2	1.7		
SULT1A1	sulfotransferase family, cytosolic, 1A, phenol-preferring, member 1	2.2	-1.5			
SUMO1	SMT3 suppressor of mif two 3 homolog 1 (S. cerevisiae)	-2.1	1.2	-1.3		
SUMO2	SMT3 suppressor of mif two 3 homolog 2 (S. cerevisiae)	-3.1	3.0			
SUMO3	SMT3 suppressor of mif two 3 homolog 3 (S. cerevisiae)	-1.4		-1.3		
SUPT3H	suppressor of Ty 3 homolog (S. cerevisiae)	10.6	-6.4			
SUPT4H1	suppressor of Ty 4 homolog 1 (S. cerevisiae)	2.0	-1.7	1.6		
SUPT6H	suppressor of Ty 6 homolog (S. cerevisiae)	-1.5	1.8			
SURB7	SRB7 suppressor of RNA polymerase B homolog (yeast)	-1.4				
SUZ12	suppressor of zeste 12 homolog (Drosophila)	-1.8				
SV2A	synaptic vesicle glycoprotein 2A	-14.8	10.1	1.9	1.7	1.4
SWAP70	SWAP-70 protein	1.6	-1.3	1.4	1.3	
SYMPK	symplekin	-1.5		-1.7		
SYNCRIP	synaptotagmin binding, cytoplasmic RNA interacting protein	-1.9	-1.7	-1.6		-1.4
SYNE2	spectrin repeat containing, nuclear envelope 2	38.9	-10.7	6.9	1.5	
SYNGR2	synaptogyrin 2	-2.4	1.9			
SYNJ2	synaptojanin 2	48.6	-13.7		1.9	
SYPL	synaptophysin-like 1	-1.9				
TACC1	transforming, acidic coiled-coil containing protein 1	1.7	2.5			
TADA3L	transcriptional adaptor 3 (NGG1 homolog, yeast)-like	1.3				
TAF1	TAF1 RNA polymerase II, TATA box binding protein (TBP)-associated factor, 250kDa	-1.4	1.9			
TAF10	TAF10 RNA polymerase II, TATA box binding protein (TBP)-associated factor, 30kDa	1.4	-1.4			
TAF11	TAF11 RNA polymerase II, TATA box binding protein (TBP)-associated factor, 28kDa	-1.2		1.2		
TAF15	TAF15 RNA polymerase II, TATA box binding protein (TBP)-associated factor, 68kDa	-1.7	1.6	-1.4		

TAF1C	TATA box binding protein (TBP)-associated factor, RNA polymerase I, C, 110kDa	**-1.6**	**1.8**			
TAF2	TAF2 RNA polymerase II, TATA box binding protein (TBP)-associated factor, 150kDa	**1.6**	**-1.3**			
TAF4B	TAF4b RNA polymerase II, TATA box binding protein (TBP)-associated factor, 105kDa	**-2.3**	**-1.3**			
TAF5	TAF5 RNA polymerase II, TATA box binding protein (TBP)-associated factor, 100kDa	**-1.7**		**-1.4**		
TAF7	TAF7 RNA polymerase II, TATA box binding protein (TBP)-associated factor, 55kDa	**1.3**				
TAF9	TAF9 RNA polymerase II, TATA box binding protein (TBP)-associated factor, 32kDa	**-3.2**	**2.2**			
TAL1	T-cell acute lymphocytic leukemia 1	**-2.3**	**1.7**		**1.6**	
TALDO1	transaldolase 1	**-1.7**	**1.8**			
TANK	TRAF family member-associated NFKB activator	**-1.3**	**2.1**	**1.4**		
TAOK3	TAO kinase 3	**-2.9**	**2.3**	**-1.4**		
TAPBP	TAP binding protein (tapasin)	**1.2**	**-1.8**	**-1.3**		
TARBP1	Tar (HIV-1) RNA binding protein 1	**-3.5**			**-1.6**	**-1.8**
TARDBP	TAR DNA binding protein	**-1.3**	**1.9**	**-1.2**		
TARS	threonyl-tRNA synthetase	**-1.8**		**-1.9**		**-1.6**
TAX1BP1	Tax1 (human T-cell leukemia virus type I) binding protein 1	**-2.6**	**4.5**	**1.4**	**1.5**	
TAX1BP3	Tax1 (human T-cell leukemia virus type I) binding protein 3	**4.2**	**-2.1**	**3.3**		
TAZ	tafazzin (cardiomyopathy, dilated 3A (X-linked); endocardial fibroelastosis 2; Barth syndrome)	**1.4**				
TBC1D1	TBC1 (tre-2/USP6, BUB2, cdc16) domain family, member 1	**7.0**		**2.7**		
TBC1D22A	TBC1 domain family, member 22A	**-1.2**	**-1.5**			
TBC1D4	TBC1 domain family, member 4	**-1.6**		**-1.8**		**-1.3**
TBCD	tubulin folding cofactor D	**2.4**		**2.4**	**1.7**	**3.4**
TBPL1	TBP-like 1	**1.5**	**-1.6**			
TCEA1	transcription elongation factor A (SII), 1	**1.5**	**-1.7**			
TCEAL1	transcription elongation factor A (SII)-like 1	**-1.4**	**1.8**			
TCEB2	transcription elongation factor B (SIII), polypeptide 2 (18kDa, elongin B)	**1.4**	**-2.9**			
TCERG1	transcription elongation regulator 1	**-1.4**	**-1.4**	**-1.4**		**-1.4**
TCF12	transcription factor 12 (HTF4, helix-loop-helix transcription factors 4)	**2.8**	**-2.5**	**-2.0**		
TCF3	transcription factor 3 (E2A immunoglobulin enhancer binding factors E12/E47)	**4.7**	**-4.7**			
TCFL1	vacuolar protein sorting 72 homolog (S. cerevisiae)	**-1.6**		**-1.3**	**-1.3**	**-1.5**
TCFL5	transcription factor-like 5 (basic helix-loop-helix)	**2.8**	**-2.5**	**2.1**		
TCP1	t-complex 1	**-1.6**	**-1.3**	**-1.5**		**-1.7**
TDE1	serine incorporator 3	**1.4**		**1.6**		
TDE2	serine incorporator 1	**2.1**		**2.0**		
TDG	thymine-DNA glycosylase	**1.3**				
TDRD7	tudor domain containing 7	**5.6**	**-2.6**			
TEBP	prostaglandin E synthase 3 (cytosolic)	**-1.3**		**-1.4**		
TEGT	testis enhanced gene transcript (BAX inhibitor 1)	**1.3**	**1.2**	**1.4**		
TERF1	telomeric repeat binding factor (NIMA-interacting) 1	**2.0**	**-1.6**			
TERF2	telomeric repeat binding factor 2	**1.4**	**-1.5**			
TERF2IP	telomeric repeat binding factor 2, interacting protein	**1.4**				
TFAM	transcription factor A, mitochondrial	**-2.0**	**-1.4**	**-1.8**		**-1.8**
TFCP2	transcription factor CP2	**1.8**	**-1.5**	**-1.9**		

TFDP1	transcription factor Dp-1	**-1.6**	**-1.8**	**-1.3**		
TFE3	transcription factor binding to IGHM enhancer 3	**2.1**				
TFPI	tissue factor pathway inhibitor (lipoprotein-associated coagulation inhibitor)	**24.6**	**4.9**	**9.6**	**3.1**	**1.9**
TFRC	transferrin receptor (p90, CD71)	**-3.0**		**-4.1**		
TGDS	TDP-glucose 4,6-dehydratase	**-1.3**			**-1.3**	
TGFB1	transforming growth factor, beta 1 (Camurati-Engelmann disease)	**-3.3**	**32.0**			
TGFBR2	transforming growth factor, beta receptor II (70/80kDa)	**6.9**	**-3.3**	**1.8**	**2.8**	**4.0**
TGIF2	TGFB-induced factor 2 (TALE family homeobox)	**-2.9**	**1.9**	**-1.6**		
TGOLN2	trans-golgi network protein 2	**-1.6**	**1.3**	**1.2**		
THOC1	THO complex 1	**-1.8**				
THOC2	THO complex 2	**-1.5**	**1.5**	**1.6**		
THOP1	thimet oligopeptidase 1	**-2.1**				
THRA	thyroid hormone receptor, alpha (erythroblastic leukemia viral (v-erb-a) oncogene homolog, avian)	**2.3**				
THRAP1	thyroid hormone receptor associated protein 1	**-1.3**	**1.9**	**1.5**		
THRAP2	thyroid hormone receptor associated protein 2	**2.3**	**-1.5**	**4.2**		
TIA1	TIA1 cytotoxic granule-associated RNA binding protein	**1.8**	**-1.7**	**-1.3**		
TIAL1	TIA1 cytotoxic granule-associated RNA binding protein-like 1	**-2.0**	**1.7**	**-1.4**		
TIF1	tripartite motif-containing 24	**-1.5**	**1.6**			
TIMELESS	timeless homolog (Drosophila)	**-2.2**		**-1.5**		
TIMM17A	translocase of inner mitochondrial membrane 17 homolog A (yeast)	**-1.6**	**-1.2**	**-1.5**		**-1.5**
TIMM44	translocase of inner mitochondrial membrane 44 homolog (yeast)	**-4.0**				
TIMP1	TIMP metallopeptidase inhibitor 1	**-1.2**				
TIMP2	TIMP metallopeptidase inhibitor 2	**-3.0**	**2.1**			
TIP120A	cullin-associated and neddylation-dissociated 1	**-1.4**	**1.6**	**-1.3**		
TIPARP	TCDD-inducible poly(ADP-ribose) polymerase	**6.1**		**4.0**		
TK1	thymidine kinase 1, soluble	**-3.6**			**1.4**	
TKT	transketolase (Wernicke-Korsakoff syndrome)	**-1.8**	**1.5**	**-1.3**		
TLE1	transducin-like enhancer of split 1 (E(sp1) homolog, Drosophila)	**7.4**	**-2.0**	**5.6**		
TLE4	transducin-like enhancer of split 4 (E(sp1) homolog, Drosophila)	**1.7**	**-1.4**	**-1.5**		
TLN1	talin 1	**2.6**	**-1.6**		**1.4**	
TM2D1	TM2 domain containing 1	**1.8**	**1.6**	**1.8**		
TM4SF2	tetraspanin 7	**-11.4**	**35.8**	**4.8**		
TM4SF7	tetraspanin 4	**-1.3**		**-1.6**		
TMED3	transmembrane emp24 protein transport domain containing 3	**-1.4**			**-1.7**	
TMED9	transmembrane emp24 protein transport domain containing 9	**-2.4**	**1.8**			
TMEM1	transmembrane protein 1	**1.5**	**-1.4**			
TMEM4	transmembrane protein 4	**-3.3**	**1.9**	**-1.5**		
TMF1	TATA element modulatory factor 1	**-2.2**	**2.1**	**1.7**	**-1.2**	
TMP21	transmembrane emp24-like trafficking protein 10 (yeast)	**2.0**	**1.5**			
TMPO	thymopoietin	**1.7**	**-2.2**	**1.4**		
TMSB10	thymosin, beta 10	**3.2**	**-1.9**	**1.6**	**1.4**	**2.2**
TMSB4X	thymosin, beta 4, X-linked	**1.6**		**1.7**		

TMSL8	thymosin-like 8	2.0	-5.4			
TNFAIP3	tumor necrosis factor, alpha-induced protein 3	4.1				-1.7
TNFAIP8	tumor necrosis factor, alpha-induced protein 8	-1.9	-1.5	-1.9	-1.3	
TNFRSF10D	tumor necrosis factor receptor superfamily, member 10d, decoy with truncated death domain	-2.0	1.3			
TNFRSF1A	tumor necrosis factor receptor superfamily, member 1A	-5.7	2.9			
TNFSF10	tumor necrosis factor (ligand) superfamily, member 10	-4.1		-5.3		
TNFSF8	tumor necrosis factor (ligand) superfamily, member 8	18.3			5.2	
TNKS	tankyrase, TRF1-interacting ankyrin-related ADP-ribose polymerase	1.3	-1.7	2.5		
TNPO1	transportin 1	-1.6	-1.4	1.4		
TOE1	target of EGR1, member 1 (nuclear)	-1.4				-1.5
TOMM20	translocase of outer mitochondrial membrane 20 homolog (yeast)	-2.1	2.0		-1.5	
TOMM34	translocase of outer mitochondrial membrane 34	-1.4				
TOMM40	translocase of outer mitochondrial membrane 40 homolog (yeast)	-1.6		-1.4		
TOMM70A	translocase of outer mitochondrial membrane 70 homolog A (S. cerevisiae)	-1.8		-1.5	-1.4	
TOP2A	topoisomerase (DNA) II alpha 170kDa	1.3		1.8	1.4	
TOP2B	topoisomerase (DNA) II beta 180kDa	1.4	-1.9			
TOPBP1	topoisomerase (DNA) II binding protein 1	-1.5	-1.2			
TOX	thymus high mobility group box protein TOX	-32.2	26.1	-1.6	-1.3	
TPD52L2	tumor protein D52-like 2	1.4	-1.6			
TPMT	thiopurine S-methyltransferase	-2.2	1.8			
TPP1	tripeptidyl peptidase I	-1.3	1.7	1.4		
TPP2	tripeptidyl peptidase II	-1.3	-1.3			
TPR	translocated promoter region (to activated MET oncogene)	-1.6	1.4	-1.3		
TPST2	tyrosylprotein sulfotransferase 2	2.9	-1.7	2.0		
TRA@	T cell receptor alpha locus	24.7	-38.6	-1.9		2.9
TRA1	heat shock protein 90kDa beta (Grp94), member 1	-2.2	1.4			
TRA2A	transformer-2 alpha	-2.3	2.6			
TRAF3	TNF receptor-associated factor 3	-7.4	2.8			
TRAF3IP2	TRAF3 interacting protein 2	3.8	-5.3			
TRAM1	translocation associated membrane protein 1	-2.3	3.3			
TRAM2	translocation associated membrane protein 2	2.0	-2.0	2.4	1.5	1.7
TRAP1	TNF receptor-associated protein 1	-1.8		-1.9	-1.6	
TRAPPC3	trafficking protein particle complex 3	-1.4	1.4			
TRIB2	tribbles homolog 2 (Drosophila)	-6.0	6.1			
TRIM14	tripartite motif-containing 14	-4.7	3.2	-2.4		
TRIM21	tripartite motif-containing 21	1.4		-1.6	1.4	
TRIM23	tripartite motif-containing 23	1.3		1.8		
TRIM28	tripartite motif-containing 28	-2.0		-1.3		
TRIM32	tripartite motif-containing 32	1.5				
TRIM33	tripartite motif-containing 33	-1.2	-1.6			
TRIM37	tripartite motif-containing 37	-14.1	1.8	-1.3		
TRIP12	thyroid hormone receptor interactor 12	2.3	1.6	1.5		

TRIP13	thyroid hormone receptor interactor 13	-2.4			-1.5	-1.6
TRRAP	transformation/transcription domain-associated protein	2.1	-1.7			
TSC2	tuberous sclerosis 2	6.8	-1.8	1.5		
TSC22D1	TSC22 domain family, member 1	-2.4	2.0	2.9		
TSC22D3	TSC22 domain family, member 3	20.0	5.2	17.5	33.1	20.4
TSC22D4	TSC22 domain family, member 4	2.8				
TSFM	Ts translation elongation factor, mitochondrial	-1.6	-1.2	-2.5	-1.5	-1.6
TSG101	tumor susceptibility gene 101	1.6				
TSN	translin	-1.4		-1.3		
TSNAX	translin-associated factor X	2.0	1.2	1.9	2.1	2.2
TSR1	TSR1, 20S rRNA accumulation, homolog (S. cerevisiae)	-1.8	-2.0	-2.4	-2.0	-2.0
TTC3	tetratricopeptide repeat domain 3	-5.1	2.3	-1.6		
TTF1	transcription termination factor, RNA polymerase I	1.8	-2.7	-1.3		
TTLL12	tubulin tyrosine ligase-like family, member 12	-4.9		-2.7	-1.4	
TTN	titin	-8.8		-1.4		
TUBA1	tubulin, alpha 1	13.5	-4.4	2.1	10.4	3.0
TUBA3	tubulin, alpha 3	15.1	-10.6	1.7		-1.6
TUBB2A	tubulin, beta 2A	54.6	-25.5	3.2		
TUBG1	tubulin, gamma 1	-3.2				-1.4
TUBGCP2	tubulin, gamma complex associated protein 2	-1.5		-1.3		-1.4
TUBGCP3	tubulin, gamma complex associated protein 3	-1.7	-1.4			
TXLNA	taxilin alpha	-2.0	1.2			
TXN	thioredoxin	-1.5	1.8	1.3		1.7
TXNDC1	thioredoxin domain containing 1	-2.0		-1.6		
TXNDC9	thioredoxin domain containing 9	-1.2	1.5			
TXNIP	thioredoxin interacting protein	2.1	10.0	7.7	2.8	3.7
TXNL1	thioredoxin-like 1	-1.4	1.3	-2.1		
TYMS	thymidylate synthetase	-4.8	2.1	-1.2		
UAP1	UDP-N-acteylglucosamine pyrophosphorylase 1	-3.9	1.8	-2.3	-1.3	-1.5
UBA2	SUMO1 activating enzyme subunit 2	-1.9		-1.6		
UBB	ubiquitin B	1.5	-1.6	1.7		
UBC	ubiquitin C	1.5		1.6		
UBE1C	ubiquitin-activating enzyme E1C (UBA3 homolog, yeast)	1.5		1.2		
UBE1L	ubiquitin-activating enzyme E1-like	1.8	-1.5	-1.4		
UBE2D1	ubiquitin-conjugating enzyme E2D 1 (UBC4/5 homolog, yeast)	1.3	-1.3			
UBE2D2	ubiquitin-conjugating enzyme E2D 2 (UBC4/5 homolog, yeast)	4.1	-1.9	1.6		
UBE2D3	ubiquitin-conjugating enzyme E2D 3 (UBC4/5 homolog, yeast)	1.6	-1.3	1.3		-1.3
UBE2E1	ubiquitin-conjugating enzyme E2E 1 (UBC4/5 homolog, yeast)	-1.8	1.4			
UBE2E3	ubiquitin-conjugating enzyme E2E 3 (UBC4/5 homolog, yeast)	-2.1	2.2			
UBE2G2	ubiquitin-conjugating enzyme E2G 2 (UBC7 homolog, yeast)	-3.2		-1.9		
UBE2I	ubiquitin-conjugating enzyme E2I (UBC9 homolog, yeast)	2.8	-4.5	-1.2		
UBE2J1	ubiquitin-conjugating enzyme E2, J1 (UBC6 homolog, yeast)	-1.5	1.5	1.5		

UBE2L3	ubiquitin-conjugating enzyme E2L 3	**-2.1**	**1.5**	**-2.1**		
UBE2M	ubiquitin-conjugating enzyme E2M (UBC12 homolog, yeast)	**-2.6**			**-1.4**	
UBE2V2	ubiquitin-conjugating enzyme E2 variant 2	**-1.7**	**-1.4**	**-1.3**		
UBE3A	ubiquitin protein ligase E3A (human papilloma virus E6-associated protein, Angelman syndrome)	**8.6**	**-5.8**	**1.4**	**-1.4**	
UBE4A	ubiquitination factor E4A (UFD2 homolog, yeast)	**1.3**				
UBE4B	ubiquitination factor E4B (UFD2 homolog, yeast)	**-1.3**	**1.3**			
UBN1	ubinuclein 1	**1.3**	**-1.8**	**-1.2**		
UBR2	ubiquitin protein ligase E3 component n-recognin 2	**1.3**				
UBTF	upstream binding transcription factor, RNA polymerase I	**-1.4**	**-1.2**	**-1.3**	**-1.3**	
UCHL3	ubiquitin carboxyl-terminal esterase L3 (ubiquitin thiolesterase)	**-2.8**		**-1.7**		
UCK2	uridine-cytidine kinase 2	**-3.0**	**-1.7**	**-1.4**		
UCKL1	uridine-cytidine kinase 1-like 1	**1.7**		**2.1**		
UFD1L	ubiquitin fusion degradation 1 like (yeast)	**-2.3**		**-1.4**		
UGCG	UDP-glucose ceramide glucosyltransferase	**3.0**	**-2.4**	**1.2**		
ULK1	unc-51-like kinase 1 (C. elegans)	**3.8**				
UMPS	uridine monophosphate synthetase (orotate phosphoribosyl transferase and orotidine-5'-decarboxylase)	**-1.6**	**-1.3**		**-1.3**	
UNG	uracil-DNA glycosylase	**-10.0**				
UPF2	UPF2 regulator of nonsense transcripts homolog (yeast)	**-2.0**	**2.2**			
UPF3A	UPF3 regulator of nonsense transcripts homolog A (yeast)	**1.4**	**-1.4**			
UQCRB	ubiquinol-cytochrome c reductase binding protein	**1.3**				
UQCRC1	ubiquinol-cytochrome c reductase core protein I	**-1.3**		**-2.0**		
UQCRC2	ubiquinol-cytochrome c reductase core protein II	**1.8**	**-1.6**			
UQCRFS1	ubiquinol-cytochrome c reductase, Rieske iron-sulfur polypeptide 1	**-1.6**	**1.2**			
UQCRH	ubiquinol-cytochrome c reductase hinge protein	**-1.9**	**1.5**			
UROD	uroporphyrinogen decarboxylase	**-2.4**	**2.2**			
UROS	uroporphyrinogen III synthase (congenital erythropoietic porphyria)	**-6.2**	**2.4**	**1.6**		
USF2	upstream transcription factor 2, c-fos interacting	**1.6**	**-1.5**			
USP10	ubiquitin specific peptidase 10	**-1.6**		**-1.7**		
USP11	ubiquitin specific peptidase 11	**1.4**	**-1.6**	**1.3**		
USP12	ubiquitin specific peptidase 12	**3.7**	**-2.0**	**2.3**		
USP14	ubiquitin specific peptidase 14 (tRNA-guanine transglycosylase)	**-1.7**	**1.3**	**-1.3**		
USP22	ubiquitin specific peptidase 22	**2.7**	**-2.6**			
USP24	ubiquitin specific peptidase 24	**-1.3**	**1.4**	**1.3**		
USP32	ubiquitin specific peptidase 32	**-2.2**	**3.1**			
USP34	ubiquitin specific peptidase 34	**2.6**	**1.3**	**1.8**		
USP4	ubiquitin specific peptidase 4 (proto-oncogene)	**2.4**	**-1.7**	**1.3**		
USP46	ubiquitin specific peptidase 46	**-3.0**	**3.5**			
USP52	ubiquitin specific peptidase 52	**1.5**				
USP7	ubiquitin specific peptidase 7 (herpes virus-associated)	**2.6**	**-3.0**			
USP8	ubiquitin specific peptidase 8	**1.5**		**1.5**		
USP9X	ubiquitin specific peptidase 9, X-linked	**1.3**		**1.4**		
UTX	ubiquitously transcribed tetratricopeptide repeat, X chromosome	**2.9**	**-1.6**	**1.4**		

VAMP1	vesicle-associated membrane protein 1 (synaptobrevin 1)	**2.1**		**1.3**		
VAMP2	vesicle-associated membrane protein 2 (synaptobrevin 2)	**4.9**	**-4.2**	**-1.3**		
VAMP3	vesicle-associated membrane protein 3 (cellubrevin)	**1.5**	**1.3**	**1.3**		
VAMP4	vesicle-associated membrane protein 4	**2.7**	**-2.6**			
VAMP8	vesicle-associated membrane protein 8 (endobrevin)	**-4.1**	**2.0**			
VAPA	VAMP (vesicle-associated membrane protein)-associated protein A, 33kDa	**-1.7**	**1.3**			
VAPB	VAMP (vesicle-associated membrane protein)-associated protein B and C	**-1.9**				
VARS	valyl-tRNA synthetase	**-2.1**		**-2.1**		
VAV1	vav 1 oncogene	**-1.2**				
VBP1	von Hippel-Lindau binding protein 1	**-1.9**				
VCL	vinculin	**-1.4**	**1.9**	**-1.5**		**1.9**
VCP	valosin-containing protein	**-1.4**	**1.3**	**-1.3**		
VDAC1	voltage-dependent anion channel 1	**-3.1**	**1.6**		**-1.6**	**-1.7**
VDAC2	voltage-dependent anion channel 2	**-2.1**	**1.9**			**-1.5**
VDAC3	voltage-dependent anion channel 3	**-1.9**	**1.8**			
VDP	vesicle docking protein p115	**-1.6**	**2.0**			
VEGF	vascular endothelial growth factor A	**-13.8**	**8.2**	**-2.4**	**-1.5**	
VIL2	villin 2 (ezrin)	**9.8**	**-2.2**	**2.4**		
VIM	vimentin	**360.4**	**-275.9**	**1.2**		
VLDLR	very low density lipoprotein receptor	**2.6**	**-4.7**	**-1.2**		
VPS13A	vacuolar protein sorting 13 homolog A (S. cerevisiae)	**-2.8**	**3.7**			**-1.3**
VPS4B	vacuolar protein sorting 4 homolog B (S. cerevisiae)	**1.3**				
VPS52	vacuolar protein sorting 52 homolog (S. cerevisiae)	**1.3**				
VRK1	vaccinia related kinase 1	**-1.7**				
VTI1B	vesicle transport through interaction with t-SNAREs homolog 1B (yeast)	**-1.7**	**1.7**	**1.3**		
WARS	tryptophanyl-tRNA synthetase	**-1.8**	**-1.5**	**-3.3**		
WAS	Wiskott-Aldrich syndrome (eczema-thrombocytopenia)	**3.4**				
WASF1	WAS protein family, member 1	**-1.3**				
WBP4	WW domain binding protein 4 (formin binding protein 21)	**1.5**	**-1.2**			
WBSCR1	eukaryotic translation initiation factor 4H	**1.5**	**-2.4**			
WDHD1	WD repeat and HMG-box DNA binding protein 1	**-5.6**				
WDR1	WD repeat domain 1	**2.8**	**-2.5**			
WDR39	cytosolic iron-sulfur protein assembly 1 homolog (S. cerevisiae)	**-2.3**		**-1.9**		
WDR46	WD repeat domain 46	**-1.6**				
WHSC2	Wolf-Hirschhorn syndrome candidate 2	**-1.4**				
WIT-1	Wilms tumor upstream neighbor 1	**2.5**	**-2.4**			
WNK1	WNK lysine deficient protein kinase 1	**4.2**				
WRB	tryptophan rich basic protein	**1.2**				
WRN	Werner syndrome	**-1.7**		**-1.6**	**-1.8**	
WSB1	WD repeat and SOCS box-containing 1	**-2.0**	**9.0**	**2.0**		
WSB2	WD repeat and SOCS box-containing 2	**-1.7**	**1.4**	**1.3**		
WT1	Wilms tumor 1	**-1.7**	**-1.6**	**1.4**	**-1.5**	**-1.8**

WTAP	Wilms tumor 1 associated protein	**1.8**	**-1.5**	**1.7**		
WWP1	WW domain containing E3 ubiquitin protein ligase 1	**2.1**		**1.6**		
XAB1	XPA binding protein 1, GTPase	**1.6**	**-1.6**			
XBP1	X-box binding protein 1	**1.4**		**1.4**		
XIST	X (inactive)-specific transcript	**1094.1**	**-808.5**			
XPNPEP1	X-prolyl aminopeptidase (aminopeptidase P) 1, soluble	**-3.9**	**3.2**			
XPO1	exportin 1 (CRM1 homolog, yeast)	**4.3**	**-1.4**			
XPO7	exportin 7	**-2.7**	**4.8**	**1.3**		
XPOT	exportin, tRNA (nuclear export receptor for tRNAs)	**-6.4**	**1.6**	**-3.2**		
XRCC4	X-ray repair complementing defective repair in Chinese hamster cells 4	**1.6**	**-1.8**	**1.4**		
YAF2	YY1 associated factor 2	**5.8**	**1.7**	**1.9**	**1.8**	**1.6**
YARS	tyrosyl-tRNA synthetase	**-2.4**	**-1.9**	**-2.6**		
YES1	v-yes-1 Yamaguchi sarcoma viral oncogene homolog 1	**-63.8**	**49.1**			**1.3**
YIF1	Yip1 interacting factor homolog A (S. cerevisiae)	**-2.1**		**-1.5**		
YME1L1	YME1-like 1 (S. cerevisiae)	**-1.7**	**1.7**	**-1.2**		
YWHAB	tyrosine 3-monooxygenase/tryptophan 5-monooxygenase activation protein, beta polypeptide	**-1.5**				
YWHAE	tyrosine 3-monooxygenase/tryptophan 5-monooxygenase activation protein, epsilon polypeptide	**2.2**	**-2.5**	**-1.6**		
YWHAH	tyrosine 3-monooxygenase/tryptophan 5-monooxygenase activation protein, eta polypeptide	**1.6**				
YWHAQ	tyrosine 3-monooxygenase/tryptophan 5-monooxygenase activation protein, theta polypeptide	**1.5**	**-1.7**	**1.6**		
YWHAZ	tyrosine 3-monooxygenase/tryptophan 5-monooxygenase activation protein, zeta polypeptide	**3.9**	**-2.5**	**1.7**		
YY1	YY1 transcription factor	**-2.7**	**2.7**			
ZA20D2	zinc finger, AN1-type domain 5	**1.5**		**1.9**		
ZAP70	zeta-chain (TCR) associated protein kinase 70kDa	**13.8**	**-10.1**			**-1.3**
ZBTB1	zinc finger and BTB domain containing 1	**1.6**	**-1.4**			
ZBTB11	zinc finger and BTB domain containing 11	**-1.6**		**-1.3**		
ZBTB5	zinc finger and BTB domain containing 5	**-1.4**				
ZC3HAV1	zinc finger CCCH-type, antiviral 1	**2.6**	**-1.7**	**1.4**		
ZDHHC17	zinc finger, DHHC-type containing 17	**1.2**				
ZFP36	zinc finger protein 36, C3H type, homolog (mouse)	**4.1**		**3.2**		
ZFP36L1	zinc finger protein 36, C3H type-like 1	**11.5**	**-24.5**			
ZFX	zinc finger protein, X-linked	**1.7**	**-1.6**			
ZFYVE16	zinc finger, FYVE domain containing 16	**2.7**		**1.6**		
ZHX2	zinc fingers and homeoboxes 2	**6.9**	**-5.1**	**1.3**		
ZMPSTE24	zinc metallopeptidase (STE24 homolog, yeast)	**-1.8**	**1.4**			
ZMYM3	zinc finger, MYM-type 3	**-1.8**				
ZNF124	zinc finger protein 124	**-1.7**		**-1.4**		
ZNF148	zinc finger protein 148	**-1.6**	**1.8**	**1.8**	**1.3**	
ZNF160	zinc finger protein 160	**1.6**	**-1.7**	**1.5**		
ZNF161	vascular endothelial zinc finger 1	**2.2**	**-1.9**			
ZNF175	zinc finger protein 175	**-2.6**	**2.9**			
ZNF198	zinc finger, MYM-type 2	**-2.5**	**4.3**	**-1.3**		
ZNF217	zinc finger protein 217	**1.3**				

ZNF22	zinc finger protein 22 (KOX 15)	**1.3**	**-1.3**			
ZNF238	zinc finger protein 238	**6.3**	**-5.5**			
ZNF259	zinc finger protein 259	**-1.6**	**-1.2**		**-1.4**	**-1.8**
ZNF263	zinc finger protein 263	**1.8**	**-1.7**	**-1.7**		
ZNF267	zinc finger protein 267	**-1.9**	**1.9**			
ZNF268	zinc finger protein 268	**-1.8**				
ZNF291	zinc finger protein 291	**2.1**	**1.4**	**1.6**		
ZNF330	zinc finger protein 330	**-1.8**				**-1.6**
ZNF364	zinc finger protein 364	**3.0**	**1.3**	**1.8**		
ZNF423	zinc finger protein 423	**5.6**	**-3.6**	**-2.2**		
ZNF451	zinc finger protein 451	**-1.6**	**2.5**			
ZNF592	zinc finger protein 592	**1.4**	**-1.7**	**1.3**		
ZNF84	zinc finger protein 84	**-1.4**				
ZNF9	CCHC-type zinc finger, nucleic acid binding protein	**-1.5**		**-1.4**		
ZNF91	zinc finger protein 91	**2.2**	**-2.2**			
ZNF96	zinc finger protein 96	**-1.9**				
ZNFN1A1	IKAROS family zinc finger 1 (Ikaros)	**2.2**	**2.2**	**1.3**		
ZRF1	zuotin related factor 1	**-2.9**	**1.4**	**-1.5**		
ZRSR2	zinc finger (CCCH type), RNA-binding motif and serine/arginine rich 2	**1.8**				
ZUBR1	zinc finger, UBR1 type 1	**-1.9**	**2.0**			
ZWINT	ZW10 interactor	**-2.3**		**-1.3**		
