# Supplementary material for: Gene expression profiling of leukemic cells and primary thymocytes predicts a signature for apoptotic sensitivity to glucocorticoids
Source: Cancer Cell Int. 2007 Nov 28;7:18. doi: 10.1186/1475-2867-7-18 (PMC2228275; doi:10.1186/1475-2867-7-18)
Supplement: Additional file 7 — T-cell ALL vs. B-cell ALL. Genes regulated in common in the same sense by Dex in GC-sensitive T-cell ALL CEM-C7–14, CEM-C1–6 and GC-sensitive B-cell ALL RS4;11, SUP-B15 cells. Bold type indicates statistically significant regulation p ≤ 0.05 between means of vehicle vs. GC-treated. [file 1475-2867-7-18-S7.pdf]

Additional file 7: T-cell ALL vs. B-cell ALL. (Pediatric and Adult)									
Page 1									
GC-response		Sensitive	Sensitive	Sensitive	Sensitive	Sensitive	Sensitive	Sensitive	Sensitive
Patient-derived cell line		Pediatric	Pediatric	Pediatric	Pediatric	Adult	Adult	Pediatric	Pediatric
Cell lineage		T-cell	T-cell	T-cell	T-cell	B-cell	B-cell	B-cell	B-cell
Sub-type of leukemia		ALL	ALL	ALL	ALL	ALL	ALL	ALL	ALL
Name	Description	C7-14 Dx	C7-14 Dx	C1-6 Dx	C1-6 Dx	RS4 Dx	RS4 Dx	SUP Dx	SUP Dx
			Stat. sign		Stat. sign		Stat. sign		Stat. sign
AARS	alanyl-tRNA synthetase	-1.3	**-1.3**	-1.2		-2.0	**-2.0**	-1.9	**-1.9**
ABI1	abl-interactor 1	1.3		1.3		1.4	**1.4**	1.7	**1.7**
ACLY	ATP citrate lyase	-1.3		-1.2		-1.4	**-1.4**	-1.7	**-1.7**
ACSL3	acyl-CoA synthetase long-chain family member 3	-1.3		-1.3		-1.8	**-1.8**	-1.3	**-1.3**
ADAM9	ADAM metallopeptidase domain 9 (meltrin gamma)	1.3		1.3		2.4	**2.4**	1.6	**1.6**
AK2	adenylate kinase 2	-1.4		-1.6	**-1.6**	-1.4	**-1.4**	-2.2	**-2.2**
AKAP1	A kinase (PRKA) anchor protein 1	-1.6	**-1.6**	-1.9		-1.8	**-1.8**	-2.4	**-2.4**
ANKRD40	ankyrin repeat domain 40	-1.4		-1.2		-1.5	**-1.5**	-1.5	
AP3S1	adaptor-related protein complex 3, sigma 1 subunit	1.6	**1.6**	1.9	**1.9**	1.4	**1.4**	2.4	**2.4**
APG12L	ATG12 autophagy related 12 homolog (S. cerevisiae)	1.6	**1.6**	1.9	**1.9**	1.3	**1.3**	1.9	**1.9**
APPBP1	amyloid beta precursor protein binding protein 1	-1.2		-1.5		-1.2	**-1.2**	-1.3	**-1.3**
APPBP2	amyloid beta precursor protein (cytoplasmic tail) binding protein 2	1.3		1.7		2.0	**2.0**	1.4	**1.4**
ARHGEF18	rho/rac guanine nucleotide exchange factor (GEF) 18	1.3	**1.3**	1.4		1.5	**1.5**	2.5	**2.5**
ATF4	activating transcription factor 4 (tax-responsive enhancer element B67)	-1.5	**-1.5**	-1.3		-1.3	**-1.3**	-1.4	**-1.4**
ATF5	activating transcription factor 5	-1.5		-1.3		-1.6	**-1.6**	-2.0	**-2.0**
ATIC	5-aminoimidazole-4-carboxamide ribonucleotide formyltransferase/IMP cyclohydrolase	-1.4	**-1.4**	-2.0	**-2.0**	-2.1	**-2.1**	-3.2	**-3.2**
ATP2B1	ATPase, Ca++ transporting, plasma membrane 1	-1.4		-2.0		-2.1	**-2.1**	-2.1	**-2.1**
ATP6V0D1	ATPase, H+ transporting, lysosomal 38kDa, V0 subunit d1	1.3	**1.3**	1.3		1.4	**1.4**	1.2	
BAG2	BCL2-associated athanogene 2	-1.2		-1.6		-1.2	**-1.2**	-1.8	**-1.8**
BCL2L11	BCL2-like 11 (apoptosis facilitator)	3.1	**3.1**	5.9	**5.9**	1.5	**1.2**	3.1	**3.1**
BDH1	3-hydroxybutyrate dehydrogenase, type 1	-1.3	**-1.3**	-1.5	**-1.5**	-1.3	**-1.3**	-1.7	
BIRC2	baculoviral IAP repeat-containing 2	1.9	**1.9**	1.5		1.4	**1.4**	1.9	**1.9**
BTG1	B-cell translocation gene 1, anti-proliferative	9.3	**9.3**	4.4	**4.4**	1.2		3.9	**3.9**
BYSL	bystin-like	-2.2	**-2.2**	-2.3	**-2.3**	-6.2	**-6.2**	-8.4	**-8.4**
C1QBP	complement component 1, q subcomponent binding protein	-1.8	**-1.8**	-2.0		-2.0	**-2.0**	-1.9	**-1.9**
C5ORF13	chromosome 5 open reading frame 13	-1.3	**-1.3**	-1.5		-3.7	**-3.7**	-3.3	**-3.3**
CAPN7	calpain 7	1.4		1.3		1.4	**1.4**	1.5	
CASP8	caspase 8, apoptosis-related cysteine peptidase	1.3		1.3		3.2	**3.2**	1.5	**1.5**
CCDC6	coiled-coil domain containing 6	1.3		1.6		2.6	**2.6**	1.9	**1.9**

Additional file 7: T-cell ALL vs. B-cell ALL. (Pediatric and Adult)									
Page 2									
GC-response		Sensitive	Sensitive	Sensitive	Sensitive	Sensitive	Sensitive	Sensitive	Sensitive
Patient-derived cell line		Pediatric	Pediatric	Pediatric	Pediatric	Adult	Adult	Pediatric	Pediatric
Cell lineage		T-cell	T-cell	T-cell	T-cell	B-cell	B-cell	B-cell	B-cell
Sub-type of leukemia		ALL	ALL	ALL	ALL	ALL	ALL	ALL	ALL
Name	Description	C7-14 Dx	C7-14 Dx	C1-6 Dx	C1-6 Dx	RS4 Dx	RS4 Dx	SUP Dx	SUP Dx
			Stat. sign		Stat. sign		Stat. sign		Stat. sign
CCT5	chaperonin containing TCP1, subunit 5 (epsilon)	-1.6	**-1.6**	-1.7		-1.3		-1.5	**-1.5**
CD53	CD53 molecule	3.1	**3.1**	3.2	**3.2**	1.4	**1.4**	3.1	**3.1**
CD69	CD69 molecule	4.9	**4.9**	2.0	**2.0**	6.3	**6.3**	3.8	**3.8**
CD99	CD99 molecule	1.2		1.8	**1.8**	1.7	**1.7**	3.4	**3.4**
CDC25A	cell division cycle 25 homolog A (S. cerevisiae)	-1.4	**-1.4**	-1.4		-1.6	**-1.6**	-1.4	**-1.4**
CDC6	cell division cycle 6 homolog (S. cerevisiae)	-1.3	**-1.3**	-1.5	**-1.5**	-1.7	**-1.7**	-2.9	**-2.9**
CEBPZ	CCAAT/enhancer binding protein zeta	-1.5	**-1.5**	-1.8	**-1.8**	-2.2	**-1.7**	-2.6	**-1.5**
CENTB2	centaurin, beta 2	1.3		1.3	**1.3**	1.5	**1.5**	1.5	**1.5**
CHC1	regulator of chromosome condensation 1	-1.7	**-1.7**	-1.6	**-1.6**	-1.2		-1.8	**-1.8**
CLK1	CDC-like kinase 1	1.2		1.3		1.5	**1.5**	1.7	**1.7**
COMT	catechol-O-methyltransferase	-1.4		-1.4		-2.1	**-2.1**	-1.4	
CPSF5	nudix (nucleoside diphosphate linked moiety X)-type motif 21	-1.3		-1.2		-1.8	**-1.8**	-1.3	**-1.3**
CSE1L	CSE1 chromosome segregation 1-like (yeast)	-1.3		-1.4		-1.6	**-1.6**	-1.4	**-1.4**
CTPS	CTP synthase	-1.7		-1.8		-1.5	**-1.5**	-1.4	**-1.4**
CUGBP2	CUG triplet repeat, RNA binding protein 2	2.1	**2.0**	1.8	**1.8**	5.7	**5.7**	2.4	**2.4**
DDIT4	DNA-damage-inducible transcript 4	4.4	**4.4**	2.5		5.5	**5.5**	4.6	**4.6**
DDX18	DEAD (Asp-Glu-Ala-Asp) box polypeptide 18	-1.5		-1.6		-1.3	**-1.3**	-1.7	**-1.7**
DGUOK	deoxyguanosine kinase	-1.3	**-1.3**	-1.3	**-1.3**	-1.7		-1.3	
DHRS1	dehydrogenase/reductase (SDR family) member 1	1.7	**1.7**	1.4		2.3	**2.3**	1.5	
DHX30	DEAH (Asp-Glu-Ala-His) box polypeptide 30	-1.3		-1.3		-2.1	**-2.1**	-1.7	**-1.7**
DLAT	dihydrolipoamide S-acetyltransferase (E2 component of pyruvate dehydrogenase complex)	-1.3		-1.5		-1.6	**-1.6**	-2.1	**-2.1**
DNAJA1	DnaJ (Hsp40) homolog, subfamily A, member 1	-1.3	**-1.3**	-1.7	**-1.7**	-1.7	**-1.7**	-1.4	**-1.4**
DNPEP	aspartyl aminopeptidase	-1.2	**-1.2**	-1.4		-4.5	**-4.5**	-1.6	**-1.6**
DSCR1	Down syndrome critical region gene 1	5.0	**5.0**	4.8	**4.8**	1.6	**1.5**	7.7	**7.7**
EEF1E1	eukaryotic translation elongation factor 1 epsilon 1	-1.7	**-1.7**	-2.3		-1.6	**-1.6**	-3.3	**-3.3**
EIF2S1	eukaryotic translation initiation factor 2, subunit 1 alpha, 35kDa	-1.4	**-1.4**	-1.4	**-1.4**	-1.2	**-1.2**	-1.6	**-1.6**
EIF3S8	eukaryotic translation initiation factor 3, subunit 8, 110kDa	-1.2	**-1.2**	-1.2		-3.1	**-3.1**	-1.6	**-1.4**
EIF3S9	eukaryotic translation initiation factor 3, subunit 9 eta, 116kDa	-1.5	**-1.5**	-1.3		-1.2	**-1.2**	-2.0	**-2.0**
EXOSC2	exosome component 2	-1.7	**-1.7**	-1.2		-1.2		-2.4	**-2.4**

Additional file 7: T-cell ALL vs. B-cell ALL. (Pediatric and Adult)									
**Page 3**									
**GC-response**		**Sensitive**	**Sensitive**	**Sensitive**	**Sensitive**	**Sensitive**	**Sensitive**	**Sensitive**	**Sensitive**
**Patient-derived cell line**		**Pediatric**	**Pediatric**	**Pediatric**	**Pediatric**	**Adult**	**Adult**	**Pediatric**	**Pediatric**
**Cell lineage**		**T-cell**	**T-cell**	**T-cell**	**T-cell**	**B-cell**	**B-cell**	**B-cell**	**B-cell**
**Sub-type of leukemia**		**ALL**	**ALL**	**ALL**	**ALL**	**ALL**	**ALL**	**ALL**	**ALL**
**Name**	**Description**	**C7-14 Dx**	**C7-14 Dx**	**C1-6 Dx**	**C1-6 Dx**	**RS4 Dx**	**RS4 Dx**	**SUP Dx**	**SUP Dx**
			**Stat. sign**		**Stat. sign**		**Stat. sign**		**Stat. sign**
FADS1	fatty acid desaturase 1	-1.5		-1.9	**-1.9**	-1.5	**-1.5**	-1.5	**-1.5**
FADS2	fatty acid desaturase 2	-1.4		-1.3		-1.9	**-1.9**	-2.0	**-2.0**
FH	fumarate hydratase	-1.2		-1.2		-1.4	**-1.4**	-2.1	**-2.1**
FKBP5	FK506 binding protein 5	6.6	**6.6**	4.4	**4.4**	7.0	**7.0**	21.4	**21.4**
FNBP1L	formin binding protein 1-like	1.9	**1.9**	1.8		1.4	**1.4**	3.0	**3.0**
FOXO3A	forkhead box O3A	1.3		1.2		1.9	**1.7**	2.7	**2.7**
FZD6	frizzled homolog 6 (Drosophila)	1.5	**1.5**	1.3		10.1	**10.1**	2.1	**2.1**
GLUL	glutamate-ammonia ligase (glutamine synthetase)	1.9	**1.9**	3.4		2.3	**2.3**	2.5	**2.5**
GM2A	GM2 ganglioside activator	1.3	**1.3**	1.5		1.4		1.5	
GMPS	guanine monphosphate synthetase	-1.3		-1.4		-1.4	**-1.4**	-1.4	**-1.4**
GSK3B	glycogen synthase kinase 3 beta	1.6		1.2		2.1	**2.1**	1.5	**1.3**
GSPT1	G1 to S phase transition 1	-1.5	**-1.5**	-1.8	**-1.8**	-1.6	**-1.6**	-1.2	**-1.2**
GTF2E2	general transcription factor IIE, polypeptide 2, beta 34kDa	-1.4	**-1.4**	-1.3		-1.5	**-1.5**	-1.3	**-1.3**
GTF3A	general transcription factor IIIA	-1.3		-1.5		-1.7	**-1.7**	-1.4	**-1.4**
H1F0	H1 histone family, member 0	1.4		2.5	**2.5**	8.1	**8.1**	2.4	**2.4**
HAX1	HCLS1 associated protein X-1	-1.2		-1.3	**-1.3**	-1.3	**-1.3**	-1.7	**-1.7**
HBP1	HMG-box transcription factor 1	1.5		1.6	**1.6**	1.6	**1.6**	1.4	**1.4**
HIP2	huntingtin interacting protein 2	-1.3		-1.3		-1.6	**-1.6**	-1.2	**-1.2**
HIPK3	homeodomain interacting protein kinase 3	1.2		2.1		1.5	**1.3**	1.5	**1.5**
HMBS	hydroxymethylbilane synthase	-1.3		-1.6		-1.6	**-1.6**	-1.7	
HMGCS1	3-hydroxy-3-methylglutaryl-Coenzyme A synthase 1 (soluble)	-2.3	**-2.3**	-1.5	**-1.5**	-3.5	**-3.5**	-1.5	
HNRPAB	heterogeneous nuclear ribonucleoprotein A/B	-1.5	**-1.5**	-1.4	**-1.4**	-1.4	**-1.4**	-1.6	**-1.6**
HNRPU	heterogeneous nuclear ribonucleoprotein U (scaffold attachment factor A)	-1.3		-1.3		-1.4	**-1.4**	-1.4	**-1.4**
HRMT1L2	protein arginine methyltransferase 1	-1.5		-1.7	**-1.7**	-1.2	**-1.2**	-2.6	**-2.6**
HS6ST1	heparan sulfate 6-O-sulfotransferase 1	1.3		2.2	**2.2**	1.3	**1.3**	1.8	**1.8**
IARS	isoleucine-tRNA synthetase	-1.6	**-1.6**	-1.7		-1.4	**-1.4**	-2.2	**-2.2**
ICAM2	intercellular adhesion molecule 2	-1.8	**-1.8**	-1.7	**-1.7**	-17.5	**-17.5**	-2.4	**-2.4**
IDH3A	isocitrate dehydrogenase 3 (NAD+) alpha	-1.5	**-1.5**	-1.5	**-1.5**	-1.6	**-1.6**	-1.7	**-1.7**
IFNGR1	interferon gamma receptor 1	1.7	**1.7**	1.2		5.7	**5.7**	3.3	**3.3**

Additional file 7: T-cell ALL vs. B-cell ALL. (Pediatric and Adult)									
Page 4									
GC-response		Sensitive	Sensitive	Sensitive	Sensitive	Sensitive	Sensitive	Sensitive	Sensitive
Patient-derived cell line		Pediatric	Pediatric	Pediatric	Pediatric	Adult	Adult	Pediatric	Pediatric
Cell lineage		T-cell	T-cell	T-cell	T-cell	B-cell	B-cell	B-cell	B-cell
Sub-type of leukemia		ALL	ALL	ALL	ALL	ALL	ALL	ALL	ALL
Name	Description	C7-14 Dx	C7-14 Dx	C1-6 Dx	C1-6 Dx	RS4 Dx	RS4 Dx	SUP Dx	SUP Dx
			Stat. sign		Stat. sign		Stat. sign		Stat. sign
IGLL1	immunoglobulin lambda-like polypeptide 1	-1.4	**-1.4**	-1.3		-44.6	**-44.6**	-1.2	**-1.2**
INPP1	inositol polyphosphate-1-phosphatase	3.5	**3.5**	17.6	**17.6**	1.4	**1.4**	2.7	**2.7**
ITGA6	integrin, alpha 6	5.0	**5.0**	3.5	**2.4**	6.1	**6.1**	7.2	**7.2**
ITPR2	inositol 1,4,5-triphosphate receptor, type 2	1.5	**1.5**	1.5	**1.5**	4.3	**4.3**	1.8	**1.8**
JAK1	Janus kinase 1 (a protein tyrosine kinase)	2.2	**2.2**	2.6	**2.6**	1.9	**1.9**	1.8	**1.8**
KATNB1	katanin p80 (WD repeat containing) subunit B 1	-1.4		-1.5		-1.4	**-1.4**	-2.1	**-2.1**
KIAA0020	KIAA0020	-1.7	**-1.7**	-2.2	**-2.2**	-1.9	**-1.9**	-2.0	**-2.0**
KIAA0133	KIAA0133	-1.5		-1.3		-1.5	**-1.5**	-1.4	**-1.4**
KIF2	kinesin heavy chain member 2A	-1.2	**-1.2**	-1.3		-1.3	**-1.3**	-1.3	
LAIR1	leukocyte-associated immunoglobulin-like receptor 1	1.7	**1.7**	1.6	**1.6**	6.7	**6.7**	2.7	**2.7**
LRP8	low density lipoprotein receptor-related protein 8, apolipoprotein e receptor	-2.2	**-2.2**	-2.8	**-2.8**	-1.6	**-1.6**	-2.1	**-2.1**
LSM7	LSM7 homolog, U6 small nuclear RNA associated (S. cerevisiae)	-1.2		-1.4		-1.5	**-1.5**	-1.5	**-1.5**
M11S1	GPI-anchored membrane protein 1	-1.2		-1.3		-2.0	**-2.0**	-1.3	**-1.3**
MAP2K1	mitogen-activated protein kinase kinase 1	1.5	**1.5**	1.7	**1.7**	1.5	**1.5**	1.9	**1.9**
MARCKSL1	MARCKS-like 1	-1.2		-1.6	**-1.6**	-1.6	**-1.6**	-1.4	**-1.4**
MARS	methionine-tRNA synthetase	-1.5		-1.5		-1.5	**-1.5**	-2.2	**-2.2**
MEP50	WD repeat domain 77	-1.7		-1.8		-1.8	**-1.8**	-1.5	**-1.5**
MGC17330	HGFL gene	7.3	**7.3**	5.5	**5.5**	34.8	**34.8**	7.3	**7.3**
MGC5508	transmembrane protein 109	-1.4	**-1.4**	-1.6	**-1.6**	-1.4	**-1.4**	-1.7	**-1.7**
MPHOSPH6	M-phase phosphoprotein 6	-1.2		-1.4		-1.5	**-1.3**	-1.9	**-1.9**
MSN	moesin	1.2		1.2		1.4	**1.4**	1.4	**1.2**
MT1H	metallothionein 1H	1.3		2.1	**2.1**	1.4	**1.4**	3.0	**3.0**
MT1X	metallothionein 1X	1.4		2.2		1.6	**1.6**	1.8	**1.8**
MTM1	myotubularin 1	1.6		1.5		2.5	**2.5**	1.4	
MYCBP2	MYC binding protein 2	-1.2		-1.3		-1.5	**-1.5**	-1.4	**-1.4**
NCK1	NCK adaptor protein 1	1.5	**1.4**	2.3	**2.3**	1.7	**1.7**	1.5	**1.5**
NCL	nucleolin	-1.3	**-1.3**	-1.2		-1.6	**-1.6**	-1.9	**-1.9**
NDRG1	N-myc downstream regulated gene 1	1.6	**1.6**	1.4		9.2	**9.2**	3.1	**3.1**
NDUFAF1	NADH dehydrogenase (ubiquinone) 1 alpha subcomplex, assembly factor 1	-1.8		-1.5	**-1.2**	-3.0	**-3.0**	-1.8	**-1.8**

Additional file 7: T-cell ALL vs. B-cell ALL. (Pediatric and Adult)									
Page 5									
GC-response		Sensitive	Sensitive	Sensitive	Sensitive	Sensitive	Sensitive	Sensitive	Sensitive
Patient-derived cell line		Pediatric	Pediatric	Pediatric	Pediatric	Adult	Adult	Pediatric	Pediatric
Cell lineage		T-cell	T-cell	T-cell	T-cell	B-cell	B-cell	B-cell	B-cell
Sub-type of leukemia		ALL	ALL	ALL	ALL	ALL	ALL	ALL	ALL
**Name**	**Description**	C7-14 Dx	C7-14 Dx	C1-6 Dx	C1-6 Dx	RS4 Dx	RS4 Dx	SUP Dx	SUP Dx
			Stat. sign		Stat. sign		Stat. sign		Stat. sign
NF2	neurofibromin 2 (bilateral acoustic neuroma)	-1.3		-1.2		-1.3	**-1.3**	-1.4	
NFATC3	nuclear factor of activated T-cells, cytoplasmic, calcineurin-dependent 3	-1.4	**-1.4**	-1.9	**-1.9**	-1.8	**-1.8**	-1.4	**-1.4**
NFKBIA	nuclear factor of kappa light polypeptide gene enhancer in B-cells inhibitor, alpha	3.0	**3.0**	2.8	**2.8**	1.9	**1.9**	2.5	**2.5**
NOL1	nucleolar protein 1, 120kDa	-1.4		-1.6		-1.4		-1.5	**-1.5**
NOLA2	nucleolar protein family A, member 2 (H/ACA small nucleolar RNPs)	-1.3		-1.6		-1.5	**-1.5**	-1.8	**-1.8**
NUP153	nucleoporin 153kDa	-1.5	**-1.5**	-1.7		-2.0	**-2.0**	-1.4	**-1.4**
NUP62	nucleoporin 62kDa	-1.4	**-1.4**	-1.3		-1.4	**-1.4**	-1.4	
NUP98	nucleoporin 98kDa	-1.2		-1.3	**-1.3**	-1.7	**-1.7**	-2.0	**-2.0**
ODC1	ornithine decarboxylase 1	-2.1	**-2.1**	-2.2	**-2.2**	-1.2	**-1.2**	-1.5	**-1.5**
OGT	O-linked N-acetylglucosamine (GlcNAc) transferase (UDP-N-acetylglucosamine:polypeptide-N-acetylglucosaminyl transferase)	1.3		2.0	**2.0**	2.0	**2.0**	1.3	**1.3**
PA2G4	proliferation-associated 2G4, 38kDa	-1.7	**-1.7**	-1.7		-1.3		-2.5	**-2.5**
PAI-RBP1	SERPINE1 mRNA binding protein 1	-1.6	**-1.6**	-1.6		-1.5	**-1.5**	-1.9	**-1.9**
PARD3	par-3 partitioning defective 3 homolog (C. elegans)	1.6		1.4		2.5	**2.5**	1.5	
PER2	period homolog 2 (Drosophila)	-1.6		-1.6		-2.0	**-2.0**	-1.7	**-1.7**
PFAS	phosphoribosylformylglycinamidine synthase (FGAR amidotransferase)	-1.4		-1.2		-2.4	**-2.4**	-2.7	**-2.7**
PICALM	phosphatidylinositol binding clathrin assembly protein	1.7	**1.7**	1.5	**1.5**	2.1	**2.1**	2.0	**2.0**
PIK3R1	phosphoinositide-3-kinase, regulatory subunit 1 (p85 alpha)	2.5	**2.5**	1.7		2.1	**2.1**	1.4	
PLCB1	phospholipase C, beta 1 (phosphoinositide-specific)	-1.3		-1.2		-7.4	**-7.4**	-1.5	**-1.5**
POLE2	polymerase (DNA directed), epsilon 2 (p59 subunit)	-1.2		-1.7		-1.8	**-1.8**	-2.1	**-2.1**
POLR2D	polymerase (RNA) II (DNA directed) polypeptide D	-1.3		-1.8	**-1.8**	-1.2		-1.3	
POLR2I	polymerase (RNA) II (DNA directed) polypeptide I, 14.5kDa	-1.4		-1.5		-1.6	**-1.6**	-1.8	**-1.8**
PON2	paraoxonase 2	1.4	**1.4**	1.3		43.3	**43.3**	19.7	**19.7**
PPP2R1B	--	-1.5		-1.4		-1.7	**-1.7**	-1.4	**-1.4**
PRDX1	peroxiredoxin 1	-1.3	**-1.3**	-1.4		-2.5	**-2.5**	-1.7	**-1.7**
PRG1	proteoglycan 1, secretory granule	2.6		3.2	**3.2**	2.7	**2.7**	1.5	**1.5**
PRMT3	protein arginine methyltransferase 3	-1.6	**-1.6**	-2.1	**-2.1**	-1.3	**-1.3**	-2.4	**-2.4**
PRPS1	phosphoribosyl pyrophosphate synthetase 1	-1.5	**-1.5**	-1.4		-2.1	**-2.1**	-2.6	**-2.6**
PSEN1	presenilin 1 (Alzheimer disease 3)	1.7	**1.7**	1.5	**1.5**	1.3	**1.3**	1.5	**1.5**
PTP4A1	protein tyrosine phosphatase type IVA, member 1	-1.4		-1.4		-1.7	**-1.7**	-1.8	**-1.8**

Additional file 7: T-cell ALL vs. B-cell ALL. (Pediatric and Adult)									
Page 6									
GC-response		Sensitive	Sensitive	Sensitive	Sensitive	Sensitive	Sensitive	Sensitive	Sensitive
Patient-derived cell line		Pediatric	Pediatric	Pediatric	Pediatric	Adult	Adult	Pediatric	Pediatric
Cell lineage		T-cell	T-cell	T-cell	T-cell	B-cell	B-cell	B-cell	B-cell
Sub-type of leukemia		ALL	ALL	ALL	ALL	ALL	ALL	ALL	ALL
Name	Description	C7-14 Dx	C7-14 Dx	C1-6 Dx	C1-6 Dx	RS4 Dx	RS4 Dx	SUP Dx	SUP Dx
			Stat. sign		Stat. sign		Stat. sign		Stat. sign
PTS	6-pyruvoyltetrahydropterin synthase	-1.6	**-1.6**	-1.5		-2.4	**-2.4**	-1.3	**-1.3**
RAD23A	RAD23 homolog A (S. cerevisiae)	-1.4	**-1.2**	-1.3		-1.2		-1.4	**-1.4**
RAG1	recombination activating gene 1	-3.6	**-3.6**	-4.2	**-4.2**	-32.2	**-32.2**	-1.3	
RALBP1	ralA binding protein 1	-1.2		-1.6		-1.8	**-1.8**	-1.4	**-1.4**
RANBP1	RAN binding protein 1	-1.3		-1.5		-1.9	**-1.9**	-1.6	
RAPGEF2	Rap guanine nucleotide exchange factor (GEF) 2	1.7		1.4		1.8	**1.8**	2.0	**2.0**
RASA1	RAS p21 protein activator (GTPase activating protein) 1	1.9	**1.9**	2.1	**2.0**	1.9	**1.9**	2.3	**2.3**
RBBP8	retinoblastoma binding protein 8	-1.3	**-1.3**	-1.3		-1.9	**-1.9**	-1.7	**-1.7**
RBL2	retinoblastoma-like 2 (p130)	1.3		1.7		1.5	**1.5**	1.5	**1.5**
RBM8A	RNA binding motif protein 8A	1.2		1.5		2.3	**2.3**	1.5	**1.5**
RBMS1	RNA binding motif, single stranded interacting protein 1	1.4		1.7		3.0	**3.0**	4.3	**4.3**
RCP9	calcitonin gene-related peptide-receptor component protein	-1.2		-1.2		-1.5	**-1.5**	-1.5	**-1.5**
RDH11	retinol dehydrogenase 11 (all-trans/9-cis/11-cis)	-1.3		-1.4		-1.3	**-1.3**	-1.8	**-1.8**
REL	v-rel reticuloendotheliosis viral oncogene homolog (avian)	1.6		2.4	**2.4**	2.4	**2.4**	1.5	**1.5**
RFC3	replication factor C (activator 1) 3, 38kDa	-1.3		-1.3		-2.1	**-2.1**	-1.5	**-1.5**
RGL2	ral guanine nucleotide dissociation stimulator-like 2	1.6	**1.6**	1.6		1.3		1.2	
RGS19	regulator of G-protein signalling 19	-1.3	**-1.3**	-1.6	**-1.6**	-1.8	**-1.8**	-1.3	**-1.3**
RRAS	related RAS viral (r-ras) oncogene homolog	1.3		1.6		1.7	**1.7**	2.6	**2.6**
RSL1D1	ribosomal L1 domain containing 1	-1.4		-1.4		-3.8	**-3.8**	-2.1	**-2.1**
RUNX1	--	-1.3	**-1.3**	-1.4		-1.5	**-1.5**	-2.5	**-2.5**
SACS	spastic ataxia of Charlevoix-Saguenay (sacsin)	-1.5		-2.0	**-2.0**	-1.7	**-1.7**	-1.4	**-1.4**
SAP30	Sin3A-associated protein, 30kDa	2.2	**2.2**	1.7		1.7	**1.7**	2.3	**2.3**
SARS	seryl-tRNA synthetase	-1.4	**-1.4**	-1.3		-2.7	**-2.7**	-1.4	**-1.4**
SCARB1	scavenger receptor class B, member 1	-1.7	**-1.7**	-1.2		-1.4	**-1.4**	-4.9	**-4.9**
SENP3	SUMO1/sentrin/SMT3 specific peptidase 3	-1.3	**-1.3**	-1.2	**-1.2**	-2.9	**-2.9**	-1.7	**-1.7**
SEPT6	septin 6	-1.3		-1.6	**-1.6**	-1.4	**-1.4**	-2.0	**-2.0**
SFRS2	splicing factor, arginine/serine-rich 2	-1.2		-1.2		-1.3	**-1.3**	-1.4	**-1.4**
SIVA	SIVA1, apoptosis-inducing factor	-1.4		-1.3		-1.6	**-1.6**	-1.7	**-1.7**
SLA	Src-like-adaptor	2.8	**2.8**	3.1	**3.1**	2.1	**2.1**	4.4	**4.4**

Additional file 7: T-cell ALL vs. B-cell ALL. (Pediatric and Adult)									
Page 7									
GC-response		Sensitive	Sensitive	Sensitive	Sensitive	Sensitive	Sensitive	Sensitive	Sensitive
Patient-derived cell line		Pediatric	Pediatric	Pediatric	Pediatric	Adult	Adult	Pediatric	Pediatric
Cell lineage		T-cell	T-cell	T-cell	T-cell	B-cell	B-cell	B-cell	B-cell
Sub-type of leukemia		ALL	ALL	ALL	ALL	ALL	ALL	ALL	ALL
Name	Description	C7-14 Dx	C7-14 Dx	C1-6 Dx	C1-6 Dx	RS4 Dx	RS4 Dx	SUP Dx	SUP Dx
			Stat. sign		Stat. sign		Stat. sign		Stat. sign
SLC16A1	solute carrier family 16, member 1 (monocarboxylic acid transporter 1)	-1.3		-1.5		-1.2	**-1.2**	-2.8	**-2.8**
SLC18A2	solute carrier family 18 (vesicular monoamine), member 2	4.1	**4.1**	4.2	**4.2**	4.3	**4.3**	1.7	**1.6**
SLC29A1	solute carrier family 29 (nucleoside transporters), member 1	-1.7	**-1.7**	-1.5		-1.3		-2.6	**-2.6**
SLC39A6	solute carrier family 39 (zinc transporter), member 6	-1.4		-1.5	**-1.5**	-1.7	**-1.7**	-1.7	**-1.7**
SLC7A1	solute carrier family 7 (cationic amino acid transporter, y+ system), member 1	-1.7		-2.1	**-2.1**	-2.4	**-2.4**	-2.7	**-2.7**
SMARCA3	helicase-like transcription factor	-1.3		-1.3		-1.4	**-1.4**	-1.4	**-1.4**
SMARCA4	SWI/SNF related, matrix associated, actin dependent regulator of chromatin, subfamily a, member 4	-1.3		-1.6		-1.9	**-1.9**	-1.6	**-1.6**
SNAPC1	small nuclear RNA activating complex, polypeptide 1, 43kDa	-1.4		-1.5		-1.3		-1.5	**-1.5**
SNRPA1	small nuclear ribonucleoprotein polypeptide A'	-1.3		-1.5		-1.4	**-1.4**	-1.4	**-1.4**
SORD	sorbitol dehydrogenase	-1.4		-1.5		-1.3	**-1.3**	-1.6	**-1.6**
SOX4	SRY (sex determining region Y)-box 4	-1.2		-1.9		-5.7	**-5.7**	-1.5	**-1.3**
SPAG9	sperm associated antigen 9	1.4		1.3		2.0	**2.0**	1.3	**1.3**
SRD5A1	steroid-5-alpha-reductase, alpha polypeptide 1 (3-oxo-5 alpha-steroid delta 4-dehydrogenase alpha 1)	2.7		3.0	**3.0**	2.0	**2.0**	1.2	**1.2**
SSBP1	single-stranded DNA binding protein 1	-1.3		-1.4		-1.4	**1.3**	-1.8	**-1.8**
SSR1	signal sequence receptor, alpha (translocon-associated protein alpha)	1.2		1.2		1.2	**1.2**	1.2	
STAT2	signal transducer and activator of transcription 2, 113kDa	1.4		1.4		1.3		1.2	**1.2**
STIP1	stress-induced-phosphoprotein 1 (Hsp70/Hsp90-organizing protein)	-1.8	**-1.8**	-1.6		-1.6	**-1.6**	-1.4	**-1.4**
STS	steroid sulfatase (microsomal), arylsulfatase C, isozyme S	-1.6	**-1.6**	-1.4		-3.6	**-3.6**	-1.3	
STX3A	syntaxin 3	1.3		1.2		1.7	**1.7**	2.1	
SV2A	synaptic vesicle glycoprotein 2A	1.7	**1.7**	1.4	**1.4**	10.1	**10.1**	1.9	**1.9**
SYNCRIP	synaptotagmin binding, cytoplasmic RNA interacting protein	-1.3		-1.4	**-1.4**	-1.7	**-1.7**	-1.6	**-1.6**
TBC1D4	TBC1 domain family, member 4	-1.4		-1.3	**-1.3**	-1.4		-1.8	**-1.8**
TCP1	t-complex 1	-1.4		-1.7	**-1.7**	-1.3	**-1.3**	-1.5	**-1.5**
TFAM	transcription factor A, mitochondrial	-1.3		-1.8	**-1.8**	-1.4	**-1.4**	-1.8	**-1.8**
TFPI	tissue factor pathway inhibitor (lipoprotein-associated coagulation inhibitor)	3.1	**3.1**	1.9	**1.9**	4.9	**4.9**	9.6	**9.6**
TIMM17A	translocase of inner mitochondrial membrane 17 homolog A (yeast)	-1.7		-1.6	**-1.5**	-1.2	**-1.2**	-1.5	**-1.5**
TNFAIP8	tumor necrosis factor, alpha-induced protein 8	-1.3	**-1.3**	-1.7		-1.5	**-1.5**	-1.9	**-1.9**
TOMM40	translocase of outer mitochondrial membrane 40 homolog (yeast)	-1.5		-1.3		-1.2		-1.4	**-1.4**
TPP1	tripeptidyl peptidase I	1.2		1.3		1.7	**1.7**	1.4	**1.4**

Additional file 7: T-cell ALL vs. B-cell ALL. (Pediatric and Adult)									
Page 8									
GC-response		Sensitive	Sensitive	Sensitive	Sensitive	Sensitive	Sensitive	Sensitive	Sensitive
Patient-derived cell line		Pediatric	Pediatric	Pediatric	Pediatric	Adult	Adult	Pediatric	Pediatric
Cell lineage		T-cell	T-cell	T-cell	T-cell	B-cell	B-cell	B-cell	B-cell
Sub-type of leukemia		ALL	ALL	ALL	ALL	ALL	ALL	ALL	ALL
Name	Description	C7-14 Dx	C7-14 Dx	C1-6 Dx	C1-6 Dx	RS4 Dx	RS4 Dx	SUP Dx	SUP Dx
			Stat. sign		Stat. sign		Stat. sign		Stat. sign
TPST2	tyrosylprotein sulfotransferase 2	1.3		1.3		-1.7	**-1.7**	2.0	**2.0**
TRAF4	TNF receptor-associated factor 4	-1.7	**-1.7**	-1.5	**-1.5**	-13.8	**-13.8**	-1.7	**-1.7**
TRAP1	TNF receptor-associated protein 1	-1.6	**-1.6**	-1.6		-1.3		-1.9	**-1.9**
TSC22D3	TSC22 domain family, member 3	33.1	**33.1**	20.4	**20.4**	5.2	**5.2**	17.5	**17.5**
TSFM	Ts translation elongation factor, mitochondrial	-1.5	**-1.5**	-1.6	**-1.6**	-1.2	**-1.2**	-2.5	**-2.5**
TSNAX	translin-associated factor X	2.1	**2.1**	2.2	**2.2**	1.2	**1.2**	1.9	**1.9**
TSR1	TSR1, 20S rRNA accumulation, homolog (S. cerevisiae)	-2.0	**-2.0**	-2.0	**-2.0**	-2.0	**-2.0**	-2.4	**-2.4**
TUFM	Tu translation elongation factor, mitochondrial	-1.2		-1.3	**-1.3**	-1.5	**-1.5**	-1.5	**-1.5**
TXNIP	thioredoxin interacting protein	2.8	**2.8**	3.7	**3.7**	10.0	**10.0**	7.7	**7.7**
TXNRD1	thioredoxin reductase 1	-1.3		-1.4	**-1.4**	-1.3	**-1.3**	-1.3	
UBE2L6	ubiquitin-conjugating enzyme E2L 6	-1.2	**-1.2**	-1.3	**-1.3**	-2.0	**-2.0**	-1.7	**-1.7**
UBE2N	ubiquitin-conjugating enzyme E2N (UBC13 homolog, yeast)	-1.2		-1.2		-1.4	**-1.4**	-1.3	**-1.3**
UBE2S	ubiquitin-conjugating enzyme E2S	-1.6		-1.3		-1.2		-2.1	**-2.1**
UBTF	upstream binding transcription factor, RNA polymerase I	-1.3	**-1.3**	-1.3		-1.4	**-1.2**	-1.3	**-1.3**
UMPS	uridine monophosphate synthetase (orotate phosphoribosyl transferase and orotidine-5'-decarboxylase)	-1.3	**-1.3**	-1.4		-1.3	**-1.3**	-1.3	
UQCRC2	ubiquinol-cytochrome c reductase core protein II	-1.2		-1.5		-1.6	**-1.6**	-1.2	
VARS	valyl-tRNA synthetase	-1.5		-1.3		-1.3		-2.1	**-2.1**
VPS26	vacuolar protein sorting 26 homolog A (yeast)	1.3		1.2		1.5	**1.5**	1.3	**1.3**
YAF2	YY1 associated factor 2	1.8	**1.8**	1.6	**1.6**	1.7	**1.7**	1.9	**1.9**
YARS	tyrosyl-tRNA synthetase	-1.6		-1.3		-1.9	**-1.9**	-2.6	**-2.6**
ZFP36L2	zinc finger protein 36, C3H type-like 2	3.0	**3.0**	2.5	**2.5**	2.2	**2.2**	1.8	**1.8**
ZHX3	zinc fingers and homeoboxes 3	1.3		1.3		2.0	**2.0**	2.0	**2.0**
ZNF259	zinc finger protein 259	-1.4	**-1.4**	-1.8	**-1.8**	-1.2	**-1.2**	-1.3	
ZNF263	zinc finger protein 263	-1.2		-1.6		-1.7	**-1.7**	-1.7	**-1.7**
ZNF364	zinc finger protein 364	1.2		1.2		1.3	**1.3**	1.8	**1.8**
