# Supplementary material for: Gene expression profiling of leukemic cells and primary thymocytes predicts a signature for apoptotic sensitivity to glucocorticoids
Source: Cancer Cell Int. 2007 Nov 28;7:18. doi: 10.1186/1475-2867-7-18 (PMC2228275; doi:10.1186/1475-2867-7-18)
Supplement: Additional file 8 — Pediatric T-cell ALL vs. Pediatric B-cell ALL. Genes regulated in common in the same sense by Dex in T-cell ALL CEM-C7–14, CEM-C1–6, and B-cell SUP-B15 cells. Bold type indicates statistically significant regulation p ≤ 0.05 between means of vehicle vs. GC-treated. [file 1475-2867-7-18-S8.pdf]

Additional file 8: T-cell ALL vs. B-cell ALL. (Pediatric only)							
Page 1							
GC-response		Sensitive	Sensitive	Sensitive	Sensitive	Sensitive	Sensitive
Patient-derived cell line		Pediatric	Pediatric	Pediatric	Pediatric	Pediatric	Pediatric
Cell lineage		T-cell	T-cell	T-cell	T-cell	B-cell	B-cell
Sub-type of leukemia		ALL	ALL	ALL	ALL	ALL	ALL
Name	Description	C7-14 Dx	C7-14 Dx	C1-6 Dx	C1-6 Dx	SUP Dx	SUP Dx
			Stat. sign		Stat. sign		Stat. sign
AARS	alanyl-tRNA synthetase	-1.3	**-1.3**	-1.2		-1.9	**-1.9**
AASDHPPT	aminoadipate-semialdehyde dehydrogenase-phosphopantetheinyl transferase	-1.4		-1.7		-1.3	**-1.3**
ABCE1	ATP-binding cassette, sub-family E (OABP), member 1	-1.5	**-1.5**	-1.7		-2.0	**-2.0**
ABI1	abl-interactor 1	1.3		1.3		1.7	**1.7**
ACLY	ATP citrate lyase	-1.3		-1.2		-1.7	**-1.7**
ACSL3	acyl-CoA synthetase long-chain family member 3	-1.3		-1.3		-1.3	**-1.3**
ADAM9	ADAM metallopeptidase domain 9 (meltrin gamma)	1.3		1.3		1.6	**1.6**
ADRBK2	adrenergic, beta, receptor kinase 2	-1.4		-1.3		-1.5	**-1.5**
AHCY	S-adenosylhomocysteine hydrolase	-1.3		-1.4		-1.4	**-1.4**
AHCYL1	S-adenosylhomocysteine hydrolase-like 1	-1.3		-1.2		-1.4	**-1.4**
AHSA1	AHA1, activator of heat shock 90kDa protein ATPase homolog 1 (yeast)	-1.3		-1.3		-1.7	**-1.7**
AK2	adenylate kinase 2	-1.4		-1.6	**-1.6**	-2.2	**-2.2**
AKAP1	A kinase (PRKA) anchor protein 1	-1.6	**-1.6**	-1.9		-2.4	**-2.4**
ANKRD40	ankyrin repeat domain 40	-1.4		-1.2		-1.5	
AP1S2	adaptor-related protein complex 1, sigma 2 subunit	1.8	**1.8**	1.2		1.6	**1.6**
AP3S1	adaptor-related protein complex 3, sigma 1 subunit	1.6	**1.6**	1.9	**1.9**	2.4	**2.4**
APG12L	ATG12 autophagy related 12 homolog (S. cerevisiae)	1.6	**1.6**	1.9	**1.9**	1.9	**1.9**
APPBP1	amyloid beta precursor protein binding protein 1	-1.2		-1.5		-1.3	**-1.3**
APPBP2	amyloid beta precursor protein (cytoplasmic tail) binding protein 2	1.3		1.7		1.4	**1.4**
ARHGEF18	rho/rac guanine nucleotide exchange factor (GEF) 18	1.3	**1.3**	1.4		2.5	**2.5**
ARHGEF7	Rho guanine nucleotide exchange factor (GEF) 7	1.3		1.3		3.6	**3.6**
ARL1	ADP-ribosylation factor-like 1	-1.2	**-1.2**	-1.6		-1.4	
ATF4	activating transcription factor 4 (tax-responsive enhancer element B67)	-1.5	**-1.5**	-1.3		-1.4	**-1.4**
ATF5	activating transcription factor 5	-1.5		-1.3		-2.0	**-2.0**
ATIC	5-aminoimidazole-4-carboxamide ribonucleotide formyltransferase/IMP cyclohydrolase	-1.4	**-1.4**	-2.0	**-2.0**	-3.2	**-3.2**
ATP11B	ATPase, Class VI, type 11B	1.3		1.5	**1.5**	5.1	**5.1**
ATP2B1	ATPase, Ca++ transporting, plasma membrane 1	-1.4		-2.0		-2.1	**-2.1**
ATP5G1	ATP synthase, H+ transporting, mitochondrial F0 complex, subunit C1 (subunit 9)	-1.2		-1.3	**-1.3**	-2.4	**-2.4**
ATP6V0D1	ATPase, H+ transporting, lysosomal 38kDa, V0 subunit d1	1.3	**1.3**	1.3		1.2	

Additional file 8: T-cell ALL vs. B-cell ALL. (Pediatric only)							
Page 2							
GC-response		Sensitive	Sensitive	Sensitive	Sensitive	Sensitive	Sensitive
Patient-derived cell line		Pediatric	Pediatric	Pediatric	Pediatric	Pediatric	Pediatric
Cell lineage		T-cell	T-cell	T-cell	T-cell	B-cell	B-cell
Sub-type of leukemia		ALL	ALL	ALL	ALL	ALL	ALL
Name	Description	C7-14 Dx	C7-14 Dx	C1-6 Dx	C1-6 Dx	SUP Dx	SUP Dx
			Stat. sign		Stat. sign		Stat. sign
ATXN2	ataxin 2	-1.4		-1.3		-1.3	
AUH	AU RNA binding protein/enoyl-Coenzyme A hydratase	2.5	**2.5**	1.7	**1.7**	1.4	
BAG2	BCL2-associated athanogene 2	-1.2		-1.6		-1.8	**-1.8**
BCL2L11	BCL2-like 11 (apoptosis facilitator)	3.1	**3.1**	5.9	**5.9**	3.1	**3.1**
BCLAF1	BCL2-associated transcription factor 1	-1.3	**-1.3**	-1.5		-1.7	**-1.7**
BDH1	3-hydroxybutyrate dehydrogenase, type 1	-1.3	**-1.3**	-1.5	**-1.5**	-1.7	
BIRC2	baculoviral IAP repeat-containing 2	1.9	**1.9**	1.5		1.9	**1.9**
BOP1	block of proliferation 1	-1.4	**-1.4**	-1.6		-2.6	**-2.6**
BTG1	B-cell translocation gene 1, anti-proliferative	9.3	**9.3**	4.4	**4.4**	3.9	**3.9**
BTG2	BTG family, member 2	3.1	**3.1**	2.3	**2.3**	3.9	**3.9**
BYSL	bystin-like	-2.2	**-2.2**	-2.3	**-2.3**	-8.4	**-8.4**
C19ORF2	chromosome 19 open reading frame 2	-1.3		-1.2		-1.3	**-1.3**
C1QBP	complement component 1, q subcomponent binding protein	-1.8	**-1.8**	-2.0		-1.9	**-1.9**
C5ORF13	chromosome 5 open reading frame 13	-1.3	**-1.3**	-1.5		-3.3	**-3.3**
CAD	carbamoyl-phosphate synthetase 2, aspartate transcarbamylase, and dihydroorotase	-1.3		-1.6		-2.8	**-2.8**
CALCOCO1	calcium binding and coiled-coil domain 1	1.6		1.8		1.3	
CALR	calreticulin	-1.9	**-1.9**	-1.6		-1.5	**-1.4**
CANX	calnexin	-1.3		-1.3		-1.3	**-1.3**
CAPN7	calpain 7	1.4		1.3		1.5	
CASP4	caspase 4, apoptosis-related cysteine peptidase	1.5	**1.5**	1.3		2.0	**2.0**
CASP8	caspase 8, apoptosis-related cysteine peptidase	1.3		1.3		1.5	**1.5**
CBX3	chromobox homolog 3 (HP1 gamma homolog, Drosophila)	-1.2		-1.2		-1.8	**1.2**
CCDC6	coiled-coil domain containing 6	1.3		1.6		1.9	**1.9**
CCDC85B	coiled-coil domain containing 85B	-1.4		-1.3		-1.8	**-1.8**
CCNF	cyclin F	1.3		1.6		2.0	**2.0**
CCNG2	cyclin G2	2.3	**2.3**	1.3		2.2	**2.2**
CCT2	chaperonin containing TCP1, subunit 2 (beta)	-1.3	**-1.3**	-1.3		-2.2	**-2.2**
CCT3	chaperonin containing TCP1, subunit 3 (gamma)	-1.3		-1.4		-1.6	**-1.6**
CCT5	chaperonin containing TCP1, subunit 5 (epsilon)	-1.6	**-1.6**	-1.7		-1.5	**-1.5**

Additional file 8: T-cell ALL vs. B-cell ALL. (Pediatric only)							
Page 3							
GC-response		Sensitive	Sensitive	Sensitive	Sensitive	Sensitive	Sensitive
Patient-derived cell line		Pediatric	Pediatric	Pediatric	Pediatric	Pediatric	Pediatric
Cell lineage		T-cell	T-cell	T-cell	T-cell	B-cell	B-cell
Sub-type of leukemia		ALL	ALL	ALL	ALL	ALL	ALL
Name	Description	C7-14 Dx	C7-14 Dx	C1-6 Dx	C1-6 Dx	SUP Dx	SUP Dx
			Stat. sign		Stat. sign		Stat. sign
CCT6A	chaperonin containing TCP1, subunit 6A (zeta 1)	-1.6	**-1.6**	-1.7	**-1.7**	-1.4	
CCT7	chaperonin containing TCP1, subunit 7 (eta)	-1.4		-1.6	**-1.6**	-1.5	**-1.5**
CCT8	chaperonin containing TCP1, subunit 8 (theta)	-1.7		-1.6		-1.6	**-1.6**
CD164	CD164 molecule, sialomucin	1.3		1.5		1.8	**1.8**
CD44	CD44 molecule (Indian blood group)	-1.7	**-1.7**	-1.9		-3.0	**-3.0**
CD53	CD53 molecule	3.1	**3.1**	3.2	**3.2**	3.1	**3.1**
CD59	CD59 molecule, complement regulatory protein	1.3		1.8	**1.8**	1.3	
CD69	CD69 molecule	4.9	**4.9**	2.0	**2.0**	3.8	**3.8**
CD79A	CD79a molecule, immunoglobulin-associated alpha	2.0	**2.0**	3.6	**3.6**	1.3	**1.3**
CD99	CD99 molecule	1.2		1.8	**1.8**	3.4	**3.4**
CDC123	cell division cycle 123 homolog (S. cerevisiae)	-1.3	**-1.3**	-1.6		-1.7	**-1.7**
CDC25A	cell division cycle 25 homolog A (S. cerevisiae)	-1.4	**-1.4**	-1.4		-1.4	**-1.4**
CDC2L6	cell division cycle 2-like 6 (CDK8-like)	1.6		1.8	**1.8**	3.3	**3.3**
CDC6	cell division cycle 6 homolog (S. cerevisiae)	-1.3	**-1.3**	-1.5	**-1.5**	-2.9	**-2.9**
CDH4	cadherin 4, type 1, R-cadherin (retinal)	1.3		1.5		2.0	**2.0**
CDK4	cyclin-dependent kinase 4	-1.4	**-1.4**	-1.6	**-1.6**	-2.4	**-2.4**
CDKN2C	cyclin-dependent kinase inhibitor 2C (p18, inhibits CDK4)	1.2		1.3		1.5	**1.3**
CEBPB	CCAAT/enhancer binding protein (C/EBP), beta	-1.4		-1.4		-1.5	**-1.5**
CEBPZ	CCAAT/enhancer binding protein zeta	-1.5	**-1.5**	-1.8	**-1.8**	-2.6	**-1.5**
CENTB2	centaurin, beta 2	1.3		1.3	**1.3**	1.5	**1.5**
CHC1	regulator of chromosome condensation 1	-1.7	**-1.7**	-1.6	**-1.6**	-1.8	**-1.8**
CLK1	CDC-like kinase 1	1.2		1.3		1.7	**1.7**
COMT	catechol-O-methyltransferase	-1.4		-1.4		-1.4	
COPS2	COP9 constitutive photomorphogenic homolog subunit 2 (Arabidopsis)	-1.2		-1.3		-1.2	**-1.2**
CPEB3	cytoplasmic polyadenylation element binding protein 3	1.4		1.3		3.0	**3.0**
CPSF5	nudix (nucleoside diphosphate linked moiety X)-type motif 21	-1.3		-1.2		-1.3	**-1.3**
CRADD	CASP2 and RIPK1 domain containing adaptor with death domain	1.3		1.4		1.5	**1.5**
CSE1L	CSE1 chromosome segregation 1-like (yeast)	-1.3		-1.4		-1.4	**-1.4**
CSNK2A1	casein kinase 2, alpha 1 polypeptide	-1.4		-1.3		-1.6	**-1.4**

Additional file 8: T-cell ALL vs. B-cell ALL. (Pediatric only)							
Page 4							
**GC-response**		Sensitive	Sensitive	Sensitive	Sensitive	Sensitive	Sensitive
**Patient-derived cell line**		Pediatric	Pediatric	Pediatric	Pediatric	Pediatric	Pediatric
**Cell lineage**		T-cell	T-cell	T-cell	T-cell	B-cell	B-cell
**Sub-type of leukemia**		ALL	ALL	ALL	ALL	ALL	ALL
**Name**	**Description**	C7-14 Dx	C7-14 Dx	C1-6 Dx	C1-6 Dx	SUP Dx	SUP Dx
			Stat. sign		Stat. sign		Stat. sign
CTBP1	C-terminal binding protein 1	1.3	**1.3**	1.3	**1.3**	1.3	**1.3**
CTPS	CTP synthase	-1.7		-1.8		-1.4	**-1.4**
CTSC	--	-1.5	**-1.5**	-1.4		-5.2	**-5.2**
CUGBP2	CUG triplet repeat, RNA binding protein 2	2.1	**2.0**	1.8	**1.8**	2.4	**2.4**
CXCR4	chemokine (C-X-C motif) receptor 4	1.3	**1.3**	1.5	**1.5**	1.8	**1.8**
CYC1	cytochrome c-1	-1.3	**-1.3**	-1.5		-1.6	**-1.6**
CYCS	cytochrome c, somatic	-1.6	**-1.6**	-1.9	**-1.9**	-1.6	**-1.6**
DCTD	dCMP deaminase	-1.3	**-1.3**	-1.3		-1.2	
DCTN3	dynactin 3 (p22)	1.3	**1.3**	1.3		1.3	**1.3**
DDIT4	DNA-damage-inducible transcript 4	4.4	**4.4**	2.5		4.6	**4.6**
DDX10	DEAD (Asp-Glu-Ala-Asp) box polypeptide 10	-1.2		-1.5		-2.1	**-2.1**
DDX18	DEAD (Asp-Glu-Ala-Asp) box polypeptide 18	-1.5		-1.6		-1.7	**-1.7**
DDX21	DEAD (Asp-Glu-Ala-Asp) box polypeptide 21	-1.7	**-1.7**	-2.0		-2.1	**-2.1**
DGUOK	deoxyguanosine kinase	-1.3	**-1.3**	-1.3	**-1.3**	-1.3	
DHCR7	7-dehydrocholesterol reductase	-1.8		-1.5		-1.7	**-1.7**
DHODH	dihydroorotate dehydrogenase	-1.9		-2.0		-1.5	**-1.5**
DHRS1	dehydrogenase/reductase (SDR family) member 1	1.7	**1.7**	1.4		1.5	
DHX30	DEAH (Asp-Glu-Ala-His) box polypeptide 30	-1.3		-1.3		-1.7	**-1.7**
DKC1	dyskeratosis congenita 1, dyskerin	-1.7	**-1.7**	-1.3	**-1.3**	-2.6	**-2.6**
DLAT	dihydrolipoamide S-acetyltransferase (E2 component of pyruvate dehydrogenase complex)	-1.3		-1.5		-2.1	**-2.1**
DLG1	discs, large homolog 1 (Drosophila)	-1.3		1.3		-1.9	**-1.9**
DNAJA1	DnaJ (Hsp40) homolog, subfamily A, member 1	-1.3	**-1.3**	-1.7	**-1.7**	-1.4	**-1.4**
DNPEP	aspartyl aminopeptidase	-1.2	**-1.2**	-1.4		-1.6	**-1.6**
DPEP1	dipeptidase 1 (renal)	1.9	**1.9**	4.0	**4.0**	13.0	**13.0**
DSCR1	Down syndrome critical region gene 1	5.0	**5.0**	4.8	**4.8**	7.7	**7.7**
DUSP7	dual specificity phosphatase 7	-2.0		-2.0	**-2.0**	-1.2	**-1.2**
E2F5	E2F transcription factor 5, p130-binding	-1.6	**-1.6**	-1.7		-1.8	
EBNA1BP2	EBNA1 binding protein 2	-1.5	**-1.5**	-2.0		-1.8	**-1.8**
EEF1E1	eukaryotic translation elongation factor 1 epsilon 1	-1.7	**-1.7**	-2.3		-3.3	**-3.3**

Additional file 8: T-cell ALL vs. B-cell ALL. (Pediatric only)							
Page 5							
GC-response		Sensitive	Sensitive	Sensitive	Sensitive	Sensitive	Sensitive
Patient-derived cell line		Pediatric	Pediatric	Pediatric	Pediatric	Pediatric	Pediatric
Cell lineage		T-cell	T-cell	T-cell	T-cell	B-cell	B-cell
Sub-type of leukemia		ALL	ALL	ALL	ALL	ALL	ALL
Name	Description	C7-14 Dx	C7-14 Dx	C1-6 Dx	C1-6 Dx	SUP Dx	SUP Dx
			Stat. sign		Stat. sign		Stat. sign
EIF2C2	eukaryotic translation initiation factor 2C, 2	-2.1		-1.9		-1.6	**-1.6**
EIF2S1	eukaryotic translation initiation factor 2, subunit 1 alpha, 35kDa	-1.4	**-1.4**	-1.4	**-1.4**	-1.6	**-1.6**
EIF3S10	eukaryotic translation initiation factor 3, subunit 10 theta, 150/170kDa	-1.4		-1.7	**-1.7**	-1.3	**-1.3**
EIF3S8	eukaryotic translation initiation factor 3, subunit 8, 110kDa	-1.2	**-1.2**	-1.2		-1.6	**-1.4**
EIF3S9	eukaryotic translation initiation factor 3, subunit 9 eta, 116kDa	-1.5	**-1.5**	-1.3		-2.0	**-2.0**
EIF4A1	eukaryotic translation initiation factor 4A, isoform 1	-1.7	**-1.4**	-1.3		-1.8	**-1.8**
EIF5	eukaryotic translation initiation factor 5	-1.3		-1.3		-1.2	
ENO1	enolase 1, (alpha)	-1.3	**-1.3**	-1.5		-1.5	**-1.5**
ESPL1	extra spindle pole bodies homolog 1 (S. cerevisiae)	1.3	**1.3**	1.2		1.3	**1.3**
ETV6	ets variant gene 6 (TEL oncogene)	-1.4		-1.6		-1.6	**-1.6**
EXOSC2	exosome component 2	-1.7	**-1.7**	-1.2		-2.4	**-2.4**
FADS1	fatty acid desaturase 1	-1.5		-1.9	**-1.9**	-1.5	**-1.5**
FADS2	fatty acid desaturase 2	-1.4		-1.3		-2.0	**-2.0**
FARSLA	phenylalanine-tRNA synthetase-like, alpha subunit	-1.9	**-1.9**	-2.0	**-2.0**	-2.1	**-2.1**
FBL	fibrillarin	-1.4	**-1.4**	-1.4	**-1.4**	-1.2	**-1.2**
FH	fumarate hydratase	-1.2		-1.2		-2.1	**-2.1**
FHL1	four and a half LIM domains 1	3.3	**3.3**	13.7	**13.7**	1.8	**1.8**
FKBP5	FK506 binding protein 5	6.6	**6.6**	4.4	**4.4**	21.4	**21.4**
FLI1	Friend leukemia virus integration 1	1.9		1.4		1.7	**1.6**
FLT1	fms-related tyrosine kinase 1 (vascular endothelial growth factor/vascular permeability factor receptor)	1.4		3.8	**3.8**	2.6	**2.6**
FNBP1L	formin binding protein 1-like	1.9	**1.9**	1.8		3.0	**3.0**
FOXO3A	forkhead box O3A	1.3		1.2		2.7	**2.7**
FZD6	frizzled homolog 6 (Drosophila)	1.5	**1.5**	1.3		2.1	**2.1**
G3BP	GTPase activating protein (SH3 domain) binding protein 1	-1.3		-1.6		-2.2	**-2.2**
GARS	glycyl-tRNA synthetase	-1.4	**-1.4**	-1.7	**-1.7**	-2.1	**-2.1**
GART	phosphoribosylglycinamide formyltransferase, phosphoribosylglycinamide synthetase, phosphoribosylaminoimidazole synthetase	-1.6	**-1.6**	-1.6		-2.4	**-2.4**
GCSH	glycine cleavage system protein H (aminomethyl carrier)	-1.6	**-1.6**	-1.7		-3.0	**-3.0**
GLO1	glyoxalase I	-1.3		-1.5		-1.5	**-1.5**
GLRX	glutaredoxin (thioltransferase)	3.4	**3.4**	2.1		1.6	**1.6**

**Additional file 8: T-cell ALL vs. B-cell ALL. (Pediatric only)**							
**Page 6**							
**GC-response**		**Sensitive**	**Sensitive**	**Sensitive**	**Sensitive**	**Sensitive**	**Sensitive**
**Patient-derived cell line**		**Pediatric**	**Pediatric**	**Pediatric**	**Pediatric**	**Pediatric**	**Pediatric**
**Cell lineage**		**T-cell**	**T-cell**	**T-cell**	**T-cell**	**B-cell**	**B-cell**
**Sub-type of leukemia**		**ALL**	**ALL**	**ALL**	**ALL**	**ALL**	**ALL**
**Name**	**Description**	**C7-14 Dx**	**C7-14 Dx**	**C1-6 Dx**	**C1-6 Dx**	**SUP Dx**	**SUP Dx**
			**Stat. sign**		**Stat. sign**		**Stat. sign**
GLUL	glutamate-ammonia ligase (glutamine synthetase)	1.9	**1.9**	3.4		2.5	**2.5**
GM2A	GM2 ganglioside activator	1.3	**1.3**	1.5		1.5	
GMPS	guanine monphosphate synthetase	-1.3		-1.4		-1.4	**-1.4**
GPR56	G protein-coupled receptor 56	1.3		1.2		25.3	**25.3**
GSK3B	glycogen synthase kinase 3 beta	1.6		1.2		1.5	**1.3**
GSPT1	G1 to S phase transition 1	-1.5	**-1.5**	-1.8	**-1.8**	-1.2	**-1.2**
GTF2E2	general transcription factor IIE, polypeptide 2, beta 34kDa	-1.4	**-1.4**	-1.3		-1.3	**-1.3**
GTF3A	general transcription factor IIIA	-1.3		-1.5		-1.4	**-1.4**
H1F0	H1 histone family, member 0	1.4		2.5	**2.5**	2.4	**2.4**
HAX1	HCLS1 associated protein X-1	-1.2		-1.3	**-1.3**	-1.7	**-1.7**
HBP1	HMG-box transcription factor 1	1.5		1.6	**1.6**	1.4	**1.4**
HDAC2	histone deacetylase 2	-1.3		-1.5	**-1.5**	-1.5	**-1.5**
HIP2	huntingtin interacting protein 2	-1.3		-1.3		-1.2	**-1.2**
HIPK3	homeodomain interacting protein kinase 3	1.2		2.1		1.5	**1.5**
HLA-G	HLA-G histocompatibility antigen, class I, G	1.5	**1.5**	1.3	**1.3**	1.5	**1.5**
HMBS	hydroxymethylbilane synthase	-1.3		-1.6		-1.7	
HMGCS1	3-hydroxy-3-methylglutaryl-Coenzyme A synthase 1 (soluble)	-2.3	**-2.3**	-1.5	**-1.5**	-1.5	
HNRPAB	heterogeneous nuclear ribonucleoprotein A/B	-1.5	**-1.5**	-1.4	**-1.4**	-1.6	**-1.6**
HNRPDL	heterogeneous nuclear ribonucleoprotein D-like	-1.4		-1.2		-1.6	**-1.6**
HNRPU	heterogeneous nuclear ribonucleoprotein U (scaffold attachment factor A)	-1.3		-1.3		-1.4	**-1.4**
HRB2	KRR1, small subunit (SSU) processome component, homolog (yeast)	-1.4		-1.7		-1.3	**-1.3**
HRMT1L2	protein arginine methyltransferase 1	-1.5		-1.7	**-1.7**	-2.6	**-2.6**
HS6ST1	heparan sulfate 6-O-sulfotransferase 1	1.3		2.2	**2.2**	1.8	**1.8**
HSPA8	heat shock 70kDa protein 8	-1.3	**-1.3**	-1.9	**-1.9**	-1.3	**-1.3**
HSPA9B	heat shock 70kDa protein 9 (mortalin)	-1.5		-1.6	**-1.6**	-2.8	**-2.8**
HSPCB	heat shock protein 90kDa alpha (cytosolic), class B member 1	-1.5	**-1.4**	-1.3	**-1.3**	-2.0	**-2.0**
HSPD1	heat shock 60kDa protein 1 (chaperonin)	-1.4	**-1.4**	-1.3		-3.0	**-3.0**
HSPE1	heat shock 10kDa protein 1 (chaperonin 10)	-1.8	**-1.8**	-2.1	**-2.1**	-2.2	**-2.2**
IARS	isoleucine-tRNA synthetase	-1.6	**-1.6**	-1.7		-2.2	**-2.2**

Additional file 8: T-cell ALL vs. B-cell ALL. (Pediatric only)							
Page 7							
GC-response		Sensitive	Sensitive	Sensitive	Sensitive	Sensitive	Sensitive
Patient-derived cell line		Pediatric	Pediatric	Pediatric	Pediatric	Pediatric	Pediatric
Cell lineage		T-cell	T-cell	T-cell	T-cell	B-cell	B-cell
Sub-type of leukemia		ALL	ALL	ALL	ALL	ALL	ALL
Name	Description	C7-14 Dx	C7-14 Dx	C1-6 Dx	C1-6 Dx	SUP Dx	SUP Dx
			Stat. sign		Stat. sign		Stat. sign
IARS2	isoleucine-tRNA synthetase 2, mitochondrial	-1.2		-1.5		-1.2	**-1.2**
ICAM2	intercellular adhesion molecule 2	-1.8	**-1.8**	-1.7	**-1.7**	-2.4	**-2.4**
IDH3A	isocitrate dehydrogenase 3 (NAD+) alpha	-1.5	**-1.5**	-1.5	**-1.5**	-1.7	**-1.7**
IFNGR1	interferon gamma receptor 1	1.7	**1.7**	1.2		3.3	**3.3**
IFRD1	interferon-related developmental regulator 1	-1.8	**-1.8**	-1.6	**-1.3**	-1.3	
IFRD2	interferon-related developmental regulator 2	-1.8	**-1.8**	-1.7	**-1.7**	-3.4	**-3.4**
IGHM	immunoglobulin heavy constant mu	1.4		1.6		1.4	**1.4**
IGLL1	immunoglobulin lambda-like polypeptide 1	-1.4	**-1.4**	-1.3		-1.2	**-1.2**
ILF3	interleukin enhancer binding factor 3, 90kDa	-1.4	**-1.3**	-1.3		-2.2	**-2.2**
IMPDH2	IMP (inosine monophosphate) dehydrogenase 2	-1.3		-1.5	**-1.5**	-1.4	**-1.4**
INPP1	inositol polyphosphate-1-phosphatase	3.5	**3.5**	17.6	**17.6**	2.7	**2.7**
IPO7	importin 7	-1.4	**-1.4**	-1.3		-1.7	**-1.7**
IQGAP1	IQ motif containing GTPase activating protein 1	1.3		1.3		2.2	**2.2**
IQGAP2	IQ motif containing GTPase activating protein 2	1.4	**1.4**	1.9	**1.9**	2.6	**2.6**
IQSEC1	IQ motif and Sec7 domain 1	1.6		1.9		1.8	**1.8**
ISG20	interferon stimulated exonuclease gene 20kDa	9.5	**9.5**	7.6	**7.6**	91.9	**91.9**
ITGA6	integrin, alpha 6	5.0	**5.0**	3.5	**2.4**	7.2	**7.2**
ITM1	STT3, subunit of the oligosaccharyltransferase complex, homolog A (S. cerevisiae)	-1.2		-1.3	**-1.3**	-1.3	
ITPR2	inositol 1,4,5-triphosphate receptor, type 2	1.5	**1.5**	1.5	**1.5**	1.8	**1.8**
JAK1	Janus kinase 1 (a protein tyrosine kinase)	2.2	**2.2**	2.6	**2.6**	1.8	**1.8**
JTV1	JTV1 gene	-1.9	**-1.9**	-2.1		-2.3	**-2.3**
KARS	lysyl-tRNA synthetase	-1.3	**-1.3**	-1.5	**-1.5**	-1.6	**-1.6**
KATNB1	katanin p80 (WD repeat containing) subunit B 1	-1.4		-1.5		-2.1	**-2.1**
KCNA5	potassium voltage-gated channel, shaker-related subfamily, member 5	1.4		1.4		5.9	**5.9**
KIAA0020	KIAA0020	-1.7	**-1.7**	-2.2	**-2.2**	-2.0	**-2.0**
KIAA0133	KIAA0133	-1.5		-1.3		-1.4	**-1.4**
KIF14	kinesin family member 14	1.3		1.3		1.4	**1.4**
KIF2	kinesin heavy chain member 2A	-1.2	**-1.2**	-1.3		-1.3	
KIR3DL2	killer cell immunoglobulin-like receptor, three domains, long cytoplasmic tail, 2	1.8	**1.8**	3.0	**3.0**	1.5	**1.5**

Additional file 8: T-cell ALL vs. B-cell ALL. (Pediatric only)							
**Page 8**							
**GC-response**		**Sensitive**	**Sensitive**	**Sensitive**	**Sensitive**	**Sensitive**	**Sensitive**
**Patient-derived cell line**		**Pediatric**	**Pediatric**	**Pediatric**	**Pediatric**	**Pediatric**	**Pediatric**
**Cell lineage**		**T-cell**	**T-cell**	**T-cell**	**T-cell**	**B-cell**	**B-cell**
**Sub-type of leukemia**		**ALL**	**ALL**	**ALL**	**ALL**	**ALL**	**ALL**
**Name**	**Description**	**C7-14 Dx**	**C7-14 Dx**	**C1-6 Dx**	**C1-6 Dx**	**SUP Dx**	**SUP Dx**
			**Stat. sign**		**Stat. sign**		**Stat. sign**
KNS2	kinesin 2	1.2		1.3		1.8	**1.8**
LAIR1	leukocyte-associated immunoglobulin-like receptor 1	1.7	**1.7**	1.6	**1.6**	2.7	**2.7**
LARP1	La ribonucleoprotein domain family, member 1	-1.4		-1.9		-2.0	**-2.0**
LGALS9	lectin, galactoside-binding, soluble, 9 (galectin 9)	-1.5	**-1.5**	-2.1	**-2.1**	-1.5	
LRCH4	leucine-rich repeats and calponin homology (CH) domain containing 4	1.3		1.7		1.9	**1.9**
LRP8	low density lipoprotein receptor-related protein 8, apolipoprotein e receptor	-2.2	**-2.2**	-2.8	**-2.8**	-2.1	**-2.1**
LRPPRC	leucine-rich PPR-motif containing	-1.8	**-1.8**	-1.7	**-1.7**	-5.1	**-5.1**
LSM7	LSM7 homolog, U6 small nuclear RNA associated (S. cerevisiae)	-1.2		-1.4		-1.5	**-1.5**
LSS	lanosterol synthase (2,3-oxidosqualene-lanosterol cyclase)	-1.5	**-1.5**	-1.3		-1.2	**-1.2**
LTB	lymphotoxin beta (TNF superfamily, member 3)	-1.6		-1.8		-1.8	
M11S1	GPI-anchored membrane protein 1	-1.2		-1.3		-1.3	**-1.3**
MAC30	transmembrane protein 97	-1.3	**-1.3**	-1.4		-3.0	**-3.0**
MAP1LC3B	microtubule-associated protein 1 light chain 3 beta	1.2		1.3		3.2	**3.2**
MAP2K1	mitogen-activated protein kinase kinase 1	1.5	**1.5**	1.7	**1.7**	1.9	**1.9**
MAP4	microtubule-associated protein 4	-1.3	**-1.3**	-1.7		-1.4	
MAPKAPK2	mitogen-activated protein kinase-activated protein kinase 2	1.3	**1.3**	1.5		1.5	**1.5**
MAPKAPK3	mitogen-activated protein kinase-activated protein kinase 3	-1.4	**-1.4**	-1.5		-1.5	**-1.5**
MARCKSL1	MARCKS-like 1	-1.2		-1.6	**-1.6**	-1.4	**-1.4**
MARS	methionine-tRNA synthetase	-1.5		-1.5		-2.2	**-2.2**
ME2	malic enzyme 2, NAD(+)-dependent, mitochondrial	-1.5		-1.8	**-1.8**	-1.6	**-1.6**
MEP50	WD repeat domain 77	-1.7		-1.8		-1.5	**-1.5**
MFGE8	milk fat globule-EGF factor 8 protein	1.3		1.3		1.7	
MGC17330	HGFL gene	7.3	**7.3**	5.5	**5.5**	7.3	**7.3**
MGC5508	transmembrane protein 109	-1.4	**-1.4**	-1.6	**-1.6**	-1.7	**-1.7**
MLH3	mutL homolog 3 (E. coli)	-1.4		-1.4		-1.3	**-1.3**
MPHOSPH10	M-phase phosphoprotein 10 (U3 small nucleolar ribonucleoprotein)	-1.4		-1.5		-1.3	**-1.3**
MPHOSPH6	M-phase phosphoprotein 6	-1.2		-1.4		-1.9	**-1.9**
MSN	moesin	1.2		1.2		1.4	**1.2**
MT1H	metallothionein 1H	1.3		2.1	**2.1**	3.0	**3.0**

Additional file 8: T-cell ALL vs. B-cell ALL. (Pediatric only)							
Page 9							
GC-response		Sensitive	Sensitive	Sensitive	Sensitive	Sensitive	Sensitive
Patient-derived cell line		Pediatric	Pediatric	Pediatric	Pediatric	Pediatric	Pediatric
Cell lineage		T-cell	T-cell	T-cell	T-cell	B-cell	B-cell
Sub-type of leukemia		ALL	ALL	ALL	ALL	ALL	ALL
Name	Description	C7-14 Dx	C7-14 Dx	C1-6 Dx	C1-6 Dx	SUP Dx	SUP Dx
			Stat. sign		Stat. sign		Stat. sign
MT1X	metallothionein 1X	1.4		2.2		1.8	**1.8**
MTHFD1	methylenetetrahydrofolate dehydrogenase (NADP+ dependent) 1, methenyltetrahydrofolate cyclohydrolase, formyltetrahydrofolate synthetase	-1.4	**-1.4**	-1.5	**-1.5**	-2.2	**-2.2**
MTHFD2	methylenetetrahydrofolate dehydrogenase (NADP+ dependent) 2, methenyltetrahydrofolate cyclohydrolase	-2.1	**-2.1**	-1.7	**-1.7**	-2.0	**-2.0**
MTM1	myotubularin 1	1.6		1.5		1.4	
MTRR	5-methyltetrahydrofolate-homocysteine methyltransferase reductase	-1.7		-1.4		-1.2	**-1.2**
MXI1	MAX interactor 1	-2.7	**-2.7**	-1.6		-1.7	**-1.7**
MYC	v-myc myelocytomatosis viral oncogene homolog (avian)	-3.8	**-3.8**	-3.6	**-3.6**	-2.8	**-2.8**
MYCBP2	MYC binding protein 2	-1.2		-1.3		-1.4	**-1.4**
MYH9	myosin, heavy chain 9, non-muscle	1.4		1.6	**1.6**	1.3	
NAP1L1	nucleosome assembly protein 1-like 1	-1.3	**-1.3**	-1.2		-1.3	**-1.2**
NARS	asparaginyl-tRNA synthetase	-1.6	**-1.6**	-1.4		-1.4	**-1.4**
NCBP2	nuclear cap binding protein subunit 2, 20kDa	-1.6	**-1.6**	-1.5		-1.2	
NCK1	NCK adaptor protein 1	1.5	**1.4**	2.3	**2.3**	1.5	**1.5**
NCL	nucleolin	-1.3	**-1.3**	-1.2		-1.9	**-1.9**
NDRG1	N-myc downstream regulated gene 1	1.6	**1.6**	1.4		3.1	**3.1**
NDUFAF1	NADH dehydrogenase (ubiquinone) 1 alpha subcomplex, assembly factor 1	-1.8		-1.5	**-1.2**	-1.8	**-1.8**
NF2	neurofibromin 2 (bilateral acoustic neuroma)	-1.3		-1.2		-1.4	
NFATC3	nuclear factor of activated T-cells, cytoplasmic, calcineurin-dependent 3	-1.4	**-1.4**	-1.9	**-1.9**	-1.4	**-1.4**
NFIL3	nuclear factor, interleukin 3 regulated	6.0	**6.0**	3.6	**3.6**	5.0	**5.0**
NFKBIA	nuclear factor of kappa light polypeptide gene enhancer in B-cells inhibitor, alpha	3.0	**3.0**	2.8	**2.8**	2.5	**2.5**
NME1	non-metastatic cells 1, protein (NM23A) expressed in	-1.5	**-1.5**	-1.7	**-1.7**	-4.4	**-4.4**
NME4	non-metastatic cells 4, protein expressed in	-1.3		-1.6		-2.1	**-2.1**
NOC2L	nucleolar complex associated 2 homolog (S. cerevisiae)	-1.5		-1.3	**-1.3**	-1.8	**-1.8**
NOL1	nucleolar protein 1, 120kDa	-1.4		-1.6		-1.5	**-1.5**
NOL5A	nucleolar protein 5A (56kDa with KKE/D repeat)	-1.9	**-1.9**	-1.8		-2.0	**-2.0**
NOLA2	nucleolar protein family A, member 2 (H/ACA small nucleolar RNPs)	-1.3		-1.6		-1.8	**-1.8**
NOLC1	nucleolar and coiled-body phosphoprotein 1	-2.1	**-2.1**	-2.0		-2.0	**-2.0**
NP	nucleoside phosphorylase	-1.8	**-1.8**	-2.0	**-2.0**	-1.3	**-1.3**
NPM3	nucleophosmin/nucleoplasmin, 3	-1.4	**-1.4**	-1.3		-1.7	

Additional file 8: T-cell ALL vs. B-cell ALL. (Pediatric only)							
Page 10							
GC-response		Sensitive	Sensitive	Sensitive	Sensitive	Sensitive	Sensitive
Patient-derived cell line		Pediatric	Pediatric	Pediatric	Pediatric	Pediatric	Pediatric
Cell lineage		T-cell	T-cell	T-cell	T-cell	B-cell	B-cell
Sub-type of leukemia		ALL	ALL	ALL	ALL	ALL	ALL
Name	Description	C7-14 Dx	C7-14 Dx	C1-6 Dx	C1-6 Dx	SUP Dx	SUP Dx
			Stat. sign		Stat. sign		Stat. sign
NR3C1	nuclear receptor subfamily 3, group C, member 1 (glucocorticoid receptor)	4.2	**4.2**	2.1	**2.1**	3.1	**3.1**
NUP153	nucleoporin 153kDa	-1.5	**-1.5**	-1.7		-1.4	**-1.4**
NUP62	nucleoporin 62kDa	-1.4	**-1.4**	-1.3		-1.4	
NUP98	nucleoporin 98kDa	-1.2		-1.3	**-1.3**	-2.0	**-2.0**
ODC1	ornithine decarboxylase 1	-2.1	**-2.1**	-2.2	**-2.2**	-1.5	**-1.5**
OGT	O-linked N-acetylglucosamine (GlcNAc) transferase (UDP-N-acetylglucosamine:polypeptide-N-acetylglucosaminyl transferase)	1.3		2.0	**2.0**	1.3	**1.3**
OS9	amplified in osteosarcoma	1.5	**1.5**	1.4	**1.4**	1.3	
P2RX5	purinergic receptor P2X, ligand-gated ion channel, 5	1.3		2.7	**2.7**	5.5	**5.5**
P53CSV	TP53 regulated inhibitor of apoptosis 1	-1.3		-1.3		-1.4	**-1.4**
PA2G4	proliferation-associated 2G4, 38kDa	-1.7	**-1.7**	-1.7		-2.5	**-2.5**
PAICS	phosphoribosylaminoimidazole carboxylase, phosphoribosylaminoimidazole succinocarboxamide synthetase	-1.7	**-1.7**	-1.7		-3.3	**-3.3**
PAI-RBP1	SERPINE1 mRNA binding protein 1	-1.6	**-1.6**	-1.6		-1.9	**-1.9**
PARD3	par-3 partitioning defective 3 homolog (C. elegans)	1.6		1.4		1.5	
PAWR	PRKC, apoptosis, WT1, regulator	-1.2	**-1.2**	-1.3		-2.4	**-2.4**
PAXIP1L	PAX interacting (with transcription-activation domain) protein 1	1.4	**1.4**	1.3	**1.3**	1.2	**1.2**
PDK1	pyruvate dehydrogenase kinase, isozyme 1	-1.8		-1.3		-1.3	
PER1	period homolog 1 (Drosophila)	1.3		1.9		1.3	
PER2	period homolog 2 (Drosophila)	-1.6		-1.6		-1.7	**-1.7**
PFAS	phosphoribosylformylglycinamidine synthase (FGAR amidotransferase)	-1.4		-1.2		-2.7	**-2.7**
PGK1	phosphoglycerate kinase 1	-1.3		-1.5		-1.9	**-1.9**
PHKB	phosphorylase kinase, beta	1.4		1.3		1.4	**1.4**
PICALM	phosphatidylinositol binding clathrin assembly protein	1.7	**1.7**	1.5	**1.5**	2.0	**2.0**
PIK3R1	phosphoinositide-3-kinase, regulatory subunit 1 (p85 alpha)	2.5	**2.5**	1.7		1.4	
PLCB1	phospholipase C, beta 1 (phosphoinositide-specific)	-1.3		-1.2		-1.5	**-1.5**
PMS1	PMS1 postmeiotic segregation increased 1 (S. cerevisiae)	-1.4		-1.3		-1.3	**-1.3**
PNN	pinin, desmosome associated protein	-1.3		-1.5	**-1.5**	-1.5	**-1.5**
POLE2	polymerase (DNA directed), epsilon 2 (p59 subunit)	-1.2		-1.7		-2.1	**-2.1**
POLR1C	polymerase (RNA) I polypeptide C, 30kDa	-1.6		-1.7		-1.7	**-1.7**
POLR2D	polymerase (RNA) II (DNA directed) polypeptide D	-1.3		-1.8	**-1.8**	-1.3	

Additional file 8: T-cell ALL vs. B-cell ALL. (Pediatric only)							
Page 11							
GC-response		Sensitive	Sensitive	Sensitive	Sensitive	Sensitive	Sensitive
Patient-derived cell line		Pediatric	Pediatric	Pediatric	Pediatric	Pediatric	Pediatric
Cell lineage		T-cell	T-cell	T-cell	T-cell	B-cell	B-cell
Sub-type of leukemia		ALL	ALL	ALL	ALL	ALL	ALL
Name	Description	C7-14 Dx	C7-14 Dx	C1-6 Dx	C1-6 Dx	SUP Dx	SUP Dx
			Stat. sign		Stat. sign		Stat. sign
POLR2H	polymerase (RNA) II (DNA directed) polypeptide H	-1.6	**-1.6**	-1.4		-1.9	**-1.9**
POLR2I	polymerase (RNA) II (DNA directed) polypeptide I, 14.5kDa	-1.4		-1.5		-1.8	**-1.8**
POLR3G	polymerase (RNA) III (DNA directed) polypeptide G (32kD)	-1.7		-1.3		-3.0	**-3.0**
PON2	paraoxonase 2	1.4	**1.4**	1.3		19.7	**19.7**
PPARBP	PPAR binding protein	-1.3		-1.2		-1.5	**-1.5**
PPAT	phosphoribosyl pyrophosphate amidotransferase	-1.7	**-1.7**	-2.2		-2.0	**-2.0**
PPP2R1B	--	-1.5		-1.4		-1.4	**-1.4**
PRDX1	peroxiredoxin 1	-1.3	**-1.3**	-1.4		-1.7	**-1.7**
PRDX3	peroxiredoxin 3	-1.2		-1.6	**-1.6**	-1.8	**-1.8**
PRDX4	peroxiredoxin 4	-1.2	**-1.2**	-1.4		-2.5	**-2.5**
PRG1	proteoglycan 1, secretory granule	2.6		3.2	**3.2**	1.5	**1.5**
PRMT3	protein arginine methyltransferase 3	-1.6	**-1.6**	-2.1	**-2.1**	-2.4	**-2.4**
PRPS1	phosphoribosyl pyrophosphate synthetase 1	-1.5	**-1.5**	-1.4		-2.6	**-2.6**
PSEN1	presenilin 1 (Alzheimer disease 3)	1.7	**1.7**	1.5	**1.5**	1.5	**1.5**
PSMA2	proteasome (prosome, macropain) subunit, alpha type, 2	-1.3		-1.2		-1.3	**-1.3**
PSMA5	proteasome (prosome, macropain) subunit, alpha type, 5	-1.2		-1.4	**-1.4**	-1.4	**-1.4**
PSMB5	proteasome (prosome, macropain) subunit, beta type, 5	-1.4	**-1.4**	-1.4	**-1.4**	-1.4	**-1.4**
PSPH	phosphoserine phosphatase	-1.6		-1.9		-2.0	**-2.0**
PTP4A1	protein tyrosine phosphatase type IVA, member 1	-1.4		-1.4		-1.8	**-1.8**
PTPN11	protein tyrosine phosphatase, non-receptor type 11 (Noonan syndrome 1)	-1.6		-1.9		-1.6	
PTPN2	protein tyrosine phosphatase, non-receptor type 2	-1.6	**-1.3**	-2.1	**-2.1**	-1.2	**-1.2**
PTPN3	protein tyrosine phosphatase, non-receptor type 3	1.3		1.3		1.4	
PTS	6-pyruvoyltetrahydropterin synthase	-1.6	**-1.6**	-1.5		-1.3	**-1.3**
RAB9P40	Rab9 effector protein with kelch motifs	-1.8	**-1.8**	-2.1	**-2.1**	-3.7	**-3.7**
RABGGTB	Rab geranylgeranyltransferase, beta subunit	-1.3	**-1.3**	-1.7	**-1.7**	-1.8	**-1.8**
RAD17	RAD17 homolog (S. pombe)	-1.3		-1.4		-2.1	**-2.1**
RAD23A	RAD23 homolog A (S. cerevisiae)	-1.4	**-1.2**	-1.3		-1.4	**-1.4**
RAG1	recombination activating gene 1	-3.6	**-3.6**	-4.2	**-4.2**	-1.3	
RAI17	zinc finger, MIZ-type containing 1	-1.3		-1.5		-1.3	**-1.3**

Additional file 8: T-cell ALL vs. B-cell ALL. (Pediatric only)							
Page 12							
GC-response		Sensitive	Sensitive	Sensitive	Sensitive	Sensitive	Sensitive
Patient-derived cell line		Pediatric	Pediatric	Pediatric	Pediatric	Pediatric	Pediatric
Cell lineage		T-cell	T-cell	T-cell	T-cell	B-cell	B-cell
Sub-type of leukemia		ALL	ALL	ALL	ALL	ALL	ALL
Name	Description	C7-14 Dx	C7-14 Dx	C1-6 Dx	C1-6 Dx	SUP Dx	SUP Dx
			Stat. sign		Stat. sign		Stat. sign
RALA	v-ral simian leukemia viral oncogene homolog A (ras related)	-1.3		-1.2		-1.3	**-1.3**
RALBP1	ralA binding protein 1	-1.2		-1.6		-1.4	**-1.4**
RANBP1	RAN binding protein 1	-1.3		-1.5		-1.6	
RAPGEF2	Rap guanine nucleotide exchange factor (GEF) 2	1.7		1.4		2.0	**2.0**
RASA1	RAS p21 protein activator (GTPase activating protein) 1	1.9	**1.9**	2.1	**2.0**	2.3	**2.3**
RBBP8	retinoblastoma binding protein 8	-1.3	**-1.3**	-1.3		-1.7	**-1.7**
RBL2	retinoblastoma-like 2 (p130)	1.3		1.7		1.5	**1.5**
RBM13	RNA binding motif protein 13	-1.6	**-1.6**	-1.3	**-1.3**	-1.8	**-1.8**
RBM8A	RNA binding motif protein 8A	1.2		1.5		1.5	**1.5**
RBMS1	RNA binding motif, single stranded interacting protein 1	1.4		1.7		4.3	**4.3**
RCN1	reticulocalbin 1, EF-hand calcium binding domain	1.5	**1.5**	1.5		1.2	**1.2**
RCP9	calcitonin gene-related peptide-receptor component protein	-1.2		-1.2		-1.5	**-1.5**
RDH11	retinol dehydrogenase 11 (all-trans/9-cis/11-cis)	-1.3		-1.4		-1.8	**-1.8**
RECK	reversion-inducing-cysteine-rich protein with kazal motifs	2.2		1.4		2.9	**2.9**
REL	v-rel reticuloendotheliosis viral oncogene homolog (avian)	1.6		2.4	**2.4**	1.5	**1.5**
RELA	v-rel reticuloendotheliosis viral oncogene homolog A, nuclear factor of kappa light polypeptide gene enhancer in B-cells 3, p65 (avian)	-1.6	**-1.6**	-1.3		-1.2	
RFC3	replication factor C (activator 1) 3, 38kDa	-1.3		-1.3		-1.5	**-1.5**
RGL2	ral guanine nucleotide dissociation stimulator-like 2	1.6	**1.6**	1.6		1.2	
RGS19	regulator of G-protein signalling 19	-1.3	**-1.3**	-1.6	**-1.6**	-1.3	**-1.3**
RPIA	ribose 5-phosphate isomerase A (ribose 5-phosphate epimerase)	-1.3		-1.4		-1.7	**-1.7**
RPP38	ribonuclease P/MRP 38kDa subunit	-1.2	**-1.2**	-1.4		-1.5	**-1.5**
RPP40	ribonuclease P 40kDa subunit	-1.9	**-1.9**	-1.4		-1.9	**-1.9**
RRAS	related RAS viral (r-ras) oncogene homolog	1.3		1.6		2.6	**2.6**
RSL1D1	ribosomal L1 domain containing 1	-1.4		-1.4		-2.1	**-2.1**
RUNX1	--	-1.3	**-1.3**	-1.4		-2.5	**-2.5**
SACS	spastic ataxia of Charlevoix-Saguenay (sacsin)	-1.5		-2.0	**-2.0**	-1.4	**-1.4**
SAP30	Sin3A-associated protein, 30kDa	2.2	**2.2**	1.7		2.3	**2.3**
SARS	seryl-tRNA synthetase	-1.4	**-1.4**	-1.3		-1.4	**-1.4**
SATB1	special AT-rich sequence binding protein 1 (binds to nuclear matrix/scaffold-associating DNA's)	-1.9	**-1.9**	-2.3	**-2.3**	-1.8	**-1.8**

Additional file 8: T-cell ALL vs. B-cell ALL. (Pediatric only)							
Page 13							
GC-response		Sensitive	Sensitive	Sensitive	Sensitive	Sensitive	Sensitive
Patient-derived cell line		Pediatric	Pediatric	Pediatric	Pediatric	Pediatric	Pediatric
Cell lineage		T-cell	T-cell	T-cell	T-cell	B-cell	B-cell
Sub-type of leukemia		ALL	ALL	ALL	ALL	ALL	ALL
Name	Description	C7-14 Dx	C7-14 Dx	C1-6 Dx	C1-6 Dx	SUP Dx	SUP Dx
			Stat. sign		Stat. sign		Stat. sign
SCAMP1	secretory carrier membrane protein 1	-1.4		-1.4		-1.3	
SCAMP3	secretory carrier membrane protein 3	-1.4		-1.4		-1.3	
SCARB1	scavenger receptor class B, member 1	-1.7	**-1.7**	-1.2		-4.9	**-4.9**
SCD	stearoyl-CoA desaturase (delta-9-desaturase)	-2.0		-1.5		-3.7	**-3.7**
SCYE1	small inducible cytokine subfamily E, member 1 (endothelial monocyte-activating)	-1.7	**-1.7**	-1.9	**-1.9**	-1.4	**-1.4**
SDHB	succinate dehydrogenase complex, subunit B, iron sulfur (Ip)	-1.2		-1.6		-1.4	**-1.4**
SEC23IP	SEC23 interacting protein	-1.2		-1.7		-1.5	
SENP3	SUMO1/sentrin/SMT3 specific peptidase 3	-1.3	**-1.3**	-1.2	**-1.2**	-1.7	**-1.7**
SEPT6	septin 6	-1.3		-1.6	**-1.6**	-2.0	**-2.0**
SF3A3	splicing factor 3a, subunit 3, 60kDa	-1.3		-1.6	**-1.6**	-1.4	**-1.4**
SFPQ	splicing factor proline/glutamine-rich (polypyrimidine tract binding protein associated)	1.4		1.2		1.2	**1.2**
SFRS2	splicing factor, arginine/serine-rich 2	-1.2		-1.2		-1.4	**-1.4**
SIVA	SIVA1, apoptosis-inducing factor	-1.4		-1.3		-1.7	**-1.7**
SLA	Src-like-adaptor	2.8	**2.8**	3.1	**3.1**	4.4	**4.4**
SLC16A1	solute carrier family 16, member 1 (monocarboxylic acid transporter 1)	-1.3		-1.5		-2.8	**-2.8**
SLC18A2	solute carrier family 18 (vesicular monoamine), member 2	4.1	**4.1**	4.2	**4.2**	1.7	**1.6**
SLC19A1	solute carrier family 19 (folate transporter), member 1	-1.6	**-1.6**	-1.7	**-1.7**	-1.7	**-1.7**
SLC29A1	solute carrier family 29 (nucleoside transporters), member 1	-1.7	**-1.7**	-1.5		-2.6	**-2.6**
SLC39A14	solute carrier family 39 (zinc transporter), member 14	-1.4	**-1.4**	-1.3	**-1.3**	-3.8	**-3.8**
SLC39A6	solute carrier family 39 (zinc transporter), member 6	-1.4		-1.5	**-1.5**	-1.7	**-1.7**
SLC39A8	solute carrier family 39 (zinc transporter), member 8	-1.3		-1.4		-3.1	**-3.1**
SLC7A1	solute carrier family 7 (cationic amino acid transporter, y+ system), member 1	-1.7		-2.1	**-2.1**	-2.7	**-2.7**
SLC9A3R1	solute carrier family 9 (sodium/hydrogen exchanger), member 3 regulator 1	-1.2		-1.9	**-1.9**	-1.4	**-1.4**
SMARCA2	SWI/SNF related, matrix associated, actin dependent regulator of chromatin, subfamily a, member 2	1.7	**1.7**	1.4	**1.4**	2.2	**2.2**
SMARCA3	helicase-like transcription factor	-1.3		-1.3		-1.4	**-1.4**
SMARCA4	SWI/SNF related, matrix associated, actin dependent regulator of chromatin, subfamily a, member 4	-1.3		-1.6		-1.6	**-1.6**
SMARCC2	SWI/SNF related, matrix associated, actin dependent regulator of chromatin, subfamily c, member 2	1.3		1.3		1.4	
SMC4L1	structural maintenance of chromosomes 4	1.7	**1.7**	1.7	**1.7**	2.0	**1.4**
SMN1	survival of motor neuron 1, telomeric	-1.6	**-1.6**	-1.8		-1.2	

Additional file 8: T-cell ALL vs. B-cell ALL. (Pediatric only)							
Page 14							
GC-response		Sensitive	Sensitive	Sensitive	Sensitive	Sensitive	Sensitive
Patient-derived cell line		Pediatric	Pediatric	Pediatric	Pediatric	Pediatric	Pediatric
Cell lineage		T-cell	T-cell	T-cell	T-cell	B-cell	B-cell
Sub-type of leukemia		ALL	ALL	ALL	ALL	ALL	ALL
Name	Description	C7-14 Dx	C7-14 Dx	C1-6 Dx	C1-6 Dx	SUP Dx	SUP Dx
			Stat. sign		Stat. sign		Stat. sign
SNAPC1	small nuclear RNA activating complex, polypeptide 1, 43kDa	-1.4		-1.5		-1.5	**-1.5**
SNRPA1	small nuclear ribonucleoprotein polypeptide A'	-1.3		-1.5		-1.4	**-1.4**
SNTB2	syntrophin, beta 2 (dystrophin-associated protein A1, 59kDa, basic component 2)	3.0	**3.0**	2.3	**2.3**	3.5	**3.5**
SOCS1	suppressor of cytokine signaling 1	15.6	**15.6**	29.3	**29.3**	8.3	**8.3**
SORD	sorbitol dehydrogenase	-1.4		-1.5		-1.6	**-1.6**
SOX4	SRY (sex determining region Y)-box 4	-1.2		-1.9		-1.5	**-1.3**
SPA17	sperm autoantigenic protein 17	1.4		1.8		1.4	
SPAG9	sperm associated antigen 9	1.4		1.3		1.3	**1.3**
SRD5A1	steroid-5-alpha-reductase, alpha polypeptide 1 (3-oxo-5 alpha-steroid delta 4-dehydrogenase alpha 1)	2.7		3.0	**3.0**	1.2	**1.2**
SRM	spermidine synthase	-1.8	**-1.8**	-1.7	**-1.7**	-3.4	**-3.4**
SRP72	signal recognition particle 72kDa	-1.2		-1.6		-1.5	**-1.5**
SRPK1	SFRS protein kinase 1	-1.3		-1.7	**-1.7**	-1.5	**-1.5**
SRPK2	SFRS protein kinase 2	1.2		1.2		1.2	**1.2**
SSB	Sjogren syndrome antigen B (autoantigen La)	-1.3		-1.6	**-1.6**	-1.7	**-1.7**
SSBP1	single-stranded DNA binding protein 1	-1.3		-1.4		-1.8	**-1.8**
SSR1	signal sequence receptor, alpha (translocon-associated protein alpha)	1.2		1.2		1.2	
ST13	suppression of tumorigenicity 13 (colon carcinoma) (Hsp70 interacting protein)	-1.3		-1.2		-1.5	**-1.5**
STAT2	signal transducer and activator of transcription 2, 113kDa	1.4		1.4		1.2	**1.2**
STIM1	stromal interaction molecule 1	1.9	**1.9**	2.0	**2.0**	1.9	**1.9**
STIP1	stress-induced-phosphoprotein 1 (Hsp70/Hsp90-organizing protein)	-1.8	**-1.8**	-1.6		-1.4	**-1.4**
STS	steroid sulfatase (microsomal), arylsulfatase C, isozyme S	-1.6	**-1.6**	-1.4		-1.3	
STX3A	syntaxin 3	1.3		1.2		2.1	
STXBP1	syntaxin binding protein 1	2.0	**2.0**	3.8	**3.8**	1.2	
SV2A	synaptic vesicle glycoprotein 2A	1.7	**1.7**	1.4	**1.4**	1.9	**1.9**
SYNCRIP	synaptotagmin binding, cytoplasmic RNA interacting protein	-1.3		-1.4	**-1.4**	-1.6	**-1.6**
TARBP1	Tar (HIV-1) RNA binding protein 1	-1.6	**-1.6**	-1.8	**-1.8**	-1.3	
TAX1BP3	Tax1 (human T-cell leukemia virus type I) binding protein 3	1.6		1.4		3.3	**3.3**
TBC1D4	TBC1 domain family, member 4	-1.4		-1.3	**-1.3**	-1.8	**-1.8**
TBCD	tubulin folding cofactor D	1.7	**1.7**	3.4	**3.4**	2.4	**2.4**

Additional file 8: T-cell ALL vs. B-cell ALL. (Pediatric only)							
Page 15							
GC-response		Sensitive	Sensitive	Sensitive	Sensitive	Sensitive	Sensitive
Patient-derived cell line		Pediatric	Pediatric	Pediatric	Pediatric	Pediatric	Pediatric
Cell lineage		T-cell	T-cell	T-cell	T-cell	B-cell	B-cell
Sub-type of leukemia		ALL	ALL	ALL	ALL	ALL	ALL
Name	Description	C7-14 Dx	C7-14 Dx	C1-6 Dx	C1-6 Dx	SUP Dx	SUP Dx
			Stat. sign		Stat. sign		Stat. sign
TCFL1	vacuolar protein sorting 72 homolog (S. cerevisiae)	-1.3	**-1.3**	-1.5	**-1.5**	-1.3	**-1.3**
TCP1	t-complex 1	-1.4		-1.7	**-1.7**	-1.5	**-1.5**
TFAM	transcription factor A, mitochondrial	-1.3		-1.8	**-1.8**	-1.8	**-1.8**
TFDP2	transcription factor Dp-2 (E2F dimerization partner 2)	-1.6	**-1.5**	-1.5		-1.4	**-1.4**
TFPI	tissue factor pathway inhibitor (lipoprotein-associated coagulation inhibitor)	3.1	**3.1**	1.9	**1.9**	9.6	**9.6**
TGFBR2	transforming growth factor, beta receptor II (70/80kDa)	2.8	**2.8**	4.0	**4.0**	1.8	**1.8**
TGIF2	TGFB-induced factor 2 (TALE family homeobox)	-1.3		-1.4		-1.6	**-1.6**
TIMM17A	translocase of inner mitochondrial membrane 17 homolog A (yeast)	-1.7		-1.6	**-1.5**	-1.5	**-1.5**
TMF1	TATA element modulatory factor 1	-1.2	**-1.2**	-1.3		1.7	**1.7**
TMSB10	thymosin, beta 10	1.4	**1.4**	2.2	**2.2**	1.6	**1.6**
TNFAIP8	tumor necrosis factor, alpha-induced protein 8	-1.3	**-1.3**	-1.7		-1.9	**-1.9**
TOMM40	translocase of outer mitochondrial membrane 40 homolog (yeast)	-1.5		-1.3		-1.4	**-1.4**
TOMM70A	translocase of outer mitochondrial membrane 70 homolog A (S. cerevisiae)	-1.4	**-1.4**	-1.4		-1.5	**-1.5**
TOP2A	topoisomerase (DNA) II alpha 170kDa	1.4	**1.4**	1.2		1.8	**1.8**
TOX	thymus high mobility group box protein TOX	-1.3	**-1.3**	-1.3		-1.6	**-1.6**
TPP1	tripeptidyl peptidase I	1.2		1.3		1.4	**1.4**
TPST2	tyrosylprotein sulfotransferase 2	1.3		1.3		2.0	**2.0**
TRAF4	TNF receptor-associated factor 4	-1.7	**-1.7**	-1.5	**-1.5**	-1.7	**-1.7**
TRAM2	translocation associated membrane protein 2	1.5	**1.5**	1.7	**1.7**	2.4	**2.4**
TRAP1	TNF receptor-associated protein 1	-1.6	**-1.6**	-1.6		-1.9	**-1.9**
TSC22D3	TSC22 domain family, member 3	33.1	**33.1**	20.4	**20.4**	17.5	**17.5**
TSFM	Ts translation elongation factor, mitochondrial	-1.5	**-1.5**	-1.6	**-1.6**	-2.5	**-2.5**
TSNAX	translin-associated factor X	2.1	**2.1**	2.2	**2.2**	1.9	**1.9**
TSR1	TSR1, 20S rRNA accumulation, homolog (S. cerevisiae)	-2.0	**-2.0**	-2.0	**-2.0**	-2.4	**-2.4**
TTC3	tetratricopeptide repeat domain 3	-1.4		-1.2		-1.6	**-1.6**
TTLL12	tubulin tyrosine ligase-like family, member 12	-1.4	**-1.4**	-1.3		-2.7	**-2.7**
TUBA1	tubulin, alpha 1	10.4	**10.4**	3.0	**3.0**	2.1	**2.1**
TUFM	Tu translation elongation factor, mitochondrial	-1.2		-1.3	**-1.3**	-1.5	**-1.5**
TXNIP	thioredoxin interacting protein	2.8	**2.8**	3.7	**3.7**	7.7	**7.7**

Additional file 8: T-cell ALL vs. B-cell ALL. (Pediatric only)							
**Page 16**							
**GC-response**		**Sensitive**	**Sensitive**	**Sensitive**	**Sensitive**	**Sensitive**	**Sensitive**
**Patient-derived cell line**		**Pediatric**	**Pediatric**	**Pediatric**	**Pediatric**	**Pediatric**	**Pediatric**
**Cell lineage**		**T-cell**	**T-cell**	**T-cell**	**T-cell**	**B-cell**	**B-cell**
**Sub-type of leukemia**		**ALL**	**ALL**	**ALL**	**ALL**	**ALL**	**ALL**
**Name**	**Description**	**C7-14 Dx**	**C7-14 Dx**	**C1-6 Dx**	**C1-6 Dx**	**SUP Dx**	**SUP Dx**
			**Stat. sign**		**Stat. sign**		**Stat. sign**
TXNRD1	thioredoxin reductase 1	-1.3		-1.4	**-1.4**	-1.3	
UAP1	UDP-N-acteylglucosamine pyrophosphorylase 1	-1.3	**-1.3**	-1.5	**-1.5**	-2.3	**-2.3**
UBE2L6	ubiquitin-conjugating enzyme E2L 6	-1.2	**-1.2**	-1.3	**-1.3**	-1.7	**-1.7**
UBE2N	ubiquitin-conjugating enzyme E2N (UBC13 homolog, yeast)	-1.2		-1.2		-1.3	**-1.3**
UBE2S	ubiquitin-conjugating enzyme E2S	-1.6		-1.3		-2.1	**-2.1**
UBTF	upstream binding transcription factor, RNA polymerase I	-1.3	**-1.3**	-1.3		-1.3	**-1.3**
UCHL3	ubiquitin carboxyl-terminal esterase L3 (ubiquitin thiolesterase)	-1.4		-1.5		-1.7	**-1.7**
UMPS	uridine monophosphate synthetase (orotate phosphoribosyl transferase and orotidine-5'-decarboxylase)	-1.3	**-1.3**	-1.4		-1.3	
UQCRC2	ubiquinol-cytochrome c reductase core protein II	-1.2		-1.5		-1.2	
UTRN	utrophin	1.7		2.5		1.6	**1.6**
VARS	valyl-tRNA synthetase	-1.5		-1.3		-2.1	**-2.1**
VPS26	vacuolar protein sorting 26 homolog A (yeast)	1.3		1.2		1.3	**1.3**
XPOT	exportin, tRNA (nuclear export receptor for tRNAs)	-1.4		-1.5		-3.2	**-3.2**
YAF2	YY1 associated factor 2	1.8	**1.8**	1.6	**1.6**	1.9	**1.9**
YARS	tyrosyl-tRNA synthetase	-1.6		-1.3		-2.6	**-2.6**
ZFP36L2	zinc finger protein 36, C3H type-like 2	3.0	**3.0**	2.5	**2.5**	1.8	**1.8**
ZFX	zinc finger protein, X-linked	1.8		1.3		1.4	
ZHX3	zinc fingers and homeoboxes 3	1.3		1.3		2.0	**2.0**
ZNF259	zinc finger protein 259	-1.4	**-1.4**	-1.8	**-1.8**	-1.3	
ZNF263	zinc finger protein 263	-1.2		-1.6		-1.7	**-1.7**
ZNF364	zinc finger protein 364	1.2		1.2		1.8	**1.8**
ZNF9	CCHC-type zinc finger, nucleic acid binding protein	-1.5		-1.6		-1.4	**-1.4**
ZRF1	zuotin related factor 1	-1.6		-1.6		-2.3	**-1.5**
