# Supplementary material for: Gene expression profiling of leukemic cells and primary thymocytes predicts a signature for apoptotic sensitivity to glucocorticoids
Source: Cancer Cell Int. 2007 Nov 28;7:18. doi: 10.1186/1475-2867-7-18 (PMC2228275; doi:10.1186/1475-2867-7-18)
Supplement: Additional file 9 — Pediatric B-cell ALL vs. Adult B-cell ALL. Genes statistically significantly regulated by Dex in pediatric SUP-B15 cells compared to adult RS4;11 cells. Blank = gene "absent" by selection criteria. [file 1475-2867-7-18-S9.pdf]

Additional file 9: Pediatric B-cell ALL vs. Adult B-cell ALL.			
Blank = "absent"			
GC-response		Sensitive	Sensitive
Patient-derived cell line		Pediatric	Adult
Cell lineage		B-cell	B-cell
Sub-type of leukemia		ALL	ALL
Name	Description	SUP Dx	RS4 Dx
		Stat. sign	Stat. sign
76P	gamma tubulin ring complex protein (76p gene)	-1.6	-1.9
A2M	alpha-2-macroglobulin		2.7
AACS	acetoacetyl-CoA synthetase		-1.8
AADAT	aminoadipate aminotransferase		36.7
AAK1	--	1.8	-2.3
AARS	alanyl-tRNA synthetase	-1.9	-2.0
AARSL	alanyl-tRNA synthetase like	-1.3	
AASDH	2-aminoadipic 6-semialdehyde dehydrogenase		2.3
AASDHPPT	aminoadipate-semialdehyde dehydrogenase-phosphopantetheinyl transferase	-1.3	-1.4
AASS	aminoadipate-semialdehyde synthase		-5.2
AATF	apoptosis antagonizing transcription factor		-1.3
ABAT	4-aminobutyrate aminotransferase		-21.9
ABCA1	ATP-binding cassette, sub-family A (ABC1), member 1	-1.6	
ABCA2	ATP-binding cassette, sub-family A (ABC1), member 2	-1.7	-2.2
ABCA7	ATP-binding cassette, sub-family A (ABC1), member 7	1.4	
ABCB1	ATP-binding cassette, sub-family B (MDR/TAP), member 1	2.2	5.2
ABCB10	ATP-binding cassette, sub-family B (MDR/TAP), member 10	1.3	
ABCB7	ATP-binding cassette, sub-family B (MDR/TAP), member 7		1.3
ABCC1	ATP-binding cassette, sub-family C (CFTR/MRP), member 1	-2.0	1.9
ABCC4	ATP-binding cassette, sub-family C (CFTR/MRP), member 4	7.2	1.6
ABCE1	ATP-binding cassette, sub-family E (OABP), member 1	-2.0	1.4
ABCF2	ATP-binding cassette, sub-family F (GCN20), member 2	-1.9	
ABHD2	abhydrolase domain containing 2	-3.6	
ABI1	abl-interactor 1	1.7	1.4
ABI2	abl interactor 2	-1.9	1.3
ABL2	v-abl Abelson murine leukemia viral oncogene homolog 2 (arg, Abelson-related gene)		1.3
ABLIM1	actin binding LIM protein 1	-3.1	-5.8
ACAA1	acetyl-Coenzyme A acyltransferase 1 (peroxisomal 3-oxoacyl-Coenzyme A thiolase)	-1.3	
ACAA2	acetyl-Coenzyme A acyltransferase 2 (mitochondrial 3-oxoacyl-Coenzyme A thiolase)		-2.6
ACACB	acetyl-Coenzyme A carboxylase beta		9.5
ACAD11	acyl-Coenzyme A dehydrogenase family, member 11		1.3
ACAD8	acyl-Coenzyme A dehydrogenase family, member 8		1.9
ACAD9	acyl-Coenzyme A dehydrogenase family, member 9	-1.4	

ACADM	acyl-Coenzyme A dehydrogenase, C-4 to C-12 straight chain		**-1.8**
ACADSB	acyl-Coenzyme A dehydrogenase, short/branched chain	**-2.0**	
ACAT1	acetyl-Coenzyme A acetyltransferase 1 (acetoacetyl Coenzyme A thiolase)	**-1.5**	
ACAT2	acetyl-Coenzyme A acetyltransferase 2 (acetoacetyl Coenzyme A thiolase)	**-1.5**	**-2.4**
ACATE2	acyl-CoA thioesterase 9		**-1.4**
ACBD3	acyl-Coenzyme A binding domain containing 3	**1.4**	**-2.0**
ACCN2	amiloride-sensitive cation channel 2, neuronal		**2.2**
ACD	adrenocortical dysplasia homolog (mouse)		**-1.3**
ACLY	ATP citrate lyase	**-1.7**	**-1.4**
ACO1	aconitase 1, soluble		**1.4**
ACOX1	acyl-Coenzyme A oxidase 1, palmitoyl		**5.2**
ACOX3	acyl-Coenzyme A oxidase 3, pristanoyl	**-1.7**	
ACP1	acid phosphatase 1, soluble		**-2.1**
ACP6	acid phosphatase 6, lysophosphatidic	**-1.5**	**12.0**
ACRV1	acrosomal vesicle protein 1	**-1.7**	**-1.5**
ACSL1	acyl-CoA synthetase long-chain family member 1	**-1.6**	**4.6**
ACSL3	acyl-CoA synthetase long-chain family member 3	**-1.3**	**-1.8**
ACSL4	acyl-CoA synthetase long-chain family member 4		**-1.7**
ACSL5	acyl-CoA synthetase long-chain family member 5	**-1.6**	**1.3**
ACTB	actin, beta		**-1.5**
ACTL6A	actin-like 6A	**-1.3**	**-1.5**
ACTN1	actinin, alpha 1	**-1.2**	**5.4**
ACTR2	ARP2 actin-related protein 2 homolog (yeast)	**1.3**	**-1.4**
ACTR3	ARP3 actin-related protein 3 homolog (yeast)		**2.0**
ACVR1	activin A receptor, type I		**-1.8**
ACVR1B	activin A receptor, type IB		**1.7**
ACVR2	activin A receptor, type IIA		**11.3**
ACVR2B	activin A receptor, type IIB		**3.6**
ACY1	aminoacylase 1		**1.7**
AD023	MIF4G domain containing	**1.2**	**1.2**
ADA	adenosine deaminase	**1.3**	**-9.1**
ADAM10	ADAM metallopeptidase domain 10	**2.1**	**-3.0**
ADAM17	ADAM metallopeptidase domain 17 (tumor necrosis factor, alpha, converting enzyme)	**1.5**	**-1.7**
ADAM9	ADAM metallopeptidase domain 9 (meltrin gamma)	**1.6**	**2.4**
ADAMTS3	ADAM metallopeptidase with thrombospondin type 1 motif, 3		**19.4**
ADAR	adenosine deaminase, RNA-specific	**-1.2**	
ADARB1	adenosine deaminase, RNA-specific, B1 (RED1 homolog rat)		**-34.5**
ADAT1	adenosine deaminase, tRNA-specific 1		**-1.3**
ADCY7	adenylate cyclase 7	**-1.5**	**-3.1**
ADCY9	adenylate cyclase 9		**-1.7**
ADD1	adducin 1 (alpha)	**1.8**	**1.2**
ADD3	adducin 3 (gamma)	**2.7**	

ADFP	adipose differentiation-related protein	**1.5**	**3.3**
ADH4	alcohol dehydrogenase 4 (class II), pi polypeptide		**1.6**
ADH5	alcohol dehydrogenase 5 (class III), chi polypeptide		**2.4**
ADIPOR2	adiponectin receptor 2	**1.9**	**-1.5**
ADK	adenosine kinase	**-2.0**	**1.6**
ADORA2A	adenosine A2a receptor	**1.5**	
ADORA2B	adenosine A2b receptor		**3.0**
ADRA1A	adrenergic, alpha-1A-, receptor	**2.0**	**-1.8**
ADRA2C	adrenergic, alpha-2C-, receptor		**2.4**
ADRB1	adrenergic, beta-1-, receptor	**2.5**	
ADRBK1	adrenergic, beta, receptor kinase 1		**-2.0**
ADRBK2	adrenergic, beta, receptor kinase 2	**-1.5**	**1.6**
ADRM1	adhesion regulating molecule 1		**2.2**
ADSL	adenylosuccinate lyase	**-1.5**	**1.5**
ADSS	adenylosuccinate synthase	**-1.3**	**1.2**
ADSSL1	adenylosuccinate synthase like 1		**3.0**
AEBP1	AE binding protein 1	**1.5**	**-1.6**
AES	amino-terminal enhancer of split		**-2.1**
AFAP	actin filament associated protein		**16.5**
AFF1	AF4/FMR2 family, member 1	**2.8**	**-3.5**
AFG3L2	AFG3 ATPase family gene 3-like 2 (yeast)		**1.3**
AGA	aspartylglucosaminidase		**-1.3**
AGGF1	angiogenic factor with G patch and FHA domains 1		**1.8**
AGL	amylo-1, 6-glucosidase, 4-alpha-glucanotransferase (glycogen debranching enzyme, glycogen storage disease type III)		**-2.0**
AGMAT	agmatine ureohydrolase (agmatinase)		**4.7**
AGPAT1	1-acylglycerol-3-phosphate O-acyltransferase 1 (lysophosphatidic acid acyltransferase, alpha)	**-1.4**	**1.8**
AGPAT2	1-acylglycerol-3-phosphate O-acyltransferase 2 (lysophosphatidic acid acyltransferase, beta)		**1.5**
AGPAT3	1-acylglycerol-3-phosphate O-acyltransferase 3		**20.0**
AGPAT5	1-acylglycerol-3-phosphate O-acyltransferase 5 (lysophosphatidic acid acyltransferase, epsilon)	**-2.2**	**1.6**
AGPAT6	1-acylglycerol-3-phosphate O-acyltransferase 6 (lysophosphatidic acid acyltransferase, zeta)		**1.6**
AGPS	alkylglycerone phosphate synthase	**5.0**	**5.5**
AGRN	agrin	**-2.8**	**-1.6**
AGT	angiotensinogen (serpin peptidase inhibitor, clade A, member 8)		**2.1**
AGTR1	angiotensin II receptor, type 1	**1.3**	
AGTRAP	angiotensin II receptor-associated protein		**2.0**
AHCY	S-adenosylhomocysteine hydrolase	**-1.4**	**1.4**
AHCYL1	S-adenosylhomocysteine hydrolase-like 1	**-1.4**	**1.6**
AHSA1	AHA1, activator of heat shock 90kDa protein ATPase homolog 1 (yeast)	**-1.7**	
AIF1	allograft inflammatory factor 1		**2.5**
AIP	aryl hydrocarbon receptor interacting protein		**-1.4**
AK1	adenylate kinase 1	**2.1**	
AK2	adenylate kinase 2	**-2.2**	**-1.4**

AK3	adenylate kinase 3-like 1	**-2.9**	**7.9**
AKAP1	A kinase (PRKA) anchor protein 1	**-2.4**	**-1.8**
AKAP10	A kinase (PRKA) anchor protein 10		**-2.7**
AKAP11	A kinase (PRKA) anchor protein 11	**-1.3**	**-1.2**
AKAP12	A kinase (PRKA) anchor protein (gravin) 12	**-1.9**	
AKAP13	A kinase (PRKA) anchor protein 13	**4.1**	**-2.9**
AKAP2	A kinase (PRKA) anchor protein 2	**4.0**	
AKAP7	A kinase (PRKA) anchor protein 7		**-3.3**
AKAP8L	A kinase (PRKA) anchor protein 8-like	**2.5**	**-1.3**
AKNA	AT-hook transcription factor	**2.3**	
AKR1A1	aldo-keto reductase family 1, member A1 (aldehyde reductase)	**-2.3**	**-2.6**
AKR1B1	aldo-keto reductase family 1, member B1 (aldose reductase)	**-1.2**	**-4.2**
AKR1C3	aldo-keto reductase family 1, member C3 (3-alpha hydroxysteroid dehydrogenase, type II)	**3.7**	
AKR7A2	aldo-keto reductase family 7, member A2 (aflatoxin aldehyde reductase)		**-1.6**
AKT1	v-akt murine thymoma viral oncogene homolog 1		**-1.5**
AKT1S1	AKT1 substrate 1 (proline-rich)		**1.6**
AKT3	v-akt murine thymoma viral oncogene homolog 3 (protein kinase B, gamma)	**1.4**	**-6.6**
ALAS1	aminolevulinate, delta-, synthase 1		**2.4**
ALCAM	activated leukocyte cell adhesion molecule	**4.0**	**1.4**
ALDH18A1	aldehyde dehydrogenase 18 family, member A1	**-2.1**	**1.4**
ALDH4A1	aldehyde dehydrogenase 4 family, member A1		**1.9**
ALDH5A1	aldehyde dehydrogenase 5 family, member A1 (succinate-semialdehyde dehydrogenase)	**-1.4**	**-1.5**
ALDH6A1	aldehyde dehydrogenase 6 family, member A1	**-1.7**	**1.6**
ALDH9A1	aldehyde dehydrogenase 9 family, member A1	**-1.6**	
ALDOA	aldolase A, fructose-bisphosphate	**-1.3**	**-1.5**
ALG2	asparagine-linked glycosylation 2 homolog (S. cerevisiae, alpha-1,3-mannosyltransferase)		**2.0**
ALG5	asparagine-linked glycosylation 5 homolog (S. cerevisiae, dolichyl-phosphate beta-glucosyltransferase)	**-1.5**	**-1.7**
ALS2	amyotrophic lateral sclerosis 2 (juvenile)		**14.0**
ALS2CR2	amyotrophic lateral sclerosis 2 (juvenile) chromosome region, candidate 2	**1.6**	**1.8**
ALS2CR3	trafficking protein, kinesin binding 2	**2.4**	**1.7**
AMACR	alpha-methylacyl-CoA racemase		**2.0**
AMD1	adenosylmethionine decarboxylase 1		**1.4**
AMPD2	adenosine monophosphate deaminase 2 (isoform L)	**-2.5**	**1.9**
AMPH	amphiphysin (Stiff-Man syndrome with breast cancer 128kDa autoantigen)		**33.8**
ANAPC1	anaphase promoting complex subunit 1	**-1.3**	**1.6**
ANAPC10	anaphase promoting complex subunit 10		**1.8**
ANAPC11	APC11 anaphase promoting complex subunit 11 homolog (yeast)	**1.4**	
ANAPC13	anaphase promoting complex subunit 13	**-1.3**	**1.2**
ANAPC4	anaphase promoting complex subunit 4		**1.3**
ANAPC5	anaphase promoting complex subunit 5	**-1.8**	**1.5**
ANAPC7	anaphase promoting complex subunit 7		**1.4**
ANGPT1	angiopoietin 1		**1.8**

ANK1	ankyrin 1, erythrocytic	**1.4**	
ANKH	ankylosis, progressive homolog (mouse)	**-2.0**	**-3.7**
ANKHD1	ankyrin repeat and KH domain containing 1	**2.1**	**1.3**
ANKRA2	ankyrin repeat, family A (RFXANK-like), 2	**1.5**	
ANKRD10	ankyrin repeat domain 10	**-1.7**	**1.3**
ANKRD25	ankyrin repeat domain 25	**-1.5**	**1.6**
ANKRD28	ankyrin repeat domain 28	**2.2**	**2.2**
ANKRD32	ankyrin repeat domain 32		**-1.7**
ANKS1	ankyrin repeat and sterile alpha motif domain containing 1A	**3.3**	
ANLN	anillin, actin binding protein	**1.8**	**2.2**
ANP32A	acidic (leucine-rich) nuclear phosphoprotein 32 family, member A	**-1.3**	**2.1**
ANP32B	acidic (leucine-rich) nuclear phosphoprotein 32 family, member B	**-1.3**	
ANPEP	alanyl (membrane) aminopeptidase (aminopeptidase N, aminopeptidase M, microsomal aminopeptidase, CD13, p150)		**2.8**
ANTXR1	anthrax toxin receptor 1		**24.1**
ANXA11	annexin A11	**1.8**	**1.8**
ANXA2	annexin A2	**1.4**	
ANXA4	annexin A4	**1.3**	
ANXA5	annexin A5	**2.0**	**-2.0**
ANXA6	annexin A6		**2.4**
ANXA7	annexin A7		**1.6**
AOF2	amine oxidase (flavin containing) domain 2	**1.5**	
AOX1	aldehyde oxidase 1		**1.6**
AP1B1	adaptor-related protein complex 1, beta 1 subunit		**-1.4**
AP1G1	adaptor-related protein complex 1, gamma 1 subunit	**-1.2**	
AP1GBP1	AP1 gamma subunit binding protein 1	**2.5**	**-1.6**
AP1S2	adaptor-related protein complex 1, sigma 2 subunit	**1.6**	**-27.2**
AP2A2	adaptor-related protein complex 2, alpha 2 subunit	**1.4**	**1.5**
AP2B1	adaptor-related protein complex 2, beta 1 subunit	**1.6**	
AP3D1	adaptor-related protein complex 3, delta 1 subunit	**1.3**	**1.5**
AP3M1	adaptor-related protein complex 3, mu 1 subunit		**1.2**
AP3M2	adaptor-related protein complex 3, mu 2 subunit	**-1.4**	
AP3S1	adaptor-related protein complex 3, sigma 1 subunit	**2.4**	**1.4**
AP3S2	adaptor-related protein complex 3, sigma 2 subunit		**2.2**
AP4S1	adaptor-related protein complex 4, sigma 1 subunit	**2.7**	
APBA2BP	amyloid beta (A4) precursor protein-binding, family A, member 2 binding protein	**2.2**	
APBA3	amyloid beta (A4) precursor protein-binding, family A, member 3 (X11-like 2)		**1.8**
APBB3	amyloid beta (A4) precursor protein-binding, family B, member 3	**-1.4**	
APC	adenomatosis polyposis coli	**1.4**	**1.2**
APEX1	APEX nuclease (multifunctional DNA repair enzyme) 1	**-2.2**	
APEX2	APEX nuclease (apurinic/apyrimidinic endonuclease) 2		**-1.4**
APG12L	ATG12 autophagy related 12 homolog (S. cerevisiae)	**1.9**	**1.3**
APG4B	ATG4 autophagy related 4 homolog B (S. cerevisiae)	**1.3**	**-1.8**

APG5L	ATG5 autophagy related 5 homolog (S. cerevisiae)		**-1.3**
API5	apoptosis inhibitor 5	**-2.1**	**1.6**
APLP2	amyloid beta (A4) precursor-like protein 2	**-1.7**	**4.2**
APOBEC3B	apolipoprotein B mRNA editing enzyme, catalytic polypeptide-like 3B		**-1.9**
APOBEC3G	apolipoprotein B mRNA editing enzyme, catalytic polypeptide-like 3G		**-2.6**
APOC4	apolipoprotein C-IV	**-1.8**	
APP	amyloid beta (A4) precursor protein (peptidase nexin-II, Alzheimer disease)	**-1.8**	**449.2**
APPBP1	amyloid beta precursor protein binding protein 1	**-1.3**	**-1.2**
APPBP2	amyloid beta precursor protein (cytoplasmic tail) binding protein 2	**1.4**	**2.0**
APPL	adaptor protein containing pH domain, PTB domain and leucine zipper motif 1	**1.3**	
APRT	adenine phosphoribosyltransferase	**-1.7**	
APS	SH2B adaptor protein 2	**-1.5**	**-1.6**
AR	androgen receptor (dihydrotestosterone receptor; testicular feminization; spinal and bulbar muscular atrophy; Kennedy disease)		**22.5**
ARF1	ADP-ribosylation factor 1	**-1.2**	**1.3**
ARF3	ADP-ribosylation factor 3	**-1.3**	**1.4**
ARF4	ADP-ribosylation factor 4		**1.3**
ARF5	ADP-ribosylation factor 5		**-2.4**
ARF6	ADP-ribosylation factor 6	**1.7**	**1.4**
ARFGAP1	ADP-ribosylation factor GTPase activating protein 1		**1.5**
ARFGAP3	ADP-ribosylation factor GTPase activating protein 3		**1.4**
ARFGEF1	ADP-ribosylation factor guanine nucleotide-exchange factor 1(brefeldin A-inhibited)	**1.5**	**-1.6**
ARFGEF2	ADP-ribosylation factor guanine nucleotide-exchange factor 2 (brefeldin A-inhibited)		**1.3**
ARHGAP11A	Rho GTPase activating protein 11A	**1.6**	
ARHGAP18	Rho GTPase activating protein 18	**4.5**	**1.6**
ARHGAP21	Rho GTPase activating protein 21	**1.3**	
ARHGAP22	Rho GTPase activating protein 22	**-18.6**	
ARHGAP26	Rho GTPase activating protein 26	**-2.0**	**1.9**
ARHGAP5	Rho GTPase activating protein 5		**-1.4**
ARHGDIA	Rho GDP dissociation inhibitor (GDI) alpha		**-1.3**
ARHGDIB	Rho GDP dissociation inhibitor (GDI) beta		**-1.4**
ARHGEF12	Rho guanine nucleotide exchange factor (GEF) 12		**-1.5**
ARHGEF2	rho/rac guanine nucleotide exchange factor (GEF) 2	**1.3**	**1.3**
ARHGEF3	Rho guanine nucleotide exchange factor (GEF) 3	**-1.5**	
ARHGEF6	Rac/Cdc42 guanine nucleotide exchange factor (GEF) 6	**-1.3**	**-1.4**
ARHGEF7	Rho guanine nucleotide exchange factor (GEF) 7	**3.6**	**-1.8**
ARHGEF9	Cdc42 guanine nucleotide exchange factor (GEF) 9		**1.6**
ARID1A	AT rich interactive domain 1A (SWI-like)	**1.5**	**-1.3**
ARID1B	AT rich interactive domain 1B (SWI1-like)	**-2.0**	**-1.7**
ARID2	AT rich interactive domain 2 (ARID, RFX-like)	**1.3**	**1.6**
ARID4A	AT rich interactive domain 4A (RBP1-like)		**2.9**
ARID5B	AT rich interactive domain 5B (MRF1-like)	**-2.2**	**-34.1**
ARIH1	ariadne homolog, ubiquitin-conjugating enzyme E2 binding protein, 1 (Drosophila)	**1.6**	**1.7**

ARIH2	ariadne homolog 2 (Drosophila)	**1.6**	**-1.5**
ARL1	ADP-ribosylation factor-like 1		**1.3**
ARL3	ADP-ribosylation factor-like 3		**-1.6**
ARL4A	ADP-ribosylation factor-like 4A		**26.8**
ARL6IP	ADP-ribosylation factor-like 6 interacting protein 1	**1.7**	**-2.2**
ARL6IP2	ADP-ribosylation factor-like 6 interacting protein 2	**-1.3**	**1.8**
ARL6IP4	ADP-ribosylation-like factor 6 interacting protein 4		**-1.3**
ARL6IP5	ADP-ribosylation-like factor 6 interacting protein 5	**1.4**	
ARL6IP6	ADP-ribosylation-like factor 6 interacting protein 6	**1.5**	
ARL7	ADP-ribosylation factor-like 4C	**2.8**	
ARMET	arginine-rich, mutated in early stage tumors	**1.4**	
ARPC1A	actin related protein 2/3 complex, subunit 1A, 41kDa		**-2.8**
ARPC1B	actin related protein 2/3 complex, subunit 1B, 41kDa		**1.3**
ARPC2	actin related protein 2/3 complex, subunit 2, 34kDa		**-1.3**
ARPC3	actin related protein 2/3 complex, subunit 3, 21kDa	**1.4**	
ARPC4	actin related protein 2/3 complex, subunit 4, 20kDa		**-4.7**
ARPC5	actin related protein 2/3 complex, subunit 5, 16kDa	**1.4**	**-1.4**
ARRB1	arrestin, beta 1	**2.2**	**2.3**
ARRB2	arrestin, beta 2		**-2.1**
ARTS-1	type 1 tumor necrosis factor receptor shedding aminopeptidase regulator	**-1.3**	**-3.5**
ASAH1	N-acylsphingosine amidohydrolase (acid ceramidase) 1	**-1.3**	**4.4**
ASB13	ankyrin repeat and SOCS box-containing 13	**3.0**	**1.7**
ASCC3L1	activating signal cointegrator 1 complex subunit 3-like 1	**-1.4**	**-1.5**
ASE-1	CD3e molecule, epsilon associated protein	**-1.9**	
ASF1B	ASF1 anti-silencing function 1 homolog B (S. cerevisiae)	**-1.4**	**-1.4**
ASK	DBF4 homolog (S. cerevisiae)	**1.5**	**-1.7**
ASMTL	acetylserotonin O-methyltransferase-like		**-2.7**
ASNS	asparagine synthetase		**2.9**
ASNSD1	asparagine synthetase domain containing 1	**-1.2**	**1.8**
ASPH	aspartate beta-hydroxylase		**1.5**
ASPM	asp (abnormal spindle) homolog, microcephaly associated (Drosophila)	**2.0**	**-1.7**
ASS	argininosuccinate synthetase 1	**-3.8**	**-1.4**
ASXL1	additional sex combs like 1 (Drosophila)	**-1.4**	**-1.6**
ATAD2	ATPase family, AAA domain containing 2	**1.7**	**1.4**
ATAD3A	ATPase family, AAA domain containing 3A	**-1.9**	
ATBF1	AT-binding transcription factor 1	**-1.5**	**-4.4**
ATE1	arginyltransferase 1	**-1.2**	**1.4**
ATF1	activating transcription factor 1		**-1.4**
ATF2	activating transcription factor 2	**1.5**	**1.6**
ATF3	activating transcription factor 3	**-1.5**	
ATF4	activating transcription factor 4 (tax-responsive enhancer element B67)	**-1.4**	**-1.3**
ATF5	activating transcription factor 5	**-2.0**	**-1.6**

ATF6	activating transcription factor 6		**5.8**
ATF7IP	activating transcription factor 7 interacting protein	**1.7**	**-1.4**
ATF7IP2	activating transcription factor 7 interacting protein 2		**-1.2**
ATIC	5-aminoimidazole-4-carboxamide ribonucleotide formyltransferase/IMP cyclohydrolase	**-3.2**	**-2.1**
ATM	ataxia telangiectasia mutated (includes complementation groups A, C and D)	**1.2**	
ATN1	atrophin 1	**-1.3**	**11.3**
ATOX1	ATX1 antioxidant protein 1 homolog (yeast)		**5.6**
ATP11B	ATPase, Class VI, type 11B	**5.1**	**-1.6**
ATP1A1	ATPase, Na+/K+ transporting, alpha 1 polypeptide	**-1.6**	**1.3**
ATP1A3	ATPase, Na+/K+ transporting, alpha 3 polypeptide		**-13.1**
ATP1B1	ATPase, Na+/K+ transporting, beta 1 polypeptide	**-2.5**	**-1.4**
ATP2A2	ATPase, Ca++ transporting, cardiac muscle, slow twitch 2	**-1.5**	**1.3**
ATP2A3	ATPase, Ca++ transporting, ubiquitous	**1.2**	**2.5**
ATP2B1	ATPase, Ca++ transporting, plasma membrane 1	**-2.1**	**-2.1**
ATP2B4	ATPase, Ca++ transporting, plasma membrane 4	**2.8**	**-3.8**
ATP2C1	ATPase, Ca++ transporting, type 2C, member 1		**1.9**
ATP4B	ATPase, H+/K+ exchanging, beta polypeptide	**7.9**	
ATP5A1	ATP synthase, H+ transporting, mitochondrial F1 complex, alpha subunit 1, cardiac muscle		**1.3**
ATP5B	ATP synthase, H+ transporting, mitochondrial F1 complex, beta polypeptide	**-1.3**	
ATP5C1	ATP synthase, H+ transporting, mitochondrial F1 complex, gamma polypeptide 1		**1.4**
ATP5E	ATP synthase, H+ transporting, mitochondrial F1 complex, epsilon subunit	**1.4**	
ATP5G1	ATP synthase, H+ transporting, mitochondrial F0 complex, subunit C1 (subunit 9)	**-2.4**	
ATP5G3	ATP synthase, H+ transporting, mitochondrial F0 complex, subunit C3 (subunit 9)		**2.3**
ATP5H	ATP synthase, H+ transporting, mitochondrial F0 complex, subunit d	**-1.2**	**1.5**
ATP5I	ATP synthase, H+ transporting, mitochondrial F0 complex, subunit E	**1.3**	
ATP5J	ATP synthase, H+ transporting, mitochondrial F0 complex, subunit F6		**-1.8**
ATP5J2	ATP synthase, H+ transporting, mitochondrial F0 complex, subunit F2		**-1.3**
ATP5L	ATP synthase, H+ transporting, mitochondrial F0 complex, subunit G	**1.6**	**1.7**
ATP5O	ATP synthase, H+ transporting, mitochondrial F1 complex, O subunit (oligomycin sensitivity conferring protein)	**-1.2**	
ATP6V0C	ATPase, H+ transporting, lysosomal 16kDa, V0 subunit c	**1.3**	**-1.4**
ATP6V0D1	ATPase, H+ transporting, lysosomal 38kDa, V0 subunit d1		**1.4**
ATP6V0E	ATPase, H+ transporting, lysosomal 9kDa, V0 subunit e1		**1.3**
ATP6V1A	ATPase, H+ transporting, lysosomal 70kDa, V1 subunit A		**1.9**
ATP6V1B2	ATPase, H+ transporting, lysosomal 56/58kDa, V1 subunit B2		**1.5**
ATP6V1C1	ATPase, H+ transporting, lysosomal 42kDa, V1 subunit C1		**-2.1**
ATP6V1D	ATPase, H+ transporting, lysosomal 34kDa, V1 subunit D	**1.3**	**-1.3**
ATP6V1E2	ATPase, H+ transporting, lysosomal 31kDa, V1 subunit E2		**-1.5**
ATP6V1F	ATPase, H+ transporting, lysosomal 14kDa, V1 subunit F	**-1.3**	
ATP7B	ATPase, Cu++ transporting, beta polypeptide		**2.4**
ATPIF1	ATPase inhibitory factor 1	**1.4**	**2.2**
ATRX	alpha thalassemia/mental retardation syndrome X-linked (RAD54 homolog, S. cerevisiae)		**1.4**
ATXN1	ataxin 1	**3.4**	**1.7**

ATXN2L	ataxin 2-like		**-2.2**
ATXN3	ataxin 3	**1.3**	
AUP1	ancient ubiquitous protein 1		**1.5**
AURKAIP1	aurora kinase A interacting protein 1	**-1.2**	**-1.4**
AURKB	aurora kinase B	**1.4**	**-3.3**
AVEN	apoptosis, caspase activation inhibitor		**-2.4**
AVO3	rapamycin-insensitive companion of mTOR		**1.3**
AXUD1	AXIN1 up-regulated 1	**1.4**	
AZI2	5-azacytidine induced 2	**1.5**	**4.3**
AZU1	azurocidin 1 (cationic antimicrobial protein 37)		**162.2**
B2M	beta-2-microglobulin		**-1.6**
B3GALNT1	beta-1,3-N-acetylgalactosaminyltransferase 1 (globoside blood group)		**4.8**
B3GALT4	UDP-Gal:betaGlcNAc beta 1,3-galactosyltransferase, polypeptide 4	**3.3**	
B3GALT6	UDP-Gal:betaGal beta 1,3-galactosyltransferase polypeptide 6		**1.4**
B4GALT1	UDP-Gal:betaGlcNAc beta 1,4- galactosyltransferase, polypeptide 1	**1.3**	
B4GALT2	UDP-Gal:betaGlcNAc beta 1,4- galactosyltransferase, polypeptide 2	**-2.1**	
B4GALT5	UDP-Gal:betaGlcNAc beta 1,4- galactosyltransferase, polypeptide 5	**-1.3**	**1.5**
B4GALT6	UDP-Gal:betaGlcNAc beta 1,4- galactosyltransferase, polypeptide 6		**2.5**
B930013M22RIK	ras responsive element binding protein 1	**2.1**	**3.1**
BACE1	beta-site APP-cleaving enzyme 1	**-1.3**	**3.6**
BACE2	beta-site APP-cleaving enzyme 2		**3.7**
BACH1	BTB and CNC homology 1, basic leucine zipper transcription factor 1	**1.3**	**1.2**
BACH2	BTB and CNC homology 1, basic leucine zipper transcription factor 2	**-1.8**	**-13.3**
BAG1	BCL2-associated athanogene	**-1.4**	
BAG2	BCL2-associated athanogene 2	**-1.8**	**-1.2**
BAG3	BCL2-associated athanogene 3		**7.3**
BAG4	BCL2-associated athanogene 4		**2.7**
BAIAP1	membrane associated guanylate kinase, WW and PDZ domain containing 1		**-3.3**
BAIAP2	BAI1-associated protein 2		**2.5**
BAMBI	BMP and activin membrane-bound inhibitor homolog (Xenopus laevis)		**-2.7**
BANF1	barrier to autointegration factor 1	**-1.5**	**-2.0**
BANK1	--	**-2.6**	
BAP1	BRCA1 associated protein-1 (ubiquitin carboxy-terminal hydrolase)	**-1.7**	
BARD1	BRCA1 associated RING domain 1	**1.3**	**-3.6**
BASP1	brain abundant, membrane attached signal protein 1		**1.6**
BAT1	HLA-B associated transcript 1	**-1.6**	**1.5**
BAT3	HLA-B associated transcript 3	**-1.4**	**-1.4**
BAT4	HLA-B associated transcript 4		**-2.0**
BAT8	euchromatic histone-lysine N-methyltransferase 2	**-4.0**	
BAX	BCL2-associated X protein		**-1.8**
BAZ1B	bromodomain adjacent to zinc finger domain, 1B	**-1.2**	**-1.9**
BBX	bobby sox homolog (Drosophila)	**-1.3**	**1.6**

BC-2	chromatin modifying protein 2A	**1.5**	**-1.4**
BCAT1	branched chain aminotransferase 1, cytosolic	**-3.3**	**-4.9**
BCAT2	branched chain aminotransferase 2, mitochondrial		**1.7**
BCCIP	BRCA2 and CDKN1A interacting protein	**1.4**	**2.3**
BCKDHB	branched chain keto acid dehydrogenase E1, beta polypeptide (maple syrup urine disease)		**2.2**
BCL10	B-cell CLL/lymphoma 10		**1.2**
BCL11A	B-cell CLL/lymphoma 11A (zinc finger protein)		**-1.8**
BCL2	B-cell CLL/lymphoma 2	**-2.8**	**-11.2**
BCL2A1	BCL2-related protein A1		**1.3**
BCL2L1	BCL2-like 1	**1.7**	
BCL2L11	BCL2-like 11 (apoptosis facilitator)	**3.1**	**1.2**
BCL2L13	BCL2-like 13 (apoptosis facilitator)		**1.3**
BCL6	B-cell CLL/lymphoma 6 (zinc finger protein 51)		**-11.8**
BCL7A	B-cell CLL/lymphoma 7A	**-1.3**	**-3.3**
BCL7B	B-cell CLL/lymphoma 7B		**-1.4**
BCL9L	B-cell CLL/lymphoma 9-like	**-1.5**	**-1.6**
BCLAF1	BCL2-associated transcription factor 1	**-1.7**	**2.0**
BCOR	BCL6 co-repressor		**-1.5**
BCR	breakpoint cluster region		**-1.4**
BDH1	3-hydroxybutyrate dehydrogenase, type 1		**-1.3**
BET1	BET1 homolog (S. cerevisiae)		**-2.2**
BET1L	blocked early in transport 1 homolog (S. cerevisiae)-like		**1.8**
BFAR	bifunctional apoptosis regulator	**-1.2**	
BICD1	bicaudal D homolog 1 (Drosophila)	**-1.4**	**-2.6**
BICD2	bicaudal D homolog 2 (Drosophila)		**-1.2**
BID	BH3 interacting domain death agonist	**-1.9**	**-3.4**
BIK	BCL2-interacting killer (apoptosis-inducing)	**6.3**	
BIN1	bridging integrator 1		**-1.3**
BIRC1	NLR family, apoptosis inhibitory protein		**-2.0**
BIRC2	baculoviral IAP repeat-containing 2	**1.9**	**1.4**
BIRC4	baculoviral IAP repeat-containing 4	**1.4**	**-1.3**
BIRC4BP	XIAP associated factor-1	**-2.4**	**-7.1**
BIRC5	baculoviral IAP repeat-containing 5 (survivin)		**-1.8**
BIRC6	--		**1.3**
BLCAP	bladder cancer associated protein	**-1.5**	**1.4**
BLK	B lymphoid tyrosine kinase	**-1.6**	**-20.5**
BLM	Bloom syndrome	**-1.8**	
BLMH	bleomycin hydrolase		**1.3**
BLNK	B-cell linker	**-2.8**	**-54.4**
BLOC1S1	biogenesis of lysosome-related organelles complex-1, subunit 1		**-1.9**
BLOC1S2	biogenesis of lysosome-related organelles complex-1, subunit 2	**-1.2**	**2.1**
BLOC1S3	biogenesis of lysosome-related organelles complex-1, subunit 3	**1.3**	**1.3**

BLVRA	biliverdin reductase A	**-1.8**	**1.8**
BMP2	bone morphogenetic protein 2	**8.9**	
BMP2K	BMP2 inducible kinase		**1.5**
BMPR2	--	**1.9**	**17.4**
BNIP2	BCL2/adenovirus E1B 19kDa interacting protein 2	**1.7**	**-1.4**
BNIP3	BCL2/adenovirus E1B 19kDa interacting protein 3		**312.5**
BNIP3L	BCL2/adenovirus E1B 19kDa interacting protein 3-like	**-1.3**	**2.0**
BOMB	WW and C2 domain containing 2		**11.9**
BOP1	block of proliferation 1	**-2.6**	
BPGM	2,3-bisphosphoglycerate mutase		**-1.5**
BPNT1	3'(2'), 5'-bisphosphate nucleotidase 1		**-2.2**
BPY2IP1	microtubule-associated protein 1S		**-1.2**
BRAF	v-raf murine sarcoma viral oncogene homolog B1	**-2.1**	**-1.4**
BRAP	BRCA1 associated protein	**1.3**	
BRCA1	breast cancer 1, early onset	**-1.4**	**-1.5**
BRCA2	breast cancer 2, early onset		**-2.7**
BRD4	bromodomain containing 4		**-1.3**
BRD7	bromodomain containing 7		**-1.2**
BRD8	bromodomain containing 8	**1.4**	
BRDG1	BCR downstream signaling 1	**-1.5**	**2.5**
BRE	brain and reproductive organ-expressed (TNFRSF1A modulator)	**-2.1**	
BRF2	BRF2, subunit of RNA polymerase III transcription initiation factor, BRF1-like	**-1.3**	**1.3**
BRMS1	breast cancer metastasis suppressor 1		**-1.6**
BRRN1	non-SMC condensin I complex, subunit H		**-1.9**
BSG	basigin (Ok blood group)	**-1.4**	
BST2	bone marrow stromal cell antigen 2		**-1.5**
BTAF1	BTAF1 RNA polymerase II, B-TFIID transcription factor-associated, 170kDa (Mot1 homolog, S. cerevisiae)		**1.6**
BTBD15	zinc finger and BTB domain containing 44	**1.8**	**1.4**
BTBD2	BTB (POZ) domain containing 2	**-1.8**	
BTG1	B-cell translocation gene 1, anti-proliferative	**3.9**	
BTG2	BTG family, member 2	**3.9**	
BTG3	BTG family, member 3		**-1.6**
BTK	Bruton agammaglobulinemia tyrosine kinase		**1.2**
BTN3A2	butyrophilin, subfamily 3, member A2		**-5.8**
BTN3A3	butyrophilin, subfamily 3, member A3	**-1.4**	**-5.9**
BTRC	beta-transducin repeat containing		**1.7**
BUB1	BUB1 budding uninhibited by benzimidazoles 1 homolog (yeast)	**1.4**	**-1.3**
BUB1B	BUB1 budding uninhibited by benzimidazoles 1 homolog beta (yeast)		**-1.9**
BUB3	BUB3 budding uninhibited by benzimidazoles 3 homolog (yeast)	**1.3**	**-1.4**
BYSL	bystin-like	**-8.4**	**-6.2**
BZRAP1	benzodiazapine receptor (peripheral) associated protein 1	**-3.6**	**6.0**
BZRP	translocator protein (18kDa)	**-1.8**	

BZW2	basic leucine zipper and W2 domains 2	**-2.5**	**1.4**
C10ORF10	chromosome 10 open reading frame 10	**3.7**	
C10ORF119	chromosome 10 open reading frame 119	**-1.5**	
C10ORF3	centrosomal protein 55kDa	**1.6**	**-2.6**
C10ORF4	chromosome 10 open reading frame 4		**2.0**
C10ORF58	chromosome 10 open reading frame 58	**-3.6**	**2.2**
C11ORF17	chromosome 11 open reading frame 17	**2.1**	**-2.0**
C13ORF12	proteasome maturation protein	**1.6**	**-4.1**
C14ORF126	chromosome 14 open reading frame 126	**-1.3**	
C14ORF130	chromosome 14 open reading frame 130	**-1.3**	**-1.5**
C14ORF46	lin-52 homolog (C. elegans)	**-1.3**	**-1.3**
C15ORF23	chromosome 15 open reading frame 23		**-1.8**
C18ORF37	chromosome 18 open reading frame 37	**1.7**	**1.7**
C19ORF10	chromosome 19 open reading frame 10		**1.3**
C19ORF2	chromosome 19 open reading frame 2	**-1.3**	**1.8**
C19ORF29	chromosome 19 open reading frame 29	**-1.6**	
C1D	nuclear DNA-binding protein		**-1.6**
C1ORF108	chromosome 1 open reading frame 108	**-1.3**	**1.3**
C1ORF19	chromosome 1 open reading frame 19	**-1.7**	**-2.5**
C1ORF24	family with sequence similarity 129, member A		**90.2**
C1ORF33	mRNA turnover 4 homolog (S. cerevisiae)	**-4.1**	**-1.2**
C1ORF48	NSL1, MIND kinetochore complex component, homolog (S. cerevisiae)		**-1.5**
C1QBP	complement component 1, q subcomponent binding protein	**-1.9**	**-2.0**
C1QR1	CD93 molecule	**2.0**	**3.3**
C1RL	complement component 1, r subcomponent-like		**2.3**
C20ORF24	chromosome 20 open reading frame 24		**1.7**
C20ORF42	chromosome 20 open reading frame 42		**18.6**
C20ORF72	chromosome 20 open reading frame 72	**1.4**	**1.2**
C21ORF33	chromosome 21 open reading frame 33		**1.8**
C21ORF6	chromosome 21 open reading frame 6		**2.3**
C21ORF7	chromosome 21 open reading frame 7	**1.6**	
C22ORF9	chromosome 22 open reading frame 9	**-1.4**	**-2.1**
C3F	membrane bound O-acyltransferase domain containing 5	**-1.5**	
C3ORF10	chromosome 3 open reading frame 10	**1.3**	
C5	complement component 5		**2.6**
C5ORF13	chromosome 5 open reading frame 13	**-3.3**	**-3.7**
C5ORF18	receptor accessory protein 5	**1.4**	**3.6**
C6ORF108	chromosome 6 open reading frame 108	**-3.3**	**2.7**
C6ORF170	chromosome 6 open reading frame 170		**1.2**
C6ORF69	potassium channel tetramerisation domain containing 20		**-1.3**
C6ORF79	coiled-coil domain containing 90A	**-1.6**	**-1.5**
C9ORF76	RMI1, RecQ mediated genome instability 1, homolog (S. cerevisiae)	**-1.3**	**-1.8**

C9ORF80	chromosome 9 open reading frame 80	**-1.4**	
C9ORF86	chromosome 9 open reading frame 86	**-1.7**	
CA2	carbonic anhydrase II		**165.9**
CAB39	calcium binding protein 39	**1.4**	**-1.8**
CABIN1	calcineurin binding protein 1	**-1.4**	**-1.3**
CABLES1	Cdk5 and Abl enzyme substrate 1	**-2.2**	
CABLES2	Cdk5 and Abl enzyme substrate 2		**-1.5**
CACNA1D	calcium channel, voltage-dependent, L type, alpha 1D subunit	**-2.8**	
CACNA2D4	--		**-12.4**
CACNB3	calcium channel, voltage-dependent, beta 3 subunit	**1.4**	
CACNB4	calcium channel, voltage-dependent, beta 4 subunit		**2.3**
CACYBP	calcyclin binding protein	**-1.5**	**-1.5**
CAD	carbamoyl-phosphate synthetase 2, aspartate transcarbamylase, and dihydroorotase	**-2.8**	
CALCRL	calcitonin receptor-like	**86.7**	
CALD1	caldesmon 1	**-1.3**	
CALM1	calmodulin 1 (phosphorylase kinase, delta)	**1.6**	**-1.4**
CALM2	calmodulin 2 (phosphorylase kinase, delta)	**1.2**	**-1.2**
CALM3	calmodulin 3 (phosphorylase kinase, delta)	**1.2**	**-1.5**
CALR	calreticulin	**-1.4**	**4.7**
CALU	calumenin	**-2.6**	
CAMK1D	calcium/calmodulin-dependent protein kinase ID	**1.9**	**-14.2**
CAMK2D	calcium/calmodulin-dependent protein kinase (CaM kinase) II delta	**2.4**	**-19.7**
CAMK2G	calcium/calmodulin-dependent protein kinase (CaM kinase) II gamma		**2.1**
CAMK4	calcium/calmodulin-dependent protein kinase IV		**-5.8**
CAMKK2	calcium/calmodulin-dependent protein kinase kinase 2, beta	**-2.1**	**1.7**
CAMLG	calcium modulating ligand	**-1.5**	**2.9**
CANT1	calcium activated nucleotidase 1	**1.4**	**2.0**
CANX	calnexin	**-1.3**	**1.6**
CAP1	CAP, adenylate cyclase-associated protein 1 (yeast)	**1.3**	**1.3**
CAP2	CAP, adenylate cyclase-associated protein, 2 (yeast)	**1.3**	
CAP350	centrosomal protein 350kDa	**2.3**	**1.6**
CAPG	capping protein (actin filament), gelsolin-like		**-49.5**
CAPN2	calpain 2, (m/II) large subunit		**2.4**
CAPN3	calpain 3, (p94)	**3.2**	**-2.0**
CAPN7	calpain 7		**1.4**
CAPNS1	calpain, small subunit 1		**-1.3**
CAPZA1	--		**1.3**
CAPZA2	capping protein (actin filament) muscle Z-line, alpha 2	**1.8**	**-2.1**
CARD6	caspase recruitment domain family, member 6		**4.0**
CARD8	caspase recruitment domain family, member 8		**1.3**
CARD9	caspase recruitment domain family, member 9		**-2.3**
CARHSP1	calcium regulated heat stable protein 1, 24kDa	**1.2**	**-5.4**

CARM1	coactivator-associated arginine methyltransferase 1	**-1.7**	**-1.8**
CARS	cysteinyl-tRNA synthetase	**-1.2**	**-1.7**
CASC5	cancer susceptibility candidate 5	**1.5**	**-3.3**
CASK	calcium/calmodulin-dependent serine protein kinase (MAGUK family)		**-1.3**
CASP1	caspase 1, apoptosis-related cysteine peptidase (interleukin 1, beta, convertase)	**-1.6**	**-2.5**
CASP2	caspase 2, apoptosis-related cysteine peptidase (neural precursor cell expressed, developmentally down-regulated 2)	**-1.8**	**-3.1**
CASP4	caspase 4, apoptosis-related cysteine peptidase	**2.0**	
CASP6	caspase 6, apoptosis-related cysteine peptidase	**1.4**	**2.4**
CASP7	caspase 7, apoptosis-related cysteine peptidase	**1.3**	
CASP8	caspase 8, apoptosis-related cysteine peptidase	**1.5**	**3.2**
CASP8AP2	CASP8 associated protein 2	**-1.5**	**-2.0**
CASP9	caspase 9, apoptosis-related cysteine peptidase		**-1.3**
CASQ1	calsequestrin 1 (fast-twitch, skeletal muscle)		**2.7**
CASR	calcium-sensing receptor (hypocalciuric hypercalcemia 1, severe neonatal hyperparathyroidism)		**-1.2**
CAST	calpastatin	**1.5**	**2.5**
CAT	catalase	**-1.3**	**4.5**
CAV1	caveolin 1, caveolae protein, 22kDa	**1.3**	
CBFA2T2	core-binding factor, runt domain, alpha subunit 2; translocated to, 2		**1.5**
CBFA2T3	core-binding factor, runt domain, alpha subunit 2; translocated to, 3	**1.5**	
CBFB	core-binding factor, beta subunit		**-2.3**
CBL	Cas-Br-M (murine) ecotropic retroviral transforming sequence		**1.6**
CBLB	Cas-Br-M (murine) ecotropic retroviral transforming sequence b	**1.8**	**-1.6**
CBLL1	Cas-Br-M (murine) ecotropic retroviral transforming sequence-like 1		**-2.2**
CBS	cystathionine-beta-synthase	**-2.6**	
CBX2	chromobox homolog 2 (Pc class homolog, Drosophila)	**-1.5**	**2.8**
CBX3	chromobox homolog 3 (HP1 gamma homolog, Drosophila)	**1.2**	**2.2**
CBX4	chromobox homolog 4 (Pc class homolog, Drosophila)	**-2.0**	
CBX5	chromobox homolog 5 (HP1 alpha homolog, Drosophila)		**-10.9**
CBX6	chromobox homolog 6	**-2.1**	**-1.4**
CCDC5	coiled-coil domain containing 5 (spindle associated)		**-1.6**
CCDC6	coiled-coil domain containing 6	**1.9**	**2.6**
CCL28	chemokine (C-C motif) ligand 28		**-4.5**
CCL3	chemokine (C-C motif) ligand 3	**1.4**	
CCL5	chemokine (C-C motif) ligand 5		**13.0**
CCNA2	cyclin A2	**1.3**	**-2.2**
CCNB1	cyclin B1	**1.9**	**-1.5**
CCNB2	cyclin B2	**1.4**	
CCNC	cyclin C		**1.3**
CCND2	cyclin D2		**65.9**
CCND3	cyclin D3		**-10.6**
CCNDBP1	cyclin D-type binding-protein 1	**1.4**	**-1.8**
CCNE1	cyclin E1		**2.3**

CCNE2	cyclin E2	**-1.3**	**-2.9**
CCNF	cyclin F	**2.0**	**-2.6**
CCNG1	cyclin G1	**1.3**	**1.8**
CCNG2	cyclin G2	**2.2**	
CCNH	cyclin H	**1.3**	**1.6**
CCNI	cyclin I	**1.5**	**-1.7**
CCNL2	cyclin L2		**1.4**
CCNT2	cyclin T2	**2.0**	**2.6**
CCRN4L	CCR4 carbon catabolite repression 4-like (S. cerevisiae)	**-1.7**	**1.4**
CCS	copper chaperone for superoxide dismutase		**-1.3**
CCT2	chaperonin containing TCP1, subunit 2 (beta)	**-2.2**	
CCT3	chaperonin containing TCP1, subunit 3 (gamma)	**-1.6**	**1.2**
CCT4	chaperonin containing TCP1, subunit 4 (delta)	**-1.4**	
CCT5	chaperonin containing TCP1, subunit 5 (epsilon)	**-1.5**	
CCT6A	chaperonin containing TCP1, subunit 6A (zeta 1)		**4.9**
CCT7	chaperonin containing TCP1, subunit 7 (eta)	**-1.5**	
CCT8	chaperonin containing TCP1, subunit 8 (theta)	**-1.6**	**1.2**
CD164	CD164 molecule, sialomucin	**1.8**	**-1.7**
CD19	CD19 molecule	**-2.8**	**-123.7**
CD200	CD200 molecule	**4.7**	**86.1**
CD24	CD24 molecule	**2.5**	**-34.4**
CD274	CD274 molecule	**-1.7**	
CD2BP2	CD2 (cytoplasmic tail) binding protein 2	**-1.4**	**-1.7**
CD34	CD34 molecule	**-3.0**	**12.3**
CD37	CD37 molecule		**2.5**
CD38	CD38 molecule	**-2.4**	**-2.9**
CD4	CD4 molecule		**5.4**
CD40	CD40 molecule, TNF receptor superfamily member 5		**-1.4**
CD44	CD44 molecule (Indian blood group)	**-3.0**	**3.2**
CD47	CD47 molecule	**-1.7**	**-2.4**
CD48	CD48 molecule	**-2.5**	**-14.5**
CD53	CD53 molecule	**3.1**	**1.4**
CD58	CD58 molecule	**3.0**	
CD59	CD59 molecule, complement regulatory protein		**-3.7**
CD63	CD63 molecule	**1.2**	**1.4**
CD69	CD69 molecule	**3.8**	**6.3**
CD72	CD72 molecule	**1.6**	**-23.3**
CD74	CD74 molecule, major histocompatibility complex, class II invariant chain		**-14.1**
CD79A	CD79a molecule, immunoglobulin-associated alpha	**1.3**	**-24.9**
CD79B	CD79b molecule, immunoglobulin-associated beta	**1.5**	**-2.1**
CD81	CD81 molecule		**-1.9**
CD83	CD83 molecule		**2.1**

CD84	CD84 molecule	**-1.5**	
CD9	CD9 molecule	**-1.9**	**-161.2**
CD96	CD96 molecule		**-2.0**
CD97	CD97 molecule	**3.0**	**5.6**
CDC14A	CDC14 cell division cycle 14 homolog A (S. cerevisiae)		**3.0**
CDC14B	--	**2.0**	
CDC16	cell division cycle 16 homolog (S. cerevisiae)	**1.2**	**1.6**
CDC2	cell division cycle 2, G1 to S and G2 to M	**2.0**	**-1.6**
CDC20	cell division cycle 20 homolog (S. cerevisiae)	**1.5**	
CDC23	cell division cycle 23 homolog (S. cerevisiae)	**-1.3**	**-1.9**
CDC25A	cell division cycle 25 homolog A (S. cerevisiae)	**-1.4**	**-1.6**
CDC25B	cell division cycle 25 homolog B (S. cerevisiae)	**1.6**	**-1.2**
CDC26	cell division cycle 26 homolog (S. cerevisiae)	**-1.4**	**-1.6**
CDC27	cell division cycle 27 homolog (S. cerevisiae)	**1.4**	
CDC2L2	cell division cycle 2-like 2 (PITSLRE proteins)	**1.5**	
CDC42	cell division cycle 42 (GTP binding protein, 25kDa)	**1.6**	**3.8**
CDC42EP3	CDC42 effector protein (Rho GTPase binding) 3	**4.0**	**-1.6**
CDC42EP4	CDC42 effector protein (Rho GTPase binding) 4		**3.9**
CDC42SE1	CDC42 small effector 1	**1.3**	**1.5**
CDC5L	CDC5 cell division cycle 5-like (S. pombe)	**1.3**	
CDC6	cell division cycle 6 homolog (S. cerevisiae)	**-2.9**	**-1.7**
CDC7	cell division cycle 7 homolog (S. cerevisiae)	**-1.4**	**-1.4**
CDCA1	NUF2, NDC80 kinetochore complex component, homolog (S. cerevisiae)	**1.6**	**-2.3**
CDCA3	cell division cycle associated 3		**-2.3**
CDCA5	cell division cycle associated 5		**-1.4**
CDCA7	cell division cycle associated 7	**-1.8**	
CDH2	cadherin 2, type 1, N-cadherin (neuronal)		**30.0**
CDH4	cadherin 4, type 1, R-cadherin (retinal)	**2.0**	
CDH6	cadherin 6, type 2, K-cadherin (fetal kidney)	**1.7**	
CDH8	cadherin 8, type 2		**-1.3**
CDIPT	CDP-diacylglycerol--inositol 3-phosphatidyltransferase (phosphatidylinositol synthase)	**1.4**	**-1.6**
CDK2	cyclin-dependent kinase 2	**-1.5**	**-2.3**
CDK4	cyclin-dependent kinase 4	**-2.4**	
CDK5R1	cyclin-dependent kinase 5, regulatory subunit 1 (p35)	**-1.5**	**-4.2**
CDK5RAP2	CDK5 regulatory subunit associated protein 2	**1.4**	
CDK5RAP3	CDK5 regulatory subunit associated protein 3		**1.4**
CDK6	cyclin-dependent kinase 6	**-1.5**	**-3.0**
CDK7	cyclin-dependent kinase 7 (MO15 homolog, Xenopus laevis, cdk-activating kinase)		**2.1**
CDK8	cyclin-dependent kinase 8		**-1.5**
CDK9	cyclin-dependent kinase 9 (CDC2-related kinase)	**1.7**	**-7.3**
CDKN1A	cyclin-dependent kinase inhibitor 1A (p21, Cip1)		**-36.4**
CDKN1B	cyclin-dependent kinase inhibitor 1B (p27, Kip1)	**1.6**	**-1.6**

CDKN1C	cyclin-dependent kinase inhibitor 1C (p57, Kip2)		**4.6**
CDKN2C	cyclin-dependent kinase inhibitor 2C (p18, inhibits CDK4)	**1.3**	
CDKN3	cyclin-dependent kinase inhibitor 3 (CDK2-associated dual specificity phosphatase)	**1.5**	**-1.4**
CDO1	cysteine dioxygenase, type I		**5.6**
CDS2	CDP-diacylglycerol synthase (phosphatidate cytidylyltransferase) 2	**1.6**	
CEACAM6	carcinoembryonic antigen-related cell adhesion molecule 6 (non-specific cross reacting antigen)	**-8.0**	
CEBPB	CCAAT/enhancer binding protein (C/EBP), beta	**-1.5**	**1.5**
CEBPD	CCAAT/enhancer binding protein (C/EBP), delta		**55.9**
CEBPE	CCAAT/enhancer binding protein (C/EBP), epsilon		**2.2**
CEBPG	CCAAT/enhancer binding protein (C/EBP), gamma		**-1.3**
CEBPZ	CCAAT/enhancer binding protein zeta	**-1.5**	**-1.7**
CECR5	cat eye syndrome chromosome region, candidate 5	**-1.5**	**-1.2**
CELSR1	cadherin, EGF LAG seven-pass G-type receptor 1 (flamingo homolog, Drosophila)		**1.9**
CENPA	centromere protein A	**1.7**	**-1.3**
CENPC1	centromere protein C 1	**1.3**	
CENPE	centromere protein E, 312kDa	**1.6**	
CENPF	centromere protein F, 350/400ka (mitosin)	**1.5**	**-2.3**
CENPH	centromere protein H		**-2.2**
CENPJ	centromere protein J		**-2.1**
CENTB1	centaurin, beta 1		**-21.1**
CENTB2	centaurin, beta 2	**1.5**	**1.5**
CENTD1	centaurin, delta 1	**1.8**	
CENTG2	centaurin, gamma 2	**-1.3**	
CEP63	centrosomal protein 63kDa	**1.2**	
CERK	ceramide kinase	**1.3**	
CETN3	centrin, EF-hand protein, 3 (CDC31 homolog, yeast)		**-1.5**
CFH	complement factor H		**-1.7**
CFL1	cofilin 1 (non-muscle)		**-1.6**
CFL2	cofilin 2 (muscle)	**-1.5**	**1.5**
CFLAR	CASP8 and FADD-like apoptosis regulator	**2.1**	**3.8**
CGI-121	TP53RK binding protein		**-1.3**
CGI-37	nuclear import 7 homolog (S. cerevisiae)	**-1.6**	
CHAF1A	chromatin assembly factor 1, subunit A (p150)	**-2.3**	
CHAF1B	chromatin assembly factor 1, subunit B (p60)		**-1.7**
CHC1	regulator of chromosome condensation 1	**-1.8**	
CHCHD7	coiled-coil-helix-coiled-coil-helix domain containing 7	**-1.4**	**-2.9**
CHD1	chromodomain helicase DNA binding protein 1	**-1.3**	
CHD2	chromodomain helicase DNA binding protein 2	**2.1**	**-1.9**
CHD3	chromodomain helicase DNA binding protein 3	**-1.3**	**-9.4**
CHD4	chromodomain helicase DNA binding protein 4	**-1.4**	
CHD8	chromodomain helicase DNA binding protein 8	**-1.3**	
CHEK1	CHK1 checkpoint homolog (S. pombe)	**-1.3**	**-1.8**

CHES1	checkpoint suppressor 1	**1.6**	**1.8**
CHGN	chondroitin beta1,4 N-acetylgalactosaminyltransferase	**2.3**	
CHI3L2	chitinase 3-like 2		**-3.1**
CHKA	choline kinase alpha	**5.5**	**2.2**
CHM	choroideremia (Rab escort protein 1)		**2.1**
CHML	choroideremia-like (Rab escort protein 2)	**1.8**	**-1.3**
CHMP1.5	chromatin modifying protein 1B		**1.8**
CHMP4A	chromatin modifying protein 4A		**-1.4**
CHMP4B	chromatin modifying protein 4B		**-1.6**
CHN2	chimerin (chimaerin) 2	**-1.4**	
CHP	calcium binding protein P22		**2.4**
CHPT1	choline phosphotransferase 1		**2.4**
CHRNA5	cholinergic receptor, nicotinic, alpha 5		**-2.2**
CHRNA6	cholinergic receptor, nicotinic, alpha 6	**-3.0**	
CHST10	carbohydrate sulfotransferase 10		**2.3**
CHST11	carbohydrate (chondroitin 4) sulfotransferase 11	**1.6**	**2.0**
CHST7	carbohydrate (N-acetylglucosamine 6-O) sulfotransferase 7	**11.7**	
CHSY1	carbohydrate (chondroitin) synthase 1	**2.5**	**1.8**
CHTF18	CTF18, chromosome transmission fidelity factor 18 homolog (S. cerevisiae)	**1.3**	**-1.4**
CHUK	conserved helix-loop-helix ubiquitous kinase		**1.5**
CIAPIN1	cytokine induced apoptosis inhibitor 1	**-1.6**	**1.3**
CIAS1	NLR family, pyrin domain containing 3		**-4.5**
CIRH1A	cirrhosis, autosomal recessive 1A (cirhin)	**-2.4**	**1.3**
CITED2	Cbp/p300-interacting transactivator, with Glu/Asp-rich carboxy-terminal domain, 2	**2.8**	**10.5**
CKAP2	cytoskeleton associated protein 2	**3.2**	**-1.3**
CKAP4	cytoskeleton-associated protein 4	**-1.9**	**-2.2**
CKB	creatine kinase, brain		**3.2**
CKIP-1	pleckstrin homology domain containing, family O member 1	**-2.3**	**-1.8**
CKLFSF3	CKLF-like MARVEL transmembrane domain containing 3	**1.5**	
CKMT1A	creatine kinase, mitochondrial 1A		**-2.0**
CKS1B	CDC28 protein kinase regulatory subunit 1B		**-1.6**
CKS2	CDC28 protein kinase regulatory subunit 2	**1.2**	**-1.3**
CLASP1	cytoplasmic linker associated protein 1		**1.5**
CLASP2	cytoplasmic linker associated protein 2	**1.7**	**-1.7**
CLCN3	chloride channel 3		**-3.0**
CLDN10	claudin 10	**-2.1**	
CLEC11A	C-type lectin domain family 11, member A	**1.4**	**1.5**
CLEC2D	--	**-3.4**	**-36.7**
CLEC5A	C-type lectin domain family 5, member A		**241.8**
CLIC1	chloride intracellular channel 1	**1.3**	**-1.3**
CLIC4	chloride intracellular channel 4		**-2.2**
CLK1	CDC-like kinase 1	**1.7**	**1.5**

CLK4	CDC-like kinase 4	**2.1**	**1.7**
CLTA	clathrin, light chain (Lca)		**1.4**
CLTB	clathrin, light chain (Lcb)	**-1.4**	**-2.0**
CLTC	clathrin, heavy chain (Hc)		**1.4**
CMAH	cytidine monophosphate-N-acetylneuraminic acid hydroxylase (CMP-N-acetylneuraminate monooxygenase)	**-1.4**	
CMIP	c-Maf-inducing protein	**-1.3**	
CNAP1	non-SMC condensin I complex, subunit D2		**-2.7**
CNO	cappuccino homolog (mouse)	**1.3**	**1.8**
CNOT1	CCR4-NOT transcription complex, subunit 1	**-1.2**	
CNOT7	CCR4-NOT transcription complex, subunit 7	**-1.4**	**1.7**
CNOT8	CCR4-NOT transcription complex, subunit 8		**-1.4**
CNP	2',3'-cyclic nucleotide 3' phosphodiesterase		**-1.3**
COBRA1	cofactor of BRCA1		**-1.3**
COG1	component of oligomeric golgi complex 1		**1.9**
COG3	component of oligomeric golgi complex 3	**2.3**	**1.8**
COG5	component of oligomeric golgi complex 5	**-1.3**	**1.3**
COG6	component of oligomeric golgi complex 6		**-1.6**
COG7	component of oligomeric golgi complex 7		**-1.8**
COL17A1	collagen, type XVII, alpha 1	**1.6**	**3.1**
COL1A1	collagen, type I, alpha 1		**-5.2**
COL4A3BP	collagen, type IV, alpha 3 (Goodpasture antigen) binding protein	**1.7**	
COL4A5	collagen, type IV, alpha 5 (Alport syndrome)		**10.8**
COL5A1	collagen, type V, alpha 1		**-1.3**
COL6A1	collagen, type VI, alpha 1	**2.1**	
COL6A3	collagen, type VI, alpha 3	**-1.2**	
COMMD1	copper metabolism (Murr1) domain containing 1	**1.3**	
COMMD10	COMM domain containing 10	**-1.4**	**-1.6**
COMMD2	COMM domain containing 2	**-1.4**	
COMMD5	--		**1.5**
COMMD6	COMM domain containing 6	**1.4**	**1.3**
COMMD8	COMM domain containing 8		**1.9**
COMT	catechol-O-methyltransferase		**-2.1**
COP1	caspase-1 dominant-negative inhibitor pseudo-ICE		**-8.8**
COPA	coatomer protein complex, subunit alpha		**1.2**
COPB	coatomer protein complex, subunit beta 1		**1.4**
COPB2	coatomer protein complex, subunit beta 2 (beta prime)		**1.2**
COPS2	COP9 constitutive photomorphogenic homolog subunit 2 (Arabidopsis)	**-1.2**	**1.6**
COPS3	COP9 constitutive photomorphogenic homolog subunit 3 (Arabidopsis)	**-1.2**	**-2.0**
COPS4	COP9 constitutive photomorphogenic homolog subunit 4 (Arabidopsis)	**-1.3**	
COPS6	COP9 constitutive photomorphogenic homolog subunit 6 (Arabidopsis)	**-1.5**	**-1.7**
COPS7A	COP9 constitutive photomorphogenic homolog subunit 7A (Arabidopsis)		**-1.4**
COPS7B	COP9 constitutive photomorphogenic homolog subunit 7B (Arabidopsis)		**-1.7**

COPS8	COP9 constitutive photomorphogenic homolog subunit 8 (Arabidopsis)		**-3.2**
COPZ1	coatomer protein complex, subunit zeta 1	**-1.4**	
COQ3	coenzyme Q3 homolog, methyltransferase (S. cerevisiae)		**1.9**
CORO1A	coronin, actin binding protein, 1A	**-1.7**	
CORO1C	coronin, actin binding protein, 1C		**-1.9**
CORO2B	coronin, actin binding protein, 2B		**-13.5**
COTL1	coactosin-like 1 (Dictyostelium)		**2.1**
COX10	COX10 homolog, cytochrome c oxidase assembly protein, heme A: farnesyltransferase (yeast)	**-1.9**	**-1.5**
COX11	COX11 homolog, cytochrome c oxidase assembly protein (yeast)	**-1.3**	**4.1**
COX15	COX15 homolog, cytochrome c oxidase assembly protein (yeast)		**8.8**
COX17	COX17 cytochrome c oxidase assembly homolog (S. cerevisiae)	**1.6**	
COX5B	cytochrome c oxidase subunit Vb	**-1.2**	
COX6A1	cytochrome c oxidase subunit VIa polypeptide 1		**-1.2**
COX7B	cytochrome c oxidase subunit VIIb	**-1.3**	
COX8A	cytochrome c oxidase subunit 8A (ubiquitous)		**-1.5**
CPD	carboxypeptidase D		**25.6**
CPM	carboxypeptidase M	**6.0**	**-2.2**
CPNE1	copine I		**-1.5**
CPNE3	copine III		**-1.5**
CPOX	coproporphyrinogen oxidase		**1.8**
CPSF1	cleavage and polyadenylation specific factor 1, 160kDa		**2.3**
CPSF2	cleavage and polyadenylation specific factor 2, 100kDa		**-1.3**
CPSF3	cleavage and polyadenylation specific factor 3, 73kDa		**1.2**
CPSF5	nudix (nucleoside diphosphate linked moiety X)-type motif 21	**-1.3**	**-1.8**
CPSF6	cleavage and polyadenylation specific factor 6, 68kDa		**-1.6**
CPT1A	carnitine palmitoyltransferase 1A (liver)		**1.6**
CRADD	CASP2 and RIPK1 domain containing adaptor with death domain	**1.5**	
CRAT	carnitine acetyltransferase	**1.8**	**6.6**
CREB1	cAMP responsive element binding protein 1	**1.4**	**-1.5**
CREB3	cAMP responsive element binding protein 3	**1.6**	
CREBBP	CREB binding protein (Rubinstein-Taybi syndrome)	**1.3**	**-10.0**
CREBL2	cAMP responsive element binding protein-like 2		**-2.6**
CREG1	cellular repressor of E1A-stimulated genes 1	**-1.5**	**1.8**
CREM	cAMP responsive element modulator		**7.7**
CRI1	EP300 interacting inhibitor of differentiation 1		**1.9**
CRIM1	cysteine rich transmembrane BMP regulator 1 (chordin-like)		**-12.7**
CRIP1	cysteine-rich protein 1 (intestinal)		**-16.0**
CRIPT	cysteine-rich PDZ-binding protein		**-1.2**
CRK	v-crk sarcoma virus CT10 oncogene homolog (avian)	**1.8**	
CRMP1	collapsin response mediator protein 1		**-4.8**
CROP	cisplatin resistance-associated overexpressed protein	**-1.5**	**1.9**
CRSP2	cofactor required for Sp1 transcriptional activation, subunit 2, 150kDa		**-1.9**

CRSP7	cofactor required for Sp1 transcriptional activation, subunit 7, 70kDa		**1.5**
CRSP8	cofactor required for Sp1 transcriptional activation, subunit 8, 34kDa		**-1.4**
CRSP9	cofactor required for Sp1 transcriptional activation, subunit 9, 33kDa	**2.2**	
CRYL1	crystallin, lambda 1		**1.4**
CRYZ	crystallin, zeta (quinone reductase)	**-1.4**	**-1.7**
CSAD	cysteine sulfinic acid decarboxylase		**1.5**
CSDA	cold shock domain protein A	**4.4**	
CSDE1	cold shock domain containing E1, RNA-binding	**1.7**	**1.7**
CSE1L	CSE1 chromosome segregation 1-like (yeast)	**-1.4**	**-1.6**
CSF2RB	colony stimulating factor 2 receptor, beta, 2		**-1.2**
CSF3R	colony stimulating factor 3 receptor (granulocyte)		**30.6**
CSK	c-src tyrosine kinase		**-1.6**
CSNK1A1	casein kinase 1, alpha 1	**1.5**	**2.0**
CSNK1D	casein kinase 1, delta	**1.3**	**1.4**
CSNK1E	casein kinase 1, epsilon	**1.7**	
CSNK1G2	casein kinase 1, gamma 2		**-1.2**
CSNK2A1	casein kinase 2, alpha 1 polypeptide	**-1.4**	**1.5**
CSNK2A2	casein kinase 2, alpha prime polypeptide	**1.9**	**1.9**
CSNK2B	casein kinase 2, beta polypeptide		**-1.3**
CSPG2	chondroitin sulfate proteoglycan 2 (versican)		**-2.1**
CSPG4	chondroitin sulfate proteoglycan 4 (melanoma-associated)		**-4.7**
CSPG6	structural maintenance of chromosomes 3	**-1.7**	**2.0**
CSRP2	cysteine and glycine-rich protein 2	**-1.8**	**-12.7**
CSRP2BP	CSRP2 binding protein		**1.6**
CST3	cystatin C (amyloid angiopathy and cerebral hemorrhage)		**7.9**
CST7	cystatin F (leukocystatin)	**2.4**	**-26.4**
CSTA	cystatin A (stefin A)		**2.5**
CSTB	cystatin B (stefin B)		**1.5**
CSTF1	cleavage stimulation factor, 3' pre-RNA, subunit 1, 50kDa	**1.3**	**1.6**
CSTF3	cleavage stimulation factor, 3' pre-RNA, subunit 3, 77kDa		**2.2**
CTBP1	C-terminal binding protein 1	**1.3**	**-1.5**
CTBP2	C-terminal binding protein 2	**1.2**	**1.8**
CTCF	CCCTC-binding factor (zinc finger protein)		**-1.2**
CTDSPL	CTD (carboxy-terminal domain, RNA polymerase II, polypeptide A) small phosphatase-like	**3.7**	**2.5**
CTGF	connective tissue growth factor	**56.6**	**-17.7**
CTH	cystathionase (cystathionine gamma-lyase)	**-2.2**	**1.5**
CTNNA1	catenin (cadherin-associated protein), alpha 1, 102kDa	**1.7**	
CTNNAL1	catenin (cadherin-associated protein), alpha-like 1		**-2.0**
CTNNB1	catenin (cadherin-associated protein), beta 1, 88kDa	**1.4**	**1.4**
CTNND1	catenin (cadherin-associated protein), delta 1	**2.0**	
CTPS	CTP synthase	**-1.4**	**-1.5**
CTPS2	CTP synthase II	**-1.3**	

CTSB	cathepsin B	**2.1**	**1.6**
CTSC	--	**-5.2**	**2.7**
CTSF	cathepsin F		**2.2**
CTSG	cathepsin G	**-1.5**	**10.6**
CTSH	cathepsin H		**-1.4**
CTSL	cathepsin L		**1.6**
CTSS	cathepsin S	**1.5**	
CTTN	cortactin	**-2.5**	**1.7**
CUGBP1	CUG triplet repeat, RNA binding protein 1	**-1.3**	**-1.8**
CUGBP2	CUG triplet repeat, RNA binding protein 2	**2.4**	**5.7**
CUL1	cullin 1		**-1.3**
CUL2	cullin 2		**3.6**
CUL3	cullin 3		**-2.3**
CUL4A	cullin 4A	**1.4**	**1.5**
CUL5	cullin 5		**1.4**
CUTL1	cut-like 1, CCAAT displacement protein (Drosophila)	**1.7**	**-1.5**
CUTL2	cut-like 2 (Drosophila)		**28.3**
CXCL16	chemokine (C-X-C motif) ligand 16		**-1.9**
CXCR4	chemokine (C-X-C motif) receptor 4	**1.8**	**-2.5**
CXORF40A	chromosome X open reading frame 40A		**-1.2**
CXORF53	BRCA1/BRCA2-containing complex, subunit 3		**-1.2**
CXXC5	CXXC finger 5	**-3.0**	**-5.0**
CYB5	cytochrome b5 type A (microsomal)	**2.3**	**-1.3**
CYB561	cytochrome b-561	**-2.0**	**1.8**
CYB5-M	cytochrome b5 type B (outer mitochondrial membrane)		**-1.4**
CYBA	cytochrome b-245, alpha polypeptide	**1.6**	
CYC1	cytochrome c-1	**-1.6**	**1.5**
CYCS	cytochrome c, somatic	**-1.6**	**4.9**
CYFIP1	cytoplasmic FMR1 interacting protein 1	**2.3**	**-2.0**
CYFIP2	cytoplasmic FMR1 interacting protein 2	**-2.8**	**-44.2**
CYLN2	CAP-GLY domain containing linker protein 2	**-2.3**	**-23.8**
CYP2C9	--		**1.4**
CYP2R1	cytochrome P450, family 2, subfamily R, polypeptide 1		**1.9**
CYP51A1	cytochrome P450, family 51, subfamily A, polypeptide 1		**1.3**
CYSLTR1	cysteinyl leukotriene receptor 1	**3.3**	**13.1**
DAAM1	dishevelled associated activator of morphogenesis 1	**1.7**	**-3.4**
DAB1	disabled homolog 1 (Drosophila)	**-1.6**	**1.6**
DAB2	disabled homolog 2, mitogen-responsive phosphoprotein (Drosophila)	**-2.4**	**50.8**
DACH1	dachshund homolog 1 (Drosophila)		**8.1**
DAD1	defender against cell death 1		**-4.1**
DAF	CD55 molecule, decay accelerating factor for complement (Cromer blood group)		**-1.5**
DAG1	dystroglycan 1 (dystrophin-associated glycoprotein 1)		**1.5**

DAP3	death associated protein 3		**1.9**
DAPK1	death-associated protein kinase 1		**3.4**
DAPK3	death-associated protein kinase 3	**-1.3**	**1.5**
DAPP1	dual adaptor of phosphotyrosine and 3-phosphoinositides		**-1.7**
DARS	aspartyl-tRNA synthetase		**1.3**
DATF1	death inducer-obliterator 1	**1.5**	**1.3**
DAZAP1	DAZ associated protein 1		**-1.4**
DAZAP2	DAZ associated protein 2		**-1.7**
DBI	diazepam binding inhibitor (GABA receptor modulator, acyl-Coenzyme A binding protein)		**-1.9**
DBN1	drebrin 1		**-1.5**
DBP	D site of albumin promoter (albumin D-box) binding protein	**-2.7**	
DBT	dihydrolipoamide branched chain transacylase E2	**1.4**	**1.3**
DCAL1	dendritic cell-associated lectin-1		**1.6**
DCI	dodecenoyl-Coenzyme A delta isomerase (3,2 trans-enoyl-Coenzyme A isomerase)	**-1.5**	
DCK	deoxycytidine kinase	**1.3**	**-1.9**
DCP1A	DCP1 decapping enzyme homolog A (S. cerevisiae)		**-2.0**
DCP1B	DCP1 decapping enzyme homolog B (S. cerevisiae)		**-2.9**
DCP2	DCP2 decapping enzyme homolog (S. cerevisiae)	**1.3**	**-1.5**
DCPS	decapping enzyme, scavenger		**1.4**
DCTN1	dynactin 1 (p150, glued homolog, Drosophila)		**1.3**
DCTN2	dynactin 2 (p50)	**1.5**	**1.4**
DCTN4	dynactin 4 (p62)	**3.8**	**1.4**
DCTN6	dynactin 6	**-1.4**	
DCXR	dicarbonyl/L-xylulose reductase	**-1.5**	**1.5**
DDA3	proline/serine-rich coiled-coil 1	**1.4**	**1.4**
DDAH1	dimethylarginine dimethylaminohydrolase 1		**204.1**
DDB1	damage-specific DNA binding protein 1, 127kDa	**-1.3**	
DDB2	damage-specific DNA binding protein 2, 48kDa		**-3.2**
DDEF1	development and differentiation enhancing factor 1	**-1.5**	**4.1**
DDEF2	development and differentiation enhancing factor 2	**1.3**	**-17.8**
DDHD1	--	**-1.4**	**-3.0**
DDIT4	DNA-damage-inducible transcript 4	**4.6**	**5.5**
DDOST	dolichyl-diphosphooligosaccharide-protein glycosyltransferase		**1.8**
DDR1	discoidin domain receptor family, member 1	**4.4**	
DDT	D-dopachrome tautomerase	**1.3**	**-1.9**
DDX1	DEAD (Asp-Glu-Ala-Asp) box polypeptide 1	**-1.3**	**1.2**
DDX11	DEAD/H (Asp-Glu-Ala-Asp/His) box polypeptide 11 (CHL1-like helicase homolog, S. cerevisiae)	**-1.5**	**-2.3**
DDX17	DEAD (Asp-Glu-Ala-Asp) box polypeptide 17		**7.2**
DDX18	DEAD (Asp-Glu-Ala-Asp) box polypeptide 18	**-1.7**	**-1.3**
DDX20	DEAD (Asp-Glu-Ala-Asp) box polypeptide 20	**-1.5**	**1.3**
DDX21	DEAD (Asp-Glu-Ala-Asp) box polypeptide 21	**-2.1**	**1.7**
DDX24	DEAD (Asp-Glu-Ala-Asp) box polypeptide 24	**-1.3**	

DDX39	DEAD (Asp-Glu-Ala-Asp) box polypeptide 39		-1.3
DDX3X	DEAD (Asp-Glu-Ala-Asp) box polypeptide 3, X-linked		-1.8
DDX42	DEAD (Asp-Glu-Ala-Asp) box polypeptide 42		1.6
DDX56	DEAD (Asp-Glu-Ala-Asp) box polypeptide 56		2.0
DDX6	DEAD (Asp-Glu-Ala-Asp) box polypeptide 6	2.1	-1.6
DEAF1	deformed epidermal autoregulatory factor 1 (Drosophila)	-2.0	
DECR1	2,4-dienoyl CoA reductase 1, mitochondrial		1.6
DEDD	death effector domain containing	1.5	
DEF6	differentially expressed in FDCP 6 homolog (mouse)	2.1	-1.6
DEGS1	degenerative spermatocyte homolog 1, lipid desaturase (Drosophila)	1.7	
DEK	DEK oncogene (DNA binding)		-1.9
DERA	2-deoxyribose-5-phosphate aldolase homolog (C. elegans)		-2.7
DET1	de-etiolated homolog 1 (Arabidopsis)		-2.0
DEXI	dexamethasone-induced transcript		-1.5
DF	complement factor D (adipsin)		77.5
DFFA	DNA fragmentation factor, 45kDa, alpha polypeptide	-2.0	1.6
DFFB	DNA fragmentation factor, 40kDa, beta polypeptide (caspase-activated DNase)	-1.9	
DGAT1	diacylglycerol O-acyltransferase homolog 1 (mouse)		3.6
DGCR6	DiGeorge syndrome critical region gene 6	-1.7	
DGCR8	--		-1.9
DGKD	diacylglycerol kinase, delta 130kDa		-2.5
DGKE	diacylglycerol kinase, epsilon 64kDa	-2.1	-1.9
DGKG	diacylglycerol kinase, gamma 90kDa		4.1
DHCR7	7-dehydrocholesterol reductase	-1.7	
DHFR	dihydrofolate reductase	-1.9	-1.7
DHODH	dihydroorotate dehydrogenase	-1.5	
DHPS	deoxyhypusine synthase		-1.5
DHRS1	dehydrogenase/reductase (SDR family) member 1		2.3
DHRS9	dehydrogenase/reductase (SDR family) member 9		5.8
DHX15	DEAH (Asp-Glu-Ala-His) box polypeptide 15	-1.6	-1.3
DHX16	DEAH (Asp-Glu-Ala-His) box polypeptide 16	-1.2	
DHX36	DEAH (Asp-Glu-Ala-His) box polypeptide 36	-2.2	-1.3
DHX9	DEAH (Asp-Glu-Ala-His) box polypeptide 9		-1.4
DIA1	cytochrome b5 reductase 3	1.3	
DIABLO	diablo homolog (Drosophila)	-1.5	-1.2
DIAPH1	diaphanous homolog 1 (Drosophila)	-1.4	-1.7
DIAPH3	diaphanous homolog 3 (Drosophila)		-1.6
DICER1	Dicer1, Dcr-1 homolog (Drosophila)		-1.6
DIP	death-inducing-protein	-1.4	-2.3
DISC1	disrupted in schizophrenia 1	1.7	2.8
DISP1	dispatched homolog 1 (Drosophila)	-1.5	
DKC1	dyskeratosis congenita 1, dyskerin	-2.6	

DKFZP564K142	implantation-associated protein	1.7	2.2
DKFZP762E1312	hypothetical protein DKFZp762E1312	1.2	-2.1
DKK1	dickkopf homolog 1 (Xenopus laevis)	21.8	
DLAT	dihydrolipoamide S-acetyltransferase (E2 component of pyruvate dehydrogenase complex)	-2.1	-1.6
DLC2	dynein, light chain, LC8-type 2	-1.3	-2.1
DLD	dihydrolipoamide dehydrogenase	-1.3	
DLEU1	deleted in lymphocytic leukemia, 1	-1.8	1.5
DLEU2	--	-1.3	-3.3
DLG1	discs, large homolog 1 (Drosophila)	-1.9	-1.5
DLG3	discs, large homolog 3 (neuroendocrine-dlg, Drosophila)	-1.4	
DLG5	discs, large homolog 5 (Drosophila)	2.0	2.2
DLG7	discs, large homolog 7 (Drosophila)		-1.3
DLK1	delta-like 1 homolog (Drosophila)		3.2
DMC1	DMC1 dosage suppressor of mck1 homolog, meiosis-specific homologous recombination (yeast)		-3.0
DMD	dystrophin (muscular dystrophy, Duchenne and Becker types)	-2.8	
DMN	desmuslin		2.3
DMXL1	Dmx-like 1	1.8	2.2
DMXL2	Dmx-like 2		1.7
DNAH3	dynein, axonemal, heavy chain 3		-2.9
DNAJA1	DnaJ (Hsp40) homolog, subfamily A, member 1	-1.4	-1.7
DNAJA2	DnaJ (Hsp40) homolog, subfamily A, member 2		15.8
DNAJA3	DnaJ (Hsp40) homolog, subfamily A, member 3	-2.1	-1.6
DNAJB1	DnaJ (Hsp40) homolog, subfamily B, member 1		-1.9
DNAJB6	DnaJ (Hsp40) homolog, subfamily B, member 6		-1.7
DNAJB9	DnaJ (Hsp40) homolog, subfamily B, member 9		-1.5
DNAJC1	DnaJ (Hsp40) homolog, subfamily C, member 1	2.6	2.8
DNAJC10	DnaJ (Hsp40) homolog, subfamily C, member 10	-1.4	1.7
DNAJC11	DnaJ (Hsp40) homolog, subfamily C, member 11	1.9	1.4
DNAJC14	DnaJ (Hsp40) homolog, subfamily C, member 14		1.3
DNAJC3	DnaJ (Hsp40) homolog, subfamily C, member 3		1.7
DNAJC7	DnaJ (Hsp40) homolog, subfamily C, member 7	-1.4	1.3
DNASE1L3	deoxyribonuclease I-like 3	-1.4	
DNCH1	dynein, cytoplasmic 1, heavy chain 1	4.0	
DNCI2	dynein, cytoplasmic 1, intermediate chain 2	1.4	2.9
DNCL2A	dynein, light chain, roadblock-type 1	1.3	
DNCLIC2	dynein, cytoplasmic 1, light intermediate chain 2	1.5	
DNMT1	DNA (cytosine-5-)-methyltransferase 1	-1.4	
DNMT3A	DNA (cytosine-5-)-methyltransferase 3 alpha	-1.5	1.6
DNMT3B	DNA (cytosine-5-)-methyltransferase 3 beta		1.3
DNTT	deoxynucleotidyltransferase, terminal	-1.7	-17.1
DOC1	filamin A interacting protein 1-like	-1.5	1.8
DOC-1R	CDK2-associated protein 2	2.0	-2.0

DOCK1	dedicator of cytokinesis 1	**-1.3**	
DOCK11	dedicator of cytokinesis 11	**1.5**	**1.6**
DOCK2	dedicator of cytokinesis 2		**1.8**
DOCK4	dedicator of cytokinesis 4		**1.8**
DOCK7	dedicator of cytokinesis 7		**2.4**
DOCK9	dedicator of cytokinesis 9	**5.7**	
DOK1	docking protein 1, 62kDa (downstream of tyrosine kinase 1)		**2.3**
DOK3	docking protein 3	**-1.6**	**-2.0**
DOLPP1	dolichyl pyrophosphate phosphatase 1	**-1.6**	**-1.3**
DONSON	downstream neighbor of SON		**-1.2**
DOT1L	DOT1-like, histone H3 methyltransferase (S. cerevisiae)	**-1.7**	
DPAGT1	dolichyl-phosphate (UDP-N-acetylglucosamine) N-acetylglucosaminephosphotransferase 1 (GlcNAc-1-P transferase)	**-1.8**	
DPEP1	dipeptidase 1 (renal)	**13.0**	
DPH2L1	DPH1 homolog (S. cerevisiae)		**-1.7**
DPM3	dolichyl-phosphate mannosyltransferase polypeptide 3		**-1.5**
DPP8	dipeptidyl-peptidase 8		**-2.1**
DPT	dermatopontin		**-22.5**
DPYD	dihydropyrimidine dehydrogenase		**-8.8**
DPYSL2	dihydropyrimidinase-like 2	**3.1**	**-4.2**
DR1	down-regulator of transcription 1, TBP-binding (negative cofactor 2)		**-1.6**
DRAP1	DR1-associated protein 1 (negative cofactor 2 alpha)	**-2.2**	**-1.5**
DRE1	kelch-like 24 (Drosophila)	**2.5**	**1.3**
DSCR1	Down syndrome critical region gene 1	**7.7**	**1.5**
DSG2	desmoglein 2		**86.2**
DST	dystonin		**-8.3**
DSTN	destrin (actin depolymerizing factor)		**3.9**
DTNBP1	dystrobrevin binding protein 1	**-1.6**	**-1.5**
DTX1	deltex homolog 1 (Drosophila)	**-1.7**	
DTX3L	deltex 3-like (Drosophila)		**-1.5**
DTX4	deltex 4 homolog (Drosophila)		**-2.2**
DTYMK	deoxythymidylate kinase (thymidylate kinase)		**-1.9**
DULLARD	dullard homolog (Xenopus laevis)	**1.8**	**-1.4**
DUSP1	dual specificity phosphatase 1	**3.2**	**-1.7**
DUSP10	dual specificity phosphatase 10		**-1.4**
DUSP11	dual specificity phosphatase 11 (RNA/RNP complex 1-interacting)	**-1.3**	
DUSP16	dual specificity phosphatase 16		**-1.9**
DUSP22	dual specificity phosphatase 22	**1.4**	**-1.3**
DUSP3	dual specificity phosphatase 3 (vaccinia virus phosphatase VH1-related)		**6.4**
DUSP6	dual specificity phosphatase 6	**1.7**	**15.8**
DUSP7	dual specificity phosphatase 7	**-1.2**	
DUT	dUTP pyrophosphatase	**-2.0**	
DVL2	dishevelled, dsh homolog 2 (Drosophila)		**-1.6**

DVL3	dishevelled, dsh homolog 3 (Drosophila)		**-1.6**
DYRK1A	dual-specificity tyrosine-(Y)-phosphorylation regulated kinase 1A	**-1.6**	
E2F1	E2F transcription factor 1		**-1.7**
E2F2	E2F transcription factor 2		**-11.0**
E2F3	E2F transcription factor 3	**1.6**	
E2F6	E2F transcription factor 6	**-1.3**	
E2F7	E2F transcription factor 7	**-1.2**	**-2.2**
E2IG5	--	**-4.6**	**2.1**
E4F1	E4F transcription factor 1		**-1.4**
EAF1	ELL associated factor 1	**1.4**	
EAF2	ELL associated factor 2		**1.6**
EARS2	glutamyl-tRNA synthetase 2 (mitochondrial)(putative)	**-2.8**	**-1.6**
EBAG9	estrogen receptor binding site associated, antigen, 9		**-1.8**
EBF	early B-cell factor 1	**-1.4**	**-420.0**
EBNA1BP2	EBNA1 binding protein 2	**-1.8**	
EBP	emopamil binding protein (sterol isomerase)		**-2.1**
ECHS1	enoyl Coenzyme A hydratase, short chain, 1, mitochondrial	**-1.4**	
ECM1	extracellular matrix protein 1	**1.7**	**1.6**
ECT2	epithelial cell transforming sequence 2 oncogene	**2.0**	
EDD1	E3 ubiquitin protein ligase, HECT domain containing, 1	**1.4**	**-1.4**
EDEM1	ER degradation enhancer, mannosidase alpha-like 1	**-1.2**	**-1.5**
EDEM2	ER degradation enhancer, mannosidase alpha-like 2		**1.7**
EDF1	endothelial differentiation-related factor 1	**1.4**	
EDG1	endothelial differentiation, sphingolipid G-protein-coupled receptor, 1	**5.1**	
EDG2	endothelial differentiation, lysophosphatidic acid G-protein-coupled receptor, 2		**53.1**
EDIL3	EGF-like repeats and discoidin I-like domains 3		**1.5**
EDNRB	endothelin receptor type B	**101.4**	
EED	embryonic ectoderm development		**-1.9**
EEF1A1	eukaryotic translation elongation factor 1 alpha 1		**4.9**
EEF1D	eukaryotic translation elongation factor 1 delta (guanine nucleotide exchange protein)		**1.6**
EEF1E1	eukaryotic translation elongation factor 1 epsilon 1	**-3.3**	**-1.6**
EEF2	eukaryotic translation elongation factor 2		**1.3**
EEF2K	eukaryotic elongation factor-2 kinase	**-2.8**	**-1.7**
EFEMP1	EGF-containing fibulin-like extracellular matrix protein 1	**-1.9**	**-8.2**
EFNB2	ephrin-B2	**-2.8**	**-5.9**
EFTUD2	elongation factor Tu GTP binding domain containing 2	**-1.3**	
EGLN1	egl nine homolog 1 (C. elegans)	**1.5**	**1.6**
EGR1	early growth response 1	**-2.0**	**-3.3**
EHBP1L1	EH domain binding protein 1-like 1		**-1.7**
EHD1	EH-domain containing 1		**1.6**
EHD4	EH-domain containing 4		**-1.4**
EHHADH	enoyl-Coenzyme A, hydratase/3-hydroxyacyl Coenzyme A dehydrogenase		**14.5**

EI24	etoposide induced 2.4 mRNA	**-1.5**	**1.3**
EIF1AX	eukaryotic translation initiation factor 1A, X-linked	**-1.5**	**-2.2**
EIF2A	eukaryotic translation initiation factor 2A, 65kDa		**1.3**
EIF2AK1	eukaryotic translation initiation factor 2-alpha kinase 1	**-1.4**	
EIF2AK2	eukaryotic translation initiation factor 2-alpha kinase 2	**-1.5**	
EIF2AK3	eukaryotic translation initiation factor 2-alpha kinase 3		**-1.5**
EIF2AK4	eukaryotic translation initiation factor 2 alpha kinase 4	**1.3**	**-1.5**
EIF2B1	eukaryotic translation initiation factor 2B, subunit 1 alpha, 26kDa		**-1.2**
EIF2B3	eukaryotic translation initiation factor 2B, subunit 3 gamma, 58kDa	**-2.0**	
EIF2C2	eukaryotic translation initiation factor 2C, 2	**-1.6**	**2.6**
EIF2S1	eukaryotic translation initiation factor 2, subunit 1 alpha, 35kDa	**-1.6**	**-1.2**
EIF2S3	eukaryotic translation initiation factor 2, subunit 3 gamma, 52kDa		**1.3**
EIF3S1	eukaryotic translation initiation factor 3, subunit 1 alpha, 35kDa	**-1.2**	**-1.5**
EIF3S10	eukaryotic translation initiation factor 3, subunit 10 theta, 150/170kDa	**-1.3**	**3.9**
EIF3S12	eukaryotic translation initiation factor 3, subunit 12	**-1.3**	
EIF3S2	eukaryotic translation initiation factor 3, subunit 2 beta, 36kDa	**-1.6**	
EIF3S5	eukaryotic translation initiation factor 3, subunit 5 epsilon, 47kDa	**1.2**	
EIF3S6	eukaryotic translation initiation factor 3, subunit 6 48kDa	**-1.6**	**-1.5**
EIF3S6IP	eukaryotic translation initiation factor 3, subunit 6 interacting protein	**1.2**	**1.3**
EIF3S7	eukaryotic translation initiation factor 3, subunit 7 zeta, 66/67kDa		**1.3**
EIF3S8	eukaryotic translation initiation factor 3, subunit 8, 110kDa	**-1.4**	**-3.1**
EIF3S9	eukaryotic translation initiation factor 3, subunit 9 eta, 116kDa	**-2.0**	**-1.2**
EIF4A1	eukaryotic translation initiation factor 4A, isoform 1	**-1.8**	
EIF4A2	eukaryotic translation initiation factor 4A, isoform 2		**1.4**
EIF4B	eukaryotic translation initiation factor 4B	**-2.4**	**1.8**
EIF4E	eukaryotic translation initiation factor 4E		**-1.3**
EIF4E2	eukaryotic translation initiation factor 4E family member 2	**1.2**	**-1.6**
EIF4EBP1	eukaryotic translation initiation factor 4E binding protein 1	**-3.1**	
EIF4EBP2	eukaryotic translation initiation factor 4E binding protein 2	**-1.3**	
EIF4G3	eukaryotic translation initiation factor 4 gamma, 3	**-1.2**	
EIF5	eukaryotic translation initiation factor 5		**1.2**
EIF5A	eukaryotic translation initiation factor 5A	**-2.0**	**-10.2**
EIF5A2	eukaryotic translation initiation factor 5A2		**7.7**
EIF5B	eukaryotic translation initiation factor 5B	**-1.6**	**2.5**
ELA2	elastase 2, neutrophil		**1680.7**
ELAVL1	ELAV (embryonic lethal, abnormal vision, Drosophila)-like 1 (Hu antigen R)	**1.6**	**-1.4**
ELF1	E74-like factor 1 (ets domain transcription factor)	**1.4**	**-1.8**
ELF2	E74-like factor 2 (ets domain transcription factor)		**-1.8**
ELF4	E74-like factor 4 (ets domain transcription factor)	**1.5**	**-1.2**
ELK3	ELK3, ETS-domain protein (SRF accessory protein 2)		**5.2**
ELL2	elongation factor, RNA polymerase II, 2	**4.3**	**3.0**
ELMO1	engulfment and cell motility 1	**2.1**	**3.6**

ELMO2	engulfment and cell motility 2	1.3	1.7
ELOVL6	ELOVL family member 6, elongation of long chain fatty acids (FEN1/Elo2, SUR4/Elo3-like, yeast)	-3.8	-3.4
ELP3	elongation protein 3 homolog (S. cerevisiae)		1.5
EMB	embigin homolog (mouse)		-1.8
EML4	echinoderm microtubule associated protein like 4		-1.3
EMP1	epithelial membrane protein 1	4.5	
EMP2	epithelial membrane protein 2	1.6	
ENAH	enabled homolog (Drosophila)	-1.5	1.2
ENC1	ectodermal-neural cortex (with BTB-like domain)	3.7	1.9
ENDOG	endonuclease G		1.4
ENO1	enolase 1, (alpha)	-1.5	
ENTH	clathrin interactor 1	1.9	2.5
ENTPD1	ectonucleoside triphosphate diphosphohydrolase 1		2.3
ENTPD4	ectonucleoside triphosphate diphosphohydrolase 4	1.3	1.5
EP400	E1A binding protein p400	1.4	1.3
EPAS1	endothelial PAS domain protein 1	15.9	
EPC1	enhancer of polycomb homolog 1 (Drosophila)	1.8	1.5
EPHA4	EPH receptor A4	28.2	
EPIM	syntaxin 2		1.3
EPLIN	LIM domain and actin binding 1	-3.1	-1.6
EPM2A	epilepsy, progressive myoclonus type 2A, Lafora disease (laforin)		-2.2
EPOR	erythropoietin receptor		16.6
EPRS	glutamyl-prolyl-tRNA synthetase	-1.7	1.3
EPS15	epidermal growth factor receptor pathway substrate 15	1.8	1.6
EPS15L1	epidermal growth factor receptor pathway substrate 15-like 1		1.7
ERBB2	v-erb-b2 erythroblastic leukemia viral oncogene homolog 2, neuro/glioblastoma derived oncogene homolog (avian)		-6.1
ERBB2IP	erbb2 interacting protein	1.3	-3.6
ERCC1	excision repair cross-complementing rodent repair deficiency, complementation group 1 (includes overlapping antisense sequence)	1.3	-1.8
ERCC3	excision repair cross-complementing rodent repair deficiency, complementation group 3 (xeroderma pigmentosum group B complementing)	-1.3	
ERCC5	excision repair cross-complementing rodent repair deficiency, complementation group 5 (xeroderma pigmentosum, complementation group G (Cockayne syndrome))		-1.4
ERCC6	excision repair cross-complementing rodent repair deficiency, complementation group 6	-1.5	
ERCC8	excision repair cross-complementing rodent repair deficiency, complementation group 8	-1.8	
ERF	Ets2 repressor factor	-1.5	2.1
ERG	v-ets erythroblastosis virus E26 oncogene homolog (avian)	1.8	-2.7
ERO1L	ERO1-like (S. cerevisiae)	-1.9	-1.3
ERP29	endoplasmic reticulum protein 29	-1.3	1.4
ESD	esterase D/formylglutathione hydrolase	1.7	2.3
ESPL1	extra spindle pole bodies homolog 1 (S. cerevisiae)	1.3	
ESRRA	estrogen-related receptor alpha		1.4
ETF1	eukaryotic translation termination factor 1	-1.6	1.3
ETFA	electron-transfer-flavoprotein, alpha polypeptide (glutaric aciduria II)		-1.3
ETFB	electron-transfer-flavoprotein, beta polypeptide	2.1	

ETNK1	ethanolamine kinase 1		-2.9
ETS1	v-ets erythroblastosis virus E26 oncogene homolog 1 (avian)		-29.1
ETS2	v-ets erythroblastosis virus E26 oncogene homolog 2 (avian)	5.1	-1.5
ETV5	ets variant gene 5 (ets-related molecule)	1.9	
ETV6	ets variant gene 6 (TEL oncogene)	-1.6	2.5
EVER1	transmembrane channel-like 6	1.6	
EVL	Enah/Vasp-like	-1.8	-1.8
EXO1	exonuclease 1	-2.1	-1.6
EXOC7	exocyst complex component 7		2.4
EXOC8	exocyst complex component 8	1.4	
EXOSC1	exosome component 1	1.3	
EXOSC2	exosome component 2	-2.4	
EXOSC3	exosome component 3		-1.4
EXOSC4	exosome component 4	-1.3	9.7
EXOSC8	exosome component 8		-2.4
EXOSC9	exosome component 9	-1.9	
EXT1	exostoses (multiple) 1	-1.8	
EXT2	exostoses (multiple) 2		2.3
EXTL2	exostoses (multiple)-like 2	-2.0	1.5
EZH1	enhancer of zeste homolog 1 (Drosophila)	-1.4	1.3
EZH2	enhancer of zeste homolog 2 (Drosophila)		-1.6
F11R	F11 receptor	-1.4	5.5
F13A1	coagulation factor XIII, A1 polypeptide	4.2	-2.3
F2R	coagulation factor II (thrombin) receptor	4.4	
F2RL3	coagulation factor II (thrombin) receptor-like 3	2.1	
F3	coagulation factor III (thromboplastin, tissue factor)	87.1	
F8A1	coagulation factor VIII-associated (intronic transcript) 1		1.6
FABP5	fatty acid binding protein 5 (psoriasis-associated)	-4.4	-2.2
FADS1	fatty acid desaturase 1	-1.5	-1.5
FADS2	fatty acid desaturase 2	-2.0	-1.9
FADS3	fatty acid desaturase 3		-3.1
FAF1	Fas (TNFRSF6) associated factor 1	-1.3	1.2
FAH	fumarylacetoacetate hydrolase (fumarylacetoacetase)		1.6
FAIM	Fas apoptotic inhibitory molecule	-1.5	-11.1
FALZ	bromodomain PHD finger transcription factor	-1.5	1.6
FAM38A	family with sequence similarity 38, member A	2.6	2.6
FAM3C	family with sequence similarity 3, member C	2.0	-2.2
FAM60A	family with sequence similarity 60, member A	-2.0	-1.9
FANCA	Fanconi anemia, complementation group A	-2.4	-2.2
FANCC	Fanconi anemia, complementation group C		-1.4
FANCF	Fanconi anemia, complementation group F	-1.7	-1.7
FANCG	Fanconi anemia, complementation group G	-1.3	

FANCL	Fanconi anemia, complementation group L		**1.6**
FARP2	FERM, RhoGEF and pleckstrin domain protein 2	**1.6**	**-2.0**
FARSLA	phenylalanine-tRNA synthetase-like, alpha subunit	**-2.1**	**1.6**
FARSLB	phenylalanine-tRNA synthetase-like, beta subunit	**-2.2**	**-1.5**
FASN	fatty acid synthase	**-3.2**	
FBL	fibrillarin	**-1.2**	**1.4**
FBLN1	fibulin 1		**-1.6**
FBN2	fibrillin 2 (congenital contractural arachnodactyly)		**27.4**
FBP1	fructose-1,6-bisphosphatase 1		**-5.3**
FBXL18	F-box and leucine-rich repeat protein 18	**-1.7**	
FBXL3	F-box and leucine-rich repeat protein 3	**1.4**	
FBXL5	F-box and leucine-rich repeat protein 5		**1.5**
FBXO18	F-box protein, helicase, 18	**3.0**	
FBXO38	F-box protein 38	**1.3**	**1.3**
FBXO42	F-box protein 42		**1.9**
FBXO5	F-box protein 5	**-1.3**	**-2.0**
FBXO6	F-box protein 6		**1.4**
FBXO9	F-box protein 9	**-1.4**	**2.2**
FBXW11	F-box and WD-40 domain protein 11	**1.3**	**1.3**
FBXW2	F-box and WD-40 domain protein 2		**1.9**
FBXW7	F-box and WD-40 domain protein 7 (archipelago homolog, Drosophila)	**3.9**	**1.3**
FCER1A	Fc fragment of IgE, high affinity I, receptor for; alpha polypeptide		**-1.4**
FCER1G	Fc fragment of IgE, high affinity I, receptor for; gamma polypeptide		**1.7**
FCGR1A	Fc fragment of IgG, high affinity Ia, receptor (CD64)		**16.0**
FCGR2A	Fc fragment of IgG, low affinity IIa, receptor (CD32)		**-2.0**
FCGRT	Fc fragment of IgG, receptor, transporter, alpha		**2.7**
FCHSD2	FCH and double SH3 domains 2	**3.0**	**1.9**
FDFT1	farnesyl-diphosphate farnesyltransferase 1		**1.4**
FDPS	farnesyl diphosphate synthase (farnesyl pyrophosphate synthetase, dimethylallyltranstransferase, geranyltranstransferase)		**-2.3**
FDX1	ferredoxin 1	**2.0**	**-2.0**
FEM1A	fem-1 homolog a (C. elegans)		**2.1**
FEM1B	fem-1 homolog b (C. elegans)	**1.3**	
FEN1	flap structure-specific endonuclease 1	**-1.8**	**-1.3**
FER1L3	fer-1-like 3, myoferlin (C. elegans)	**19.8**	
FEZ2	fasciculation and elongation protein zeta 2 (zygin II)		**1.5**
FGD2	FYVE, RhoGEF and PH domain containing 2	**3.3**	
FGF13	fibroblast growth factor 13		**1.9**
FGF18	fibroblast growth factor 18		**-1.9**
FGF7	fibroblast growth factor 7 (keratinocyte growth factor)	**-1.6**	**-2.0**
FGF9	fibroblast growth factor 9 (glia-activating factor)	**-2.1**	**-45.7**
FGFR1	fibroblast growth factor receptor 1 (fms-related tyrosine kinase 2, Pfeiffer syndrome)		**7.4**
FH	fumarate hydratase	**-2.1**	**-1.4**

FHL1	four and a half LIM domains 1	**1.8**	**-2.1**
FHOD1	formin homology 2 domain containing 1	**1.9**	**1.9**
FIBP	fibroblast growth factor (acidic) intracellular binding protein	**-1.3**	**-1.8**
FIP1L1	FIP1 like 1 (S. cerevisiae)	**1.8**	
FKBP1A	FK506 binding protein 1A, 12kDa	**-1.3**	**1.5**
FKBP4	FK506 binding protein 4, 59kDa		**3.0**
FKBP5	FK506 binding protein 5	**21.4**	**7.0**
FKSG14	centromere protein K		**-2.2**
FLAD1	FAD1 flavin adenine dinucleotide synthetase homolog (S. cerevisiae)	**-1.5**	
FLCN	folliculin	**1.6**	**1.3**
FLI1	Friend leukemia virus integration 1	**1.6**	
FLII	flightless I homolog (Drosophila)		**-1.4**
FLJ10287	coiled-coil domain containing 76		**1.4**
FLJ11730	chromosome 1 open reading frame 149		**-1.5**
FLJ12443	acyltransferase like 2		**-1.6**
FLJ12750	vacuolar protein sorting 37 homolog B (S. cerevisiae)	**22.1**	
FLJ20364	coiled-coil domain containing 99	**1.2**	
FLJ20516	TIMELESS interacting protein	**-2.4**	**-1.4**
FLJ20701	hypothetical protein FLJ20701	**4.0**	
FLJ20729	chromosome 1 open reading frame 181	**-1.6**	
FLJ20758	Pentatricopeptide repeat domain 3	**-1.3**	**1.8**
FLJ22794	family with sequence similarity 111, member A		**-1.5**
FLJ30092	AF-1 specific protein phosphatase	**1.6**	
FLJ38973	hypothetical protein FLJ38973	**1.9**	**2.1**
FLNA	filamin A, alpha (actin binding protein 280)		**-2.2**
FLNB	filamin B, beta (actin binding protein 278)		**1.6**
FLOT1	flotillin 1		**-2.4**
FLOT2	flotillin 2		**-3.6**
FLRT1	fibronectin leucine rich transmembrane protein 1	**-2.7**	
FLRT2	fibronectin leucine rich transmembrane protein 2		**5.1**
FLT1	fms-related tyrosine kinase 1 (vascular endothelial growth factor/vascular permeability factor receptor)	**2.6**	
FLT3	fms-related tyrosine kinase 3		**-1.7**
FMO2	flavin containing monooxygenase 2 (non-functional)	**-2.0**	
FMO5	flavin containing monooxygenase 5	**1.9**	**4.8**
FMR1	fragile X mental retardation 1	**1.4**	**-2.3**
FN1	fibronectin 1		**-2.8**
FNBP1	formin binding protein 1	**-2.9**	**-17.0**
FNBP1L	formin binding protein 1-like	**3.0**	**1.4**
FNBP3	PRP40 pre-mRNA processing factor 40 homolog A (yeast)	**-2.1**	**2.2**
FNTA	farnesyltransferase, CAAX box, alpha		**1.4**
FOLR1	folate receptor 1 (adult)	**1.6**	**1.6**
FOSL1	FOS-like antigen 1		**2.0**

FOSL2	FOS-like antigen 2	**4.0**	**7.1**
FOXM1	forkhead box M1	**1.4**	**-3.5**
FOXO1A	forkhead box O1A (rhabdomyosarcoma)	**2.1**	**-2.4**
FOXO3A	forkhead box O3A	**2.7**	**1.7**
FOXP1	forkhead box P1	**-1.8**	**1.4**
FPGS	folylpolyglutamate synthase		**-1.9**
FPGT	fucose-1-phosphate guanylyltransferase	**-1.2**	
FRAT1	frequently rearranged in advanced T-cell lymphomas		**4.5**
FRAT2	frequently rearranged in advanced T-cell lymphomas 2		**1.4**
FRMD4B	FERM domain containing 4B	**1.4**	**-34.9**
FRMD6	FERM domain containing 6		**18.3**
FSCN1	fascin homolog 1, actin-bundling protein (Strongylocentrotus purpuratus)	**1.6**	**1.7**
FST	follistatin	**30.6**	
FTH1	ferritin, heavy polypeptide 1	**1.5**	**-2.0**
FTL	ferritin, light polypeptide	**-1.4**	**1.9**
FUBP1	far upstream element (FUSE) binding protein 1	**-1.8**	**1.7**
FUS	fusion (involved in t(12;16) in malignant liposarcoma)	**-1.5**	**-2.6**
FUSIP1	FUS interacting protein (serine/arginine-rich) 1	**-1.9**	**-1.5**
FUT4	fucosyltransferase 4 (alpha (1,3) fucosyltransferase, myeloid-specific)	**1.7**	**1.3**
FUT7	fucosyltransferase 7 (alpha (1,3) fucosyltransferase)	**-1.8**	
FUT8	fucosyltransferase 8 (alpha (1,6) fucosyltransferase)		**-1.4**
FVT1	follicular lymphoma variant translocation 1	**-1.6**	
FXC1	fracture callus 1 homolog (rat)		**1.5**
FXR1	fragile X mental retardation, autosomal homolog 1	**1.4**	**1.4**
FXR2	fragile X mental retardation, autosomal homolog 2	**1.4**	
FXYD5	FXYD domain containing ion transport regulator 5	**-1.7**	**1.6**
FYB	FYN binding protein (FYB-120/130)		**11.8**
FYN	FYN oncogene related to SRC, FGR, YES		**-8.6**
FZD3	frizzled homolog 3 (Drosophila)		**-2.9**
FZD6	frizzled homolog 6 (Drosophila)	**2.1**	**10.1**
FZD8	frizzled homolog 8 (Drosophila)	**3.8**	
FZR1	fizzy/cell division cycle 20 related 1 (Drosophila)		**-1.9**
G1P2	ISG15 ubiquitin-like modifier		**-3.1**
G1P3	interferon, alpha-inducible protein 6	**-2.6**	
G22P1	X-ray repair complementing defective repair in Chinese hamster cells 6 (Ku autoantigen, 70kDa)	**-1.4**	
G3BP	GTPase activating protein (SH3 domain) binding protein 1	**-2.2**	**1.4**
G3BP2	GTPase activating protein (SH3 domain) binding protein 2		**1.3**
G6PD	glucose-6-phosphate dehydrogenase	**1.3**	
GAA	glucosidase, alpha; acid (Pompe disease, glycogen storage disease type II)	**-2.7**	**8.6**
GAB1	GRB2-associated binding protein 1	**2.0**	**-2.4**
GAB2	GRB2-associated binding protein 2	**1.9**	**-3.2**
GABARAP	GABA(A) receptor-associated protein		**-1.3**

GABBR1	gamma-aminobutyric acid (GABA) B receptor, 1		**-1.6**
GABPA	GA binding protein transcription factor, alpha subunit 60kDa	**1.3**	**-1.5**
GABPB2	GA binding protein transcription factor, beta subunit 2	**1.5**	**-1.7**
GABRA2	gamma-aminobutyric acid (GABA) A receptor, alpha 2		**-1.5**
GADD45A	growth arrest and DNA-damage-inducible, alpha	**15.7**	
GADD45B	growth arrest and DNA-damage-inducible, beta	**1.5**	**1.3**
GAK	cyclin G associated kinase		**1.4**
GAL	galanin		**75.8**
GALC	galactosylceramidase		**-2.1**
GALK2	galactokinase 2		**1.3**
GALNAC4S-6ST	B cell RAG associated protein	**1.6**	**-19.8**
GALNACT-2	chondroitin sulfate GalNAcT-2	**1.4**	
GALNT1	UDP-N-acetyl-alpha-D-galactosamine:polypeptide N-acetylgalactosaminyltransferase 1 (GalNAc-T1)		**2.0**
GALNT11	UDP-N-acetyl-alpha-D-galactosamine:polypeptide N-acetylgalactosaminyltransferase 11 (GalNAc-T11)		**28.8**
GALNT14	UDP-N-acetyl-alpha-D-galactosamine:polypeptide N-acetylgalactosaminyltransferase 14 (GalNAc-T14)		**2.1**
GALNT2	UDP-N-acetyl-alpha-D-galactosamine:polypeptide N-acetylgalactosaminyltransferase 2 (GalNAc-T2)	**1.4**	**-2.2**
GALNT3	UDP-N-acetyl-alpha-D-galactosamine:polypeptide N-acetylgalactosaminyltransferase 3 (GalNAc-T3)		**42.4**
GALNT6	UDP-N-acetyl-alpha-D-galactosamine:polypeptide N-acetylgalactosaminyltransferase 6 (GalNAc-T6)		**1.7**
GALNT7	UDP-N-acetyl-alpha-D-galactosamine:polypeptide N-acetylgalactosaminyltransferase 7 (GalNAc-T7)	**1.7**	**1.5**
GALNTL4	UDP-N-acetyl-alpha-D-galactosamine:polypeptide N-acetylgalactosaminyltransferase-like 4		**2.8**
GAMT	guanidinoacetate N-methyltransferase	**-2.2**	
GANAB	glucosidase, alpha; neutral AB	**-1.3**	
GANC	glucosidase, alpha; neutral C		**-2.3**
GAPD	glyceraldehyde-3-phosphate dehydrogenase		**-1.2**
GAPDHS	glyceraldehyde-3-phosphate dehydrogenase, spermatogenic		**1.8**
GARNL1	GTPase activating Rap/RanGAP domain-like 1	**1.6**	
GARS	glycyl-tRNA synthetase	**-2.1**	**1.9**
GART	phosphoribosylglycinamide formyltransferase, phosphoribosylglycinamide synthetase, phosphoribosylaminoimidazole synthetase	**-2.4**	**1.4**
GAS1	growth arrest-specific 1		**-6.0**
GAS2L1	growth arrest-specific 2 like 1	**15.6**	
GAS5	growth arrest-specific 5		**1.8**
GAS7	growth arrest-specific 7	**1.5**	**-10.7**
GATA2	GATA binding protein 2		**3.6**
GATA3	GATA binding protein 3		**-3.1**
GATAD2B	GATA zinc finger domain containing 2B	**-1.2**	**1.6**
GATM	glycine amidinotransferase (L-arginine:glycine amidinotransferase)		**-2.1**
GBA3	glucosidase, beta, acid 3 (cytosolic)	**2.2**	
GBAS	glioblastoma amplified sequence		**4.0**
GBE1	glucan (1,4-alpha-), branching enzyme 1 (glycogen branching enzyme, Andersen disease, glycogen storage disease type IV)	**1.3**	
GBGT1	globoside alpha-1,3-N-acetylgalactosaminyltransferase 1	**-1.6**	**-2.7**
GBL	G protein beta subunit-like		**-2.6**
GBP1	guanylate binding protein 1, interferon-inducible, 67kDa	**-5.8**	**-26.9**

GBP2	guanylate binding protein 2, interferon-inducible	**-2.5**	
GBP4	guanylate binding protein 4	**-1.3**	
GCA	grancalcin, EF-hand calcium binding protein		**3.5**
GCAT	glycine C-acetyltransferase (2-amino-3-ketobutyrate coenzyme A ligase)	**-1.4**	**9.1**
GCET2	germinal center expressed transcript 2	**3.5**	**-4.5**
GCH1	GTP cyclohydrolase 1 (dopa-responsive dystonia)	**-1.7**	**-1.7**
GCHFR	GTP cyclohydrolase I feedback regulator	**-1.8**	
GCL	germ cell-less homolog 1 (Drosophila)	**-2.1**	
GCLC	glutamate-cysteine ligase, catalytic subunit	**1.7**	**2.0**
GCLM	glutamate-cysteine ligase, modifier subunit	**2.4**	**-2.5**
GCN1L1	GCN1 general control of amino-acid synthesis 1-like 1 (yeast)	**-1.4**	**1.3**
GCN5L2	GCN5 general control of amino-acid synthesis 5-like 2 (yeast)	**-1.3**	
GCNT2	glucosaminyl (N-acetyl) transferase 2, I-branching enzyme (I blood group)	**2.2**	
GCS1	glucosidase I	**-1.3**	
GCSH	glycine cleavage system protein H (aminomethyl carrier)	**-3.0**	**2.1**
GDA	guanine deaminase	**-1.5**	
GDF11	growth differentiation factor 11		**-2.6**
GDI1	GDP dissociation inhibitor 1	**1.4**	**-1.6**
GDI2	GDP dissociation inhibitor 2	**1.4**	**1.7**
GEMIN4	gem (nuclear organelle) associated protein 4		**-1.6**
GEMIN6	gem (nuclear organelle) associated protein 6	**-1.9**	**-1.5**
GEMIN7	gem (nuclear organelle) associated protein 7	**-1.6**	**-1.2**
GFI1	growth factor independent 1		**65.2**
GFPT1	glutamine-fructose-6-phosphate transaminase 1		**-2.1**
GGA1	golgi associated, gamma adaptin ear containing, ARF binding protein 1	**-1.6**	**1.2**
GGA2	golgi associated, gamma adaptin ear containing, ARF binding protein 2	**-1.8**	**1.6**
GGA3	golgi associated, gamma adaptin ear containing, ARF binding protein 3		**2.0**
GGCX	gamma-glutamyl carboxylase		**1.9**
GGH	gamma-glutamyl hydrolase (conjugase, folylpolygammaglutamyl hydrolase)	**1.2**	**-2.7**
GGPS1	geranylgeranyl diphosphate synthase 1	**1.2**	**-1.5**
GGTA1	glycoprotein, alpha-galactosyltransferase 1		**3.5**
GHR	growth hormone receptor	**8.0**	**2.1**
GIT1	G protein-coupled receptor kinase interactor 1		**-1.4**
GIT2	G protein-coupled receptor kinase interactor 2		**12.8**
GJA7	gap junction protein, alpha 7, 45kDa (connexin 45)	**1.6**	**-13.5**
GK2	glycerol kinase 2	**2.1**	
GKAP1	G kinase anchoring protein 1		**-3.0**
GLA	galactosidase, alpha		**2.3**
GLB1	galactosidase, beta 1		**2.3**
GLB1L3	galactosidase, beta 1 like 3	**1.8**	
GLCCI1	glucocorticoid induced transcript 1		**1.7**
GLDC	glycine dehydrogenase (decarboxylating)	**-2.6**	**-11.6**

GLE1L	GLE1 RNA export mediator-like (yeast)	**-1.2**	
GLIPR1	GLI pathogenesis-related 1 (glioma)		**1.5**
GLMN	glomulin, FKBP associated protein		**3.5**
GLO1	glyoxalase I	**-1.5**	**1.3**
GLRX	glutaredoxin (thioltransferase)	**1.6**	**-4.0**
GLRX2	glutaredoxin 2	**-1.3**	**-1.5**
GLS	glutaminase	**-2.1**	**2.1**
GLTSCR2	glioma tumor suppressor candidate region gene 2	**1.5**	**-1.3**
GLUD1	glutamate dehydrogenase 1	**-1.3**	
GLUL	glutamate-ammonia ligase (glutamine synthetase)	**2.5**	**2.3**
GMFG	glia maturation factor, gamma	**1.7**	
GMNN	geminin, DNA replication inhibitor	**-1.6**	
GMPS	guanine monphosphate synthetase	**-1.4**	**-1.4**
GNA11	guanine nucleotide binding protein (G protein), alpha 11 (Gq class)		**1.6**
GNA12	guanine nucleotide binding protein (G protein) alpha 12	**2.9**	**-1.5**
GNA13	guanine nucleotide binding protein (G protein), alpha 13		**1.6**
GNA15	guanine nucleotide binding protein (G protein), alpha 15 (Gq class)	**1.4**	**-3.2**
GNAI1	guanine nucleotide binding protein (G protein), alpha inhibiting activity polypeptide 1	**1.7**	**5.9**
GNAI3	guanine nucleotide binding protein (G protein), alpha inhibiting activity polypeptide 3	**2.0**	
GNAQ	guanine nucleotide binding protein (G protein), q polypeptide	**1.3**	**5.0**
GNAS	GNAS complex locus	**1.7**	**-1.7**
GNAZ	guanine nucleotide binding protein (G protein), alpha z polypeptide		**-8.3**
GNB1	guanine nucleotide binding protein (G protein), beta polypeptide 1		**-1.4**
GNB2	guanine nucleotide binding protein (G protein), beta polypeptide 2	**1.4**	**-1.7**
GNB2L1	guanine nucleotide binding protein (G protein), beta polypeptide 2-like 1	**-1.6**	
GNB4	guanine nucleotide binding protein (G protein), beta polypeptide 4	**1.2**	
GNB5	guanine nucleotide binding protein (G protein), beta 5	**-1.3**	**-2.8**
GNE	glucosamine (UDP-N-acetyl)-2-epimerase/N-acetylmannosamine kinase		**-3.9**
GNG11	guanine nucleotide binding protein (G protein), gamma 11	**4.8**	
GNG12	guanine nucleotide binding protein (G protein), gamma 12	**1.6**	**1.8**
GNG2	guanine nucleotide binding protein (G protein), gamma 2	**2.1**	**22.0**
GNG7	guanine nucleotide binding protein (G protein), gamma 7	**1.7**	**-1.4**
GNL1	guanine nucleotide binding protein-like 1		**-1.3**
GNL2	guanine nucleotide binding protein-like 2 (nucleolar)	**-1.4**	
GNL3	guanine nucleotide binding protein-like 3 (nucleolar)	**-2.4**	
GNMT	glycine N-methyltransferase	**-1.6**	**1.4**
GNPDA1	glucosamine-6-phosphate deaminase 1		**11.5**
GNPNAT1	glucosamine-phosphate N-acetyltransferase 1	**-1.5**	**-1.4**
GNS	glucosamine (N-acetyl)-6-sulfatase (Sanfilippo disease IIID)		**2.8**
GOLGA2	golgi autoantigen, golgin subfamily a, 2		**1.7**
GOLGA5	golgi autoantigen, golgin subfamily a, 5	**1.2**	**1.5**
GOLGA7	golgi autoantigen, golgin subfamily a, 7		**1.7**

GOLGB1	golgi autoantigen, golgin subfamily b, macrogolgin (with transmembrane signal), 1		**4.9**
GOPC	golgi associated PDZ and coiled-coil motif containing		**1.3**
GORASP1	golgi reassembly stacking protein 1, 65kDa		**1.8**
GORASP2	golgi reassembly stacking protein 2, 55kDa	**-1.3**	**1.8**
GOSR1	golgi SNAP receptor complex member 1		**1.5**
GP9	glycoprotein IX (platelet)	**9.6**	
GPAM	glycerol-3-phosphate acyltransferase, mitochondrial	**-2.4**	**2.1**
GPBP1	GC-rich promoter binding protein 1	**1.2**	
GPC1	glypican 1	**1.4**	
GPD1L	glycerol-3-phosphate dehydrogenase 1-like	**-2.1**	**1.5**
GPD2	glycerol-3-phosphate dehydrogenase 2 (mitochondrial)		**-1.3**
GPHN	gephyrin		**7.7**
GPI	glucose phosphate isomerase	**-3.3**	
GPLD1	glycosylphosphatidylinositol specific phospholipase D1	**-1.7**	**-2.3**
GPM6B	glycoprotein M6B	**1.8**	**-174.8**
GPR	G protein-coupled receptor 176	**-1.3**	**-18.0**
GPR160	G protein-coupled receptor 160		**1.8**
GPR51	gamma-aminobutyric acid (GABA) B receptor, 2	**-2.4**	**-5.3**
GPR56	G protein-coupled receptor 56	**25.3**	
GPRASP1	G protein-coupled receptor associated sorting protein 1		**11.0**
GPS2	G protein pathway suppressor 2		**-4.7**
GPSM1	G-protein signalling modulator 1 (AGS3-like, C. elegans)		**-3.7**
GPSM2	G-protein signalling modulator 2 (AGS3-like, C. elegans)	**1.6**	**-1.8**
GPSM3	G-protein signalling modulator 3 (AGS3-like, C. elegans)		**-1.9**
GPT2	glutamic pyruvate transaminase (alanine aminotransferase) 2	**-2.0**	**-4.4**
GPX1	glutathione peroxidase 1	**1.7**	**1.7**
GPX4	glutathione peroxidase 4 (phospholipid hydroperoxidase)		**1.8**
GPX7	glutathione peroxidase 7		**1.9**
GRAP	GRB2-related adaptor protein	**3.2**	**-2.0**
GRB10	growth factor receptor-bound protein 10	**-1.4**	**-12.4**
GRB2	growth factor receptor-bound protein 2	**-1.8**	**1.9**
GREB1	GREB1 protein		**8.1**
GREM1	gremlin 1, cysteine knot superfamily, homolog (Xenopus laevis)		**-42.1**
GRHPR	glyoxylate reductase/hydroxypyruvate reductase	**-1.6**	**1.4**
GRIK5	glutamate receptor, ionotropic, kainate 5		**4.2**
GRIN2B	glutamate receptor, ionotropic, N-methyl D-aspartate 2B		**-2.6**
GRINA	glutamate receptor, ionotropic, N-methyl D-asparate-associated protein 1 (glutamate binding)	**-1.8**	
GRK5	G protein-coupled receptor kinase 5	**2.8**	**14.8**
GRK6	G protein-coupled receptor kinase 6		**-3.3**
GRM3	glutamate receptor, metabotropic 3	**-1.7**	
GRN	granulin		**1.4**
GSK3B	glycogen synthase kinase 3 beta	**1.3**	**2.1**

GSN	gelsolin (amyloidosis, Finnish type)	**5.4**	**7.8**
GSPT1	G1 to S phase transition 1	**-1.2**	**-1.6**
GSPT2	G1 to S phase transition 2		**14.3**
GSR	glutathione reductase		**-1.6**
GSS	glutathione synthetase	**-1.3**	
GSTK1	glutathione S-transferase kappa 1		**1.8**
GSTM1	glutathione S-transferase M1	**-1.7**	**-1.6**
GSTM2	glutathione S-transferase M2 (muscle)	**-2.2**	**-1.2**
GSTM3	glutathione S-transferase M3 (brain)		**69.2**
GSTM4	glutathione S-transferase M4	**-1.7**	
GSTO1	glutathione S-transferase omega 1	**-1.3**	**1.8**
GSTO2	glutathione S-transferase omega 2		**4.3**
GSTP1	glutathione S-transferase pi		**-1.2**
GTF2A1	general transcription factor IIA, 1, 19/37kDa		**-1.6**
GTF2A2	general transcription factor IIA, 2, 12kDa	**-1.2**	**-2.8**
GTF2B	general transcription factor IIB		**-1.2**
GTF2E2	general transcription factor IIE, polypeptide 2, beta 34kDa	**-1.3**	**-1.5**
GTF2F1	general transcription factor IIF, polypeptide 1, 74kDa	**-1.7**	
GTF2H1	general transcription factor IIH, polypeptide 1, 62kDa	**-1.3**	**1.3**
GTF2H2	general transcription factor IIH, polypeptide 2, 44kDa		**2.1**
GTF2H3	general transcription factor IIH, polypeptide 3, 34kDa	**-1.4**	
GTF2H4	general transcription factor IIH, polypeptide 4, 52kDa		**1.7**
GTF2I	general transcription factor II, i		**-2.1**
GTF2IRD1	GTF2I repeat domain containing 1		**1.2**
GTF3A	general transcription factor IIIA	**-1.4**	**-1.7**
GTF3C1	general transcription factor IIIC, polypeptide 1, alpha 220kDa		**-1.3**
GTF3C2	general transcription factor IIIC, polypeptide 2, beta 110kDa	**-2.0**	
GTF3C3	general transcription factor IIIC, polypeptide 3, 102kDa		**2.3**
GTF3C4	general transcription factor IIIC, polypeptide 4, 90kDa	**-1.6**	
GTF3C5	general transcription factor IIIC, polypeptide 5, 63kDa		**1.5**
GTL3	chromosome 16 open reading frame 80	**-1.4**	**-1.3**
GTPBP4	GTP binding protein 4	**-1.7**	**3.0**
GTPBP6	GTP binding protein 6 (putative)		**-2.2**
GTSE1	G-2 and S-phase expressed 1	**1.6**	**-1.7**
GUCY1A3	guanylate cyclase 1, soluble, alpha 3		**-1.2**
GUCY1B3	guanylate cyclase 1, soluble, beta 3	**-2.2**	**1.5**
GUSB	glucuronidase, beta		**1.2**
GVIN1	GTPase, very large interferon inducible 1	**1.4**	**-2.0**
GYPC	glycophorin C (Gerbich blood group)		**1.8**
GYS1	glycogen synthase 1 (muscle)	**-1.3**	**1.5**
H1F0	H1 histone family, member 0	**2.4**	**8.1**
H1FX	H1 histone family, member X		**-3.2**

H2AFX	H2A histone family, member X		**-2.6**
H2AFY	H2A histone family, member Y	**-1.6**	**-1.9**
H3F3A	H3 histone, family 3A		**-1.2**
H3F3B	H3 histone, family 3B (H3.3B)	**1.3**	**4.1**
H41	CDV3 homolog (mouse)	**-1.2**	**1.3**
H6PD	hexose-6-phosphate dehydrogenase (glucose 1-dehydrogenase)		**-2.4**
HABP2	hyaluronan binding protein 2	**-1.3**	
HADH2	hydroxysteroid (17-beta) dehydrogenase 10		**-1.2**
HADHA	hydroxyacyl-Coenzyme A dehydrogenase/3-ketoacyl-Coenzyme A thiolase/enoyl-Coenzyme A hydratase (trifunctional protein), alpha subunit	**1.5**	
HADHB	hydroxyacyl-Coenzyme A dehydrogenase/3-ketoacyl-Coenzyme A thiolase/enoyl-Coenzyme A hydratase (trifunctional protein), beta subunit		**1.8**
HADHSC	--	**-1.7**	
HAN11	WD repeat domain 68	**-1.2**	**1.8**
HAT1	histone acetyltransferase 1		**1.6**
HAX1	HCLS1 associated protein X-1	**-1.7**	**-1.3**
HBP1	HMG-box transcription factor 1	**1.4**	**1.6**
HBXIP	hepatitis B virus x interacting protein	**1.2**	
HCAP-D3	non-SMC condensin II complex, subunit D3	**-2.1**	
HCAP-G	--	**1.7**	
HCCA2	HCCA2 protein	**1.3**	
HCFC1	host cell factor C1 (VP16-accessory protein)		**-1.5**
HCFC1R1	host cell factor C1 regulator 1 (XPO1 dependent)		**-1.7**
HCLS1	hematopoietic cell-specific Lyn substrate 1	**-1.4**	**-1.6**
HCMOGT-1	sperm antigen with calponin homology and coiled-coil domains 1		**-1.6**
HDAC2	histone deacetylase 2	**-1.5**	
HDAC3	histone deacetylase 3		**1.5**
HDAC4	histone deacetylase 4	**-1.8**	**2.6**
HDAC9	histone deacetylase 9		**-4.3**
HDGF	hepatoma-derived growth factor (high-mobility group protein 1-like)	**-1.5**	
HELLS	helicase, lymphoid-specific	**-3.8**	**-1.6**
HELZ	helicase with zinc finger		**1.5**
HERC1	hect (homologous to the E6-AP (UBE3A) carboxyl terminus) domain and RCC1 (CHC1)-like domain (RLD) 1	**1.7**	**1.6**
HERPUD1	homocysteine-inducible, endoplasmic reticulum stress-inducible, ubiquitin-like domain member 1		**-2.1**
HES6	hairy and enhancer of split 6 (Drosophila)		**-7.5**
HEXA	hexosaminidase A (alpha polypeptide)		**1.7**
HEXIM1	hexamethylene bis-acetamide inducible 1	**1.8**	**4.5**
HGS	hepatocyte growth factor-regulated tyrosine kinase substrate		**1.5**
HHEX	homeobox, hematopoietically expressed	**-2.0**	**3.4**
HIBADH	3-hydroxyisobutyrate dehydrogenase		**3.0**
HIF1A	hypoxia-inducible factor 1, alpha subunit (basic helix-loop-helix transcription factor)	**1.2**	
HIF1AN	hypoxia-inducible factor 1, alpha subunit inhibitor	**1.3**	
HIG1	HIG1 domain family, member 1A	**1.2**	
HIG2	hypoxia-inducible protein 2	**-2.2**	**-1.3**

HINT1	histidine triad nucleotide binding protein 1	**1.4**	**1.3**
HIP1	huntingtin interacting protein 1	**1.8**	
HIP1R	huntingtin interacting protein 1 related	**2.3**	**-1.5**
HIP2	huntingtin interacting protein 2	**-1.2**	**-1.6**
HIPK1	homeodomain interacting protein kinase 1	**-1.5**	
HIPK2	homeodomain interacting protein kinase 2	**1.4**	**1.3**
HIPK3	homeodomain interacting protein kinase 3	**1.5**	**1.3**
HISPPD2A	histidine acid phosphatase domain containing 2A		**-3.1**
HIST1H1C	histone cluster 1, H1c	**2.6**	**-3.9**
HIST2H2AA	histone cluster 2, H2aa3		**-2.0**
HIVEP1	human immunodeficiency virus type I enhancer binding protein 1	**2.4**	**1.4**
HIVEP2	human immunodeficiency virus type I enhancer binding protein 2		**-6.5**
HIVEP3	human immunodeficiency virus type I enhancer binding protein 3	**4.3**	
HK1	hexokinase 1		**3.4**
HK2	hexokinase 2	**-3.1**	**1.4**
HLA-A	major histocompatibility complex, class I, A		**-2.5**
HLA-B	major histocompatibility complex, class I, B	**1.2**	**-3.4**
HLA-C	major histocompatibility complex, class I, C	**1.6**	**-2.9**
HLA-DMA	major histocompatibility complex, class II, DM alpha		**-15.9**
HLA-DMB	major histocompatibility complex, class II, DM beta		**-1.7**
HLA-DPA1	major histocompatibility complex, class II, DP alpha 1	**1.2**	**-20.4**
HLA-DPB1	major histocompatibility complex, class II, DP beta 1	**1.3**	**-17.6**
HLA-DQA1	major histocompatibility complex, class II, DQ alpha 1		**-3.3**
HLA-DQB1	major histocompatibility complex, class II, DQ beta 1	**1.9**	**-3.3**
HLA-DRA	major histocompatibility complex, class II, DR alpha	**1.3**	**-212.7**
HLA-DRB1	major histocompatibility complex, class II, DR beta 1		**-54.1**
HLA-DRB5	major histocompatibility complex, class II, DR beta 5	**1.2**	**-25.1**
HLA-E	major histocompatibility complex, class I, E	**2.6**	**-1.3**
HLA-F	major histocompatibility complex, class I, F	**2.2**	**-3.0**
HLA-G	HLA-G histocompatibility antigen, class I, G	**1.5**	**-2.0**
HLCS	holocarboxylase synthetase (biotin-(proprionyl-Coenzyme A-carboxylase (ATP-hydrolysing)) ligase)		**2.9**
HLX1	H2.0-like homeobox 1 (Drosophila)		**-4.3**
HMGA1	high mobility group AT-hook 1	**-1.6**	
HMGA2	high mobility group AT-hook 2	**-5.0**	**-1.5**
HMGB1	high-mobility group box 1		**-3.0**
HMGB2	high-mobility group box 2	**1.3**	
HMGB3	high-mobility group box 3	**1.4**	
HMGCR	3-hydroxy-3-methylglutaryl-Coenzyme A reductase		**-1.9**
HMGCS1	3-hydroxy-3-methylglutaryl-Coenzyme A synthase 1 (soluble)		**-3.5**
HMMR	hyaluronan-mediated motility receptor (RHAMM)	**1.8**	**-1.5**
HN1	hematological and neurological expressed 1		**2.0**
HNRPA1	heterogeneous nuclear ribonucleoprotein A1	**1.4**	**1.9**

HNRPA2B1	heterogeneous nuclear ribonucleoprotein A2/B1		5.0
HNRPA3	heterogeneous nuclear ribonucleoprotein A3	-1.4	1.8
HNRPAB	heterogeneous nuclear ribonucleoprotein A/B	-1.6	-1.4
HNRPC	heterogeneous nuclear ribonucleoprotein C (C1/C2)	-1.5	-1.2
HNRPD	heterogeneous nuclear ribonucleoprotein D (AU-rich element RNA binding protein 1, 37kDa)		3.1
HNRPDL	heterogeneous nuclear ribonucleoprotein D-like	-1.6	1.7
HNRPF	heterogeneous nuclear ribonucleoprotein F		1.3
HNRPH1	heterogeneous nuclear ribonucleoprotein H1 (H)	1.3	-2.9
HNRPH2	heterogeneous nuclear ribonucleoprotein H2 (H')	1.5	
HNRPK	heterogeneous nuclear ribonucleoprotein K	-1.4	
HNRPL	heterogeneous nuclear ribonucleoprotein L	-2.0	-1.4
HNRPM	heterogeneous nuclear ribonucleoprotein M	1.8	1.8
HNRPU	heterogeneous nuclear ribonucleoprotein U (scaffold attachment factor A)	-1.4	-1.4
HNRPUL1	heterogeneous nuclear ribonucleoprotein U-like 1		-1.4
HOMER1	homer homolog 1 (Drosophila)		-2.3
HOMER2	homer homolog 2 (Drosophila)		2.4
HOMER3	homer homolog 3 (Drosophila)		10.4
HOM-TES-103	hypothetical protein LOC25900	2.3	
HOXA1	homeobox A1		-1.5
HOXA10	homeobox A10		-1.3
HOXA2	homeobox A2		2.6
HOXA7	homeobox A7		-4.8
HOXA9	homeobox A9		-91.3
HOXD13	--		6.6
HPGD	hydroxyprostaglandin dehydrogenase 15-(NAD)	2.6	
HPRP8BP	--	-1.5	-1.3
HPRT1	hypoxanthine phosphoribosyltransferase 1 (Lesch-Nyhan syndrome)	-1.6	
HPS1	Hermansky-Pudlak syndrome 1		1.2
HPS3	Hermansky-Pudlak syndrome 3		-1.2
HPS4	Hermansky-Pudlak syndrome 4	2.8	-3.7
HPSE	heparanase	-3.0	
HRAS	v-Ha-ras Harvey rat sarcoma viral oncogene homolog		-1.8
HRB	HIV-1 Rev binding protein	1.5	-1.7
HRB2	KRR1, small subunit (SSU) processome component, homolog (yeast)	-1.3	
HRH4	histamine receptor H4		-2.6
HRK	harakiri, BCL2 interacting protein (contains only BH3 domain)	3.2	
HRMT1L1	protein arginine methyltransferase 2	1.9	
HRMT1L2	protein arginine methyltransferase 1	-2.6	-1.2
HRSP12	heat-responsive protein 12	-2.0	
HS2ST1	heparan sulfate 2-O-sulfotransferase 1		1.8
HS3ST3B1	heparan sulfate (glucosamine) 3-O-sulfotransferase 3B1	3.2	1.4
HS6ST1	heparan sulfate 6-O-sulfotransferase 1	1.8	1.3

HS6ST2	heparan sulfate 6-O-sulfotransferase 2	**1.6**	
HSD17B4	hydroxysteroid (17-beta) dehydrogenase 4	**1.9**	**2.0**
HSD17B7	hydroxysteroid (17-beta) dehydrogenase 7		**-1.9**
HSF2	heat shock transcription factor 2		**1.2**
HSH2D	hematopoietic SH2 domain containing		**-6.8**
HSPA1A	heat shock 70kDa protein 1A		**6.3**
HSPA1B	heat shock 70kDa protein 1B	**-1.7**	**-4.3**
HSPA4	heat shock 70kDa protein 4	**-1.4**	
HSPA4L	heat shock 70kDa protein 4-like		**25.8**
HSPA5	heat shock 70kDa protein 5 (glucose-regulated protein, 78kDa)		**1.6**
HSPA8	heat shock 70kDa protein 8	**-1.3**	
HSPA9B	heat shock 70kDa protein 9 (mortalin)	**-2.8**	
HSPB1	heat shock 27kDa protein 1	**-1.3**	**-2.7**
HSPBAP1	HSPB (heat shock 27kDa) associated protein 1		**1.5**
HSPC121	protein tyrosine phosphatase-like A domain containing 1		**1.5**
HSPCA	heat shock protein 90kDa alpha (cytosolic), class A member 1		**-1.2**
HSPCB	heat shock protein 90kDa alpha (cytosolic), class B member 1	**-2.0**	**1.4**
HSPD1	heat shock 60kDa protein 1 (chaperonin)	**-3.0**	
HSPE1	heat shock 10kDa protein 1 (chaperonin 10)	**-2.2**	
HSPG2	heparan sulfate proteoglycan 2 (perlecan)		**4.9**
HSPH1	heat shock 105kDa/110kDa protein 1	**-3.7**	**-2.8**
HTATIP	HIV-1 Tat interacting protein, 60kDa		**-1.5**
HTATIP2	HIV-1 Tat interactive protein 2, 30kDa	**-1.3**	**-1.7**
HTATSF1	HIV-1 Tat specific factor 1		**1.4**
HTR1F	5-hydroxytryptamine (serotonin) receptor 1F		**-2.6**
HTR2C	5-hydroxytryptamine (serotonin) receptor 2C	**-2.1**	
HTR7	5-hydroxytryptamine (serotonin) receptor 7 (adenylate cyclase-coupled)		**-1.4**
HUS1	HUS1 checkpoint homolog (S. pombe)		**2.4**
HUWE1	HECT, UBA and WWE domain containing 1		**1.4**
HYI	hydroxypyruvate isomerase homolog (E. coli)	**1.7**	**-1.3**
HYOU1	hypoxia up-regulated 1	**-1.7**	**1.8**
HYPB	SET domain containing 2	**1.3**	
IARS	isoleucine-tRNA synthetase	**-2.2**	**-1.4**
IARS2	isoleucine-tRNA synthetase 2, mitochondrial	**-1.2**	**1.4**
IBRDC3	IBR domain containing 3		**-2.0**
IBTK	inhibitor of Bruton agammaglobulinemia tyrosine kinase	**1.6**	**1.5**
ICAM2	intercellular adhesion molecule 2	**-2.4**	**-17.5**
ICAM4	intercellular adhesion molecule 4 (Landsteiner-Wiener blood group)		**5.2**
ICK	intestinal cell (MAK-like) kinase	**1.5**	**-2.3**
ICMT	isoprenylcysteine carboxyl methyltransferase		**1.4**
ID2	inhibitor of DNA binding 2, dominant negative helix-loop-helix protein	**3.0**	**5.8**
ID3	inhibitor of DNA binding 3, dominant negative helix-loop-helix protein	**-1.9**	**-33.8**

IDE	insulin-degrading enzyme		**1.4**
IDH1	isocitrate dehydrogenase 1 (NADP+), soluble	**-1.6**	**-1.3**
IDH2	isocitrate dehydrogenase 2 (NADP+), mitochondrial	**-1.4**	**-1.4**
IDH3A	isocitrate dehydrogenase 3 (NAD+) alpha	**-1.7**	**-1.6**
IDH3B	isocitrate dehydrogenase 3 (NAD+) beta		**1.5**
IDI1	isopentenyl-diphosphate delta isomerase 1	**1.7**	**1.5**
IDI2	isopentenyl-diphosphate delta isomerase 2		**2.5**
IDS	iduronate 2-sulfatase (Hunter syndrome)		**-1.6**
IER2	immediate early response 2		**1.6**
IER5	immediate early response 5	**2.4**	
IER5L	immediate early response 5-like		**22.2**
IFI16	interferon, gamma-inducible protein 16	**2.4**	**2.0**
IFI30	interferon, gamma-inducible protein 30		**-6.4**
IFIT2	interferon-induced protein with tetratricopeptide repeats 2		**2.0**
IFIT3	interferon-induced protein with tetratricopeptide repeats 3	**-1.9**	
IFIT5	interferon-induced protein with tetratricopeptide repeats 5	**-1.6**	**-55.1**
IFITM1	interferon induced transmembrane protein 1 (9-27)	**3.2**	**4.6**
IFITM2	interferon induced transmembrane protein 2 (1-8D)	**2.2**	**-3.1**
IFITM3	interferon induced transmembrane protein 3 (1-8U)	**1.8**	
IFNAR1	interferon (alpha, beta and omega) receptor 1		**-1.4**
IFNAR2	interferon (alpha, beta and omega) receptor 2		**-2.9**
IFNGR1	interferon gamma receptor 1	**3.3**	**5.7**
IFRD2	interferon-related developmental regulator 2	**-3.4**	
IFT20	intraflagellar transport 20 homolog (Chlamydomonas)	**1.5**	
IFT52	intraflagellar transport 52 homolog (Chlamydomonas)	**-1.3**	**-1.3**
IFT88	intraflagellar transport 88 homolog (Chlamydomonas)		**-2.8**
IGF1R	--	**-1.4**	**1.4**
IGF2R	insulin-like growth factor 2 receptor	**1.7**	**1.3**
IGFBP4	insulin-like growth factor binding protein 4	**-6.0**	**16.3**
IGFBP7	insulin-like growth factor binding protein 7	**2.5**	**-2.3**
IGHG1	immunoglobulin heavy constant gamma 1 (G1m marker)	**1.3**	**-2.6**
IGHM	immunoglobulin heavy constant mu	**1.4**	**-39.4**
IGJ	immunoglobulin J polypeptide, linker protein for immunoglobulin alpha and mu polypeptides	**-7.6**	
IGLC1	immunoglobulin lambda constant 1 (Mcg marker)		**-10.6**
IGLL1	immunoglobulin lambda-like polypeptide 1	**-1.2**	**-44.6**
IGSF8	immunoglobulin superfamily, member 8		**-1.6**
IHPK1	inositol hexaphosphate kinase 1	**-1.4**	**1.8**
IHPK2	inositol hexaphosphate kinase 2		**1.4**
IKBKG	inhibitor of kappa light polypeptide gene enhancer in B-cells, kinase gamma		**-1.5**
IL13RA1	interleukin 13 receptor, alpha 1		**10.5**
IL15	interleukin 15	**-1.3**	
IL18	interleukin 18 (interferon-gamma-inducing factor)		**6.9**

IL18BP	interleukin 18 binding protein		**2.7**
IL18R1	interleukin 18 receptor 1		**11.1**
IL1B	interleukin 1, beta	**-3.3**	**-5.2**
IL1RAP	interleukin 1 receptor accessory protein	**9.6**	**7.6**
IL21R	interleukin 21 receptor	**-2.4**	**-31.8**
IL27RA	interleukin 27 receptor, alpha		**20.9**
IL28A	interleukin 28A (interferon, lambda 2)		**2.3**
IL28RA	interleukin 28 receptor, alpha (interferon, lambda receptor)	**2.8**	**-20.1**
IL2RG	interleukin 2 receptor, gamma (severe combined immunodeficiency)	**-1.4**	**-1.4**
IL6ST	interleukin 6 signal transducer (gp130, oncostatin M receptor)	**4.7**	**4.6**
IL7R	interleukin 7 receptor	**-3.1**	**-56.6**
IL8	interleukin 8		**7.7**
ILF3	interleukin enhancer binding factor 3, 90kDa	**-2.2**	**1.7**
ILK	integrin-linked kinase		**1.4**
ILKAP	integrin-linked kinase-associated serine/threonine phosphatase 2C		**-2.2**
IMMT	inner membrane protein, mitochondrial (mitofilin)		**1.4**
IMP-3	insulin-like growth factor 2 mRNA binding protein 3	**2.0**	**1.9**
IMPA1	inositol(myo)-1(or 4)-monophosphatase 1	**1.2**	**-1.7**
IMPA2	inositol(myo)-1(or 4)-monophosphatase 2	**-12.4**	**1.8**
IMPACT	Impact homolog (mouse)		**25.9**
IMPDH1	IMP (inosine monophosphate) dehydrogenase 1		**-2.0**
IMPDH2	IMP (inosine monophosphate) dehydrogenase 2	**-1.4**	**1.3**
ING2	inhibitor of growth family, member 2	**-2.4**	
ING3	inhibitor of growth family, member 3	**1.3**	**-1.9**
ING5	inhibitor of growth family, member 5		**-1.6**
INHBC	inhibin, beta C		**-1.3**
INPP1	inositol polyphosphate-1-phosphatase	**2.7**	**1.4**
INPP4B	inositol polyphosphate-4-phosphatase, type II, 105kDa	**1.8**	**6.6**
INPP5D	inositol polyphosphate-5-phosphatase, 145kDa	**2.0**	**-1.6**
INPP5F	inositol polyphosphate-5-phosphatase F	**2.1**	
INSIG1	insulin induced gene 1	**1.7**	**-5.5**
INSM1	insulinoma-associated 1		**-6.5**
INSR	insulin receptor	**2.9**	**1.4**
INVS	inversin		**1.9**
IPO11	--	**1.9**	
IPO13	importin 13	**-1.4**	**1.4**
IPO4	importin 4	**-2.1**	
IPO7	importin 7	**-1.7**	**1.5**
IPO9	importin 9	**-1.3**	**1.5**
IPPK	inositol 1,3,4,5,6-pentakisphosphate 2-kinase	**-1.3**	**1.3**
IQGAP1	IQ motif containing GTPase activating protein 1	**2.2**	**-2.4**
IQGAP2	IQ motif containing GTPase activating protein 2	**2.6**	

IQSEC1	IQ motif and Sec7 domain 1	**1.8**	
IRAK1	interleukin-1 receptor-associated kinase 1		**-1.3**
IRAK1BP1	interleukin-1 receptor-associated kinase 1 binding protein 1	**-3.0**	**-1.6**
IRAK3	interleukin-1 receptor-associated kinase 3	**2.9**	**3.0**
IREB2	iron-responsive element binding protein 2		**1.5**
IRF1	interferon regulatory factor 1	**-1.8**	**-2.1**
IRF2	interferon regulatory factor 2		**-2.3**
IRF3	interferon regulatory factor 3		**-1.2**
IRF4	interferon regulatory factor 4		**-4.9**
IRF5	interferon regulatory factor 5		**3.9**
IRF7	interferon regulatory factor 7	**1.6**	**-2.0**
IRF8	interferon regulatory factor 8		**-2.4**
IRS1	insulin receptor substrate 1	**1.5**	**-4.8**
IRS2	insulin receptor substrate 2	**2.6**	**1.8**
IRS4	insulin receptor substrate 4	**-1.4**	
IRX3	iroquois homeobox protein 3		**54.5**
IRX5	iroquois homeobox protein 5		**5.0**
ISG20	interferon stimulated exonuclease gene 20kDa	**91.9**	
ISYNA1	myo-inositol 1-phosphate synthase A1		**2.9**
ITCH	itchy homolog E3 ubiquitin protein ligase (mouse)	**1.8**	**2.0**
ITGA10	integrin, alpha 10	**6.2**	
ITGA5	integrin, alpha 5 (fibronectin receptor, alpha polypeptide)	**1.4**	**3.0**
ITGA6	integrin, alpha 6	**7.2**	**6.1**
ITGA9	integrin, alpha 9	**1.4**	**46.2**
ITGAE	integrin, alpha E (antigen CD103, human mucosal lymphocyte antigen 1; alpha polypeptide)	**1.5**	**-2.9**
ITGAL	integrin, alpha L (antigen CD11A (p180), lymphocyte function-associated antigen 1; alpha polypeptide)	**-1.4**	**-1.7**
ITGAV	integrin, alpha V (vitronectin receptor, alpha polypeptide, antigen CD51)	**2.3**	**6.1**
ITGB1	integrin, beta 1 (fibronectin receptor, beta polypeptide, antigen CD29 includes MDF2, MSK12)	**1.4**	**3.1**
ITGB1BP1	integrin beta 1 binding protein 1		**-1.2**
ITGB2	integrin, beta 2 (complement component 3 receptor 3 and 4 subunit)	**-5.6**	**2.6**
ITGB3BP	integrin beta 3 binding protein (beta3-endonexin)	**-1.2**	
ITM2B	integral membrane protein 2B	**2.0**	**1.7**
ITM2C	integral membrane protein 2C		**-2.0**
ITPKB	inositol 1,4,5-trisphosphate 3-kinase B		**-4.6**
ITPR1	inositol 1,4,5-triphosphate receptor, type 1	**2.4**	**3.8**
ITPR2	inositol 1,4,5-triphosphate receptor, type 2	**1.8**	**4.3**
ITSN1	intersectin 1 (SH3 domain protein)	**-1.3**	**-3.8**
ITSN2	intersectin 2	**1.4**	
IVNS1ABP	influenza virus NS1A binding protein	**-1.3**	**-1.3**
IWS1	IWS1 homolog (S. cerevisiae)	**-1.6**	
JAG1	jagged 1 (Alagille syndrome)		**4.9**
JAG2	jagged 2	**1.2**	

JAK1	Janus kinase 1 (a protein tyrosine kinase)	**1.8**	**1.9**
JAK2	Janus kinase 2 (a protein tyrosine kinase)	**2.3**	**1.6**
JAK3	Janus kinase 3 (a protein tyrosine kinase, leukocyte)	**-1.5**	
JAM2	junctional adhesion molecule 2		**-2.1**
JAM3	junctional adhesion molecule 3		**12.9**
JARID1A	jumonji, AT rich interactive domain 1A	**1.2**	**-2.3**
JARID1B	jumonji, AT rich interactive domain 1B		**37.6**
JARID2	jumonji, AT rich interactive domain 2		**-1.4**
JDP2	jun dimerization protein 2		**4.8**
JMJD1C	jumonji domain containing 1C	**1.9**	**-2.0**
JMY	junction-mediating and regulatory protein		**58.8**
JRK	jerky homolog (mouse)		**2.1**
JTV1	JTV1 gene	**-2.3**	
JUB	jub, ajuba homolog (Xenopus laevis)	**-2.0**	**-2.4**
JUN	jun oncogene	**18.9**	**42.1**
JUND	jun D proto-oncogene	**1.8**	**1.3**
JUP	junction plakoglobin	**-1.3**	**1.3**
KAB	centrosomal protein 170kDa	**1.2**	**3.8**
KALRN	kalirin, RhoGEF kinase	**9.8**	
KARS	lysyl-tRNA synthetase	**-1.6**	
KATNA1	katanin p60 (ATPase-containing) subunit A 1	**1.5**	
KCNA5	potassium voltage-gated channel, shaker-related subfamily, member 5	**5.9**	
KCNAB1	potassium voltage-gated channel, shaker-related subfamily, beta member 1		**1.8**
KCNAB2	potassium voltage-gated channel, shaker-related subfamily, beta member 2	**-1.6**	
KCNE3	potassium voltage-gated channel, Isk-related family, member 3	**1.8**	**8.5**
KCNJ12	potassium inwardly-rectifying channel, subfamily J, member 12		**14.1**
KCNJ16	potassium inwardly-rectifying channel, subfamily J, member 16	**-1.4**	
KCNK3	potassium channel, subfamily K, member 3	**3.8**	
KCNMB1	potassium large conductance calcium-activated channel, subfamily M, beta member 1		**4.7**
KCNMB2	potassium large conductance calcium-activated channel, subfamily M, beta member 2		**-1.6**
KCNMB3	potassium large conductance calcium-activated channel, subfamily M beta member 3		**-2.6**
KCNMB4	potassium large conductance calcium-activated channel, subfamily M, beta member 4	**-2.4**	
KCNN4	potassium intermediate/small conductance calcium-activated channel, subfamily N, member 4	**-13.3**	**23.1**
KCNQ1OT1	KCNQ1 overlapping transcript 1		**4.2**
KCNQ4	potassium voltage-gated channel, KQT-like subfamily, member 4		**2.8**
KCTD13	potassium channel tetramerisation domain containing 13		**-2.1**
KCTD3	potassium channel tetramerisation domain containing 3	**2.4**	
KDELR2	KDEL (Lys-Asp-Glu-Leu) endoplasmic reticulum protein retention receptor 2		**1.3**
KEAP1	kelch-like ECH-associated protein 1		**-1.4**
KHDRBS1	KH domain containing, RNA binding, signal transduction associated 1	**-1.3**	**-1.3**
KHDRBS3	KH domain containing, RNA binding, signal transduction associated 3	**2.3**	**9.0**
KHK	ketohexokinase (fructokinase)		**-4.1**

KHSRP	KH-type splicing regulatory protein (FUSE binding protein 2)	**-2.3**	
KIAA0101	KIAA0101	**-1.9**	
KIAA0133	KIAA0133	**-1.4**	**-1.5**
KIAA0143	KIAA0143 protein	**1.4**	**1.9**
KIAA0310	KIAA0310		**1.5**
KIAA0368	KIAA0368	**1.8**	**1.8**
KIAA0685	SAPS domain family, member 2	**2.1**	
KIAA0802	KIAA0802	**-1.7**	**-8.8**
KIAA0828	adenosylhomocysteinase 3		**1.9**
KIAA0992	palladin, cytoskeletal associated protein		**1.6**
KIAA1008	KIAA1008		**1.7**
KIAA1115	SAPS domain family, member 1		**1.4**
KIAA1539	KIAA1539	**1.7**	
KIAA1600	KIAA1600	**1.8**	**3.5**
KIAA1794	KIAA1794		**-1.9**
KIAA1967	--	**-1.3**	**-1.2**
KIDINS220	kinase D-interacting substance of 220 kDa	**1.4**	**1.5**
KIF11	kinesin family member 11	**1.5**	**-1.6**
KIF1B	kinesin family member 1B	**1.5**	
KIF2	kinesin heavy chain member 2A		**-1.3**
KIF20A	kinesin family member 20A	**1.6**	**-1.4**
KIF22	kinesin family member 22	**1.2**	
KIF23	kinesin family member 23	**1.9**	
KIF2C	kinesin family member 2C	**1.3**	
KIF3B	kinesin family member 3B		**1.4**
KIF4A	kinesin family member 4A	**1.4**	
KIF5B	kinesin family member 5B	**1.5**	**1.2**
KIF9	kinesin family member 9	**-2.1**	
KIFC1	kinesin family member C1		**-1.6**
KIT	v-kit Hardy-Zuckerman 4 feline sarcoma viral oncogene homolog		**528.3**
KLC2L	kinesin light chain 3	**-1.9**	**1.4**
KLF10	Kruppel-like factor 10	**2.4**	**-2.3**
KLF11	Kruppel-like factor 11		**39.1**
KLF12	Kruppel-like factor 12	**3.4**	
KLF13	Kruppel-like factor 13	**1.4**	**-1.5**
KLF2	Kruppel-like factor 2 (lung)	**47.7**	
KLF3	Kruppel-like factor 3 (basic)	**2.3**	**3.5**
KLF4	Kruppel-like factor 4 (gut)	**5.8**	
KLF6	Kruppel-like factor 6	**7.2**	**1.8**
KLF7	Kruppel-like factor 7 (ubiquitous)	**4.1**	
KLF9	Kruppel-like factor 9	**6.1**	**4.6**
KLHDC2	kelch domain containing 2		**1.5**

KLHDC3	kelch domain containing 3	**-1.5**	
KLRC4	killer cell lectin-like receptor subfamily C, member 4		**-7.7**
KLRK1	killer cell lectin-like receptor subfamily K, member 1		**-25.0**
KNS2	kinesin 2	**1.8**	
KNTC1	kinetochore associated 1		**-1.5**
KNTC2	kinetochore associated 2	**1.9**	**-1.3**
KPNA1	karyopherin alpha 1 (importin alpha 5)		**-1.2**
KPNA2	karyopherin alpha 2 (RAG cohort 1, importin alpha 1)	**1.5**	**1.3**
KPNA3	karyopherin alpha 3 (importin alpha 4)	**-1.3**	**-1.5**
KPNA4	karyopherin alpha 4 (importin alpha 3)	**2.1**	**1.6**
KPNA6	karyopherin alpha 6 (importin alpha 7)		**2.0**
KPNB1	karyopherin (importin) beta 1	**-1.6**	**1.5**
KRAS	v-Ki-ras2 Kirsten rat sarcoma viral oncogene homolog	**13.3**	**-2.1**
KRT10	keratin 10 (epidermolytic hyperkeratosis; keratosis palmaris et plantaris)	**1.2**	**-2.8**
KRT8	keratin 8		**-1.4**
KTN1	kinectin 1 (kinesin receptor)		**1.8**
KYNU	kynureninase (L-kynurenine hydrolase)		**14.0**
L2HGDH	L-2-hydroxyglutarate dehydrogenase	**2.7**	
L3MBTL2	l(3)mbt-like 2 (Drosophila)		**-1.3**
LACTB	lactamase, beta		**2.2**
LAIR1	leukocyte-associated immunoglobulin-like receptor 1	**2.7**	**6.7**
LAMA5	laminin, alpha 5		**2.7**
LAMB1	laminin, beta 1		**37.6**
LAMC1	laminin, gamma 1 (formerly LAMB2)	**1.4**	**-1.6**
LAMC3	laminin, gamma 3		**3.1**
LAMP1	lysosomal-associated membrane protein 1	**1.5**	**1.8**
LAMP2	lysosomal-associated membrane protein 2	**1.7**	**2.1**
LANCL1	LanC lantibiotic synthetase component C-like 1 (bacterial)		**-1.7**
LAP1B	torsin A interacting protein 1		**1.4**
LAP3	leucine aminopeptidase 3	**-1.8**	
LAPTM5	lysosomal associated multispanning membrane protein 5	**2.0**	**-4.3**
LARP1	La ribonucleoprotein domain family, member 1	**-2.0**	
LARS	leucyl-tRNA synthetase	**-1.9**	**1.3**
LARS2	leucyl-tRNA synthetase 2, mitochondrial	**-1.5**	**-1.2**
LASP1	LIM and SH3 protein 1		**1.6**
LATS2	LATS, large tumor suppressor, homolog 2 (Drosophila)	**1.7**	
LAX	lymphocyte transmembrane adaptor 1		**1.4**
LBR	lamin B receptor	**1.4**	
LCK	lymphocyte-specific protein tyrosine kinase		**-49.3**
LCN12	lipocalcin 12		**-1.4**
LCP1	lymphocyte cytosolic protein 1 (L-plastin)	**-2.5**	**-4.1**
LCP2	lymphocyte cytosolic protein 2 (SH2 domain containing leukocyte protein of 76kDa)	**-2.4**	**1.6**

LDB1	LIM domain binding 1	**-1.2**	**1.7**
LDB3	LIM domain binding 3	**87.6**	**-1.7**
LDHA	lactate dehydrogenase A	**-1.7**	
LDHB	lactate dehydrogenase B	**-1.6**	
LDLR	low density lipoprotein receptor (familial hypercholesterolemia)	**-1.3**	**-3.6**
LEF1	lymphoid enhancer-binding factor 1	**-1.3**	**-288.8**
LEMD3	LEM domain containing 3	**-1.3**	
LEPR	leptin receptor	**2.2**	**1.8**
LEPRE1	leucine proline-enriched proteoglycan (leprecan) 1	**-1.3**	
LETMD1	LETM1 domain containing 1		**3.1**
LGALS1	lectin, galactoside-binding, soluble, 1 (galectin 1)	**2.9**	**-22.2**
LGALS3BP	lectin, galactoside-binding, soluble, 3 binding protein	**-2.3**	**-4.3**
LGALS8	lectin, galactoside-binding, soluble, 8 (galectin 8)		**1.9**
LHX6	LIM homeobox 6		**7.5**
LIG4	ligase IV, DNA, ATP-dependent	**1.8**	**1.9**
LILRB1	leukocyte immunoglobulin-like receptor, subfamily B (with TM and ITIM domains), member 1	**2.2**	**-4.4**
LILRB2	leukocyte immunoglobulin-like receptor, subfamily B (with TM and ITIM domains), member 2	**1.7**	**-1.8**
LILRB4	leukocyte immunoglobulin-like receptor, subfamily B (with TM and ITIM domains), member 4		**-2.7**
LIMD1	LIM domains containing 1	**3.2**	**-1.4**
LIMK1	LIM domain kinase 1		**-2.0**
LIMS1	LIM and senescent cell antigen-like domains 1	**1.3**	**1.3**
LIMS2	LIM and senescent cell antigen-like domains 2		**-2.5**
LIN7A	lin-7 homolog A (C. elegans)		**2.1**
LIN7C	lin-7 homolog C (C. elegans)		**1.3**
LIPA	lipase A, lysosomal acid, cholesterol esterase (Wolman disease)	**-2.0**	**1.2**
LITAF	lipopolysaccharide-induced TNF factor		**-29.0**
LLGL1	lethal giant larvae homolog 1 (Drosophila)		**-1.9**
LMAN1	lectin, mannose-binding, 1		**1.6**
LMNB1	lamin B1		**-2.0**
LMO2	LIM domain only 2 (rhombotin-like 1)	**4.4**	**7.9**
LMO4	LIM domain only 4		**-3.8**
LNK	SH2B adaptor protein 3	**-1.2**	**1.3**
LNPEP	leucyl/cystinyl aminopeptidase		**-2.2**
LNX	ligand of numb-protein X 1	**1.3**	
LNX2	ligand of numb-protein X 2		**-1.7**
LOC126731	chromosome 1 open reading frame 96		**-1.9**
LOC129607	hypothetical protein LOC129607	**1.9**	**-1.9**
LOC221955	diacylglycerol lipase beta	**1.3**	
LOC284058	KIAA1267	**1.4**	
LOC51123	zinc finger protein 706	**2.7**	
LOC51234	transmembrane protein 85	**-1.4**	**-1.5**
LOC58486	zinc finger, BED-type containing 5		**-2.3**

LOC63929	X-prolyl aminopeptidase (aminopeptidase P) 3, putative	**-1.3**	**2.2**
LOC91661	zinc finger protein 765		**-2.9**
LOC93081	chromosome 13 open reading frame 27		**2.0**
LOXL4	lysyl oxidase-like 4	**3.0**	
LPHN1	latrophilin 1		**11.8**
LPP	LIM domain containing preferred translocation partner in lipoma	**1.4**	**-1.6**
LPXN	leupaxin	**-1.7**	**-3.2**
LRG1	leucine-rich alpha-2-glycoprotein 1		**3.4**
LRIG1	leucine-rich repeats and immunoglobulin-like domains 1	**1.5**	**-86.8**
LRMP	lymphoid-restricted membrane protein	**-1.4**	**-18.6**
LRP5	low density lipoprotein receptor-related protein 5	**1.6**	
LRP8	low density lipoprotein receptor-related protein 8, apolipoprotein e receptor	**-2.1**	**-1.6**
LRPAP1	low density lipoprotein receptor-related protein associated protein 1		**3.2**
LRPPRC	leucine-rich PPR-motif containing	**-5.1**	**1.7**
LRRC16	leucine rich repeat containing 16	**5.6**	
LRRC17	leucine rich repeat containing 17		**7.3**
LRRC47	leucine rich repeat containing 47		**1.5**
LRRC5	leucine rich repeat containing 8 family, member D		**1.6**
LRRC8C	leucine rich repeat containing 8 family, member C	**-1.6**	**3.2**
LRRFIP1	leucine rich repeat (in FLII) interacting protein 1	**2.5**	**-6.0**
LRRFIP2	leucine rich repeat (in FLII) interacting protein 2		**-2.4**
LSM1	LSM1 homolog, U6 small nuclear RNA associated (S. cerevisiae)		**2.2**
LSM10	LSM10, U7 small nuclear RNA associated	**-1.3**	
LSM4	LSM4 homolog, U6 small nuclear RNA associated (S. cerevisiae)	**-1.4**	**-1.6**
LSM5	LSM5 homolog, U6 small nuclear RNA associated (S. cerevisiae)		**2.9**
LSM6	LSM6 homolog, U6 small nuclear RNA associated (S. cerevisiae)	**-1.4**	
LSM7	LSM7 homolog, U6 small nuclear RNA associated (S. cerevisiae)	**-1.5**	**-1.5**
LSM8	LSM8 homolog, U6 small nuclear RNA associated (S. cerevisiae)		**-1.4**
LSS	lanosterol synthase (2,3-oxidosqualene-lanosterol cyclase)	**-1.2**	
LTA4H	leukotriene A4 hydrolase		**2.6**
LTB4R	leukotriene B4 receptor		**1.9**
LTBP1	latent transforming growth factor beta binding protein 1		**-1.6**
LTBP2	latent transforming growth factor beta binding protein 2		**-5.2**
LTK	leukocyte tyrosine kinase		**-2.3**
LUZP5	non-SMC condensin II complex, subunit G2		**-1.8**
LXN	latexin		**3.6**
LY6E	lymphocyte antigen 6 complex, locus E		**2.0**
LY86	lymphocyte antigen 86	**-2.9**	**-8.9**
LY9	lymphocyte antigen 9	**2.2**	**-9.6**
LY96	lymphocyte antigen 96	**3.1**	**-9.8**
LYK5	protein kinase LYK5		**1.5**
LYL1	lymphoblastic leukemia derived sequence 1		**3.5**

LYN	v-yes-1 Yamaguchi sarcoma viral related oncogene homolog	**-1.9**	**-2.5**
LYPLA1	lysophospholipase I	**1.3**	**-1.3**
LYPLA2	lysophospholipase II	**-1.5**	
M11S1	GPI-anchored membrane protein 1	**-1.3**	**-2.0**
M6PR	mannose-6-phosphate receptor (cation dependent)	**-2.3**	
MAC30	transmembrane protein 97	**-3.0**	**1.4**
MACF1	microtubule-actin crosslinking factor 1	**-1.3**	**-1.5**
MAD1L1	MAD1 mitotic arrest deficient-like 1 (yeast)	**-1.4**	**-3.8**
MAD2L1	MAD2 mitotic arrest deficient-like 1 (yeast)		**-2.0**
MAD2L2	MAD2 mitotic arrest deficient-like 2 (yeast)		**-3.3**
MAF	v-maf musculoaponeurotic fibrosarcoma oncogene homolog (avian)	**4.4**	
MAFF	v-maf musculoaponeurotic fibrosarcoma oncogene homolog F (avian)	**4.4**	**-9.3**
MAFG	v-maf musculoaponeurotic fibrosarcoma oncogene homolog G (avian)		**-2.0**
MAG	myelin associated glycoprotein	**-2.3**	**-40.4**
MAGED1	melanoma antigen family D, 1	**-2.1**	**2.4**
MAGI1	CNKSR family member 3		**41.1**
MAGI-3	membrane associated guanylate kinase, WW and PDZ domain containing 3	**1.6**	
MAGOH	mago-nashi homolog, proliferation-associated (Drosophila)		**-1.2**
MALT1	mucosa associated lymphoid tissue lymphoma translocation gene 1	**-1.7**	**-1.7**
MAML2	mastermind-like 2 (Drosophila)	**2.0**	
MAML3	mastermind-like 3 (Drosophila)		**1.9**
MAN1A1	mannosidase, alpha, class 1A, member 1	**4.4**	
MAN1A2	mannosidase, alpha, class 1A, member 2		**1.2**
MAN1B1	mannosidase, alpha, class 1B, member 1		**1.6**
MAN1C1	mannosidase, alpha, class 1C, member 1		**-1.7**
MAN2A1	mannosidase, alpha, class 2A, member 1	**1.7**	**2.5**
MAN2A2	mannosidase, alpha, class 2A, member 2	**1.3**	**8.4**
MAP1B	microtubule-associated protein 1B		**1.3**
MAP1LC3B	microtubule-associated protein 1 light chain 3 beta	**3.2**	
MAP2	microtubule-associated protein 2		**-4.1**
MAP2K1	mitogen-activated protein kinase kinase 1	**1.9**	**1.5**
MAP2K1IP1	mitogen-activated protein kinase kinase 1 interacting protein 1	**1.6**	**-1.3**
MAP2K2	mitogen-activated protein kinase kinase 2		**1.3**
MAP2K3	mitogen-activated protein kinase kinase 3		**-2.3**
MAP2K4	mitogen-activated protein kinase kinase 4		**-1.4**
MAP2K6	mitogen-activated protein kinase kinase 6		**-2.5**
MAP3K1	mitogen-activated protein kinase kinase kinase 1		**-1.3**
MAP3K11	mitogen-activated protein kinase kinase kinase 11		**1.3**
MAP3K12	mitogen-activated protein kinase kinase kinase 12		**2.1**
MAP3K2	mitogen-activated protein kinase kinase kinase 2	**1.4**	**-1.9**
MAP3K3	mitogen-activated protein kinase kinase kinase 3	**1.7**	**2.2**
MAP3K5	mitogen-activated protein kinase kinase kinase 5	**2.1**	**1.6**

MAP3K6	mitogen-activated protein kinase kinase kinase 6		**17.8**
MAP3K7	mitogen-activated protein kinase kinase kinase 7		**1.3**
MAP3K7IP2	mitogen-activated protein kinase kinase kinase 7 interacting protein 2	**1.5**	**-1.4**
MAP3K8	mitogen-activated protein kinase kinase kinase 8		**7.8**
MAP4K1	mitogen-activated protein kinase kinase kinase kinase 1	**-2.7**	**-2.6**
MAP4K4	mitogen-activated protein kinase kinase kinase kinase 4	**1.7**	**-1.5**
MAP4K5	mitogen-activated protein kinase kinase kinase kinase 5	**2.2**	**1.9**
MAPK1	mitogen-activated protein kinase 1	**2.3**	**-1.4**
MAPK13	mitogen-activated protein kinase 13		**-3.3**
MAPK14	mitogen-activated protein kinase 14		**4.1**
MAPK8	mitogen-activated protein kinase 8		**-1.6**
MAPKAP1	mitogen-activated protein kinase associated protein 1		**-1.4**
MAPKAPK2	mitogen-activated protein kinase-activated protein kinase 2	**1.5**	
MAPKAPK3	mitogen-activated protein kinase-activated protein kinase 3	**-1.5**	**10.5**
MAPKAPK5	mitogen-activated protein kinase-activated protein kinase 5		**-1.6**
MAPRE1	microtubule-associated protein, RP/EB family, member 1	**1.5**	**-1.4**
MAPRE2	microtubule-associated protein, RP/EB family, member 2	**-2.5**	**-3.4**
MARCH2	membrane-associated ring finger (C3HC4) 2	**2.3**	
MARCH8	membrane-associated ring finger (C3HC4) 8		**1.4**
MARCKS	myristoylated alanine-rich protein kinase C substrate	**-2.5**	
MARCKSL1	MARCKS-like 1	**-1.4**	**-1.6**
MARK1	MAP/microtubule affinity-regulating kinase 1		**-16.2**
MARK3	MAP/microtubule affinity-regulating kinase 3		**1.5**
MARS	methionine-tRNA synthetase	**-2.2**	**-1.5**
MARS2	methionine-tRNA synthetase 2 (mitochondrial)	**-1.6**	
MASK	serine/threonine protein kinase MST4		**1.7**
MASP2	mannan-binding lectin serine peptidase 2	**1.2**	**1.5**
MAT2B	methionine adenosyltransferase II, beta		**-1.4**
MATK	megakaryocyte-associated tyrosine kinase		**6.9**
MATN2	matrilin 2		**3.0**
MATR3	matrin 3	**-14.6**	
MAX	MYC associated factor X	**-1.7**	**-1.2**
MAZ	MYC-associated zinc finger protein (purine-binding transcription factor)	**-1.7**	**-3.2**
MBD1	methyl-CpG binding domain protein 1		**1.3**
MBD2	methyl-CpG binding domain protein 2		**-1.6**
MBD3	methyl-CpG binding domain protein 3	**-1.4**	**-1.6**
MBD4	methyl-CpG binding domain protein 4		**1.7**
MBIP	MAP3K12 binding inhibitory protein 1		**1.2**
MBNL1	muscleblind-like (Drosophila)	**2.0**	**-4.4**
MBP	myelin basic protein	**-1.7**	**-3.1**
MCAM	melanoma cell adhesion molecule	**2.4**	**-2.6**
MCCC1	methylcrotonoyl-Coenzyme A carboxylase 1 (alpha)	**-1.4**	

MCCC2	methylcrotonoyl-Coenzyme A carboxylase 2 (beta)	**-1.5**	**1.4**
MCF2L	MCF.2 cell line derived transforming sequence-like	**2.5**	
MCFD2	multiple coagulation factor deficiency 2		**1.6**
MCL1	myeloid cell leukemia sequence 1 (BCL2-related)	**1.7**	**1.4**
MCM10	MCM10 minichromosome maintenance deficient 10 (S. cerevisiae)	**-2.3**	
MCM2	MCM2 minichromosome maintenance deficient 2, mitotin (S. cerevisiae)	**-3.1**	
MCM3	MCM3 minichromosome maintenance deficient 3 (S. cerevisiae)	**-3.1**	**-1.4**
MCM4	MCM4 minichromosome maintenance deficient 4 (S. cerevisiae)	**-3.3**	**-2.1**
MCM5	MCM5 minichromosome maintenance deficient 5, cell division cycle 46 (S. cerevisiae)	**-1.5**	**-1.5**
MCM6	minichromosome maintenance deficient 6 homolog (S. cerevisiae)	**-2.1**	**1.3**
MCM7	MCM7 minichromosome maintenance deficient 7 (S. cerevisiae)	**-1.3**	**-1.9**
MCM8	MCM8 minichromosome maintenance deficient 8 (S. cerevisiae)	**-1.3**	**-1.5**
MCP	CD46 molecule, complement regulatory protein	**1.4**	**1.6**
MCRS1	microspherule protein 1		**-1.7**
MCTS1	malignant T cell amplified sequence 1	**-1.2**	**-2.3**
MDC1	mediator of DNA damage checkpoint 1		**-4.7**
MDFI	MyoD family inhibitor	**-3.0**	**2.5**
MDFIC	MyoD family inhibitor domain containing		**-3.1**
MDH1	malate dehydrogenase 1, NAD (soluble)	**-1.3**	**1.9**
MDH2	malate dehydrogenase 2, NAD (mitochondrial)	**-1.5**	**-2.3**
MDK	midkine (neurite growth-promoting factor 2)		**1.5**
MDM2	Mdm2, transformed 3T3 cell double minute 2, p53 binding protein (mouse)	**1.8**	**-2.3**
MDM4	Mdm4, transformed 3T3 cell double minute 4, p53 binding protein (mouse)	**2.2**	**-2.4**
ME2	malic enzyme 2, NAD(+)-dependent, mitochondrial	**-1.6**	
ME3	malic enzyme 3, NADP(+)-dependent, mitochondrial		**-6.2**
MED12	mediator of RNA polymerase II transcription, subunit 12 homolog (S. cerevisiae)		**1.4**
MED28	mediator of RNA polymerase II transcription, subunit 28 homolog (S. cerevisiae)		**1.8**
MED31	mediator of RNA polymerase II transcription, subunit 31 homolog (S. cerevisiae)		**-2.4**
MED4	mediator of RNA polymerase II transcription, subunit 4 homolog (S. cerevisiae)		**-1.6**
MED6	mediator of RNA polymerase II transcription, subunit 6 homolog (S. cerevisiae)		**-1.4**
MED8	mediator of RNA polymerase II transcription, subunit 8 homolog (S. cerevisiae)		**1.7**
MEF2A	MADS box transcription enhancer factor 2, polypeptide A (myocyte enhancer factor 2A)	**1.7**	**-3.6**
MEF2C	MADS box transcription enhancer factor 2, polypeptide C (myocyte enhancer factor 2C)	**-1.5**	**-3.5**
MEF2D	MADS box transcription enhancer factor 2, polypeptide D (myocyte enhancer factor 2D)		**-5.7**
MEGF11	multiple EGF-like-domains 11	**-2.5**	
MEIS1	Meis1, myeloid ecotropic viral integration site 1 homolog (mouse)		**-43.1**
MEIS2	Meis1, myeloid ecotropic viral integration site 1 homolog 2 (mouse)	**-2.1**	**3.6**
MELK	maternal embryonic leucine zipper kinase	**1.4**	**-1.3**
MEN1	multiple endocrine neoplasia I	**-1.5**	**1.3**
MEP50	WD repeat domain 77	**-1.5**	**-1.8**
MEST	mesoderm specific transcript homolog (mouse)		**2.0**
METAP2	--	**-1.3**	**1.4**

METTL2	methyltransferase like 2B	**-1.3**	**1.5**
MFAP1	microfibrillar-associated protein 1	**-1.4**	**-1.6**
MFHAS1	malignant fibrous histiocytoma amplified sequence 1	**-1.5**	
MFNG	MFNG O-fucosylpeptide 3-beta-N-acetylglucosaminyltransferase	**-1.8**	**3.0**
MGA	MAX gene associated	**-1.8**	**3.0**
MGAM	maltase-glucoamylase (alpha-glucosidase)		**2.1**
MGAT2	mannosyl (alpha-1,6-)-glycoprotein beta-1,2-N-acetylglucosaminyltransferase		**-1.3**
MGAT4A	mannosyl (alpha-1,3-)-glycoprotein beta-1,4-N-acetylglucosaminyltransferase, isozyme A	**2.1**	**1.9**
MGAT4B	mannosyl (alpha-1,3-)-glycoprotein beta-1,4-N-acetylglucosaminyltransferase, isozyme B	**1.5**	
MGC10540	vacuolar protein sorting 25 homolog (S. cerevisiae)		**-1.5**
MGC17330	HGFL gene	**7.3**	**34.8**
MGC5306	Josephin domain containing 3	**-1.4**	**2.6**
MGC5508	transmembrane protein 109	**-1.7**	**-1.4**
MGC61598	similar to ankyrin-repeat protein Nrarp		**1.6**
MGMT	O-6-methylguanine-DNA methyltransferase	**-1.9**	
MGST1	microsomal glutathione S-transferase 1		**51.7**
MGST3	microsomal glutathione S-transferase 3	**1.5**	
MIB1	mindbomb homolog 1 (Drosophila)		**4.1**
MICA	MHC class I polypeptide-related sequence A	**1.5**	
MICAL1	microtubule associated monoxygenase, calponin and LIM domain containing 1	**1.5**	**-20.5**
MICB	MHC class I polypeptide-related sequence B		**-1.4**
MID1IP1	MID1 interacting protein 1 (gastrulation specific G12 homolog (zebrafish))	**-2.4**	**-1.9**
MI-ER1	mesoderm induction early response 1 homolog (Xenopus laevis)	**1.8**	**2.1**
MIF	macrophage migration inhibitory factor (glycosylation-inhibiting factor)	**-1.9**	
MINA	MYC induced nuclear antigen	**-1.7**	**-1.2**
MINPP1	multiple inositol polyphosphate histidine phosphatase, 1		**1.6**
MIR16	membrane interacting protein of RGS16	**-1.4**	**2.1**
MIS12	MIS12, MIND kinetochore complex component, homolog (yeast)		**-3.0**
MKI67	antigen identified by monoclonal antibody Ki-67	**2.1**	**-2.2**
MKI67IP	MKI67 (FHA domain) interacting nucleolar phosphoprotein	**-2.6**	**1.4**
MKL1	megakaryoblastic leukemia (translocation) 1	**-1.5**	**1.5**
MKL2	MKL/myocardin-like 2	**-2.4**	
MKLN1	muskelin 1, intracellular mediator containing kelch motifs	**1.8**	**-2.0**
MKNK1	MAP kinase interacting serine/threonine kinase 1		**1.3**
MKNK2	MAP kinase interacting serine/threonine kinase 2	**-1.3**	**1.6**
MKRN1	makorin, ring finger protein, 1		**-1.5**
MLC1	megalencephalic leukoencephalopathy with subcortical cysts 1		**1.7**
MLF1	myeloid leukemia factor 1		**4.9**
MLH1	mutL homolog 1, colon cancer, nonpolyposis type 2 (E. coli)	**-1.6**	
MLH3	mutL homolog 3 (E. coli)	**-1.3**	**2.2**
MLL	myeloid/lymphoid or mixed-lineage leukemia (trithorax homolog, Drosophila)	**-1.8**	**1.8**
MLL2	myeloid/lymphoid or mixed-lineage leukemia 2	**-1.7**	**1.5**

MLL3	myeloid/lymphoid or mixed-lineage leukemia 3	**1.3**	**-2.6**
MLLT10	myeloid/lymphoid or mixed-lineage leukemia (trithorax homolog, Drosophila); translocated to, 10		**2.7**
MLLT3	myeloid/lymphoid or mixed-lineage leukemia (trithorax homolog, Drosophila); translocated to, 3		**2.8**
MLLT4	myeloid/lymphoid or mixed-lineage leukemia (trithorax homolog, Drosophila); translocated to, 4	**2.0**	**-1.4**
MMD	monocyte to macrophage differentiation-associated	**-2.6**	
MME	membrane metallo-endopeptidase (neutral endopeptidase, enkephalinase)	**-1.3**	
MMP14	matrix metallopeptidase 14 (membrane-inserted)	**-1.3**	
MMP16	matrix metallopeptidase 16 (membrane-inserted)		**-1.6**
MMRP19	APAF1 interacting protein		**-1.3**
MNDA	myeloid cell nuclear differentiation antigen		**3.8**
MNT	MAX binding protein		**-1.5**
MOAP1	modulator of apoptosis 1	**1.5**	
MOBK1B	MOB1, Mps One Binder kinase activator-like 1B (yeast)		**-1.6**
MONDOA	MLX interacting protein	**1.7**	**1.5**
MORF4L1	mortality factor 4 like 1		**-1.5**
MORF4L2	mortality factor 4 like 2		**1.2**
MOV10	Mov10, Moloney leukemia virus 10, homolog (mouse)	**-1.6**	**-1.6**
MPG	N-methylpurine-DNA glycosylase		**-4.1**
MPHOSPH1	M-phase phosphoprotein 1		**1.9**
MPHOSPH10	M-phase phosphoprotein 10 (U3 small nucleolar ribonucleoprotein)	**-1.3**	
MPHOSPH6	M-phase phosphoprotein 6	**-1.9**	**-1.3**
MPO	myeloperoxidase		**743.3**
MPP1	membrane protein, palmitoylated 1, 55kDa	**-1.3**	**-1.9**
MPP6	membrane protein, palmitoylated 6 (MAGUK p55 subfamily member 6)		**38.6**
MPST	mercaptopyruvate sulfurtransferase	**1.3**	
MPZL1	myelin protein zero-like 1	**-1.8**	**-7.5**
MRC1	mannose receptor, C type 1	**4.6**	
MRC2	mannose receptor, C type 2		**2.0**
MRCL3	myosin regulatory light chain MRCL3	**2.2**	**-2.0**
MRFAP1	Mof4 family associated protein 1	**1.3**	
M-RIP	myosin phosphatase-Rho interacting protein	**1.2**	**-5.4**
MRPL11	mitochondrial ribosomal protein L11	**-1.4**	
MRPL12	mitochondrial ribosomal protein L12	**-1.7**	
MRPL13	mitochondrial ribosomal protein L13		**-2.1**
MRPL37	mitochondrial ribosomal protein L37		**-1.3**
MRPL38	mitochondrial ribosomal protein L38	**-1.3**	
MRPL42	mitochondrial ribosomal protein L42	**-1.9**	**-1.9**
MRPS10	mitochondrial ribosomal protein S10	**-1.4**	**1.2**
MRPS12	mitochondrial ribosomal protein S12		**-1.5**
MRPS15	mitochondrial ribosomal protein S15	**-1.8**	**-1.3**
MRPS27	mitochondrial ribosomal protein S27	**-1.5**	
MS4A1	membrane-spanning 4-domains, subfamily A, member 1	**-6.1**	

MS4A4A	membrane-spanning 4-domains, subfamily A, member 4	**2.4**	
MSH2	mutS homolog 2, colon cancer, nonpolyposis type 1 (E. coli)	**-1.6**	**-1.5**
MSH3	mutS homolog 3 (E. coli)	**-1.5**	
MSH6	mutS homolog 6 (E. coli)	**-2.7**	**-2.5**
MSN	moesin	**1.2**	**1.4**
MSRB3	methionine sulfoxide reductase B3		**149.6**
MSX1	msh homeobox 1	**-1.6**	
MT1E	metallothionein 1E (functional)	**2.3**	**1.8**
MT1G	metallothionein 1G	**1.7**	
MT1H	metallothionein 1H	**3.0**	**1.4**
MT1X	metallothionein 1X	**1.8**	**1.6**
MT2A	metallothionein 2A	**3.0**	**3.2**
MTA1	metastasis associated 1	**-1.4**	**1.4**
MTAP	methylthioadenosine phosphorylase		**13.2**
MTBP	Mdm2, transformed 3T3 cell double minute 2, p53 binding protein (mouse) binding protein, 104kDa		**-1.7**
MTCH1	mitochondrial carrier homolog 1 (C. elegans)	**1.7**	**1.3**
MTCH2	mitochondrial carrier homolog 2 (C. elegans)	**-1.4**	**2.2**
MTCP1	mature T-cell proliferation 1		**-1.4**
MTHFD1	methylenetetrahydrofolate dehydrogenase (NADP+ dependent) 1, methenyltetrahydrofolate cyclohydrolase, formyltetrahydrofolate synthetase	**-2.2**	**1.5**
MTHFD1L	methylenetetrahydrofolate dehydrogenase (NADP+ dependent) 1-like	**-2.9**	**-3.1**
MTHFD2	methylenetetrahydrofolate dehydrogenase (NADP+ dependent) 2, methenyltetrahydrofolate cyclohydrolase	**-2.0**	
MTHFR	5,10-methylenetetrahydrofolate reductase (NADPH)	**1.4**	
MTHFS	5,10-methenyltetrahydrofolate synthetase (5-formyltetrahydrofolate cyclo-ligase)		**-1.4**
MTL5	metallothionein-like 5, testis-specific (tesmin)		**8.4**
MTM1	myotubularin 1		**2.5**
MTMR2	myotubularin related protein 2	**-1.3**	**-1.2**
MTMR6	myotubularin related protein 6	**1.7**	**-1.4**
MTPN	myotrophin		**-1.4**
MTR	5-methyltetrahydrofolate-homocysteine methyltransferase	**1.4**	
MTSS1	metastasis suppressor 1	**6.6**	**13.4**
MTUS1	mitochondrial tumor suppressor 1	**13.8**	
MUC4	mucin 4, cell surface associated	**5.9**	
MUF1	leucine rich repeat containing 41		**1.5**
MULK	multiple substrate lipid kinase	**-1.5**	**5.2**
MUT	methylmalonyl Coenzyme A mutase		**-1.6**
MVP	major vault protein	**-1.5**	
MXD1	MAX dimerization protein 1		**3.3**
MXD4	MAX dimerization protein 4	**3.9**	**13.4**
MXI1	MAX interactor 1	**-1.7**	**6.4**
MYB	v-myb myeloblastosis viral oncogene homolog (avian)	**1.8**	**2.2**
MYBBP1A	MYB binding protein (P160) 1a	**-1.8**	**-1.3**
MYC	v-myc myelocytomatosis viral oncogene homolog (avian)	**-2.8**	**1.6**

MYCBP	c-myc binding protein	-1.2	1.3
MYCBP2	MYC binding protein 2	-1.4	-1.5
MYCN	v-myc myelocytomatosis viral related oncogene, neuroblastoma derived (avian)		7.3
MYD88	myeloid differentiation primary response gene (88)	-1.6	
MYH10	myosin, heavy chain 10, non-muscle	-1.3	-3.7
MYH11	myosin, heavy chain 11, smooth muscle	1.7	-2.6
MYH9	myosin, heavy chain 9, non-muscle		-1.8
MYL4	myosin, light chain 4, alkali; atrial, embryonic	1.3	
MYL5	myosin, light chain 5, regulatory		7.5
MYL6	myosin, light chain 6, alkali, smooth muscle and non-muscle	1.5	
MYL6B	myosin, light chain 6B, alkali, smooth muscle and non-muscle		-1.7
MYLK	myosin, light chain kinase	3.3	
MYO10	myosin X	5.3	-2.3
MYO1B	myosin IB	2.3	3.5
MYO5A	myosin VA (heavy chain 12, myoxin)		-8.2
MYO5C	myosin VC	1.3	-1.4
MYO6	myosin VI	2.1	
MYO9B	myosin IXB	-1.3	
MYOCD	myocardin	-10.3	
MYRIP	myosin VIIA and Rab interacting protein	2.7	-2.4
MYST1	MYST histone acetyltransferase 1		-1.9
MYST2	MYST histone acetyltransferase 2	-1.3	
MYST3	MYST histone acetyltransferase (monocytic leukemia) 3	1.4	
MYST4	MYST histone acetyltransferase (monocytic leukemia) 4	-1.9	2.2
N4BP3	Nedd4 binding protein 3		-2.2
NACA	nascent-polypeptide-associated complex alpha polypeptide		1.3
NADK	NAD kinase	1.3	
NADSYN1	NAD synthetase 1		2.0
NAGA	N-acetylgalactosaminidase, alpha-		1.6
NAGLU	N-acetylglucosaminidase, alpha- (Sanfilippo disease IIIB)	-1.3	1.3
NANOS1	nanos homolog 1 (Drosophila)		23.2
NANS	N-acetylneuraminic acid synthase (sialic acid synthase)	-1.5	1.4
NAP1L1	nucleosome assembly protein 1-like 1	-1.2	1.9
NAP1L4	nucleosome assembly protein 1-like 4		-1.5
NAPA	N-ethylmaleimide-sensitive factor attachment protein, alpha		-1.5
NAPG	N-ethylmaleimide-sensitive factor attachment protein, gamma	1.5	
NARS	asparaginyl-tRNA synthetase	-1.4	
NARS2	asparaginyl-tRNA synthetase 2 (mitochondrial)(putative)	-1.8	-2.5
NASP	nuclear autoantigenic sperm protein (histone-binding)	-2.4	
NAT1	N-acetyltransferase 1 (arylamine N-acetyltransferase)	1.3	1.2
NBL1	neuroblastoma, suppression of tumorigenicity 1		2.3
NBR1	neighbor of BRCA1 gene 1	1.5	2.6

NBS1	nibrin	**-1.2**	**-1.6**
NCALD	neurocalcin delta	**-1.9**	
NCBP1	nuclear cap binding protein subunit 1, 80kDa	**-1.2**	**-1.3**
NCF1	neutrophil cytosolic factor 1C pseudogene	**9.4**	
NCF2	neutrophil cytosolic factor 2 (65kDa, chronic granulomatous disease, autosomal 2)		**63.4**
NCF4	neutrophil cytosolic factor 4, 40kDa	**-1.5**	**-1.9**
NCK1	NCK adaptor protein 1	**1.5**	**1.7**
NCK2	NCK adaptor protein 2		**3.0**
NCKAP1	NCK-associated protein 1		**6.0**
NCL	nucleolin	**-1.9**	**-1.6**
NCOA1	nuclear receptor coactivator 1	**5.1**	
NCOA2	nuclear receptor coactivator 2	**1.9**	**-1.5**
NCOA3	nuclear receptor coactivator 3	**2.1**	**-1.4**
NCOA4	nuclear receptor coactivator 4	**1.3**	**1.9**
NCOA5	nuclear receptor coactivator 5		**-1.4**
NCOA6IP	trimethylguanosine synthase homolog (S. cerevisiae)		**-1.2**
NCOA7	nuclear receptor coactivator 7	**1.5**	
NCOR1	nuclear receptor co-repressor 1	**-1.5**	**-1.9**
NCOR2	nuclear receptor co-repressor 2	**1.5**	
NCSTN	nicastrin		**1.9**
NDE1	nudE nuclear distribution gene E homolog 1 (A. nidulans)	**1.5**	**-1.4**
NDEL1	nudE nuclear distribution gene E homolog like 1 (A. nidulans)		**-1.7**
NDFIP1	Nedd4 family interacting protein 1	**1.4**	**51.5**
NDRG1	N-myc downstream regulated gene 1	**3.1**	**9.2**
NDST1	N-deacetylase/N-sulfotransferase (heparan glucosaminyl) 1		**2.5**
NDUFA10	NADH dehydrogenase (ubiquinone) 1 alpha subcomplex, 10, 42kDa		**-1.3**
NDUFA11	NADH dehydrogenase (ubiquinone) 1 alpha subcomplex, 11, 14.7kDa		**-1.8**
NDUFA12	NADH dehydrogenase (ubiquinone) 1 alpha subcomplex, 12	**-1.5**	**-1.2**
NDUFA3	NADH dehydrogenase (ubiquinone) 1 alpha subcomplex, 3, 9kDa	**-1.2**	
NDUFA5	NADH dehydrogenase (ubiquinone) 1 alpha subcomplex, 5, 13kDa		**-2.4**
NDUFA6	NADH dehydrogenase (ubiquinone) 1 alpha subcomplex, 6, 14kDa		**-1.2**
NDUFA8	NADH dehydrogenase (ubiquinone) 1 alpha subcomplex, 8, 19kDa	**-1.3**	
NDUFA9	NADH dehydrogenase (ubiquinone) 1 alpha subcomplex, 9, 39kDa		**-2.1**
NDUFAB1	NADH dehydrogenase (ubiquinone) 1, alpha/beta subcomplex, 1, 8kDa	**-1.5**	**-1.9**
NDUFB10	NADH dehydrogenase (ubiquinone) 1 beta subcomplex, 10, 22kDa		**-3.0**
NDUFB2	NADH dehydrogenase (ubiquinone) 1 beta subcomplex, 2, 8kDa		**-1.5**
NDUFB3	NADH dehydrogenase (ubiquinone) 1 beta subcomplex, 3, 12kDa		**1.2**
NDUFB5	NADH dehydrogenase (ubiquinone) 1 beta subcomplex, 5, 16kDa		**1.7**
NDUFB7	NADH dehydrogenase (ubiquinone) 1 beta subcomplex, 7, 18kDa	**1.9**	**-1.3**
NDUFB8	NADH dehydrogenase (ubiquinone) 1 beta subcomplex, 8, 19kDa		**1.2**
NDUFB9	NADH dehydrogenase (ubiquinone) 1 beta subcomplex, 9, 22kDa	**2.3**	**6.9**
NDUFC1	NADH dehydrogenase (ubiquinone) 1, subcomplex unknown, 1, 6kDa		**1.4**

NDUFS1	NADH dehydrogenase (ubiquinone) Fe-S protein 1, 75kDa (NADH-coenzyme Q reductase)	**-1.5**	**-1.6**
NDUFS2	NADH dehydrogenase (ubiquinone) Fe-S protein 2, 49kDa (NADH-coenzyme Q reductase)		**1.9**
NDUFS3	NADH dehydrogenase (ubiquinone) Fe-S protein 3, 30kDa (NADH-coenzyme Q reductase)	**-1.4**	
NDUFS5	NADH dehydrogenase (ubiquinone) Fe-S protein 5, 15kDa (NADH-coenzyme Q reductase)	**-1.5**	**-1.9**
NDUFS6	NADH dehydrogenase (ubiquinone) Fe-S protein 6, 13kDa (NADH-coenzyme Q reductase)		**-1.5**
NDUFS7	NADH dehydrogenase (ubiquinone) Fe-S protein 7, 20kDa (NADH-coenzyme Q reductase)	**-1.6**	
NDUFS8	NADH dehydrogenase (ubiquinone) Fe-S protein 8, 23kDa (NADH-coenzyme Q reductase)	**-1.5**	
NDUFV1	NADH dehydrogenase (ubiquinone) flavoprotein 1, 51kDa	**-1.4**	**-1.6**
NDUFV3	NADH dehydrogenase (ubiquinone) flavoprotein 3, 10kDa		**2.2**
NECAP2	NECAP endocytosis associated 2	**1.4**	
NEDD4	neural precursor cell expressed, developmentally down-regulated 4		**2.8**
NEDD4L	neural precursor cell expressed, developmentally down-regulated 4-like		**5.3**
NEDD9	neural precursor cell expressed, developmentally down-regulated 9	**5.8**	**-1.7**
NEK1	NIMA (never in mitosis gene a)-related kinase 1	**-2.4**	**-1.5**
NEK2	NIMA (never in mitosis gene a)-related kinase 2	**1.5**	**-1.9**
NEK4	NIMA (never in mitosis gene a)-related kinase 4	**1.3**	
NEK6	NIMA (never in mitosis gene a)-related kinase 6		**5.5**
NEK8	NIMA (never in mitosis gene a)- related kinase 8		**-2.6**
NET1	neuroepithelial cell transforming gene 1	**1.2**	**1.7**
NEU1	sialidase 1 (lysosomal sialidase)		**2.5**
NEXN	nexilin (F actin binding protein)	**-5.6**	**-56.2**
NF1	neurofibromin 1 (neurofibromatosis, von Recklinghausen disease, Watson disease)	**2.0**	
NF2	neurofibromin 2 (bilateral acoustic neuroma)		**-1.3**
NFAT5	nuclear factor of activated T-cells 5, tonicity-responsive		**6.1**
NFATC1	nuclear factor of activated T-cells, cytoplasmic, calcineurin-dependent 1	**2.3**	
NFATC2	nuclear factor of activated T-cells, cytoplasmic, calcineurin-dependent 2		**-1.9**
NFATC2IP	nuclear factor of activated T-cells, cytoplasmic, calcineurin-dependent 2 interacting protein	**-1.3**	**-2.4**
NFATC3	nuclear factor of activated T-cells, cytoplasmic, calcineurin-dependent 3	**-1.4**	**-1.8**
NFE2L1	nuclear factor (erythroid-derived 2)-like 1		**1.6**
NFE2L2	nuclear factor (erythroid-derived 2)-like 2	**1.3**	**2.2**
NFE2L3	nuclear factor (erythroid-derived 2)-like 3		**18.4**
NFIB	nuclear factor I/B		**-2.1**
NFIC	nuclear factor I/C (CCAAT-binding transcription factor)	**-1.9**	**5.9**
NFIL3	nuclear factor, interleukin 3 regulated	**5.0**	**-2.5**
NFIX	nuclear factor I/X (CCAAT-binding transcription factor)	**-2.1**	**-1.8**
NFKB1	nuclear factor of kappa light polypeptide gene enhancer in B-cells 1 (p105)	**-2.2**	
NFKB2	nuclear factor of kappa light polypeptide gene enhancer in B-cells 2 (p49/p100)		**-1.5**
NFKBIA	nuclear factor of kappa light polypeptide gene enhancer in B-cells inhibitor, alpha	**2.5**	**1.9**
NFKBIZ	nuclear factor of kappa light polypeptide gene enhancer in B-cells inhibitor, zeta	**7.3**	**3.5**
NFRKB	nuclear factor related to kappaB binding protein		**2.2**
NFYA	nuclear transcription factor Y, alpha		**1.6**
NFYB	nuclear transcription factor Y, beta		**-1.6**

NFYC	nuclear transcription factor Y, gamma	**-1.3**	**-1.4**
NGFR	nerve growth factor receptor (TNFR superfamily, member 16)	**6.7**	
NGLY1	N-glycanase 1		**1.4**
NHP2L1	NHP2 non-histone chromosome protein 2-like 1 (S. cerevisiae)	**-1.6**	
NIPSNAP1	nipsnap homolog 1 (C. elegans)	**-2.9**	
NKD2	naked cuticle homolog 2 (Drosophila)	**1.5**	
NKIR	CD300 molecule-like family member f		**5.3**
NKTR	natural killer-tumor recognition sequence		**2.0**
NLK	nemo-like kinase	**-1.3**	**-1.8**
NMB	neuromedin B		**1.4**
NME1	non-metastatic cells 1, protein (NM23A) expressed in	**-4.4**	
NME4	non-metastatic cells 4, protein expressed in	**-2.1**	
NME6	non-metastatic cells 6, protein expressed in (nucleoside-diphosphate kinase)		**1.6**
NME7	non-metastatic cells 7, protein expressed in (nucleoside-diphosphate kinase)	**-2.4**	
NMI	N-myc (and STAT) interactor		**1.2**
NMNAT1	nicotinamide nucleotide adenylyltransferase 1		**1.4**
NMNAT3	nicotinamide nucleotide adenylyltransferase 3		**10.8**
NMT1	N-myristoyltransferase 1	**-1.3**	
NMT2	N-myristoyltransferase 2	**-2.4**	
NNT	nicotinamide nucleotide transhydrogenase		**1.3**
NOC2L	nucleolar complex associated 2 homolog (S. cerevisiae)	**-1.8**	
NOG	noggin	**-4.9**	**-2.1**
NOL1	nucleolar protein 1, 120kDa	**-1.5**	
NOL5A	nucleolar protein 5A (56kDa with KKE/D repeat)	**-2.0**	
NOL8	nucleolar protein 8	**-1.3**	**1.4**
NOLA2	nucleolar protein family A, member 2 (H/ACA small nucleolar RNPs)	**-1.8**	**-1.5**
NOLA3	nucleolar protein family A, member 3 (H/ACA small nucleolar RNPs)	**-1.2**	**-1.5**
NOLC1	nucleolar and coiled-body phosphoprotein 1	**-2.0**	**1.5**
NOP5/NOP58	nucleolar protein NOP5/NOP58	**-1.6**	**1.4**
NOTCH1	Notch homolog 1, translocation-associated (Drosophila)		**-2.3**
NOTCH2	Notch homolog 2 (Drosophila)	**2.5**	**-5.3**
NOV	nephroblastoma overexpressed gene		**2.9**
NP	nucleoside phosphorylase	**-1.3**	
N-PAC	cytokine-like nuclear factor n-pac	**-1.5**	**-1.5**
NPAT	nuclear protein, ataxia-telangiectasia locus	**-1.2**	
NPC2	Niemann-Pick disease, type C2		**1.8**
NPHP3	nephronophthisis 3 (adolescent)	**1.7**	**1.9**
NPL4	nuclear protein localization 4 homolog (S. cerevisiae)		**1.6**
NPM1	nucleophosmin (nucleolar phosphoprotein B23, numatrin)	**-1.9**	**1.2**
NPM3	nucleophosmin/nucleoplasmin, 3		**2.5**
NPR3	natriuretic peptide receptor C/guanylate cyclase C (atrionatriuretic peptide receptor C)		**5.2**
NQO2	NAD(P)H dehydrogenase, quinone 2	**-1.5**	**2.2**

NR1D2	nuclear receptor subfamily 1, group D, member 2		**1.3**
NR2C1	nuclear receptor subfamily 2, group C, member 1	**1.3**	**1.6**
NR3C1	nuclear receptor subfamily 3, group C, member 1 (glucocorticoid receptor)	**3.1**	**-1.8**
NR3C2	nuclear receptor subfamily 3, group C, member 2	**2.4**	
NRAS	neuroblastoma RAS viral (v-ras) oncogene homolog		**-1.4**
NRD1	nardilysin (N-arginine dibasic convertase)		**1.7**
NRF1	nuclear respiratory factor 1	**1.5**	**-1.4**
NRGN	neurogranin (protein kinase C substrate, RC3)	**-3.1**	**1.2**
NRIP1	nuclear receptor interacting protein 1	**1.5**	**-1.4**
NRN1	neuritin 1	**1.4**	**-1.4**
NRP1	neuropilin 1		**-1.8**
NSBP1	nucleosomal binding protein 1		**1.8**
NSEP1	Y box binding protein 1	**-1.7**	
NSF	N-ethylmaleimide-sensitive factor	**-1.3**	**1.4**
NSFL1C	NSFL1 (p97) cofactor (p47)	**1.5**	**1.5**
NSMAF	neutral sphingomyelinase (N-SMase) activation associated factor	**-2.7**	**-1.3**
NT5C2	5'-nucleotidase, cytosolic II	**1.5**	**2.3**
NT5C3	5'-nucleotidase, cytosolic III		**3.3**
NT5E	5'-nucleotidase, ecto (CD73)	**4.8**	
NTHL1	nth endonuclease III-like 1 (E. coli)	**-1.7**	**-2.2**
NUBP1	nucleotide binding protein 1 (MinD homolog, E. coli)		**-1.9**
NUCB2	nucleobindin 2	**-1.3**	**1.3**
NUCKS	nuclear casein kinase and cyclin-dependent kinase substrate 1	**1.3**	**-2.1**
NUDC	nuclear distribution gene C homolog (A. nidulans)	**-1.5**	**-1.2**
NUDCD3	NudC domain containing 3		**2.2**
NUDT1	nudix (nucleoside diphosphate linked moiety X)-type motif 1		**-2.3**
NUDT5	nudix (nucleoside diphosphate linked moiety X)-type motif 5	**-1.8**	**1.3**
NUDT9	nudix (nucleoside diphosphate linked moiety X)-type motif 9	**-1.4**	
NUFIP1	nuclear fragile X mental retardation protein interacting protein 1	**-1.8**	**-1.8**
NUMA1	nuclear mitotic apparatus protein 1		**-2.1**
NUP107	nucleoporin 107kDa	**-1.5**	
NUP153	nucleoporin 153kDa	**-1.4**	**-2.0**
NUP155	nucleoporin 155kDa		**-1.5**
NUP210	nucleoporin 210kDa	**-1.7**	**-3.4**
NUP50	nucleoporin 50kDa		**-1.3**
NUP62	nucleoporin 62kDa		**-1.4**
NUP88	nucleoporin 88kDa		**-2.2**
NUP98	nucleoporin 98kDa	**-2.0**	**-1.7**
NUPL2	nucleoporin like 2		**3.8**
NUSAP1	nucleolar and spindle associated protein 1	**1.8**	**-2.8**
NUTF2	nuclear transport factor 2	**-1.6**	**-1.5**
NXF1	nuclear RNA export factor 1		**1.3**

NXT1	NTF2-like export factor 1	**-1.8**	**-1.4**
NYREN18	negative regulator of ubiquitin-like proteins 1		**-2.3**
OAS2	2'-5'-oligoadenylate synthetase 2, 69/71kDa	**-2.8**	**-3.2**
OAT	ornithine aminotransferase (gyrate atrophy)		**90.9**
OAZ1	ornithine decarboxylase antizyme 1	**1.4**	**-1.3**
OAZIN	antizyme inhibitor 1		**-2.2**
ODC1	ornithine decarboxylase 1	**-1.5**	**-1.2**
ODF1	outer dense fiber of sperm tails 1	**-1.6**	
OGDH	oxoglutarate (alpha-ketoglutarate) dehydrogenase (lipoamide)	**-1.7**	**1.8**
OGFOD2	2-oxoglutarate and iron-dependent oxygenase domain containing 2	**-1.3**	
OGG1	8-oxoguanine DNA glycosylase		**-3.9**
OGT	O-linked N-acetylglucosamine (GlcNAc) transferase (UDP-N-acetylglucosamine:polypeptide-N-acetylglucosaminyl transferase)	**1.3**	**2.0**
OIP5	Opa interacting protein 5		**-2.6**
OLAH	oleoyl-ACP hydrolase	**18.7**	
OMA1	OMA1 homolog, zinc metallopeptidase (S. cerevisiae)		**1.2**
OPA1	optic atrophy 1 (autosomal dominant)		**1.5**
OPN1SW	opsin 1 (cone pigments), short-wave-sensitive (color blindness, tritan)	**-2.1**	
OPN3	opsin 3 (encephalopsin, panopsin)	**1.4**	**4.6**
OPN5	opsin 5	**-1.3**	
OPTN	optineurin	**3.8**	
ORC1L	origin recognition complex, subunit 1-like (yeast)		**1.7**
ORC2L	origin recognition complex, subunit 2-like (yeast)		**2.6**
ORC3L	origin recognition complex, subunit 3-like (yeast)		**1.3**
ORC4L	origin recognition complex, subunit 4-like (yeast)		**2.3**
ORC5L	origin recognition complex, subunit 5-like (yeast)		**1.3**
OSBPL1A	oxysterol binding protein-like 1A		**2.4**
OSBPL8	oxysterol binding protein-like 8		**-1.3**
OSTF1	osteoclast stimulating factor 1	**1.2**	**-1.3**
OSTM1	osteopetrosis associated transmembrane protein 1	**1.7**	**-1.5**
OXSR1	oxidative-stress responsive 1		**1.3**
P29	SYF2 homolog, RNA splicing factor (S. cerevisiae)	**1.4**	
P2RX1	purinergic receptor P2X, ligand-gated ion channel, 1	**1.8**	**-2.1**
P2RX4	purinergic receptor P2X, ligand-gated ion channel, 4		**2.0**
P2RX5	purinergic receptor P2X, ligand-gated ion channel, 5	**5.5**	**-2.0**
P2RY1	purinergic receptor P2Y, G-protein coupled, 1		**3.1**
P4HA1	procollagen-proline, 2-oxoglutarate 4-dioxygenase (proline 4-hydroxylase), alpha polypeptide I		**3.0**
P4HB	procollagen-proline, 2-oxoglutarate 4-dioxygenase (proline 4-hydroxylase), beta polypeptide		**3.7**
P53CSV	TP53 regulated inhibitor of apoptosis 1	**-1.4**	
PA2G4	proliferation-associated 2G4, 38kDa	**-2.5**	
PABPC1	poly(A) binding protein, cytoplasmic 1	**-1.4**	**-2.2**
PABPC4	poly(A) binding protein, cytoplasmic 4 (inducible form)	**-1.8**	
PABPN1	poly(A) binding protein, nuclear 1	**-1.3**	**1.6**

PACAP	hypothetical protein MGC29506		**-48.6**
PACSIN1	protein kinase C and casein kinase substrate in neurons 1	**3.0**	
PACSIN2	protein kinase C and casein kinase substrate in neurons 2	**1.4**	
PAEP	progestagen-associated endometrial protein (placental protein 14, pregnancy-associated endometrial alpha-2-globulin, a		**1.5**
PAF53	polymerase (RNA) I polypeptide E, 53kDa	**-1.3**	
PAFAH1B1	platelet-activating factor acetylhydrolase, isoform Ib, alpha subunit 45kDa	**1.2**	**-2.4**
PAG	phosphoprotein associated with glycosphingolipid microdomains 1	**2.1**	**-3.0**
PAICS	phosphoribosylaminoimidazole carboxylase, phosphoribosylaminoimidazole succinocarboxamide synthetase	**-3.3**	**1.7**
PAIP1	poly(A) binding protein interacting protein 1	**-1.4**	
PAIP2	poly(A) binding protein interacting protein 2		**-1.3**
PAI-RBP1	SERPINE1 mRNA binding protein 1	**-1.9**	**-1.5**
PAK1	p21/Cdc42/Rac1-activated kinase 1 (STE20 homolog, yeast)	**-1.3**	**2.5**
PAK1IP1	PAK1 interacting protein 1	**-1.7**	
PAK2	p21 (CDKN1A)-activated kinase 2	**1.5**	**-1.6**
PAK4	p21(CDKN1A)-activated kinase 4	**1.4**	
PAK6	p21(CDKN1A)-activated kinase 6		**2.6**
PALM	paralemmin		**2.7**
PAM	peptidylglycine alpha-amidating monooxygenase	**-1.4**	**1.3**
PAN3	PAN3 polyA specific ribonuclease subunit homolog (S. cerevisiae)	**1.6**	**-8.3**
PANK1	pantothenate kinase 1	**-2.1**	
PANK2	pantothenate kinase 2 (Hallervorden-Spatz syndrome)		**1.4**
PANK3	pantothenate kinase 3	**1.3**	
PAPOLA	poly(A) polymerase alpha		**1.4**
PAPSS1	3'-phosphoadenosine 5'-phosphosulfate synthase 1	**-1.4**	
PAPSS2	3'-phosphoadenosine 5'-phosphosulfate synthase 2		**98.1**
PARD3	par-3 partitioning defective 3 homolog (C. elegans)		**2.5**
PARD6B	par-6 partitioning defective 6 homolog beta (C. elegans)		**-1.9**
PARD6G	par-6 partitioning defective 6 homolog gamma (C. elegans)	**1.6**	**1.4**
PARN	poly(A)-specific ribonuclease (deadenylation nuclease)		**-2.1**
PARP1	poly (ADP-ribose) polymerase family, member 1	**1.7**	**-2.5**
PARP2	poly (ADP-ribose) polymerase family, member 2	**-1.6**	
PARP9	poly (ADP-ribose) polymerase family, member 9	**1.3**	
PARVB	parvin, beta	**1.5**	**51.7**
PAWR	PRKC, apoptosis, WT1, regulator	**-2.4**	**42.6**
PAX5	paired box gene 5 (B-cell lineage specific activator)	**-1.5**	**-15.6**
PAX8	paired box gene 8		**3.7**
PAXIP1L	PAX interacting (with transcription-activation domain) protein 1	**1.2**	**-3.5**
PBEF1	pre-B-cell colony enhancing factor 1		**1.7**
PBK	PDZ binding kinase		**-2.0**
PBP	phosphatidylethanolamine binding protein 1	**-2.0**	
PBX2	pre-B-cell leukemia transcription factor 2	**-1.6**	**1.7**
PBX3	pre-B-cell leukemia transcription factor 3	**-1.5**	**-172.9**

PBXIP1	pre-B-cell leukemia transcription factor interacting protein 1	**1.6**	
PC4	SUB1 homolog (S. cerevisiae)	**-1.4**	**2.4**
PCBD2	pterin-4 alpha-carbinolamine dehydratase/dimerization cofactor of hepatocyte nuclear factor 1 alpha (TCF1) 2	**1.8**	**1.7**
PCBP2	poly(rC) binding protein 2		**2.0**
PCCA	propionyl Coenzyme A carboxylase, alpha polypeptide	**-1.2**	
PCCB	propionyl Coenzyme A carboxylase, beta polypeptide		**1.3**
PCDHGC3	protocadherin gamma subfamily C, 3	**-3.1**	**-4.4**
PCF11	PCF11, cleavage and polyadenylation factor subunit, homolog (S. cerevisiae)		**-2.1**
PCGF4	B lymphoma Mo-MLV insertion region (mouse)	**1.3**	**-1.5**
PCGF6	polycomb group ring finger 6		**1.2**
PCK2	phosphoenolpyruvate carboxykinase 2 (mitochondrial)	**-1.7**	
PCM1	pericentriolar material 1	**1.8**	**2.3**
PCMT1	protein-L-isoaspartate (D-aspartate) O-methyltransferase	**1.4**	**-1.3**
PCNA	proliferating cell nuclear antigen	**-1.6**	**-1.7**
PCNT2	pericentrin (kendrin)		**1.4**
PCNX	pecanex homolog (Drosophila)		**2.8**
PCOLN3	procollagen (type III) N-endopeptidase	**-1.7**	
PCTK1	PCTAIRE protein kinase 1		**-1.4**
PCTK2	PCTAIRE protein kinase 2		**-1.2**
PCTP	phosphatidylcholine transfer protein	**5.3**	**3.7**
PDCD2	programmed cell death 2	**1.3**	**2.0**
PDCD4	programmed cell death 4 (neoplastic transformation inhibitor)		**3.9**
PDCD5	programmed cell death 5		**-1.2**
PDCD6	programmed cell death 6	**2.0**	**-11.8**
PDCD6IP	programmed cell death 6 interacting protein		**1.5**
PDCD8	apoptosis-inducing factor, mitochondrion-associated, 1	**-1.6**	
PDCL	phosducin-like		**-1.7**
PDE1A	phosphodiesterase 1A, calmodulin-dependent	**2.3**	
PDE3B	phosphodiesterase 3B, cGMP-inhibited	**-1.4**	**6.1**
PDE4A	phosphodiesterase 4A, cAMP-specific (phosphodiesterase E2 dunce homolog, Drosophila)	**-1.5**	
PDE4C	phosphodiesterase 4C, cAMP-specific (phosphodiesterase E1 dunce homolog, Drosophila)	**1.5**	
PDE4D	phosphodiesterase 4D, cAMP-specific (phosphodiesterase E3 dunce homolog, Drosophila)		**5.3**
PDE6D	phosphodiesterase 6D, cGMP-specific, rod, delta	**1.2**	**-2.1**
PDE7A	phosphodiesterase 7A	**1.8**	**-2.1**
PDE8A	phosphodiesterase 8A	**1.6**	**-1.5**
PDGFA	platelet-derived growth factor alpha polypeptide	**1.3**	
PDGFB	platelet-derived growth factor beta polypeptide (simian sarcoma viral (v-sis) oncogene homolog)	**-5.4**	
PDGFC	platelet derived growth factor C		**40.5**
PDGFD	platelet derived growth factor D	**2.1**	
PDGFRB	platelet-derived growth factor receptor, beta polypeptide		**-3.6**
PDHA1	pyruvate dehydrogenase (lipoamide) alpha 1	**-1.6**	
PDHX	pyruvate dehydrogenase complex, component X		**-1.5**

PDIA3	protein disulfide isomerase family A, member 3		**-1.3**
PDIA4	protein disulfide isomerase family A, member 4		**-1.2**
PDIA6	protein disulfide isomerase family A, member 6	**-1.3**	**-1.5**
PDK1	pyruvate dehydrogenase kinase, isozyme 1		**2.8**
PDK2	pyruvate dehydrogenase kinase, isozyme 2		**3.1**
PDK3	pyruvate dehydrogenase kinase, isozyme 3	**-1.6**	**-1.3**
PDK4	pyruvate dehydrogenase kinase, isozyme 4	**5.9**	
PDLIM1	PDZ and LIM domain 1 (elfin)		**-4.0**
PDLIM5	PDZ and LIM domain 5	**-1.6**	**1.6**
PDLIM7	PDZ and LIM domain 7 (enigma)	**-2.1**	
PDPK1	3-phosphoinositide dependent protein kinase-1		**-1.8**
PDPR	pyruvate dehydrogenase phosphatase regulatory subunit		**1.4**
PDXK	pyridoxal (pyridoxine, vitamin B6) kinase		**2.8**
PDZK3	PDZ domain containing 2	**1.8**	
PEA15	phosphoprotein enriched in astrocytes 15		**-1.3**
PECAM1	platelet/endothelial cell adhesion molecule (CD31 antigen)	**-1.7**	
PECI	peroxisomal D3,D2-enoyl-CoA isomerase	**-2.2**	**1.3**
PEF1	penta-EF-hand domain containing 1		**-1.7**
PELI1	pellino homolog 1 (Drosophila)		**-4.9**
PELI2	pellino homolog 2 (Drosophila)		**-5.0**
PELO	pelota homolog (Drosophila)		**1.5**
PEMT	phosphatidylethanolamine N-methyltransferase		**-3.7**
PEO1	progressive external ophthalmoplegia 1	**-3.1**	**1.3**
PEPD	peptidase D	**-1.8**	
PER2	period homolog 2 (Drosophila)	**-1.7**	**-2.0**
PEX1	peroxisome biogenesis factor 1	**1.5**	
PEX11B	peroxisomal biogenesis factor 11B	**1.4**	**-1.4**
PEX14	peroxisomal biogenesis factor 14		**1.3**
PEX16	peroxisomal biogenesis factor 16		**1.3**
PEX3	peroxisomal biogenesis factor 3	**-2.4**	**1.3**
PFAS	phosphoribosylformylglycinamidine synthase (FGAR amidotransferase)	**-2.7**	**-2.4**
PFDN2	prefoldin subunit 2	**-1.3**	
PFDN5	prefoldin subunit 5	**1.4**	**1.4**
PFKL	phosphofructokinase, liver	**1.7**	
PFKM	phosphofructokinase, muscle	**-4.3**	**-26.2**
PFKP	phosphofructokinase, platelet	**1.7**	**1.5**
PFN1	profilin 1		**-2.1**
PFTK1	PFTAIRE protein kinase 1	**1.7**	**-3.1**
PGAM1	phosphoglycerate mutase 1 (brain)	**-1.2**	**1.5**
PGD	phosphogluconate dehydrogenase	**-1.3**	**1.8**
PGF	placental growth factor, vascular endothelial growth factor-related protein	**1.4**	
PGGT1B	protein geranylgeranyltransferase type I, beta subunit		**-3.2**

PGK1	phosphoglycerate kinase 1	**-1.9**	**1.3**
PGLS	6-phosphogluconolactonase		**-1.8**
PGM1	phosphoglucomutase 1	**-1.8**	**1.7**
PGM2	phosphoglucomutase 2	**-1.7**	**-1.5**
PGM3	phosphoglucomutase 3		**-2.6**
PGRMC1	progesterone receptor membrane component 1	**-1.5**	
PGRMC2	progesterone receptor membrane component 2		**1.7**
PHACTR1	phosphatase and actin regulator 1	**4.5**	**-3.8**
PHB2	prohibitin 2		**-1.7**
PHC1	polyhomeotic homolog 1 (Drosophila)	**-1.2**	
PHC2	polyhomeotic homolog 2 (Drosophila)		**-1.6**
PHF1	PHD finger protein 1		**1.8**
PHF17	PHD finger protein 17		**-1.4**
PHF19	PHD finger protein 19	**1.3**	**1.5**
PHF21A	PHD finger protein 21A		**2.0**
PHGDH	phosphoglycerate dehydrogenase	**-2.4**	**-1.7**
PHKA1	phosphorylase kinase, alpha 1 (muscle)		**3.0**
PHKA2	phosphorylase kinase, alpha 2 (liver)	**-1.3**	**1.7**
PHKB	phosphorylase kinase, beta	**1.4**	**-1.7**
PHLDA1	pleckstrin homology-like domain, family A, member 1	**7.5**	
PHLDB2	pleckstrin homology-like domain, family B, member 2		**-2.6**
PHYH	phytanoyl-CoA 2-hydroxylase	**1.7**	
PIAS1	protein inhibitor of activated STAT, 1	**1.4**	**1.7**
PIAS2	protein inhibitor of activated STAT, 2	**-1.7**	
PIAS4	protein inhibitor of activated STAT, 4	**1.3**	
PICALM	phosphatidylinositol binding clathrin assembly protein	**2.0**	**2.1**
PIG8	centrosomal protein 57kDa		**1.6**
PIGA	phosphatidylinositol glycan anchor biosynthesis, class A (paroxysmal nocturnal hemoglobinuria)	**1.5**	
PIGC	phosphatidylinositol glycan anchor biosynthesis, class C	**2.5**	**1.9**
PIGK	phosphatidylinositol glycan anchor biosynthesis, class K	**1.4**	**2.0**
PIGO	phosphatidylinositol glycan anchor biosynthesis, class O	**-1.6**	**1.6**
PIGS	phosphatidylinositol glycan anchor biosynthesis, class S		**2.4**
PIK3AP1	phosphoinositide-3-kinase adaptor protein 1	**1.3**	**-4.5**
PIK3C2A	phosphoinositide-3-kinase, class 2, alpha polypeptide	**1.7**	**2.5**
PIK3CA	phosphoinositide-3-kinase, catalytic, alpha polypeptide	**1.3**	**-2.5**
PIK3CB	phosphoinositide-3-kinase, catalytic, beta polypeptide	**1.2**	**3.3**
PIK3CD	phosphoinositide-3-kinase, catalytic, delta polypeptide	**-2.7**	**-1.7**
PIK3CG	phosphoinositide-3-kinase, catalytic, gamma polypeptide	**2.0**	
PIK3R1	phosphoinositide-3-kinase, regulatory subunit 1 (p85 alpha)		**2.1**
PIK3R4	phosphoinositide-3-kinase, regulatory subunit 4, p150		**1.3**
PIK4CB	phosphatidylinositol 4-kinase, catalytic, beta polypeptide	**-1.4**	
PILRB	paired immunoglobin-like type 2 receptor beta	**1.4**	

PIM3	pim-3 oncogene		**-1.4**
PIN1	protein (peptidylprolyl cis/trans isomerase) NIMA-interacting 1	**1.6**	
PIN4	protein (peptidylprolyl cis/trans isomerase) NIMA-interacting, 4 (parvulin)		**-1.3**
PINK1	PTEN induced putative kinase 1		**1.3**
PIP3AP	myotubularin related protein 12		**-3.2**
PIP3-E	phosphoinositide-binding protein PIP3-E		**9.5**
PIP5K1B	phosphatidylinositol-4-phosphate 5-kinase, type I, beta	**-1.8**	
PIP5K1C	phosphatidylinositol-4-phosphate 5-kinase, type I, gamma		**2.7**
PIP5K2A	phosphatidylinositol-4-phosphate 5-kinase, type II, alpha		**1.3**
PIP5K2B	phosphatidylinositol-4-phosphate 5-kinase, type II, beta	**-1.6**	
PIP5K3	phosphatidylinositol-3-phosphate/phosphatidylinositol 5-kinase, type III		**-1.6**
PITPNA	phosphatidylinositol transfer protein, alpha	**-1.3**	**-2.0**
PITPNB	phosphatidylinositol transfer protein, beta		**-1.5**
PITPNM2	phosphatidylinositol transfer protein, membrane-associated 2		**-2.5**
PITRM1	pitrilysin metallopeptidase 1		**1.6**
PJA1	praja 1	**-1.5**	**-2.0**
PKD1	polycystic kidney disease 1 (autosomal dominant)	**3.2**	**-3.0**
PKD2	polycystic kidney disease 2 (autosomal dominant)		**-2.0**
PKIA	protein kinase (cAMP-dependent, catalytic) inhibitor alpha		**-6.9**
PKM2	pyruvate kinase, muscle	**-2.3**	**-3.2**
PKMYT1	protein kinase, membrane associated tyrosine/threonine 1	**1.2**	**-2.2**
PKN2	protein kinase N2	**1.4**	**1.4**
PKN3	protein kinase N3		**3.8**
PKNOX1	PBX/knotted 1 homeobox 1	**1.6**	**-1.7**
PKP2	plakophilin 2		**40.3**
PKP3	plakophilin 3	**-2.0**	
PKP4	plakophilin 4		**2.5**
PLA2G12A	phospholipase A2, group XIIA		**1.9**
PLA2G4B	phospholipase A2, group IVB (cytosolic)		**-1.3**
PLA2G6	phospholipase A2, group VI (cytosolic, calcium-independent)	**1.3**	
PLAA	phospholipase A2-activating protein		**-1.3**
PLAG1	pleiomorphic adenoma gene 1	**1.4**	**-5.2**
PLAGL1	pleiomorphic adenoma gene-like 1		**15.8**
PLAUR	plasminogen activator, urokinase receptor	**-3.9**	**-1.6**
PLCB1	phospholipase C, beta 1 (phosphoinositide-specific)	**-1.5**	**-7.4**
PLCG1	phospholipase C, gamma 1	**1.9**	**1.9**
PLCG2	phospholipase C, gamma 2 (phosphatidylinositol-specific)	**-1.6**	**-1.3**
PLCL2	phospholipase C-like 2	**1.7**	**-1.6**
PLCXD1	phosphatidylinositol-specific phospholipase C, X domain containing 1	**-2.7**	**1.5**
PLD1	phospholipase D1, phosphatidylcholine-specific		**-1.3**
PLDN	pallidin homolog (mouse)		**-1.8**
PLEK	pleckstrin		**54.7**

PLEKHA2	pleckstrin homology domain containing, family A (phosphoinositide binding specific) member 2		**4.5**
PLEKHA5	pleckstrin homology domain containing, family A member 5	**1.8**	
PLEKHC1	pleckstrin homology domain containing, family C (with FERM domain) member 1	**3.1**	
PLEKHE1	PH domain and leucine rich repeat protein phosphatase		**-1.6**
PLEKHG2	pleckstrin homology domain containing, family G (with RhoGef domain) member 2	**1.5**	
PLK1	polo-like kinase 1 (Drosophila)	**1.5**	**-1.6**
PLK4	polo-like kinase 4 (Drosophila)	**1.7**	
PLOD1	procollagen-lysine 1, 2-oxoglutarate 5-dioxygenase 1	**-1.4**	**1.5**
PLOD2	procollagen-lysine, 2-oxoglutarate 5-dioxygenase 2		**3.5**
PLP2	proteolipid protein 2 (colonic epithelium-enriched)	**1.5**	**-3.3**
PLRG1	pleiotropic regulator 1 (PRL1 homolog, Arabidopsis)		**1.3**
PLS1	plastin 1 (I isoform)	**-3.2**	**9.1**
PLS3	plastin 3 (T isoform)		**15.8**
PLSCR1	phospholipid scramblase 1	**1.5**	**1.8**
PLTP	phospholipid transfer protein		**13.4**
PLXDC1	plexin domain containing 1	**1.5**	
PLXNA1	plexin A1		**-1.6**
PLXNB2	plexin B2	**1.3**	**-3.9**
PLXNC1	plexin C1		**-32.3**
PMAIP1	phorbol-12-myristate-13-acetate-induced protein 1	**1.5**	**-3.7**
PME-1	protein phosphatase methylesterase 1	**-1.4**	
PMF1	polyamine-modulated factor 1		**-1.6**
PML	promyelocytic leukemia		**1.8**
PMS1	PMS1 postmeiotic segregation increased 1 (S. cerevisiae)	**-1.3**	**2.5**
PMS2	PMS2 postmeiotic segregation increased 2 (S. cerevisiae)	**-1.3**	
PMS2L3	postmeiotic segregation increased 2-like 3		**-1.7**
PMVK	phosphomevalonate kinase		**-1.3**
PNMA1	paraneoplastic antigen MA1		**2.6**
PNN	pinin, desmosome associated protein	**-1.5**	**1.8**
PNPT1	polyribonucleotide nucleotidyltransferase 1	**-2.1**	
PNRC1	proline-rich nuclear receptor coactivator 1		**2.0**
PODXL	podocalyxin-like	**-2.8**	**-1.9**
POLA	polymerase (DNA directed), alpha 1	**-2.7**	**-1.5**
POLA2	polymerase (DNA directed), alpha 2 (70kD subunit)	**1.3**	**-1.4**
POLB	polymerase (DNA directed), beta	**-1.5**	**1.3**
POLD1	polymerase (DNA directed), delta 1, catalytic subunit 125kDa	**-1.4**	
POLD2	polymerase (DNA directed), delta 2, regulatory subunit 50kDa	**-1.9**	**1.9**
POLD3	polymerase (DNA-directed), delta 3, accessory subunit	**-1.8**	**-1.6**
POLDIP2	polymerase (DNA-directed), delta interacting protein 2	**-1.4**	
POLE	polymerase (DNA directed), epsilon		**-1.4**
POLE2	polymerase (DNA directed), epsilon 2 (p59 subunit)	**-2.1**	**-1.8**
POLE3	polymerase (DNA directed), epsilon 3 (p17 subunit)	**-2.0**	**-1.5**

POLG	polymerase (DNA directed), gamma	**1.4**	
POLG2	polymerase (DNA directed), gamma 2, accessory subunit	**-1.3**	**1.7**
POLH	polymerase (DNA directed), eta	**-1.4**	**-2.1**
POLI	polymerase (DNA directed) iota	**1.7**	**1.4**
POLQ	polymerase (DNA directed), theta	**-1.3**	
POLR1B	polymerase (RNA) I polypeptide B, 128kDa	**-2.7**	
POLR1C	polymerase (RNA) I polypeptide C, 30kDa	**-1.7**	
POLR1D	polymerase (RNA) I polypeptide D, 16kDa		**-1.9**
POLR2A	polymerase (RNA) II (DNA directed) polypeptide A, 220kDa		**-2.4**
POLR2B	polymerase (RNA) II (DNA directed) polypeptide B, 140kDa		**2.6**
POLR2C	polymerase (RNA) II (DNA directed) polypeptide C, 33kDa	**-1.4**	
POLR2E	polymerase (RNA) II (DNA directed) polypeptide E, 25kDa	**-1.9**	**-1.5**
POLR2G	polymerase (RNA) II (DNA directed) polypeptide G	**-1.4**	**-1.7**
POLR2H	polymerase (RNA) II (DNA directed) polypeptide H	**-1.9**	**1.4**
POLR2I	polymerase (RNA) II (DNA directed) polypeptide I, 14.5kDa	**-1.8**	**-1.6**
POLR2J	polymerase (RNA) II (DNA directed) polypeptide J, 13.3kDa		**-1.3**
POLR2J2	DNA directed RNA polymerase II polypeptide J-related gene		**1.8**
POLR2K	polymerase (RNA) II (DNA directed) polypeptide K, 7.0kDa		**-2.5**
POLR3B	polymerase (RNA) III (DNA directed) polypeptide B	**-1.9**	
POLR3C	polymerase (RNA) III (DNA directed) polypeptide C (62kD)		**-1.8**
POLR3E	polymerase (RNA) III (DNA directed) polypeptide E (80kD)	**-1.3**	**-1.7**
POLR3G	polymerase (RNA) III (DNA directed) polypeptide G (32kD)	**-3.0**	**1.3**
POLR3H	polymerase (RNA) III (DNA directed) polypeptide H (22.9kD)	**-2.0**	
POLR3K	polymerase (RNA) III (DNA directed) polypeptide K, 12.3 kDa	**-1.3**	**-3.0**
POP1	processing of precursor 1, ribonuclease P/MRP subunit (S. cerevisiae)		**-2.2**
POP4	processing of precursor 4, ribonuclease P/MRP subunit (S. cerevisiae)		**-1.3**
POP5	processing of precursor 5, ribonuclease P/MRP subunit (S. cerevisiae)	**-1.6**	
POP7	processing of precursor 7, ribonuclease P subunit (S. cerevisiae)	**-2.0**	
POR	P450 (cytochrome) oxidoreductase	**2.9**	
POT1	POT1 protection of telomeres 1 homolog (S. pombe)	**-1.3**	**-2.2**
POU2AF1	POU domain, class 2, associating factor 1		**-56.3**
POU2F1	POU domain, class 2, transcription factor 1	**-2.0**	
POU3F3	POU domain, class 3, transcription factor 3	**-1.7**	**-6.0**
POU4F1	POU domain, class 4, transcription factor 1	**-1.7**	
PPA1	pyrophosphatase (inorganic) 1	**-1.9**	**1.4**
PPA2	pyrophosphatase (inorganic) 2	**-1.3**	**2.1**
PPAP2B	phosphatidic acid phosphatase type 2B		**19.2**
PPARA	peroxisome proliferator-activated receptor alpha		**1.6**
PPARBP	PPAR binding protein	**-1.5**	**1.8**
PPAT	phosphoribosyl pyrophosphate amidotransferase	**-2.0**	**2.0**
PPFIA1	protein tyrosine phosphatase, receptor type, f polypeptide (PTPRF), interacting protein (liprin), alpha 1		**1.3**
PPFIBP1	PTPRF interacting protein, binding protein 1 (liprin beta 1)	**1.3**	**-1.8**

PPHLN1	periphilin 1	-3.5	
PPIA	peptidylprolyl isomerase A (cyclophilin A)	-1.2	1.9
PPIB	peptidylprolyl isomerase B (cyclophilin B)		1.5
PPIC	peptidylprolyl isomerase C (cyclophilin C)		3.4
PPIF	peptidylprolyl isomerase F (cyclophilin F)	-2.6	-1.3
PPIG	peptidylprolyl isomerase G (cyclophilin G)	-1.3	1.8
PPIH	peptidylprolyl isomerase H (cyclophilin H)	-1.7	
PPIL1	peptidylprolyl isomerase (cyclophilin)-like 1	-1.7	
PPIL4	peptidylprolyl isomerase (cyclophilin)-like 4		-1.6
PPIL5	peptidylprolyl isomerase (cyclophilin)-like 5		-1.3
PPM1A	protein phosphatase 1A (formerly 2C), magnesium-dependent, alpha isoform	1.5	-1.7
PPM1B	protein phosphatase 1B (formerly 2C), magnesium-dependent, beta isoform	1.4	1.3
PPM1F	protein phosphatase 1F (PP2C domain containing)		-2.1
PPM2C	protein phosphatase 2C, magnesium-dependent, catalytic subunit	1.5	-1.6
PPP1CA	protein phosphatase 1, catalytic subunit, alpha isoform		-2.1
PPP1CB	protein phosphatase 1, catalytic subunit, beta isoform	1.5	1.5
PPP1R10	protein phosphatase 1, regulatory subunit 10		1.4
PPP1R11	protein phosphatase 1, regulatory (inhibitor) subunit 11		-1.6
PPP1R12A	protein phosphatase 1, regulatory (inhibitor) subunit 12A	1.7	1.4
PPP1R14B	protein phosphatase 1, regulatory (inhibitor) subunit 14B	-1.8	-1.3
PPP1R16A	protein phosphatase 1, regulatory (inhibitor) subunit 16A	-2.3	
PPP1R2	protein phosphatase 1, regulatory (inhibitor) subunit 2	1.4	2.1
PPP1R3D	protein phosphatase 1, regulatory subunit 3D		2.1
PPP1R7	protein phosphatase 1, regulatory subunit 7		-2.5
PPP2CA	protein phosphatase 2 (formerly 2A), catalytic subunit, alpha isoform		-1.4
PPP2CB	protein phosphatase 2 (formerly 2A), catalytic subunit, beta isoform	1.2	1.5
PPP2R1A	protein phosphatase 2 (formerly 2A), regulatory subunit A (PR 65), alpha isoform	-1.4	
PPP2R1B	--	-1.4	-1.7
PPP2R2A	protein phosphatase 2 (formerly 2A), regulatory subunit B (PR 52), alpha isoform	1.7	2.4
PPP2R2B	protein phosphatase 2 (formerly 2A), regulatory subunit B (PR 52), beta isoform		1.4
PPP2R4	protein phosphatase 2A, regulatory subunit B' (PR 53)		-1.2
PPP2R5A	protein phosphatase 2, regulatory subunit B (B56), alpha isoform	1.9	-1.2
PPP2R5B	protein phosphatase 2, regulatory subunit B (B56), beta isoform	1.7	
PPP2R5C	protein phosphatase 2, regulatory subunit B (B56), gamma isoform		-25.3
PPP2R5E	protein phosphatase 2, regulatory subunit B (B56), epsilon isoform	1.2	2.4
PPP3CA	protein phosphatase 3 (formerly 2B), catalytic subunit, alpha isoform (calcineurin A alpha)	-1.3	1.9
PPP3CB	protein phosphatase 3 (formerly 2B), catalytic subunit, beta isoform (calcineurin A beta)	1.6	1.7
PPP3CC	protein phosphatase 3 (formerly 2B), catalytic subunit, gamma isoform (calcineurin A gamma)	1.8	
PPP4C	protein phosphatase 4 (formerly X), catalytic subunit		-1.8
PPP4R2	protein phosphatase 4, regulatory subunit 2	1.4	2.1
PPP6C	protein phosphatase 6, catalytic subunit		-1.8
PPRC1	peroxisome proliferator-activated receptor gamma, coactivator-related 1	-2.4	1.7

PQBP1	polyglutamine binding protein 1	**1.3**	**1.3**
PRC1	protein regulator of cytokinesis 1	**1.9**	**-1.6**
PRDM1	PR domain containing 1, with ZNF domain	**2.3**	
PRDX1	peroxiredoxin 1	**-1.7**	**-2.5**
PRDX2	peroxiredoxin 2	**-3.1**	**-8.5**
PRDX3	peroxiredoxin 3	**-1.8**	
PRDX4	peroxiredoxin 4	**-2.5**	**1.3**
PRDX5	peroxiredoxin 5	**-1.6**	**1.2**
PRDX6	peroxiredoxin 6	**1.4**	**1.5**
PREB	prolactin regulatory element binding	**-1.3**	**1.7**
PREI3	preimplantation protein 3	**1.3**	**1.5**
PREP	prolyl endopeptidase		**1.5**
PRES	solute carrier family 26, member 5 (prestin)		**-1.2**
PRG1	proteoglycan 1, secretory granule	**1.5**	**2.7**
PRG4	proteoglycan 4	**-1.8**	**1.7**
PRIM1	primase, polypeptide 1, 49kDa	**-1.8**	**-1.3**
PRIM2A	primase, polypeptide 2A, 58kDa		**1.4**
PRKAA1	protein kinase, AMP-activated, alpha 1 catalytic subunit	**1.6**	**1.3**
PRKAB1	protein kinase, AMP-activated, beta 1 non-catalytic subunit		**1.5**
PRKAB2	protein kinase, AMP-activated, beta 2 non-catalytic subunit		**5.1**
PRKACB	protein kinase, cAMP-dependent, catalytic, beta	**-2.7**	**2.4**
PRKAG1	protein kinase, AMP-activated, gamma 1 non-catalytic subunit		**1.2**
PRKAG2	protein kinase, AMP-activated, gamma 2 non-catalytic subunit	**4.0**	**2.1**
PRKAR1A	protein kinase, cAMP-dependent, regulatory, type I, alpha (tissue specific extinguisher 1)	**1.4**	**2.0**
PRKAR2A	protein kinase, cAMP-dependent, regulatory, type II, alpha	**1.3**	**8.5**
PRKCA	protein kinase C, alpha	**1.3**	**6.6**
PRKCB1	protein kinase C, beta 1	**1.6**	**20.7**
PRKCBP1	protein kinase C binding protein 1	**1.4**	**-1.2**
PRKCE	protein kinase C, epsilon	**-1.3**	**-61.5**
PRKCH	protein kinase C, eta	**3.7**	**3.4**
PRKCQ	protein kinase C, theta		**1.6**
PRKCSH	--		**1.3**
PRKD2	protein kinase D2	**1.8**	**-3.2**
PRKDC	protein kinase, DNA-activated, catalytic polypeptide	**-1.6**	**-1.2**
PRKRA	protein kinase, interferon-inducible double stranded RNA dependent activator	**2.5**	**-1.9**
PRKRIR	protein-kinase, interferon-inducible double stranded RNA dependent inhibitor, repressor of (P58 repressor)	**-1.6**	
PRKX	protein kinase, X-linked	**3.4**	**-2.3**
PRL	prolactin		**-72.9**
PRNP	prion protein (p27-30) (Creutzfeldt-Jakob disease, Gerstmann-Strausler-Scheinker syndrome, fatal familial insomnia)		**-1.4**
PROCR	protein C receptor, endothelial (EPCR)	**-1.5**	**6.6**
PROS1	protein S (alpha)	**1.8**	**4.8**
PRPF4	PRP4 pre-mRNA processing factor 4 homolog (yeast)	**-2.2**	**-1.3**

PRPF8	PRP8 pre-mRNA processing factor 8 homolog (S. cerevisiae)	**-1.3**	**-1.4**
PRPS1	phosphoribosyl pyrophosphate synthetase 1	**-2.6**	**-2.1**
PRPS2	phosphoribosyl pyrophosphate synthetase 2		**1.3**
PRPSAP1	phosphoribosyl pyrophosphate synthetase-associated protein 1		**1.9**
PRPSAP2	phosphoribosyl pyrophosphate synthetase-associated protein 2		**-1.8**
PRSS25	HtrA serine peptidase 2	**-1.5**	**1.5**
PRTFDC1	phosphoribosyl transferase domain containing 1		**3.8**
PRTN3	proteinase 3 (serine proteinase, neutrophil, Wegener granulomatosis autoantigen)		**434.0**
PRUNE	prune homolog (Drosophila)	**-1.9**	**-1.5**
PRX	periaxin	**34.6**	
PSAP	prosaposin (variant Gaucher disease and variant metachromatic leukodystrophy)	**-1.4**	**3.1**
PSAT1	phosphoserine aminotransferase 1	**-2.6**	**-1.5**
PSCD2	pleckstrin homology, Sec7 and coiled-coil domains 2 (cytohesin-2)	**1.4**	
PSCD3	pleckstrin homology, Sec7 and coiled-coil domains 3	**4.1**	**1.7**
PSCDBP	pleckstrin homology, Sec7 and coiled-coil domains, binding protein	**-1.5**	**-1.6**
PSEN1	presenilin 1 (Alzheimer disease 3)	**1.5**	**1.3**
PSENEN	presenilin enhancer 2 homolog (C. elegans)	**1.8**	
PSF1	GINS complex subunit 1 (Psf1 homolog)	**-1.6**	**-1.9**
PSIP1	PC4 and SFRS1 interacting protein 1	**-1.5**	**-2.0**
PSMA1	proteasome (prosome, macropain) subunit, alpha type, 1		**-1.2**
PSMA2	proteasome (prosome, macropain) subunit, alpha type, 2	**-1.3**	**3.5**
PSMA3	proteasome (prosome, macropain) subunit, alpha type, 3	**-1.7**	**1.3**
PSMA4	proteasome (prosome, macropain) subunit, alpha type, 4	**-1.3**	
PSMA5	proteasome (prosome, macropain) subunit, alpha type, 5	**-1.4**	
PSMA6	proteasome (prosome, macropain) subunit, alpha type, 6		**-1.3**
PSMA7	proteasome (prosome, macropain) subunit, alpha type, 7	**1.7**	**-1.4**
PSMB10	proteasome (prosome, macropain) subunit, beta type, 10		**-3.7**
PSMB2	proteasome (prosome, macropain) subunit, beta type, 2	**-1.6**	**-1.2**
PSMB3	proteasome (prosome, macropain) subunit, beta type, 3	**-1.2**	
PSMB4	proteasome (prosome, macropain) subunit, beta type, 4	**-1.4**	**1.3**
PSMB5	proteasome (prosome, macropain) subunit, beta type, 5	**-1.4**	**1.5**
PSMB6	proteasome (prosome, macropain) subunit, beta type, 6		**-1.6**
PSMB8	proteasome (prosome, macropain) subunit, beta type, 8 (large multifunctional peptidase 7)		**-18.9**
PSMB9	proteasome (prosome, macropain) subunit, beta type, 9 (large multifunctional peptidase 2)	**-1.3**	**-202.7**
PSMC3	proteasome (prosome, macropain) 26S subunit, ATPase, 3		**-1.4**
PSMC4	proteasome (prosome, macropain) 26S subunit, ATPase, 4	**-1.3**	
PSMC5	proteasome (prosome, macropain) 26S subunit, ATPase, 5		**1.6**
PSMC6	proteasome (prosome, macropain) 26S subunit, ATPase, 6		**1.3**
PSMD1	proteasome (prosome, macropain) 26S subunit, non-ATPase, 1	**-1.4**	**-1.6**
PSMD10	proteasome (prosome, macropain) 26S subunit, non-ATPase, 10		**-1.2**
PSMD12	proteasome (prosome, macropain) 26S subunit, non-ATPase, 12		**1.6**
PSMD2	proteasome (prosome, macropain) 26S subunit, non-ATPase, 2		**1.3**

PSMD3	proteasome (prosome, macropain) 26S subunit, non-ATPase, 3	**-1.5**	
PSMD4	proteasome (prosome, macropain) 26S subunit, non-ATPase, 4		**1.3**
PSMD5	proteasome (prosome, macropain) 26S subunit, non-ATPase, 5		**3.0**
PSMD6	proteasome (prosome, macropain) 26S subunit, non-ATPase, 6	**-1.2**	
PSMD8	proteasome (prosome, macropain) 26S subunit, non-ATPase, 8	**-1.3**	**-1.5**
PSME1	proteasome (prosome, macropain) activator subunit 1 (PA28 alpha)	**-1.8**	**-1.9**
PSME2	proteasome (prosome, macropain) activator subunit 2 (PA28 beta)	**-1.8**	**-2.4**
PSME3	proteasome (prosome, macropain) activator subunit 3 (PA28 gamma; Ki)	**-2.2**	**1.5**
PSPC1	paraspeckle component 1	**1.5**	**-2.2**
PSPH	phosphoserine phosphatase	**-2.0**	**3.3**
PTBP1	polypyrimidine tract binding protein 1	**-1.5**	
PTBP2	polypyrimidine tract binding protein 2	**1.5**	
PTCH	patched homolog 1 (Drosophila)	**-2.7**	**-3.1**
PTDSR	phosphatidylserine receptor	**1.4**	
PTEN	phosphatase and tensin homolog (mutated in multiple advanced cancers 1)	**-1.3**	**4.2**
PTGER2	prostaglandin E receptor 2 (subtype EP2), 53kDa		**4.8**
PTGER3	prostaglandin E receptor 3 (subtype EP3)	**1.7**	
PTGER4	prostaglandin E receptor 4 (subtype EP4)	**-2.2**	**-4.6**
PTGFRN	prostaglandin F2 receptor negative regulator	**-8.2**	
PTGS1	prostaglandin-endoperoxide synthase 1 (prostaglandin G/H synthase and cyclooxygenase)		**2.8**
PTHLH	parathyroid hormone-like hormone	**29.4**	
PTHR2	parathyroid hormone receptor 2		**17.2**
PTK2	PTK2 protein tyrosine kinase 2	**1.5**	**2.2**
PTK2B	PTK2B protein tyrosine kinase 2 beta		**-1.3**
PTMA	prothymosin, alpha (gene sequence 28)	**-1.3**	**-1.7**
PTOV1	prostate tumor overexpressed gene 1	**-1.8**	
PTP4A1	protein tyrosine phosphatase type IVA, member 1	**-1.8**	**-1.7**
PTP4A2	protein tyrosine phosphatase type IVA, member 2	**1.5**	**1.2**
PTP4A3	protein tyrosine phosphatase type IVA, member 3	**6.6**	
PTPLB	protein tyrosine phosphatase-like (proline instead of catalytic arginine), member b	**2.3**	**2.2**
PTPN11	protein tyrosine phosphatase, non-receptor type 11 (Noonan syndrome 1)		**1.3**
PTPN12	protein tyrosine phosphatase, non-receptor type 12	**2.4**	**-10.9**
PTPN2	protein tyrosine phosphatase, non-receptor type 2	**-1.2**	**1.3**
PTPN22	protein tyrosine phosphatase, non-receptor type 22 (lymphoid)		**2.9**
PTPN4	protein tyrosine phosphatase, non-receptor type 4 (megakaryocyte)	**-1.6**	**-1.6**
PTPN6	protein tyrosine phosphatase, non-receptor type 6		**-5.5**
PTPN7	protein tyrosine phosphatase, non-receptor type 7	**-1.5**	**3.3**
PTPNS1	signal-regulatory protein alpha		**12.6**
PTPRA	protein tyrosine phosphatase, receptor type, A		**1.3**
PTPRB	protein tyrosine phosphatase, receptor type, B	**2.2**	**-7.2**
PTPRC	protein tyrosine phosphatase, receptor type, C		**-6.4**
PTPRCAP	protein tyrosine phosphatase, receptor type, C-associated protein	**1.5**	**-2.1**

PTPRE	protein tyrosine phosphatase, receptor type, E	**2.1**	**2.2**
PTPRF	protein tyrosine phosphatase, receptor type, F		**91.0**
PTPRG	protein tyrosine phosphatase, receptor type, G	**6.8**	**39.8**
PTPRK	protein tyrosine phosphatase, receptor type, K	**-2.9**	**2.0**
PTPRM	protein tyrosine phosphatase, receptor type, M		**8.5**
PTPRR	protein tyrosine phosphatase, receptor type, R		**-226.8**
PTS	6-pyruvoyltetrahydropterin synthase	**-1.3**	**-2.4**
PTTG1	pituitary tumor-transforming 1		**-2.1**
PTTG1IP	pituitary tumor-transforming 1 interacting protein	**1.5**	**1.4**
PTX3	pentraxin-related gene, rapidly induced by IL-1 beta	**1.5**	**1.7**
PUM2	pumilio homolog 2 (Drosophila)		**-1.4**
PUNC	putative neuronal cell adhesion molecule	**-1.5**	
PURA	purine-rich element binding protein A		**-1.3**
PURB	purine-rich element binding protein B		**7.3**
PUS3	pseudouridylate synthase 3	**-1.4**	
PXDN	peroxidasin homolog (Drosophila)	**2.0**	
PXMP3	peroxisomal membrane protein 3, 35kDa (Zellweger syndrome)	**1.7**	**-1.7**
PXN	paxillin		**7.1**
PYCARD	PYD and CARD domain containing		**-3.0**
PYCR1	pyrroline-5-carboxylate reductase 1	**-2.8**	**-8.7**
PYCR2	pyrroline-5-carboxylate reductase family, member 2	**-1.7**	
PYGL	phosphorylase, glycogen; liver (Hers disease, glycogen storage disease type VI)		**48.6**
QDPR	quinoid dihydropteridine reductase		**2.2**
QKI	quaking homolog, KH domain RNA binding (mouse)	**2.5**	**2.7**
QP-C	ubiquinol-cytochrome c reductase, complex III subunit VII, 9.5kDa	**-1.2**	
QPRT	quinolinate phosphoribosyltransferase (nicotinate-nucleotide pyrophosphorylase (carboxylating))	**-3.6**	**-2.1**
RAB11A	RAB11A, member RAS oncogene family	**1.3**	**-2.2**
RAB11B	RAB11B, member RAS oncogene family		**-1.4**
RAB11FIP1	RAB11 family interacting protein 1 (class I)	**2.0**	**3.7**
RAB11FIP2	RAB11 family interacting protein 2 (class I)	**-2.0**	
RAB11FIP3	RAB11 family interacting protein 3 (class II)		**-5.1**
RAB14	RAB14, member RAS oncogene family	**1.3**	**1.3**
RAB1A	RAB1A, member RAS oncogene family	**1.3**	**1.5**
RAB1B	RAB1B, member RAS oncogene family		**-1.3**
RAB2	RAB2, member RAS oncogene family		**-1.9**
RAB20	RAB20, member RAS oncogene family		**13.4**
RAB27A	RAB27A, member RAS oncogene family	**-1.8**	**2.2**
RAB27B	RAB27B, member RAS oncogene family		**70.0**
RAB31	RAB31, member RAS oncogene family		**3.8**
RAB33A	RAB33A, member RAS oncogene family		**6.8**
RAB3GAP	RAB3 GTPase activating protein subunit 1 (catalytic)	**-1.5**	**1.8**
RAB3-GAP150	RAB3 GTPase activating protein subunit 2 (non-catalytic)		**1.3**

RAB3IP	RAB3A interacting protein (rabin3)	**1.5**	
RAB4A	RAB4A, member RAS oncogene family		**3.8**
RAB5A	RAB5A, member RAS oncogene family	**1.3**	**1.2**
RAB5C	RAB5C, member RAS oncogene family		**1.2**
RAB6A	RAB6A, member RAS oncogene family	**1.5**	**-1.4**
RAB6IP2	ELKS/RAB6-interacting/CAST family member 1		**-1.3**
RAB7	RAB7, member RAS oncogene family		**1.9**
RAB7L1	RAB7, member RAS oncogene family-like 1	**-1.4**	**2.1**
RAB9A	RAB9, member RAS oncogene family		**1.3**
RAB9P40	Rab9 effector protein with kelch motifs	**-3.7**	**1.9**
RABAC1	Rab acceptor 1 (prenylated)		**-1.5**
RABEP1	rabaptin, RAB GTPase binding effector protein 1		**-2.1**
RABEP2	rabaptin, RAB GTPase binding effector protein 2		**-2.5**
RABGEF1	RAB guanine nucleotide exchange factor (GEF) 1	**1.3**	
RABGGTA	Rab geranylgeranyltransferase, alpha subunit	**-1.2**	
RABGGTB	Rab geranylgeranyltransferase, beta subunit	**-1.8**	**2.1**
RABIF	RAB interacting factor		**-1.2**
RABL2B	RAB, member of RAS oncogene family-like 2B		**-1.4**
RAC1	ras-related C3 botulinum toxin substrate 1 (rho family, small GTP binding protein Rac1)	**1.4**	**-1.9**
RAC2	ras-related C3 botulinum toxin substrate 2 (rho family, small GTP binding protein Rac2)		**-2.7**
RACGAP1	Rac GTPase activating protein 1	**1.5**	
RAD1	RAD1 homolog (S. pombe)	**-1.7**	**-1.6**
RAD17	RAD17 homolog (S. pombe)	**-2.1**	
RAD18	RAD18 homolog (S. cerevisiae)		**-1.4**
RAD21	RAD21 homolog (S. pombe)	**1.3**	**-2.3**
RAD23A	RAD23 homolog A (S. cerevisiae)	**-1.4**	
RAD23B	RAD23 homolog B (S. cerevisiae)		**-1.3**
RAD50	RAD50 homolog (S. cerevisiae)		**-2.1**
RAD51	RAD51 homolog (RecA homolog, E. coli) (S. cerevisiae)		**-3.5**
RAD51AP1	RAD51 associated protein 1	**-1.4**	**-2.8**
RAD51C	RAD51 homolog C (S. cerevisiae)	**-1.9**	
RAD51L3	RAD51-like 3 (S. cerevisiae)	**-1.7**	
RAD52	RAD52 homolog (S. cerevisiae)	**1.5**	**-1.6**
RAD54B	RAD54 homolog B (S. cerevisiae)	**-3.5**	**-2.1**
RAD54L	RAD54-like (S. cerevisiae)	**-1.5**	
RAD9A	RAD9 homolog A (S. pombe)	**-1.6**	**-1.7**
RAE1	RAE1 RNA export 1 homolog (S. pombe)	**-1.4**	**-1.3**
RAF1	v-raf-1 murine leukemia viral oncogene homolog 1		**-1.3**
RAFTLIN	raftlin, lipid raft linker 1	**-1.5**	**-1.5**
RAG1	recombination activating gene 1		**-32.2**
RAG2	recombination activating gene 2		**-7.4**
RAI17	zinc finger, MIZ-type containing 1	**-1.3**	

RALA	v-ral simian leukemia viral oncogene homolog A (ras related)	**-1.3**	**1.7**
RALB	v-ral simian leukemia viral oncogene homolog B (ras related; GTP binding protein)	**1.9**	**1.5**
RALBP1	ralA binding protein 1	**-1.4**	**-1.8**
RALY	RNA binding protein, autoantigenic (hnRNP-associated with lethal yellow homolog (mouse))		**-1.4**
RAM2	cell division cycle associated 7-like	**1.4**	**2.9**
RAMP	denticleless homolog (Drosophila)	**-1.8**	**-1.5**
RAN	RAN, member RAS oncogene family	**-1.2**	**-1.2**
RANBP1	RAN binding protein 1		**-1.9**
RANBP2	RAN binding protein 2	**-1.4**	**2.5**
RANBP5	RAN binding protein 5	**-2.0**	**-1.3**
RANBP9	RAN binding protein 9	**-1.3**	**-1.6**
RANGAP1	--	**-1.4**	
RAP1A	RAP1A, member of RAS oncogene family		**2.0**
RAP1B	RAP1B, member of RAS oncogene family	**1.3**	
RAP1GDS1	RAP1, GTP-GDP dissociation stimulator 1		**-1.5**
RAP2A	RAP2A, member of RAS oncogene family	**1.6**	**-1.4**
RAPGEF1	Rap guanine nucleotide exchange factor (GEF) 1	**2.5**	**-1.5**
RAPGEF2	Rap guanine nucleotide exchange factor (GEF) 2	**2.0**	**1.8**
RAPGEF4	Rap guanine nucleotide exchange factor (GEF) 4	**-3.9**	
RAPGEF5	Rap guanine nucleotide exchange factor (GEF) 5	**3.0**	**-8.6**
RAPTOR	raptor		**2.9**
RARRES1	retinoic acid receptor responder (tazarotene induced) 1		**1.4**
RARSL	arginyl-tRNA synthetase-like	**1.3**	**1.2**
RASA1	RAS p21 protein activator (GTPase activating protein) 1	**2.3**	**1.9**
RASA2	RAS p21 protein activator 2	**1.6**	
RASA3	RAS p21 protein activator 3	**3.9**	**-2.6**
RASA4	RAS p21 protein activator 4		**2.0**
RASAL2	RAS protein activator like 2	**8.3**	
RASD1	RAS, dexamethasone-induced 1	**1.8**	
RASGRP1	RAS guanyl releasing protein 1 (calcium and DAG-regulated)	**-7.2**	**-111.1**
RASGRP2	RAS guanyl releasing protein 2 (calcium and DAG-regulated)	**2.1**	**-2.3**
RASSF2	Ras association (RalGDS/AF-6) domain family 2	**-2.5**	**-6.4**
RASSF4	Ras association (RalGDS/AF-6) domain family 4	**14.4**	**13.4**
RASSF5	Ras association (RalGDS/AF-6) domain family 5	**-1.5**	**-4.4**
RAVER1	ribonucleoprotein, PTB-binding 1	**-1.5**	**-1.3**
RB1	retinoblastoma 1 (including osteosarcoma)	**1.5**	**-2.6**
RB1CC1	RB1-inducible coiled-coil 1	**1.7**	**-1.4**
RBBP4	retinoblastoma binding protein 4	**-1.4**	**-1.3**
RBBP5	retinoblastoma binding protein 5		**-1.3**
RBBP6	retinoblastoma binding protein 6		**-1.8**
RBBP7	retinoblastoma binding protein 7	**-1.6**	**-1.3**
RBBP8	retinoblastoma binding protein 8	**-1.7**	**-1.9**

RBBP9	retinoblastoma binding protein 9	**-1.3**	
RBKS	ribokinase		**1.5**
RBL1	retinoblastoma-like 1 (p107)		**-1.8**
RBL2	retinoblastoma-like 2 (p130)	**1.5**	**1.5**
RBM10	RNA binding motif protein 10	**-1.3**	
RBM13	RNA binding motif protein 13	**-1.8**	
RBM17	RNA binding motif protein 17	**-1.2**	**2.0**
RBM25	RNA binding motif protein 25	**1.3**	**-1.3**
RBM4	RNA binding motif protein 4	**1.3**	**1.7**
RBM5	RNA binding motif protein 5		**1.6**
RBM8A	RNA binding motif protein 8A	**1.5**	**2.3**
RBM9	RNA binding motif protein 9	**-1.3**	**8.5**
RBMS1	RNA binding motif, single stranded interacting protein 1	**4.3**	**3.0**
RBMX	RNA binding motif protein, X-linked		**1.4**
RBMY1A1	RNA binding motif protein, Y-linked, family 1, member A1		**-2.5**
RBPMS	RNA binding protein with multiple splicing	**1.7**	**8.0**
RBPSUH	recombining binding protein suppressor of hairless (Drosophila)	**1.4**	**1.9**
RBX1	ring-box 1		**-1.4**
RCHY1	ring finger and CHY zinc finger domain containing 1		**1.5**
RCN1	reticulocalbin 1, EF-hand calcium binding domain	**1.2**	**-1.5**
RCN2	reticulocalbin 2, EF-hand calcium binding domain	**2.0**	**1.4**
RCOR1	REST corepressor 1		**-1.3**
RCP9	calcitonin gene-related peptide-receptor component protein	**-1.5**	**-1.5**
RDBP	RD RNA binding protein		**-1.2**
RDH11	retinol dehydrogenase 11 (all-trans/9-cis/11-cis)	**-1.8**	**-1.3**
RECK	reversion-inducing-cysteine-rich protein with kazal motifs	**2.9**	
RECQL	RecQ protein-like (DNA helicase Q1-like)		**-1.4**
RECQL5	RecQ protein-like 5		**2.3**
REL	v-rel reticuloendotheliosis viral oncogene homolog (avian)	**1.5**	**2.4**
REPS1	RALBP1 associated Eps domain containing 1		**1.3**
RET	ret proto-oncogene (multiple endocrine neoplasia and medullary thyroid carcinoma 1, Hirschsprung disease)		**8.3**
REV1L	REV1 homolog (S. cerevisiae)		**1.8**
REV3L	REV3-like, catalytic subunit of DNA polymerase zeta (yeast)	**1.5**	**-2.1**
RFC1	replication factor C (activator 1) 1, 145kDa	**-1.4**	**-1.2**
RFC2	replication factor C (activator 1) 2, 40kDa	**-1.5**	**-3.9**
RFC3	replication factor C (activator 1) 3, 38kDa	**-1.5**	**-2.1**
RFC4	replication factor C (activator 1) 4, 37kDa	**-1.6**	
RFC5	replication factor C (activator 1) 5, 36.5kDa	**-1.7**	**-1.3**
RFK	riboflavin kinase	**-2.1**	**-1.9**
RFP	tripartite motif-containing 27	**-1.5**	
RFWD2	ring finger and WD repeat domain 2		**-2.1**
RFX3	regulatory factor X, 3 (influences HLA class II expression)	**1.9**	

RFX5	regulatory factor X, 5 (influences HLA class II expression)		**-1.6**
RFXAP	regulatory factor X-associated protein	**-1.7**	**-1.6**
RGC32	response gene to complement 32	**3.0**	**6.1**
RGL1	ral guanine nucleotide dissociation stimulator-like 1	**2.6**	**-2.2**
RGS10	regulator of G-protein signalling 10	**-2.5**	**19.3**
RGS14	regulator of G-protein signalling 14	**-1.3**	
RGS18	regulator of G-protein signalling 18		**6.1**
RGS19	regulator of G-protein signalling 19	**-1.3**	**-1.8**
RHEB	Ras homolog enriched in brain	**1.9**	**-1.6**
RHOA	ras homolog gene family, member A	**1.4**	**-1.4**
RHOB	ras homolog gene family, member B	**4.9**	**-6.3**
RHOC	ras homolog gene family, member C	**3.2**	**3.0**
RHOF	ras homolog gene family, member F (in filopodia)		**4.2**
RHOG	ras homolog gene family, member G (rho G)		**1.9**
RHOH	ras homolog gene family, member H	**1.3**	**-15.8**
RHOQ	ras homolog gene family, member Q	**1.3**	**1.8**
RHOT1	ras homolog gene family, member T1		**1.8**
RHOU	ras homolog gene family, member U		**12.1**
RHPN1	rhophilin, Rho GTPase binding protein 1	**1.6**	
RICS	Rho GTPase-activating protein		**-3.3**
RIMS3	regulating synaptic membrane exocytosis 3		**-6.8**
RIN1	Ras and Rab interactor 1	**-1.3**	**-1.3**
RIN2	Ras and Rab interactor 2	**2.0**	
RIPK1	receptor (TNFRSF)-interacting serine-threonine kinase 1		**1.5**
RIT1	Ras-like without CAAX 1	**1.8**	**-1.4**
RNASE2	ribonuclease, RNase A family, 2 (liver, eosinophil-derived neurotoxin)		**13.8**
RNASE6	ribonuclease, RNase A family, k6	**17.2**	
RNASEH1	ribonuclease H1	**-1.3**	
RNASEL	--		**-2.0**
RNF103	ring finger protein 103	**1.5**	
RNF11	ring finger protein 11	**1.5**	
RNF12	ring finger protein 12	**-1.3**	**1.3**
RNF13	ring finger protein 13	**-1.5**	**1.3**
RNF139	ring finger protein 139		**1.4**
RNF14	ring finger protein 14	**1.4**	
RNF144	ring finger protein 144		**3.5**
RNF19	ring finger protein 19	**1.9**	**-1.9**
RNF36	tripartite motif-containing 69		**-2.3**
RNF41	ring finger protein 41	**-1.6**	
RNF6	ring finger protein (C3H2C3 type) 6	**1.2**	**-2.2**
RNF7	ring finger protein 7	**1.6**	**1.4**
RNGTT	RNA guanylyltransferase and 5'-phosphatase		**-1.6**

RNH	ribonuclease/angiogenin inhibitor 1	**-1.5**	**2.4**
RNMT	RNA (guanine-7-) methyltransferase		**1.3**
RNPC1	RNA binding motif protein 38	**1.4**	**-1.9**
RNPC2	RNA binding motif protein 39	**2.3**	**1.8**
RNPS1	RNA binding protein S1, serine-rich domain	**-1.2**	**-5.0**
RNUT1	snurportin 1		**1.3**
ROBO1	roundabout, axon guidance receptor, homolog 1 (Drosophila)	**-3.4**	**12.5**
ROCK2	Rho-associated, coiled-coil containing protein kinase 2	**1.3**	
RP2	retinitis pigmentosa 2 (X-linked recessive)	**1.2**	
RP9	retinitis pigmentosa 9 (autosomal dominant)	**-1.7**	**2.9**
RPA1	replication protein A1, 70kDa	**-1.6**	**-2.8**
RPA2	replication protein A2, 32kDa	**-1.4**	
RPA3	replication protein A3, 14kDa		**-2.0**
RPE	ribulose-5-phosphate-3-epimerase	**-1.5**	**-2.1**
RPGR	retinitis pigmentosa GTPase regulator		**6.5**
RPIA	ribose 5-phosphate isomerase A (ribose 5-phosphate epimerase)	**-1.7**	
RPL12	ribosomal protein L12		**1.5**
RPL13	ribosomal protein L13		**1.4**
RPL13A	ribosomal protein L13a		**1.4**
RPL15	ribosomal protein L15	**2.1**	**1.5**
RPL17	ribosomal protein L17	**-1.8**	**2.1**
RPL18	ribosomal protein L18	**2.4**	
RPL18A	ribosomal protein L18a	**1.3**	**1.4**
RPL22	ribosomal protein L22		**1.2**
RPL23	ribosomal protein L23	**-1.3**	**1.3**
RPL26	ribosomal protein L26	**1.2**	
RPL29	ribosomal protein L29	**1.4**	**2.0**
RPL3	ribosomal protein L3		**1.3**
RPL31	ribosomal protein L31	**1.3**	**2.9**
RPL35	ribosomal protein L35	**2.4**	**2.2**
RPL35A	ribosomal protein L35a		**1.6**
RPL37	ribosomal protein L37	**-1.5**	**1.6**
RPL38	ribosomal protein L38		**2.0**
RPL4	ribosomal protein L4		**1.3**
RPL5	ribosomal protein L5	**1.2**	**1.3**
RPL7A	ribosomal protein L7a		**1.7**
RPLP2	ribosomal protein, large, P2		**1.4**
RPN1	ribophorin I		**1.5**
RPN2	ribophorin II	**-1.5**	**1.7**
RPP14	ribonuclease P 14kDa subunit	**-1.7**	**1.9**
RPP30	ribonuclease P/MRP 30kDa subunit	**-1.6**	
RPP38	ribonuclease P/MRP 38kDa subunit	**-1.5**	**1.7**

RPP40	ribonuclease P 40kDa subunit	**-1.9**	
RPS10	ribosomal protein S10	**1.4**	**1.3**
RPS14	ribosomal protein S14	**1.6**	**1.5**
RPS15A	ribosomal protein S15a		**1.5**
RPS17	ribosomal protein S17	**1.5**	**1.5**
RPS18	ribosomal protein S18		**2.2**
RPS19	ribosomal protein S19	**-1.6**	**1.3**
RPS2	ribosomal protein S2		**2.6**
RPS23	ribosomal protein S23		**2.0**
RPS24	ribosomal protein S24		**2.2**
RPS25	ribosomal protein S25	**1.3**	
RPS28	ribosomal protein S28		**1.2**
RPS3	ribosomal protein S3		**1.2**
RPS6	ribosomal protein S6	**-1.9**	**-1.7**
RPS6KA1	ribosomal protein S6 kinase, 90kDa, polypeptide 1	**-1.3**	**-2.9**
RPS6KA2	ribosomal protein S6 kinase, 90kDa, polypeptide 2	**6.0**	**7.9**
RPS6KA3	ribosomal protein S6 kinase, 90kDa, polypeptide 3	**-1.5**	
RPS6KA4	ribosomal protein S6 kinase, 90kDa, polypeptide 4	**-1.3**	
RPS6KB1	ribosomal protein S6 kinase, 70kDa, polypeptide 1		**1.7**
RPS8	ribosomal protein S8		**1.3**
RPS9	ribosomal protein S9	**2.5**	
RPUSD4	RNA pseudouridylate synthase domain containing 4	**-1.6**	
RQCD1	RCD1 required for cell differentiation1 homolog (S. pombe)	**-1.7**	**-2.3**
RRAGA	Ras-related GTP binding A	**1.3**	**-2.6**
RRAGC	Ras-related GTP binding C	**1.5**	
RRAGD	Ras-related GTP binding D	**-2.4**	**3.0**
RRAS	related RAS viral (r-ras) oncogene homolog	**2.6**	**1.7**
RRAS2	related RAS viral (r-ras) oncogene homolog 2		**-16.2**
RRM1	ribonucleotide reductase M1 polypeptide	**-1.4**	**-1.6**
RRM2	ribonucleotide reductase M2 polypeptide		**-2.3**
RRM2B	ribonucleotide reductase M2 B (TP53 inducible)		**-5.8**
RRN3	RRN3 RNA polymerase I transcription factor homolog (S. cerevisiae)	**-2.7**	**-1.9**
RRS1	RRS1 ribosome biogenesis regulator homolog (S. cerevisiae)		**-1.4**
RSAD2	radical S-adenosyl methionine domain containing 2		**1.9**
RSAFD1	tRNA-yW synthesizing protein 1 homolog (S. cerevisiae)	**-1.3**	
RSF1	remodeling and spacing factor 1		**3.9**
RTN1	reticulon 1		**2.6**
RTN4	reticulon 4	**2.3**	
RTN4IP1	reticulon 4 interacting protein 1		**-1.4**
RTN4R	reticulon 4 receptor	**-4.1**	**-2.6**
RUFY1	RUN and FYVE domain containing 1	**-1.3**	**1.3**
RUFY2	RUN and FYVE domain containing 2	**1.8**	**-1.5**

RUNX1	--	**-2.5**	**-1.5**
RUNX1T1	runt-related transcription factor 1; translocated to, 1 (cyclin D-related)		**144.2**
RUNX2	runt-related transcription factor 2	**12.3**	**2.2**
RUNX3	runt-related transcription factor 3		**9.2**
RUVBL1	RuvB-like 1 (E. coli)		**-1.6**
RUVBL2	RuvB-like 2 (E. coli)	**-1.4**	**1.2**
RW1	transmembrane protein 131	**1.3**	**1.4**
RXRA	retinoid X receptor, alpha		**2.4**
RYBP	RING1 and YY1 binding protein	**1.3**	**1.4**
RYK	RYK receptor-like tyrosine kinase		**11.1**
RYR3	ryanodine receptor 3		**-1.5**
S100A1	S100 calcium binding protein A1	**-1.9**	
S100A10	S100 calcium binding protein A10		**-26.4**
S100A11	S100 calcium binding protein A11		**1.5**
S100A13	S100 calcium binding protein A13	**-1.7**	
S100A16	S100 calcium binding protein A16		**-2.3**
S100A4	S100 calcium binding protein A4	**-4.0**	**-7.7**
S100A6	S100 calcium binding protein A6		**-11.2**
S100P	S100 calcium binding protein P		**8.7**
S100Z	S100 calcium binding protein Z		**4.7**
SAE1	SUMO1 activating enzyme subunit 1	**-1.3**	**-1.3**
SAFB2	scaffold attachment factor B2	**1.4**	
SAMD4	sterile alpha motif domain containing 4A	**-1.7**	
SAMSN1	SAM domain, SH3 domain and nuclear localization signals 1		**48.7**
SAP18	Sin3A-associated protein, 18kDa		**-2.2**
SAP30	Sin3A-associated protein, 30kDa	**2.3**	**1.7**
SAPS3	SAPS domain family, member 3	**2.8**	**-1.3**
SARA1	SAR1 gene homolog A (S. cerevisiae)		**1.6**
SARA2	SAR1 gene homolog B (S. cerevisiae)		**1.3**
SARS	seryl-tRNA synthetase	**-1.4**	**-2.7**
SART2	squamous cell carcinoma antigen recognized by T cells 2	**-2.9**	**-4.5**
SART3	squamous cell carcinoma antigen recognized by T cells 3	**-1.4**	
SAT	spermidine/spermine N1-acetyltransferase 1	**3.9**	**5.5**
SATB1	special AT-rich sequence binding protein 1 (binds to nuclear matrix/scaffold-associating DNA's)	**-1.8**	**6.4**
SBF1	SET binding factor 1	**3.1**	**-1.9**
SBF2	SET binding factor 2	**-1.2**	**2.4**
SC4MOL	sterol-C4-methyl oxidase-like	**1.6**	**-3.2**
SC5DL	sterol-C5-desaturase (ERG3 delta-5-desaturase homolog, fungal)-like		**-1.7**
SCAMP1	secretory carrier membrane protein 1		**1.7**
SCAMP2	secretory carrier membrane protein 2	**1.4**	
SCAMP5	secretory carrier membrane protein 5		**4.4**
SCAND1	SCAN domain containing 1	**1.6**	

SCAP	SREBF chaperone		1.2
SCAP2	src kinase associated phosphoprotein 2	1.3	1.5
SCARB1	scavenger receptor class B, member 1	-4.9	-1.4
SCARF1	scavenger receptor class F, member 1		-1.4
SCC-112	SCC-112 protein	1.3	-1.4
SCD	stearoyl-CoA desaturase (delta-9-desaturase)	-3.7	
SCFD1	sec1 family domain containing 1	2.2	
SCHIP1	schwannomin interacting protein 1	1.4	-5.1
SCO2	SCO cytochrome oxidase deficient homolog 2 (yeast)		1.5
SCP2	sterol carrier protein 2	1.6	-12.0
SCRIB	scribbled homolog (Drosophila)		1.5
SCUBE2	signal peptide, CUB domain, EGF-like 2	2.4	
SCYE1	small inducible cytokine subfamily E, member 1 (endothelial monocyte-activating)	-1.4	1.6
SDC4	syndecan 4 (amphiglycan, ryudocan)	2.2	
SDCBP	syndecan binding protein (syntenin)	1.5	-1.5
SDFR1	neuroplastin		1.5
SDHA	succinate dehydrogenase complex, subunit A, flavoprotein (Fp)	2.0	-1.5
SDHB	succinate dehydrogenase complex, subunit B, iron sulfur (Ip)	-1.4	
SDHC	succinate dehydrogenase complex, subunit C, integral membrane protein, 15kDa		-1.3
SDS3	suppressor of defective silencing 3 homolog (S. cerevisiae)	-1.3	
SEC10L1	exocyst complex component 5	1.3	
SEC13L1	SEC13 homolog (S. cerevisiae)		-1.2
SEC14L1	SEC14-like 1 (S. cerevisiae)	24.5	
SEC15L1	exocyst complex component 6		2.1
SEC22L1	SEC22 vesicle trafficking protein homolog B (S. cerevisiae)		-1.9
SEC23A	Sec23 homolog A (S. cerevisiae)	1.9	
SEC23B	Sec23 homolog B (S. cerevisiae)	-1.5	
SEC23IP	SEC23 interacting protein		1.4
SEC3L1	exocyst complex component 1	1.3	3.1
SEC5L1	exocyst complex component 2		-1.8
SEC61A2	Sec61 alpha 2 subunit (S. cerevisiae)	-1.8	1.5
SEC61G	Sec61 gamma subunit		2.2
SEC63	SEC63 homolog (S. cerevisiae)	-1.3	1.5
SEC6L1	exocyst complex component 3	1.8	
SEC8L1	exocyst complex component 4	1.8	
SECISBP2	SECIS binding protein 2	-1.3	-1.7
SEL1L	sel-1 suppressor of lin-12-like (C. elegans)		1.7
SELL	selectin L (lymphocyte adhesion molecule 1)	-3.1	-7.7
SELS	selenoprotein S		1.7
SEMA4C	sema domain, immunoglobulin domain (Ig), transmembrane domain (TM) and short cytoplasmic domain, (semaphorin) 4C		5.7
SEMA4D	sema domain, immunoglobulin domain (Ig), transmembrane domain (TM) and short cytoplasmic domain, (semaphorin) 4D		-1.5
SENP1	SUMO1/sentrin specific peptidase 1		-1.4

SENP3	SUMO1/sentrin/SMT3 specific peptidase 3	**-1.7**	**-2.9**
SENP6	SUMO1/sentrin specific peptidase 6	**1.5**	**2.0**
SEPHS1	selenophosphate synthetase 1	**-2.1**	**-1.6**
SEPP1	selenoprotein P, plasma, 1	**1.9**	
SEPT11	septin 11	**1.7**	**-1.9**
SEPT2	septin 2	**-1.5**	**-3.6**
SEPT6	septin 6	**-2.0**	**-1.4**
SEPT7	septin 7	**1.3**	**2.6**
SEPT8	septin 8	**-1.4**	**1.5**
SEPT9	septin 9		**-2.8**
SERP1	stress-associated endoplasmic reticulum protein 1	**-1.7**	**1.5**
SERPINA1	serpin peptidase inhibitor, clade A (alpha-1 antiproteinase, antitrypsin), member 1		**213.5**
SERPINB1	serpin peptidase inhibitor, clade B (ovalbumin), member 1	**3.1**	**40.4**
SERPINB6	serpin peptidase inhibitor, clade B (ovalbumin), member 6		**29.7**
SERPINB9	serpin peptidase inhibitor, clade B (ovalbumin), member 9	**-1.5**	**104.2**
SERPINF1	serpin peptidase inhibitor, clade F (alpha-2 antiplasmin, pigment epithelium derived factor), member 1		**-4.7**
SERPINH1	serpin peptidase inhibitor, clade H (heat shock protein 47), member 1, (collagen binding protein 1)		**18.1**
SERTAD1	SERTA domain containing 1	**2.5**	**2.4**
SERTAD2	SERTA domain containing 2		**1.5**
SESN1	sestrin 1	**4.4**	**1.6**
SESN2	sestrin 2	**-2.1**	
SET	SET translocation (myeloid leukemia-associated)	**-1.9**	**-1.3**
SET8	SET domain containing (lysine methyltransferase) 8		**-1.6**
SF1	splicing factor 1	**-1.4**	**-1.6**
SF3A1	splicing factor 3a, subunit 1, 120kDa		**-1.4**
SF3A2	splicing factor 3a, subunit 2, 66kDa	**-1.6**	**-1.5**
SF3A3	splicing factor 3a, subunit 3, 60kDa	**-1.4**	
SF3B1	splicing factor 3b, subunit 1, 155kDa		**1.8**
SF3B3	splicing factor 3b, subunit 3, 130kDa	**-1.7**	**-1.4**
SFI1	--		**-1.4**
SFN	stratifin	**-1.8**	**-1.7**
SFPQ	splicing factor proline/glutamine-rich (polypyrimidine tract binding protein associated)	**1.2**	**1.4**
SFRS1	splicing factor, arginine/serine-rich 1 (splicing factor 2, alternate splicing factor)	**-1.7**	**-1.6**
SFRS10	splicing factor, arginine/serine-rich 10 (transformer 2 homolog, Drosophila)	**-1.2**	**-1.7**
SFRS11	splicing factor, arginine/serine-rich 11	**4.2**	**2.7**
SFRS12	splicing factor, arginine/serine-rich 12	**-1.7**	**4.5**
SFRS2	splicing factor, arginine/serine-rich 2	**-1.4**	**-1.3**
SFRS3	splicing factor, arginine/serine-rich 3	**1.3**	**-1.4**
SFRS4	splicing factor, arginine/serine-rich 4		**-1.2**
SFRS6	splicing factor, arginine/serine-rich 6		**-3.6**
SFRS7	splicing factor, arginine/serine-rich 7, 35kDa	**-1.5**	**-1.8**
SFRS9	splicing factor, arginine/serine-rich 9	**1.2**	**-2.7**

SGCB	sarcoglycan, beta (43kDa dystrophin-associated glycoprotein)		**-6.4**
SGK	serum/glucocorticoid regulated kinase	**4.4**	
SGKL	serum/glucocorticoid regulated kinase family, member 3	**-1.4**	**1.9**
SGOL1	shugoshin-like 1 (S. pombe)	**1.7**	**-1.5**
SGPL1	sphingosine-1-phosphate lyase 1	**-1.3**	
SGPP1	sphingosine-1-phosphate phosphatase 1	**1.8**	**-3.0**
SH2D2A	SH2 domain protein 2A		**-5.6**
SH2D3C	SH2 domain containing 3C	**-1.9**	**-32.9**
SH3BP5	SH3-domain binding protein 5 (BTK-associated)	**2.6**	
SH3D19	SH3 domain protein D19		**2.9**
SH3GLB1	SH3-domain GRB2-like endophilin B1	**2.2**	**1.5**
SH3GLB2	SH3-domain GRB2-like endophilin B2		**-4.7**
SH3KBP1	SH3-domain kinase binding protein 1		**-1.6**
SH3MD1	SH3 and PX domains 2A	**2.3**	**4.8**
SH3MD2	SH3 domain containing ring finger 1	**2.1**	**2.1**
SHANK3	SH3 and multiple ankyrin repeat domains 3	**2.1**	**74.4**
SHB	Src homology 2 domain containing adaptor protein B	**-1.7**	**8.2**
SHC1	SHC (Src homology 2 domain containing) transforming protein 1		**-1.7**
SHCBP1	SHC SH2-domain binding protein 1	**1.2**	**-1.3**
SHFM1	split hand/foot malformation (ectrodactyly) type 1		**-1.8**
SHMT1	serine hydroxymethyltransferase 1 (soluble)	**-1.8**	**-1.5**
SHMT2	serine hydroxymethyltransferase 2 (mitochondrial)	**-1.8**	**-2.3**
SHPRH	SNF2 histone linker PHD RING helicase		**-1.3**
SIAH1	seven in absentia homolog 1 (Drosophila)		**-1.5**
SIAHBP1	fuse-binding protein-interacting repressor	**-1.2**	
SIM2	single-minded homolog 2 (Drosophila)		**2.4**
SIN3A	SIN3 homolog A, transcription regulator (yeast)	**-1.4**	
SIN3B	SIN3 homolog B, transcription regulator (yeast)	**1.3**	
SIPA1	signal-induced proliferation-associated gene 1		**-4.6**
SIRT1	sirtuin (silent mating type information regulation 2 homolog) 1 (S. cerevisiae)	**1.3**	
SIT	signaling threshold regulating transmembrane adaptor 1		**-18.5**
SIVA	SIVA1, apoptosis-inducing factor	**-1.7**	**-1.6**
SIX3	sine oculis homeobox homolog 3 (Drosophila)	**-2.3**	
SIX4	sine oculis homeobox homolog 4 (Drosophila)		**5.2**
SKB1	protein arginine methyltransferase 5	**-2.4**	**-1.3**
SKI	v-ski sarcoma viral oncogene homolog (avian)		**7.9**
SKIL	SKI-like oncogene	**1.7**	**-2.5**
SKIP (C62)	skeletal muscle and kidney enriched inositol phosphatase		**-1.7**
SKP2	S-phase kinase-associated protein 2 (p45)	**-3.1**	**-2.7**
SLA	Src-like-adaptor	**4.4**	**2.1**
SLAMF6	SLAM family member 6	**-1.8**	
SLBP	stem-loop (histone) binding protein	**-1.3**	**-1.8**

SLC11A2	solute carrier family 11 (proton-coupled divalent metal ion transporters), member 2	**-2.1**	**2.1**
SLC12A2	solute carrier family 12 (sodium/potassium/chloride transporters), member 2		**-1.9**
SLC12A7	solute carrier family 12 (potassium/chloride transporters), member 7		**-2.1**
SLC16A1	solute carrier family 16, member 1 (monocarboxylic acid transporter 1)	**-2.8**	**-1.2**
SLC16A2	solute carrier family 16, member 2 (monocarboxylic acid transporter 8)	**1.3**	
SLC16A3	solute carrier family 16, member 3 (monocarboxylic acid transporter 4)		**9.6**
SLC16A5	solute carrier family 16, member 5 (monocarboxylic acid transporter 6)		**2.4**
SLC16A6	solute carrier family 16, member 6 (monocarboxylic acid transporter 7)	**-1.6**	
SLC18A2	solute carrier family 18 (vesicular monoamine), member 2	**1.6**	**4.3**
SLC19A1	solute carrier family 19 (folate transporter), member 1	**-1.7**	
SLC1A4	solute carrier family 1 (glutamate/neutral amino acid transporter), member 4	**-1.8**	**-11.7**
SLC1A5	solute carrier family 1 (neutral amino acid transporter), member 5	**-2.3**	**-2.0**
SLC20A1	solute carrier family 20 (phosphate transporter), member 1	**-1.4**	**-2.2**
SLC23A2	solute carrier family 23 (nucleobase transporters), member 2	**-1.8**	**1.3**
SLC25A1	solute carrier family 25 (mitochondrial carrier; citrate transporter), member 1	**-1.3**	
SLC25A15	solute carrier family 25 (mitochondrial carrier; ornithine transporter) member 15		**-1.3**
SLC25A20	solute carrier family 25 (carnitine/acylcarnitine translocase), member 20	**1.4**	**2.2**
SLC25A3	solute carrier family 25 (mitochondrial carrier; phosphate carrier), member 3		**1.2**
SLC26A2	solute carrier family 26 (sulfate transporter), member 2	**1.3**	**2.8**
SLC26A6	solute carrier family 26, member 6		**2.9**
SLC27A2	solute carrier family 27 (fatty acid transporter), member 2		**2.2**
SLC27A3	solute carrier family 27 (fatty acid transporter), member 3	**3.3**	**-3.5**
SLC29A1	solute carrier family 29 (nucleoside transporters), member 1	**-2.6**	
SLC29A2	solute carrier family 29 (nucleoside transporters), member 2		**-1.3**
SLC2A3	solute carrier family 2 (facilitated glucose transporter), member 3	**6.0**	**-1.9**
SLC2A5	solute carrier family 2 (facilitated glucose/fructose transporter), member 5	**2.4**	
SLC2A9	solute carrier family 2 (facilitated glucose transporter), member 9		**1.7**
SLC30A1	solute carrier family 30 (zinc transporter), member 1		**2.2**
SLC30A5	solute carrier family 30 (zinc transporter), member 5	**-1.5**	**1.2**
SLC30A7	solute carrier family 30 (zinc transporter), member 7	**1.2**	**1.9**
SLC30A9	solute carrier family 30 (zinc transporter), member 9		**2.1**
SLC38A1	solute carrier family 38, member 1	**3.0**	**-2.4**
SLC38A2	solute carrier family 38, member 2	**1.5**	
SLC39A14	solute carrier family 39 (zinc transporter), member 14	**-3.8**	**1.9**
SLC39A8	solute carrier family 39 (zinc transporter), member 8	**-3.1**	
SLC3A2	solute carrier family 3 (activators of dibasic and neutral amino acid transport), member 2		**-1.3**
SLC40A1	solute carrier family 40 (iron-regulated transporter), member 1		**10.4**
SLC43A1	solute carrier family 43, member 1	**-2.0**	**1.8**
SLC43A3	solute carrier family 43, member 3	**-2.1**	**-5.3**
SLC44A2	solute carrier family 44, member 2		**-3.0**
SLC6A6	solute carrier family 6 (neurotransmitter transporter, taurine), member 6		**8.8**
SLC7A1	solute carrier family 7 (cationic amino acid transporter, y+ system), member 1	**-2.7**	**-2.4**

SLC7A11	solute carrier family 7, (cationic amino acid transporter, y+ system) member 11		**-3.7**
SLC7A5	solute carrier family 7 (cationic amino acid transporter, y+ system), member 5		**-3.9**
SLC7A7	solute carrier family 7 (cationic amino acid transporter, y+ system), member 7	**-1.5**	
SLC8A1	solute carrier family 8 (sodium/calcium exchanger), member 1	**-8.5**	**-3.0**
SLC9A2	solute carrier family 9 (sodium/hydrogen exchanger), member 2	**-1.5**	**1.4**
SLC9A3R1	solute carrier family 9 (sodium/hydrogen exchanger), member 3 regulator 1	**-1.4**	**1.6**
SLIT2	slit homolog 2 (Drosophila)	**-1.8**	
SLK	STE20-like kinase (yeast)		**4.3**
SLPI	secretory leukocyte peptidase inhibitor		**6.9**
SMAD1	SMAD family member 1	**2.7**	**1.2**
SMAD2	SMAD family member 2	**-1.3**	**-1.7**
SMAD3	SMAD family member 3	**3.8**	
SMAD4	SMAD family member 4	**1.6**	**-1.5**
SMAD5	SMAD family member 5		**-1.5**
SMAD6	SMAD family member 6		**5.6**
SMAD7	SMAD family member 7	**2.6**	
SMAD9	SMAD family member 9	**-1.6**	**-18.4**
SMARCA2	SWI/SNF related, matrix associated, actin dependent regulator of chromatin, subfamily a, member 2	**2.2**	**-1.2**
SMARCA3	helicase-like transcription factor	**-1.4**	**-1.4**
SMARCA4	SWI/SNF related, matrix associated, actin dependent regulator of chromatin, subfamily a, member 4	**-1.6**	**-1.9**
SMARCA5	SWI/SNF related, matrix associated, actin dependent regulator of chromatin, subfamily a, member 5	**-1.5**	
SMARCAD1	SWI/SNF-related, matrix-associated actin-dependent regulator of chromatin, subfamily a, containing DEAD/H box 1	**-2.4**	**-1.2**
SMARCB1	SWI/SNF related, matrix associated, actin dependent regulator of chromatin, subfamily b, member 1		**-1.3**
SMARCC1	SWI/SNF related, matrix associated, actin dependent regulator of chromatin, subfamily c, member 1	**-2.2**	**-1.4**
SMARCC2	SWI/SNF related, matrix associated, actin dependent regulator of chromatin, subfamily c, member 2		**-1.5**
SMARCD1	SWI/SNF related, matrix associated, actin dependent regulator of chromatin, subfamily d, member 1		**-1.9**
SMARCD2	SWI/SNF related, matrix associated, actin dependent regulator of chromatin, subfamily d, member 2	**-1.2**	**1.3**
SMARCE1	SWI/SNF related, matrix associated, actin dependent regulator of chromatin, subfamily e, member 1		**-2.2**
SMC1L1	structural maintenance of chromosomes 1A	**-1.2**	**-2.2**
SMC2L1	structural maintenance of chromosomes 2	**1.3**	**-2.3**
SMC4L1	structural maintenance of chromosomes 4	**1.4**	**-1.5**
SMC6L1	structural maintenance of chromosomes 6	**-1.4**	**2.0**
SMG1	PI-3-kinase-related kinase SMG-1		**-1.8**
SMNDC1	survival motor neuron domain containing 1		**1.6**
SMO	smoothened homolog (Drosophila)		**1.7**
SMOX	spermine oxidase	**2.1**	
SMS	spermine synthase	**-1.4**	
SMTN	smoothelin	**2.0**	
SMUG1	single-strand-selective monofunctional uracil-DNA glycosylase 1		**1.7**
SMURF1	SMAD specific E3 ubiquitin protein ligase 1		**-1.7**
SMURF2	SMAD specific E3 ubiquitin protein ligase 2	**1.6**	**1.6**
SMYD3	SET and MYND domain containing 3	**-1.5**	**1.4**

SNAI2	snail homolog 2 (Drosophila)		**3.1**
SNAP29	synaptosomal-associated protein, 29kDa	**1.5**	
SNAPAP	SNAP-associated protein		**-1.5**
SNAPC1	small nuclear RNA activating complex, polypeptide 1, 43kDa	**-1.5**	
SNAPC3	small nuclear RNA activating complex, polypeptide 3, 50kDa	**1.3**	**-3.2**
SNCA	synuclein, alpha (non A4 component of amyloid precursor)		**-2.7**
SNCAIP	synuclein, alpha interacting protein (synphilin)		**-1.3**
SND1	staphylococcal nuclease and tudor domain containing 1		**-1.3**
SNF8	SNF8, ESCRT-II complex subunit, homolog (S. cerevisiae)	**-1.3**	
SNN	stannin		**-3.8**
SNRK	SNF related kinase		**1.2**
SNRP70	small nuclear ribonucleoprotein 70kDa polypeptide (RNP antigen)	**-1.5**	
SNRPA	small nuclear ribonucleoprotein polypeptide A	**-1.9**	
SNRPB	small nuclear ribonucleoprotein polypeptides B and B1	**-1.7**	**-1.6**
SNRPC	small nuclear ribonucleoprotein polypeptide C	**-1.5**	**-1.3**
SNRPD1	small nuclear ribonucleoprotein D1 polypeptide 16kDa	**-1.7**	**-1.9**
SNRPD2	small nuclear ribonucleoprotein D2 polypeptide 16.5kDa	**-1.2**	**-1.2**
SNRPD3	small nuclear ribonucleoprotein D3 polypeptide 18kDa		**-1.6**
SNRPE	small nuclear ribonucleoprotein polypeptide E	**-1.3**	**-1.3**
SNRPN	small nuclear ribonucleoprotein polypeptide N	**-1.2**	
SNTB1	syntrophin, beta 1 (dystrophin-associated protein A1, 59kDa, basic component 1)	**-2.0**	**-1.8**
SNTB2	syntrophin, beta 2 (dystrophin-associated protein A1, 59kDa, basic component 2)	**3.5**	**-5.9**
SNTG1	syntrophin, gamma 1	**-2.1**	
SNX1	sorting nexin 1		**3.6**
SNX2	sorting nexin 2	**-1.4**	**-1.6**
SNX26	sorting nexin 26		**-2.4**
SNX3	sorting nexin 3	**-1.5**	**1.3**
SNX4	sorting nexin 4		**-1.2**
SNX5	sorting nexin 5	**4.1**	**2.1**
SNX6	sorting nexin 6		**1.3**
SNX9	sorting nexin 9		**4.6**
SOAT1	sterol O-acyltransferase (acyl-Coenzyme A: cholesterol acyltransferase) 1		**-1.4**
SOCS1	suppressor of cytokine signaling 1	**8.3**	**-1.6**
SOCS2	suppressor of cytokine signaling 2	**2.8**	**-1.8**
SOCS4	suppressor of cytokine signaling 4		**-1.4**
SOCS5	suppressor of cytokine signaling 5	**1.3**	**1.7**
SOCS6	suppressor of cytokine signaling 6	**1.9**	**1.3**
SOCS7	suppressor of cytokine signaling 7	**-1.7**	
SOD2	superoxide dismutase 2, mitochondrial	**2.2**	**-1.6**
SON	SON DNA binding protein	**1.7**	
SORD	sorbitol dehydrogenase	**-1.6**	**-1.3**
SORL1	sortilin-related receptor, L(DLR class) A repeats-containing		**4.2**

SORT1	sortilin 1	**1.6**	**1.5**
SOS1	son of sevenless homolog 1 (Drosophila)		**1.3**
SOS2	son of sevenless homolog 2 (Drosophila)	**-1.5**	
SOX4	SRY (sex determining region Y)-box 4	**-1.3**	**-5.7**
SOX7	SRY (sex determining region Y)-box 7	**1.7**	**-5.9**
SP1	Sp1 transcription factor	**-1.3**	**-1.4**
SP100	SP100 nuclear antigen	**2.3**	**-1.7**
SP110	--	**-1.9**	**-4.1**
SP140	SP140 nuclear body protein	**1.7**	
SP3	Sp3 transcription factor		**-2.8**
SP4	Sp4 transcription factor	**-1.4**	**1.3**
SPAG1	sperm associated antigen 1		**-7.7**
SPAG4	sperm associated antigen 4	**6.3**	
SPAG5	sperm associated antigen 5	**1.5**	
SPAG9	sperm associated antigen 9	**1.3**	**2.0**
SPARC	secreted protein, acidic, cysteine-rich (osteonectin)	**1.9**	**30.2**
SPAST	spastin		**1.3**
SPBC25	spindle pole body component 25 homolog (S. cerevisiae)		**1.4**
SPEN	spen homolog, transcriptional regulator (Drosophila)	**-1.3**	
SPG20	spastic paraplegia 20, spartin (Troyer syndrome)		**-1.9**
SPG21	spastic paraplegia 21, maspardin (autosomal recessive, Mast syndrome)	**-1.4**	**1.4**
SPHK1	sphingosine kinase 1		**6.4**
SPHK2	sphingosine kinase 2		**-1.7**
SPIB	Spi-B transcription factor (Spi-1/PU.1 related)		**-9.3**
SPIN	spindlin 1		**-1.4**
SPINT2	serine peptidase inhibitor, Kunitz type, 2		**4.2**
SPN	sialophorin (leukosialin, CD43)	**-1.2**	**-1.9**
SPR	sepiapterin reductase (7,8-dihydrobiopterin:NADP+ oxidoreductase)		**10.3**
SPRED1	sprouty-related, EVH1 domain containing 1	**2.2**	**-3.2**
SPRY1	sprouty homolog 1, antagonist of FGF signaling (Drosophila)	**11.8**	
SPRY2	sprouty homolog 2 (Drosophila)	**1.5**	
SPTA1	spectrin, alpha, erythrocytic 1 (elliptocytosis 2)	**3.0**	
SPTAN1	spectrin, alpha, non-erythrocytic 1 (alpha-fodrin)	**-1.3**	
SPTBN1	spectrin, beta, non-erythrocytic 1	**2.4**	**-1.4**
SPTBN2	spectrin, beta, non-erythrocytic 2		**-2.3**
SPTLC1	serine palmitoyltransferase, long chain base subunit 1		**1.7**
SPTLC2	serine palmitoyltransferase, long chain base subunit 2		**2.0**
SQLE	squalene epoxidase	**-1.4**	**-2.0**
SQRDL	sulfide quinone reductase-like (yeast)		**7.9**
SQSTM1	--		**1.4**
SRA1	steroid receptor RNA activator 1		**-1.3**
SRD5A1	steroid-5-alpha-reductase, alpha polypeptide 1 (3-oxo-5 alpha-steroid delta 4-dehydrogenase alpha 1)	**1.2**	**2.0**

SREBF2	sterol regulatory element binding transcription factor 2		-1.8
SRF	serum response factor (c-fos serum response element-binding transcription factor)		-1.3
SRFBP1	serum response factor binding protein 1	-1.7	
SRGAP2	SLIT-ROBO Rho GTPase activating protein 2	2.3	
SRI	sorcin		1.5
SRM	spermidine synthase	-3.4	
SRP14	signal recognition particle 14kDa (homologous Alu RNA binding protein)		-1.4
SRP68	signal recognition particle 68kDa	-1.2	1.7
SRP72	signal recognition particle 72kDa	-1.5	3.8
SRPK1	SFRS protein kinase 1	-1.5	
SRPK2	SFRS protein kinase 2	1.2	-1.3
SRR	serine racemase	-2.2	-2.1
SRRM1	serine/arginine repetitive matrix 1	-1.5	
SRRM2	serine/arginine repetitive matrix 2	-1.4	-1.9
SS18	synovial sarcoma translocation, chromosome 18		1.5
SSA2	TROVE domain family, member 2	1.4	
SSB	Sjogren syndrome antigen B (autoantigen La)	-1.7	1.7
SSB1	splA/ryanodine receptor domain and SOCS box containing 1	-1.5	-2.7
SSB3	splA/ryanodine receptor domain and SOCS box containing 3		-1.4
SSBP1	single-stranded DNA binding protein 1	-1.8	1.3
SSH1	slingshot homolog 1 (Drosophila)	-1.4	2.3
SSR3	signal sequence receptor, gamma (translocon-associated protein gamma)	1.9	2.0
SSR4	signal sequence receptor, delta (translocon-associated protein delta)	-1.3	1.7
SSRP1	structure specific recognition protein 1	-2.2	-1.8
SSTR2	somatostatin receptor 2	-1.8	-1.2
SSX2IP	synovial sarcoma, X breakpoint 2 interacting protein	-1.7	
ST13	suppression of tumorigenicity 13 (colon carcinoma) (Hsp70 interacting protein)	-1.5	1.5
ST18	suppression of tumorigenicity 18 (breast carcinoma) (zinc finger protein)		37.3
ST3GAL1	ST3 beta-galactoside alpha-2,3-sialyltransferase 1	1.9	
ST3GAL2	ST3 beta-galactoside alpha-2,3-sialyltransferase 2		1.7
ST3GAL3	ST3 beta-galactoside alpha-2,3-sialyltransferase 3		-1.2
ST3GAL5	ST3 beta-galactoside alpha-2,3-sialyltransferase 5		4.7
ST3GAL6	ST3 beta-galactoside alpha-2,3-sialyltransferase 6	1.9	13.7
ST6GAL1	ST6 beta-galactosamide alpha-2,6-sialyltranferase 1		-1.7
ST6GALNAC2	ST6 (alpha-N-acetyl-neuraminyl-2,3-beta-galactosyl-1,3)-N-acetylgalactosaminide alpha-2,6-sialyltransferase 2	-2.3	2.7
ST6GALNAC4	ST6 (alpha-N-acetyl-neuraminyl-2,3-beta-galactosyl-1,3)-N-acetylgalactosaminide alpha-2,6-sialyltransferase 4		-1.7
ST8SIA4	ST8 alpha-N-acetyl-neuraminide alpha-2,8-sialyltransferase 4	3.0	-1.7
STAF42	transcriptional adaptor 1 (HFI1 homolog, yeast)-like	-1.2	
STAG1	stromal antigen 1		-1.4
STAG2	stromal antigen 2		-1.6
STAG3	stromal antigen 3	9.7	
STAM	signal transducing adaptor molecule (SH3 domain and ITAM motif) 1		1.6

STAM2	signal transducing adaptor molecule (SH3 domain and ITAM motif) 2	**1.9**	**2.4**
STAMBP	STAM binding protein		**-1.2**
STAT1	signal transducer and activator of transcription 1, 91kDa	**-1.5**	**1.7**
STAT2	signal transducer and activator of transcription 2, 113kDa	**1.2**	
STAT3	signal transducer and activator of transcription 3 (acute-phase response factor)		**3.8**
STAT5A	signal transducer and activator of transcription 5A		**2.7**
STAT6	signal transducer and activator of transcription 6, interleukin-4 induced	**-1.4**	
STATIP1	signal transducer and activator of transcription 3 interacting protein 1		**1.4**
STAU	staufen, RNA binding protein, homolog 1 (Drosophila)		**-1.2**
STAU2	staufen, RNA binding protein, homolog 2 (Drosophila)	**1.6**	**-2.9**
STCH	stress 70 protein chaperone, microsome-associated, 60kDa		**1.6**
STIM1	stromal interaction molecule 1	**1.9**	**-1.4**
STIM2	stromal interaction molecule 2	**-2.2**	**-7.4**
STIP1	stress-induced-phosphoprotein 1 (Hsp70/Hsp90-organizing protein)	**-1.4**	**-1.6**
STK10	serine/threonine kinase 10		**-1.4**
STK17B	serine/threonine kinase 17b (apoptosis-inducing)	**1.6**	
STK24	serine/threonine kinase 24 (STE20 homolog, yeast)	**1.3**	**-2.2**
STK25	serine/threonine kinase 25 (STE20 homolog, yeast)	**1.5**	
STK3	serine/threonine kinase 3 (STE20 homolog, yeast)	**1.5**	**-2.2**
STK35	serine/threonine kinase 35		**-1.2**
STK38	serine/threonine kinase 38	**1.7**	**-2.3**
STK39	serine threonine kinase 39 (STE20/SPS1 homolog, yeast)	**1.8**	**-2.5**
STK4	serine/threonine kinase 4	**-1.5**	
STK6	aurora kinase A	**1.7**	
STMN1	stathmin 1/oncoprotein 18	**-1.4**	
STMN3	stathmin-like 3		**-2.4**
STN2	stonin 2		**1.7**
STOM	stomatin	**-1.8**	**6.2**
STRAP	serine/threonine kinase receptor associated protein		**-1.4**
STRN3	striatin, calmodulin binding protein 3	**1.5**	
STS	steroid sulfatase (microsomal), arylsulfatase C, isozyme S		**-3.6**
STS-1	Cbl-interacting protein Sts-1	**2.6**	**-2.8**
STUB1	STIP1 homology and U-box containing protein 1	**-1.2**	**-1.4**
STX16	syntaxin 16		**1.5**
STX18	syntaxin 18	**-1.6**	
STX3A	syntaxin 3		**1.7**
STX6	syntaxin 6		**-1.9**
STX7	syntaxin 7	**1.3**	**1.5**
STX8	syntaxin 8		**-2.6**
STXBP1	syntaxin binding protein 1		**-13.5**
STXBP3	syntaxin binding protein 3	**1.7**	**-1.2**
STXBP4	syntaxin binding protein 4	**-1.9**	

STXBP5	syntaxin binding protein 5 (tomosyn)		3.8
STXBP6	syntaxin binding protein 6 (amisyn)		22.5
STYK1	serine/threonine/tyrosine kinase 1	2.7	
SUCLA2	succinate-CoA ligase, ADP-forming, beta subunit	-1.3	
SUCLG1	succinate-CoA ligase, GDP-forming, alpha subunit	-1.2	1.3
SUCLG2	succinate-CoA ligase, GDP-forming, beta subunit	-1.6	-1.4
SUFU	suppressor of fused homolog (Drosophila)		5.2
SUGT1	SGT1, suppressor of G2 allele of SKP1 (S. cerevisiae)		-1.6
SULF2	sulfatase 2	3.0	
SULT1A1	sulfotransferase family, cytosolic, 1A, phenol-preferring, member 1		-1.5
SULT1A3	sulfotransferase family, cytosolic, 1A, phenol-preferring, member 3	1.3	-2.2
SUMO1	SMT3 suppressor of mif two 3 homolog 1 (S. cerevisiae)	-1.3	1.2
SUMO2	SMT3 suppressor of mif two 3 homolog 2 (S. cerevisiae)		3.0
SUMO3	SMT3 suppressor of mif two 3 homolog 3 (S. cerevisiae)	-1.3	
SUPT16H	suppressor of Ty 16 homolog (S. cerevisiae)	-1.7	-1.4
SUPT3H	suppressor of Ty 3 homolog (S. cerevisiae)		-6.4
SUPT4H1	suppressor of Ty 4 homolog 1 (S. cerevisiae)	1.6	-1.7
SUPT6H	suppressor of Ty 6 homolog (S. cerevisiae)		1.8
SUPT7L	suppressor of Ty 7 (S. cerevisiae)-like	-1.9	1.7
SURF4	surfeit 4		1.9
SURF5	surfeit 5		-1.4
SURF6	surfeit 6		1.5
SUV39H2	suppressor of variegation 3-9 homolog 2 (Drosophila)	-1.3	-1.3
SV2A	synaptic vesicle glycoprotein 2A	1.9	10.1
SVIL	supervillin		1.9
SWAP70	SWAP-70 protein	1.4	-1.3
SYBL1	synaptobrevin-like 1	1.2	1.3
SYCP3	synaptonemal complex protein 3		1.4
SYK	spleen tyrosine kinase		-2.9
SYMPK	symplekin	-1.7	
SYN2	synapsin II	2.5	
SYNC1	syncoilin, intermediate filament 1	-1.6	2.0
SYNCRIP	synaptotagmin binding, cytoplasmic RNA interacting protein	-1.6	-1.7
SYNE2	spectrin repeat containing, nuclear envelope 2	6.9	-10.7
SYNGR2	synaptogyrin 2		1.9
SYNJ2	synaptojanin 2		-13.7
SYNJ2BP	synaptojanin 2 binding protein		-2.3
SYT1	synaptotagmin I	-1.5	-16.7
SYT11	synaptotagmin XI		-1.9
SYVN1	synovial apoptosis inhibitor 1, synoviolin		-1.8
T1A-2	podoplanin	-4.9	-11.5
TAB3	mitogen-activated protein kinase kinase kinase 7 interacting protein 3		1.3

TACC1	transforming, acidic coiled-coil containing protein 1		2.5
TACC3	transforming, acidic coiled-coil containing protein 3	1.9	
TACSTD1	tumor-associated calcium signal transducer 1		178.1
TAF1	TAF1 RNA polymerase II, TATA box binding protein (TBP)-associated factor, 250kDa		1.9
TAF10	TAF10 RNA polymerase II, TATA box binding protein (TBP)-associated factor, 30kDa		-1.4
TAF11	TAF11 RNA polymerase II, TATA box binding protein (TBP)-associated factor, 28kDa	1.2	
TAF15	TAF15 RNA polymerase II, TATA box binding protein (TBP)-associated factor, 68kDa	-1.4	1.6
TAF1A	TATA box binding protein (TBP)-associated factor, RNA polymerase I, A, 48kDa	-1.8	-2.1
TAF1B	TATA box binding protein (TBP)-associated factor, RNA polymerase I, B, 63kDa		-1.5
TAF1C	TATA box binding protein (TBP)-associated factor, RNA polymerase I, C, 110kDa		1.8
TAF2	TAF2 RNA polymerase II, TATA box binding protein (TBP)-associated factor, 150kDa		-1.3
TAF3	TAF3 RNA polymerase II, TATA box binding protein (TBP)-associated factor, 140kDa	-1.6	
TAF4B	TAF4b RNA polymerase II, TATA box binding protein (TBP)-associated factor, 105kDa		-1.3
TAF5	TAF5 RNA polymerase II, TATA box binding protein (TBP)-associated factor, 100kDa	-1.4	
TAF9	TAF9 RNA polymerase II, TATA box binding protein (TBP)-associated factor, 32kDa		2.2
TAF9B	TAF9B RNA polymerase II, TATA box binding protein (TBP)-associated factor, 31kDa	1.5	-1.5
TAGLN2	transgelin 2		-3.0
TAL1	T-cell acute lymphocytic leukemia 1		1.7
TALDO1	transaldolase 1		1.8
TANK	TRAF family member-associated NFKB activator	1.4	2.1
TAOK1	TAO kinase 1	1.5	1.9
TAOK3	TAO kinase 3	-1.4	2.3
TAP1	transporter 1, ATP-binding cassette, sub-family B (MDR/TAP)	1.9	
TAP2	transporter 2, ATP-binding cassette, sub-family B (MDR/TAP)	-1.8	-6.7
TAPBP	TAP binding protein (tapasin)	-1.3	-1.8
TARBP2	Tar (HIV-1) RNA binding protein 2	-1.4	
TARDBP	TAR DNA binding protein	-1.2	1.9
TARS	threonyl-tRNA synthetase	-1.9	
TARSL1	threonyl-tRNA synthetase-like 1	-1.8	
TARSL2	threonyl-tRNA synthetase-like 2	2.2	-1.5
TAX1BP1	Tax1 (human T-cell leukemia virus type I) binding protein 1	1.4	4.5
TAX1BP3	Tax1 (human T-cell leukemia virus type I) binding protein 3	3.3	-2.1
TBC1D1	TBC1 (tre-2/USP6, BUB2, cdc16) domain family, member 1	2.7	
TBC1D10A	TBC1 domain family, member 10A	6.2	-1.5
TBC1D22A	TBC1 domain family, member 22A		-1.5
TBC1D4	TBC1 domain family, member 4	-1.8	
TBC1D8	TBC1 domain family, member 8 (with GRAM domain)	-1.6	1.7
TBCD	tubulin folding cofactor D	2.4	
TBL1X	transducin (beta)-like 1X-linked	1.3	2.2
TBL1XR1	transducin (beta)-like 1X-linked receptor 1	1.5	1.3
TBP	TATA box binding protein	-1.3	
TBPL1	TBP-like 1		-1.6

TBRG4	transforming growth factor beta regulator 4		**15.7**
TBX21	T-box 21	**-2.4**	
TBXA2R	thromboxane A2 receptor	**-1.3**	**3.2**
TCEA1	transcription elongation factor A (SII), 1		**-1.7**
TCEAL1	transcription elongation factor A (SII)-like 1		**1.8**
TCEB1	transcription elongation factor B (SIII), polypeptide 1 (15kDa, elongin C)	**1.4**	**-1.6**
TCEB2	transcription elongation factor B (SIII), polypeptide 2 (18kDa, elongin B)		**-2.9**
TCEB3	transcription elongation factor B (SIII), polypeptide 3 (110kDa, elongin A)		**1.5**
TCERG1	transcription elongation regulator 1	**-1.4**	**-1.4**
TCF12	transcription factor 12 (HTF4, helix-loop-helix transcription factors 4)	**-2.0**	**-2.5**
TCF19	transcription factor 19 (SC1)		**-1.8**
TCF3	transcription factor 3 (E2A immunoglobulin enhancer binding factors E12/E47)		**-4.7**
TCF4	transcription factor 4	**-2.0**	**-17.2**
TCF7L2	transcription factor 7-like 2 (T-cell specific, HMG-box)		**2.0**
TCF8	transcription factor 8 (represses interleukin 2 expression)	**2.1**	**3.8**
TCFL1	vacuolar protein sorting 72 homolog (S. cerevisiae)	**-1.3**	
TCFL5	transcription factor-like 5 (basic helix-loop-helix)	**2.1**	**-2.5**
TCIRG1	T-cell, immune regulator 1, ATPase, H+ transporting, lysosomal V0 subunit A3	**1.8**	
TCL1A	T-cell leukemia/lymphoma 1A	**1.6**	**-5.7**
TCOF1	Treacher Collins-Franceschetti syndrome 1	**-1.5**	
TCP1	t-complex 1	**-1.5**	**-1.3**
TCTE1L	dynein, light chain, Tctex-type 3		**-1.2**
TCTEL1	dynein, light chain, Tctex-type 1	**1.5**	**1.2**
TD-60	regulator of chromosome condensation 2	**-1.5**	**-1.7**
TDE1	serine incorporator 3	**1.6**	
TDE2	serine incorporator 1	**2.0**	
TDRD7	tudor domain containing 7		**-2.6**
TEAD4	TEA domain family member 4		**5.1**
TEBP	prostaglandin E synthase 3 (cytosolic)	**-1.4**	
TEGT	testis enhanced gene transcript (BAX inhibitor 1)	**1.4**	**1.2**
TEP1	telomerase-associated protein 1	**1.7**	
TERF1	telomeric repeat binding factor (NIMA-interacting) 1		**-1.6**
TERF2	telomeric repeat binding factor 2		**-1.5**
TES	testis derived transcript (3 LIM domains)		**1.4**
TESK1	testis-specific kinase 1	**2.2**	
TEX10	testis expressed sequence 10	**-1.4**	**-1.2**
TFAM	transcription factor A, mitochondrial	**-1.8**	**-1.4**
TFAP2A	transcription factor AP-2 alpha (activating enhancer binding protein 2 alpha)	**-1.7**	
TFB1M	transcription factor B1, mitochondrial	**-1.5**	
TFB2M	transcription factor B2, mitochondrial	**-1.4**	
TFCP2	transcription factor CP2	**-1.9**	**-1.5**
TFDP1	transcription factor Dp-1	**-1.3**	**-1.8**

TFDP2	transcription factor Dp-2 (E2F dimerization partner 2)	**-1.4**	**3.1**
TFEB	transcription factor EB	**1.4**	**-3.9**
TFEC	transcription factor EC		**8.4**
TFIP11	tuftelin interacting protein 11	**3.5**	
TFPI	tissue factor pathway inhibitor (lipoprotein-associated coagulation inhibitor)	**9.6**	**4.9**
TFR2	transferrin receptor 2		**-2.5**
TFRC	transferrin receptor (p90, CD71)	**-4.1**	
TGFB1	transforming growth factor, beta 1 (Camurati-Engelmann disease)		**32.0**
TGFB1I1	transforming growth factor beta 1 induced transcript 1	**2.3**	
TGFBR2	transforming growth factor, beta receptor II (70/80kDa)	**1.8**	**-3.3**
TGFBR3	transforming growth factor, beta receptor III (betaglycan, 300kDa)		**-2.3**
TGFBRAP1	transforming growth factor, beta receptor associated protein 1		**1.3**
TGIF	TGFB-induced factor (TALE family homeobox)	**1.7**	
TGIF2	TGFB-induced factor 2 (TALE family homeobox)	**-1.6**	**1.9**
TGM4	transglutaminase 4 (prostate)	**-1.7**	
TGOLN2	trans-golgi network protein 2	**1.2**	**1.3**
THAP7	THAP domain containing 7	**-1.3**	
THBS1	thrombospondin 1	**13.1**	
THOC2	THO complex 2	**1.6**	**1.5**
THOC3	THO complex 3	**-1.2**	**-1.5**
THOC4	THO complex 4	**-1.4**	**-1.5**
THRAP1	thyroid hormone receptor associated protein 1	**1.5**	**1.9**
THRAP2	thyroid hormone receptor associated protein 2	**4.2**	**-1.5**
THRAP4	thyroid hormone receptor associated protein 4	**-1.5**	
THRAP5	thyroid hormone receptor associated protein 5		**-1.6**
THRAP6	thyroid hormone receptor associated protein 6	**1.8**	**-3.1**
THY1	Thy-1 cell surface antigen	**-1.4**	
TIA1	TIA1 cytotoxic granule-associated RNA binding protein	**-1.3**	**-1.7**
TIAL1	TIA1 cytotoxic granule-associated RNA binding protein-like 1	**-1.4**	**1.7**
TIAM1	T-cell lymphoma invasion and metastasis 1	**2.2**	
TICAM1	toll-like receptor adaptor molecule 1	**1.6**	
TICAM2	toll-like receptor adaptor molecule 2		**-2.4**
TIF1	tripartite motif-containing 24		**1.6**
TIMELESS	timeless homolog (Drosophila)	**-1.5**	
TIMM10	translocase of inner mitochondrial membrane 10 homolog (yeast)	**-2.2**	**-1.5**
TIMM13	translocase of inner mitochondrial membrane 13 homolog (yeast)	**-1.4**	
TIMM22	translocase of inner mitochondrial membrane 22 homolog (yeast)	**-1.4**	**-2.6**
TIMM23	translocase of inner mitochondrial membrane 23 homolog (yeast)	**-1.5**	
TIMM50	translocase of inner mitochondrial membrane 50 homolog (S. cerevisiae)	**-1.7**	**1.3**
TIMM9	translocase of inner mitochondrial membrane 9 homolog (yeast)	**-1.6**	**1.4**
TIMP2	TIMP metallopeptidase inhibitor 2		**2.1**
TIP120A	cullin-associated and neddylation-dissociated 1	**-1.3**	**1.6**

TIRAP	toll-interleukin 1 receptor (TIR) domain containing adaptor protein		**-1.2**
TJP2	tight junction protein 2 (zona occludens 2)	**-1.8**	
TK2	thymidine kinase 2, mitochondrial	**1.5**	
TKT	transketolase (Wernicke-Korsakoff syndrome)	**-1.3**	**1.5**
TLE1	transducin-like enhancer of split 1 (E(sp1) homolog, Drosophila)	**5.6**	**-2.0**
TLE3	transducin-like enhancer of split 3 (E(sp1) homolog, Drosophila)		**1.7**
TLE4	transducin-like enhancer of split 4 (E(sp1) homolog, Drosophila)	**-1.5**	**-1.4**
TLK1	tousled-like kinase 1	**1.4**	**1.7**
TLK2	tousled-like kinase 2		**1.7**
TLN1	talin 1		**-1.6**
TLP19	thioredoxin domain containing 12 (endoplasmic reticulum)	**-1.7**	
TLR1	toll-like receptor 1	**-5.0**	**-5.6**
TLR10	toll-like receptor 10	**-10.0**	**-9.5**
TLR2	toll-like receptor 2		**8.4**
TLR4	toll-like receptor 4	**-2.5**	
TLR5	toll-like receptor 5	**2.8**	
TLR7	toll-like receptor 7		**-18.6**
TLR9	toll-like receptor 9		**-2.3**
TM4SF2	tetraspanin 7	**4.8**	**35.8**
TM4SF7	tetraspanin 4	**-1.6**	
TM6SF1	transmembrane 6 superfamily member 1		**-7.2**
TM7SF2	transmembrane 7 superfamily member 2		**2.6**
TM7SF3	transmembrane 7 superfamily member 3	**-1.7**	**5.3**
TMED9	transmembrane emp24 protein transport domain containing 9		**1.8**
TMEM11	transmembrane protein 11		**-1.7**
TMEM23	transmembrane protein 23	**1.4**	**2.7**
TMEM4	transmembrane protein 4	**-1.5**	**1.9**
TMEM87B	transmembrane protein 87B	**1.2**	**1.8**
TMF1	TATA element modulatory factor 1	**1.7**	**2.1**
TMOD3	tropomodulin 3 (ubiquitous)	**1.6**	**-1.2**
TMP21	transmembrane emp24-like trafficking protein 10 (yeast)		**1.5**
TMPO	thymopoietin	**1.4**	**-2.2**
TMSB10	thymosin, beta 10	**1.6**	**-1.9**
TMSB4X	thymosin, beta 4, X-linked	**1.7**	
TMSL8	thymosin-like 8		**-5.4**
TNFAIP8	tumor necrosis factor, alpha-induced protein 8	**-1.9**	**-1.5**
TNFRSF10A	tumor necrosis factor receptor superfamily, member 10a	**2.4**	**6.0**
TNFRSF10D	tumor necrosis factor receptor superfamily, member 10d, decoy with truncated death domain		**1.3**
TNFRSF11A	tumor necrosis factor receptor superfamily, member 11a, NFKB activator		**-2.1**
TNFRSF1A	tumor necrosis factor receptor superfamily, member 1A		**2.9**
TNFRSF21	tumor necrosis factor receptor superfamily, member 21	**6.3**	
TNFRSF7	CD27 molecule	**2.3**	

TNFSF10	tumor necrosis factor (ligand) superfamily, member 10	**-5.3**	
TNFSF13	tumor necrosis factor (ligand) superfamily, member 13		**-1.8**
TNFSF13B	tumor necrosis factor (ligand) superfamily, member 13b		**16.6**
TNFSF4	tumor necrosis factor (ligand) superfamily, member 4 (tax-transcriptionally activated glycoprotein 1, 34kDa)	**2.8**	**-7.7**
TNFSF7	CD70 molecule		**-4.3**
TNIK	TRAF2 and NCK interacting kinase		**2.2**
TNIP1	TNFAIP3 interacting protein 1	**1.9**	
TNIP2	TNFAIP3 interacting protein 2	**1.5**	
TNK2	tyrosine kinase, non-receptor, 2	**1.6**	
TNKS	tankyrase, TRF1-interacting ankyrin-related ADP-ribose polymerase	**2.5**	**-1.7**
TNKS2	tankyrase, TRF1-interacting ankyrin-related ADP-ribose polymerase 2		**1.9**
TNPO1	transportin 1	**1.4**	**-1.4**
TNPO2	transportin 2 (importin 3, karyopherin beta 2b)	**-1.5**	
TNPO3	transportin 3		**1.4**
TNRC6A	trinucleotide repeat containing 6A	**-1.3**	**-2.7**
TNS	tensin 1	**3.4**	
TOB1	transducer of ERBB2, 1	**-1.6**	**1.9**
TOB2	transducer of ERBB2, 2	**1.4**	**1.9**
TOLLIP	toll interacting protein		**1.6**
TOM1L1	target of myb1-like 1 (chicken)		**5.1**
TOMM20	translocase of outer mitochondrial membrane 20 homolog (yeast)		**2.0**
TOMM22	translocase of outer mitochondrial membrane 22 homolog (yeast)	**-1.4**	
TOMM40	translocase of outer mitochondrial membrane 40 homolog (yeast)	**-1.4**	
TOMM70A	translocase of outer mitochondrial membrane 70 homolog A (S. cerevisiae)	**-1.5**	
TOP2A	topoisomerase (DNA) II alpha 170kDa	**1.8**	
TOP2B	topoisomerase (DNA) II beta 180kDa		**-1.9**
TOP3A	topoisomerase (DNA) III alpha	**-1.2**	**-1.5**
TOPBP1	topoisomerase (DNA) II binding protein 1		**-1.2**
TP53	tumor protein p53 (Li-Fraumeni syndrome)	**-2.5**	**-2.6**
TP53BP2	tumor protein p53 binding protein, 2		**2.0**
TP53INP1	tumor protein p53 inducible nuclear protein 1	**11.1**	**3.2**
TP53RK	TP53 regulating kinase	**-1.6**	**-1.3**
TP73L	tumor protein p73-like	**1.5**	
TPD52	tumor protein D52	**1.6**	**-3.0**
TPD52L2	tumor protein D52-like 2		**-1.6**
TPH2	tryptophan hydroxylase 2		**-4.6**
TPI1	triosephosphate isomerase 1	**-1.5**	**-1.5**
TPM1	tropomyosin 1 (alpha)		**-3.2**
TPM3	tropomyosin 3	**1.6**	**-3.4**
TPM4	tropomyosin 4	**1.8**	**-2.5**
TPMT	thiopurine S-methyltransferase		**1.8**
TPP1	tripeptidyl peptidase I	**1.4**	**1.7**

TPP2	tripeptidyl peptidase II		**-1.3**
TPR	translocated promoter region (to activated MET oncogene)	**-1.3**	**1.4**
TRA@	T cell receptor alpha locus	**-1.9**	**-38.6**
TRA1	heat shock protein 90kDa beta (Grp94), member 1		**1.4**
TRA2A	transformer-2 alpha		**2.6**
TRAF3	TNF receptor-associated factor 3		**2.8**
TRAF3IP2	TRAF3 interacting protein 2		**-5.3**
TRAF4	TNF receptor-associated factor 4	**-1.7**	**-13.8**
TRAF7	TNF receptor-associated factor 7		**-1.3**
TRAM1	translocation associated membrane protein 1		**3.3**
TRAM2	translocation associated membrane protein 2	**2.4**	**-2.0**
TRAPPC3	trafficking protein particle complex 3		**1.4**
TRERF1	transcriptional regulating factor 1	**-2.1**	**2.3**
TRFP	Trf (TATA binding protein-related factor)-proximal homolog (Drosophila)	**-1.4**	**-1.7**
TRH	thyrotropin-releasing hormone		**33.1**
TRHDE	thyrotropin-releasing hormone degrading enzyme		**-10.0**
TRIB3	tribbles homolog 3 (Drosophila)	**-2.3**	**1.4**
TRIM14	tripartite motif-containing 14	**-2.4**	**3.2**
TRIM21	tripartite motif-containing 21	**-1.6**	
TRIM22	tripartite motif-containing 22	**-1.3**	**-2.8**
TRIM23	tripartite motif-containing 23	**1.8**	
TRIM28	tripartite motif-containing 28	**-1.3**	
TRIM33	tripartite motif-containing 33		**-1.6**
TRIM5	tripartite motif-containing 5		**-2.2**
TRIM8	tripartite motif-containing 8	**1.5**	**-2.2**
TRIM9	tripartite motif-containing 9	**1.7**	
TRIO	triple functional domain (PTPRF interacting)	**2.8**	**2.0**
TRIP11	thyroid hormone receptor interactor 11	**1.3**	
TRIP12	thyroid hormone receptor interactor 12	**1.5**	**1.6**
TRIP6	thyroid hormone receptor interactor 6	**-1.2**	**-1.4**
TRPM6	transient receptor potential cation channel, subfamily M, member 6		**80.5**
TRPM7	transient receptor potential cation channel, subfamily M, member 7	**-1.5**	**-1.2**
TRPV2	transient receptor potential cation channel, subfamily V, member 2	**1.8**	
TRRAP	transformation/transcription domain-associated protein		**-1.7**
TRUB1	TruB pseudouridine (psi) synthase homolog 1 (E. coli)	**-2.0**	**-1.8**
TRUB2	TruB pseudouridine (psi) synthase homolog 2 (E. coli)	**-2.1**	
TSAP6	STEAP family member 3		**1.5**
TSC2	tuberous sclerosis 2	**1.5**	**-1.8**
TSC22D1	TSC22 domain family, member 1	**2.9**	**2.0**
TSC22D3	TSC22 domain family, member 3	**17.5**	**5.2**
TSEN2	tRNA splicing endonuclease 2 homolog (S. cerevisiae)	**-1.6**	
TSEN54	tRNA splicing endonuclease 54 homolog (S. cerevisiae)	**-1.5**	

TSN	translin	-1.3	
TSNAX	translin-associated factor X	1.9	1.2
TSPAN5	tetraspanin 5		3.2
TST	thiosulfate sulfurtransferase (rhodanese)	1.4	
TSTA3	tissue specific transplantation antigen P35B	1.6	
TTC11	fission 1 (mitochondrial outer membrane) homolog (S. cerevisiae)		-1.6
TTC3	tetratricopeptide repeat domain 3	-1.6	2.3
TTC5	tetratricopeptide repeat domain 5	-2.0	
TTF1	transcription termination factor, RNA polymerase I	-1.3	-2.7
TTF2	transcription termination factor, RNA polymerase II		-1.2
TTK	TTK protein kinase	1.3	
TTN	titin	-1.4	
TTPA	tocopherol (alpha) transfer protein (ataxia (Friedreich-like) with vitamin E deficiency)		-11.3
TUBA1	tubulin, alpha 1	2.1	-4.4
TUBA3	tubulin, alpha 3	1.7	-10.6
TUBB	tubulin, beta		-1.6
TUBB2A	tubulin, beta 2A	3.2	-25.5
TUBB2C	tubulin, beta 2C	1.4	-1.3
TUBB3	tubulin, beta 3		-1.8
TUBE1	tubulin, epsilon 1	-2.0	-1.5
TUBGCP2	tubulin, gamma complex associated protein 2	-1.3	
TUBGCP3	tubulin, gamma complex associated protein 3		-1.4
TUBGCP5	tubulin, gamma complex associated protein 5		-3.0
TUFM	Tu translation elongation factor, mitochondrial	-1.5	-1.5
TWIST1	twist homolog 1 (acrocephalosyndactyly 3; Saethre-Chotzen syndrome) (Drosophila)		-28.5
TWSG1	twisted gastrulation homolog 1 (Drosophila)	1.8	1.5
TXLNA	taxilin alpha		1.2
TXN	thioredoxin	1.3	1.8
TXN2	thioredoxin 2		-1.4
TXNDC9	thioredoxin domain containing 9		1.5
TXNIP	thioredoxin interacting protein	7.7	10.0
TXNL1	thioredoxin-like 1	-2.1	1.3
TXNL2	thioredoxin-like 2	-1.3	1.3
TXNRD1	thioredoxin reductase 1		-1.3
TYMS	thymidylate synthetase	-1.2	2.1
U2AF2	U2 small nuclear RNA auxiliary factor 2		-1.3
UAP1	UDP-N-acteylglucosamine pyrophosphorylase 1	-2.3	1.8
UBA2	SUMO1 activating enzyme subunit 2	-1.6	
UBA52	ubiquitin A-52 residue ribosomal protein fusion product 1		1.3
UBB	ubiquitin B	1.7	-1.6
UBC	ubiquitin C	1.6	
UBE1	ubiquitin-activating enzyme E1 (A1S9T and BN75 temperature sensitivity complementing)	-1.4	-1.3

UBE1C	ubiquitin-activating enzyme E1C (UBA3 homolog, yeast)	**1.2**	
UBE1L	ubiquitin-activating enzyme E1-like	**-1.4**	**-1.5**
UBE2A	ubiquitin-conjugating enzyme E2A (RAD6 homolog)	**1.4**	
UBE2B	ubiquitin-conjugating enzyme E2B (RAD6 homolog)	**1.2**	**-1.6**
UBE2C	ubiquitin-conjugating enzyme E2C	**1.7**	**1.3**
UBE2D1	ubiquitin-conjugating enzyme E2D 1 (UBC4/5 homolog, yeast)		**-1.3**
UBE2D2	ubiquitin-conjugating enzyme E2D 2 (UBC4/5 homolog, yeast)	**1.6**	**-1.9**
UBE2D3	ubiquitin-conjugating enzyme E2D 3 (UBC4/5 homolog, yeast)	**1.3**	**-1.3**
UBE2E1	ubiquitin-conjugating enzyme E2E 1 (UBC4/5 homolog, yeast)		**1.4**
UBE2E2	ubiquitin-conjugating enzyme E2E 2 (UBC4/5 homolog, yeast)		**2.7**
UBE2E3	ubiquitin-conjugating enzyme E2E 3 (UBC4/5 homolog, yeast)		**2.2**
UBE2G1	ubiquitin-conjugating enzyme E2G 1 (UBC7 homolog, yeast)		**-1.6**
UBE2G2	ubiquitin-conjugating enzyme E2G 2 (UBC7 homolog, yeast)	**-1.9**	
UBE2H	ubiquitin-conjugating enzyme E2H (UBC8 homolog, yeast)	**2.1**	**1.3**
UBE2I	ubiquitin-conjugating enzyme E2I (UBC9 homolog, yeast)	**-1.2**	**-4.5**
UBE2J1	ubiquitin-conjugating enzyme E2, J1 (UBC6 homolog, yeast)	**1.5**	**1.5**
UBE2J2	ubiquitin-conjugating enzyme E2, J2 (UBC6 homolog, yeast)	**-1.4**	**-1.3**
UBE2L3	ubiquitin-conjugating enzyme E2L 3	**-2.1**	**1.5**
UBE2L6	ubiquitin-conjugating enzyme E2L 6	**-1.7**	**-2.0**
UBE2N	ubiquitin-conjugating enzyme E2N (UBC13 homolog, yeast)	**-1.3**	**-1.4**
UBE2S	ubiquitin-conjugating enzyme E2S	**-2.1**	
UBE2T	ubiquitin-conjugating enzyme E2T (putative)	**-1.5**	**-2.3**
UBE2V1	ubiquitin-conjugating enzyme E2 variant 1		**1.6**
UBE2V2	ubiquitin-conjugating enzyme E2 variant 2	**-1.3**	**-1.4**
UBE3A	ubiquitin protein ligase E3A (human papilloma virus E6-associated protein, Angelman syndrome)	**1.4**	**-5.8**
UBE3C	ubiquitin protein ligase E3C		**-1.3**
UBE4B	ubiquitination factor E4B (UFD2 homolog, yeast)		**1.3**
UBL5	ubiquitin-like 5	**1.5**	**1.2**
UBQLN1	ubiquilin 1	**1.2**	**-1.8**
UBQLN4	ubiquilin 4		**1.4**
UBR1	ubiquitin protein ligase E3 component n-recognin 1		**-2.1**
UBTF	upstream binding transcription factor, RNA polymerase I	**-1.3**	**-1.2**
UCHL3	ubiquitin carboxyl-terminal esterase L3 (ubiquitin thiolesterase)	**-1.7**	
UCHL5	ubiquitin carboxyl-terminal hydrolase L5	**-1.7**	**1.6**
UCK1	uridine-cytidine kinase 1	**-1.5**	**-1.5**
UCK2	uridine-cytidine kinase 2	**-1.4**	**-1.7**
UCKL1	uridine-cytidine kinase 1-like 1	**2.1**	
UCP2	uncoupling protein 2 (mitochondrial, proton carrier)		**1.3**
UCRC	ubiquinol-cytochrome c reductase complex (7.2 kD)	**-1.2**	
UEVLD	UEV and lactate/malate dehyrogenase domains	**1.4**	**-2.6**
UFD1L	ubiquitin fusion degradation 1 like (yeast)	**-1.4**	
UGCG	UDP-glucose ceramide glucosyltransferase	**1.2**	**-2.4**

UGCGL1	UDP-glucose ceramide glucosyltransferase-like 1	**-1.3**	
UGDH	UDP-glucose dehydrogenase	**-1.5**	
UGP2	UDP-glucose pyrophosphorylase 2	**1.3**	
UHMK1	U2AF homology motif (UHM) kinase 1		**-1.8**
UHRF1	ubiquitin-like, containing PHD and RING finger domains, 1		**-3.9**
ULBP2	UL16 binding protein 2	**-1.8**	
UMP-CMPK	cytidylate kinase		**1.3**
UMPS	uridine monophosphate synthetase (orotate phosphoribosyl transferase and orotidine-5'-decarboxylase)		**-1.3**
UPF2	UPF2 regulator of nonsense transcripts homolog (yeast)		**2.2**
UPF3A	UPF3 regulator of nonsense transcripts homolog A (yeast)		**-1.4**
UPF3B	UPF3 regulator of nonsense transcripts homolog B (yeast)	**-1.3**	
UQCR	ubiquinol-cytochrome c reductase, 6.4kDa subunit		**-1.3**
UQCRC1	ubiquinol-cytochrome c reductase core protein I	**-2.0**	
UQCRC2	ubiquinol-cytochrome c reductase core protein II		**-1.6**
UQCRFS1	ubiquinol-cytochrome c reductase, Rieske iron-sulfur polypeptide 1		**1.2**
UQCRH	ubiquinol-cytochrome c reductase hinge protein		**1.5**
USF2	upstream transcription factor 2, c-fos interacting		**-1.5**
USP1	ubiquitin specific peptidase 1		**-1.5**
USP10	ubiquitin specific peptidase 10	**-1.7**	
USP11	ubiquitin specific peptidase 11	**1.3**	**-1.6**
USP13	ubiquitin specific peptidase 13 (isopeptidase T-3)	**-1.7**	**-1.7**
USP14	ubiquitin specific peptidase 14 (tRNA-guanine transglycosylase)	**-1.3**	**1.3**
USP16	ubiquitin specific peptidase 16		**-1.4**
USP18	ubiquitin specific peptidase 18	**-2.6**	
USP2	ubiquitin specific peptidase 2		**33.8**
USP21	ubiquitin specific peptidase 21	**-1.4**	
USP24	ubiquitin specific peptidase 24	**1.3**	**1.4**
USP33	ubiquitin specific peptidase 33	**1.3**	**1.5**
USP37	ubiquitin specific peptidase 37	**-1.6**	**-1.6**
USP39	ubiquitin specific peptidase 39	**-1.4**	
USP4	ubiquitin specific peptidase 4 (proto-oncogene)	**1.3**	**-1.7**
USP6	ubiquitin specific peptidase 6 (Tre-2 oncogene)	**-1.3**	**1.4**
USP6NL	USP6 N-terminal like	**2.1**	
USP7	ubiquitin specific peptidase 7 (herpes virus-associated)		**-3.0**
USP8	ubiquitin specific peptidase 8	**1.5**	
USP9X	ubiquitin specific peptidase 9, X-linked	**1.4**	
UTRN	utrophin	**1.6**	**-1.4**
UTS2	urotensin 2		**13.2**
UVRAG	UV radiation resistance associated gene		**1.6**
UXT	ubiquitously-expressed transcript		**-1.4**
VAMP1	vesicle-associated membrane protein 1 (synaptobrevin 1)	**1.3**	
VAMP2	vesicle-associated membrane protein 2 (synaptobrevin 2)	**-1.3**	**-4.2**

VAMP3	vesicle-associated membrane protein 3 (cellubrevin)	**1.3**	**1.3**
VAMP4	vesicle-associated membrane protein 4		**-2.6**
VAMP8	vesicle-associated membrane protein 8 (endobrevin)		**2.0**
VAPA	VAMP (vesicle-associated membrane protein)-associated protein A, 33kDa		**1.3**
VARS	valyl-tRNA synthetase	**-2.1**	
VASP	vasodilator-stimulated phosphoprotein	**-1.3**	**1.8**
VAV2	vav 2 oncogene		**-1.9**
VAV3	vav 3 oncogene	**3.0**	**2.5**
VCL	vinculin	**-1.5**	**1.9**
VCP	valosin-containing protein	**-1.3**	**1.3**
VDAC1	voltage-dependent anion channel 1		**1.6**
VDAC2	voltage-dependent anion channel 2		**1.9**
VDAC3	voltage-dependent anion channel 3		**1.8**
VDP	vesicle docking protein p115		**2.0**
VDR	vitamin D (1,25- dihydroxyvitamin D3) receptor	**3.5**	**3.3**
VEGF	vascular endothelial growth factor A	**-2.4**	**8.2**
VEGFB	vascular endothelial growth factor B		**-2.5**
VGLL4	vestigial like 4 (Drosophila)		**1.3**
VIL2	villin 2 (ezrin)	**2.4**	**-2.2**
VIM	vimentin	**1.2**	**-275.9**
VLDLR	very low density lipoprotein receptor	**-1.2**	**-4.7**
VNN1	vanin 1		**4.6**
VPREB1	pre-B lymphocyte gene 1		**-171.4**
VPREB3	pre-B lymphocyte gene 3		**-6.1**
VPS11	vacuolar protein sorting 11 homolog (S. cerevisiae)		**1.6**
VPS18	vacuolar protein sorting 18 homolog (S. cerevisiae)		**1.4**
VPS24	vacuolar protein sorting 24 homolog (S. cerevisiae)		**1.5**
VPS26	vacuolar protein sorting 26 homolog A (yeast)	**1.3**	**1.5**
VPS28	vacuolar protein sorting 28 homolog (S. cerevisiae)	**1.6**	
VPS29	vacuolar protein sorting 29 homolog (S. cerevisiae)		**-1.3**
VPS36	vacuolar protein sorting 36 homolog (yeast)	**1.4**	**1.4**
VPS41	vacuolar protein sorting 41 homolog (S. cerevisiae)		**3.5**
VPS4A	vacuolar protein sorting 4 homolog A (S. cerevisiae)		**-1.3**
VTI1A	vesicle transport through interaction with t-SNAREs homolog 1A (yeast)		**2.2**
VTI1B	vesicle transport through interaction with t-SNAREs homolog 1B (yeast)	**1.3**	**1.7**
WAC	WW domain containing adaptor with coiled-coil		**3.9**
WARS	tryptophanyl-tRNA synthetase	**-3.3**	**-1.5**
WARS2	tryptophanyl tRNA synthetase 2 (mitochondrial)		**1.5**
WASF2	WAS protein family, member 2	**3.6**	**1.8**
WASF3	WAS protein family, member 3		**9.9**
WASL	Wiskott-Aldrich syndrome-like	**2.4**	**1.7**
WASPIP	WAS/WASL interacting protein family, member 1		**1.4**

WBP11	WW domain binding protein 11		**-2.1**
WBP4	WW domain binding protein 4 (formin binding protein 21)		**-1.2**
WBSCR1	eukaryotic translation initiation factor 4H		**-2.4**
WBSCR5	linker for activation of T cells family, member 2	**-2.4**	**-70.5**
WDFY3	WD repeat and FYVE domain containing 3	**1.3**	**2.6**
WDR1	WD repeat domain 1		**-2.5**
WDR12	WD repeat domain 12	**-2.5**	**1.5**
WDR39	cytosolic iron-sulfur protein assembly 1 homolog (S. cerevisiae)	**-1.9**	
WDR45	WD repeat domain 45	**1.8**	
WDR48	WD repeat domain 48	**1.7**	**-2.0**
WEE1	WEE1 homolog (S. pombe)	**1.2**	**-1.4**
WIT-1	Wilms tumor upstream neighbor 1		**-2.4**
WRN	Werner syndrome	**-1.6**	
WSB1	WD repeat and SOCS box-containing 1	**2.0**	**9.0**
WSB2	WD repeat and SOCS box-containing 2	**1.3**	**1.4**
WT1	Wilms tumor 1	**1.4**	**-1.6**
WTAP	Wilms tumor 1 associated protein	**1.7**	**-1.5**
WWOX	WW domain containing oxidoreductase	**-1.7**	**-1.4**
WWP1	WW domain containing E3 ubiquitin protein ligase 1	**1.6**	
WWP2	WW domain containing E3 ubiquitin protein ligase 2		**-1.5**
XAB1	XPA binding protein 1, GTPase		**-1.6**
XBP1	X-box binding protein 1	**1.4**	
XDH	xanthine dehydrogenase	**-2.2**	
XIST	X (inactive)-specific transcript		**-808.5**
XPMC2H	REX4, RNA exonuclease 4 homolog (S. cerevisiae)		**1.3**
XPNPEP1	X-prolyl aminopeptidase (aminopeptidase P) 1, soluble		**3.2**
XPO1	exportin 1 (CRM1 homolog, yeast)		**-1.4**
XPO5	exportin 5	**-2.0**	**1.8**
XPO7	exportin 7	**1.3**	**4.8**
XPOT	exportin, tRNA (nuclear export receptor for tRNAs)	**-3.2**	**1.6**
XRCC1	X-ray repair complementing defective repair in Chinese hamster cells 1		**-1.4**
XRCC3	X-ray repair complementing defective repair in Chinese hamster cells 3		**-1.7**
XRCC4	X-ray repair complementing defective repair in Chinese hamster cells 4	**1.4**	**-1.8**
XRCC5	X-ray repair complementing defective repair in Chinese hamster cells 5 (double-strand-break rejoining; Ku autoantigen, 80kDa)	**-1.7**	**-2.0**
XRN1	5'-3' exoribonuclease 1	**1.6**	
XYLT2	xylosyltransferase II		**4.8**
YAF2	YY1 associated factor 2	**1.9**	**1.7**
YARS	tyrosyl-tRNA synthetase	**-2.6**	**-1.9**
YEATS4	YEATS domain containing 4		**-1.4**
YES1	v-yes-1 Yamaguchi sarcoma viral oncogene homolog 1		**49.1**
YIF1	Yip1 interacting factor homolog A (S. cerevisiae)	**-1.5**	
YME1L1	YME1-like 1 (S. cerevisiae)	**-1.2**	**1.7**

YPEL3	yippee-like 3 (Drosophila)	**2.2**	**-1.9**
YT521	--	**2.0**	**-1.3**
YWHAE	tyrosine 3-monooxygenase/tryptophan 5-monooxygenase activation protein, epsilon polypeptide	**-1.6**	**-2.5**
YWHAG	tyrosine 3-monooxygenase/tryptophan 5-monooxygenase activation protein, gamma polypeptide	**-1.3**	**-1.3**
YWHAQ	tyrosine 3-monooxygenase/tryptophan 5-monooxygenase activation protein, theta polypeptide	**1.6**	**-1.7**
YWHAZ	tyrosine 3-monooxygenase/tryptophan 5-monooxygenase activation protein, zeta polypeptide	**1.7**	**-2.5**
YY1	YY1 transcription factor		**2.7**
YY1AP1	YY1 associated protein 1	**1.5**	
ZA20D2	zinc finger, AN1-type domain 5	**1.9**	
ZAK	sterile alpha motif and leucine zipper containing kinase AZK		**-1.4**
ZAP70	zeta-chain (TCR) associated protein kinase 70kDa		**-10.1**
ZBTB1	zinc finger and BTB domain containing 1		**-1.4**
ZBTB11	zinc finger and BTB domain containing 11	**-1.3**	
ZBTB16	zinc finger and BTB domain containing 16	**2.8**	**5.9**
ZC3H8	zinc finger CCCH-type containing 8	**-1.7**	
ZDHHC8	zinc finger, DHHC-type containing 8	**-1.9**	
ZFHX1B	zinc finger homeobox 1b		**-2.6**
ZFP260	zinc finger protein 260	**1.6**	**-1.2**
ZFP36	zinc finger protein 36, C3H type, homolog (mouse)	**3.2**	
ZFP36L1	zinc finger protein 36, C3H type-like 1		**-24.5**
ZFP36L2	zinc finger protein 36, C3H type-like 2	**1.8**	**2.2**
ZFX	zinc finger protein, X-linked		**-1.6**
ZFYVE16	zinc finger, FYVE domain containing 16	**1.6**	
ZFYVE20	zinc finger, FYVE domain containing 20		**-2.4**
ZHX1	zinc fingers and homeoboxes 1	**2.0**	
ZHX2	zinc fingers and homeoboxes 2	**1.3**	**-5.1**
ZHX3	zinc fingers and homeoboxes 3	**2.0**	**2.0**
ZIC1	Zic family member 1 (odd-paired homolog, Drosophila)	**1.5**	
ZIC2	Zic family member 2 (odd-paired homolog, Drosophila)	**2.3**	**-3.5**
ZMYND11	zinc finger, MYND domain containing 11	**1.3**	**2.6**
ZMYND19	zinc finger, MYND-type containing 19	**-3.1**	
ZNF124	zinc finger protein 124	**-1.4**	
ZNF140	zinc finger protein 140		**1.4**
ZNF143	zinc finger protein 143		**-1.4**
ZNF148	zinc finger protein 148	**1.8**	**1.8**
ZNF161	vascular endothelial zinc finger 1		**-1.9**
ZNF175	zinc finger protein 175		**2.9**
ZNF183	--		**1.4**
ZNF198	zinc finger, MYM-type 2	**-1.3**	**4.3**
ZNF202	zinc finger protein 202	**-1.8**	**-1.4**
ZNF207	zinc finger protein 207	**-1.4**	**2.4**
ZNF224	zinc finger protein 224	**-1.2**	**-1.6**

ZNF238	zinc finger protein 238		**-5.5**
ZNF24	zinc finger protein 24	**-1.2**	
ZNF259	zinc finger protein 259		**-1.2**
ZNF263	zinc finger protein 263	**-1.7**	**-1.7**
ZNF267	zinc finger protein 267		**1.9**
ZNF278	POZ (BTB) and AT hook containing zinc finger 1	**-1.4**	
ZNF281	zinc finger protein 281		**1.5**
ZNF291	zinc finger protein 291	**1.6**	**1.4**
ZNF318	zinc finger protein 318	**1.3**	**2.3**
ZNF323	zinc finger protein 323		**-1.8**
ZNF329	zinc finger protein 329		**1.8**
ZNF336	GDNF-inducible zinc finger protein 1		**-1.3**
ZNF33A	zinc finger protein 33A	**1.5**	**1.5**
ZNF350	zinc finger protein 350		**-4.2**
ZNF354A	zinc finger protein 354A		**2.6**
ZNF384	zinc finger protein 384	**-1.6**	**-1.6**
ZNF398	zinc finger protein 398		**-1.4**
ZNF42	myeloid zinc finger 1	**-1.4**	
ZNF423	zinc finger protein 423	**-2.2**	**-3.6**
ZNF443	zinc finger protein 443	**1.5**	
ZNF467	zinc finger protein 467	**1.7**	**14.2**
ZNF468	--		**-5.5**
ZNF496	zinc finger protein 496	**-1.4**	**2.2**
ZNF622	zinc finger protein 622		**1.3**
ZNF629	zinc finger protein 629	**-1.2**	
ZNF74	zinc finger protein 74	**-3.2**	
ZNF9	CCHC-type zinc finger, nucleic acid binding protein	**-1.4**	
ZNF91	zinc finger protein 91		**-2.2**
ZNFN1A1	IKAROS family zinc finger 1 (Ikaros)	**1.3**	**2.2**
ZNFN1A4	IKAROS family zinc finger 4 (Eos)	**-1.4**	**1.3**
ZNFN1A5	IKAROS family zinc finger 5 (Pegasus)		**1.4**
ZNRD1	zinc ribbon domain containing 1	**-1.8**	
ZRANB1	zinc finger, RAN-binding domain containing 1		**1.3**
ZRF1	zuotin related factor 1	**-1.5**	**1.4**
ZW10	ZW10, kinetochore associated, homolog (Drosophila)	**-1.4**	
ZWILCH	Zwilch, kinetochore associated, homolog (Drosophila)	**-1.5**	**-1.6**
ZWINT	ZW10 interactor	**-1.3**	
